# Enantioselective Catalytic Synthesis of α-Halogenated α-Aryl-β^2,2^-amino Acid Derivatives

**DOI:** 10.1021/acsorginorgau.1c00025

**Published:** 2021-09-24

**Authors:** Paul Zebrowski, Isabella Eder, Andreas Eitzinger, Sharath Chandra Mallojjala, Mario Waser

**Affiliations:** † Institute of Organic Chemistry, Johannes Kepler University Linz, Altenbergerstrasse 69, 4040 Linz, Austria; ‡ Department of Chemistry, State University of New York at Binghamton, Binghamton, New York 13902, United States

**Keywords:** Asymmetric Catalysis, Ammonium Salt Catalysis, Kinetic Resolution, DFT Calculations

## Abstract

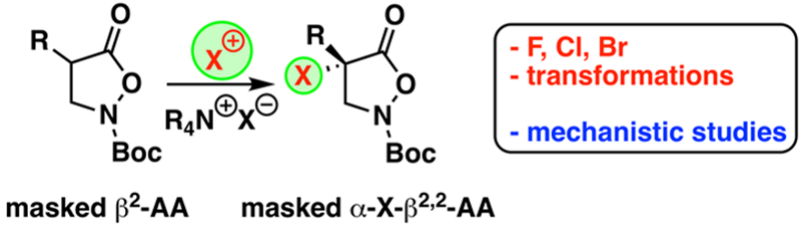

The enantioselective synthesis of a broad variety of novel differently functionalized α-halogenated α-aryl-β^2,2^-amino acid derivatives by means of an ammonium-salt-catalyzed asymmetric α-halogenation of isoxazolidin-5-ones was accomplished. Key to success to obtain high levels of enantioselectivities was the use of Maruoka’s spirocyclic binaphthyl-based ammonium salts, and detailed accompanying mechanistic studies using density functional theory methods revealed the key features for the catalyst–substrate interactions.

## Introduction

Investigations focusing on the asymmetric synthesis and further utilization of chiral non-natural amino acid derivatives have for decades been among the most prominent research topics in organic and bioorganic chemistry.^1−5^ A broad variety of conceptually different (catalytic) approaches to access non-natural amino acids (AA) with high levels of stereocontrol have been established, and the development of new synthesis strategies is still a highly contemporary field of research.^1,6−12^ In addition to the more classical focus on synthesis and applications of α-amino acids (α-AA) and α-AA-based peptides,^1−7^ non-natural β-AA have emerged as targets of significant interest over the past decades.^8−17^ The introduction of β-AA into the peptides, as well as the preparation of chiral β-AA-based heterocycles, can lead to peptidomimetics displaying unique (improved) biological properties,^8−17^ which makes the development of novel asymmetric approaches toward (masked) β-AA derivatives an important task.

**Scheme 1:**
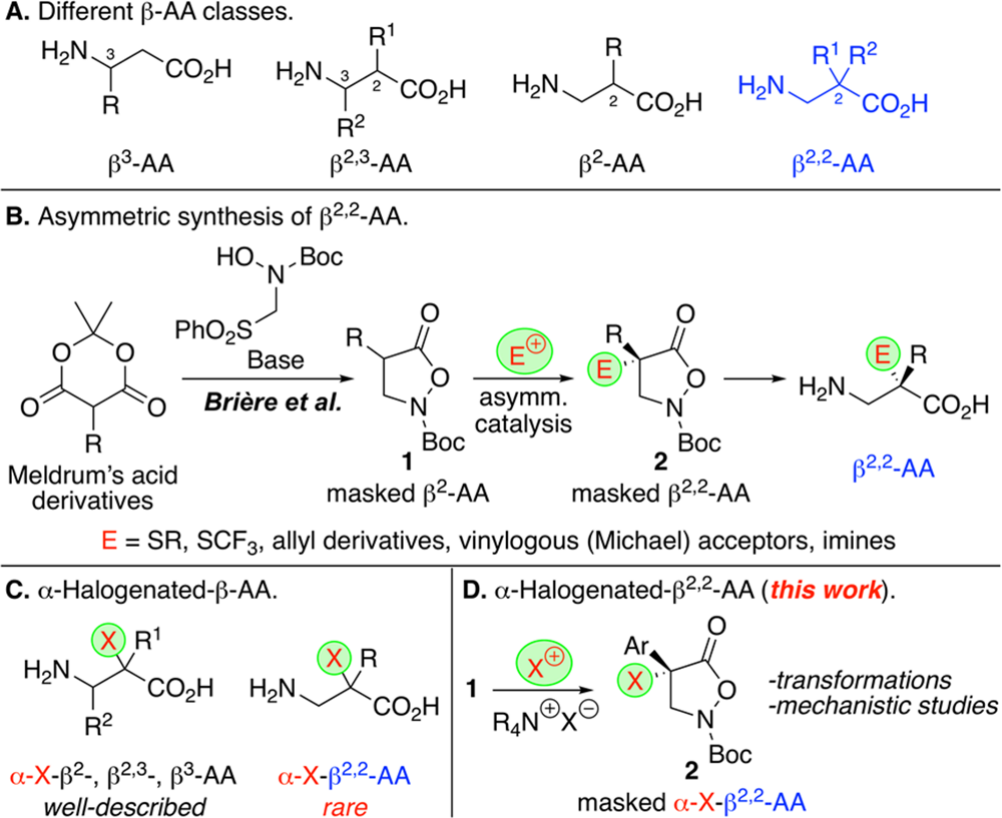
β-AA, Recently Established Strategy for β^2,2^-AA, α-Halogenated β-AA, and the Herein Investigated α-Halogenation of β^2,2^-AA Derivatives

Depending on their substitution pattern, different classes of β-AA can be defined (Scheme 1A). While several highly efficient strategies for the catalytic enantioselective synthesis of β^3^-, β^2,3^-, and β^2^-AA have been reported,^8−12^ the asymmetric construction of β^2,2^-AA remains challenging. In 2013, the Brière group reported the direct synthesis of isoxazolidin-5-ones **1** starting from Meldrum’s acid derivatives (Scheme 1B).^18^ Compounds **1** are versatile masked β^2^-AA derivatives which can be reacted in an asymmetric manner with different electrophiles to access the β^2,2^-AA derivatives **2** straightforwardly.^19−29^ These chiral heterocycles subsequently allow for the synthesis of free β^2,2^-AA and small peptides^20−29^ as well as for the synthesis of heterocyclic amino acids,^30,31^ to mention three potential applications only.

Over the past few years, this powerful concept has successfully been used for a handful of asymmetric C–C bond forming reactions (conjugate additions to classical Michael acceptors, MBH-carbonates, and quinone methides;^23−25^ Mannich-type reactions;^26,27^ Pd-catalyzed allylations^28,29^) as well as asymmetric α-sulfanylations,^20^ α-trifluoromethylthiolations,^21,22^ and one α-amination example.^23^ Apart from these few recent reports, however, the suitability of compounds **1** to access a broader variety of α-(hetero)-functionalized β^2,2^-AA has so far not systematically been explored.

The asymmetric α-heterofunctionalization of amino acids^32^ has been a very versatile strategy to access novel AA derivatives with promising biological properties or may serve as useful building blocks for further manipulations. Interestingly, while asymmetric approaches toward α-halogenated β^3^-, β^2,3^-, and β^2^-AA have been well-described,^32^ stereoselective syntheses of α-halogenated β^2,2^-AA have so far very sparingly been reported^33−37^ (Scheme 1C). Considering the unique potential of compounds **1** to serve as precursors for novel masked β^2,2^-AA derivatives **2**, we now became interested in exploring the suitability of compounds **1** for asymmetric α-halogenation reactions with different electrophilic halogen-transfer agents. This should give access to a new family of so far unprecedented α-halogenated α-arylated-β^2,2^-AA in a unique and direct manner by utilizing the easily available starting materials **1**. Based on our own previous experience with compounds **1**,^22,24,25^ as well as taking inspiration from Brière’s early reports,^20,23^ we focused on the use of chiral ammonium salt ion pairing catalysts^38−42^ to control compounds **1** in the herein targeted asymmetric α-halogenation approaches (chiral ammonium salt catalysts were also successfully used by Della Sala and Alemán for α-trifluoromethylthiolations of compounds **1**(21)). In addition, we also thought about gathering a deeper understanding of these reactions by carrying out detailed density functional theory (DFT) studies within the context of this project.

## Results and Discussion

### Asymmetric α-Chlorination

**Figure 1:**
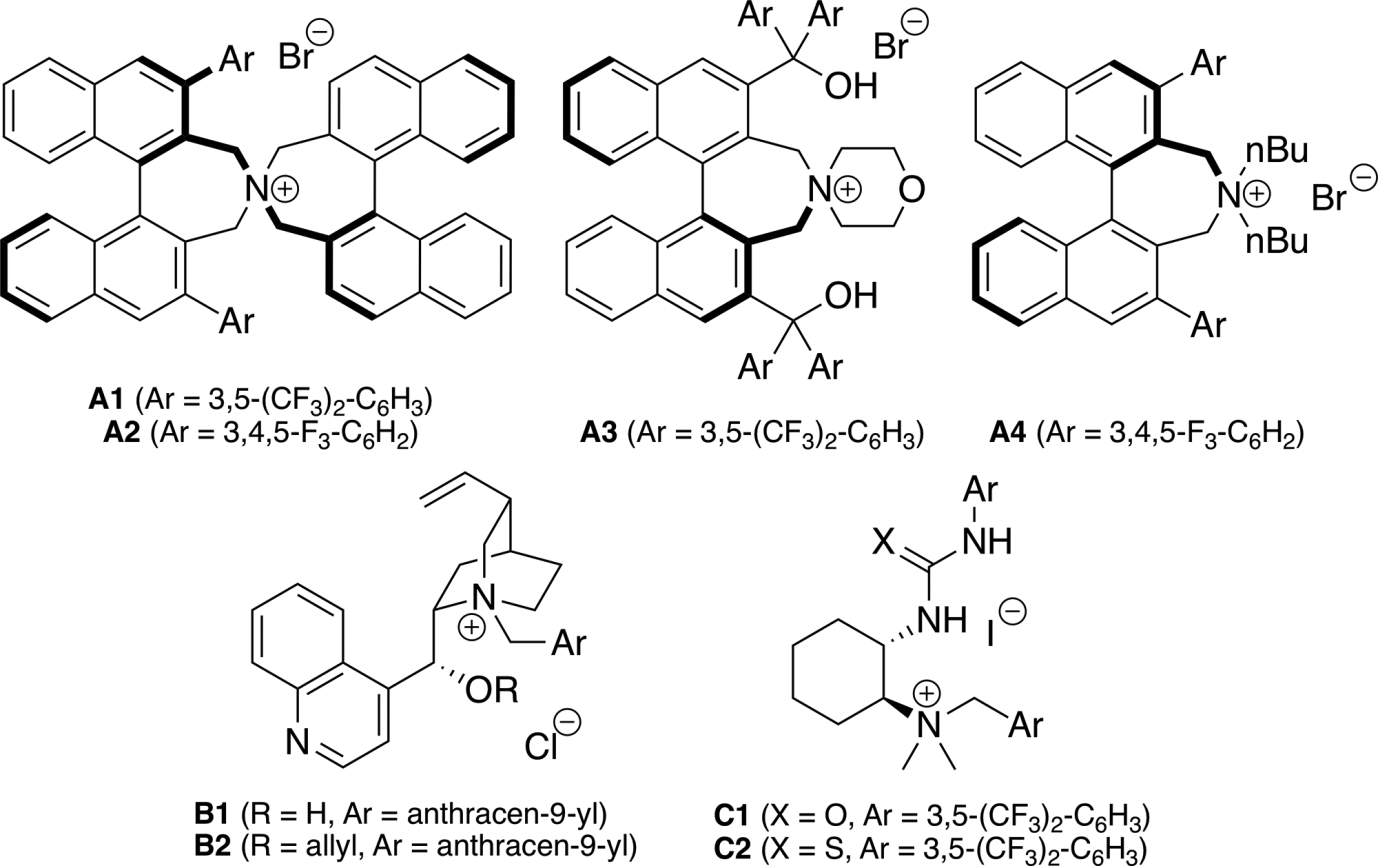
Chiral ammonium salt ion pairing catalysts tested for the asymmetric α-halogenations of compounds **1**.

The synthesis of chiral α-Cl-β^2,2^-AA has been very sparingly reported so far,^37,43,44^ and to the best of our knowledge, a reliable asymmetric catalysis approach to access (masked) α-Cl-β^2,2^-AA derivatives is yet missing. Considering the general value of enantioenriched α-Cl-carbonyl compounds to serve as building blocks for further manipulations (i.e., stereospecific S_N_2-type reactions),^45−47^ we now became interested in developing a protocol for the asymmetric electrophilic α-chlorination^48,49^ of isoxazolidin-5-ones **1** using the established chiral ammonium salt ion pairing catalysts **A**–**C** (Figure 1).

As summarized in Table 1, a variety of different conditions and catalysts were tested for the α-chlorination of the α-phenyl-substituted parent substrate **1a** using *N*-chlorosuccinimide (NCS, **3**) as a readily available and established electrophilic Cl-transfer agent.^48,49^ Based on the recently observed privileged application potential of Maruoka’s spirocyclic ammonium salt catalysts **A1** and **A2**(50) for asymmetric transformations of isoxazolidin-5-ones **1**,^20−25^ we started our screening using 5 mol % of the ammonium salt **A1** (*R*,*R*-configuration as depicted in Figure 1) in toluene in the presence of different mild bases (entries 1–3). Gratifyingly, in all cases, a complete conversion of **1a** was observed, and the targeted product **2a**^**Cl**^ could be obtained in reasonable isolated yields and with promising initial enantioselectivities up to 85:15 (favoring the (+)-isomer; please see the discussion below concerning the assignment of the depicted *S*-configuration). A further screening of different carbonate bases in different solvents did not allow for any improvement (results not given in the table), and in some cases, we also observed formation of the elimination product **4**. Surprisingly, however (considering our previous observations with compounds **1** where weaker inorganic bases were beneficial^20−25^), it was possible to obtain **2a**^**Cl**^ with a high er of 94:6 when using sodium phenoxide (PhONa) as a base instead (entry 4).^51^ Interestingly, despite the fact that we observed full conversion of **1a**, product **2a**^**Cl**^ could only be obtained in around 50% isolated yield, accompanied by formation of a, at this time not characterized, hardly soluble white precipitate. Initially, we suspected a problem with elimination and decomposition of product **2a**^**Cl**^ in the presence of this base as well as homogenization difficulties of the base in toluene. We therefore tested the use of an ultrasonic bath, different temperatures and reaction times, and order of addition of reagents (conditions A vs conditions B) next (entries 4–8). The overall transformation turned out to be much faster when carried out in an ultrasonic bath with more or less identical yield and er (entry 5). To achieve a better mixing and homogenization without using an ultrasonic bath, we next tested the stepwise addition of reagents (conditions B, entry 6). In addition, we also reduced the amount of the valuable catalyst to 2 mol % for the further optimization. Interestingly, even with this lower amount of catalyst, a full conversion of starting material **1a** was observed within 4 h under these conditions, and product **2a**^**Cl**^ was again obtained in around 50% isolated yield with a reasonable er of 91:9. Surprisingly, the reaction as such was found to be relatively clean, with no formation of elimination product **4**, and no other significant byproducts were observable in the crude product ^1^H NMR spectrum (recorded in CDCl_3_). Thus, we had a closer look on the hardly soluble precipitate that forms during this reaction and which was so far assumed to contain succinimide **3**- or phenoxide-originating byproducts. This precipitate was separated during workup by means of a simple filtration and was found to be insoluble in CDCl_3_. In contrast, however, it was well-soluble in H_2_O and DMSO and, upon closer analysis, was identified as the (relatively instable) acid **5a**^**Cl**^. Mechanistically, this compound most likely gets formed by ring-opening addition of phenoxide to **2a**^**Cl**^ followed by subsequent hydrolysis of the phenylester of **5a**^**Cl**^. It should be noted that we were not able to isolate this postulated phenylester, but when we tested the stability and reactivity of isolated **2a**^**Cl**^ in the presence of catalyst **A1** and different bases (including hydroxides), we realized that the nucleophilic PhONa was the only one which allowed for the formation of **5a**^**Cl**^, whereas other bases led to quantitative formation of the elimination product **4** only.

**Table 1:**
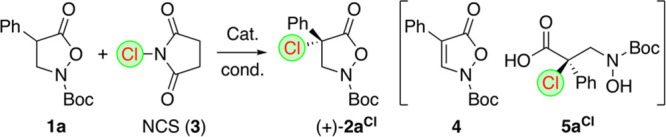
Optimization of the Asymmetric α-Chlorination of Isoxazolidin-5-one **1a**^a^

entry	cat. (mol %)	solvent	base (equiv)	condition^b^	*t* (h)	conv. (%)^c^	yield (%)^d^	er^e^
1	**A1** (5)	toluene	K_2_CO_3_ (1.2)	A	18	100	64	85:15
2	**A1** (5)	toluene	K_2_HPO_4_ (1.2)	A	18	100	82	80:20
3	**A1** (5)	toluene	NaOAc (1.2)	A	18	100	90	81:19
4	**A1** (5)	toluene	PhONa (1.1)	A	18	100	47	94:6
5^f^	**A1** (5)	toluene	PhONa (1.1)	A	1.5	100	53	93:7
6	**A1** (2)	toluene	PhONa (1.1)	B	4	100	53	91:9
7	**A1** (2)	toluene	PhONa (1.1)	B	6	100	32	97:3
8	**A1** (2)	toluene	PhONa (1.1)	B	17	100	25	99.5:0.5
9	**A1** (2)	toluene	4-NO_2_-C_6_H_4_ONa (1.1)	B	5	100	71	84:16
10	**A1** (2)	THF	PhONa (1.1)	B	4	100	34	81:19
11	**A1** (2)	Et_2_O	PhONa (1.1)	B	4	100	36	85:15
12	**A1** (5)	toluene	PhONa (0.5)	B	24	100	59	92:8
13	**A1** (5)	toluene	PhONa (0.5)	B	72	100	52 (33)^h^	95:5
14	**A1** (2)	toluene	PhONa (0.5)	B	72	100	46	91:9
15^g^	**A1** (5)	toluene	PhONa (0.5)	B	72	100	52	95:5
16	**A2** (5)	toluene	PhONa (0.5)	B	72	100	52	85:15
17	**A3** (5)	toluene	PhONa (0.5)	B	72	100	54	62:38
18	**A4** (5)	toluene	PhONa (0.5)	B	72	100	46	73:27
19	**B1** (5)	toluene	PhONa (0.5)	B	72	100	54	55:45
20	**B2** (5)	toluene	PhONa (0.5)	B	72	100	54	55:45
21	**C1** (5)	toluene	PhONa (0.5)	B	72	100	51	53:47
22	**C2** (5)	toluene	PhONa (0.5)	B	72	100	62	55:45

a Unless otherwise stated, all reactions were carried out at 25 °C in the indicated solvent using 0.1 mmol **1a** and 0.11 mmol **3** (0.05 M with respect to **1a**).

b Conditions A: **1a**, **3**, catalyst, and base were all placed in a flask followed by addition of the solvent. Conditions B: **1a**, **3**, and catalyst were dissolved in 50 vol % of the total solvent amount, followed by addition of a finely suspended mixture of the base (PhONa) in the remaining 50 vol % of the indicated solvent.

c Conversion of **1a** (determined by ^1^H NMR of the crude product mixture).

d Isolated yield of **2a**^**Cl**^ (after column chromatography).

e Given as (+)/(−)-**2a**^**Cl**^ ratio (determined by HPLC using a chiral stationary phase).

f Carried out in an ultrasonic bath.

g Carried out at 0 °C.

h Isolated yield of **5a**^**Cl**^.

Interestingly, Birman’s group recently reported a very appealing kinetic resolution (KR) of β-substituted isoxazolidin-5-ones with aliphatic alcohols in the presence of chiral squaramide catalysts, which gave access to various acyclic β^3^-AA esters in a mechanistically related manner.^52^ To probe if a conceptually similar KR may also account for our observations, we next treated racemic **2a**^**Cl**^ with 0.5 equiv of PhONa in the presence of catalyst **A1**, which resulted in the formation of **5a**^**Cl**^ and the recovery of enantioenriched (+)-**2a**^**Cl**^ (45% yield, er = 69:31, *s* = 3). In addition, when carrying out the overall α-chlorination protocol for prolonged reaction times with 1.1 equiv of PhONa (compare entries 6–8), the isolated yield of cyclic **2a**^**Cl**^ constantly decreased, combined with a significantly increasing enantiopurity up to er = 99.5:0.5 (entry 8), thus substantiating the involvement of a chiral ammonium-salt-catalyzed resolution step. Additionally, the use of the less nucleophilic 4-NO_2_-C_6_H_4_-ONa resulted in a higher **2a**^**Cl**^ yield but with lower selectivity (entry 9), which supports our proposal that the aryloxide serves as a nucleophile in the resolution step. Based on these results, it can therefore be postulated that the overall transformation most likely proceeds via two distinct steps, a relatively fast asymmetric ammonium-salt-catalyzed α-chlorination first, followed by a subsequent (slower) ammonium phenoxide-controlled kinetic resolution of the already enantioenriched **2a**. These two asymmetric processes match each other, resulting in a reasonably selective two-step one-pot strategy to access enantioenriched **2a**^**Cl**^ (overall, this reaction is best carried out in toluene while different ether solvents turned out to be not beneficial (entries 10 and 11)).

In our recent investigations concerning the asymmetric α-trifluoromethylthiolation of compounds **1** with succinimide or phthalimide-based SCF_3_-transfer reagents, we found that catalytic amounts of external bases may be sufficient, as the in situ formed succinimide or phthalimide can serve as a base, as well.^22^ Analogously, when we carried out the α-chlorination of **1a** with 10–20 mol % of K_2_CO_3_ only, we obtained the same yield and enantioselectivity as observed for the use of 1.2 equiv of this base (compare with entry 1), demonstrating that the α-chlorination step itself is a fast autocatalytic process where the nature of the external base has a less pronounced effect only. We therefore speculated that it should be possible to use only 50 mol % of the phenoxide (in order to primarily control the KR step), which should allow for a synthetically useful compromise between isolated yield and enantiopurity. In addition, control of the reaction progress/reaction time should be less critical compared to the use of an excess of phenoxide. As shown in entries 12 and 13, the use of 50 mol % of PhONa in the presence of 5 mol % of **A1** results in 59% isolated **2a**^**Cl**^ yield after 24 h already (er = 92:8) and allows for a further enantioenrichment when stirring for a prolonged reaction time (52% isolated yield in combination with a satisfying er of 95:5 after 3 days, entry 13). As expected, lowering the catalyst amount to 2 mol % had a slightly detrimental effect on the overall selectivity (entry 14), whereas lower temperatures had no influence at all (entry 15), provided the reaction was run long enough to allow for a satisfying progress of the KR (substantiating that the resolution step is the slower process in this two-step protocol). Finally, other ammonium salt catalysts **A**–**C** were tested, but in close analogy to previous observations,^20−25^ only the spirocyclic Maruoka ammonium salts **A1** and **A2** allowed for reasonable selectivities (compare entries 16–22), while the other well-established systems failed to allow for any reasonable selectivities.

**Scheme 2:**
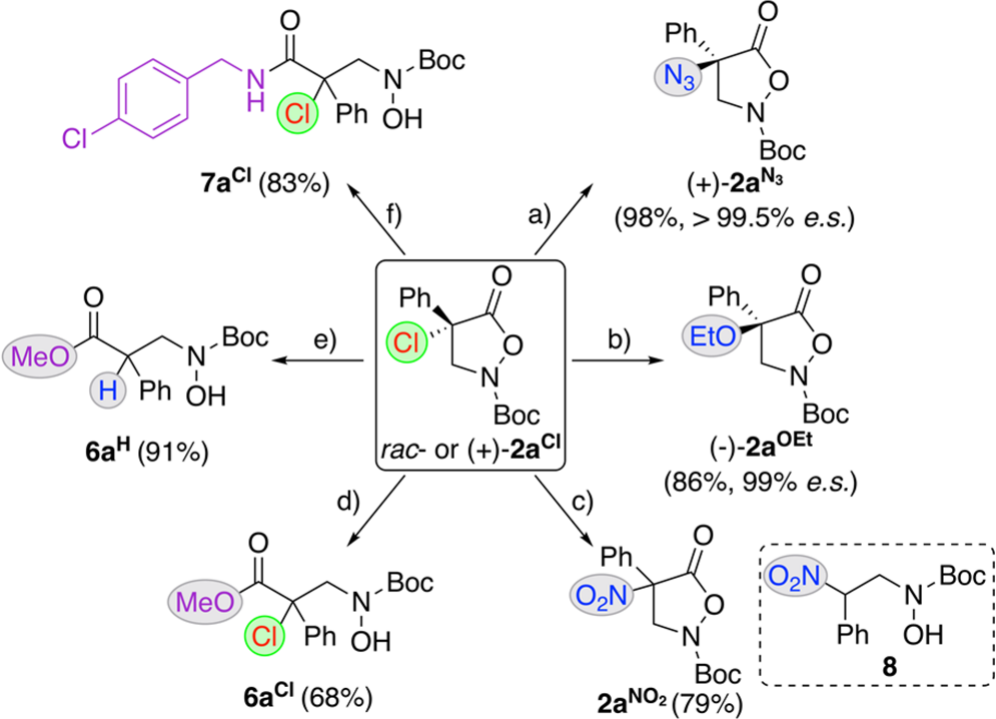
Further Manipulations of the Masked α-Chlorinated β^2,2^-AA Derivative **2a^Cl^** Conditions (a) NaN_3_ (1.1 equiv), acetone, 25 °C, 24 h [with (+)-**2a**^**Cl**^ (er = 99.0:0.5)]; (b) CHCl_3_ (0.6 vol % of EtOH), CsF (3 equiv), 18-crown-6, 25 °C, 1 h [with (+)-**2a**^**Cl**^ (er = 95:5)]; (c) NaNO_2_ (1.1 equiv), DMSO, 25 °C, 1 h [with *rac*-**2a**^**Cl**^]; (d) Y(OTf)_3_ (10 mol %), MeOH, 25 °C, 72 h [with *rac*-**2a**^**Cl**^]; (e) H_2_ (1 atm), Pd/C, MeOH, 25 °C, 24 h [with *rac*-**2a**^**Cl**^]; (f) 4-ClC_6_H_4_CH_2_NH_2_ (5 equiv), MeOH, 25 °C, 24 h [with *rac*-**2a**^**Cl**^].

Having identified reliable conditions for this combined α-chlorination–kinetic resolution approach to access enantioenriched **2a**^**Cl**^, we next investigated a series of further manipulations of this masked α-chlorinated β-AA derivative (Scheme 2). First, it was possible to directly replace the chlorine with a NO_2_ (product **2a**^**NO2**^), an EtO (product **2a**^**OEt**^), and a N_3_ group (product **2a**^**N3**^). Here, it should be noted that the α-NO_2_-containing **2a**^**NO2**^ turned out to be a fairly unstable compound, which rapidly undergoes ring opening and decarboxylation to compound **8** upon exposure to silica gel. In contrast, **2a**^**N3**^ and **2a**^**OEt**^ could be obtained with excellent levels of enantiospecificity when carrying out the nucleophilic S_N_2 displacement on enantioenriched **2a**^**Cl**^. In addition to these α-substitutions, it was also possible to carry out nucleophilic ring-opening reactions, as shown for the synthesis of the Me ester **6a**^**Cl**^ or the amide **7a**^**Cl**^ (analogous reactions could be carried out with **2a**^**N3**^, as well^53^). Interestingly, however, while it was possible to hydrogenate the N–O bond of other α,α-disubstituted isoxazolidinones **2** with classical Pd-catalyzed approaches (either with H_2_ or HCOONH_4_) in the past,^20−29^ this was not possible for **2a**^**Cl**^, as illustrated for the formation of the dehalogenated ester **6a**^**H**^ under established heterogeneous Pd-catalyzed hydrogenation conditions (other methods were tested as well, but we were not able to reduce the N–O bond without cleaving the C–Cl bond).

**Scheme 3:**
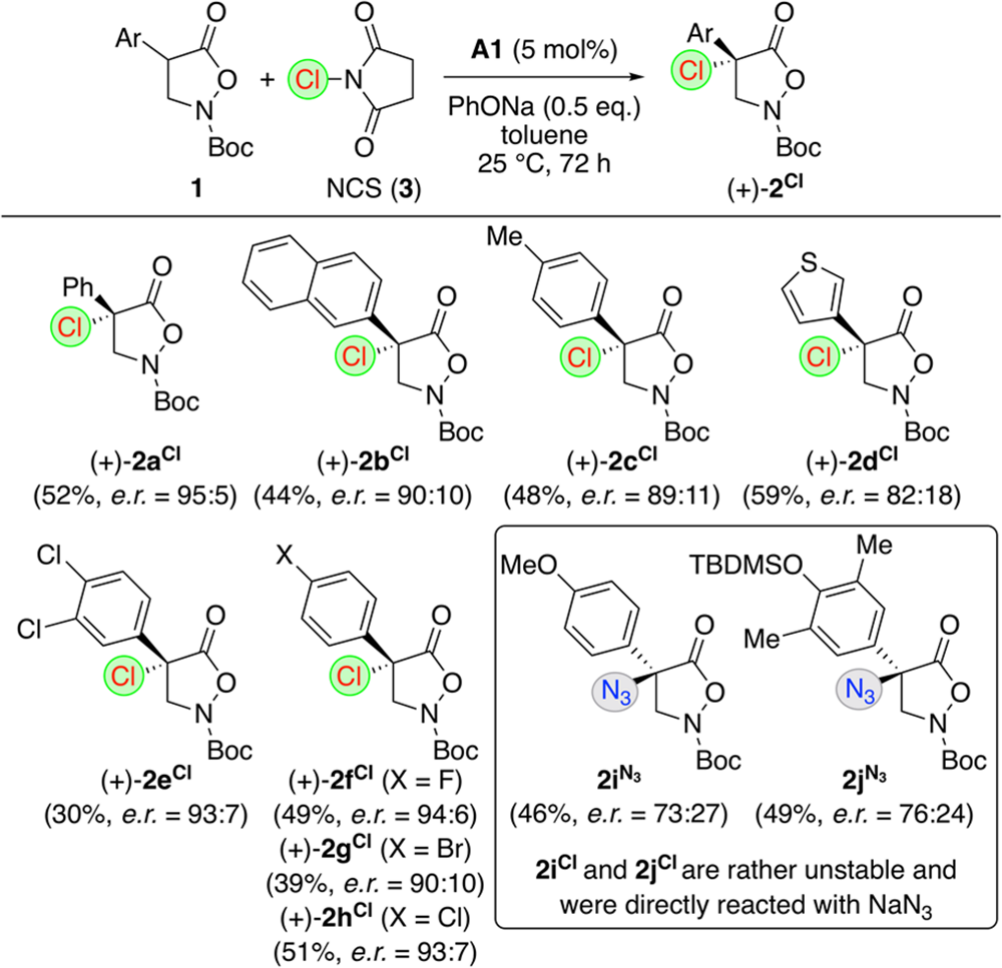
Asymmetric Application Scope for the Synthesis of Masked α-Chlorinated β^2,2^-AA Derivatives (+)-**2^Cl^**

When testing the α-chlorination of a variety of alternatively substituted α-arylated starting materials **1** under the optimized conditions next (Scheme 3), it turned out that this protocol, in general, tolerates different substitution patterns (like the halogenated derivatives **2e**^**Cl**^–**2h**^**Cl**^), but some interesting limitations also became obvious. The thiophene-containing **2d**^**Cl**^ was obtained with more than 50% yield but a lower enantioselectivity, indicating that the KR step is less efficient for this substrate as compared to others. In addition, the *p*-OMe- and *p*-OTBDMS-containing **2i**^**Cl**^ and **2j**^**Cl**^ could not be isolated as they decomposed very quickly, forming colored byproducts which most likely possess *p*-quinone methide-type structures. It was however possible to add NaN_3_ directly after completion of the α-chlorination, resulting in formation of the α-azidated products **2i**^**N3**^ and **2j**^**N3**^ instead. Unfortunately, enantioselectivities were not very high, which can be rationalized by a partial erosion of the enantiopurity of the primary reaction products **2i**^**Cl**^ and **2j**^**Cl**^ because of the aforementioned formation of quinone methide-type intermediates (to which NaN_3_ can add, as well^54,55^). It should be noted that we also tried to carry out this chlorination on α-alkyl-substituted derivatives **1** (e.g., Bn instead of Ar), but unfortunately, these turned out to be less reactive and gave trace amounts of the product only (the same outcome was obtained for the analogous fluorination reaction), which underscores the strong influence of the nature of the α-substituent on the reactivity of compounds **1**.

### Asymmetric α-Fluorination

Based on the knowledge gathered for the asymmetric α-chlorination of isoxazolidin-5-ones **1**, we next investigated the (analogous) α-fluorination of these compounds. Although a handful of previous reports described successful examples for the asymmetric synthesis of some α-F-β^2,2^-AA derivatives,^33−36^ the general enantioselective synthesis of these valuable targets is still far from being a solved challenge. Thus, we focused on the asymmetric ammonium-salt-catalyzed α-fluorination of the parent substrate **1a** with *N*-fluorobenzenesulfonimide (NFSI, **9**) as the electrophilic F-transfer agent next (Table 2 gives an overview about the most significant results obtained in a detailed screening of different catalysts and conditions). First attempts trying to apply our chlorination-inspired α-heterofunctionalization–kinetic resolution strategy with NaOPh failed, resulting in full decomposition of starting **1a**, without any product **2a**^**F**^ formation (entry 1). We next changed for “more common” asymmetric ammonium salt conditions using Cs_2_CO_3_ as a solid inorganic base. This allowed for a promising first hit, giving (+)-**2a**^**F**^ with reasonable conversion and a good er of 85:15 when using 5 mol % of the Maruoka catalyst **A1** in toluene (entry 2). Noteworthy, at this point, we already observed a rather pronounced sensitivity of product **2a**^**F**^ to prolonged exposure to base or acid (including silica gel), leading to formation of the elimination product **4** as well as other unidentified decomposition products. This made purification of **2a**^**F**^ a bit tricky, requiring either a rather fast column chromatographic isolation or recrystallization from cyclohexane to obtain **2a**^**F**^ in reasonable purity and yield (although some loss of material was observed, as well, especially after silica gel column chromatography). For that reason, we calculated in situ yields using an internal NMR standard in all cases and carried out further isolation attempts only once suited overall conditions were identified.

**Table 2:**
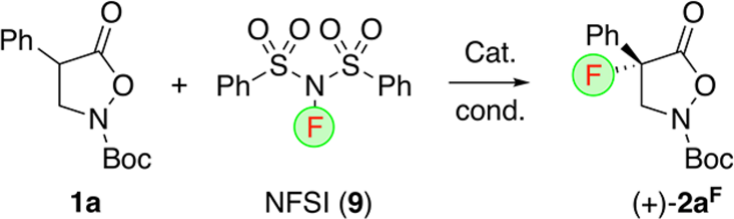
Optimization of the Asymmetric α-Fluorination of Isoxazolidin-5-one **1a**^a^

entry	cat. (mol %)	solvent	base (equiv)	*T* (°C)	*t* (h)	yield (%)^b^	er^c^
1	**A1** (5)	toluene	PhONa (1.1)	25	24		
2	**A1** (5)	toluene	Cs_2_CO_3_ (1.5)	25	24	65	85:15
3	**A1** (5)	MTBE	Cs_2_CO_3_ (1.5)	25	24	70	93:7
4	**A1** (5)	Et_2_O	Cs_2_CO_3_ (1.5)	25	24	75	92:8
5	**A1** (5)	Et_2_O	K_2_CO_3_ (1.5)	25	24	<5	
6	**A1** (10)	Et_2_O	Cs_2_CO_3_ (1.5)	25	65	85 (71^d^)	92:8
7	**A2** (10)	Et_2_O	Cs_2_CO_3_ (1.5)	25	65	60	91:9
8	**A1** (1)	Et_2_O	Cs_2_CO_3_ (1.5)	25	65	45	85:15
9	**A1** (5)	Et_2_O	Cs_2_CO_3_ (1.5)	–20	65	75	94:6
10	**A1** (5)	Et_2_O	Cs_2_CO_3_ (1.5)	–40	65	60	95:5
11	**A1** (5)	Et_2_O	Cs_2_CO_3_ (1.5)	–60	144	15	96:4
12	**A1** (5)	MTBE	Cs_2_CO_3_ (1.5)	25	40	85 (73^d^, 55^e^)	93:7

a Unless otherwise stated, all reactions were carried out using 0.1 mmol **1a** and 0.25 mmol **9** in the indicated solvent (0.017 M with respect to **1a**) under the given conditions.

b In situ yields of **2a**^**F**^ determined using 4-fluoroanisol as an internal NMR standard.

c Given as (+)/(−)-**2a**^**F**^ ratio (determined by HPLC using a chiral stationary phase); please see the discussion below concerning the assignment of the *S*-configuration for the (+)-enantiomer.

d Isolated yield after precipitation of excess of reagents, off-products, and catalyst with cyclohexane (containing less than 5 mol % of remaining diphenylsulfonimide, the given yield has been corrected for this “contamination”).

e Isolated yield after column chromatography.

Conversion and er using Cs_2_CO_3_ could be improved by changing for ethereal solvents next (entries 3 and 4). While MTBE allowed for a marginally higher er, reactions in Et_2_O showed a slightly better conversion, and a further screening of conditions was carried out in Et_2_O then. Other bases were tested, as well, but, as exemplified for K_2_CO_3_ (entry 5), turned out to be not suitable, and we therefore relied on Cs_2_CO_3_ for the remaining optimization (variations of reagent and base ratios were also tested but without any improvement). To increase yield and er, we next used 10 mol % of **A1** (65 h overall reaction time, entry 6). This allowed for a high er of 92:8 accompanied by a satisfying in situ yield of 85% and an isolated yield of 71% (after precipitation of reagents, off-products and catalyst with cyclohexane). Using other catalysts, the alternatively substituted Maruoka catalyst **A2** gave almost the same selectivity (entry 7), while all the other scaffolds shown in Figure 1 again gave more or less racemic **2a**^**F**^ only (results not given in Table 2). Lowering the catalyst loading to 1 mol % (entry 8) led to a reduced yield and selectivity, and we thus again used 5 mol % of **A1** for further attempts at lower temperatures (entries 9–11). Although it was possible to increase the er up to 96:4 at −60 °C, this increase in selectivity came with a significantly reduced conversion/yield. Therefore, to obtain a practical balance of yield and er, we finally opted for room temperature conditions and carried out the α-fluorination of **1a** in MTBE for a slightly prolonged reaction time of 40 h (entry 12). This allowed for the synthesis of (+)-**2a**^**F**^ in 85% in situ yield (isolated yields 73% after crystallization or 55% after column chromatography) and with an er of 93:7.

**Scheme 4:**
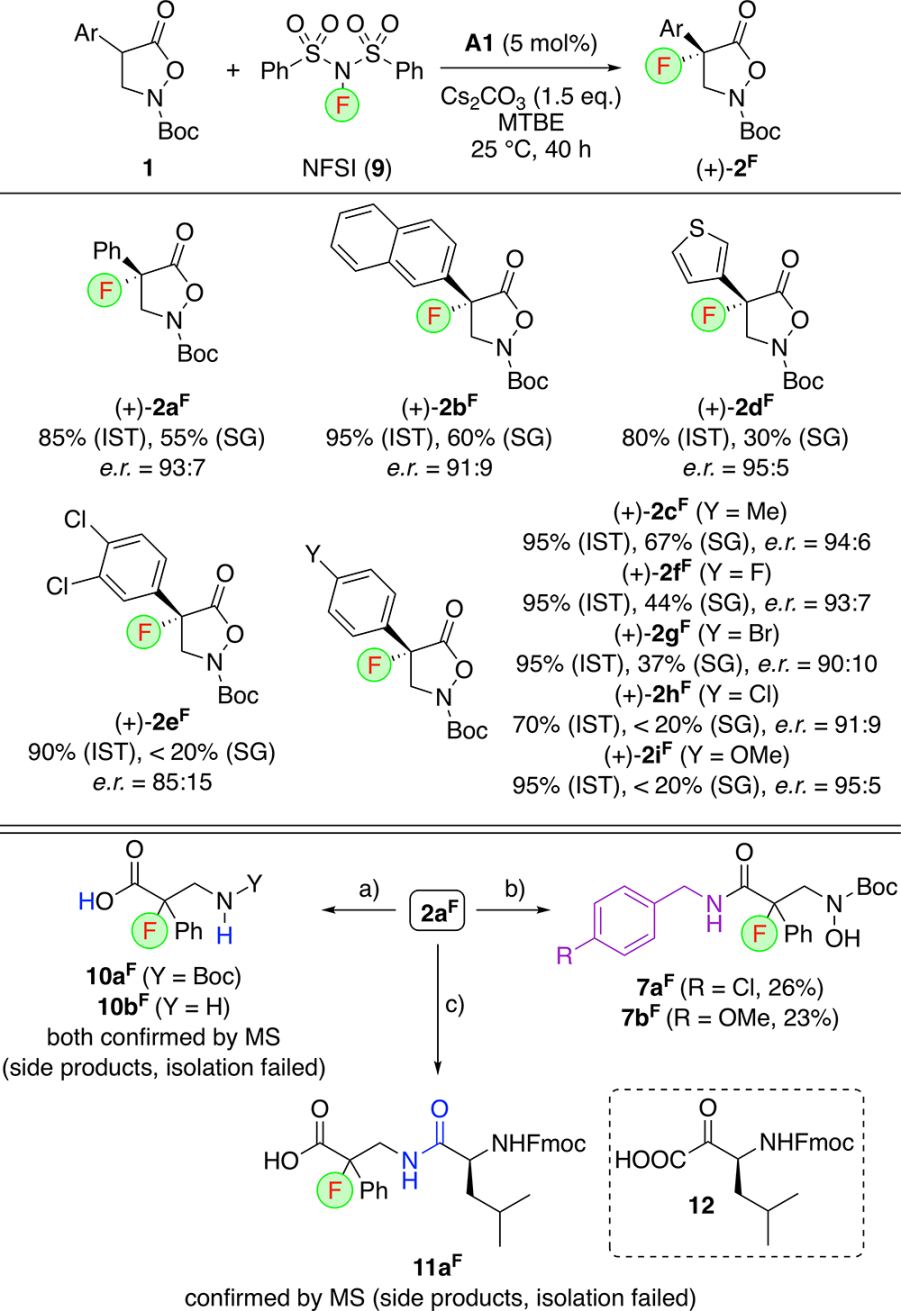
Asymmetric Application Scope for the Synthesis of Masked α-Fluorinated β^2,2^-AA Derivatives (+)-**2^F^** and (Attempted) Further Transformations, IST is the yield determined using 4-fluoroanisol as an internal NMR standard; SG is isolated yield after silica gel column chromatography. Conditions: (a) Different hydrogenation conditions (with or without previous TFA-mediated Boc-deprotection of **2a**^**F**^); (b) ArCH_2_NH_2_ in *t*-BuOH, 90 °C; (c) TFA in CH_2_Cl_2_ followed by addition of **12** in DMF.

With these conditions in hand, we next investigated the application scope for the α-fluorination of starting materials **1** and the suitability of products **2**^**F**^ for further manipulations (Scheme 4). A variety of different aryl substituents were well-tolerated, resulting in reasonable enantioselectivities and in situ yields for products **2**^**F**^. Unfortunately, the pronounced sensitivity of these compounds, however, made isolation by silica gel column chromatography difficult, especially for electron-rich aryl derivatives like **2i**^**F**^ (it should, however, be emphasized that we did not try to develop crystallization methods for each derivative as we did for the parent **2a**^**F**^).

Finally, we also investigated the use of the masked α-F-β-AA **2a**^**F**^ to carry out further transformations (Scheme 4, lower part). Hereby, we first investigated the reductive ring opening toward the free carboxylic acids **10** as well as the nucleophilic ring opening with benzylamine derivatives to access products **7**. While the latter could be isolated in relatively low yields (accompanied by decomposition of **2a**^**F**^ under the basic reaction conditions), formation of the acids **10** could only be detected by direct LRMS analysis of the crude products (which contained significant amounts of unspecified side products already), but all attempts to isolate these products failed. Similar results were unfortunately obtained when testing the well-established KAHA ligation of **2a**^**F**^ with the ketoacid **12**.^56,57^ Formation of the dipeptide **11a**^**F**^ could be confirmed by LRMS analysis, but again all attempts to isolate this interesting target failed because of its high sensitivity.

### Asymmetric α-Bromination and Stereochemical Considerations

**Scheme 5:**
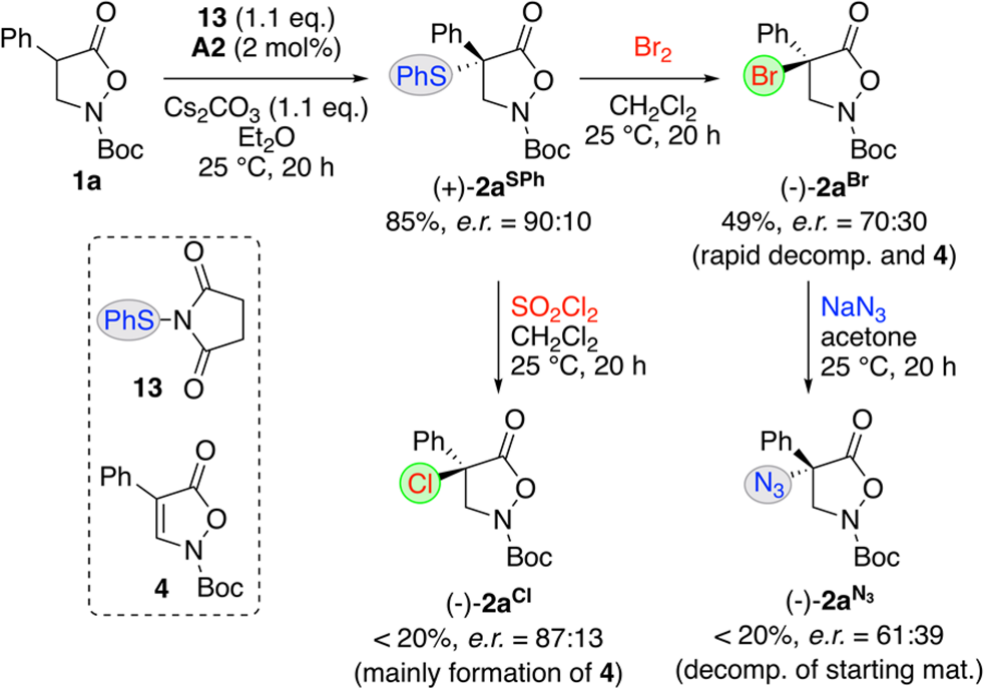
Asymmetric Sulfanylation and Stereospecific Desulfurylation–Halogenation Procedures

Having investigated the asymmetric synthesis of masked α-Cl and α-F-β^2,2^-AA derivatives **2**^**Cl**^ and **2**^**F**^ in much detail, we became interested in testing if analogous α-Br derivatives **2**^**Br**^ may be accessible, as well. Obviously, considering the observed sensitivity of compounds **2**^**Cl**^ and **2**^**F**^ under acidic and/or basic conditions, we expected an even more pronounced lability of the related Br target **2**^**Br**^. Thus, we were also not too much surprised that we did not succeed in carrying out the direct electrophilic α-bromination of the parent substrate **1a** with *N*-bromosuccinimide. Under several conditions that were tried, the starting material remained either unreacted or decomposed, and we therefore opted for an alternative approach to access **2a**^**Br**^ next. Recently the groups of Ibrahim and Adamo described the stereospecific S_N_2-type substitution of enantioenriched alkylphenylsulfides with Cl or Br,^58−60^ which provides an appealing entry to halogenated alkanes with good levels of stereocontrol (inversion of configuration). Inspired by these reports,^58−60^ and considering the fact that α-benzyl-substituted isoxazolidin-5-ones **1** were successfully α-sulfanylated under asymmetric ammonium salt catalysis by Brière before,^20^ we became interested if an asymmetric α-sulfanylation–desulfurylative bromination sequence may allow us to access the target α-Br derivative **2**^**Br**^. In analogy to Brière’s pioneering report,^20^ the α-sulfanylation of the phenyl-substituted **1a** could be carried out with good enantioselectivity with catalyst **A2** (using succinimide **13** as the PhS-transfer agent; Scheme 5). Gratifyingly, utilizing the reported desulfurylation–bromination conditions,^59^ it was possible to access (−)-**2a**^**Br**^ with good in situ yield and moderate levels of enantiospecificity (the loss in enantiopurity can be attributed to a rapid epimerization of product **2a**^**Br**^ under the reaction conditions). As expected, this compound turned out to be relatively unstable, resulting in the fast formation of unidentified decomposition products as well as in the elimination of HBr (giving alkene **4**), which made further purifications (e.g., by column chromatography) not possible. Interestingly, however, crude (−)-**2a**^**Br**^ can directly be reacted with NaN_3_ to access (−)-**2a**^**N3**^ under conditions similar to those established starting from (+)-**2a**^**Cl**^ already (which gave (+)-**2a**^**N3**^, as shown in Scheme 2).

The opposite sense of optical rotation (as well as HPLC retention orders) of products **2a**^**N3**^ obtained via these two different approaches clearly confirm the opposite absolute configuration of (−)-**2a**^**Br**^ relative to (+)-**2a**^**Cl**^ and (+)-**2a**^**F**^. In addition, it was also possible to convert (+)-**2a**^**SPh**^ into (−)-**2a**^**Cl**^ upon treatment with SO_2_Cl_2_. This process again proceeds with good enantiospecificity, and the optical rotations (as well as HPLC retention orders) of all these products accessed by different paths now confirm that (+)-**2a**^**SPh**^ as well as (+)-**2a**^**Cl**^ and (+)-**2a**^**F**^ prepared by means of an α-heterofunctionalization of **1a** in the presence of (*R*,*R*)-**A1** or (*R*,*R*)-**A2** have identical absolute configurations. This high level of catalyst-controlled face-selectivity, independent of the nature of the employed electrophile, is also in full accordance with previous observations.^20−25^ There, it was always found that the *R*,*R*-enantiomers of catalysts **A1** and **A2** efficiently block the *Re*-face of compounds **1** and thus favor *Si*-face approaches of the electrophiles (proven by single-crystal X-ray analysis for various enantiomerically enriched analogous products).^20−25^ Accordingly, when considering these earlier observations as well as the above-described chemical correlation, and based on our additional computational studies (vide infra), the absolute configuration of the major (+)-enantiomers of products **2**^**Cl,F,SPh**^ can be assigned to be *S*, despite of the fact that we were unfortunately not able to obtain crystals of enantioenriched products **2** suited for X-ray analysis.

### Computational Studies

**Figure 2:**
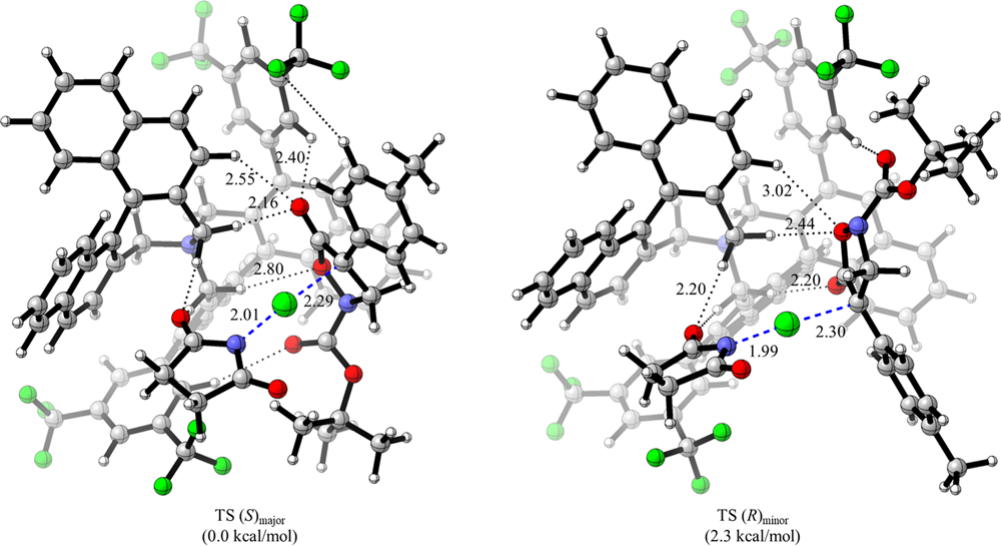
Competing enantiomeric TSS for the **A1**-catalyzed α-chlorination computed at PCM(toluene)-UFF:M062X/6-31+G(d,p)//PM7:B3LYP/6-31G*.

To better understand the origin of selectivity and to elaborate on the importance of the Maruoka catalysts **A** in catalyzing these reactions efficiently, we performed DFT studies on the chlorination and fluorination reactions catalyzed by **A1** (as well as the slightly less selective derivative **A2**(53)). In addition, these calculations will help us in further supporting the proposed absolute configurations for the favored enantiomers of products **2**. First, we modeled the competing major and minor enantiomeric transition state structures (TSS) for the **A1**-catalyzed chlorination reaction of **1c** with reagent **3**. In line with the outcome of our chemical correlation (vide supra) and previous observations,^20−25^ (*R*,*R*)-**A1** efficiently favors the *Si*-face chlorination of starting material **1c** (resulting in (*S*)-**2c**^**Cl**^). The lowest-lying TSS for the major *S*-enantiomer was found to be favored by 2.3 kcal/mol at 298 K (Figure 2). Closer inspection of the TSS revealed that the transferring electrophilic chlorine was found to nearly be at the same distance in both TSS. However, the TS(*S*)_major_ enjoys stronger hydrogen bonding interactions between the reactant fragments and the Maruoka ammonium catalyst compared to TS(*R*)_minor_.^61^ Distortion–interaction analysis decomposed the 2.6 kcal/mol electronic energy difference between the competing TSS into 1.1 kcal/mol of activation strain/distortion and 1.5 kcal/mol of interaction energy favoring the major enantiomer. Furthermore, decomposition of the interaction energy revealed strong electrostatic interactions (+1.8 kcal/mol) between the catalyst and the reactants favoring TS(*S*)_major_ (note that this electrostatic interaction was lower for the **A2**-catalyzed chlorination, thus substantiating the importance of the CF_3_ groups^53^)_._ Finally, TS(*S*)_major_ was found to enjoy dispersion interactions (0.5 kcal/mol) more favorable than those of TS(*R*)_minor_.

**Figure 3:**
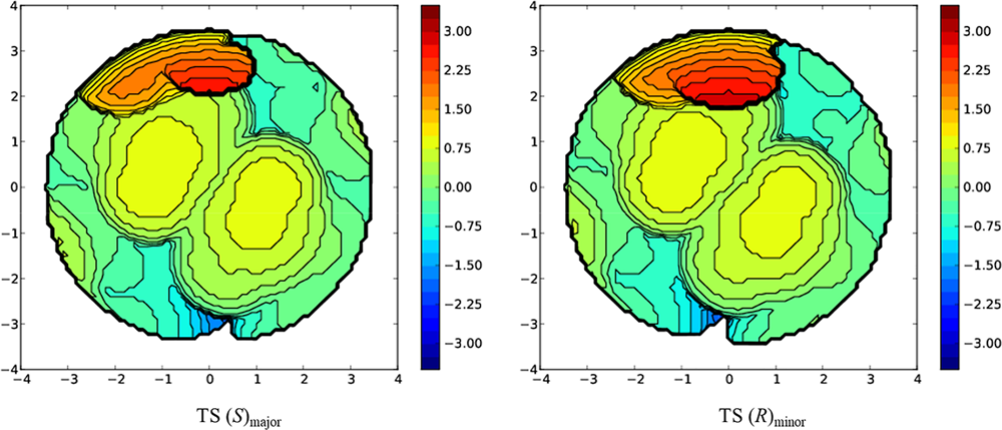
Buried volume plots for the major and minor enantiomeric TSS for the (*R*,*R*)-**A1**-catalyzed α-chlorination.

The steric cavity provided by the Maruoka catalyst was visualized by help from the SambVca algorithm (Figure 3). We observed that the TS leading to the major enantiomer suffered less steric interactions owing to a higher percentage of free volume (43.1% for TS(*S*)_major_ vs 42.7% for TS(*R*)_minor_) in the cavity. Furthermore, the area affected by the steric interactions of the catalyst arms indicated by area in red (Figure 3) is smaller in TS(*S*)_major_.

**Figure 4:**
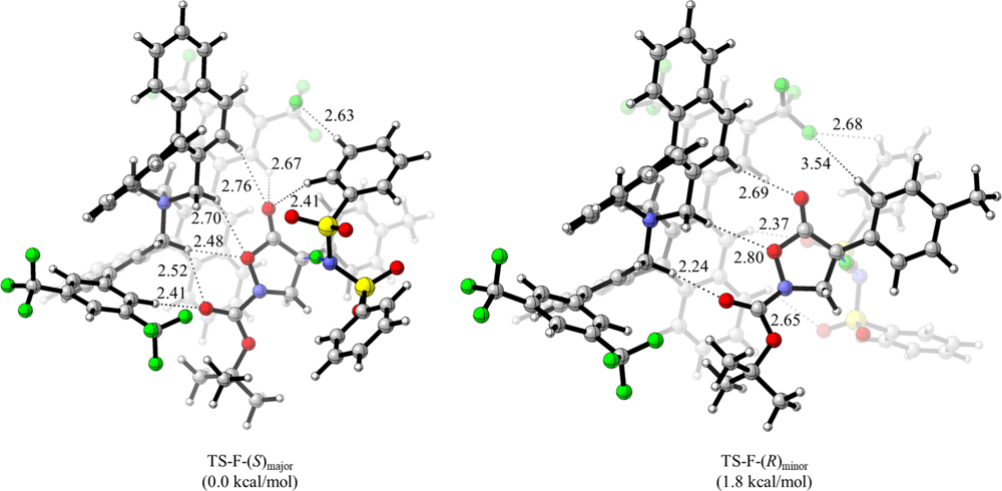
Competing enantiomeric TSS for the **A1**-catalyzed α-fluorination computed at PCM(diethyl ether)-UFF:M062X/6-31+G(d,p)//PM7:B3LYP/6-31G*.

Additionally, we also investigated the α-fluorination of **1c** catalyzed by (*R*,*R*)-**A1**. In line with the α-chlorination, computations clearly support the *Si*-face attack as well (favoring (*S*)-**2c**^**F**^), as TS-F-(*S*)_major_ was found to be favored by 1.8 kcal/mol over the minor enantiomer (Figure 4; this energy difference corresponds to a theoretical er = 96:4, which is slightly higher than the experimental outcome (er = 93:7)). Overall, similar key interactions between the catalyst and the substrates as observed for the chlorination were identified, thus underscoring the rather general activation mode of ammonium salt **A1** when used for asymmetric α-functionalizations of isoxazolidinones **1**.

A slightly reduced cavity volume was observed in the case of the **A1**-catalyzed fluorination reaction (44% for TS(*S*)_major_ vs 44.4% for TS(*R*)_minor_), presumably due to the slightly longer hydrogen bonding distances observed in the fluorination reaction. Based on these results, the *Si*-face preference by the Maruoka’s catalyst can be attributed to both the uniquely confined steric pocket generated by the arms of the biphenyl groups and the electrostatic environment generated by the electron-withdrawing substituents on the arms, as these groups were found to play a key role in modulating the hydrogen bond strength of the spirocyclic Maruoka-type catalysts.

## Conclusion

A detailed experimental and computational study on the enantioselective synthesis of (masked) α-halogenated β^2,2^-amino acid derivatives by means of asymmetric α-halogenation strategies of α-arylisoxazolidin-5-ones **1** has been carried out. High levels of enantioselectivities were possible by carrying out the electrophilic α-chlorination and α-fluorination in the presence of Maruoka’s spirocyclic binaphthyl-based ammonium salts. Noteworthy, while the α-fluorination followed a classical α-functionalization pathway, the α-chlorination protocol was most selective when carried out as a tandem process consisting of the electrophilic α-chlorination first, followed by a direct kinetic resolution via a nucleophilic ring opening. In addition, the α-bromination was possible, as well, via an alternative strategy by carrying out an enantioselective α-sulfanylation first, followed by a stereospecific desulfurylative bromination. All of the accessed targets **2** were investigated for their potential to undergo further manipulations. Moreover, detailed accompanying mechanistic studies using DFT methods revealed the key features for the catalyst–substrate interactions and provided an explanation for the high potential of the used catalysts to facilitate reactions of substrates **1** with a broad variety of electrophiles.

## Experimental Details

### General Methods

^1^H, ^13^C, and ^19^F NMR spectra were recorded on a Bruker Avance III 300 MHz spectrometer with a broad band observation probe and a sample changer for 16 samples, a Bruker Avance DRX 500 MHz spectrometer, and on a Bruker Avance III 700 MHz spectrometer with an Ascend magnet and TCI cryoprobe, which are both property of the Austro Czech NMR Research Center “RERI uasb”. NMR spectra were referenced on the solvent peak and chemical shifts are given in parts per million.

High-resolution mass spectra (HRMS) were obtained using a Thermo Fisher Scientific LTQ Orbitrap XL with an Ion Max API source. Analyses were made in the positive ionization mode if not otherwise stated. HPLC was performed using a Thermo Scientific Dionex Ultimate 3000 or a Shimadzu Prominence system with diode array detector with a CHIRALPAK AD-H, OD-H, CHIRAL ART amylose-SA or cellulose-SB (250 × 4.6 mm, 5 μm) chiral stationary phase. Optical rotations were recorded on a Schmidt + Haensch polarimeter model UniPol L1000 at 589 nm.

All chemicals were purchased from commercial suppliers and used without further purification unless otherwise stated. Starting materials **1** were synthesized as described previously.^20−22^ Dry solvents were obtained from an MBraun-SPS-800 solvent purification system. All reactions were carried out under argon atmosphere unless stated otherwise.

### General α-Chlorination Procedure

A flame-dried reaction vial was charged with catalyst **A1** (5.6 mg, 5 mol %), 4-aryl isoxazolidin-5-one **1** (0.1 mmol, 1.0 equiv), and dry toluene (1 mL). After complete dissolution, *N*-chlorosuccinimide **3** (15.1 mg, 1.1 equiv) and PhONa (5.9 mg, 0.5 equiv; finely suspended in 1 mL of toluene) were added successively. The reaction mixture was layered with argon and stirred for 72 h at room temperature. Afterward, the reaction was quenched by addition of saturated NH_4_Cl solution and diluted with EtOAc and H_2_O. The aqueous phase was extracted with EtOAc (3×), and the combined organic phases were washed with brine, dried over anhydrous Na_2_SO_4_, filtered, and concentrated under reduced pressure. The crude product was subjected to flash column chromatography (silica gel, heptanes/EtOAc) to obtain products **2**^**Cl**^ in the given yields and enantiopurities.

#### (+)-**2a**^**Cl**^:

Obtained by α-chlorination of **1a** (26.2 mg, 0.100 mmol) in 52% isolated yield (15.4 mg, 0.052 mmol) with er = 95:5; *R_f_* (heptanes/EtOAc = 5/1) = 0.37; [α]_D_^23^ = +64.2 (*c* = 1.0, CHCl_3_); ^1^H NMR (300 MHz, δ, CDCl_3_, 298 K) 7.64–7.60 (m, 2H), 7.49–7.40 (m, 3H), 4.86 (d, *J* = 13.2 Hz, 1H), 4.49 (d, *J* = 13.2 Hz, 1H), 1.51 (s, 9H); ^13^C NMR (75 MHz, δ, CDCl_3_, 298 K) 170.2, 156.1, 133.8, 130.3, 129.4 (2C), 127.2 (2C), 85.3, 64.5, 63.5, 28.2 (3C); HRMS (ESI) *m*/*z* [M + NH_4_]^+^ calcd for C_14_H_20_ClN_2_O_4_^+^ 315.1106; found 315.1113; HPLC (CHIRALCEL OD-H, eluent: hexanes/*i*-PrOH = 4/1, 0.5 mL/min, 10 °C) *t*_r_ = 14.2 min (major), 16.5 min (minor).

### General α-Fluorination Procedure

A flame-dried reaction vial was charged with *N*-fluorobenzenesulfonimide **9** (81.3 mg, 2.5 equiv), **A1** (5.6 mg, 5 mol %), Cs_2_CO_3_ (48.9 mg, 1.5 equiv), and 4-arylisoxazolidin-5-one **1** (0.1 mmol, 1 equiv). Then the vial was flushed with argon, and anhydrous MTBE (6 mL) was added counter-currently to the gas flow. After the reaction mixture was stirred at room temperature for 40 h, the mixture was filtered through a bed of Na_2_SO_4_ and washed with DCM, and the solvent was removed under reduced pressure. The crude product was purified via column chromatography (silica gel, heptanes/Et_2_O = 2/1) to obtain products **2**^**F**^ in the given yields and enantiopurities.

#### (+)-**2a**^**F**^:

Obtained from **1a** (26.3 mg, 0.100 mmol) in 85% NMR yield, 73% after precipitation of byproducts with cyclohexane, and 55% isolated yield after silica gel column chromatography (15.8 mg, 0.056 mmol) with er = 93:7; *R_f_* (heptanes/Et_2_O = 2/1) = 0.44; [α]_D_^22^ = +12.9 (*c* = 0.44, CHCl_3_); ^1^H NMR (300 MHz, δ, CDCl_3_, 298 K) 7.48 (s, 5H), 4.62 (dd, *J*_HF_ = 17.8 Hz, *J*_HH_ = 13.1 Hz, 1H), 4.44 (dd, *J*_HF_ = 21.9 Hz, *J*_HH_ = 13.1 Hz, 1H), 1.50 (s, 9H); ^13^C NMR (75 MHz, δ, CDCl_3_, 298 K) 169.1 (d, *J*_CF_ = 25.5 Hz), 155.9, 132.4 (d, *J*_CF_ = 23.2 Hz), 130.5 (d, *J*_CF_ = 6.6 Hz), 129.2, 125.6 (d, *J*_CF_ = 6.6 Hz), 93.1 (d, *J*_CF_ = 190.1 Hz), 59.9 (d, *J*_CF_ = 26.7 Hz), 28.0; ^19^F NMR (282 MHz, δ, CDCl_3_, 298 K) −155.2 (dd, *J*_FH_ = 21.9 Hz, *J*_FH_ = 17.8 Hz); HRMS (ESI) *m*/*z* [M + NH_4_]^+^ calcd for C_14_H_20_FN_2_O_4_ 299.1401; found 299.1413; HPLC (YMC CHIRAL ART Cellulose-SA, eluent: hexane/*i*-PrOH = 100:1, 0.5 mL/min, 10 °C) *t*_r_ = 27.2 min (major), 35.2 min (minor).

## Supporting Information Available

Experimental and analytical details of all new compounds, including copies of HPLC traces, as well as computational details (PDF)
Structure of (*R*)-minor (XYZ)

## Supplementary Material

gg1c00025_si_001.pdf

gg1c00025_si_002.xyz

## References

[ref1] Hughes A. B., Ed. Amino Acids, Peptides and Proteins in Organic Chemistry; Wiley-VCH, 2009; Vols. 1–5.

[ref2] Lang K.; Chin J. W. Cellular Incorporation of Unnatural Amino Acids and Bioorthogonal Labeling of Proteins. Chem. Rev. 2014, 114, 4764–4806. 10.1021/cr400355w. 24655057

[ref3] Maza J. C.; Jacobs T. H.; Uthappa D. M.; Young D. D. Employing Unnatural Amino Acids in the Preparation of Bioconjugates. Synlett 2016, 27, 805–813. 10.1055/s-0035-1560551.

[ref4] Nödling A. R.; Spear L. A.; Williams T. L.; Luk L. Y. P.; Tsai Y.-H. Using genetically incorporated unnatural amino acids to control protein functions in mammalian cells. Essays Biochem. 2019, 63, 237–266. 10.1042/EBC20180042. 31092687 PMC6610526

[ref5] Narancic T.; Almahboub S. A.; O’Connor K. E. Unnatural Amino Acids: Production and Biotechnological Potential. World J. Microbiol. Biotechnol. 2019, 35, 67 10.1007/s11274-019-2642-9. 30963257

[ref6] Najera C.; Sansano J. M. Catalytic Asymmetric Synthesis of α-Amino Acids. Chem. Rev. 2007, 107, 4584–4671. 10.1021/cr050580o. 17915933

[ref7] Williams R. M. Synthesis of Optically Active α-Amino Acids; Perlmutter P., Ed.; Tetrahedron Organic Chemistry; Pergamon, 2016; Vol. 7.

[ref8] Abele S.; Seebach D. Preparation of Achiral and of Enantiopure Geminally Disubstituted, beta-Amino Acids for beta-Peptide Synthesis. Eur. J. Org. Chem. 2000, 2000, 1–15. 10.1002/(SICI)1099-0690(200001)2000:1<1::AID-EJOC1>3.0.CO;2-6.

[ref9] Juaristi E., Soloshonok V. A., Eds. Enantioselective Synthesis of β-Amino Acids, 2nd ed.; John Wiley & Sons, 2005.

[ref10] Weiner B.; Szymanski W.; Janssen D. B.; Minnaard A. J.; Feringa B. L. Recent advances in the catalytic asymmetric synthesis of β-amino acids. Chem. Soc. Rev. 2010, 39, 1656–1691. 10.1039/b919599h. 20419214

[ref11] Ashfaq M.; Tabassum R.; Ahmad M. M.; Hassan N. A.; Oku H.; Rivera G. Enantioselective Synthesis of β-amino acids: A Review. Med. Chem. 2015, 5, 295–309. 10.4172/2161-0444.1000278.

[ref12] Noda H.; Shibasaki M. Recent Advances in the Catalytic Asymmetric Synthesis of β^2^- and β^2,2^-Amino Acids. Eur. J. Org. Chem. 2020, 2020, 2350–2361. 10.1002/ejoc.201901596.

[ref13] Cheng R. P.; Gellman S. H.; DeGrado W. F. β-Peptides: From structure to function. Chem. Rev. 2001, 101, 3219–3232. 10.1021/cr000045i. 11710070

[ref14] Lelais G.; Seebach D. β-Amino Acids-Syntheses, Occurrence in Natural Products, and Components of β-Peptides. Biopolymers 2004, 76, 206–243. 10.1002/bip.20088. 15148683

[ref15] Seebach D.; Gardiner J. β-Peptidic Peptidomimetics. Acc. Chem. Res. 2008, 41, 1366–1375. 10.1021/ar700263g. 18578513

[ref16] Seebach D.; Beck A. K.; Capone S.; Deniau G.; Grošelj U.; Zass E. Enantioselective Preparation of β^2^-Amino Acid Derivatives for β-Peptide Synthesis. Synthesis 2009, 2009, 1–32. 10.1055/s-0028-1087490.

[ref17] Cabrele C.; Martinek T. A.; Reiser O.; Berlicki Ł. Peptides Containing β-Amino Acid Patterns: Challenges and Successes in Medicinal Chemistry. J. Med. Chem. 2014, 57, 9718–9739. 10.1021/jm5010896. 25207470

[ref18] Tite T.; Sabbah M.; Levacher V.; Brière J.-F. Organocatalysed decarboxylative protonation process from Meldrum’s acid: enantioselective synthesis of isoxazolidinones. Chem. Commun. 2013, 49, 11569–11571. 10.1039/c3cc47695b. 24178176

[ref19] Macchia A.; Eitzinger A.; Brière J.-F.; Waser M.; Massa A. Asymmetric Synthesis of Isoxazol-5-ones and Isoxazolidin-5-ones. Synthesis 2021, 53, 107–122. 10.1055/s-0040-1706483.

[ref20] Cadart T.; Berthonneau C.; Levacher V.; Perrio S.; Brière J.-F. Enantioselective Phase-Transfer Catalyzed α-Sulfanylation of Isoxazolidin-5-ones: An Entry to β^2,2^-Amino Acid Derivatives. Chem. - Eur. J. 2016, 22, 15261–15264. 10.1002/chem.201603910. 27625021

[ref21] Capaccio V.; Sicignano M.; Rodríguez R. I.; Della Sala G.; Alemán J. Asymmetric Synthesis of α-Trifluoromethylthio-β-Amino Acids under Phase Transfer Catalysis. Org. Lett. 2020, 22, 219–223. 10.1021/acs.orglett.9b04195. 31833776

[ref22] Eitzinger A.; Brière J. F.; Cahard D.; Waser M. Enantioselective Catalytic Synthesis of α-Aryl-α-SCF_3_-β^2,2^-Amino Acids. Org. Biomol. Chem. 2020, 18, 405–408. 10.1039/C9OB02666E. 31915785 PMC6989214

[ref23] Cadart T.; Levacher V.; Perrio S.; Brière J.-F. Construction of Isoxazolidin-5-ones with a Tetrasubstituted Carbon Center: Enantioselective Conjugate Addition Mediated by Phase-Transfer Catalysis. Adv. Synth. Catal. 2018, 360, 1499–1509. 10.1002/adsc.201800009.

[ref24] Capaccio V.; Zielke K.; Eitzinger A.; Massa A.; Palombi L.; Faust K.; Waser M. Asymmetric phase-transfer catalysed β-addition of isoxazolidin-5-ones to MBH carbonates. Org. Chem. Front. 2018, 5, 3336–3340. 10.1039/C8QO01057A. 30505454 PMC6261335

[ref25] Eitzinger A.; Winter M.; Schörgenhumer J.; Waser M. Quaternary β^2,2^-Amino Acid Derivatives by Asymmetric Addition of Isoxazolidin-5-ones to para-Quinone Methides. Chem. Commun. 2020, 56, 579–582. 10.1039/C9CC09239K. PMC7082149 31830176

[ref26] Yu J.-S.; Noda H.; Shibasaki M. Exploiting β-Amino Acid Enolates in Direct Catalytic Diastereo- and Enantioselective C–C Bond-Forming Reactions. Chem. - Eur. J. 2018, 24, 15796–15800. 10.1002/chem.201804346. 30152580

[ref27] Amemiya F.; Noda H.; Shibasaki M. Lewis Base Assisted Lithium Brønsted Base Catalysis: A New Entry for Catalytic Asymmetric Synthesis of β^2,2^-Amino Acids. Chem. Pharm. Bull. 2019, 67, 1046–1049. 10.1248/cpb.c19-00569. 31341115

[ref28] Yu J.-S.; Noda H.; Shibasaki M. Quaternary β^2,2^-Amino Acids: Catalytic Asymmetric Synthesis and Incorporation into Peptides by Fmoc-Based Solid-Phase Peptide Synthesis. Angew. Chem., Int. Ed. 2018, 57, 818–822. 10.1002/anie.201711143. 29168280

[ref29] Nascimento de Oliveira M.; Arseniyadis S.; Cossy J. Palladium-Catalyzed Asymmetric Allylic Alkylation of 4-Substituted Isoxazolidin-5-ones: Straightforward Access to β^2,2^-Amino Acids. Chem. - Eur. J. 2018, 24, 4810–4814. 10.1002/chem.201800641. 29436035

[ref30] Yu J.-S.; Espinosa M.; Noda H.; Shibasaki M. Traceless Electrophilic Amination for the Synthesis of Unprotected Cyclic β-Amino Acids. J. Am. Chem. Soc. 2019, 141, 10530–10537. 10.1021/jacs.9b05476. 31188574

[ref31] Espinosa M.; Noda H.; Shibasaki M. Synthesis of Unprotected Spirocyclic β-Prolines and β-Homoprolines by Rh-Catalyzed C–H Insertion. Org. Lett. 2019, 21, 9296–9299. 10.1021/acs.orglett.9b03198. 31580682

[ref32] Eder I.; Haider V.; Zebrowski P.; Waser M. Recent Progress in the Asymmetric Syntheses of α-Heterofunctionalized (Masked) α- and β-Amino Acid Derivatives. Eur. J. Org. Chem. 2021, 2021, 202–219. 10.1002/ejoc.202001077.

[ref33] Avenoza A.; Busto J. H.; Corzana F.; Jimenez-Oses G.; Peregrina J. M. S_N_2 vs. E2 on quaternary centres: an application to the synthesis of enantiopure β^2,2^-amino acids. Chem. Commun. 2004, 980–981. 10.1039/B400282B. 15069504

[ref34] Edmonds M. K.; Graichen F. H. M.; Gardiner J.; Abell A. D. Enantioselective Synthesis of α-Fluorinated β^2^-Amino Acids. Org. Lett. 2008, 10, 885–887. 10.1021/ol703045z. 18232705

[ref35] Peddie V.; Pietsch M.; Bromfield K. M.; Pike R. N.; Duggan P. J.; Abell A. D. Fluorinated β^2^- and β^3^-Amino Acids: Synthesis and Inhibition of α-Chymotrypsin. Synthesis 2010, 2010, 1845–1859. 10.1055/s-0029-1218743.

[ref36] Sun A. W.; Hess S. N.; Stoltz B. M. Enantioselective synthesis of *gem*-disubstituted *N*-Boc diazaheterocycles via decarboxylative asymmetric allylic alkylation. Chem. Sci. 2019, 10, 788–792. 10.1039/C8SC03967D. 30774872 PMC6345351

[ref37] Lakner F. J.; Hager L. P. Chloroperoxidase-mediated asymmetric epoxidation. Synthesis of (*R*)-dimethyl 2-methvlaziridine-1,2-dicarboxvlate**—**a potential α-methylamino acid synthon. Tetrahedron: Asymmetry 1997, 8, 3547–3550. 10.1016/S0957-4166(97)00467-9.

[ref38] Shirakawa S.; Maruoka K. Recent Developments in Asymmetric Phase-Transfer Reactions. Angew. Chem., Int. Ed. 2013, 52, 4312–4348. 10.1002/anie.201206835. 23450630

[ref39] Tan J.; Yasuda N. Contemporary Asymmetric Phase Transfer Catalysis: Large-Scale Industrial Applications. Org. Process Res. Dev. 2015, 19, 1731–1746. 10.1021/acs.oprd.5b00304.

[ref40] Kaneko S.; Kumatabara Y.; Shirakawa S. A new generation of chiral phase-transfer catalysts. Org. Biomol. Chem. 2016, 14, 5367–5376. 10.1039/C5OB02446C. 26754659

[ref41] Schörgenhumer J.; Tiffner M.; Waser M. Chiral Phase-Transfer Catalysis in the Asymmetric α-Heterofunctionalization of Prochiral Nucleophiles. Beilstein J. Org. Chem. 2017, 13, 1753–1769. 10.3762/bjoc.13.170. 28904619 PMC5588627

[ref42] Qian D.; Sun J. Recent Progress in Asymmetric Ion-Pairing Catalysis with Ammonium Salts. Chem. - Eur. J. 2019, 25, 3740–3751. 10.1002/chem.201803752. 30358913

[ref43] Žukauskaitė A.; Mangelinckx S.; Šačkus A.; De Kimpe N. Synthesis of Alkyl 3-Chloroazetidine-3-carboxylates via Regioselective Ring Transformation of Alkyl 2-(Bromomethyl)aziridine-2-carboxylates. Heterocycles 2014, 88, 731–740. 10.3987/COM-13-S(S)35.

[ref44] Pu X.-Q.; Zhao H.-Y.; Lu Z.-H.; He X.-P.; Yang X.-J. Aminochlorination of Alkenes with CFBSA. Eur. J. Org. Chem. 2016, 2016, 4526–4533. 10.1002/ejoc.201600709.

[ref45] Shibatomi K.; Soga Y.; Narayama A.; Fujisawa I.; Iwasa S. Highly Enantioselective Chlorination of β-Keto Esters and Subsequent S_N_2 Displacement of Tertiary Chlorides: A Flexible Method for the Construction of Quaternary Stereogenic Centers. J. Am. Chem. Soc. 2012, 134, 9836–9839. 10.1021/ja304806j. 22651700

[ref46] Shibatomi K.; Kotozaki M.; Sasaki N.; Fujisawa I.; Iwasa S. Williamson Ether Synthesis with Phenols at a Tertiary Stereogenic Carbon: Formal Enantioselective Phenoxylation of β-Keto Esters. Chem. - Eur. J. 2015, 21, 14095–14098. 10.1002/chem.201502042. 26284459

[ref47] Liu R. Y.; Wasa M.; Jacobsen E. N. Enantioselective synthesis of tertiary α-chloro esters by non-covalent catalysis. Tetrahedron Lett. 2015, 56, 3428–3430. 10.1016/j.tetlet.2015.01.124. 26085694 PMC4465138

[ref48] Shibatomi K.; Narayama A. Catalytic Enantioselective α-Chlorination of Carbonyl Compounds. Asian J. Org. Chem. 2013, 2, 812–823. 10.1002/ajoc.201300058.

[ref49] Gomez-Martinez M.; Alonso D. A.; Pastor I. M.; Guillena G.; Baeza A. Organocatalyzed Assembly of Chlorinated Quaternary Stereogenic Centers. Asian J. Org. Chem. 2016, 5, 1428–1437. 10.1002/ajoc.201600404.

[ref50] Ooi T.; Kameda M.; Maruoka K. Molecular Design of a C2-Symmetric Chiral Phase-Transfer Catalyst for Practical Asymmetric Synthesis of α-Amino Acids. J. Am. Chem. Soc. 1999, 121, 6519–6520. 10.1021/ja991062w. 12708866

[ref51] Godemert J.; Oudeyer S.; Levacher V. Chiral Ammonium Aryloxides: Efficient Multipurpose Basic Organocatalysts. ChemCatChem 2016, 8, 74–85. 10.1002/cctc.201500616.

[ref52] Straub M. R.; Birman V. B. Organocatalytic Kinetic Resolution of N-Boc-Isoxazolidine-5-ones. Org. Lett. 2021, 23, 984–988. 10.1021/acs.orglett.0c04196. 33476151

[ref53] Further details can be found in the online Supporting Information.

[ref54] Lima C. G. S.; Pauli F. P.; Costa D. C. S.; de Souza A. S.; Forezi L. S. M.; Ferreira V. F.; de Carvalho da Silva F. para-Quinone Methides as Acceptors in 1,6-NucleophilicConjugate Addition Reactions for the Synthesis of Structurally Diverse Molecules. Eur. J. Org. Chem. 2020, 2020, 2650–2692. 10.1002/ejoc.201901796.

[ref55] Wang J.-Y.; Hao W.-J.; Tu S.-J.; Jiang B. Recent developments in 1,6-addition reactions of para-quinone methides (p-QMs). Org. Chem. Front. 2020, 7, 1743–1778. 10.1039/D0QO00387E.

[ref56] Bode J. W.; Fox R. M.; Baucom K. D. Chemoselective Amide Ligations by Decarboxylative Condensations of *N*-Alkylhydroxylamines and α-Ketoacids. Angew. Chem., Int. Ed. 2006, 45, 1248–1252. 10.1002/anie.200503991. 16416482

[ref57] Wucherpfennig T. G.; Pattabiraman V. R.; Limberg F. R. P.; Ruiz-Rodriguez J.; Bode J. W. Traceless Preparation of C-Terminal α-Ketoacids for Chemical Protein Synthesis by α-Ketoacid–Hydroxylamine Ligation: Synthesis of SUMO2/3. Angew. Chem., Int. Ed. 2014, 53, 12248–12252. 10.1002/anie.201407014. 25244549

[ref58] Canestrari D.; Lancianesi S.; Badiola E.; Strinna C.; Ibrahim H.; Adamo M. F. A. Desulfurative Chlorination of Alkyl Phenyl Sulfides. Org. Lett. 2017, 19, 918–921. 10.1021/acs.orglett.7b00077. 28151676

[ref59] Canestrari D.; Cioffi C.; Biancofiore I.; Lancianesi S.; Ghisu L.; Ruether M.; O’Brien J.; Adamo M. F. A.; Ibrahim H. Sulphide as a leaving group: highly stereoselective bromination of alkyl phenyl sulphides. Chem. Sci. 2019, 10, 9042–9050. 10.1039/C9SC03560E. 31827746 PMC6889150

[ref60] Alletto F.; Adamo M. F. A. Enantiospecific on-water bromination: a mild and efficient protocol for the preparation of alkyl bromides. Green Chem. 2020, 22, 8692–8698. 10.1039/D0GC02855J.

[ref61] Bencivenni G.; Salazar Illera D.; Moccia M.; Houk K. N.; Izzo J. A.; Novacek J.; Grieco P.; Vetticatt M. J.; Waser M.; Adamo M. F. A. Study of ground state interactions of enantiopure chiral quaternary ammonium salts and amides, nitroalkanes, nitroalkenes, esters, heterocycles, ketones and fluoroamides. Chem. - Eur. J. 2021, 27, 11352–11366. 10.1002/chem.202100908. 33963788 PMC8453964

