# Supplementary material for: Enantioselective Catalytic Synthesis of α-Halogenated α-Aryl-β2,2-amino Acid Derivatives
Source: ACS Org Inorg Au. 2021 Sep 24;2(1):34–43. doi: 10.1021/acsorginorgau.1c00025 (PMC8815071; doi:10.1021/acsorginorgau.1c00025)
Supplement: Supplementary file 1 — gg1c00025_si_001.pdf [file gg1c00025_si_001.pdf]

SUPPORTING INFORMATION

Enantioselective Catalytic Synthesis of α-halogenated-α-aryl-$\beta^{2,2}$-amino acid derivatives

Paul Zebrowski,[a] Isabella Eder,[a] Andreas Eitzinger,[a] Sharath Chandra Mallojjala,[b*] Mario Waser[a*]

[a] *Institute of Organic Chemistry, Johannes Kepler University Linz, Altenbergerstr. 69, 4040 Linz, Austria*

[b] *Department of Chemistry, State University of New York at Binghamton, Binghamton, New York-13902, United States*

Corresponding Authors

* Mario Waser − Institute of Organic Chemistry, Johannes Kepler University Linz, 4040 Linz, Austria; orcid.org/0000-0002-8421-8642;

Phone: +4373224685411; Email: mario.waser@jku.at

* Sharath Chandra Mallojjala – Department of Chemistry, State University of New York at Binghamton, Binghamton, New York-13902, United States, orcid.org/0000-0003-0446-792X, Email: sharathc@binghamton.edu.

List of Contents

1. General Information: 3
    1.1. General Methods 3
2. Syntheses and Analytic Details of Targets 2, 4, 5, 6, 7 and 8 4
    2.1 α-Chlorination 4
    2.2 α-Fluorination 8
    2.3 α-Sulfanylation - Bromination 12
    2.4 Further Transformations and Products 13
4. Copies of Product NMR Spectra 18
5. Copies of HPLC Chromatograms 58
6. Computational methods 81

1. General Information:

1.1. General Methods

^{1}H-, ^{13}C- and ^{19}F-NMR spectra were recorded on a Bruker Avance III 300 MHz spectrometer with a broad band observe probe and a sample changer for 16 samples, a Bruker Avance DRX 500 MHz spectrometer, and on a Bruker Avance III 700 MHz spectrometer with an Ascend magnet and TCI cryoprobe, which are both property of the Austro-Czech NMR-Research Center "RERI-uasb". NMR spectra were referenced on the solvent peak and chemical shifts are given in ppm.

High resolution mass spectra were obtained using a Thermo Fisher Scientific LTQ Orbitrap XL with an Ion Max API Source. Analyses were made in the positive ionization mode if not otherwise stated. Purine (exact mass for $[M+H]^+$ = 121.050873) and 1,2,3,4,5,6-hexakis(2,2,3,3-tetrafluoropropoxy)-1,3,5,2,4,6-triazatriphosphinane (exact mass for $[M+H]^+$ = 922.009798) were used for internal mass calibration.

HPLC was performed using a Thermo Scientific Dionex Ultimate 3000 or a Shimadzu Prominence system with diode array detector with a CHIRALPAK AD-H, OD-H, CHIRAL ART Amylose-SA or Cellulose-SB (250 × 4.6 mm, 5 µm) chiral stationary phase. Optical rotations were recorded on a Schmidt + Haensch Polarimeter Model UniPol L1000 at 589 nm.

All chemicals were purchased from commercial suppliers and used without further purification unless otherwise stated. Starting materials **1** were synthesized as described previously.[1-3] Dry solvents were obtained from an MBraun-SPS-800 solvent purification system. All reactions were carried out under argon atmosphere, unless stated otherwise.

[1] Cadart, T.; Berthonneau, C.; Levacher, V.; Perrio, S.; Brière, J.-F. Enantioselective Phase-Transfer Catalyzed α-Sulfanylation of Isoxazolidin-5-ones: An Entry to $\beta^{2,2}$-Amino Acid Derivatives, *Chem. Eur. J.* **2016**, *22*, 15261–15264.
[2] Capaccio, V.; Sicignano, M.; Rodríguez, R. I.; Della Sala, G.; Alemán, J. Asymmetric Synthesis of α-Trifluoromethylthio-β-Amino Acids under Phase Transfer Catalysis. *Org. Lett.* **2020**, *22*, 219-223.
[3] Eitzinger, A.; Brière, J. F.; Cahard, D.; Waser, M. Enantioselective Catalytic Synthesis of α-Aryl-α-SCF_3-$\beta^{2,2}$-Amino Acids. *Org Biomol Chem* **2020**, *18*, 405-408.

2. Syntheses and Analytic Details of Targets 2, 4, 5, 6, 7 and 8

2.1 α-Chlorination

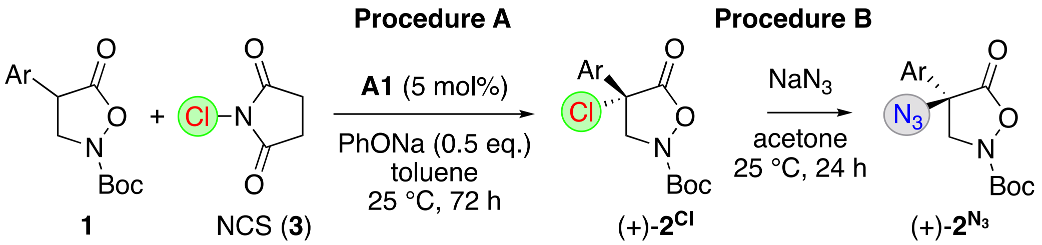

General Procedure A (Chlorination): A flame-dried reaction vial was charged with catalyst **A1** (5.6 mg, 5 mol%), 4-aryl-isoxazolidin-5-one **1** (0.1 mmol, 1.0 equiv) and dry toluene (1 mL). After complete dissolution, *N*-chlorosuccinimide **3** (15.1 mg, 1.1 equiv) and PhONa (5.9 mg, 0.5 equiv, finely-suspendended mixture in 1 mL toluene) were added successively. The reaction mixture was layered with argon and stirred for 72 h at room temperature, whereupon it was quenched with sat. NH_4Cl solution and diluted with EtOAc and H_2O. The aqueous phase was extracted with EtOAc (3x) and the combined organic phases were washed with brine, dried over anhydrous Na_2SO_4, filtered and concentrated under reduced pressure. The crude product was subjected to flash column chromatography (silica gel, heptanes/EtOAc) to obtain alpha-chloride **(+)-2^{Cl}** or used without any further purification in the azidation step.

General Procedure B (Azidation): The product of the chlorination step (1 equiv) was dissolved in acetone (0.05 M) and treated with NaN_3 (1.1 equiv). After stirring for 24 h at room temperature, the mixture was filtered through a bed of Celite, washed with DCM and concentrated under reduced pressure. The crude product was purified *via* flash column chromatography (silica gel, heptanes/EtOAc) to obtain alpha-azide **(+)-2^{N3}**.

Compound (+)-**2a^{Cl}**: Following procedure A, the α-chlorination of **1a** (26.2 mg, 0.100 mmol) gave (+)-**2a^{Cl}** in 52% isolated yield (15.4 mg, 0.052 mmol) with *e.r.* = 95:5. R_f (heptanes/EtOAc = 5/1) = 0.37. $[a]_D^{23}$ (c = 1.00, $CHCl_3$) = +64.2°. ^{1}H NMR (300 MHz, δ, $CDCl_3$, 298 K): 7.64–7.60 (m, 2H), 7.49–7.40 (m, 3H), 4.86 (d, *J* = 13.2 Hz, 1H), 4.49 (d, *J* = 13.2 Hz, 1H), 1.51 (s, 9H). ^{13}C NMR (75 MHz, δ, $CDCl_3$, 298 K): 170.2, 156.1, 133.8, 130.3, 129.4 (2C), 127.2 (2C), 85.3, 64.5, 63.5, 28.2 (3C). HRMS (ESI): calcd *m/z* for $C_{14}H_{20}ClN_2O_4^+$: 315.1106 $[M+NH_4]^+$; found: 315.1113. HPLC (CHIRALCEL® OD-H, eluent: hexane:*i*-PrOH = 4/1, 0.5 mL/min, 10 °C) retention times: t_{major} = 14.2 min, t_{minor} = 16.5 min.

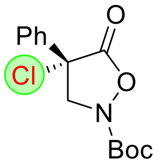

(+)-**2a^{Cl}**
52%, *e.r.* = 95:5

Compound (+)-**2b**^{Cl}: Following procedure A, the α-chlorination of **1b** (30.4 mg, 0.097 mmol) gave

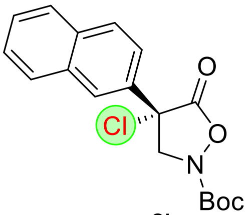

(+)-**2b**^{Cl}
44%, *e.r.* = 90:10

(+)-**2b**^{Cl} in 44% isolated yield (14.8 mg, 0.043 mmol) with *e.r.* = 90:10. R_f (heptanes/EtOAc = 5/1) = 0.38. $[a]_D^{23}$ (c = 0.47, $CHCl_3$) = +48.3°. ^1H NMR (300 MHz, δ, $CDCl_3$, 298 K): 8.10 (d, J = 2.0 Hz, 1H), 7.94 (d, J = 8.7 Hz, 1H), 7.90–7.85 (m, 2H), 7.68 (dd, J = 8.7, 2.0 Hz, 1H), 7.61–7.52 (m, 2H), 4.93 (d, J = 13.2 Hz, 1H), 4.62 (d, J = 13.2 Hz, 1H), 1.47 (s, 9H). ^{13}C NMR (75 MHz, δ, $CDCl_3$, 298 K): 170.3, 156.1, 133.8. 132.9, 130.8, 129.7, 128.9, 128.1, 128.0, 127.5, 127.0, 124.0, 85.3, 64.7, 63.5, 28.2 (3C). HRMS (ESI): calcd *m/z* for $C_{18}H_{22}ClN_2O_4^+$: 365.1263 $[M+NH_4]^+$; found: 365.1266. HPLC (YMC Chiral ART Cellulose-SB, eluent: hexane:*i*-PrOH = 20/1, 1.0 mL/min, 10 °C) retention times: t_{major} = 34.3 min, t_{minor} = 31.8 min.

Compound (+)-**2c**^{Cl}: Following procedure A, the α-chlorination of **1c** (28.0 mg, 0.101 mmol) gave

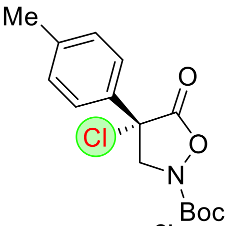

(+)-**2c**^{Cl}
48%, *e.r.* = 89:11

(+)-**2c**^{Cl} in 48% isolated yield (15.2 mg, 0.049 mmol) with *e.r.* = 89:11. R_f (heptanes/EtOAc = 4/1) = 0.47. $[a]_D^{23}$ (c = 0.69, $CHCl_3$) = +65.8°. ^1H NMR (300 MHz, δ, $CDCl_3$, 298 K): 7.51 (d, J = 8.3 Hz, 2H), 7.25 (d, J = 7.4 Hz, 2H), 4.84 (d, J = 13.2 Hz, 1H), 4.47 (d, J = 13.2 Hz, 1H), 2.38 (s, 3H), 1.51 (s, 9H). ^{13}C NMR (75 MHz, δ, $CDCl_3$, 298 K): 170.3, 156.2, 140.6, 130.8, 130.1 (2C), 127.2 (2C), 85.2, 64.4, 63.4, 28.2 (3C), 21.5. HRMS (ESI): calcd *m/z* for $C_{15}H_{22}ClN_2O_4^+$: 329.1263 $[M+NH_4]^+$; found: 329.1263. HPLC (YMC Chiral ART Cellulose-SB, eluent: hexane:*i*-PrOH = 100/1, 1.0 mL/min, 10 °C), retention times: t_{major} = 18.5 min, t_{minor} = 20.5 min.

Compound (+)-**2d**^{Cl}: Following procedure A, the α-chlorination of **1d** (27.2 mg, 0.101 mmol) gave

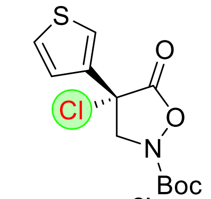

(+)-**2d**^{Cl}
59%, *e.r.* = 82:18

(+)-**2d**^{Cl} in 59% isolated yield (18.0 mg, 0.059 mmol) with *e.r.* = 82:18. R_f (heptanes/EtOAc = 5/1) = 0.37. $[a]_D^{23}$ (c = 0.83, $CHCl_3$) = +44.2°. ^1H NMR (300 MHz, δ, $CDCl_3$, 298 K): 7.64 (dd, J = 3.0, 1.3 Hz, 1H), 7.43 (dd, J = 5.1, 3.0 Hz, 1H), 7.28 (dd, J = 5.1, 1.3 Hz, 1H), 4.86 (d, J = 13.2 Hz, 1H), 4.48 (d, J = 13.2 Hz, 1H), 1.52 (s, 9H). ^{13}C NMR (75 MHz, δ, $CDCl_3$, 298 K): 169.9, 156.2, 134.0, 128.2, 126.4, 125.9, 85.4, 62.9, 60.9, 28.2 (3C). HRMS (ESI): calcd *m/z* for $C_{12}H_{18}ClN_2O_4S^+$: 321.0670 $[M+NH_4]^+$; found: 321.0675. HPLC (CHIRALCEL® OD-H, eluent: hexane:*i*-PrOH = 20/1, 1.0 mL/min, 10 °C), retention times: t_{major} = 15.0 min, t_{minor} = 13.6 min.

Compound (+)-**2e**^{Cl}: Following procedure A, the α-chlorination of **1e** (27.1 mg, 0.082 mmol) gave

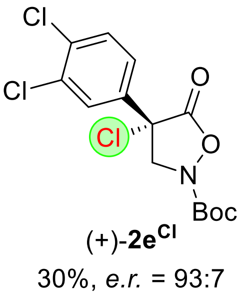

(+)-**2e**^{Cl}
30%, *e.r.* = 93:7

(+)-**2e**^{Cl} in 30% isolated yield (9.1 mg, 0.025 mmol) with *e.r.* = 93:7. R_f (heptanes/EtOAc = 4/1) = 0.47. $[a]_D^{23}$ (c = 0.33, $CHCl_3$) = +65.1°. ^1H NMR (700 MHz, δ, $CDCl_3$, 298 K): 7.75 (d, *J* = 2.3 Hz, 1H), 7.54 (d, *J* = 8.5 Hz, 1H), 7.48 (dd, *J* = 8.5, 2.3 Hz, 1H), 4.87 (d, *J* = 13.3 Hz, 1H), 4.40 (d, *J* = 13.3 Hz, 1H), 1.54 (s, 9H). ^{13}C NMR (176 MHz, δ, $CDCl_3$, 298 K): 169.4, 155.9, 135.1, 133.9, 133.8, 131.4, 129.5, 126.6, 85.7, 63.0 (2C), 28.3 (3C). HRMS (ESI): calcd *m/z* for $C_{14}H_{18}Cl_3N_2O_4^+$: 383.0327 $[M+NH_4]^+$; found: 383.0337. HPLC (CHIRALCEL® OD-H, eluent: hexane:*i*-PrOH = 4/1, 0.5 mL/min, 10 °C) retention times: t_{major} = 21.2 min, t_{minor} = 22.9 min.

Compound (+)-**2f**^{Cl}: Following procedure A, the α-chlorination of **1f** (28.2 mg, 0.100 mmol) gave

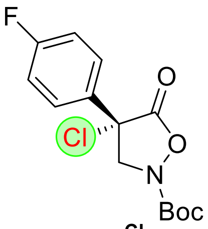

(+)-**2f**^{Cl}
49%, *e.r.* = 94:6

(+)-**2f**^{Cl} in 49% isolated yield (15.4 mg, 0.049 mmol) with *e.r.* = 94:6. R_f (heptanes/EtOAc = 4/1) = 0.46. $[a]_D^{23}$ (c = 0.75, $CHCl_3$) = +60.3°. ^1H NMR (300 MHz, δ, $CDCl_3$, 298 K): 7.68–7.61 (m, 2H), 7.18–7.11 (m, 2H), 4.87 (d, *J* = 13.2 Hz, 1H), 4.45 (d, *J* = 13.2 Hz, 1H), 1.52 (s, 9H). ^{13}C NMR (75 MHz, δ, $CDCl_3$, 298 K): 170.0, 163.7 (d, *J* = 251.5 Hz), 156.1, 129.7 (d, *J* = 3.6 Hz), 129.5 (d, *J* = 8.7 Hz, 2C), 116.6 (d, *J* = 22.1 Hz, 2C), 85.5, 63.6, 63.2, 28.2 (3C). ^{19}F NMR (282 MHz, δ, $CDCl_3$, 298 K): -110.1 (m). HRMS (ESI): calcd *m/z* for $C_{14}H_{19}ClFN_2O_4^+$: 333.1012 $[M+NH_4]^+$; found: 333.1011. HPLC (YMC Chiral ART Cellulose-SB, eluent: hexane:*i*-PrOH = 20/1, 1.0 mL/min, 10 °C), retention times: t_{major} = 13.4 min, t_{minor} = 12.1 min.

Compound (+)-**2g**^{Cl}: Following procedure A, the α-chlorination of **1g** (28.4 mg, 0.083 mmol) gave

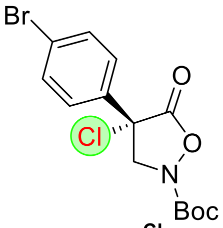

(+)-**2g**^{Cl}
39%, *e.r.* = 90:10

(+)-**2g**^{Cl} in 39% isolated yield (12.2 mg, 0.032 mmol) with *e.r.* = 90:10. R_f (heptanes/EtOAc = 5/1) = 0.40. $[a]_D^{23}$ (c = 0.21, $CHCl_3$) = +58.6°. ^1H NMR (300 MHz, δ, $CDCl_3$, 298 K): 7.62–7.57 (m, 2H), 7.53–7.49 (m, 2H), 4.86 (d, *J* = 13.2 Hz, 1H), 4.42 (d, *J* = 13.2 Hz, 1H), 1.52 (s, 9H). ^{13}C NMR (75 MHz, δ, $CDCl_3$, 298 K): 169.8, 156.0, 132.8, 132.7 (2C), 129.0 (2C), 124.9, 85.5, 63.7, 63.1, 28.2 (3C). HRMS (ESI): calcd *m/z* for $C_{14}H_{19}BrClN_2O_4^+$: 393.0211 $[M+NH_4]^+$; found: 393.0217. HPLC (YMC Chiral ART Amylose-SA, eluent: hexane:*i*-PrOH = 20/1, 1.0 mL/min, 10 °C), retention times: t_{major} = 13.8 min, t_{minor} = 15.4 min.

Compound (+)-**2h**^{Cl}: Following procedure A, the α-chlorination of **1h** (30.0 mg, 0.101 mmol) gave

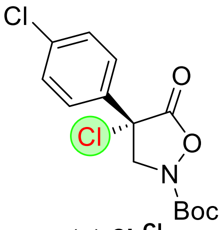

(+)-**2h**^{Cl}
51%, *e.r.* = 93:7

(+)-**2h**^{Cl} in 51% isolated yield (17.0 mg, 0.051 mmol) with *e.r.* = 93:7. R_f (heptanes/EtOAc = 5/1) = 0.40. $[a]_D^{23}$ (c = 0.45, $CHCl_3$) = +65.8°. ^{1}H NMR (300 MHz, δ, $CDCl_3$, 298 K): 7.60–7.56 (m, 2H), 7.46–7.41 (m, 2H), 4.87 (d, J = 13.2 Hz, 1H), 4.43 (d, J = 13.2 Hz, 1H), 1.52 (s, 9H). ^{13}C NMR (75 MHz, δ, $CDCl_3$, 298 K): 169.9, 156.0, 136.7, 132.3, 129.7 (2C), 128.7 (2C), 85.5, 63.7, 63.2, 28.2 (3C). HRMS (ESI): calcd *m/z* for $C_{14}H_{19}Cl_2N_2O_4^+$: 349.0716 $[M+NH_4]^+$; found: 349.0721. HPLC (YMC Chiral ART Amylose-SA, eluent: hexane:*i*-PrOH = 30/1, 1.0 mL/min, 10 °C), retention times: t_{major} = 15.1 min, t_{minor} = 16.6 min.

Compound (+)-**2i**^{N3}: Following procedures A+B, the α-chlorination and subsequent azidiation of **1i**

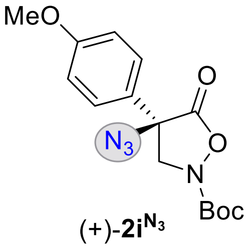

(+)-**2i**^{N_3}
46%, *e.r.* = 73:27

(29.4 mg, 0.100 mmol) gave (+)-**2i**^{N3} in 46% isolated yield (15.3 mg, 0.046 mmol) with *e.r.* = 73:27. R_f (heptanes/EtOAc = 4/1) = 0.37. $[a]_D^{23}$ (c = 0.49, $CHCl_3$) = +36.1°. ^{1}H NMR (300 MHz, δ, $CDCl_3$, 298 K): 7.46–7.41 (m, 2H), 7.01–6.96 (m, 2H), 4.40 (d, J = 12.2 Hz, 1H), 4.18 (d, J = 12.2 Hz, 1H), 3.83 (s, 3H), 1.37 (s, 9H). ^{13}C NMR (75 MHz, δ, $CDCl_3$, 298 K): 172.4, 161.3, 156.1, 128.7 (2C), 124.1, 115.2 (2C), 85.0, 67.4, 60.8, 55.8, 28.1 (3C). HRMS (ESI): calcd *m/z* for $C_{15}H_{22}N_2O_5^+$: 352.1615 $[M+NH_4]^+$; found: 352.1609. HPLC (YMC Chiral ART Amylose-SA, eluent: hexane:*i*-PrOH = 30/1, 0.5 mL/min, 10 °C), retention times: t_{major} = 24.8 min, t_{minor} = 23.3 min.

Compound (+)-**2j**^{N3}: Following procedures A+B, the α-chlorination and subsequent azidiation of **1j**

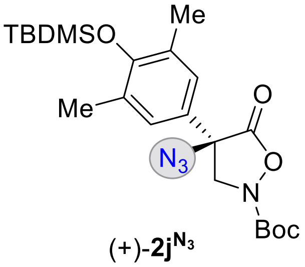

(+)-**2j**^{N_3}
49%, *e.r.* = 76:24

(42.6 mg, 0.101 mmol) gave (+)-**2j**^{N3} in 49% isolated yield (23.0 mg, 0.050 mmol) with *e.r.* = 76:24. R_f (heptanes/EtOAc = 10/1) = 0.37. $[a]_D^{23}$ (c = 0.99, $CHCl_3$) = +27.0°. ^{1}H NMR (300 MHz, δ, $CDCl_3$, 298 K): 7.08 (s, 2H), 4.42 (d, J = 12.1 Hz, 1H), 4.12 (d, J = 12.1 Hz, 1H), 2.23 (s, 6H), 1.37 (s, 9H), 1.03 (s, 9H), 0.19 (d, J = 2.0 Hz, 6H). ^{13}C NMR (75 MHz, δ, $CDCl_3$, 298 K): 172.6, 156.1, 154.1, 130.3 (2C), 127.6 (2C), 124.6, 84.8, 67.5, 60.8, 28.1 (3C), 26.4 (3C), 19.1, 18.4 (2C), -2.5 (2C). HRMS (ESI): calcd *m/z* for $C_{22}H_{38}N_5O_5Si^+$: 480.2637 $[M+NH_4]^+$; found: 480.2639. HPLC (CHIRALCEL® AD-H, eluent: hexane:*i*-PrOH = 100/1, 0.5 mL/min, 10 °C), retention times: t_{major} = 16.6 min, t_{minor} = 14.6 min.

2.2 α-Fluorination

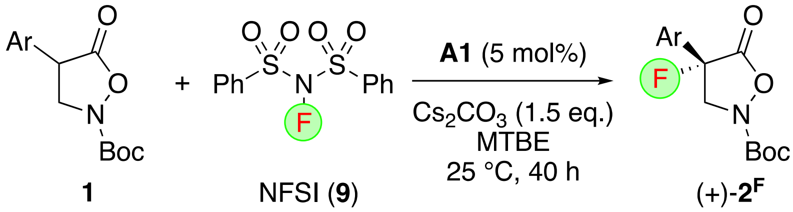

1 NFSI (**9**) (+)-**2**^{F}

General Procedure C (Fluorination): A flame-dried reaction vial was charged with *N*-fluorobenzenesulfonimide **9** (81.3 mg, 2.5 equiv), **A1** (5.6 mg, 5 mol%), Cs_2CO_3 (48.9 mg, 1.5 equiv) and 4-aryl-isoxazolidin-5-one **1** (0.1 mmol, 1 equiv). Then the vial was flushed with argon and anhydrous TBME (6 mL) was added counter currently to the gas flow. After stirring the reaction mixture at rt. for 40 h, the mixture was filtered through a bed of Na_2SO_4, washed with DCM and the solvent was removed under reduced pressure. The crude product was purified *via* column chromatography (silica gel, Et_2O/heptanes = 1/2) to yield α-fluoride **(+)-2**^{F}.

Compound (+)-**2a**^{F}: Following procedure C, the α-fluorination of **1a** (26.3 mg, 0.100 mmol) gave **(+)-2a**^{F} in 85% ^{1}H NMR yield (using 4-fluoroanisol as internal standard) and 55% isolated yield (15.8 mg, 0.056 mmol) after column chromatography with *e.r.* = 93:7. R_f (Et_2O/heptanes = 2/1) = 0.44. $[a]_D^{22}$ (c = 0.44, $CHCl_3$) = +12.87°. ^{1}H NMR (300 MHz, δ, $CDCl_3$, 298 K): 7.48 (s, 5H), 4.62 (dd, $^3J_{HF}$ = 17.8 Hz, $^3J_{HH}$ = 13.1 Hz, 1H), 4.44 (dd, $^3J_{HF}$ = 21.9 Hz, $^3J_{HH}$ = 13.1 Hz, 1H), 1.50 (s, 9H). ^{13}C NMR (75 MHz, δ, $CDCl_3$, 298 K): 169.1 (d, $^2J_{CF}$ = 25.5 Hz), 155.9, 132.4 (d, $^2J_{CF}$ = 23.2 Hz), 130.5 (d, $^4J_{CF}$ = 6.6 Hz), 129.2, 125.6 (d, $^3J_{CF}$ = 6.6 Hz), 93.1 (d, $^1J_{CF}$ = 190.1 Hz), 59.9 (d, $^2J_{CF}$ = 26.7 Hz), 28.0. ^{19}F NMR (282 MHz, δ, $CDCl_3$, 298 K): -155.2 (dd, $^3J_{FH}$ = 21.9 Hz, $^3J_{FH}$ = 17.8 Hz). HRMS (ESI): calcd *m/z* for $C_{14}H_{20}FN_2O_4$: 299.1401 $[M+NH_4]^+$; found: 299.1413. HPLC (YMC CHIRAL ART Cellulose-SA, eluent: hexane:*i*-PrOH = 100:1, 0.5 mL/min, 10 °C) retention times: t_{major} = 27.2 min, t_{minor} = 35.2 min.

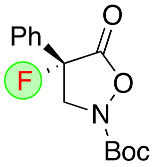

(+)-**2a**^{F}
85% (IST), 55% (SG)
e.r. = 93:7

Compound (+)-**2b**^{F}: Following procedure C, the α-fluorination of **1b** (31.3 mg, 0.100 mmol) gave **(+)-2b**^{F} in 95% ^{1}H NMR yield (using 4-fluoroanisol as internal standard) and 60% isolated yield (19.9 mg, 0.060 mmol) after column chromatography with *e.r.* = 91:9. R_f (heptanes/Et_2O = 2/1) = 0.40. $[a]_D^{23}$ (c = 0.38, $CHCl_3$) = +10.8°; ^{1}H NMR (300 MHz, δ, $CDCl_3$, 298 K): 7.98–7.94 (m, 2H), 7.90–7.87 (m, 2H), 7.61–7.54 (m, 3H), 4.69 (dd, $^3J_{HF}$ = 17.7 Hz, $^3J_{HH}$ = 13.1 Hz, 1H), 4.58 (dd, $^3J_{HF}$ = 20.8 Hz, $^3J_{HH}$ = 13.0 Hz, 1H), 1.48 (s, 9H). ^{13}C NMR (75 MHz, δ, $CDCl_3$, 298 K): 169.3 (d, $^2J_{CF}$ = 25.2 Hz), 156.2, 134.2, 132.9, 129.7, 129.3 (d, $^2J_{CF}$ = 22.9 Hz), 128.9, 128.1, 128.0, 127.5, 125.9 (d, $^3J_{CF}$ = 7.5 Hz), 122.4 (d, $^3J_{CF}$ = 5.8 Hz), 93.5 (d, $^1J_{CF}$ = 190.0 Hz), 85.5, 60.0 (d, $^2J_{CF}$ = 27.0 Hz), 28.2. ^{19}F NMR (282 MHz, δ, $CDCl_3$,

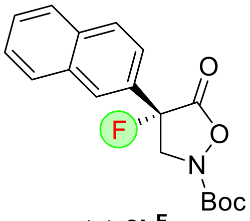

(+)-**2b**^{F}
95% (IST), 60% (SG)
e.r. = 91:9

298 K): -154.4 (dd, $^3J_{FH}$ = 20.8 Hz, $^3J_{FH}$ = 17.7 Hz). HRMS (ESI): calcd *m/z* for $C_{18}H_{22}FN_2O_4$: 349.1558 $[M+NH_4]^+$; found: 349.1557. HPLC (YMC CHIRAL ART Cellulose-SA, eluent: hexane:*i*-PrOH = 10:1, 0.5 mL/min, 10 °C) retention times: t_{major} = 15.1 min, t_{minor} = 17.4 min.

Compound (+)-**2c^F**: Following procedure C, the α-fluorination of **1c** (27.7 mg, 0.100 mmol) gave **(+)-2c^F** in 95% ^{1}H NMR yield (using 4-fluoroanisol as internal standard) and 67% isolated yield (19.6 mg, 0.066 mmol) after column chromatography with *e.r.* = 94:6. R_f (heptanes/EtOAc = 1/1) = 0.85. $[a]_D^{23}$ (c = 0.41, $CHCl_3$) = +22.3°; ^{1}H NMR (300 MHz, δ, $CDCl_3$, 298 K): 7.39 (d, $^3J_{HH}$ = 8.1 Hz, 2H), 7.27 (d, $^3J_{HH}$ = 8.1 Hz, 2H), 4.60 (dd, $^3J_{HF}$ = 17.5 Hz, $^3J_{HH}$ = 13.0 Hz, 1H), 4.45 (dd, $^3J_{HF}$ = 21.5 Hz, $^3J_{HH}$ = 13.0 Hz, 1H), 2.39 (s, 3H), 1.49 (s, 9H). ^{13}C NMR (75 MHz, δ, $CDCl_3$, 298 K): 169.4 (d, $^2J_{CF}$ = 26.0 Hz), 156.2, 141.0 (d, $^4J_{CF}$ = 2.2 Hz), 130.1, 129.1 (d, $^2J_{CF}$ = 23.3 Hz), 125.8 (d, $^3J_{CF}$ = 6.1 Hz), 93.2 (d, $^1J_{CF}$ = 129.2 Hz), 85.4, 59.9 (d, $^2J_{CF}$ = 26.7 Hz), 28.2, 21.6. ^{19}F NMR (282 MHz, δ, $CDCl_3$, 298 K): -153.7 (dd, $^3J_{FH}$ = 21.5 Hz, $^3J_{FH}$ = 17.5 Hz). HRMS (ESI): calcd *m/z* for $C_{15}H_{22}FN_2O_4$: 313.1558 $[M+NH_4]^+$; found: 313.1560. HPLC (YMC CHIRAL ART Cellulose-SA, eluent: hexane:*i*-PrOH = 100:1, 0.5 mL/min, 10 °C) retention times: t_{major} = 24.5 min, t_{minor} = 31.7 min.

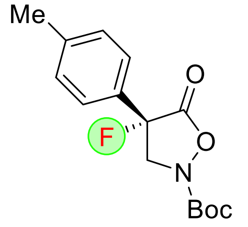

(+)-**2c^F**
95% (IST), 67% (SG)
e.r. = 94:6

Compound (+)-**2d^F**: Following procedure C, the α-fluorination of **1d** (26.9 mg, 0.100 mmol) gave **(+)-2d^F** in 80% ^{1}H NMR yield (using 4-fluoroanisol as internal standard) and 30% isolated yield (8.5 mg, 0.030 mmol) after column chromatography with *e.r.* = 95:5. R_f (Et_2O/heptanes = 2/1) = 0.20. $[a]_D^{23}$ (c = 0.42, $CHCl_3$) = +3.9°; ^{1}H NMR (300 MHz, δ, $CDCl_3$, 298 K): 7.65–7.63 (m, 1H), 7.47–7.44 (m, 1H), 7.27–7.24 (m, 1H), 7.26–7.25 (m, 1H), 4.60 (dd, $^3J_{HF}$ = 22.3 Hz, $^3J_{HH}$ = 13.0 Hz, 1H), 4.54 (dd, $^3J_{HF}$ = 24.5 Hz, $^3J_{HH}$ = 13.0 Hz, 1H), 1.48 (s, 9H). ^{13}C NMR (75 MHz, δ, $CDCl_3$, 298 K): 169.1 (d, $^2J_{CF}$ = 26.8 Hz), 156.2, 132.3 (d, $^2J_{CF}$ = 25.4 Hz), 128.3, 126.5 (d, $^3J_{CF}$ = 6.6 Hz), 125.6 (d, $^4J_{CF}$ = 2.5 Hz), 90.1 (d, $^1J_{CF}$ = 191.1 Hz), 85.5, 59.2 (d, $^2J_{CF}$ = 27.2 Hz), 28.2. ^{19}F NMR (282 MHz, δ, $CDCl_3$, 298 K): -145.8 (dd, $^3J_{FH}$ = 24.5 Hz, $^3J_{FH}$ = 22.3 Hz). HRMS (ESI): calcd *m/z* for $C_{12}H_{18}FN_2O_4S$: 305.0966 $[M+NH_4]^+$; found: 305.0969. HPLC (YMC CHIRAL ART Cellulose-SA, eluent: hexane:*i*-PrOH = 10:1, 0.5 mL/min, 10 °C) retention times: t_{major} = 16.6 min, t_{minor} = 18.7 min.

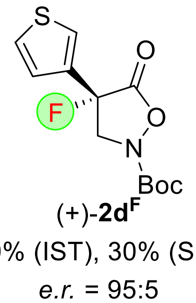

(+)-**2d^F**
80% (IST), 30% (SG)
e.r. = 95:5

Compound (+)-**2e^{F}**: Following procedure C, the α-fluorination of **1e** (33.2 mg, 0.100 mmol) gave

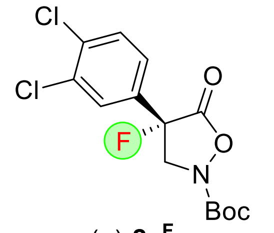

(+)-**2e^{F}**

90% (IST), < 20% (SG)

e.r. = 85:15

(+)-2e^{F} in 90% ^{1}H NMR yield (using 4-fluoroanisol as internal standard) and 13% isolated yield (4.6 mg, 0.013 mmol) after column chromatography with *e.r.* = 85:15. R_f (Et_2O/heptanes = 1/2) = 0.47. $[a]_D^{23}$ (c = 0.06, $CHCl_3$) = +7.14; ^{1}H NMR (300 MHz, δ, $CDCl_3$, 298 K): 7.61 (d, $^{4}J_{HH}$ = 2.1 Hz, 1H), 7.56 (d, $^{3}J_{HH}$ = 8.4 Hz, 1H), 7.33 (dd, $^{3}J_{HH}$ = 8.4 Hz, $^{4}J_{HH}$ = 2.1 Hz, 1H), 4.63 (dd, $^{3}J_{HF}$ = 18.4 Hz, $^{3}J_{HH}$ = 13.2 Hz, 1H), 4.37 (dd, $^{3}J_{HF}$ = 23.1 Hz, $^{3}J_{HH}$ = 13.2 Hz, 1H), 1.53 (s, 9H). ^{13}C NMR (176 MHz, δ, $CDCl_3$, 298 K): 168.6 (d, $^{2}J_{CF}$ = 25.8 Hz), 155.9, 136.9, 130.4 (d, $^{2}J_{CF}$ = 23.4 Hz), 129.5, 128.9, 127.1 (d, $^{3}J_{CF}$ = 6.7 Hz), 126.4 (d, $^{3}J_{CF}$ = 25.8 Hz), 92.7 (d, $^{1}J_{CF}$ = 191.0 Hz), 85.5, 59.6 (d, $^{2}J_{CF}$ = 26.4 Hz), 28.1. ^{19}F NMR (282 MHz, δ, $CDCl_3$, 298 K): -155.9 (dd, $^{3}J_{FH}$ = 23.1 Hz, $^{3}J_{FH}$ = 18.4 Hz). HRMS (ESI): calcd *m/z* for $C_{14}H_{18}FCl_2N_2O_4$: 367.0622 $[M+NH_4]^+$; found: 367.0611. HPLC (YMC CHIRAL ART Cellulose-SA, eluent: hexane:*i*-PrOH = 100:1, 0.5 mL/min, 10 °C) retention times: t_{major} = 35.5 min, t_{minor} = 53.2 min.

Compound (+)-**2f^{F}**: Following procedure C, the α-fluorination of **1f** (28.1 mg, 0.100 mmol) gave **(+)-2f^{F}**

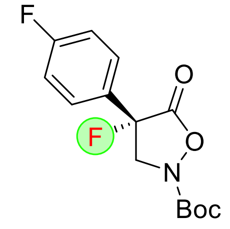

(+)-**2f^{F}**

95% (IST), 44% (SG)

e.r. = 93:7

in 95% ^{1}H NMR yield (using 4-fluoroanisol as internal standard) and 44% isolated yield (13.1 mg, 0.044 mmol) after column chromatography with *e.r.* = 93:7. R_f (Et_2O/heptanes = 2/1) = 0.41. $[a]_D^{23}$ (c = 0.38, $CHCl_3$) = +22.3°; ^{1}H NMR (300 MHz, δ, $CDCl_3$, 298 K): 7.54–7.49 (m, 2H), 7.20–7.14 (m, 2H), 4.63 (dd, $^{3}J_{HF}$ = 17.7 Hz, $^{3}J_{HF}$ = 13.1 Hz, 1H), 4.42 (dd, $^{3}J_{HF}$ = 22.1 Hz, $^{3}J_{HH}$ = 13.1 Hz,1H), 1.51 (s, 9H). ^{13}C NMR (75 MHz, δ, $CDCl_3$, 298 K): 169.0 (d, $^{2}J_{CF}$ = 26.5 Hz), 164.2 (dd, $^{1}J_{CF}$ = 251.4 Hz, $^{5}J_{CF}$ = 2.3 Hz), 156.1, 128.2 (dd, $^{2}J_{CF}$ = 23.9 Hz, $^{4}J_{CF}$ = 3.5 Hz), 128.0 (dd, $^{3}J_{CF}$ = 8.6 Hz, $^{3}J_{CF}$ = 6.5 Hz), 116.6 (d, $^{2}J_{CF}$ = 22.5 Hz), 92.8 (d, $^{1}J_{CF}$ = 189.4 Hz), 85.6, 59.8 (d, $^{2}J_{CF}$ = 26.6 Hz), 28.2. ^{19}F NMR (282 MHz, δ, $CDCl_3$, 298 K): -109.6 (m), -152.9 (m). HRMS (ESI): calcd *m/z* for $C_{14}H_{19}F_2N_2O_4$: 317.1307 $[M+NH_4]^+$; found: 317.1319. YMC CHIRAL ART Cellulose-SA, eluent: hexane:*i*-PrOH = 10:1, 0.5 mL/min, 10 °C) retention times: t_{major} = 15.4 min, t_{minor} = 18.6 min.

Compound (+)-**2g^{F}**: Following procedure C, the α-fluorination of **1g** (34.2 mg, 0.100 mmol) gave

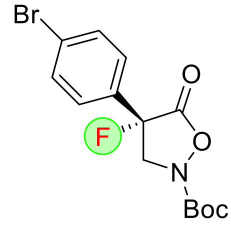

(+)-**2g^{F}**

95% (IST), 37% (SG)

e.r. = 90:10

(+)-2g^{F} in 95% ^{1}H NMR yield (using 4-fluoroanisol as internal standard) and 37% isolated yield (12.9 mg, 0.037 mmol) after column chromatography with *e.r.* = 90:10. R_f (Et_2O/heptanes = 1/2) = 0.50. $[a]_D^{23}$ (c =0.05, $CHCl_3$) = +19.00; ^{1}H NMR (300 MHz, δ, $CDCl_3$, 298 K): 7.61 (d, $^{3}J_{HH}$ = 8.1 Hz, 2H), 7.37 (d, $^{3}J_{HH}$ = 8.1 Hz, 2H), 4.63 (dd, $^{3}J_{HF}$ = 18.1 Hz, $^{3}J_{HH}$ = 13.0 Hz, 1H), 4.38 (dd, $^{3}J_{HF}$ = 22.9 Hz, $^{3}J_{HH}$ = 13.0 Hz, 1H), 1.51 (s, 9H). ^{13}C NMR (75 MHz, δ, $CDCl_3$, 298 K): 168.8 (d, $^{2}J_{CF}$ = 24.9 Hz), 156.1, 132.7, 131.2 (d, $^{2}J_{CF}$ = 23.1 Hz), 127.5 (d, $^{3}J_{CF}$ = 6.8 Hz), 125.3 (d, $^{4}J_{CF}$ = 2.0 Hz), 93.0 (d, $^{1}J_{CF}$ = 189.7 Hz), 85.7, 59.9 (d, $^{2}J_{CF}$ = 27.4 Hz), 28.2. ^{19}F NMR (282 MHz, δ, $CDCl_3$, 298 K): -155.5 (dd, $^{3}J_{FH}$ = 22.9 Hz,

$^3J_{FH}$ = 18.1 Hz). HRMS (ESI): calcd *m/z* for $C_{14}H_{19}BrFN_2O_4$: 377.0506 $[M+NH_4]^+$; found: 377.0497. HPLC (YMC CHIRAL ART Cellulose-SA, eluent: hexane:*i*-PrOH = 100:1, 0.5 mL/min, 10 °C) retention times: t_{major} = 38.4 min, t_{minor} = 62.8 min.

Compound (+)-**2h**$^{\mathbf{F}}$: Following procedure C, the α-fluorination of **1h** (29.8 mg, 0.100 mmol) gave **(+)-2h**$^{\mathbf{F}}$ in 70% ^{1}H NMR yield (using 4-fluoroanisol as internal standard) and 17% isolated yield (5.4 mg, 0.017 mmol) after column chromatography with *e.r.* = 91:9. ^{1}H NMR (300 MHz, δ, $CDCl_3$, 298 K): 7.45 (s, 4H), 4.63 (dd, $^3J_{HF}$ = 18.0 Hz, $^3J_{HH}$ = 13.1 Hz, 1H), 4.39 (dd, $^3J_{HF}$ = 22.8 Hz, $^3J_{HH}$ = 13.1 Hz,1H). ^{13}C NMR (75 MHz, δ, $CDCl_3$, 298 K): 168.8 (d, $^2J_{CF}$ = 25.0 Hz), 156.1, 137.1 (d, $^4J_{CF}$ = 2.0 Hz), 130.6 (d, $^2J_{CF}$ = 23.0 Hz), 129.7, 127.3 (d, $^3J_{CF}$ = 6.6 Hz), 92.9 (d, $^2J_{CF}$ = 194.1 Hz), 85.7, 59.8 (d, $^2J_{CF}$ = 28.7 Hz), 28.2. ^{19}F NMR (282 MHz, δ, $CDCl_3$, 298 K): -155.0 (dd, $^3J_{FH}$ = 22.8 Hz, $^3J_{FH}$ = 18.0 Hz). HRMS (ESI): calcd *m/z* for $C_{14}H_{19}ClFN_2O_4$: 333.1012 $[M+NH_4]^+$; found: 333.1015. HPLC (YMC CHIRAL ART Cellulose-SA, eluent: hexane:*i*-PrOH = 100:1, 0.5 mL/min, 10 °C) retention times: t_{major} = 35.7 min, t_{minor} = 55.3 min.

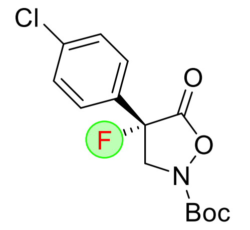

(+)-**2h**$^{\mathbf{F}}$
70% (IST), < 20% (SG)
e.r. = 91:9

Compound (+)-**2i**$^{\mathbf{F}}$: Following procedure C, the α-fluorination of **1i** (29.3 mg, 0.100 mmol) gave **(+)-2i**$^{\mathbf{F}}$ in 95% ^{1}H NMR yield (using 4-fluoroanisol as internal standard) and 12% isolated yield (3.6 mg, 0.012 mmol) after column chromatography with *e.r.* = 95:5. R_f (Et_2O/heptanes = 1/2) = 0.32. $[a]_D^{23}$ (c = 0.05, $CHCl_3$) = +12.9°; ^{1}H NMR (300 MHz, δ, $CDCl_3$, 298 K): 7.46 (d, $^3J_{HH}$ = 8.7 Hz, 2H), 7.00 (d, $^3J_{HH}$ = 8.7 Hz, 2H), 4.57 (dd, $^3J_{HH}$ = 30.3 Hz, $^3J_{HF}$ = 13.0 Hz, 1H), 4.51 (dd, $^3J_{HH}$ = 33.5 Hz, $^3J_{HF}$ = 13.0 Hz,1H), 3.83 (s, 3H), 1.48 (s, 9H). ^{13}C NMR (126 MHz, δ, $CDCl_3$, 298 K): 169.5 (d, $^2J_{CF}$ = 26.3 Hz), 161.6 (d, $^5J_{CF}$ = 2.2 Hz), 156.2, 127.8 (d, $^3J_{CF}$ = 6.0 Hz), 127.3 (d, $^2J_{CF}$ = 42.0 Hz), 114.8, 92.8 (d, $^1J_{CF}$ = 189.3 Hz), 85.4, 59.6 (d, $^2J_{CF}$ = 27.2 Hz), 55.8, 28.2. ^{19}F NMR (282 MHz, δ, $CDCl_3$, 298 K): -149.9 (dd, $^3J_{FH}$ = 13.0 Hz, $^3J_{FH}$ = 13.0 Hz). HRMS (ESI): calcd *m/z* for $C_{15}H_{22}FN_2O_5$: 329.1507 $[M+NH_4]^+$; found: 329.1512. HPLC (YMC CHIRAL ART Cellulose-SA, eluent: hexane:*i*-PrOH = 100:1, 0.5 mL/min, 10 °C) retention times: t_{major} = 51.3 min, t_{minor} = 62.3 min).

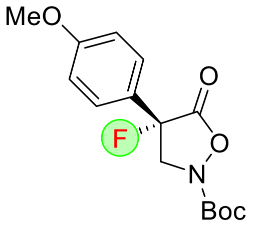

(+)-**2i**$^{\mathbf{F}}$
95% (IST), < 20% (SG)
e.r. = 95:5

2.3 α-Sulfanylation - Bromination

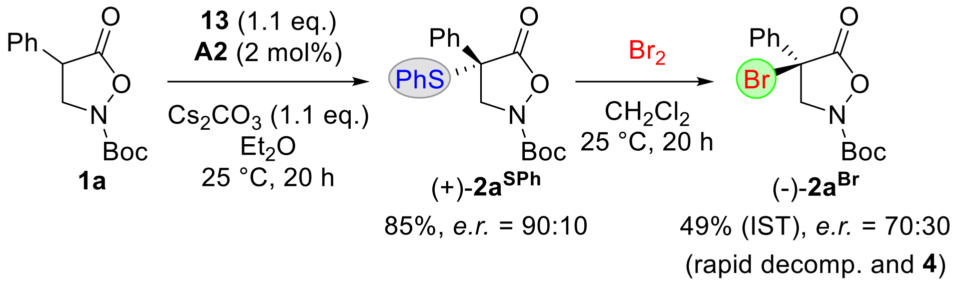

Synthesis of (+)-**2a**$^{\mathbf{SPh}}$ and (-)-**2a**$^{\mathbf{Br}}$ was accomplished in analogy to the procedures reported by J.-F. Brière[1], M. F. A. Adamo and H. Ibrahim[4].

Sulfanylation: A flame-dried Schlenk tube was charged with catalyst **A2** (2 mol%), 4-phenyl-isoxazolidin-5-one **1a** (1 equiv) and dry Et_2O (0.1 M). *N*-(phenylthio)succinimide **13** (1.1 equiv) and Cs_2CO_3 (1.1 equiv) were added successively. The reaction mixture was layered with argon and stirred for 20 h at room temperature, whereupon it was quenched with sat. NH_4Cl solution and diluted with EtOAc and H_2O. The aqueous phase was extracted with EtOAc (3x) and the combined organic phases were washed with brine, dried over anhydrous Na_2SO_4, filtered and concentrated under reduced pressure. The crude product was subjected to flash column chromatography (silica gel, heptanes/DCM = 3/1–1/3) to yield (+)-**2a**$^{\mathbf{SPh}}$.

Compound (+)-**2a**$^{\mathbf{SPh}}$: Following the procedure described above, the α-sulfanylation of **1a** (158 mg,

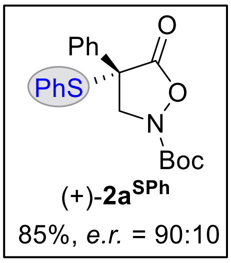

0.600 mmol) gave (+)-**2a**$^{\mathbf{SPh}}$ in 85% isolated yield (190 mg, 0.512 mmol) with *e.r.* = 90:10. R_f (heptanes/DCM = 1/3) = 0.48. $[\alpha]_D^{23}$ (c = 1.00, $CHCl_3$) = +75.3°. ^{1}H NMR (500 MHz, δ, $CDCl_3$, 298 K): 7.46–7.42 (m, 2H), 7.38–7.31 (m, 4H), 7.25–7.19 (m, 4H), 4.69 (d, *J* = 12.5 Hz, 1H), 4.38 (d, *J* = 12.5 Hz, 1H), 1.53 (s, 9H). ^{13}C NMR (126 MHz, δ, $CDCl_3$, 298 K): 171.4, 156.8, 137.3 (2C), 135.1, 130.7 (2C), 129.2 (2C), 129.0 (2C), 128.8, 127.8 (2C), 84.7, 59.9, 56.8, 28.4 (3C). HRMS (ESI): calcd *m/z* for $C_{20}H_{25}N_2O_4S^+$: 389.1530 $[M+NH_4]^+$; found: 389.1536. HPLC (YMC Chiral ART Amylose-SA, eluent: hexane:*i*-PrOH = 49/1, 1.0 mL/min, 10 °C) retention times: t_{major} = 12.0 min, t_{minor} = 16.4 min.

Desulfurative Bromination: A flame-dried Schlenk tube was charged with (+)-**2a**$^{\mathbf{SPh}}$ (1 equiv) and dry DCM (0.1 M). Br_2 (2 equiv) was added and the mixture was stirred for 20 h at room temperature under argon atmosphere. The reaction was quenched with sat. $Na_2S_2O_3$ solution and the aqueous phase was extracted with DCM (3x). The collected organic layers were washed with brine, dried over anhydrous

[1] Cadart, T.; Berthonneau, C.; Levacher, V.; Perrio, S.; Brière, J.-F. Enantioselective Phase-Transfer Catalyzed α-Sulfanylation of Isoxazolidin-5-ones: An Entry to $\beta^{2,2}$-Amino Acid Derivatives, *Chem. Eur. J.* **2016**, *22*, 15261–15264.
[4] Canestrari, D.; Cioffi, C.; Biancofiore, I.; Lancianesi, S.; Ghisu, L.; Ruether, M.; O'Brien, J.; Adamo, M. F. A.; Ibrahim, H. Sulphide as a leaving group: highly stereoselective bromination of alkyl phenyl sulphides. *Chem. Sci.* **2019**, *10*, 9042-9050.

Na_2SO_4, filtered and concentrated under reduced pressure. The crude product contained mainly alpha-bromide (-)-**2a**^{Br} and was not further purified due to its relative instability.

Compound (-)-**2a**^{Br}: Following the procedure described above, the desulfurative bromination of

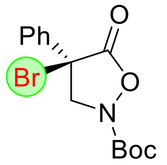

(-)-**2a**^{Br}
49% (IST), *e.r.* = 70:30

(+)-**2a**^{SPh} (40.4 mg, 0.109 mmol) gave (-)-**2a**^{Br} in 49% yield (18.2 mg, 0.053 mmol, ^{1}H NMR yield using *o*-xylene as an internal standard) with *e.r.* = 70:30. $[\alpha]_D^{23}$ (c = 1.00, $CHCl_3$) = -31.0°. ^{1}H NMR (300 MHz, δ, $CDCl_3$, 298 K): 7.76–7.70 (m, 2H), 7.47–7.40 (m, 3H), 5.01 (d, J = 13.6 Hz, 1H), 4.43 (d, J = 13.6 Hz, 1H), 1.55 (s, 9H). ^{13}C NMR (75 MHz, δ, $CDCl_3$, 298 K): 170.2, 156.0, 134.5, 130.3, 129.5 (2C), 127.6 (2C), 85.3, 63.3, 53.4, 28.3 (3C). HRMS (ESI): calcd *m/z* for $C_{14}H_{20}BrN_2O_4^+$: 359.0601 $[M+NH_4]^+$; found: 359.0608. HPLC (CHIRALCEL® AD-H, eluent: hexane:*i*-PrOH = 19/1, 1.0 mL/min, 10 °C) retention times: t_{major} = 10.9 min, t_{minor} = 12.9 min.

2.4 Further Transformations and Products

Compound (+)-**2a**^{N3}: Following **2.1. Chlorination procedure B**, the nucleophilic azidation of

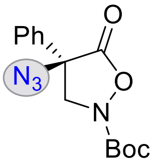

(+)-**2a**^{N_3}
(98%, *e.r.* = 99:1)

enantioenriched (+)-**2a**^{Cl} (29.8 mg, 0.100 mmol, *e.r.* = 99:1) gave (+)-**2a**^{N3} in 98% isolated yield (29.9 mg, 0.098 mmol) with *e.s.* > 99.5%. R_f (heptanes/EtOAc = 5/1) = 0.37. $[a]_D^{23}$ (c = 1.00, $CHCl_3$) = +110.8°. ^{1}H NMR (300 MHz, δ, $CDCl_3$, 298 K): 7.49 (s, 5H), 4.39 (d, J = 12.0 Hz, 1H), 4.21 (d, J = 12.0 Hz, 1H), 1.37 (s, 9H).^{13}C NMR (75 MHz, δ, $CDCl_3$, 298 K): 172.2, 156.0, 132.7, 130.6, 129.9 (2C), 127.1 (2C), 85.1, 67.8, 60.9, 28.1. HRMS (ESI): calcd *m/z* for $C_{14}H_{20}N_5O_4^+$: 322.1510 $[M+NH_4]^+$; found: 322.1517. HPLC (CHIRALCEL® OD-H, eluent: hexane:*i*-PrOH = 4/1, 0.5 mL/min, 10 °C) retention times: t_{major} = 14.4 min, t_{minor} = 12.1 min.

Compound **2a**^{NO2}: To a flame-dried Schlenk tube were added racemic **2a**^{Cl} (30.0 mg, 0.101 mmol) and

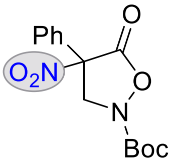

2a^{NO_2} (79%)

anhydrous DMSO (1 mL). $NaNO_2$ (7.5 mg, 1.1 equiv) was added and the resulting reaction mixture was stirred at room temperature under argon atmosphere. After reaction completion (1 h, determined by TLC analysis), the mixture was diluted with EtOAc and H_2O. The aqueous phase was extracted with EtOAc (3x) and the combined organic layers were washed with brine, dried over anhydrous Na_2SO_4, filtered and concentrated under reduced pressure to obtain **2a**^{NO2} in 79% yield (24.6 mg, 0.080 mmol). R_f (ACN/H_2O = 7/3, RP18) = 0.44. ^{1}H NMR (300 MHz, δ, $CDCl_3$, 298 K): 7.68–7.65 (m, 2H), 7.55–7.47 (m, 3H), 5.33 (d, J = 13.2 Hz, 1H), 4.78 (d, J = 13.2 Hz, 1H), 1.43 (s, 9H). ^{13}C NMR (75 MHz, δ, $CDCl_3$, 298 K): 165.4, 155.3, 131.9, 129.8 (2C), 128.5, 128.1 (2C), 91.5, 86.0, 58.4, 28.0 (3C). HRMS (ESI): calcd *m/z* for $C_{14}H_{20}N_3O_6^+$: 326.1347 $[M+NH_4]^+$; found: 326.1355.

Compound (+)-**2a**$^{\mathbf{OEt}}$: A reaction vial was charged with 18-crown-6 (26.4 mg, 0.099 mmol), enantioenriched (+)-**2a**$^{\mathbf{Cl}}$ (29.9 mg, 1 equiv, *e.r.* = 95:5) and $CHCl_3$ (2 mL, 0.6 Vol% EtOH). CsF (44.8 mg, 3 equiv) was added at once under stirring at room temperature. After reaction completion (1 h, determined by TLC analysis), the mixture was directly purified by flash column chromatography (silica gel, heptanes/EtOAc = 2/1) to obtain (-)-**2a**$^{\mathbf{OEt}}$ in 86% isolated yield (26.5 mg, 0.086 mmol) with *e.s.* = 99%. R_f (heptanes/EtOAc = 2/1) = 0.52. $[a]_D^{23}$ (c = 0.22, $CHCl_3$) = -36.7°. ^{1}H NMR (300 MHz, δ, $CDCl_3$, 298 K): 7.52–7.48 (m, 2H), 7.45–7.37 (m, 3H), 5.08 (d, *J* = 8.4 Hz, 1H), 4.55 (d, *J* = 8.4 Hz, 1H), 4.29 (dq, *J* = 7.2, 0.7 Hz, 2H), 1.50 (s, 9H), 1.28 (t, *J* = 7.2 Hz, 3H). ^{13}C NMR (75 MHz, δ, $CDCl_3$, 298 K): 170.7, 161.3, 137.7, 129.4, 129.1 (2C), 124.9 (2C), 83.8 (2C), 63.2, 62.7, 28.4 (3C), 14.3. HRMS (ESI): calcd *m/z* for $C_{16}H_{25}N_2O_5^+$: 325.1758 $[M+NH_4]^+$; found: 325.1763. HPLC (CHIRALCEL® AD-H, eluent: hexane:*i*-PrOH = 49/1, 0.5 mL/min, 10 °C) retention times: t_{major} = 23.1 min, t_{minor} = 22.3 min.

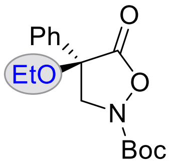

(-)-**2a**$^{\mathbf{OEt}}$
(86%, *e.r.* = 94:6)

Compound **4**: Following **2.1. Chlorination procedure A**, elimination product **4** was formed as a byproduct in varying ratios when employing e.g. K_2CO_3 instead of PhONa as base and isolated by preperative TLC. R_f (heptanes/EtOAc = 5/1) = 0.34. ^{1}H NMR (700 MHz, δ, $CDCl_3$, 298 K): 8.51 (s, 1H), 7.78–7.76 (m, 2H), 7.42–7.40 (m, 2H), 7.35–7.32 (m, 1H), 1.62 (s, 9H). ^{13}C NMR (176 MHz, δ, $CDCl_3$, 298 K): 166.5, 144.9, 138.9, 129.2 (2C), 128.7, 128.0, 126.0 (2C), 107.5, 87.5, 28.4 (3C). HRMS (ESI): calcd *m/z* for $C_{14}H_{19}N_2O_4^+$: 279.1339 $[M+NH_4]^+$; found: 279.1343.

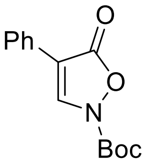

4

Compound **5a**$^{\mathbf{Cl}}$: Following **2.1. Chlorination procedure A**, ring-opening product **5a**$^{\mathbf{Cl}}$ was obtained after extraction of (+)-**2a**$^{\mathbf{Cl}}$ with EtOAc. The pH value of the aqueous phase was lowered to 2 with 1N HCl before it was extracted with EtOAc (3x). The collected organic layers were washed with brine, dried over anhydrous Na_2SO_4, filtered and concentrated under reduced pressure. Due to stability issues, the product could not be fully characterized and was therefore directly transformed to its corresponding methyl ester **6a**$^{\mathbf{Cl}}$ by treatment with $TMSCHN_2$ (2.2 equiv) in MeOH (0.1 M) at room temperature (analytical data see below). ^{1}H NMR (300 MHz, δ, MeOD-d4, 298 K): 7.65–7.63 (m, 2H), 7.38–7.28 (m, 3H), 4.35 (s, 2H), 1.32 (s, 9H) (2 acidic protons not visible in NMR). HRMS (ESI): calcd *m/z* for $C_{14}H_{17}ClNO_5^-$: 314.0801 $[M-H]^-$; found: 314.0809.

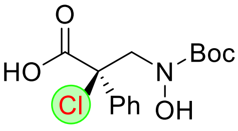

5a$^{\mathbf{Cl}}$

Compound **6a**^{Cl}: To a flame-dried Schlenk tube were added $Y(OTf)_3$ (5.4 mg, 10 mol%), racemic **2a**^{Cl} (30.0 mg, 0.101 mmol) and MeOH (1 mL). The resulting mixture was stirred for 72 h at room temperature under argon atmosphere, whereupon it was concentrated under reduced pressure. The crude product was purified by a short silica plug with heptanes/EtOAc = 1/1 to obtain **6a**^{Cl} in 68% isolated yield (22.6 mg, 0.069 mmol). R_f (heptanes/EtOAc = 1/1) = 0.54. ^{1}H NMR (700 MHz, δ, $CDCl_3$, 298 K): 7.51–7.50 (m, 2H), 7.40–7.34 (m, 3H), 6.30 (s, 1H), 4.48 (d, *J* = 15.0 Hz, 1H), 4.44 (d, *J* = 15.0 Hz, 1H), 3.81 (s, 3H), 1.40 (s, 9H). ^{13}C NMR (176 MHz, δ, $CDCl_3$, 298 K): 170.8, 156.2, 137.7, 129.2, 128.9 (2C), 126.8 (2C), 82.9, 72.9, 59.3, 54.1, 28.4 (3C). HRMS (ESI): calcd *m/z* for $C_{15}H_{20}ClNNaO_5^+$: 352.0922 [M+Na]$^+$; found: 352.0925.

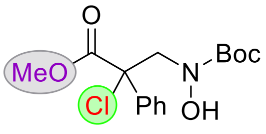

6a^{Cl} (68%)

Compound **6a**^{H}: A flame-dried Schlenk tube equipped with an argon balloon was loaded with 10% Pd/C (3.0 mg, 10 wt%). The flask was evacuated and backfilled with argon (3x) and a solution of racemic **2a**^{Cl} (29.7 mg, 0.100 mmol) in dry MeOH (1 mL) was added under a counterflow of argon. The argon balloon was switched to one filled with hydrogen and the flask was carefully evacuated and backfilled with hydrogen (3x). The reaction mixture was stirred vigorously for 24 h at room temperature under hydrogen atmosphere, whereupon it was filtered through a bed of Celite, washed with DCM and concentrated under reduced pressure. The crude product was purified by a short silica plug with heptanes/EtOAc = 2/1 to obtain **6a**^{H} in 91% isolated yield (26.7 mg, 0.090 mmol). R_f (heptanes/EtOAc = 2/1) = 0.43. ^{1}H NMR (700 MHz, δ, $CDCl_3$, 298 K): 7.35–7.28 (m, 5H), 6.35 (s, 1H), 4.10 (dd, *J* = 7.5, 7.4 Hz, 1H), 4.05 (dd, *J* = 14.3, 7.5 Hz, 1H), 3.96 (dd, *J* = 14.3, 7.4 Hz, 1H), 3.69 (s, 3H), 1.36 (s, 9H). ^{13}C NMR (176 MHz, δ, $CDCl_3$, 298 K): 173.4, 156.2, 136.5, 129.1 (2C), 128.6 (2C), 128.1, 82.4, 52.9, 52.6, 49.6, 28.5 (3C). HRMS (ESI): calcd *m/z* for $C_{15}H_{21}NNaO_5^+$: 318.1317 [M+Na]$^+$; found: 318.1308.

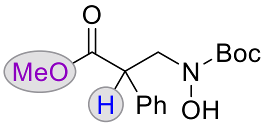

6a^{H} (91%)

Compound **6a**^{N3}: To a flame-dried Schlenk tube were added $Y(OTf)_3$ (5.4 mg, 10 mol%), racemic **2a**^{N3} (30.3 mg, 0.100 mmol) and MeOH (1 mL). The resulting mixture was stirred for 72 h at room temperature under argon atmosphere, whereupon it was concentrated under reduced pressure. The crude product was purified by a short silica plug with heptanes/EtOAc = 1/1 to obtain **6a**^{N3} in 82% isolated yield (27.6 mg, 0.082 mmol). Rf (heptanes/EtOAc = 1/1) = 0.54. ^{1}H NMR (700 MHz, δ, $CDCl_3$, 298 K): 7.44–7.37 (m, 5H), 6.28 (s, 1H). 4.28 (d, *J* = 14.8 Hz, 1H), 4.21 (d, *J* = 14.8 Hz, 1H), 3.86 (s, 3H), 1.45 (s, 9H). ^{13}C NMR (176 MHz, δ, $CDCl_3$, 298 K): 171.1, 156.5, 136.1, 129.4 (3C), 126.5 (2C), 83.0, 72.0, 57.0, 53.7, 28.5 (3C). HRMS (ESI): calcd m/z for $C_{15}H_{20}N_4NaO_5^+$: 359.1326 [M+Na]$^+$; found: 359.1331.

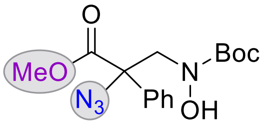

6a^{N3} (82%)

Compound **7a**$^{\mathbf{Cl}}$: A reaction vial was charged with racemic **2a**$^{\mathbf{Cl}}$ (30.3 mg, 0.102 mmol), 4-chlorobenzylamine (62 µL, 5 equiv) and MeOH (1 mL). After stirring for 24 h at room temperature, the solvent was removed under reduced pressure. The crude product was purified *via* flash column chromatography (silica gel, heptanes/EtOAc = 5/1–2/1) to obtain **7a**$^{\mathbf{Cl}}$ in 83% isolated yield (36.9 mg, 0.084 mmol). R_f (heptanes/EtOAc = 2/1) = 0.19. ^{1}H NMR (300 MHz, δ, $CDCl_3$, 298 K): 7.56–7.51 (m, 2H), 7.40–7.31 (m, 4H), 7.29 (d, *J* = 8.4 Hz, 2H), 7.16 (d, *J* = 8.4 Hz, 2H), 7.01–6.91 (m, 1H), 4.51 (d, *J* = 15.2 Hz, 1H), 4.47 (d, *J* = 4.7 Hz, 1H), 4.45 (d, *J* = 4.7 Hz, 1H), 4.36 (d, *J* = 15.2 Hz, 1H), 1.38 (s, 9H). ^{13}C NMR (75 MHz, δ, $CDCl_3$, 298 K): 170.8, 155.7, 138.1, 136.0, 133.9, 129.3 (5C), 128.9 (2C), 127.1 (2C), 82.3, 75.5, 59.9, 44.0, 28.4 (3C). HRMS (ESI): calcd *m/z* for $C_{21}H_{23}Cl_2N_2O_4^-$: 437.1040 [M-H]$^-$; found: 437.1042.

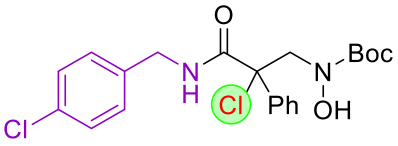

7a$^{\mathbf{Cl}}$ (83%)

Compound **7a**$^{\mathbf{F}}$: In a pressure Schlenk-tube racemic **2a**$^{\mathbf{F}}$ (15.1 mg, 0.054 mmol) was dissolved in t-BuOH. After the addition of 4-chlorobenzylamine (31 µL, 5 equiv), the vial was flushed with argon and heated to 90 °C. After completion of reaction, the mixture was cooled to rt. and the solvent was removed *in vacuo*. The crude product was purified by column chromatography (silica gel, heptanes/EtOAc = 1/1) to obtain **7a**$^{\mathbf{F}}$ in 26% isolated yield (6.2 mg, 0.015 mmol). R_f (heptanes/EtOAc = 1/1) = 0.47. ^{1}H NMR (500 MHz, δ, $CDCl_3$, 298 K): 7.60 – 7.58 (m, 2H), 7.40 – 7.38 (m, 3H), 7.26 (d, $^{3}J_{HH}$ = 8.4 Hz, 2H), 7.14 (d, $^{3}J_{HH}$ = 8.4 Hz, 2H), 6.83 (d, $^{3}J_{HH}$ = 5.3 Hz, 1H), 4.48 (dd, $^{2}J_{HH}$ = 14.9 Hz, $^{3}J_{HH}$ = 5.3 Hz, 1H), 4.40 – 4.25 (m, 3H), 1.41 (s, 9H). ^{13}C NMR (126 MHz, δ, $CDCl_3$, 298 K): 169.8 (d, $^{2}J_{CF}$ = 22.3 Hz), 156.2, 136.2 (d, $^{2}J_{CF}$ = 21.6 Hz), 136.0, 133.7, 129.1, 129.1, 129.0, 128.7 (d, $^{4}J_{CF}$ = 1.7 Hz), 124.8 (d, $^{3}J_{CF}$ = 9.8 Hz), 97.8 (d, $^{1}J_{CF}$ = 194.8 Hz), 82.5, 57.0 (d, $^{2}J_{CF}$ = 23.6 Hz), 42.9, 28.2. ^{19}F NMR (282 MHz, δ, $CDCl_3$, 298 K): -165.2 (s). HRMS (ESI): calcd *m/z* for $C_{16}H_{17}ClFN_2O_2$ (fragment-detection): 323.0957 [M+H]$^+$; found: 323.0959.

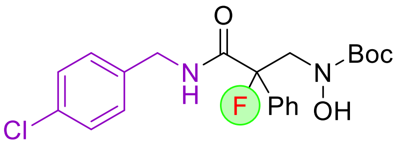

7a$^{\mathbf{F}}$ (26%)

Compound **7a**$^{\mathbf{N3}}$: A reaction vial was charged with racemic **2a**$^{\mathbf{N3}}$ (30.3 mg, 0.100 mmol), 4-chlorobenzylamine (14 µL, 1.1 equiv) and MeOH (1 mL). After stirring for 24 h at room temperature, the solvent was removed under reduced pressure. The crude product was purified *via* flash column chromatography (silica gel, heptanes/EtOAc = 5/1–2/1) to obtain **7a**$^{\mathbf{N3}}$ in 64% isolated yield (28.5 mg, 0.064 mmol). R_f (heptanes/EtOAc = 2/1) = 0.19. ^{1}H NMR (300 MHz, δ, $CDCl_3$, 298 K): 7.48–7.35 (m, 5H), 7.28 (d, *J* = 8.4 Hz, 2H), 7.15 (d, *J* = 8.4 Hz, 2H), 7.06–7.02 (m, 1H), 6.92 (s, 1H), 4.51 (d, *J* = 15.1 Hz, 1H), 4.44–4.36 (m, 3H), 1.46 (s, 9H). ^{13}C NMR (75 MHz, δ, $CDCl_3$, 298 K): 170.3, 156.5, 136.8, 136.4, 133.8, 129.4 (4C), 129.3, 129.2 (2C), 126.5 (2C), 83.1, 72.2, 55.8, 43.6, 28.5 (3C). HRMS (ESI): calcd m/z for $C_{21}H_{23}ClN_5O_4^-$: 444.1444 [M-H]$^-$; found: 444.1444.

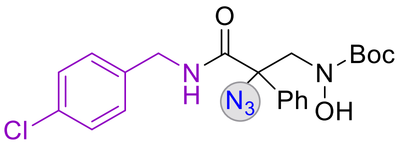

7a$^{\mathbf{N3}}$ (64%)

Compound **7b^{F}**: Analoguessly prepared to **7a^{F}**, using 4-methoxybenzylamine (33 µL, 5 equiv). The crude product was purified by preparative thin-layer chromatography (silica gel, heptanes/EtOAc = 1/1) (5.2 mg, 0.012 mmol, 23%). R_f (heptanes/EtOAc = 1/1) = 0.54. ^{1}H NMR (500 MHz, δ, $CDCl_3$, 298 K): 7.61 – 7.59 (m, 2H), 7.41 – 7.35 (m, 3H), 7.14 (d, $^{3}J_{HH}$ = 8.6 Hz, 2H), 6.83 (d, $^{3}J_{HH}$ = 8.6 Hz, 2H), 6.83 (d, $^{3}J_{HH}$ = 5.9 Hz, 1H), 6.64 (s, 1H), 4.46 (dd, $^{2}J_{HH}$ = 14.7 Hz, $^{3}J_{HH}$ = 5.9 Hz, 1H), 4.36 – 4.33 (m, 2H), 4.29 (s, 1H), 3.78 (s, 3H), 1.41 (s, 9H). ^{13}C NMR (126 MHz, δ, $CDCl_3$, 298 K): 169.5 (d, $^{2}J_{CF}$ = 21.6 Hz), 159.2, 155.9, 136.2 (d, $^{2}J_{CF}$ = 21.5 Hz), 129.3, 129.1, 128.9, 128.5, 124.7 (d, $^{3}J_{CF}$ = 9.7 Hz), 114.2, 97.7 (d, $^{1}J_{CF}$ = 194.1 Hz), 82.2, 56.9 (d, $^{2}J_{CF}$ = 23.6 Hz), 55.3, 43.0, 28.1. ^{19}F NMR (282 MHz, δ, $CDCl_3$, 298 K): -164.7 (s). HRMS (ESI): calcd *m/z* for $C_{17}H_{20}FN_2O_3$ (fragment-detection): 319.1453 $[M+H]^+$; found: 319.1454.

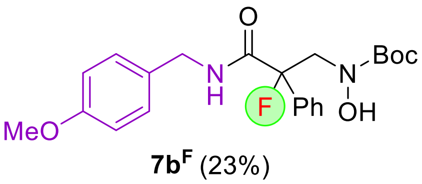

7b^{F} (23%)

Compound **8**: The nucleophilic nitration product **2a^{NO2}** was dissolved in DCM, filtered through a short silica plug and concentrated under reduced pressure to obtain **8** in quantitative yield. R_f (heptanes/EtOAc = 2/1) = 0.59. ^{1}H NMR (700 MHz, δ, $CDCl_3$, 298 K): 7.49–7.46 (m, 2H), 7.45–7.39 (m, 3H), 6.81 (s, 1H), 5.89 (dd, *J* = 9.4, 4.5 Hz, 1H), 4.68 (dd, *J* = 14.9, 9.4 Hz), 3.93 (dd, *J* = 14.9, 4.5 Hz), 1.45 (s, 9H). ^{13}C NMR (176 MHz, δ, $CDCl_3$, 298 K): 156.5, 132.3, 130.6, 129.5 (2C), 128.2 (2C), 87.9, 83.7, 53.3, 28.4 (3C). HRMS (ESI): calcd *m/z* for $C_{13}H_{18}N_2NaO_5^+$: 305.1108 $[M+Na]^+$; found: 305.1113.

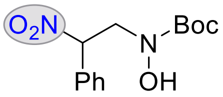

8

4. Copies of Product NMR Spectra

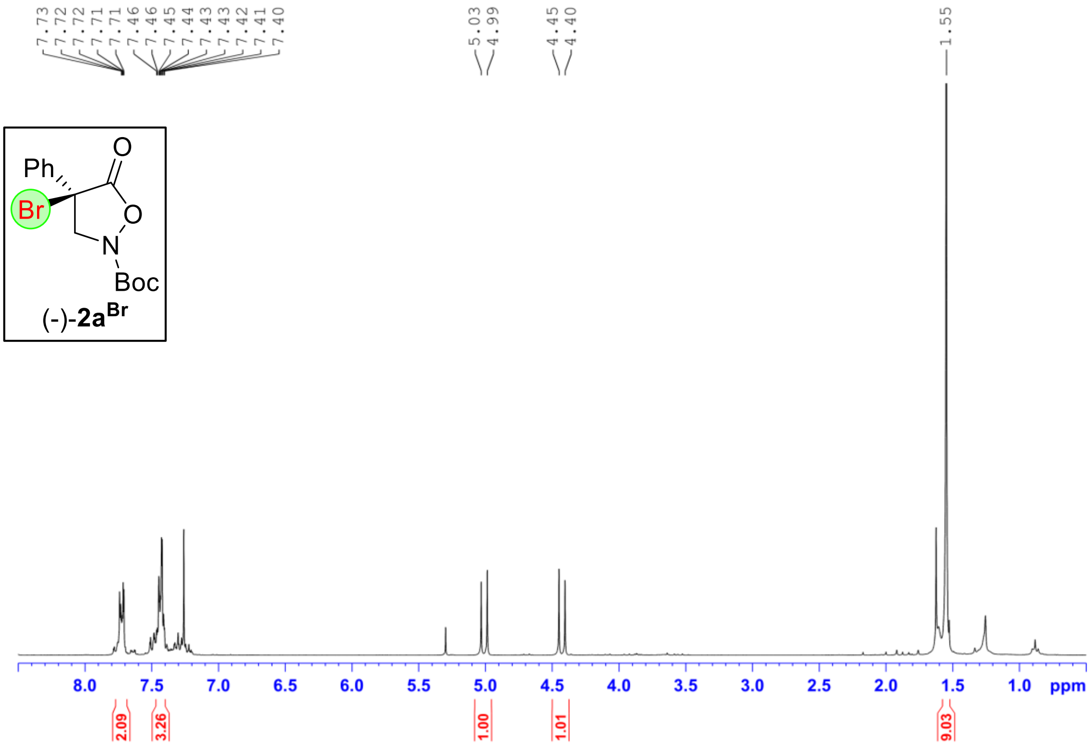

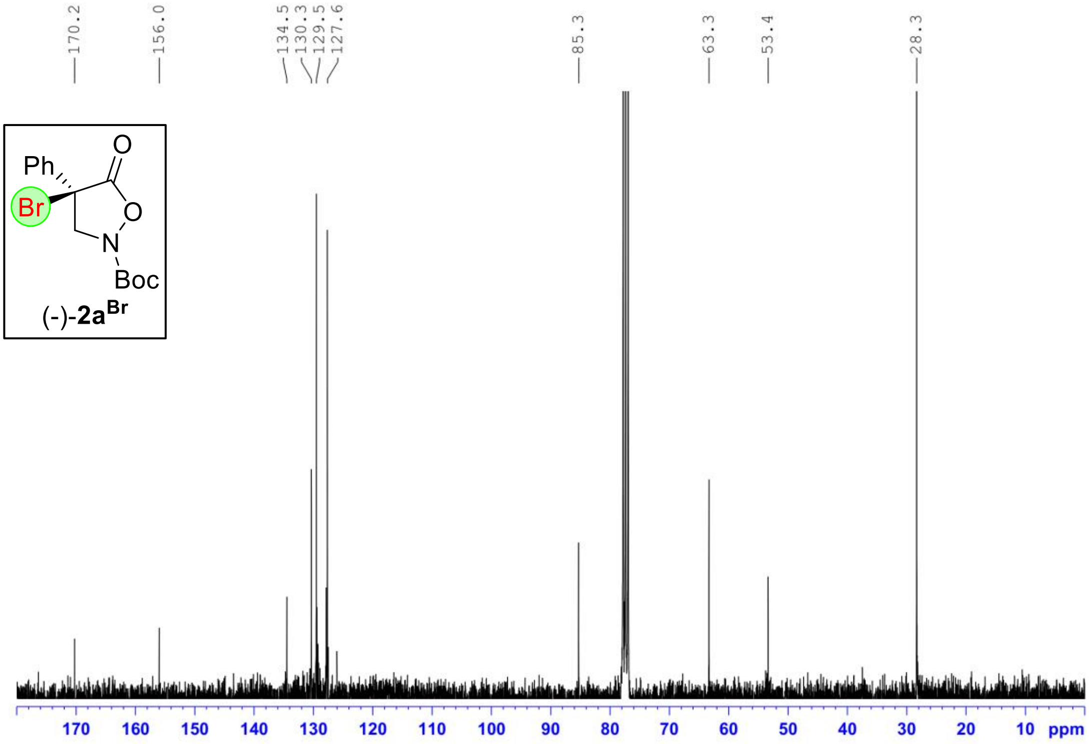

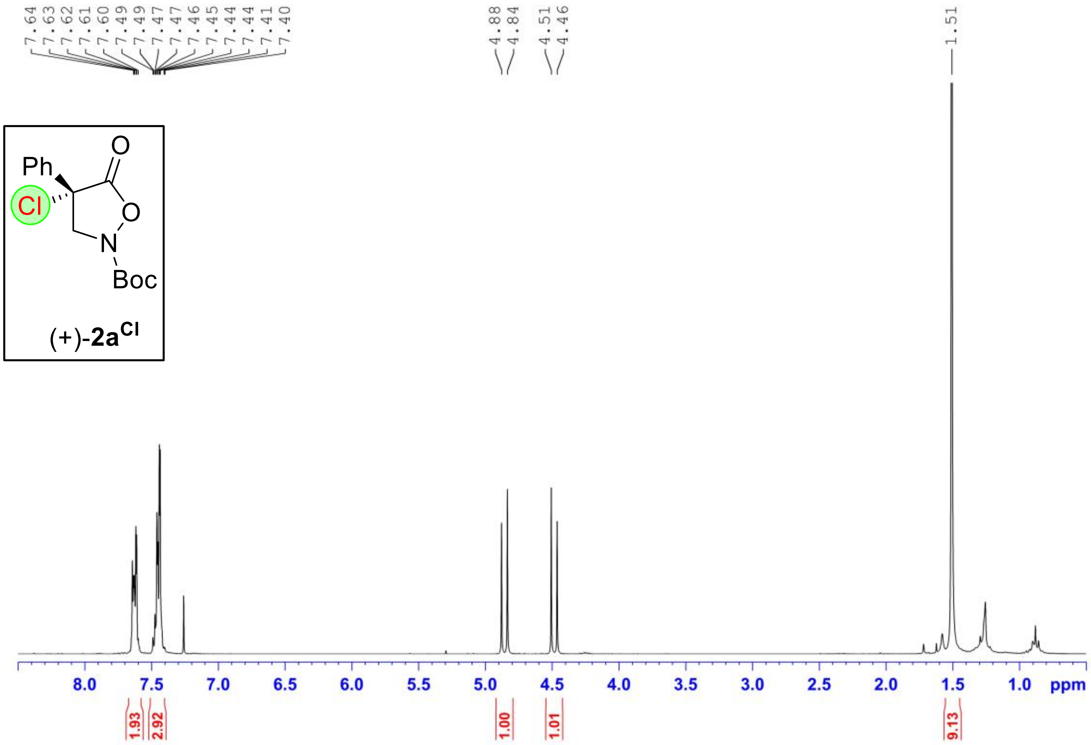
7.64
7.63
7.62
7.61
7.60
7.49
7.49
7.47
7.47
7.46
7.45
7.44
7.44
7.41
7.40
4.88
4.84
4.51
4.46
1.51
Ph
Cl
O
O
N
Boc
(+)-2a^Cl
8.0
7.5
7.0
6.5
6.0
5.5
5.0
4.5
4.0
3.5
3.0
2.5
2.0
1.5
1.0
ppm
1.93
2.92
1.00
1.01
9.13

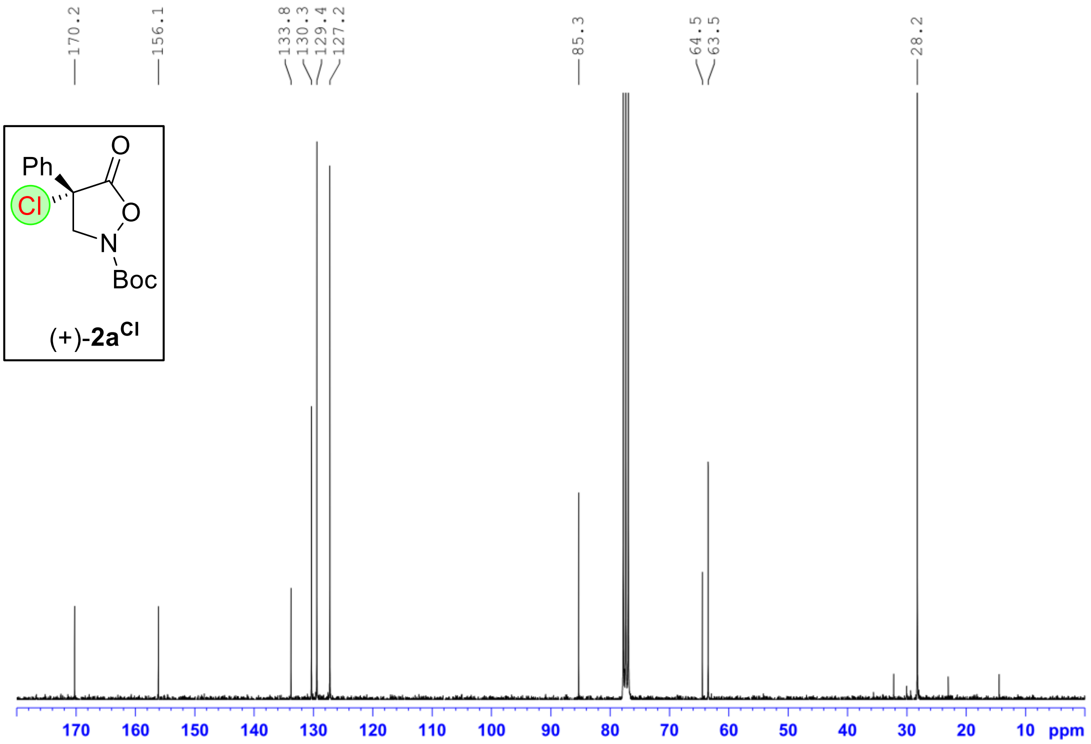
170.2
156.1
133.8
130.3
129.4
127.2
85.3
64.5
63.5
28.2
Ph
Cl
O
O
N
Boc
(+)-2a^Cl
170
160
150
140
130
120
110
100
90
80
70
60
50
40
30
20
10
ppm

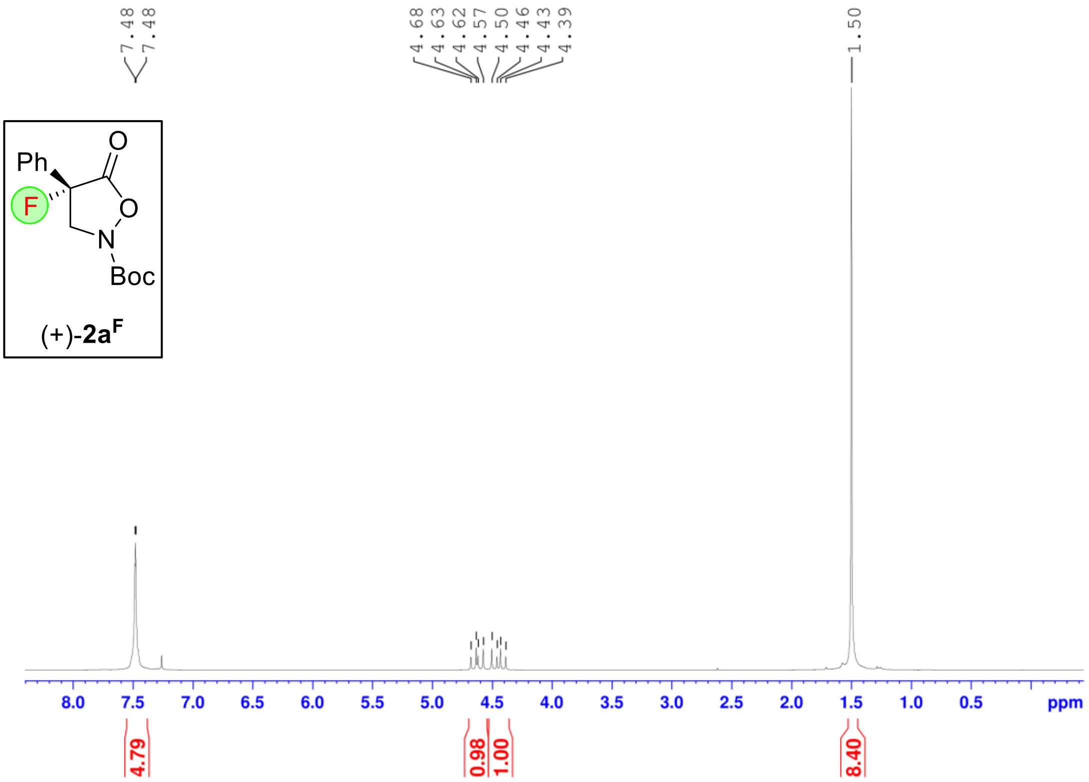
7.48
7.48
4.68
4.63
4.62
4.57
4.50
4.46
4.43
4.39
1.50
Ph
F
O
O
N
Boc
(+)-2a^F
8.0
7.5
7.0
6.5
6.0
5.5
5.0
4.5
4.0
3.5
3.0
2.5
2.0
1.5
1.0
0.5
ppm
4.79
0.98
1.00
8.40

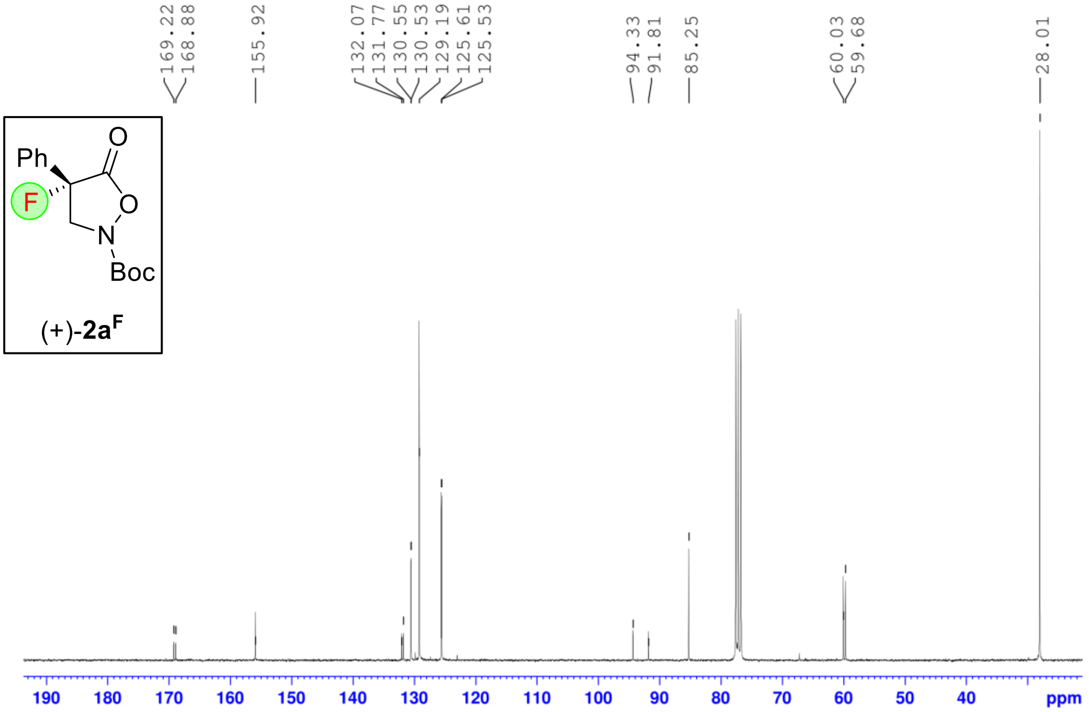
169.22
168.88
155.92
132.07
131.77
130.55
130.53
129.19
125.61
125.53
94.33
91.81
85.25
60.03
59.68
28.01
Ph
F
O
O
N
Boc
(+)-2a^F
190
180
170
160
150
140
130
120
110
100
90
80
70
60
50
40
ppm

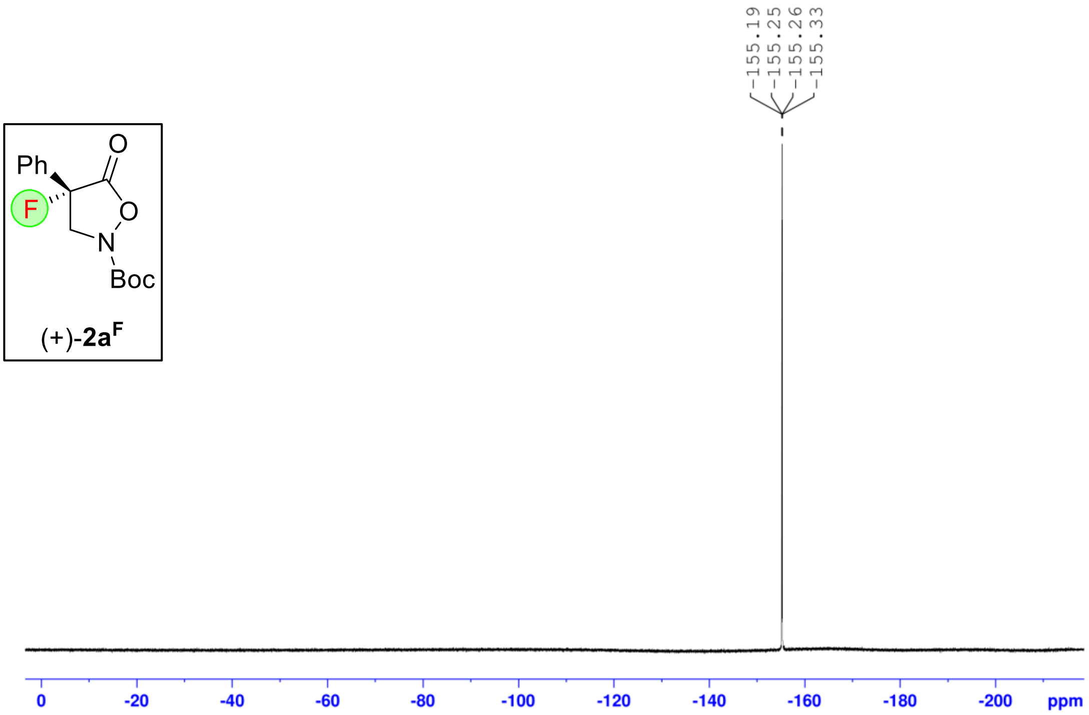

-155.19
-155.25
-155.26
-155.33
Ph
F
O
O
N
Boc
(+)-2a^F
0
-20
-40
-60
-80
-100
-120
-140
-160
-180
-200
ppm

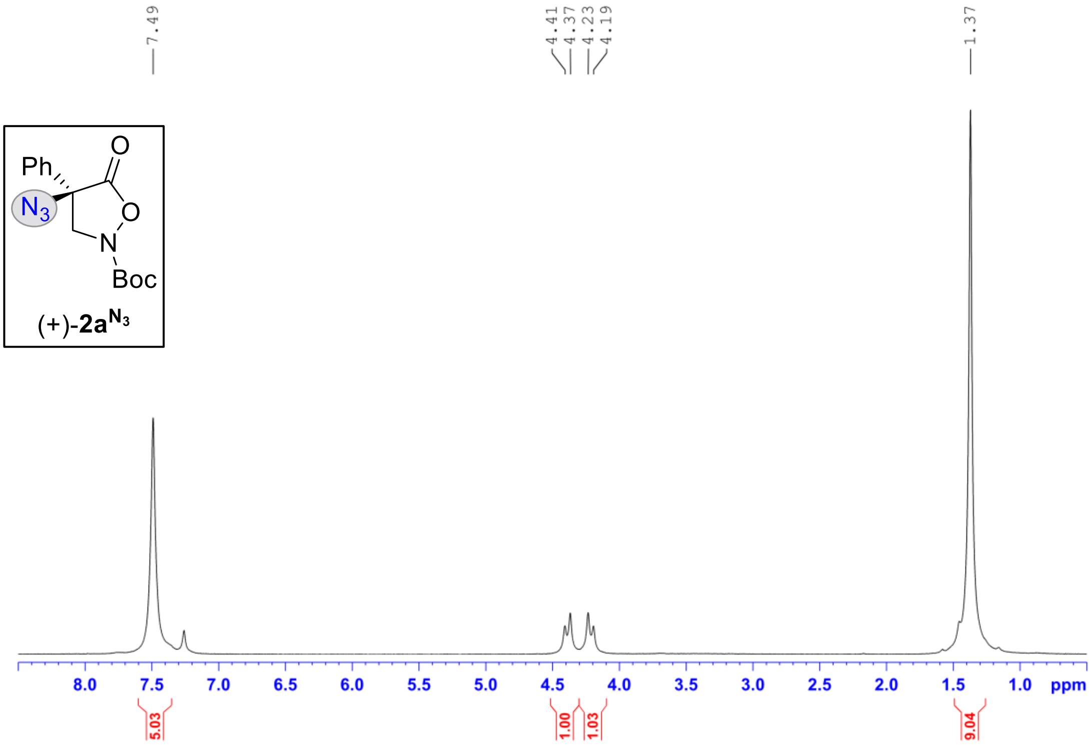

7.49
4.41
4.37
4.23
4.19
1.37
Ph
N3
O
O
N
Boc
(+)-2a^N3
8.0
7.5
7.0
6.5
6.0
5.5
5.0
4.5
4.0
3.5
3.0
2.5
2.0
1.5
1.0
ppm
5.03
1.00
1.03
9.04

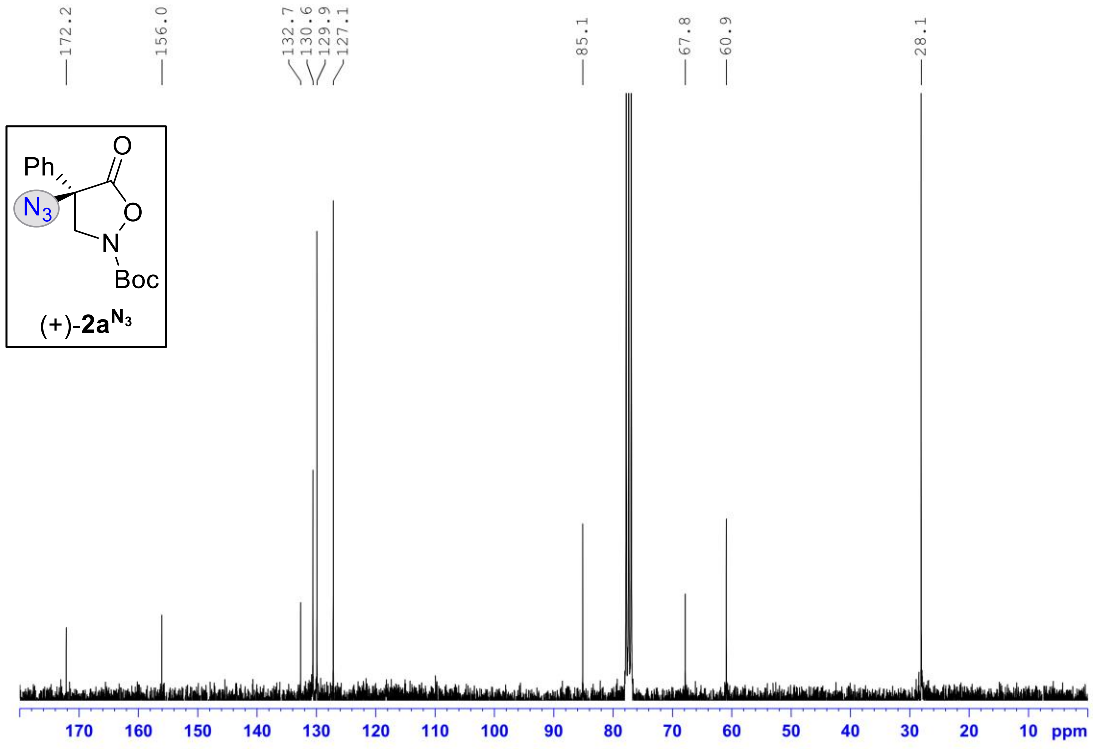
172.2
156.0
132.7
130.6
129.9
127.1
85.1
67.8
60.9
28.1
Ph
O
N_3
O
N
Boc
(+)-2a^{N_3}
170
160
150
140
130
120
110
100
90
80
70
60
50
40
30
20
10
ppm

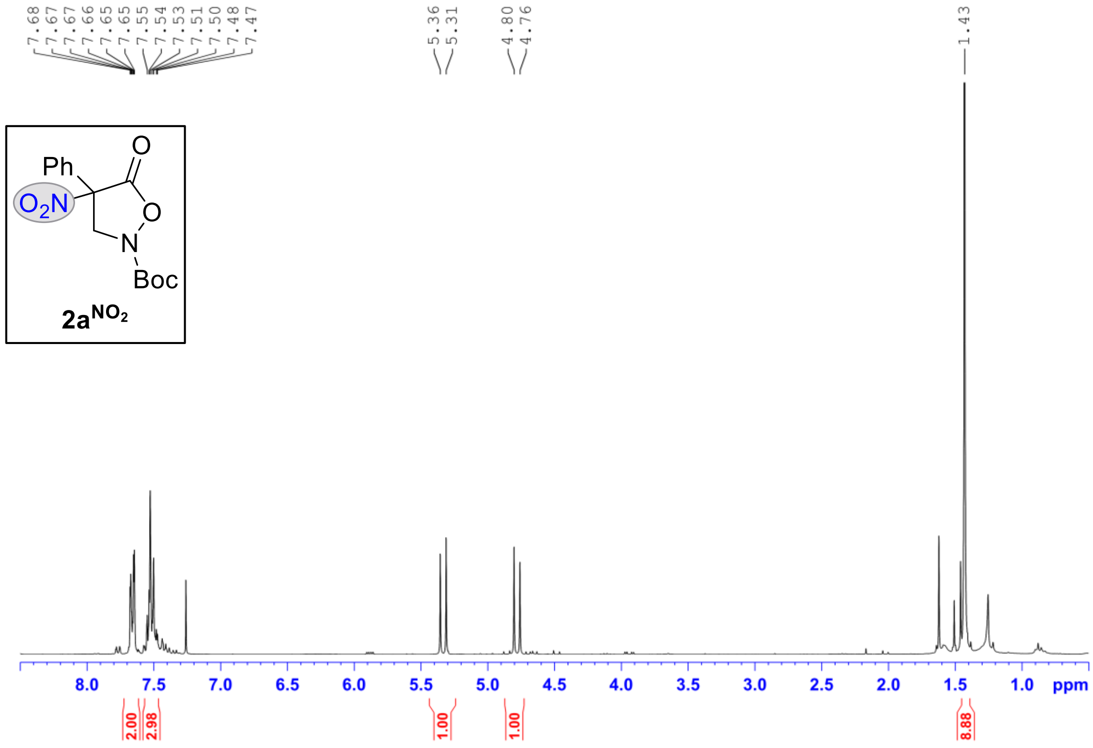
7.68
7.67
7.67
7.66
7.65
7.65
7.55
7.54
7.53
7.51
7.50
7.48
7.47
5.36
5.31
4.80
4.76
1.43
Ph
O
O_2N
O
N
Boc
2a^{NO_2}
8.0
7.5
7.0
6.5
6.0
5.5
5.0
4.5
4.0
3.5
3.0
2.5
2.0
1.5
1.0
ppm
2.00
2.98
1.00
1.00
8.88

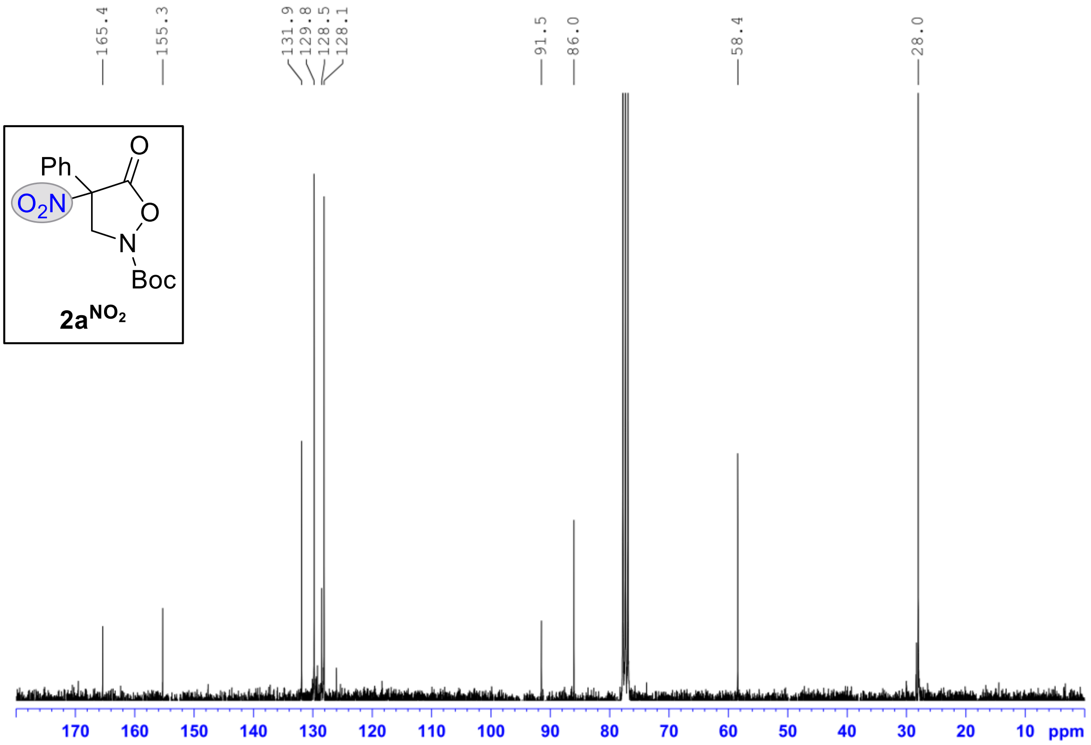

165.4
155.3
131.9
129.8
128.5
128.1
91.5
86.0
58.4
28.0
Ph
O2N
O
N
Boc
2a^NO2
170 160 150 140 130 120 110 100 90 80 70 60 50 40 30 20 10 ppm

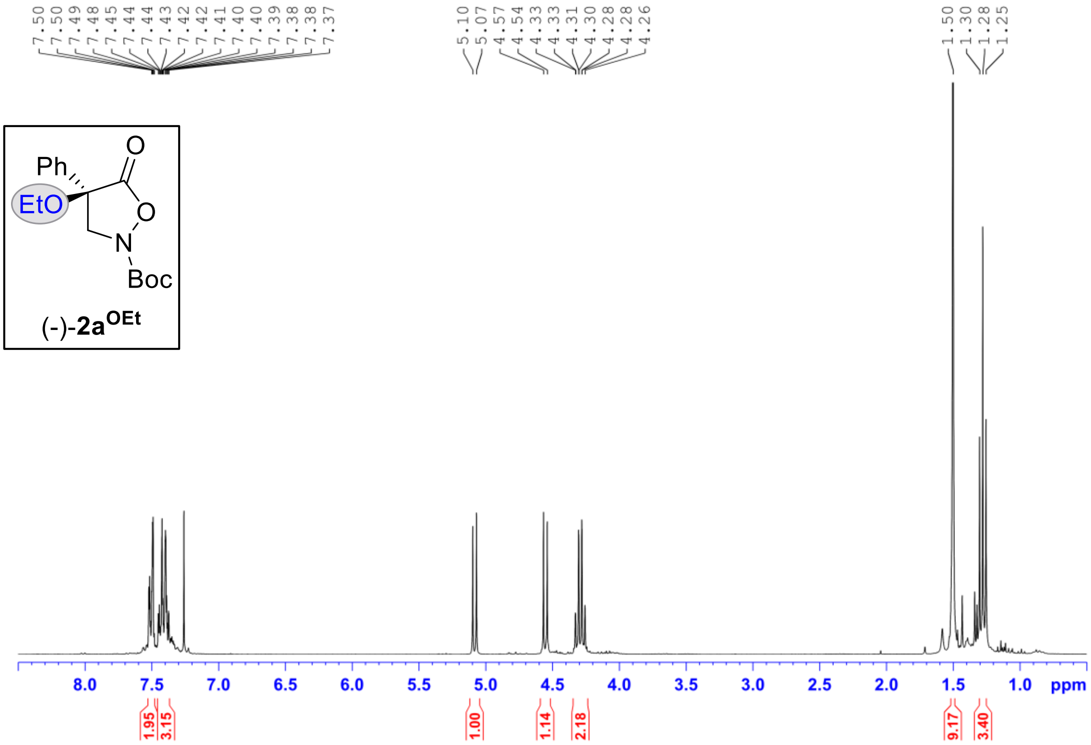

7.50 7.50 7.49 7.48 7.45 7.44 7.44 7.43 7.42 7.42 7.41 7.40 7.40 7.39 7.38 7.38 7.37
5.10 5.07 4.57 4.54 4.33 4.33 4.31 4.30 4.28 4.28 4.26
1.50 1.30 1.28 1.25
Ph
EtO
O
N
Boc
(-)-2a^OEt
8.0 7.5 7.0 6.5 6.0 5.5 5.0 4.5 4.0 3.5 3.0 2.5 2.0 1.5 1.0 ppm
1.95 3.15 1.00 1.14 2.18 9.17 3.40

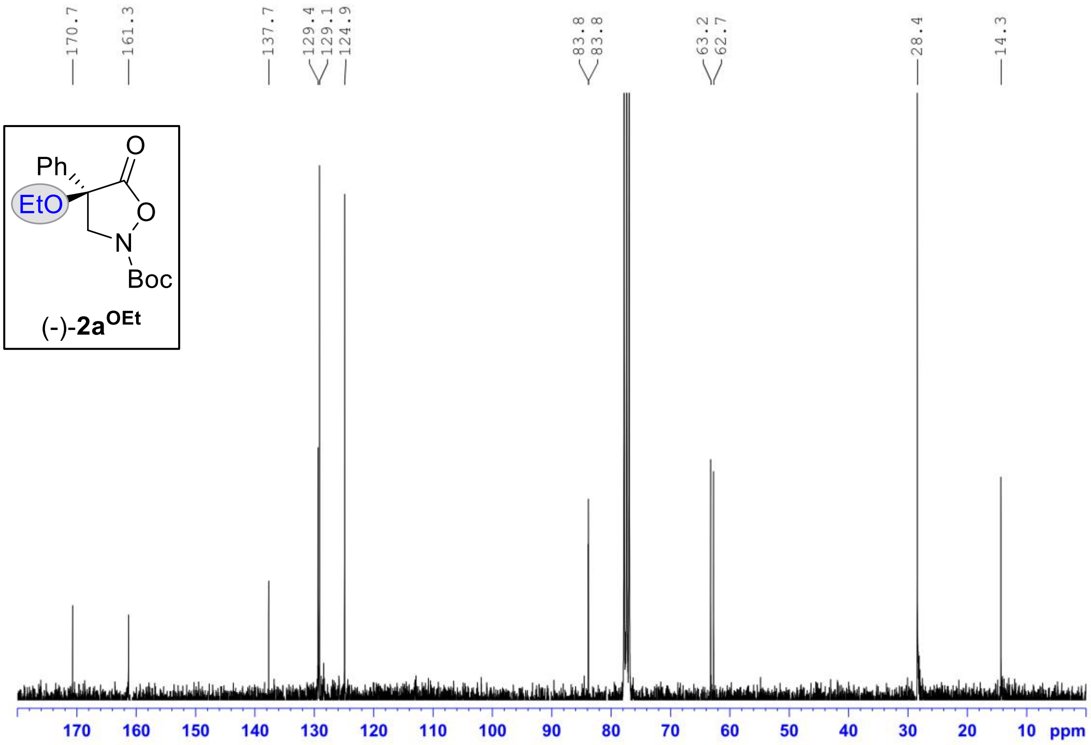

170.7
161.3
137.7
129.4
129.1
124.9
83.8
83.8
63.2
62.7
28.4
14.3
Ph
EtO
O
O
N
Boc
(-)-2a^OEt
170
160
150
140
130
120
110
100
90
80
70
60
50
40
30
20
10
ppm

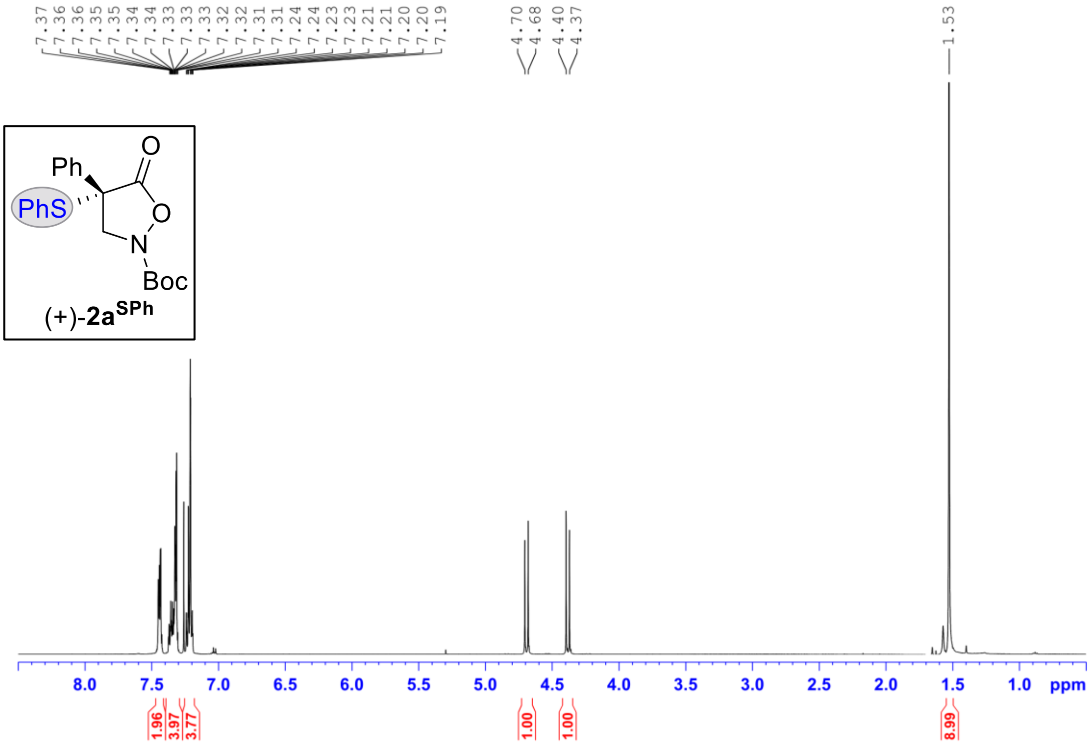

7.37
7.36
7.36
7.35
7.35
7.34
7.34
7.33
7.33
7.33
7.32
7.31
7.31
7.24
7.24
7.23
7.23
7.21
7.21
7.20
7.20
7.19
4.70
4.68
4.40
4.37
1.53
Ph
PhS
O
O
N
Boc
(+)-2a^SPh
8.0
7.5
7.0
6.5
6.0
5.5
5.0
4.5
4.0
3.5
3.0
2.5
2.0
1.5
1.0
ppm
1.96
3.97
3.77
1.00
1.00
8.99

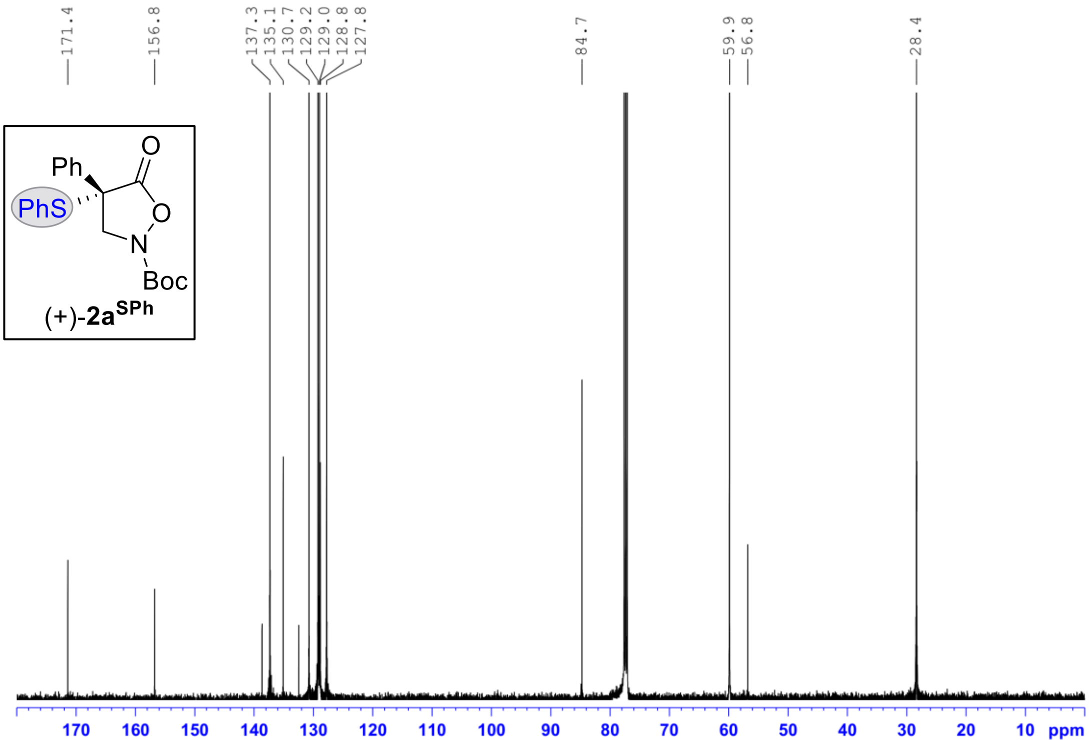
171.4
156.8
137.3
135.1
130.7
129.2
129.0
128.8
127.8
84.7
59.9
56.8
28.4
Ph
O
PhS
O
N
Boc
(+)-2a^SPh
170 160 150 140 130 120 110 100 90 80 70 60 50 40 30 20 10 ppm

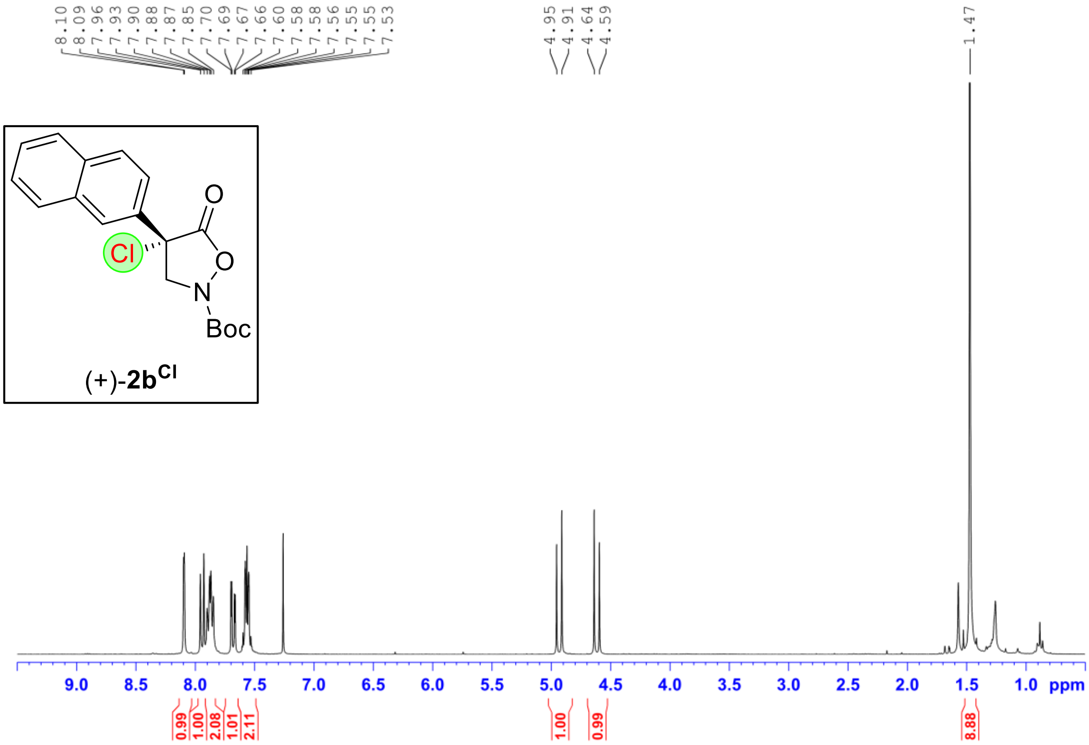
8.10
8.09
7.96
7.93
7.90
7.88
7.87
7.85
7.70
7.69
7.67
7.66
7.60
7.58
7.58
7.56
7.55
7.55
7.53
4.95
4.91
4.64
4.59
1.47
O
Cl
O
N
Boc
(+)-2b^Cl
9.0 8.5 8.0 7.5 7.0 6.5 6.0 5.5 5.0 4.5 4.0 3.5 3.0 2.5 2.0 1.5 1.0 ppm
0.99
1.00
2.08
1.01
2.11
1.00
0.99
8.88

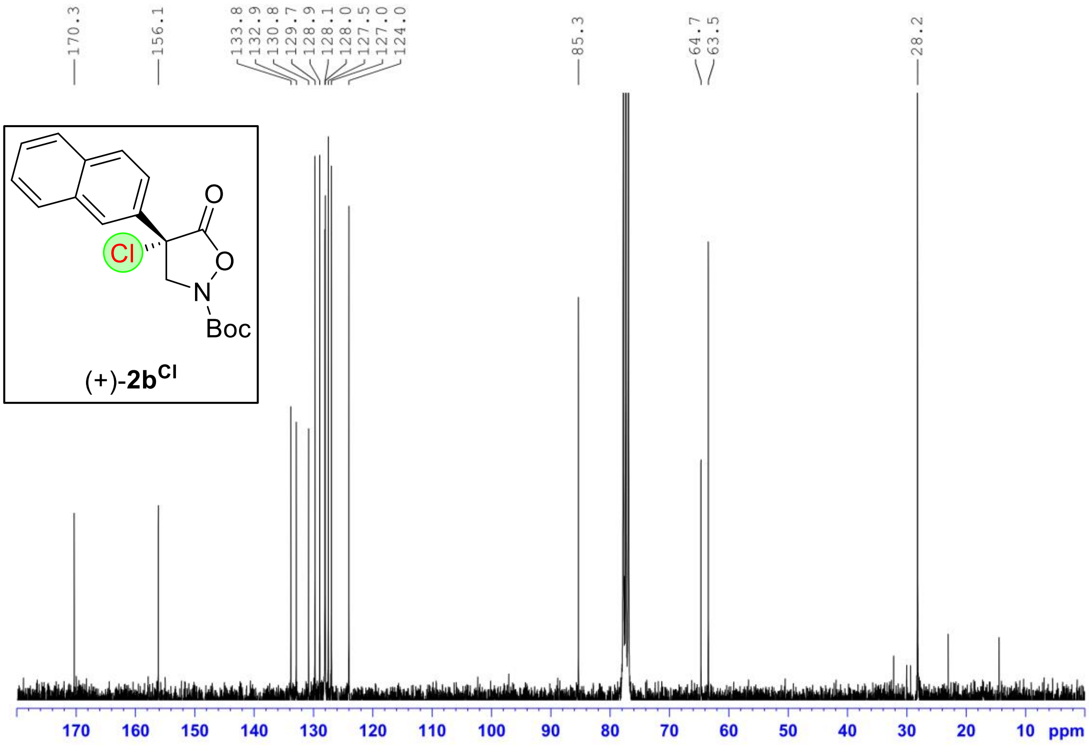

170.3
156.1
133.8
132.9
130.8
129.7
128.9
128.1
128.0
127.5
127.0
124.0
85.3
64.7
63.5
28.2
Cl
O
O
N
Boc
(+)-2b^Cl
170 160 150 140 130 120 110 100 90 80 70 60 50 40 30 20 10 ppm

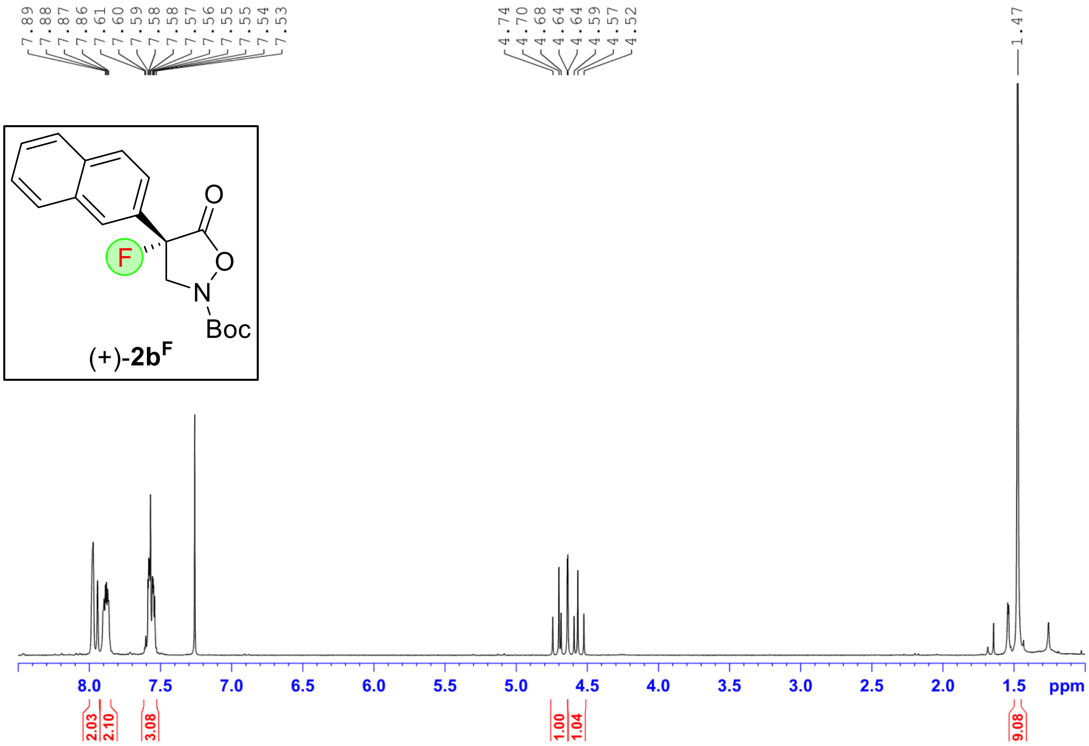

7.89
7.88
7.87
7.86
7.61
7.60
7.59
7.58
7.58
7.57
7.56
7.55
7.55
7.54
7.53
4.74
4.70
4.68
4.64
4.64
4.59
4.57
4.52
1.47
F
O
O
N
Boc
(+)-2b^F
8.0 7.5 7.0 6.5 6.0 5.5 5.0 4.5 4.0 3.5 3.0 2.5 2.0 1.5 ppm
2.03
2.10
3.08
1.00
1.04
9.08

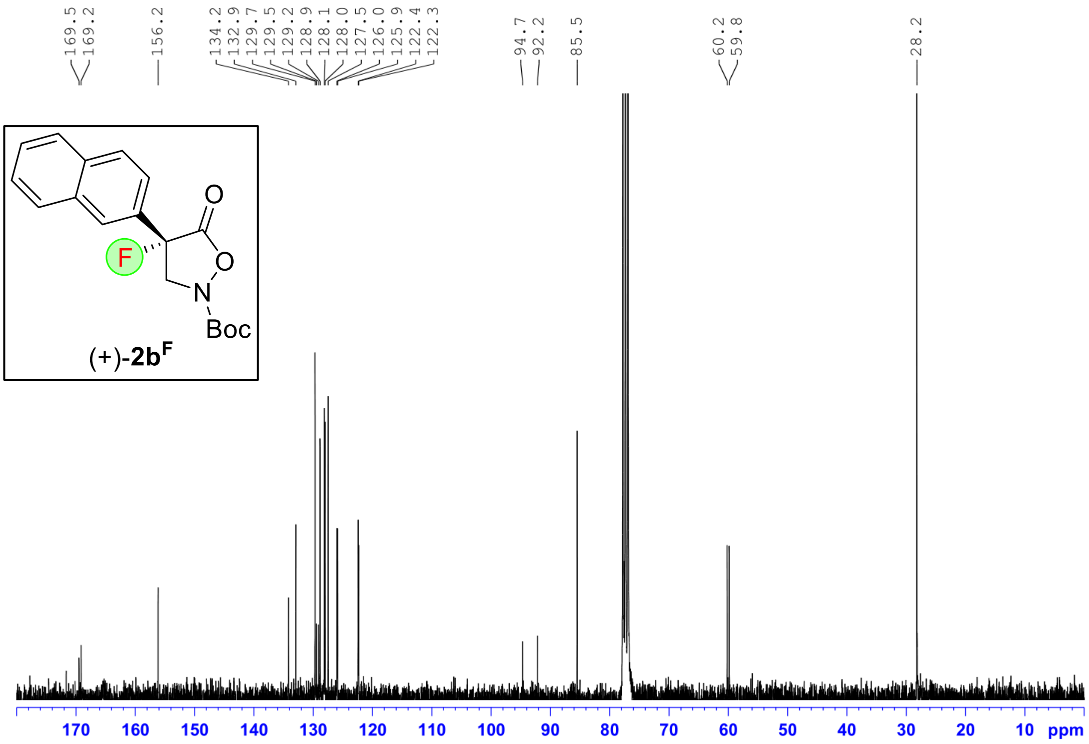
169.5
169.2
156.2
134.2
132.9
129.7
129.5
129.2
128.9
128.1
128.0
127.5
126.0
125.9
122.4
122.3
94.7
92.2
85.5
60.2
59.8
28.2
(+)-2b^F
Boc
170 160 150 140 130 120 110 100 90 80 70 60 50 40 30 20 10 ppm

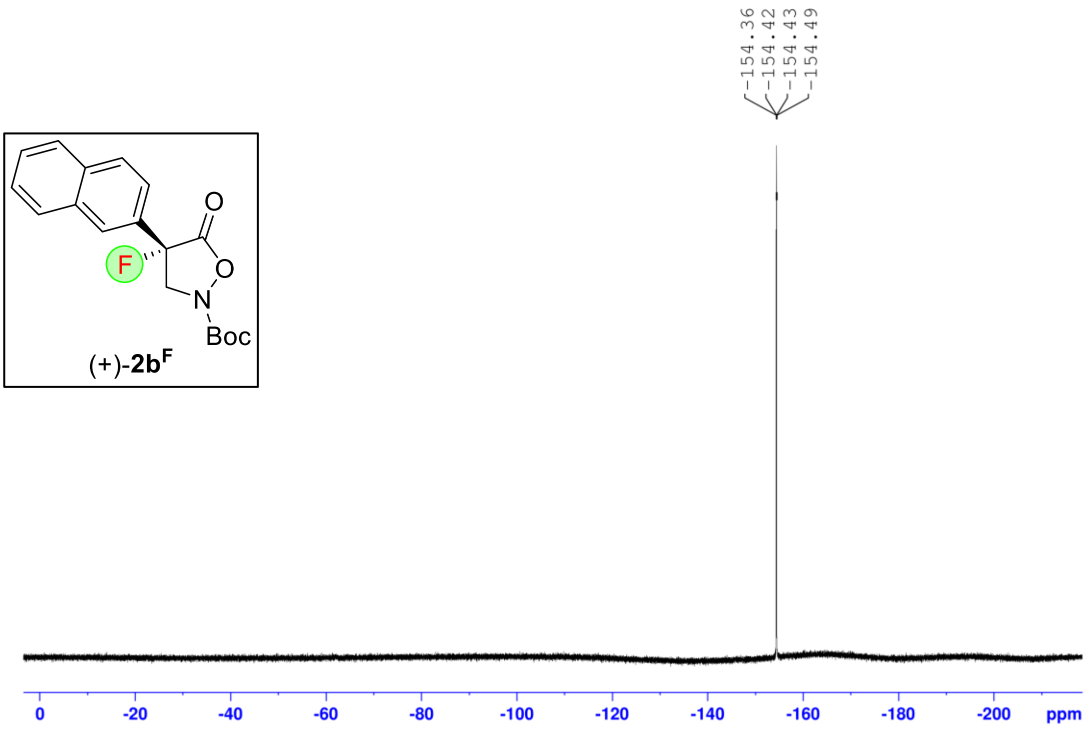
-154.36
-154.42
-154.43
-154.49
(+)-2b^F
Boc
0 -20 -40 -60 -80 -100 -120 -140 -160 -180 -200 ppm

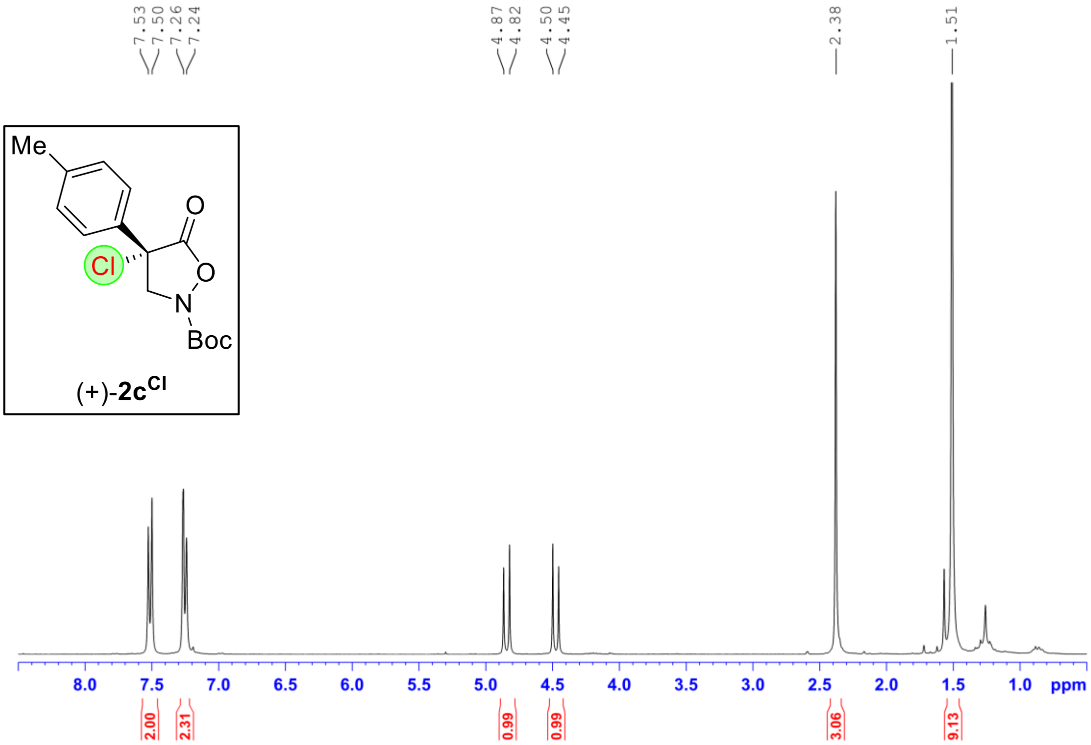
7.53
7.50
7.26
7.24
4.87
4.82
4.50
4.45
2.38
1.51
Me
O
Cl
O
N
Boc
(+)-2c^Cl
8.0 7.5 7.0 6.5 6.0 5.5 5.0 4.5 4.0 3.5 3.0 2.5 2.0 1.5 1.0 ppm
2.00
2.31
0.99
0.99
3.06
9.13

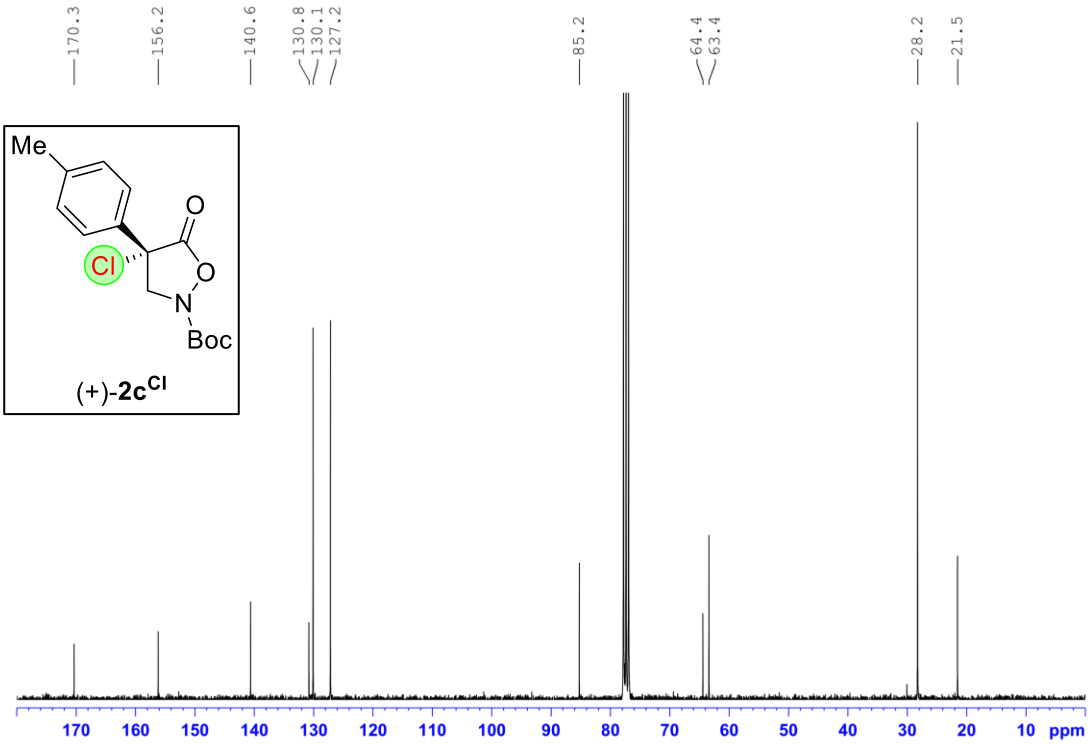
170.3
156.2
140.6
130.8
130.1
127.2
85.2
64.4
63.4
28.2
21.5
Me
O
Cl
O
N
Boc
(+)-2c^Cl
170 160 150 140 130 120 110 100 90 80 70 60 50 40 30 20 10 ppm

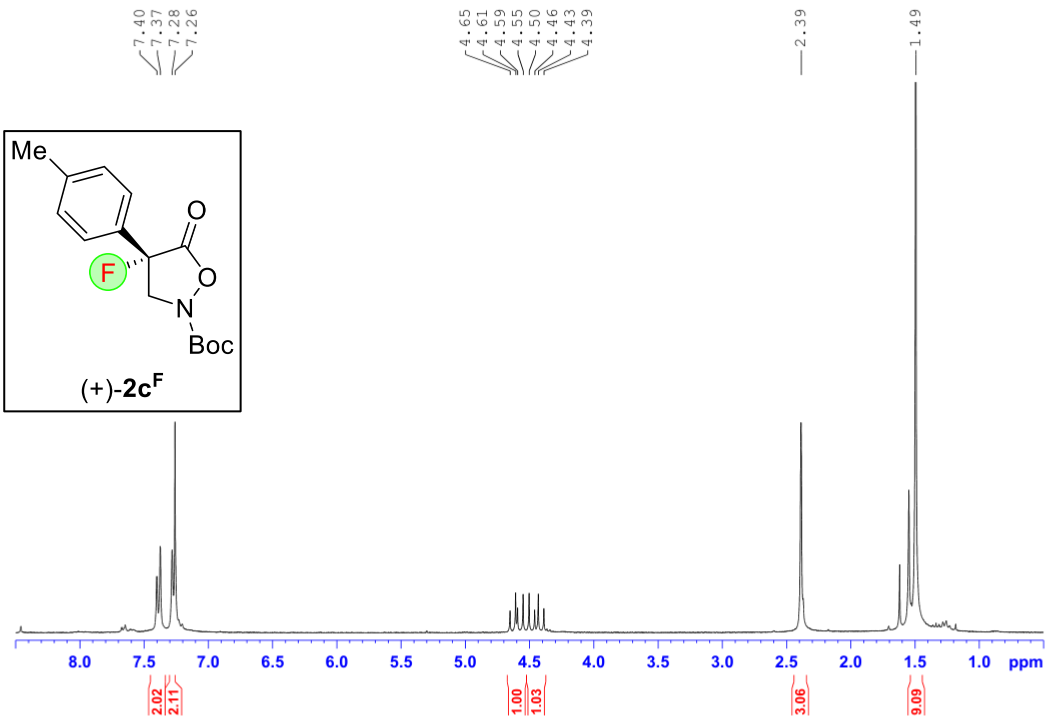
7.40
7.37
7.28
7.26
4.65
4.61
4.59
4.55
4.50
4.46
4.43
4.39
2.39
1.49
Me
O
F
O
N
Boc
(+)-2c^F
8.0
7.5
7.0
6.5
6.0
5.5
5.0
4.5
4.0
3.5
3.0
2.5
2.0
1.5
1.0
ppm
2.02
2.11
1.00
1.03
3.06
9.09

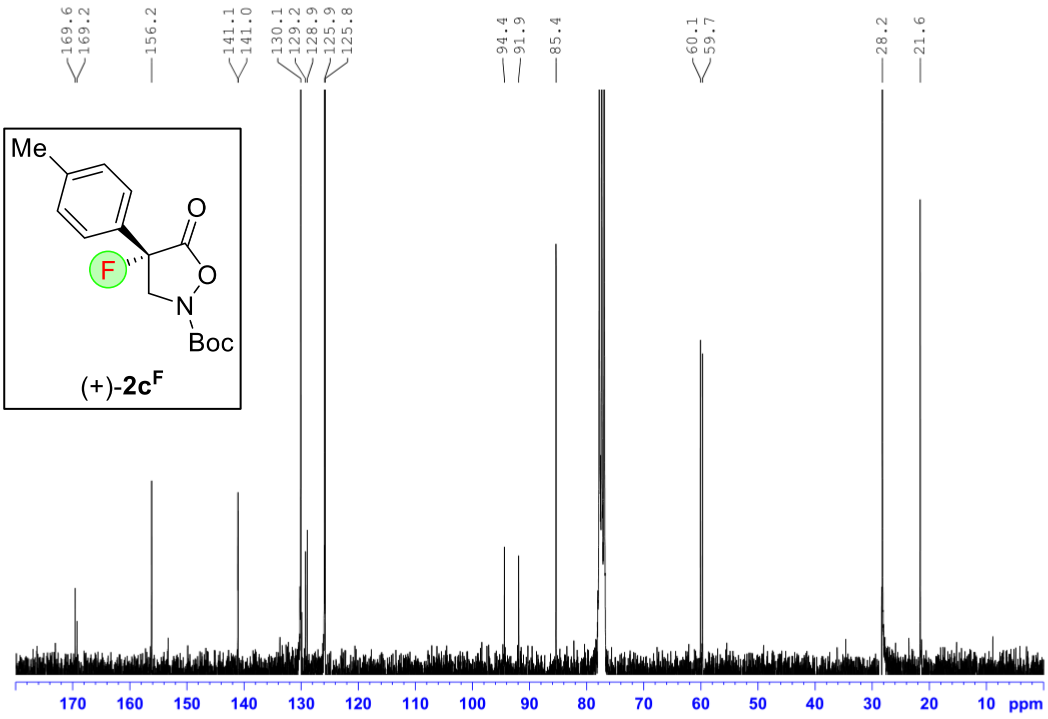
169.6
169.2
156.2
141.1
141.0
130.1
129.2
128.9
125.9
125.8
94.4
91.9
85.4
60.1
59.7
28.2
21.6
Me
O
F
O
N
Boc
(+)-2c^F
170
160
150
140
130
120
110
100
90
80
70
60
50
40
30
20
10
ppm

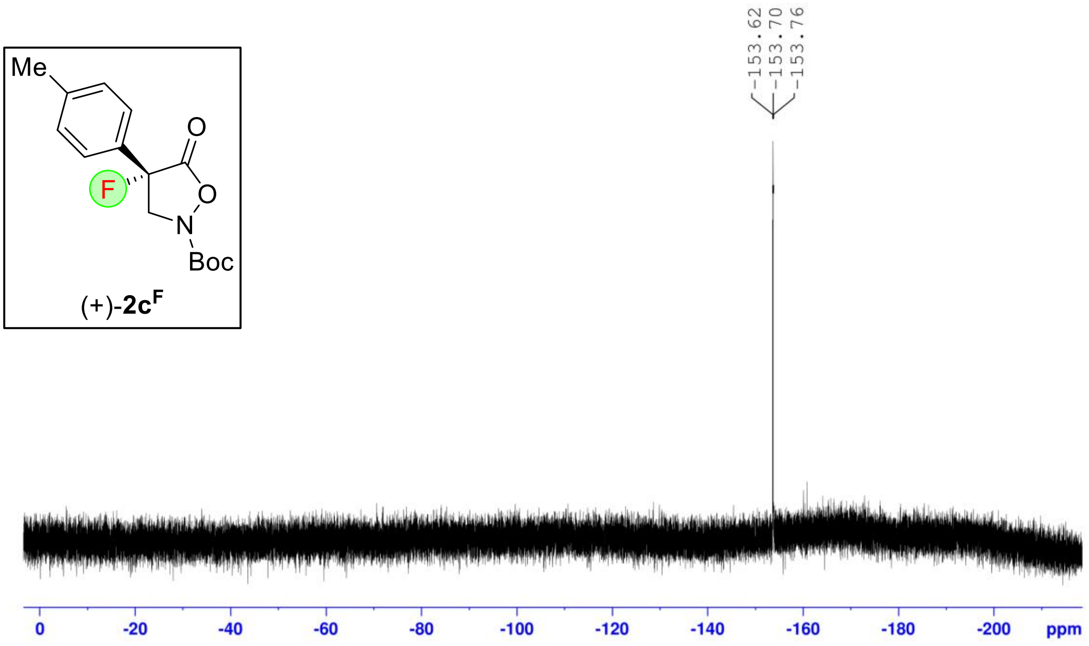
Me
F
O
O
N
Boc
(+)-2c^F
-153.62
-153.70
-153.76
0
-20
-40
-60
-80
-100
-120
-140
-160
-180
-200
ppm

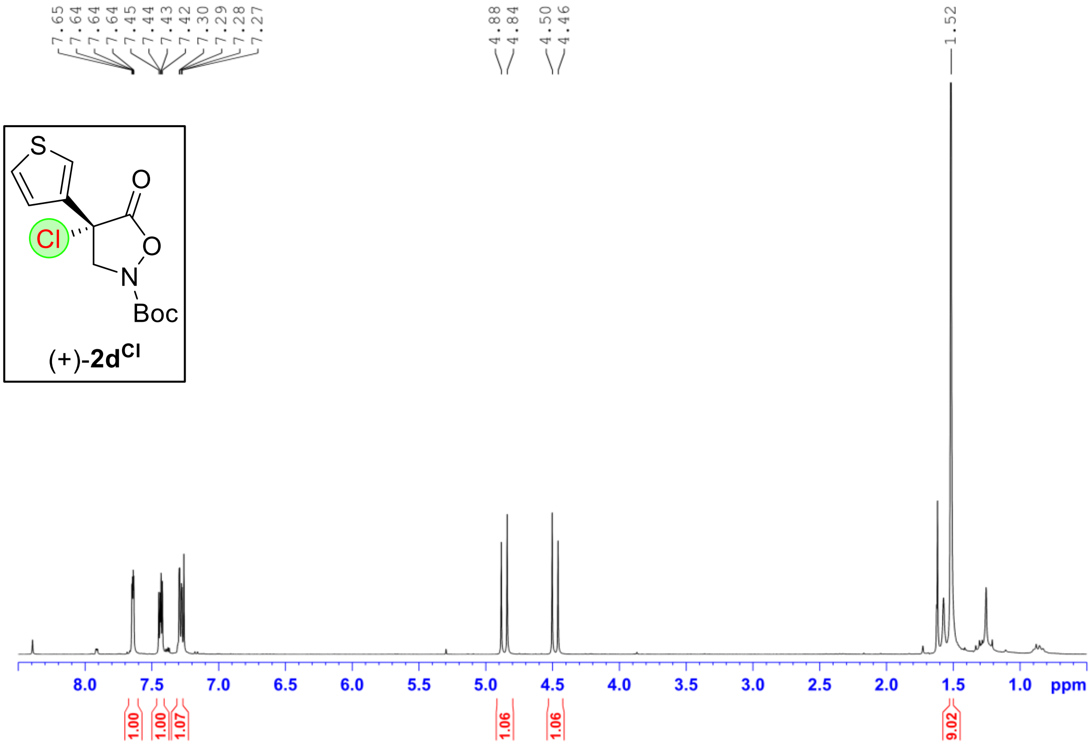
S
Cl
O
O
N
Boc
(+)-2d^Cl
7.65
7.64
7.64
7.64
7.45
7.44
7.43
7.42
7.30
7.29
7.28
7.27
4.88
4.84
4.50
4.46
1.52
8.0
7.5
7.0
6.5
6.0
5.5
5.0
4.5
4.0
3.5
3.0
2.5
2.0
1.5
1.0
ppm
1.00
1.00
1.07
1.06
1.06
9.02

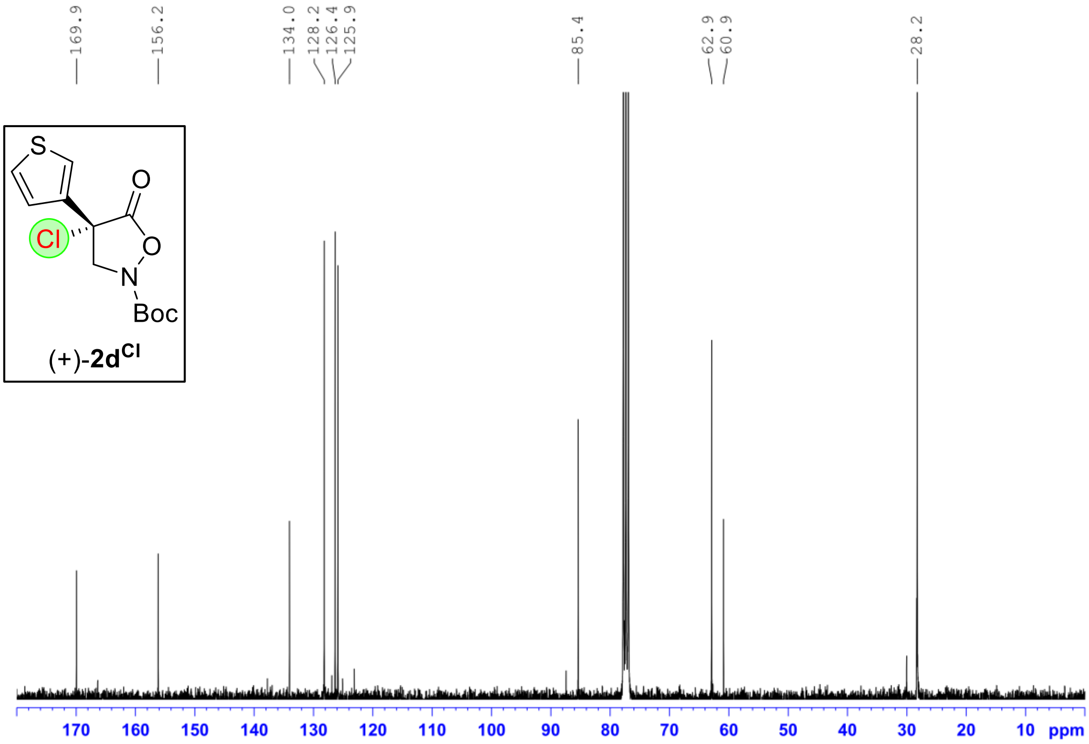
169.9
156.2
134.0
128.2
126.4
125.9
85.4
62.9
60.9
28.2
S
O
Cl
O
N
Boc
(+)-2d^Cl
170
160
150
140
130
120
110
100
90
80
70
60
50
40
30
20
10
ppm

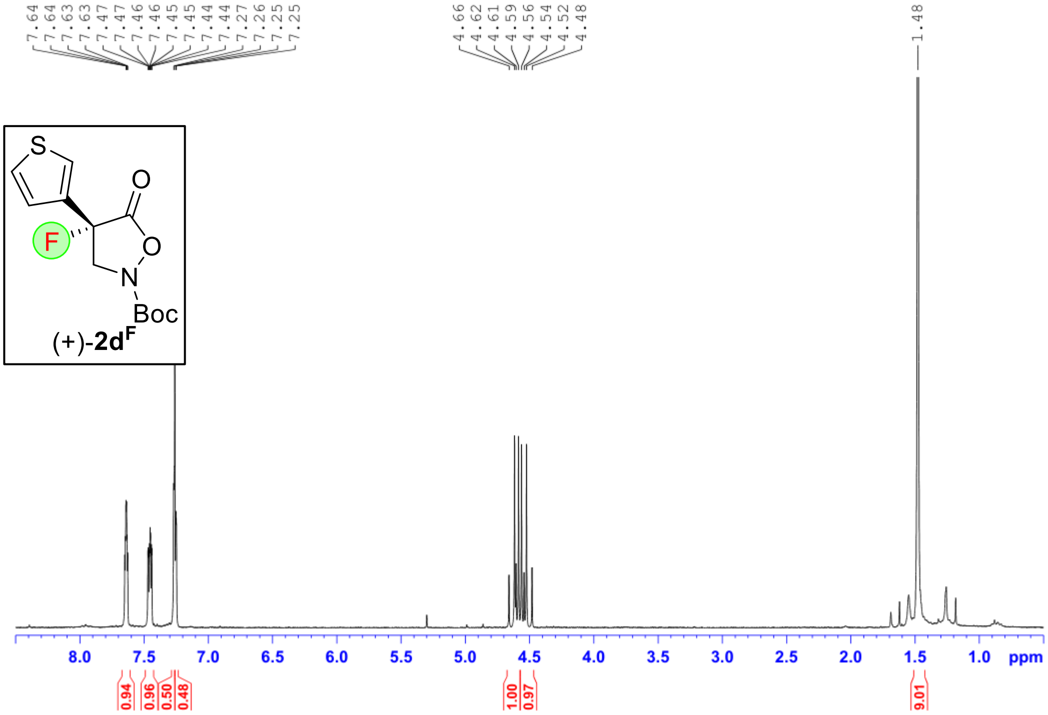
7.64
7.64
7.63
7.63
7.47
7.47
7.46
7.46
7.45
7.45
7.44
7.44
7.27
7.26
7.25
7.25
4.66
4.62
4.61
4.59
4.56
4.54
4.52
4.48
1.48
S
O
F
O
N
Boc
(+)-2d^F
8.0
7.5
7.0
6.5
6.0
5.5
5.0
4.5
4.0
3.5
3.0
2.5
2.0
1.5
1.0
ppm
0.94
0.96
0.50
0.48
1.00
0.97
9.01

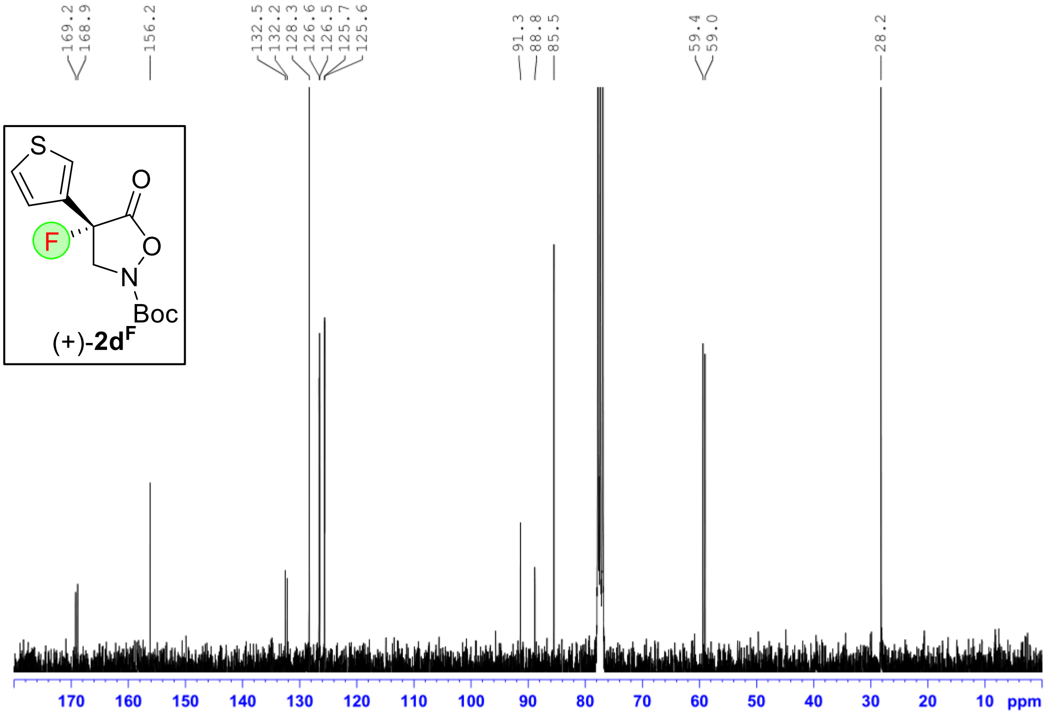
169.2
168.9
156.2
132.5
132.2
128.3
126.6
126.5
125.7
125.6
91.3
88.8
85.5
59.4
59.0
28.2
S
O
F
O
N
Boc
(+)-2d^F
170
160
150
140
130
120
110
100
90
80
70
60
50
40
30
20
10
ppm

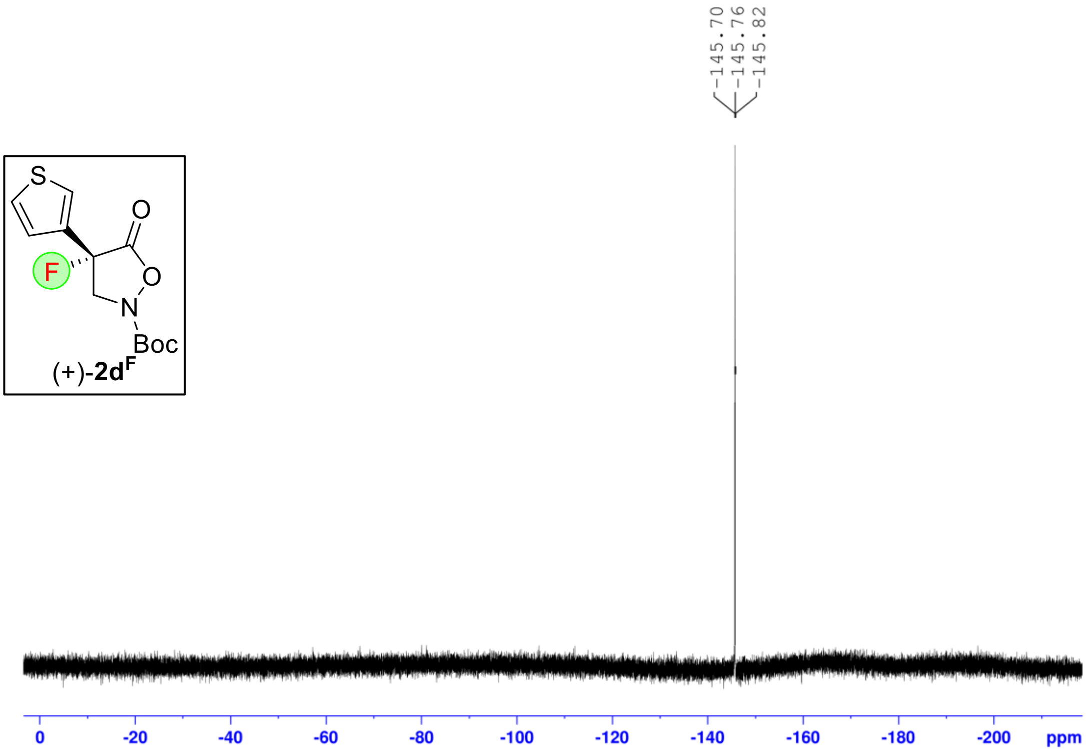
-145.70
-145.76
-145.82
S
O
F
O
N
Boc
(+)-2d^F
0
-20
-40
-60
-80
-100
-120
-140
-160
-180
-200
ppm

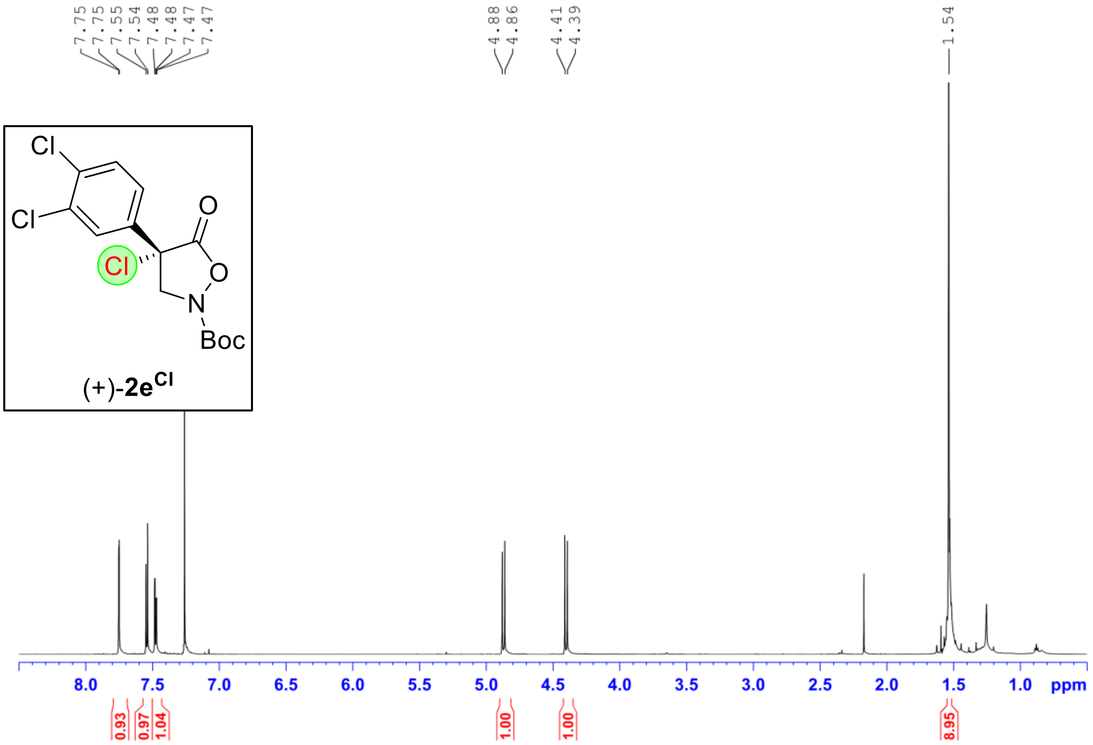
7.75
7.75
7.55
7.54
7.48
7.48
7.47
7.47
4.88
4.86
4.41
4.39
1.54
Cl
Cl
O
Cl
O
N
Boc
(+)-2e^Cl
8.0
7.5
7.0
6.5
6.0
5.5
5.0
4.5
4.0
3.5
3.0
2.5
2.0
1.5
1.0
ppm
0.93
0.97
1.04
1.00
1.00
8.95

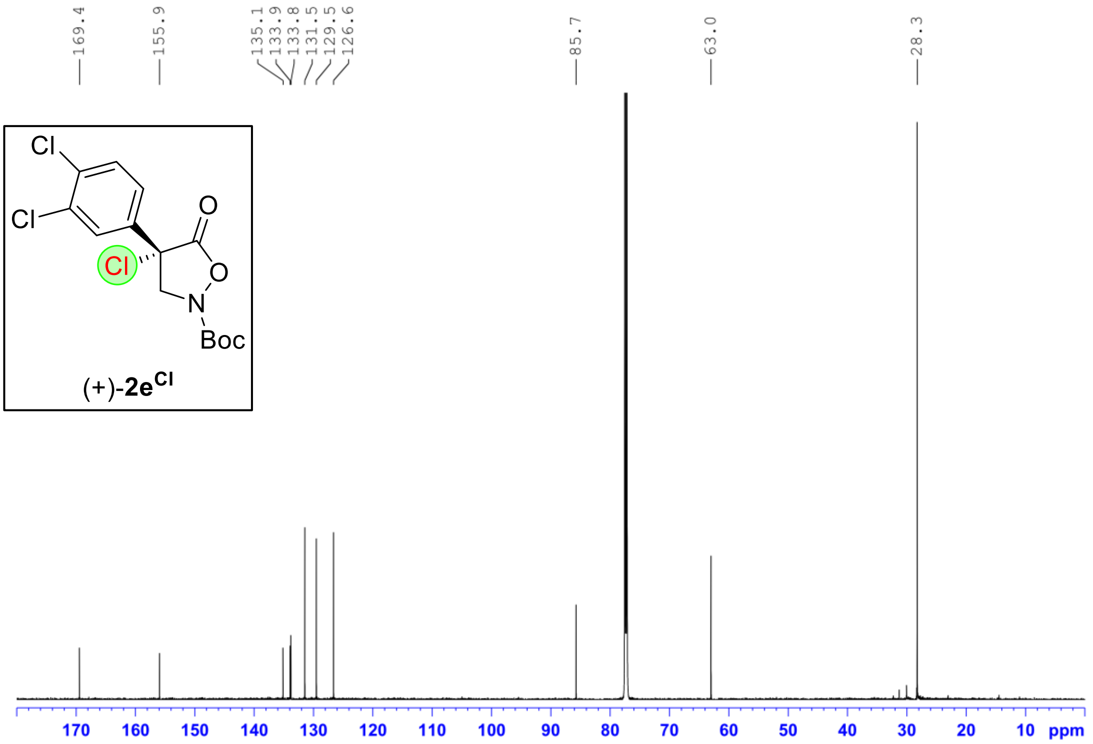
169.4
155.9
135.1
133.9
133.8
131.5
129.5
126.6
85.7
63.0
28.3
Cl
Cl
O
Cl
O
N
Boc
(+)-2e^Cl
170
160
150
140
130
120
110
100
90
80
70
60
50
40
30
20
10
ppm

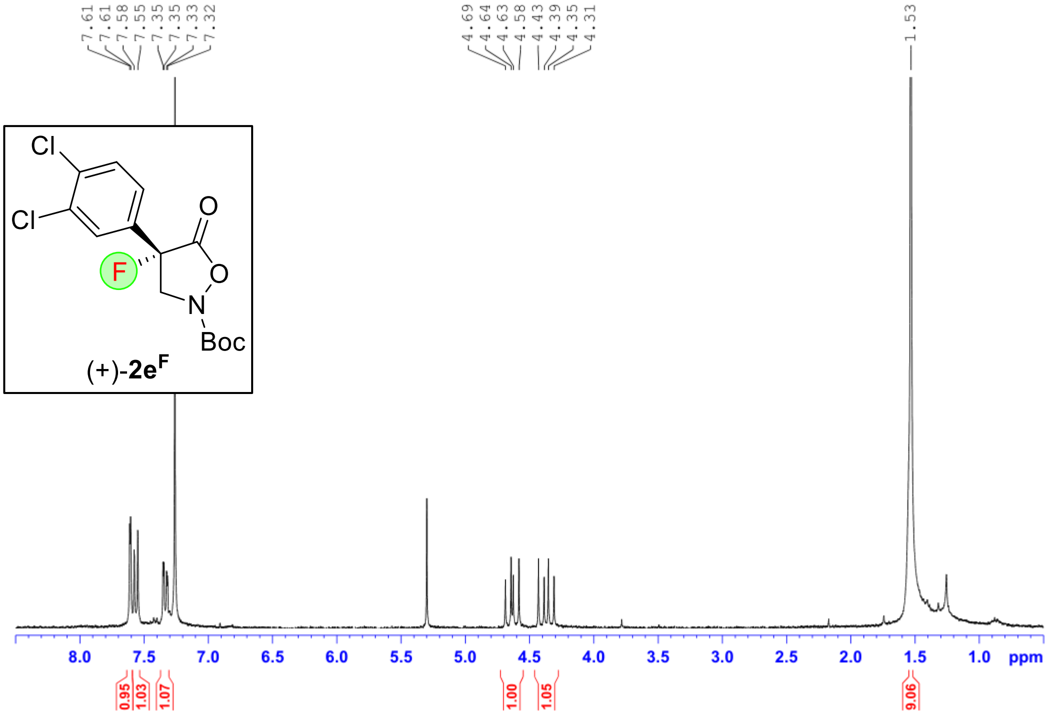
7.61
7.61
7.58
7.55
7.35
7.35
7.33
7.32
4.69
4.64
4.63
4.58
4.43
4.39
4.35
4.31
1.53
Cl
Cl
O
F
O
N
Boc
(+)-2e^F
8.0
7.5
7.0
6.5
6.0
5.5
5.0
4.5
4.0
3.5
3.0
2.5
2.0
1.5
1.0
ppm
0.95
1.03
1.07
1.00
1.05
9.06

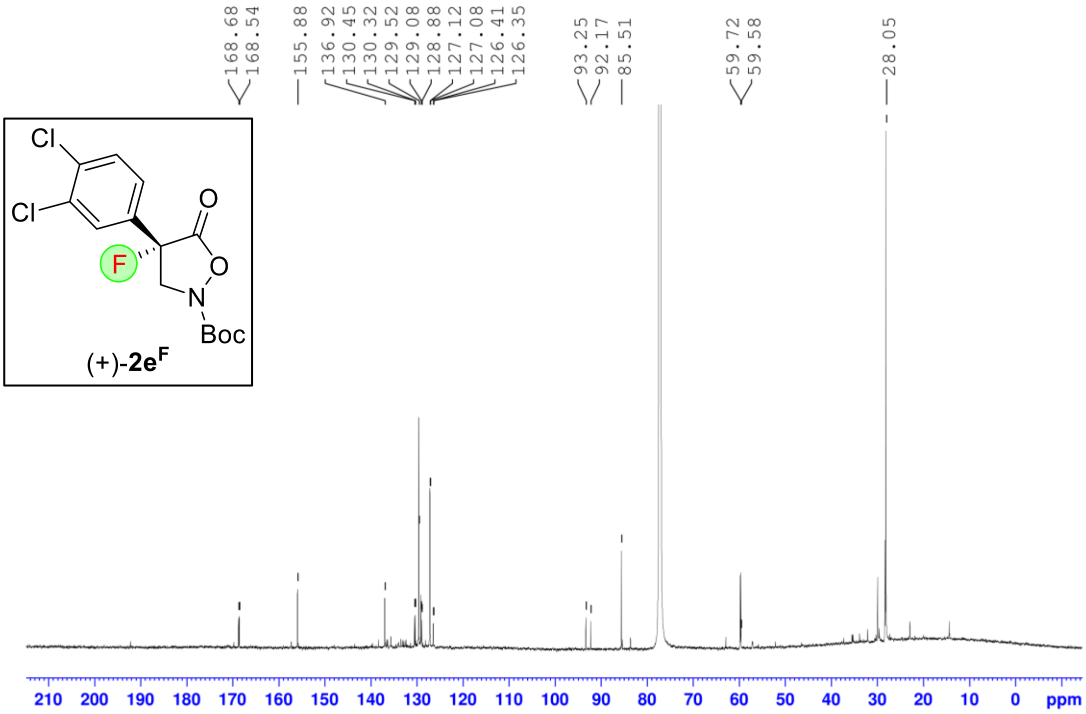
168.68
168.54
155.88
136.92
130.45
130.32
129.52
129.08
128.88
127.12
127.08
126.41
126.35
93.25
92.17
85.51
59.72
59.58
28.05
Cl
Cl
O
F
O
N
Boc
(+)-2e^F
210
200
190
180
170
160
150
140
130
120
110
100
90
80
70
60
50
40
30
20
10
0
ppm

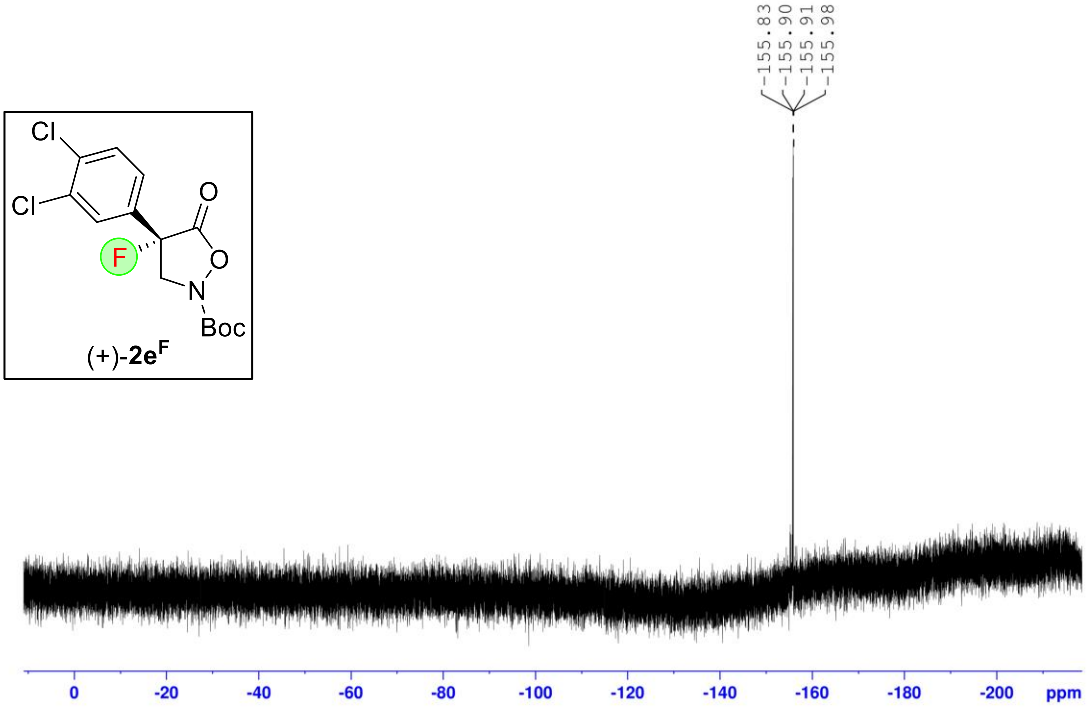

-155.83
-155.90
-155.91
-155.98
Cl
Cl
O
F
O
N
Boc
(+)-2e^F
0
-20
-40
-60
-80
-100
-120
-140
-160
-180
-200
ppm

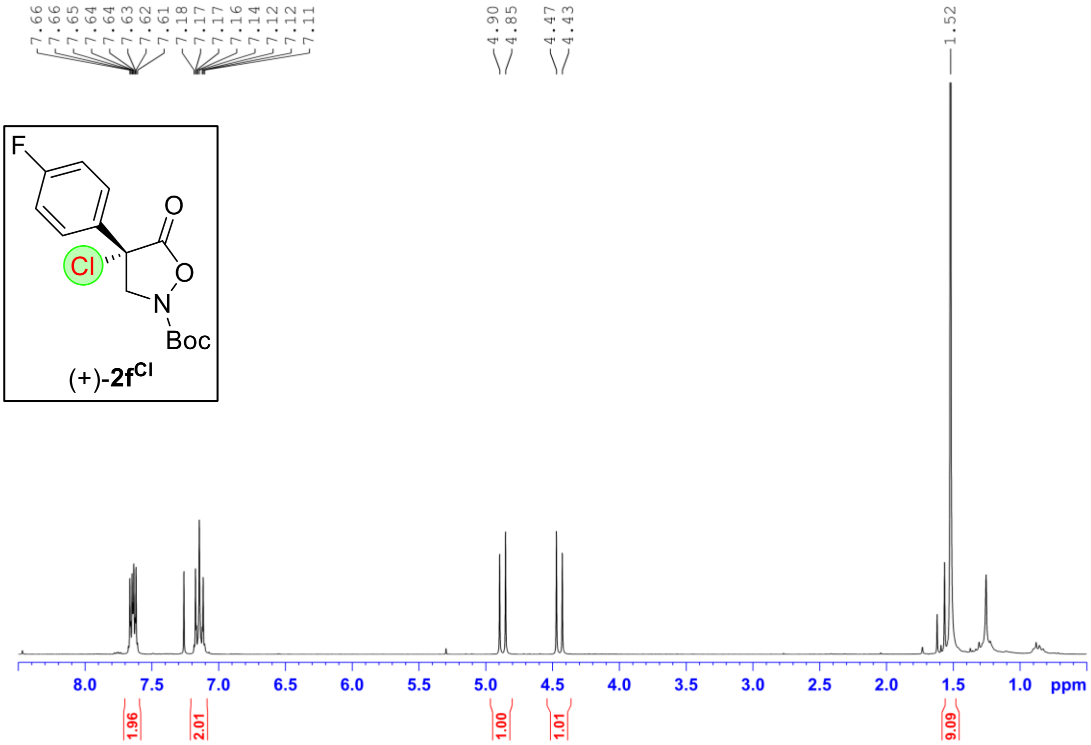

7.66
7.66
7.65
7.64
7.64
7.63
7.62
7.61
7.18
7.17
7.17
7.16
7.14
7.12
7.12
7.11
4.90
4.85
4.47
4.43
1.52
F
O
Cl
O
N
Boc
(+)-2f^Cl
8.0
7.5
7.0
6.5
6.0
5.5
5.0
4.5
4.0
3.5
3.0
2.5
2.0
1.5
1.0
ppm
1.96
2.01
1.00
1.01
9.09

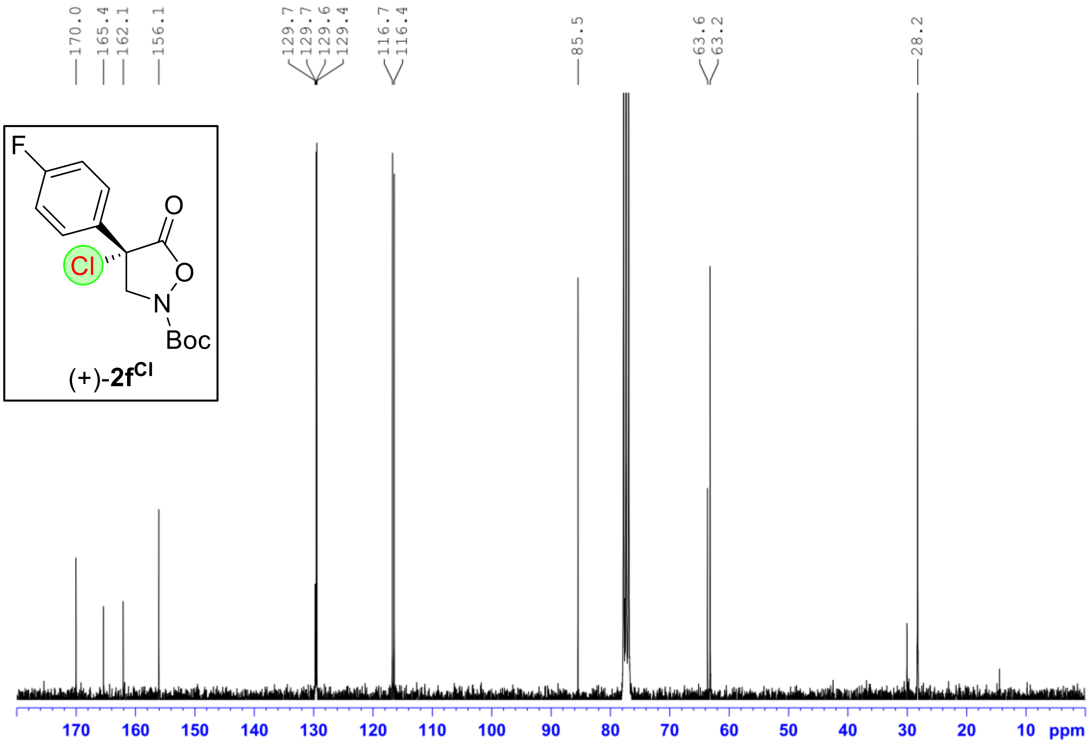
170.0
165.4
162.1
156.1
129.7
129.7
129.6
129.4
116.7
116.4
85.5
63.6
63.2
28.2
F
O
Cl
O
N
Boc
(+)-2f^{Cl}
170
160
150
140
130
120
110
100
90
80
70
60
50
40
30
20
10
ppm

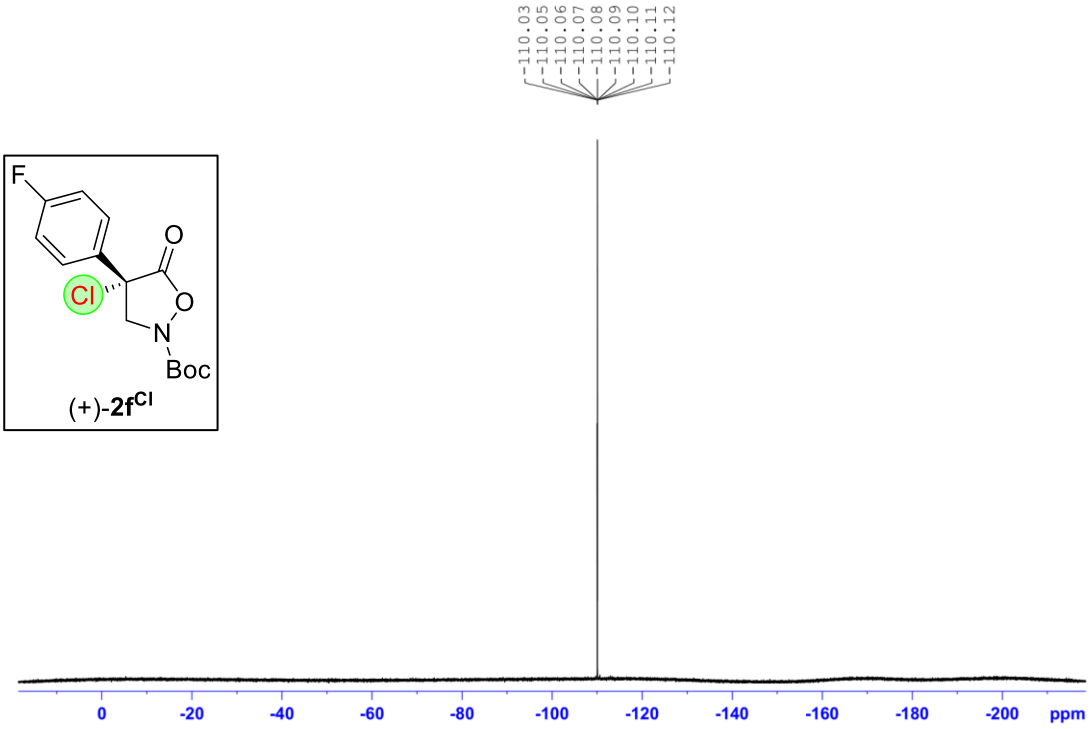
-110.03
-110.05
-110.06
-110.07
-110.08
-110.09
-110.10
-110.11
-110.12
F
O
Cl
O
N
Boc
(+)-2f^{Cl}
0
-20
-40
-60
-80
-100
-120
-140
-160
-180
-200
ppm

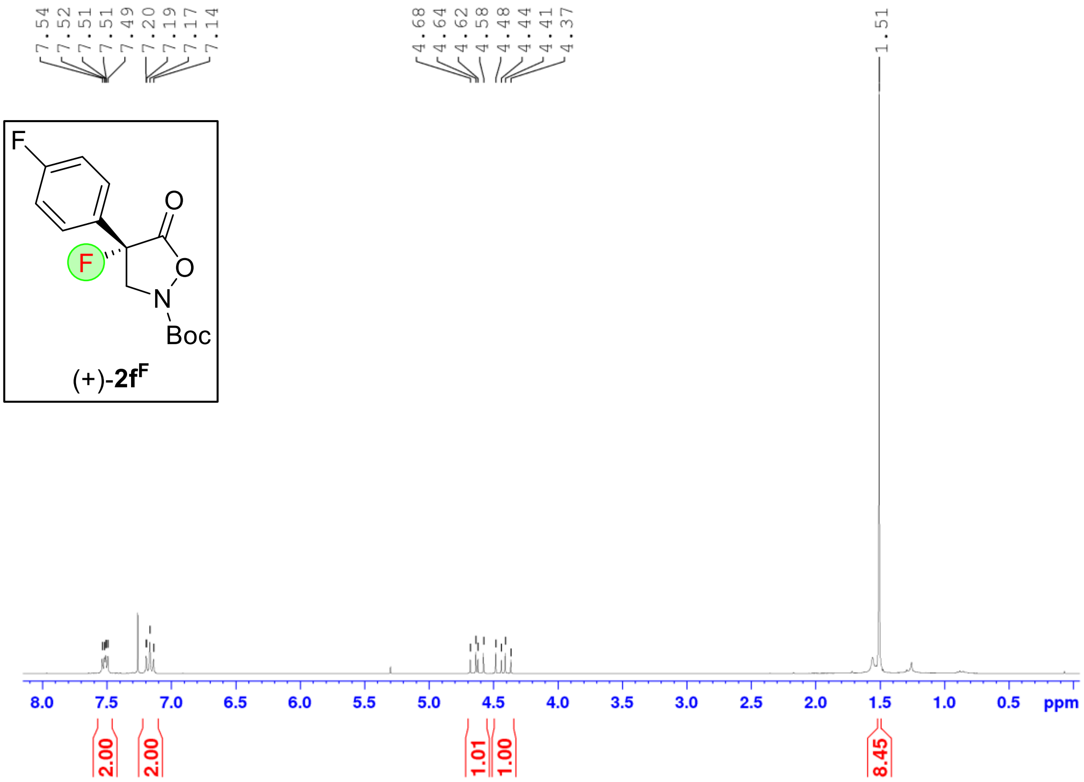
7.54
7.52
7.51
7.51
7.49
7.20
7.19
7.17
7.14
4.68
4.64
4.62
4.58
4.48
4.44
4.41
4.37
1.51
F
O
F
O
N
Boc
(+)-2f^F
8.0 7.5 7.0 6.5 6.0 5.5 5.0 4.5 4.0 3.5 3.0 2.5 2.0 1.5 1.0 0.5 ppm
2.00
2.00
1.01
1.00
8.45

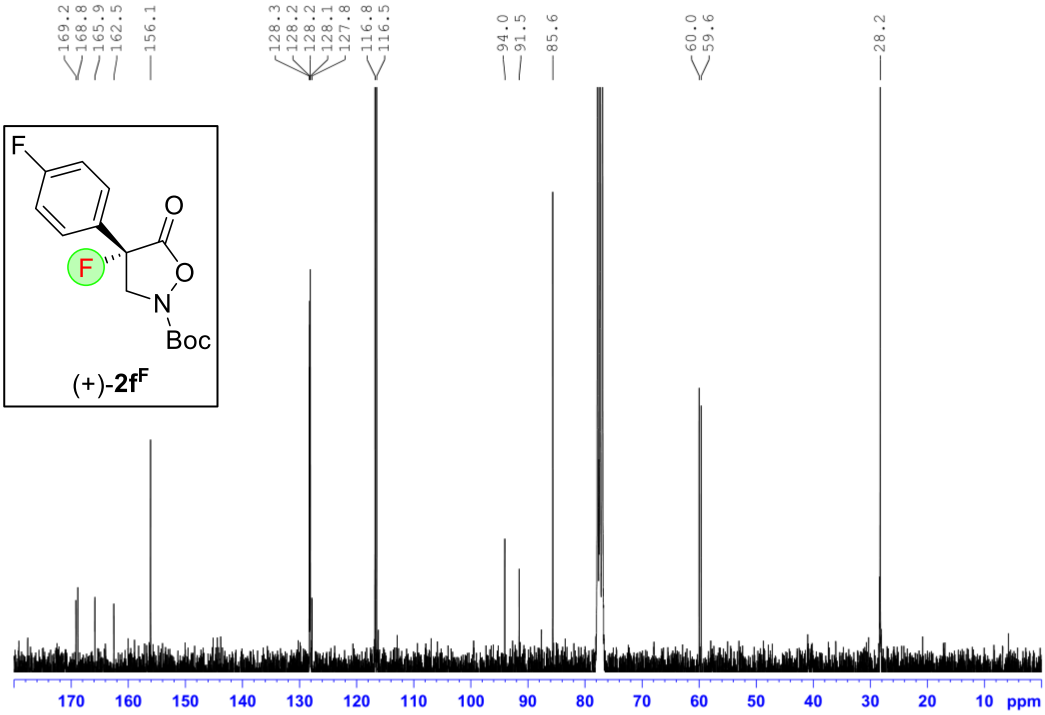
169.2
168.8
165.9
162.5
156.1
128.3
128.2
128.2
128.1
127.8
116.8
116.5
94.0
91.5
85.6
60.0
59.6
28.2
F
O
F
O
N
Boc
(+)-2f^F
170 160 150 140 130 120 110 100 90 80 70 60 50 40 30 20 10 ppm

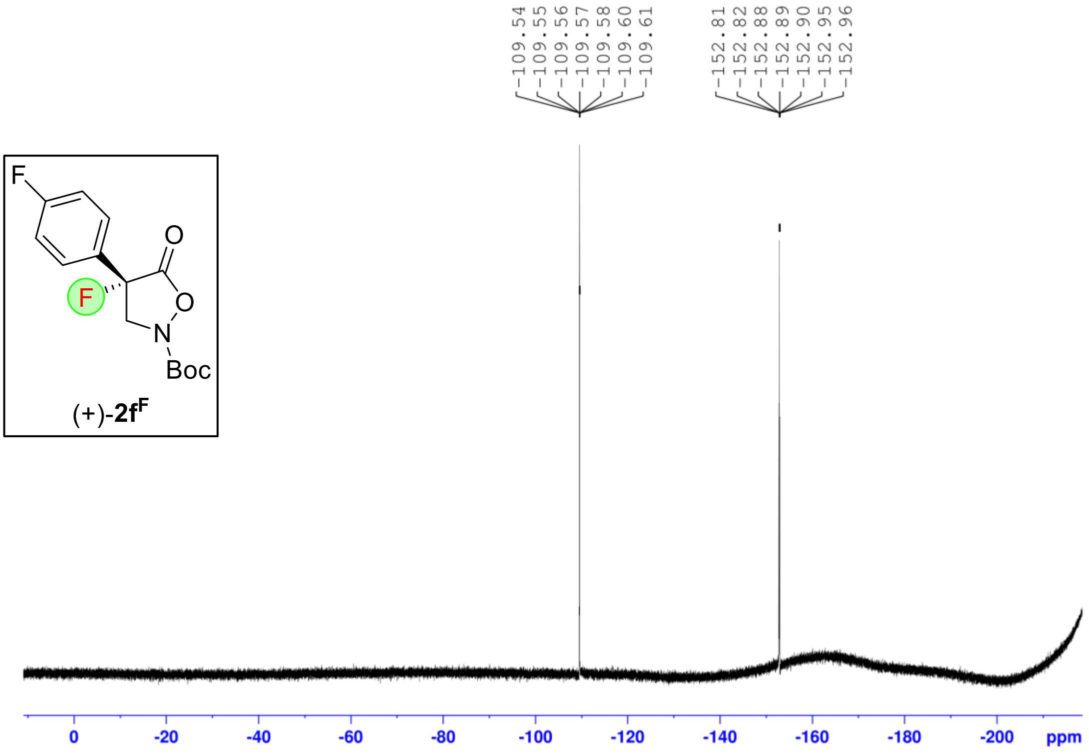
-109.54
-109.55
-109.56
-109.57
-109.58
-109.60
-109.61
-152.81
-152.82
-152.88
-152.89
-152.90
-152.95
-152.96
F
O
F
O
N
Boc
(+)-2f^F
0
-20
-40
-60
-80
-100
-120
-140
-160
-180
-200
ppm

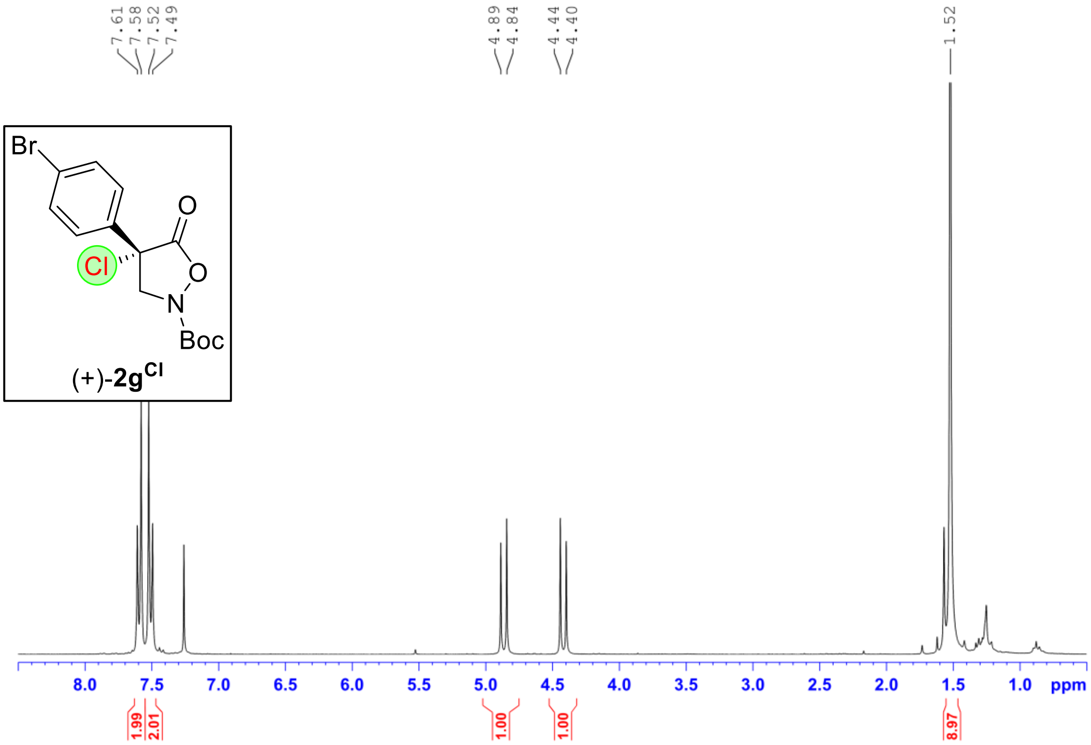
7.61
7.58
7.52
7.49
4.89
4.84
4.44
4.40
1.52
Br
O
Cl
O
N
Boc
(+)-2g^Cl
8.0
7.5
7.0
6.5
6.0
5.5
5.0
4.5
4.0
3.5
3.0
2.5
2.0
1.5
1.0
ppm
1.99
2.01
1.00
1.00
8.97

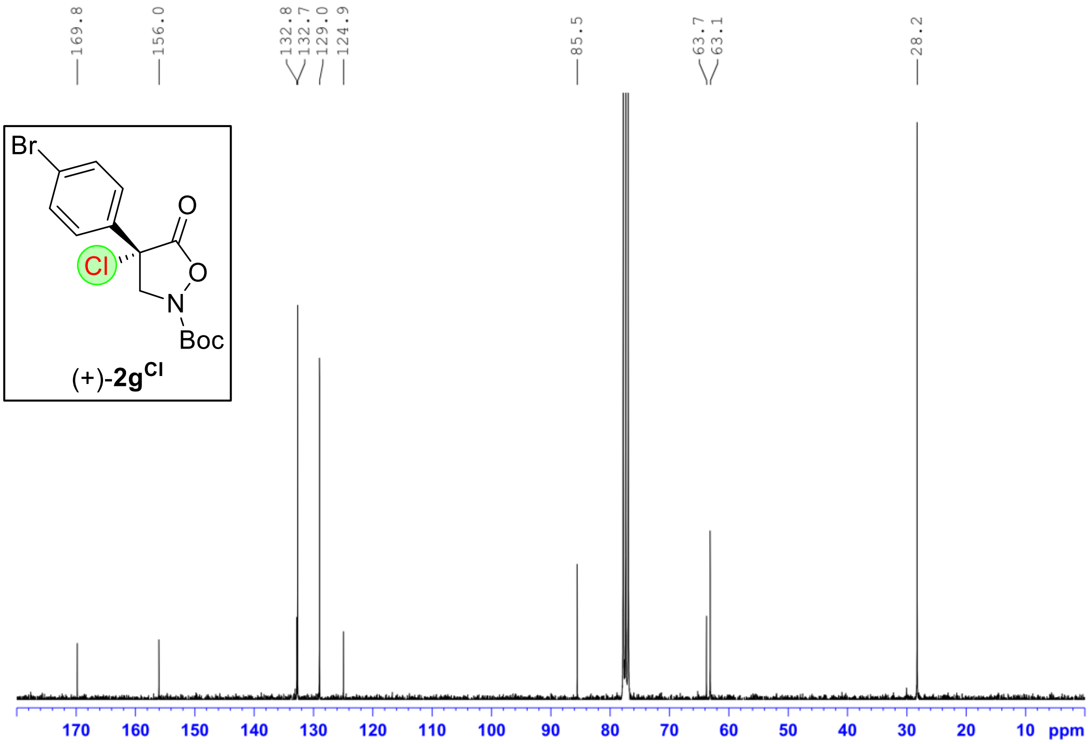
169.8
156.0
132.8
132.7
129.0
124.9
85.5
63.7
63.1
28.2
Br
O
Cl
O
N
Boc
(+)-2gCl
170
160
150
140
130
120
110
100
90
80
70
60
50
40
30
20
10
ppm

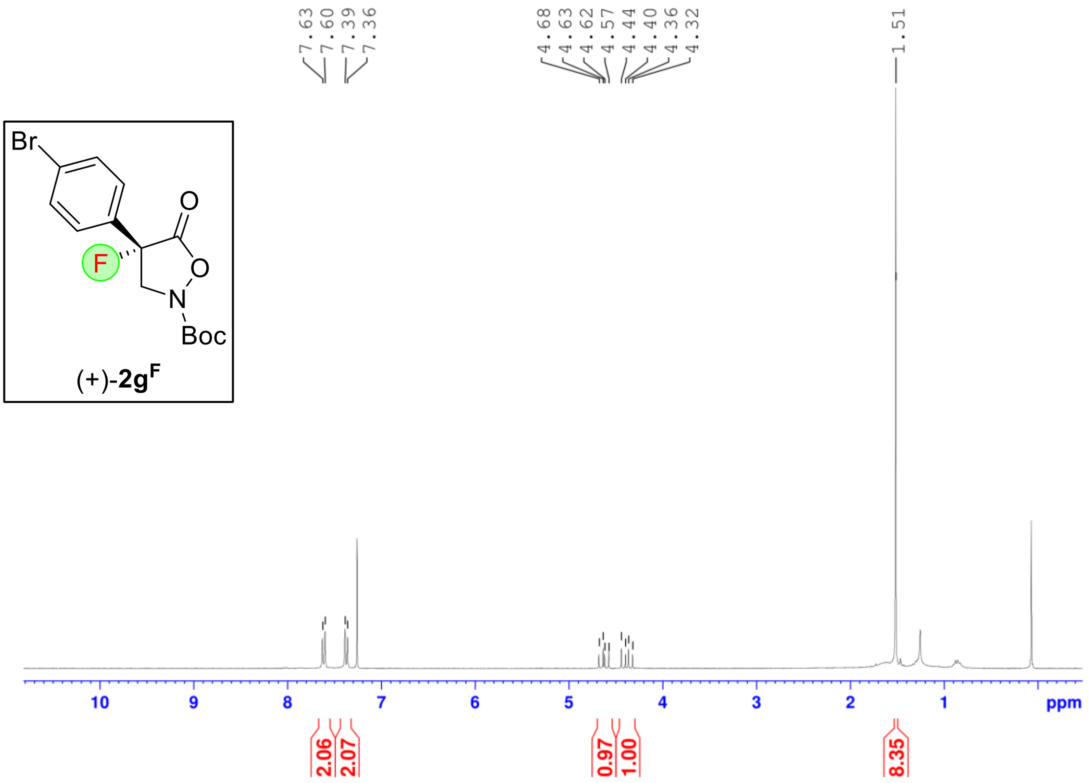
7.63
7.60
7.39
7.36
4.68
4.63
4.62
4.57
4.44
4.40
4.36
4.32
1.51
Br
O
F
O
N
Boc
(+)-2gF
10
9
8
7
6
5
4
3
2
1
ppm
2.06
2.07
0.97
1.00
8.35

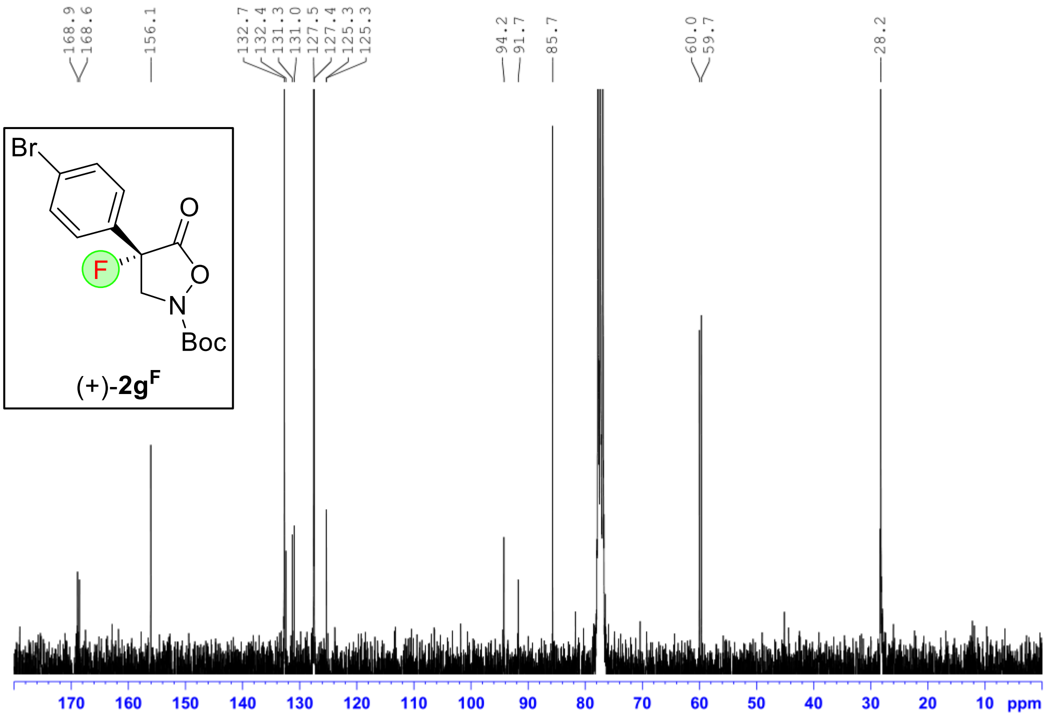

168.9
168.6
156.1
132.7
132.4
131.3
131.0
127.5
127.4
125.3
125.3
94.2
91.7
85.7
60.0
59.7
28.2
Br
O
F
O
N
Boc
(+)-2g^F
170 160 150 140 130 120 110 100 90 80 70 60 50 40 30 20 10 ppm

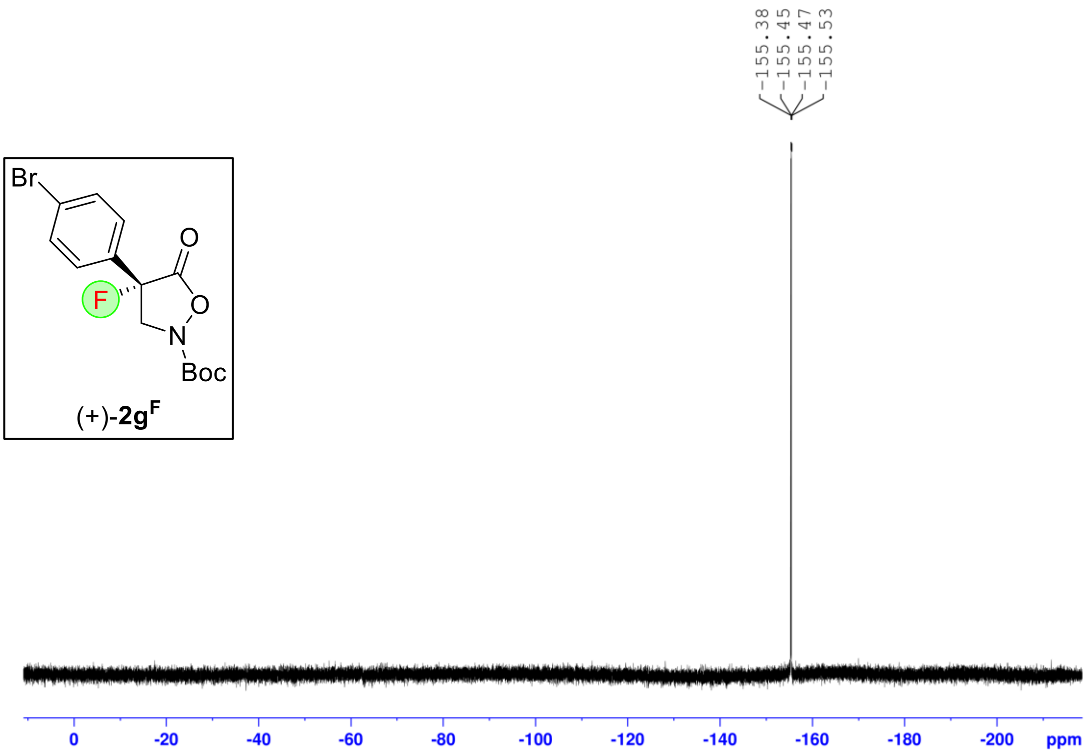

-155.38
-155.45
-155.47
-155.53
Br
O
F
O
N
Boc
(+)-2g^F
0 -20 -40 -60 -80 -100 -120 -140 -160 -180 -200 ppm

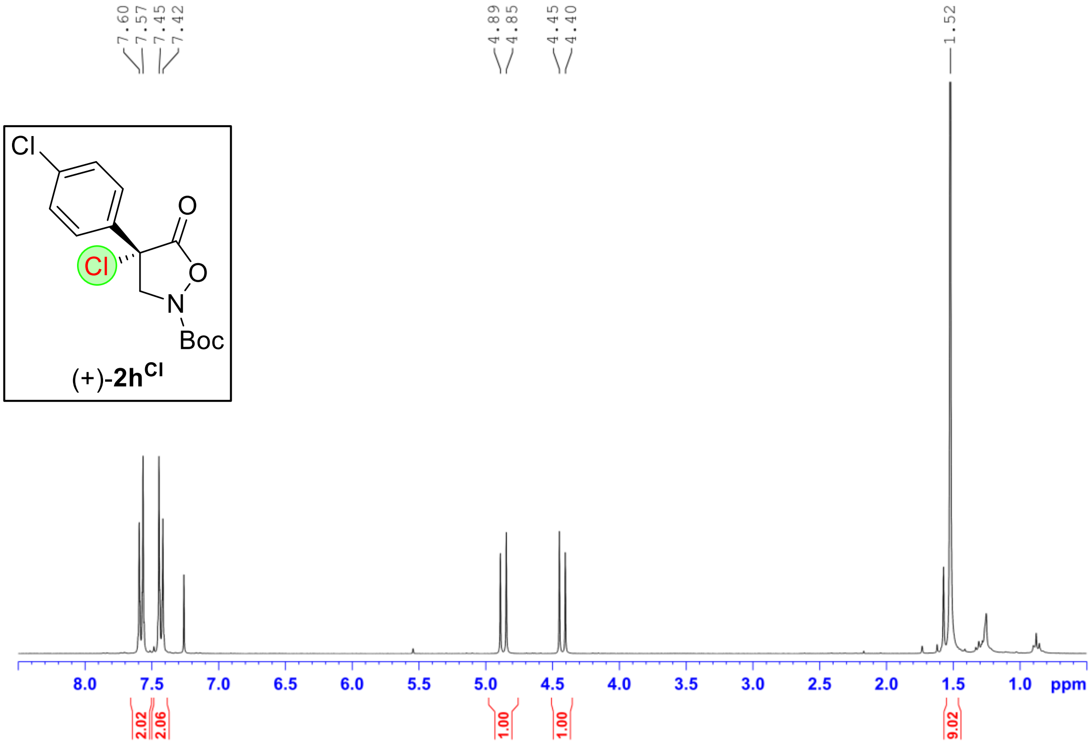
7.60
7.57
7.45
7.42
4.89
4.85
4.45
4.40
1.52
Cl
Cl
O
O
N
Boc
(+)-2h^Cl
8.0 7.5 7.0 6.5 6.0 5.5 5.0 4.5 4.0 3.5 3.0 2.5 2.0 1.5 1.0 ppm
2.02
2.06
1.00
1.00
9.02

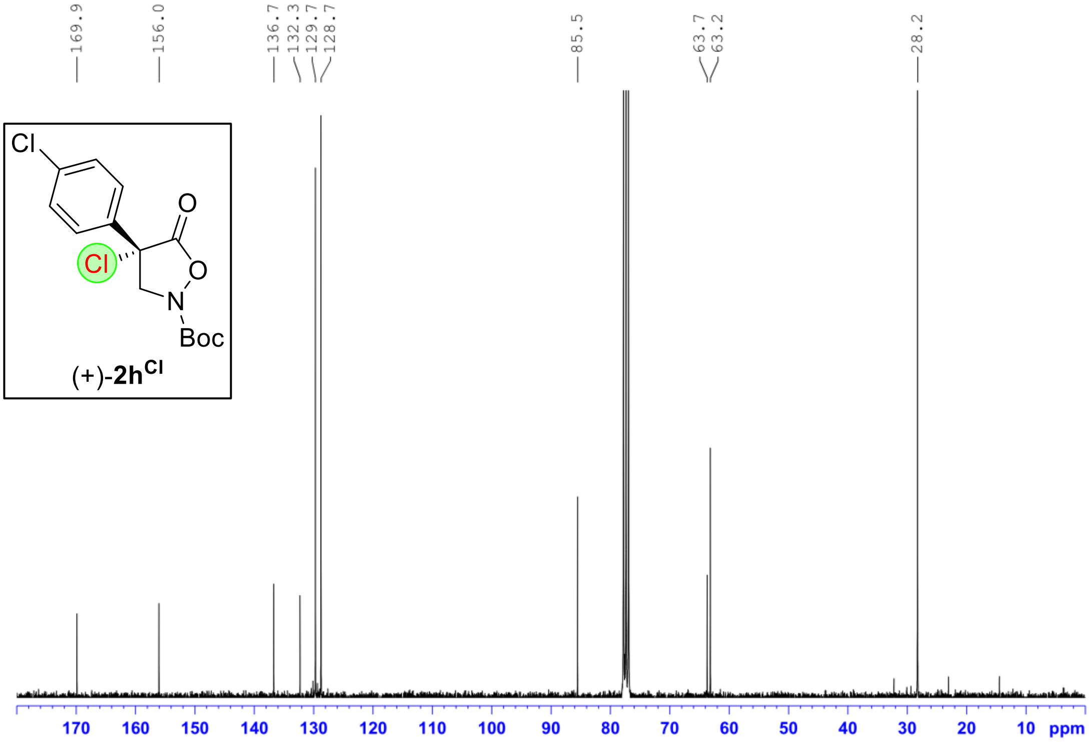
169.9
156.0
136.7
132.3
129.7
128.7
85.5
63.7
63.2
28.2
Cl
Cl
O
O
N
Boc
(+)-2h^Cl
170 160 150 140 130 120 110 100 90 80 70 60 50 40 30 20 10 ppm

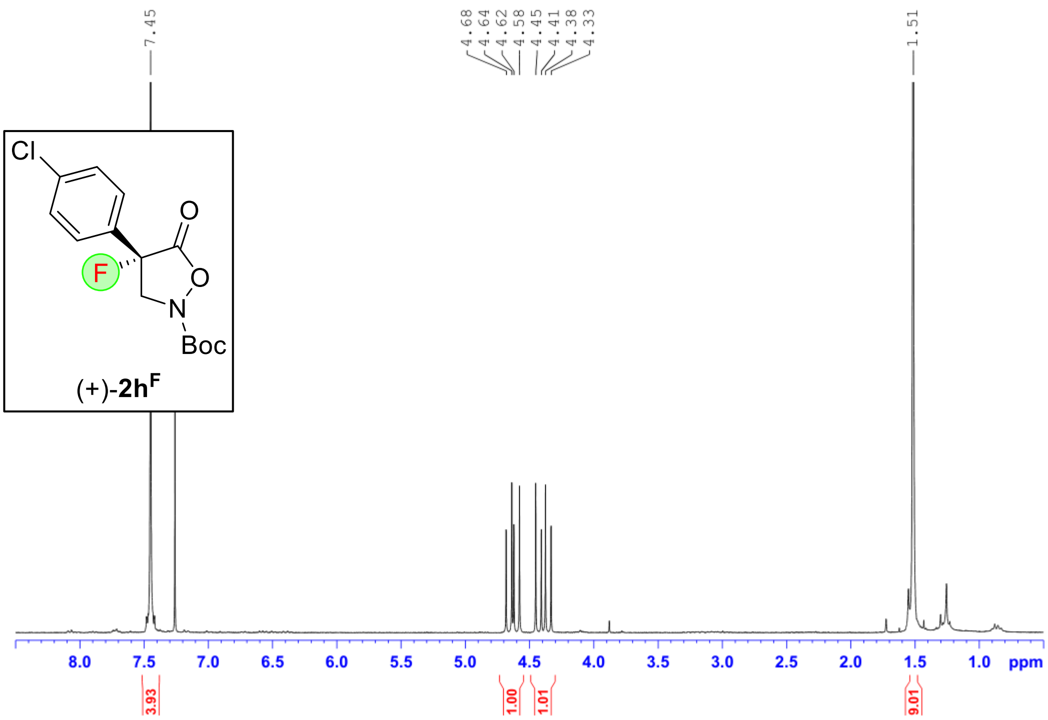
7.45
4.68
4.64
4.62
4.58
4.45
4.41
4.38
4.33
1.51
Cl
O
F
O
N
Boc
(+)-2h^{F}
8.0
7.5
7.0
6.5
6.0
5.5
5.0
4.5
4.0
3.5
3.0
2.5
2.0
1.5
1.0
ppm
3.93
1.00
1.01
9.01

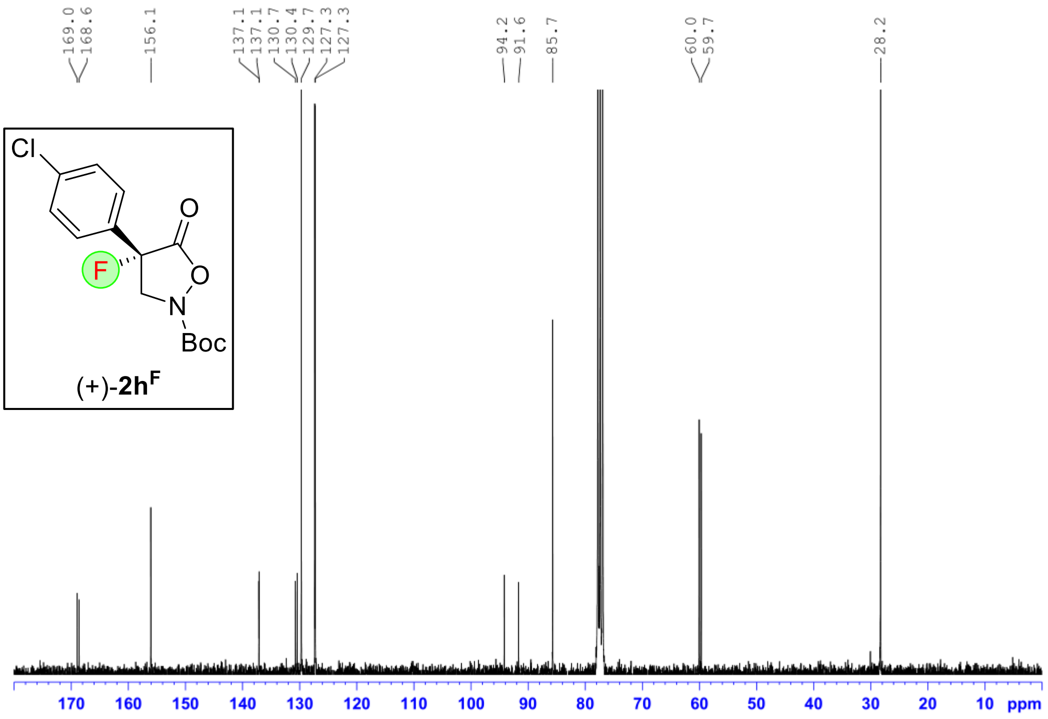
169.0
168.6
156.1
137.1
137.1
130.7
130.4
129.7
127.3
127.3
94.2
91.6
85.7
60.0
59.7
28.2
Cl
O
F
O
N
Boc
(+)-2h^{F}
170
160
150
140
130
120
110
100
90
80
70
60
50
40
30
20
10
ppm

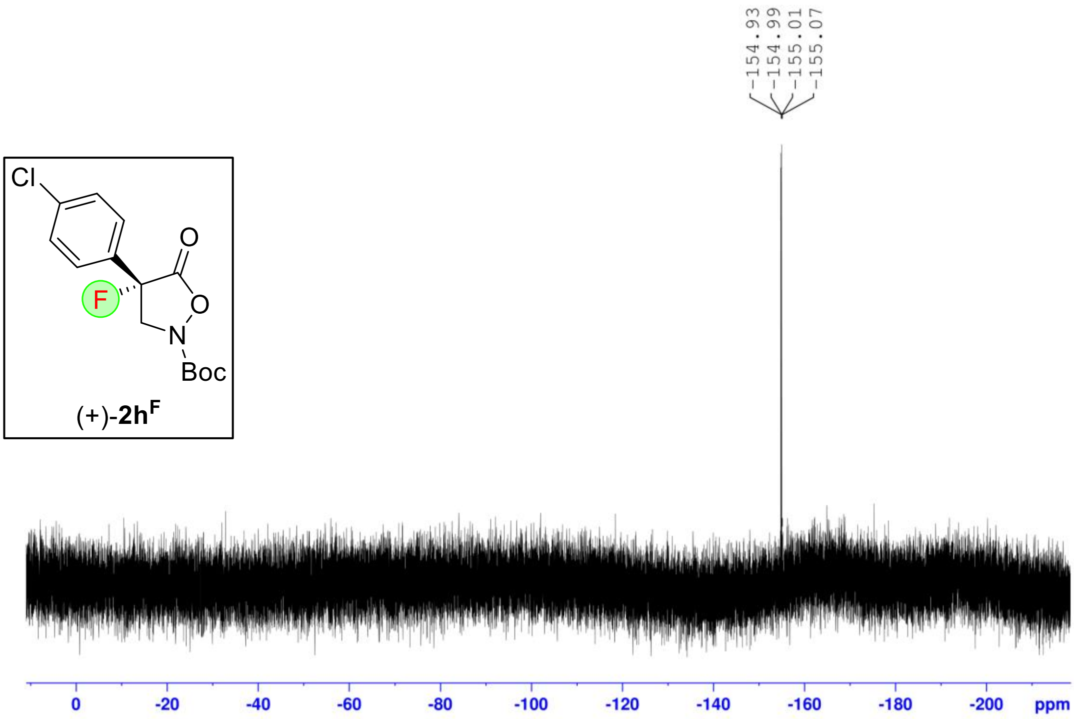

-154.93
-154.99
-155.01
-155.07
Cl
O
F
O
N
Boc
(+)-2h^{F}
0
-20
-40
-60
-80
-100
-120
-140
-160
-180
-200
ppm

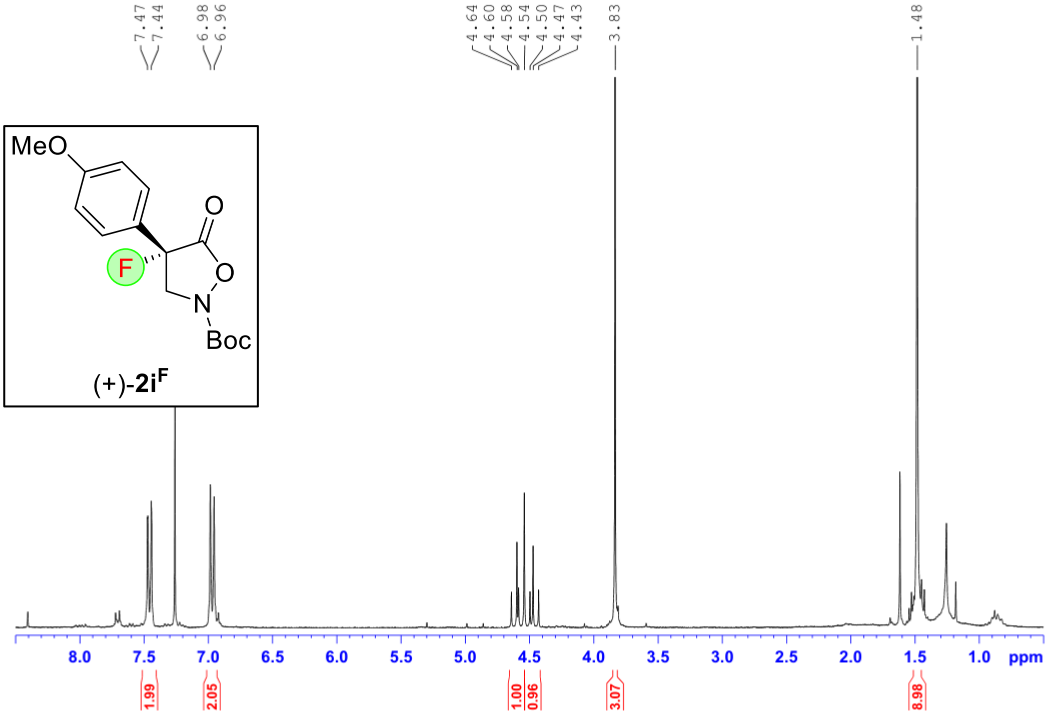

7.47
7.44
6.98
6.96
4.64
4.60
4.58
4.54
4.50
4.47
4.43
3.83
1.48
MeO
O
F
O
N
Boc
(+)-2i^{F}
8.0
7.5
7.0
6.5
6.0
5.5
5.0
4.5
4.0
3.5
3.0
2.5
2.0
1.5
1.0
ppm
1.99
2.05
1.00
0.96
3.07
8.98

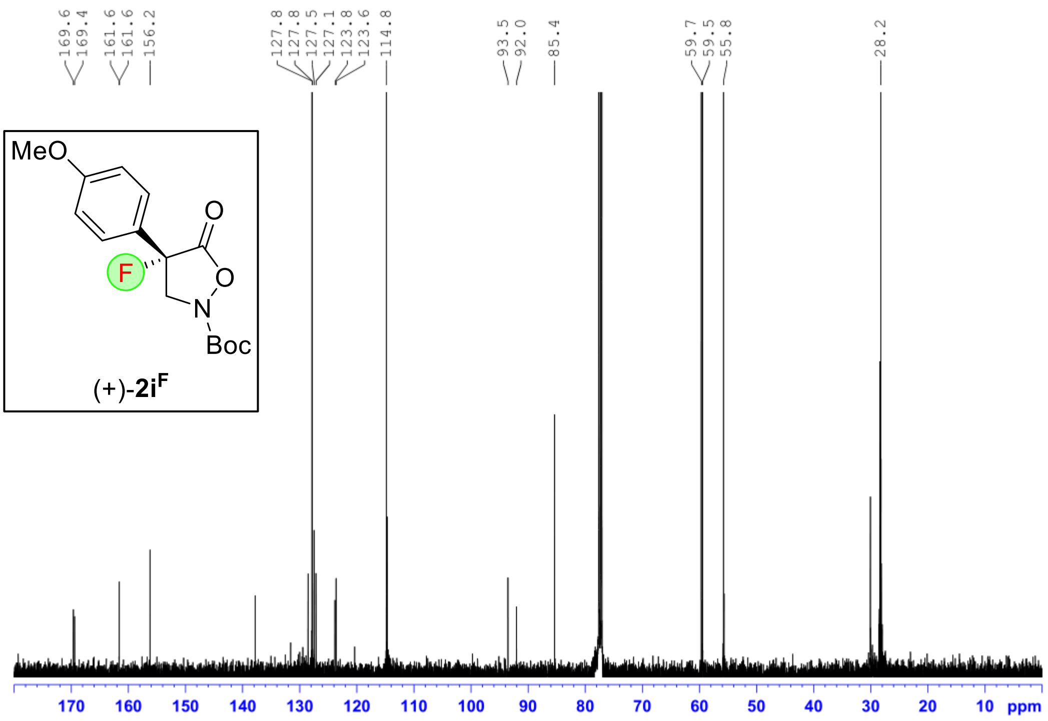

169.6
169.4
161.6
161.6
156.2
127.8
127.8
127.5
127.1
123.8
123.6
114.8
93.5
92.0
85.4
59.7
59.5
55.8
28.2
MeO
O
F
O
N
Boc
(+)-2i^F
170 160 150 140 130 120 110 100 90 80 70 60 50 40 30 20 10 ppm

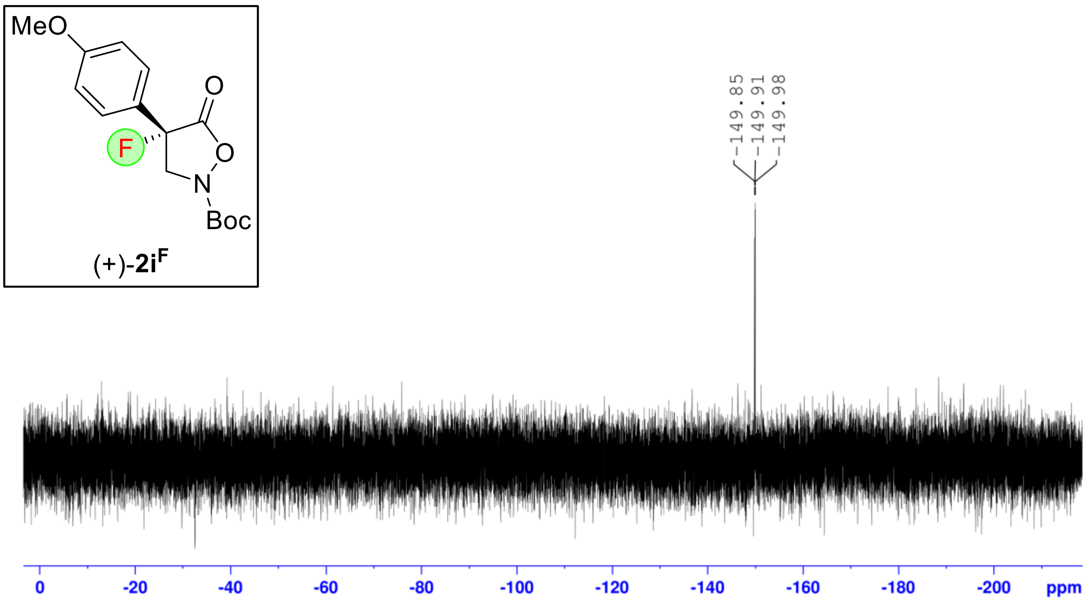

MeO
O
F
O
N
Boc
(+)-2i^F
-149.85
-149.91
-149.98
0 -20 -40 -60 -80 -100 -120 -140 -160 -180 -200 ppm

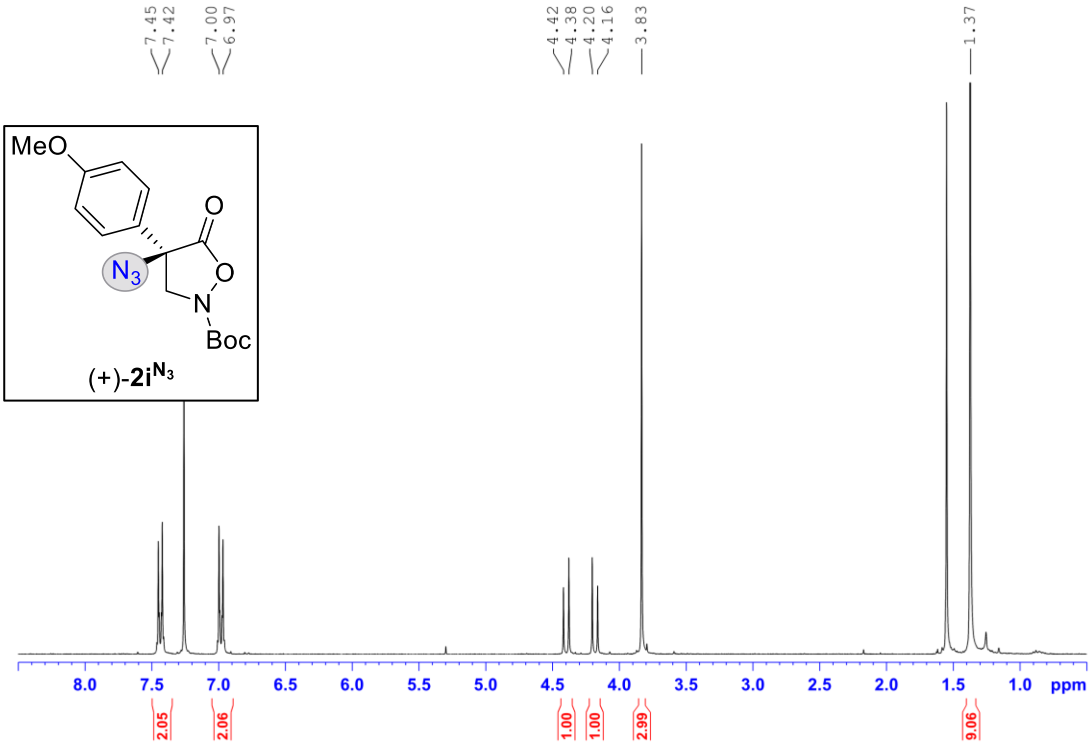
7.45
7.42
7.00
6.97
4.42
4.38
4.20
4.16
3.83
1.37
MeO
O
N3
O
N
Boc
(+)-2i^N3
8.0
7.5
7.0
6.5
6.0
5.5
5.0
4.5
4.0
3.5
3.0
2.5
2.0
1.5
1.0
ppm
2.05
2.06
1.00
1.00
2.99
9.06

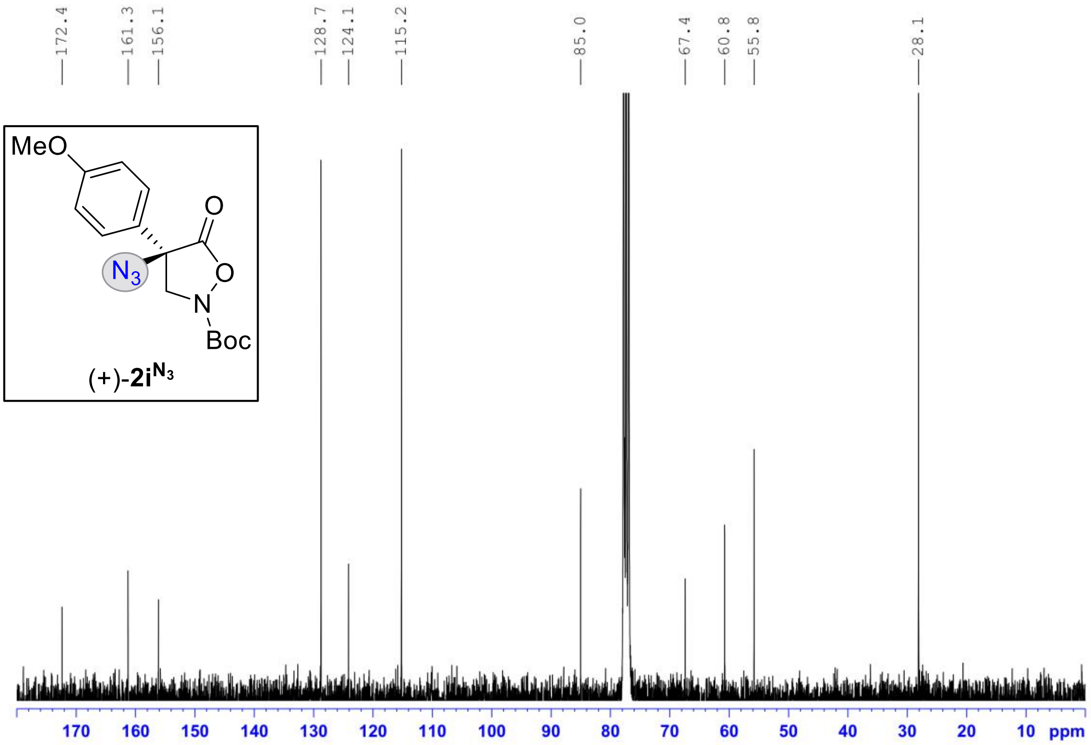
172.4
161.3
156.1
128.7
124.1
115.2
85.0
67.4
60.8
55.8
28.1
MeO
O
N3
O
N
Boc
(+)-2i^N3
170
160
150
140
130
120
110
100
90
80
70
60
50
40
30
20
10
ppm

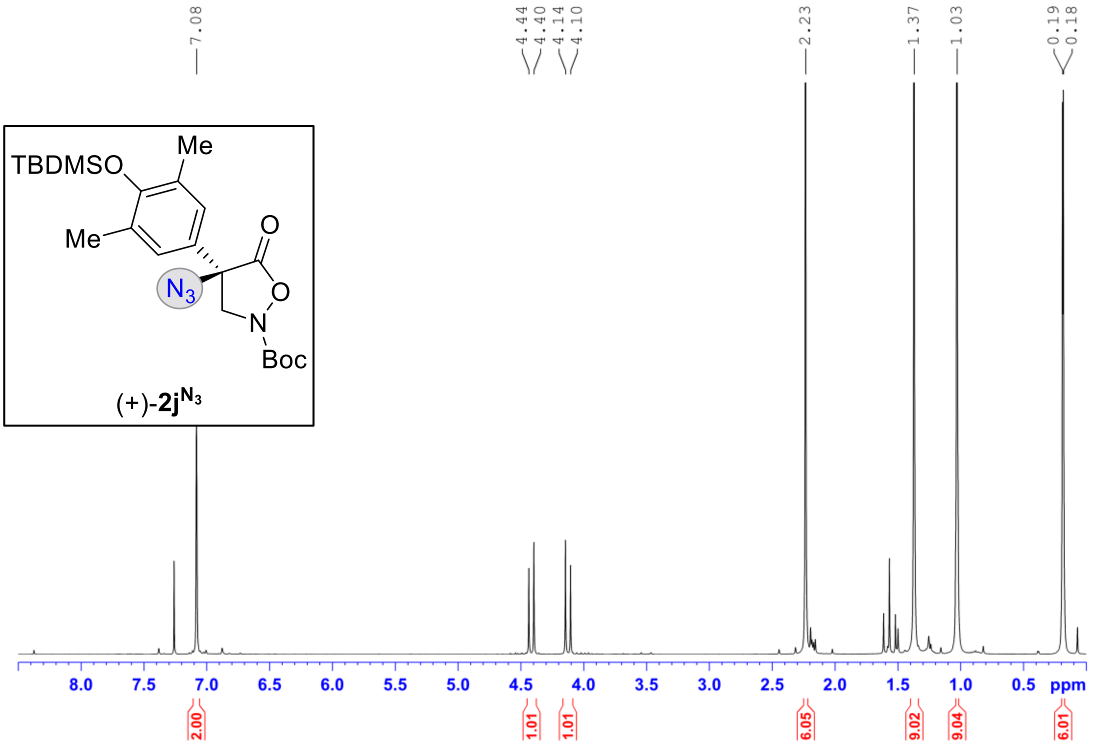
7.08
4.44
4.40
4.14
4.10
2.23
1.37
1.03
0.19
0.18
TBDMSO
Me
Me
O
N3
O
N
Boc
(+)-2j^N3
8.0
7.5
7.0
6.5
6.0
5.5
5.0
4.5
4.0
3.5
3.0
2.5
2.0
1.5
1.0
0.5
ppm
2.00
1.01
1.01
6.05
9.02
9.04
6.01

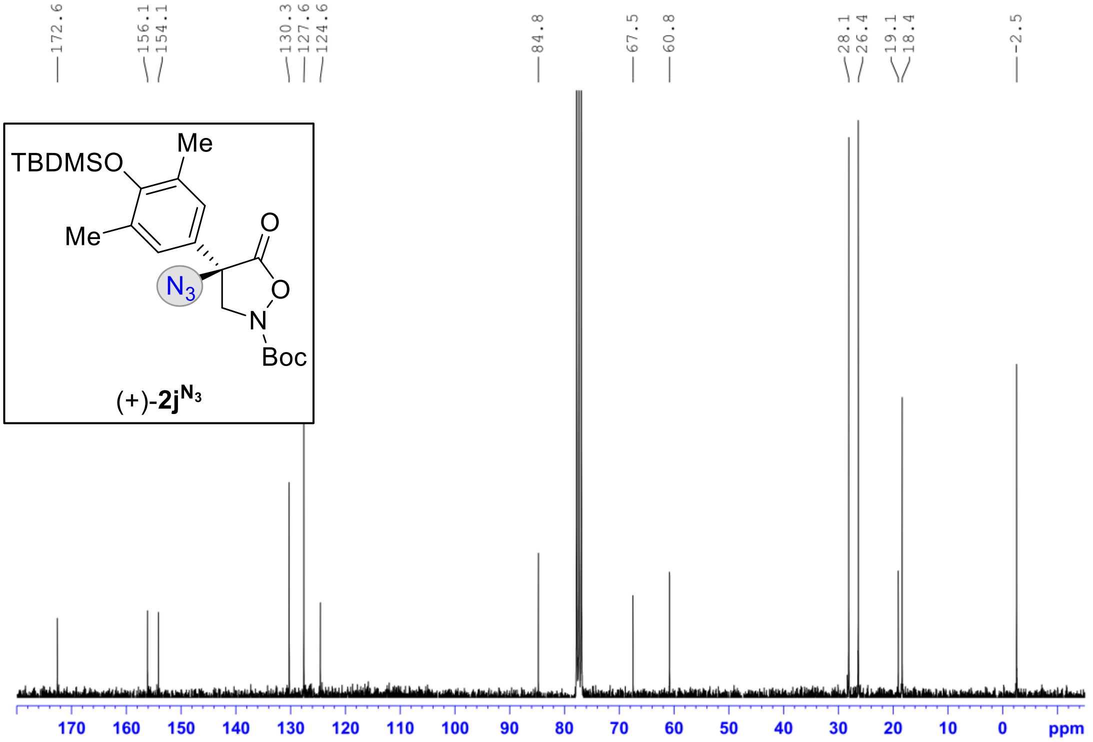
172.6
156.1
154.1
130.3
127.6
124.6
84.8
67.5
60.8
28.1
26.4
19.1
18.4
-2.5
TBDMSO
Me
Me
O
N3
O
N
Boc
(+)-2j^N3
170
160
150
140
130
120
110
100
90
80
70
60
50
40
30
20
10
0
ppm

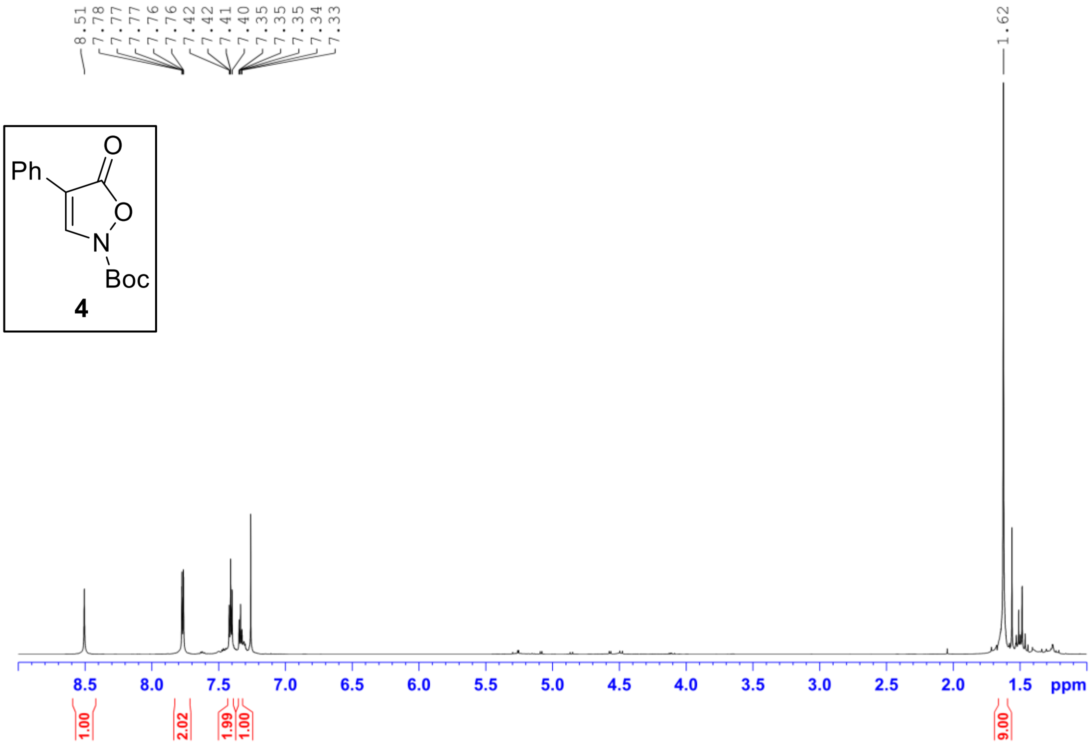
8.51
7.78
7.77
7.77
7.76
7.76
7.42
7.42
7.41
7.40
7.35
7.35
7.35
7.34
7.33
1.62
Ph
O
O
N
Boc
4
8.5
8.0
7.5
7.0
6.5
6.0
5.5
5.0
4.5
4.0
3.5
3.0
2.5
2.0
1.5
ppm
1.00
2.02
1.99
1.00
9.00

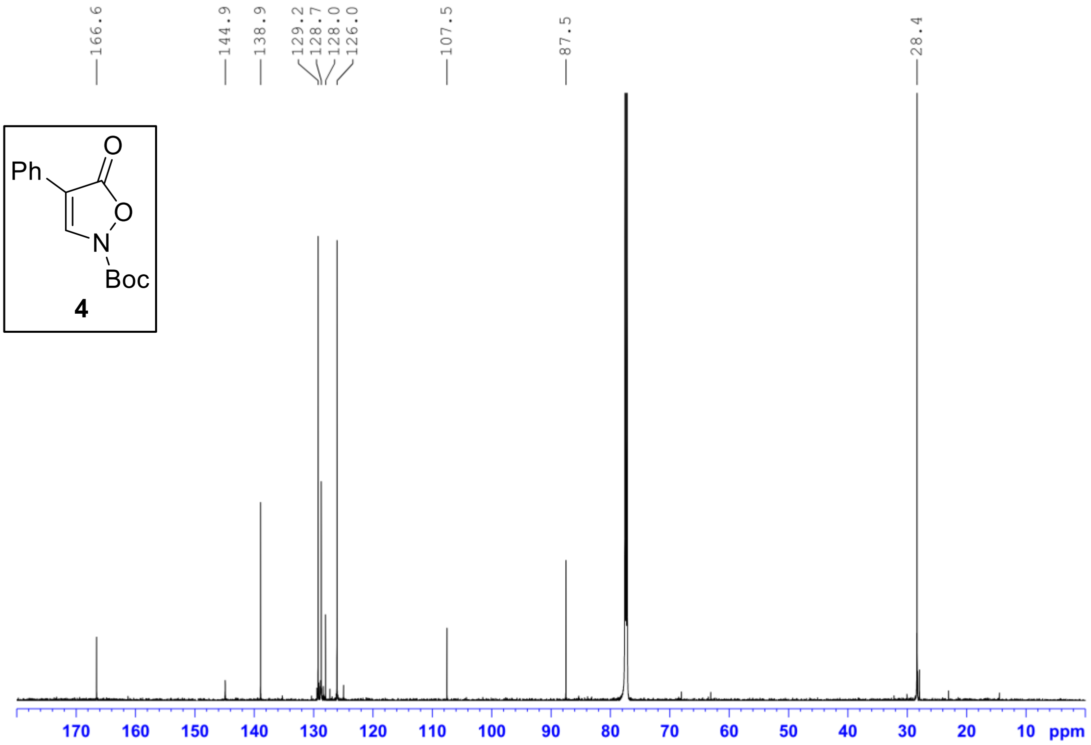
166.6
144.9
138.9
129.2
128.7
128.0
126.0
107.5
87.5
28.4
Ph
O
O
N
Boc
4
170
160
150
140
130
120
110
100
90
80
70
60
50
40
30
20
10
ppm

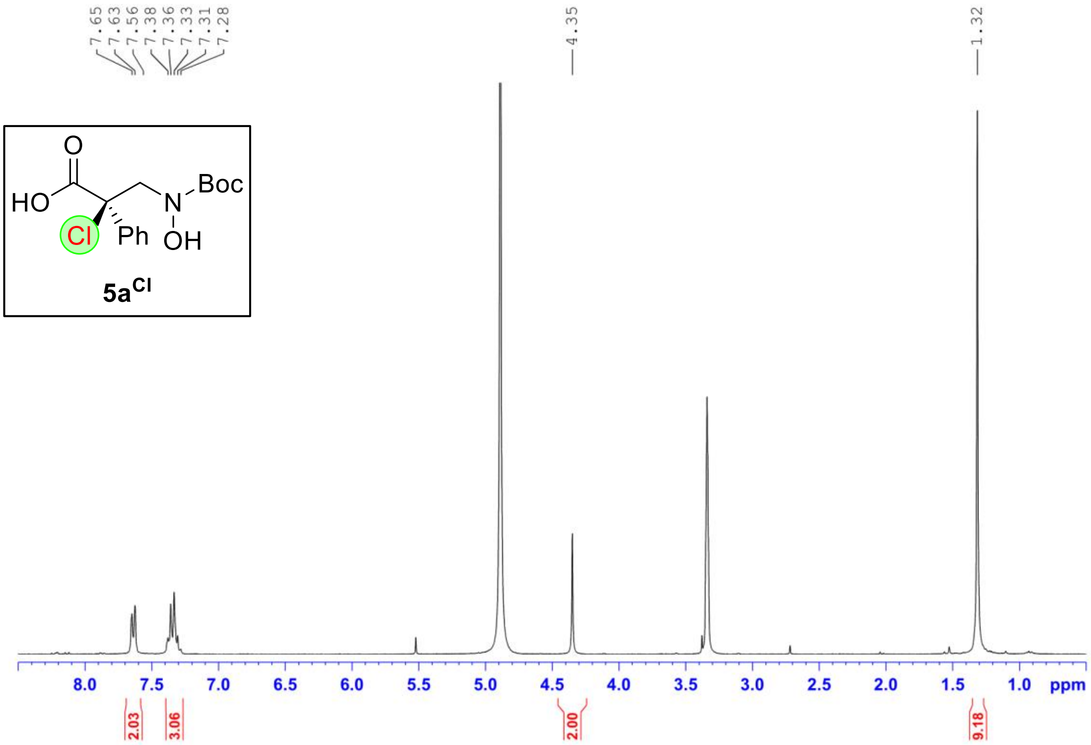
7.65
7.63
7.56
7.38
7.36
7.33
7.31
7.28
4.35
1.32
HO
O
N
Boc
Cl
Ph
OH
5a^Cl
8.0
7.5
7.0
6.5
6.0
5.5
5.0
4.5
4.0
3.5
3.0
2.5
2.0
1.5
1.0
ppm
2.03
3.06
2.00
9.18

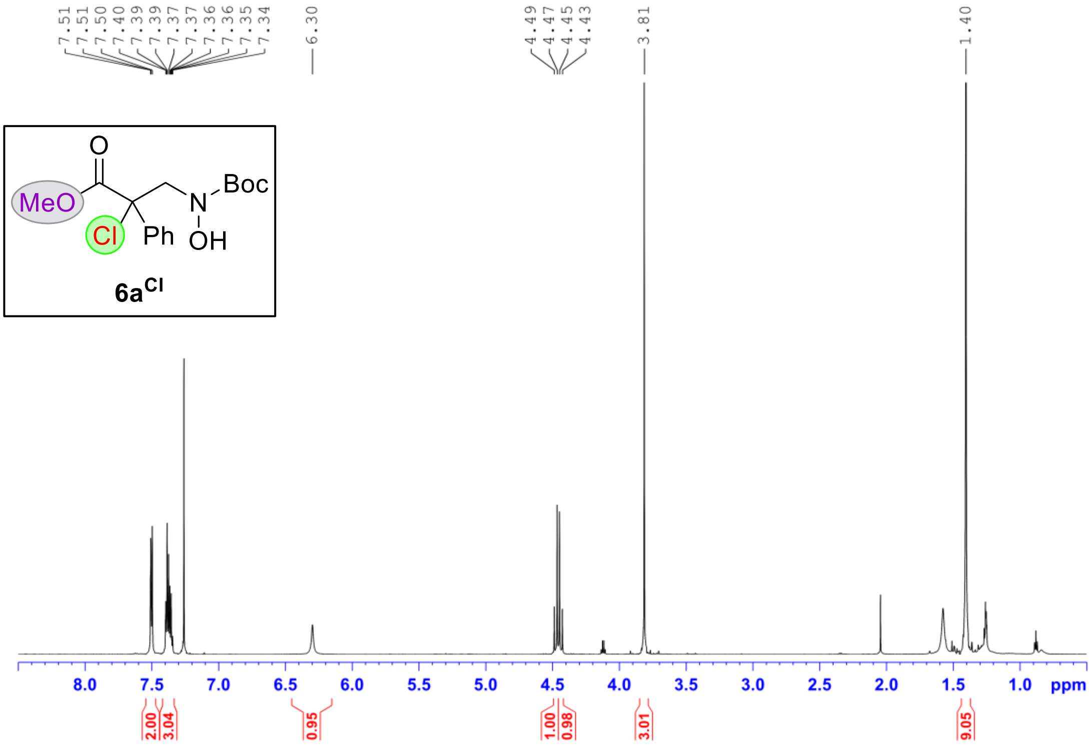
7.51
7.51
7.50
7.40
7.39
7.39
7.37
7.37
7.36
7.36
7.35
7.34
6.30
4.49
4.47
4.45
4.43
3.81
1.40
MeO
O
N
Boc
Cl
Ph
OH
6a^Cl
8.0
7.5
7.0
6.5
6.0
5.5
5.0
4.5
4.0
3.5
3.0
2.5
2.0
1.5
1.0
ppm
2.00
3.04
0.95
1.00
0.98
3.01
9.05

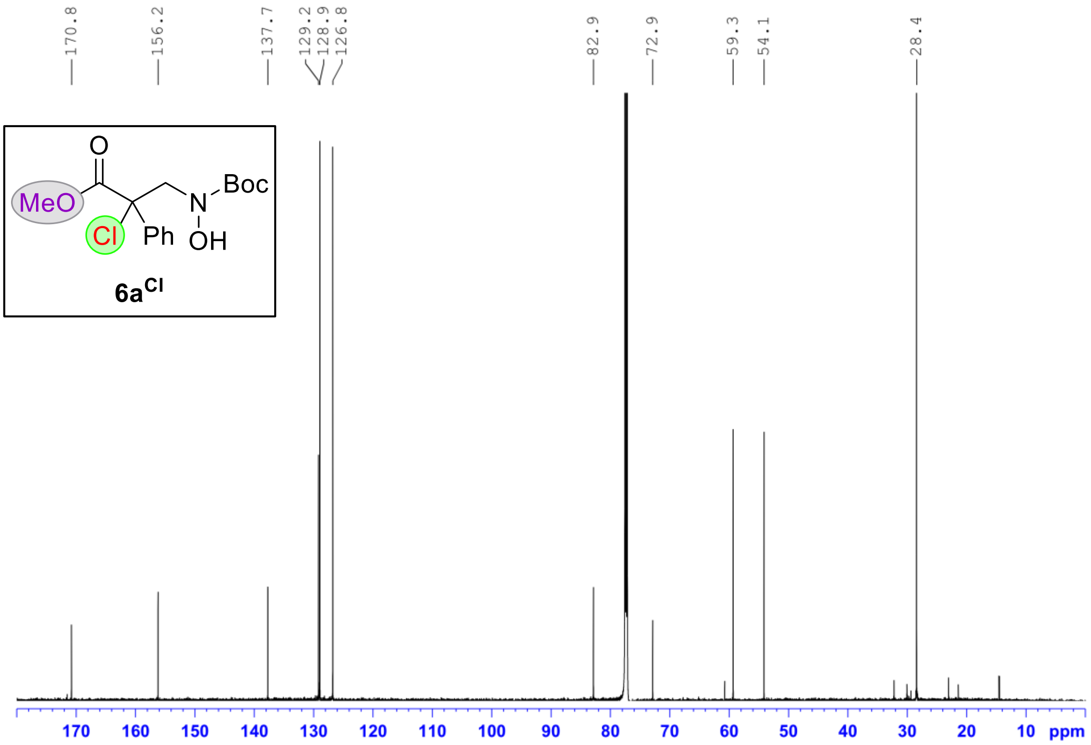
170.8
156.2
137.7
129.2
128.9
126.8
82.9
72.9
59.3
54.1
28.4
MeO
Cl
Ph
N
Boc
OH
O
6a^Cl
ppm

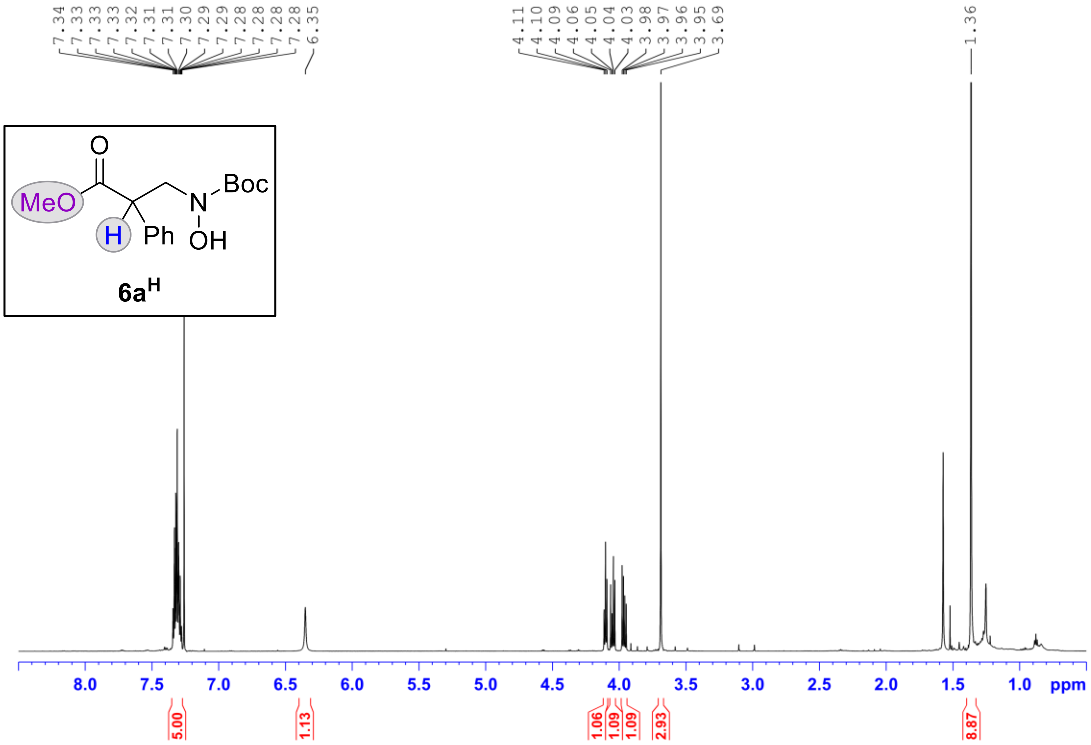
6.35
1.36
3.69
MeO
H
Ph
N
Boc
OH
O
6a^H
5.00
1.13
1.06
1.09
1.09
2.93
8.87
ppm

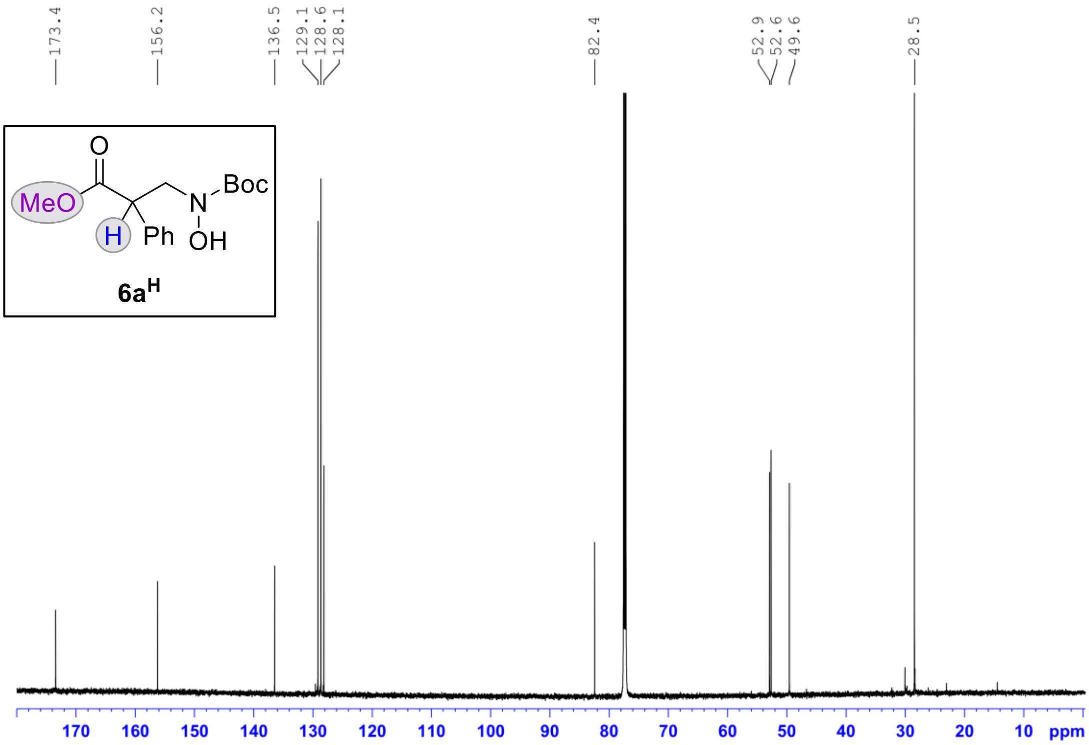

173.4
156.2
136.5
129.1
128.6
128.1
82.4
52.9
52.6
49.6
28.5
O
MeO
H
Ph
N
Boc
OH
6aH
170 160 150 140 130 120 110 100 90 80 70 60 50 40 30 20 10 ppm

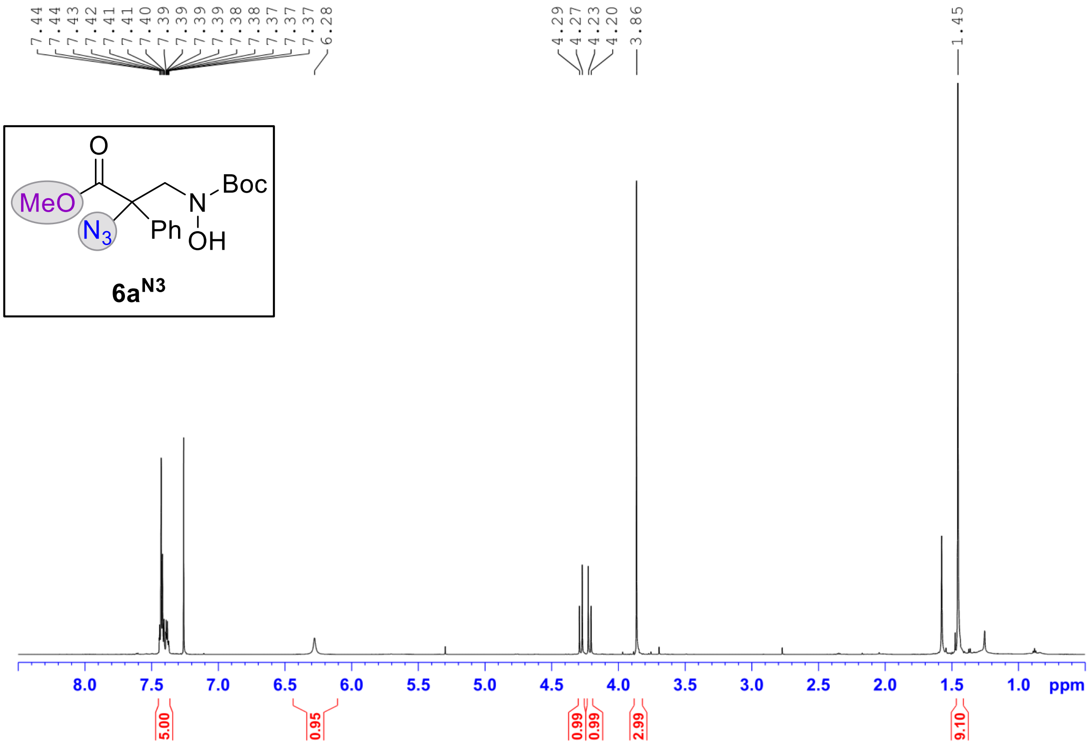

7.44
7.44
7.43
7.42
7.41
7.41
7.40
7.39
7.39
7.39
7.39
7.38
7.38
7.37
7.37
7.37
6.28
4.29
4.27
4.23
4.20
3.86
1.45
O
MeO
N3
Ph
N
Boc
OH
6aN3
8.0 7.5 7.0 6.5 6.0 5.5 5.0 4.5 4.0 3.5 3.0 2.5 2.0 1.5 1.0 ppm
5.00
0.95
0.99
0.99
2.99
9.10

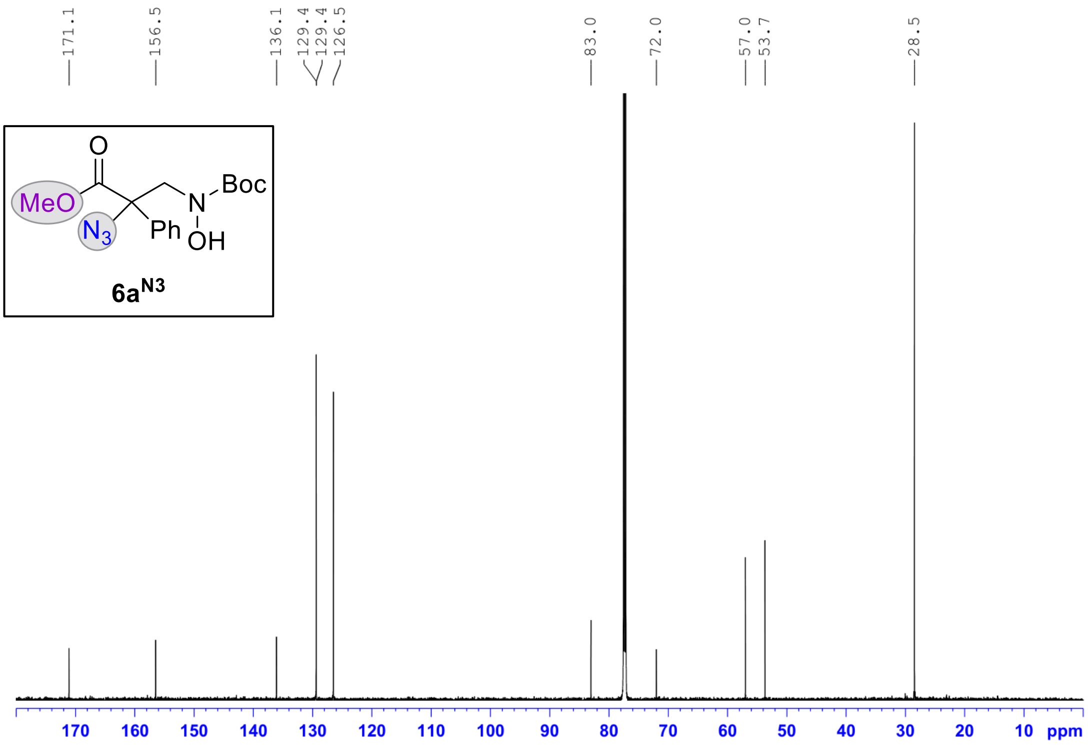

171.1
156.5
136.1
129.4
129.4
126.5
83.0
72.0
57.0
53.7
28.5
MeO
N3
Ph
N
Boc
OH
O
6a^N3
170 160 150 140 130 120 110 100 90 80 70 60 50 40 30 20 10 ppm

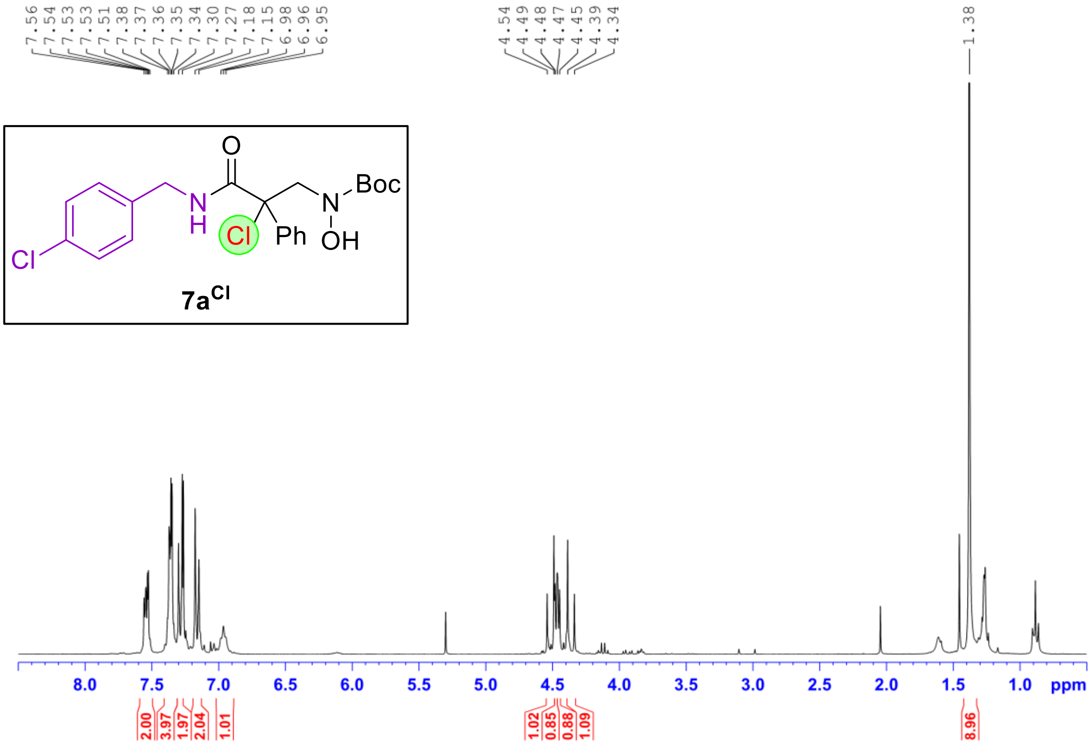

7.56 7.54 7.53 7.53 7.51 7.38 7.37 7.36 7.35 7.34 7.30 7.27 7.18 7.15 6.98 6.96 6.95
4.54 4.49 4.48 4.47 4.45 4.39 4.34
1.38
Cl
N
H
O
Cl
Ph
N
Boc
OH
7a^Cl
8.0 7.5 7.0 6.5 6.0 5.5 5.0 4.5 4.0 3.5 3.0 2.5 2.0 1.5 1.0 ppm
2.00 3.97 1.97 2.04 1.01
1.02 0.85 0.88 1.09
8.96

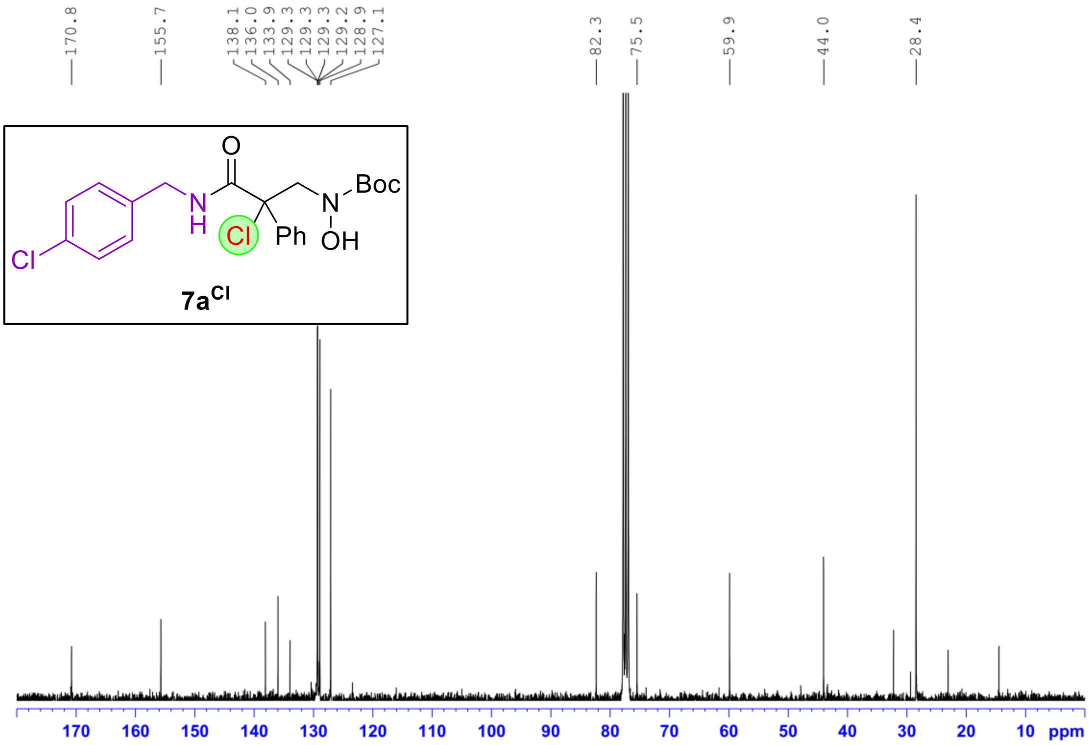
7a^Cl

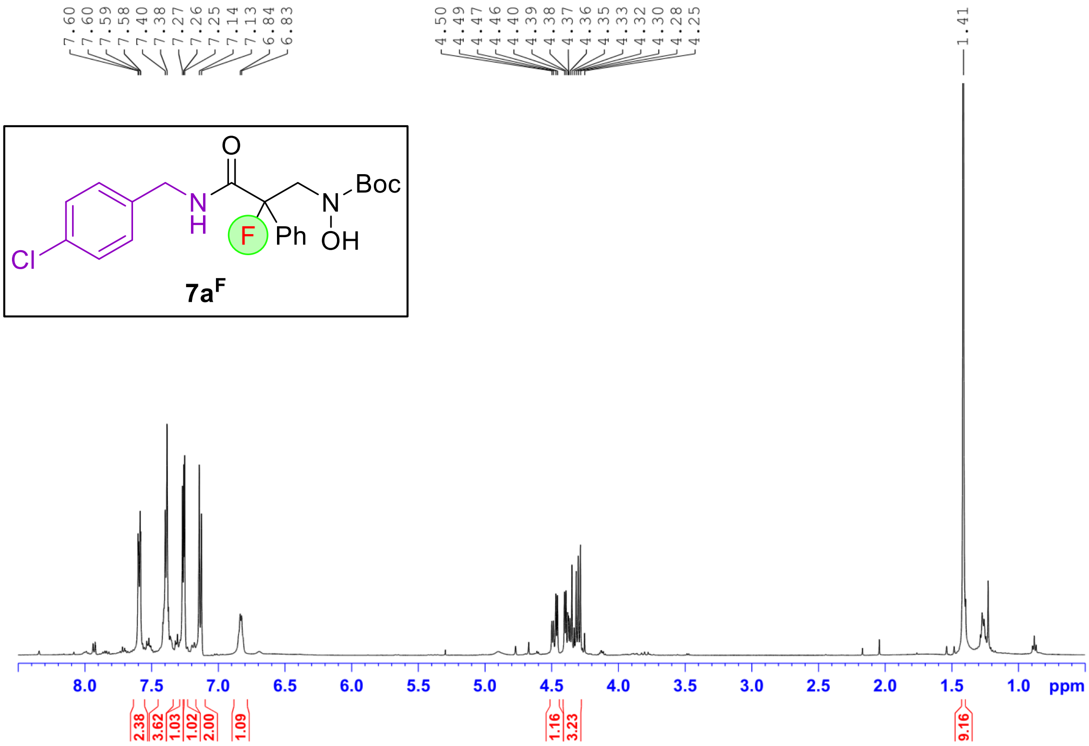
7a^F

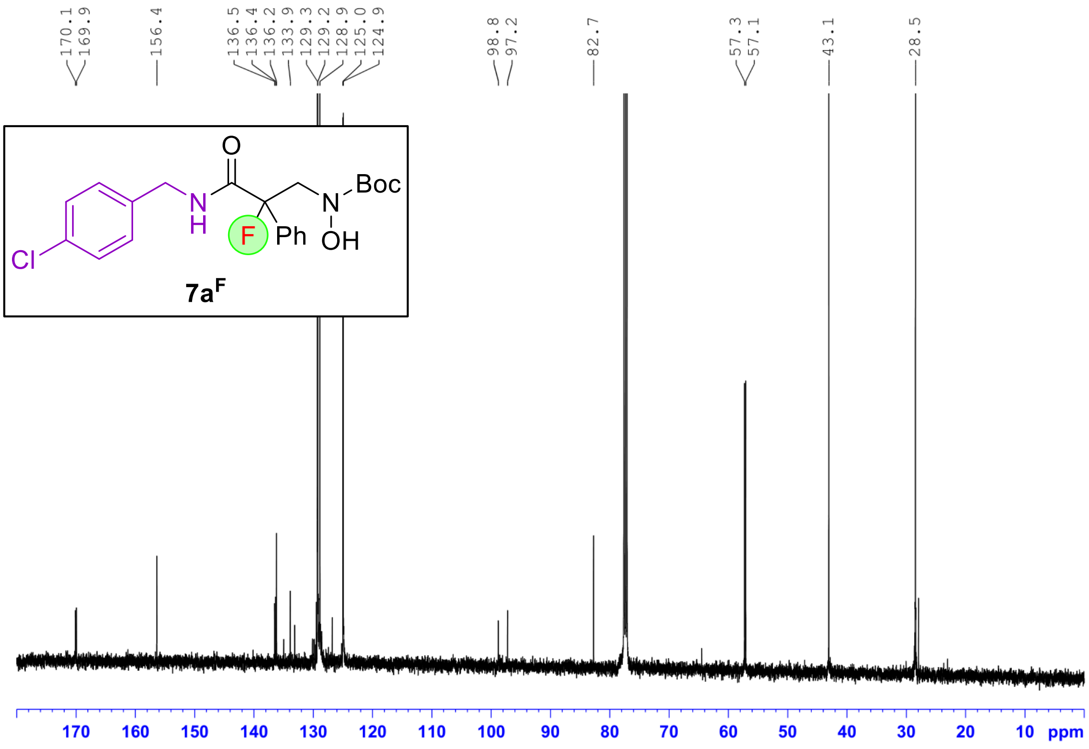
170.1
169.9
156.4
136.5
136.4
136.2
133.9
129.3
129.2
128.9
125.0
124.9
98.8
97.2
82.7
57.3
57.1
43.1
28.5
O
N
H
Boc
N
OH
F
Ph
Cl
7a^F
170
160
150
140
130
120
110
100
90
80
70
60
50
40
30
20
10
ppm

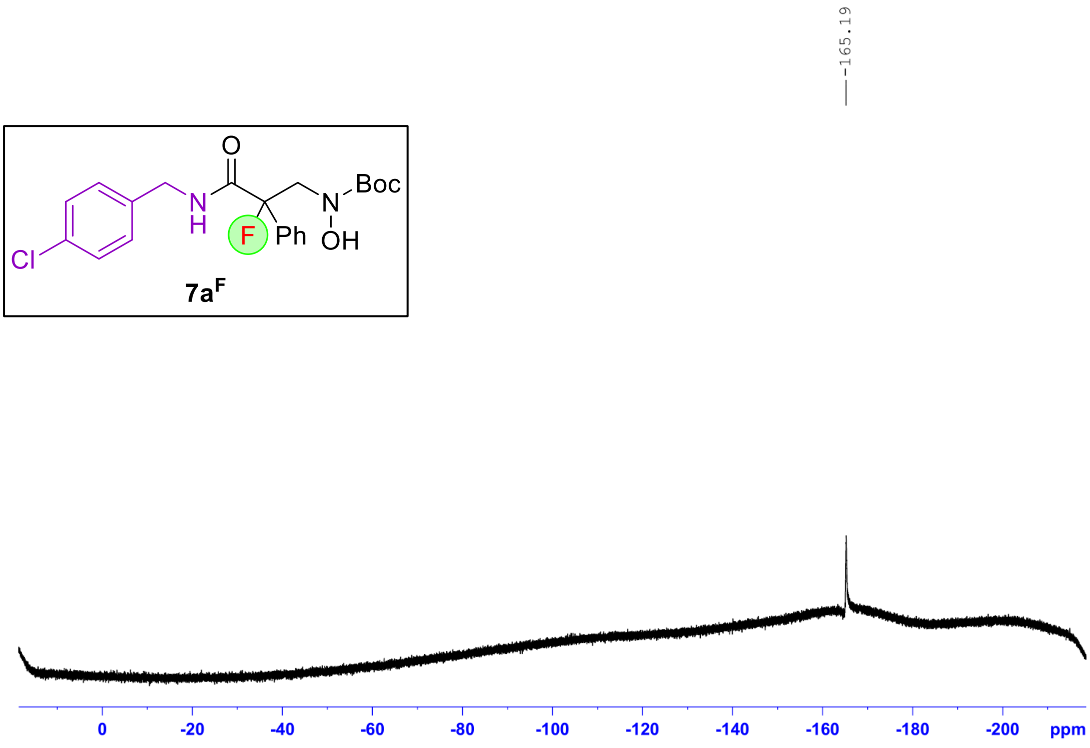
-165.19
O
N
H
Boc
N
OH
F
Ph
Cl
7a^F
0
-20
-40
-60
-80
-100
-120
-140
-160
-180
-200
ppm

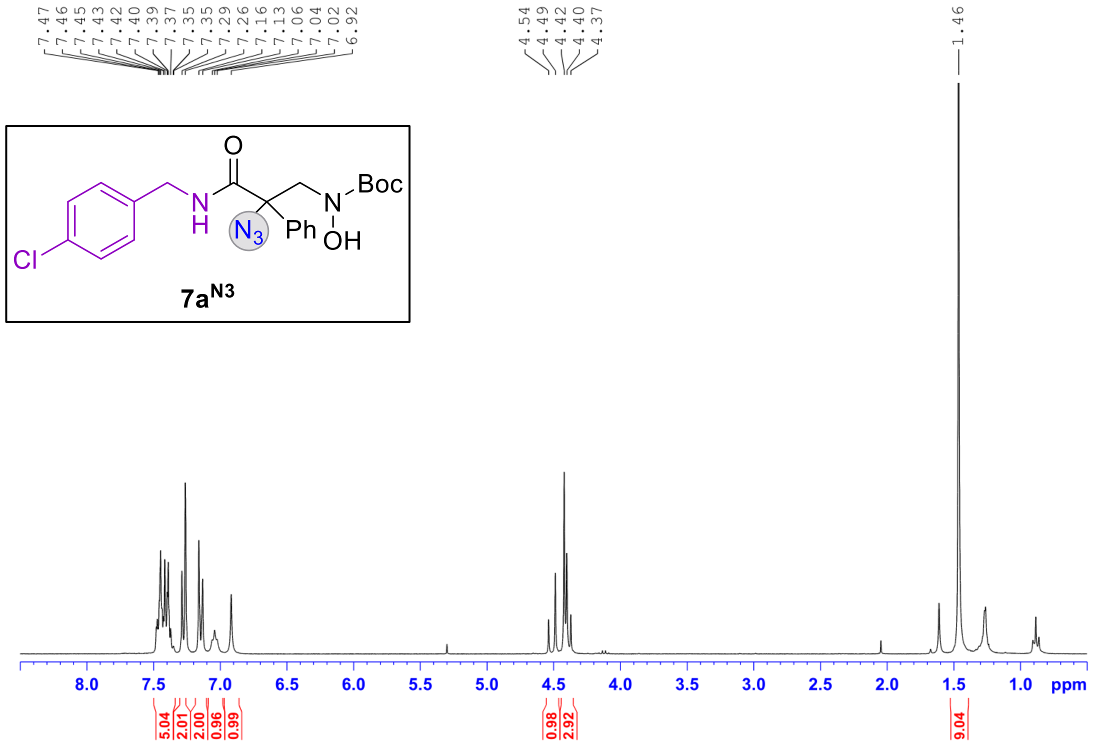
7.47
7.46
7.45
7.43
7.42
7.40
7.39
7.37
7.35
7.35
7.29
7.26
7.16
7.13
7.06
7.04
7.02
6.92
4.54
4.49
4.42
4.40
4.37
1.46
O
N
H
Boc
N
OH
N3
Ph
Cl
7a^N3
ppm
5.04
2.01
2.00
0.96
0.99
0.98
2.92
9.04

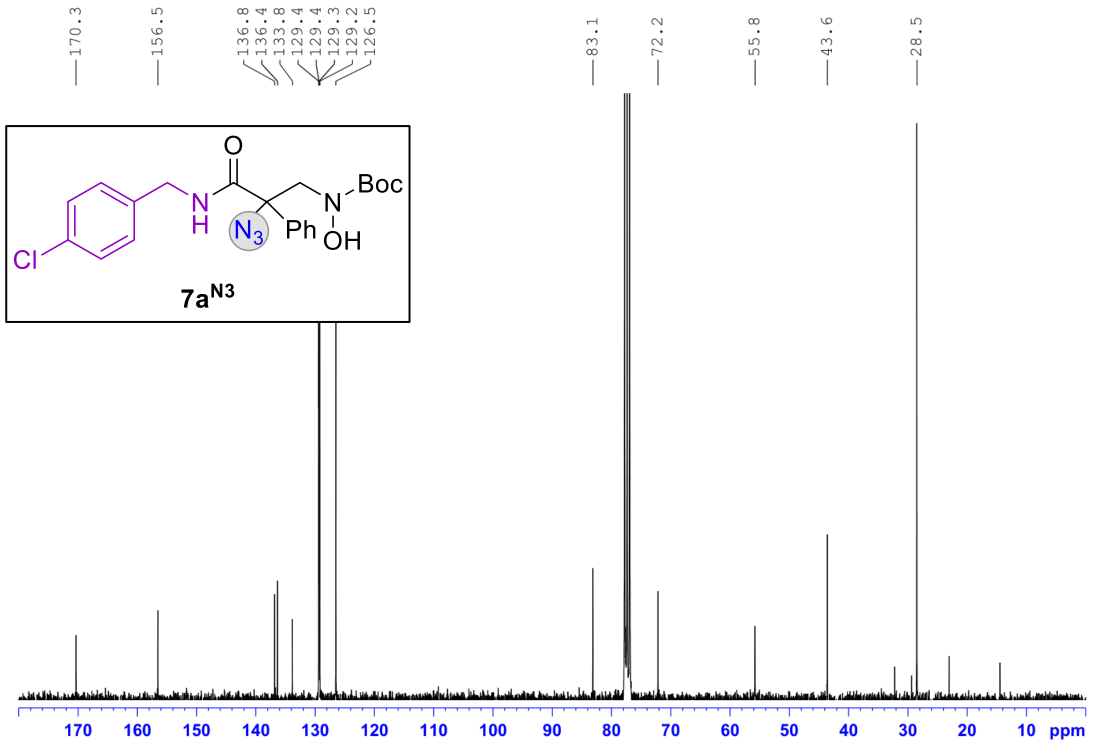
170.3
156.5
136.8
136.4
133.8
129.4
129.4
129.3
129.2
126.5
83.1
72.2
55.8
43.6
28.5
O
N
H
Boc
N
OH
N3
Ph
Cl
7a^N3
ppm

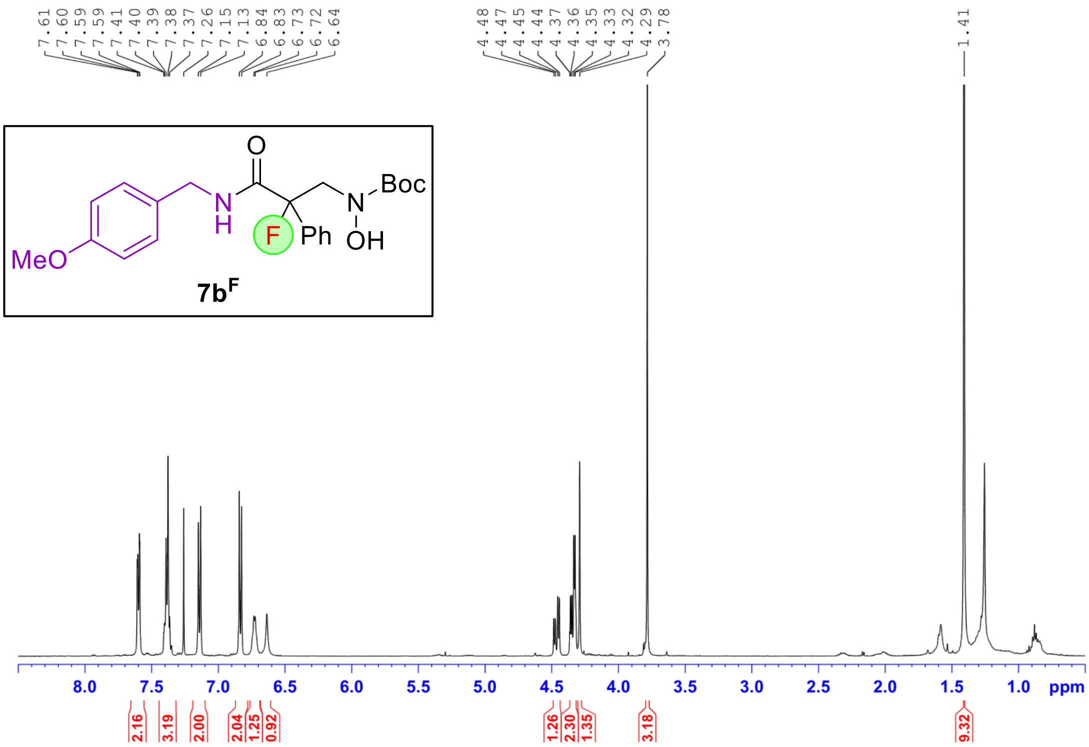
7.61
7.60
7.59
7.59
7.41
7.40
7.39
7.38
7.37
7.26
7.15
7.13
6.84
6.83
6.73
6.72
6.64
4.48
4.47
4.45
4.44
4.37
4.36
4.35
4.33
4.32
4.29
3.78
1.41
O
N
H
Boc
N
OH
F
Ph
MeO
7b[F]
8.0
7.5
7.0
6.5
6.0
5.5
5.0
4.5
4.0
3.5
3.0
2.5
2.0
1.5
1.0
ppm
2.16
3.19
2.00
2.04
1.25
0.92
1.26
2.30
1.35
3.18
9.32

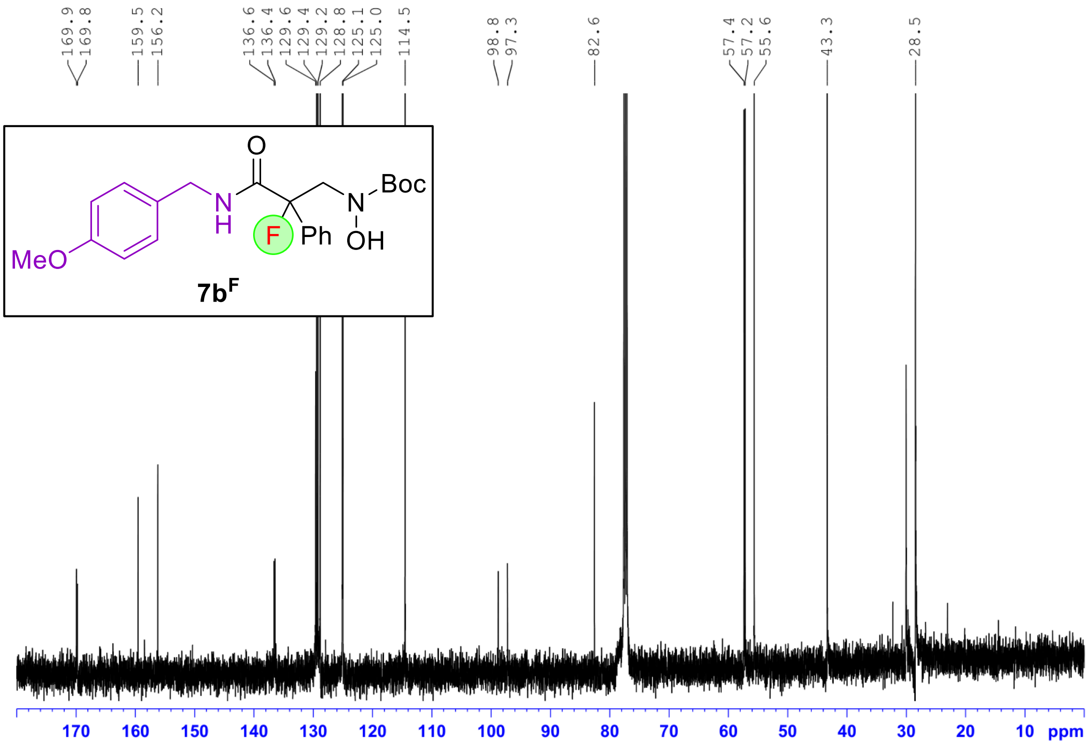
169.9
169.8
159.5
156.2
136.6
136.4
129.6
129.4
129.2
128.8
125.1
125.0
114.5
98.8
97.3
82.6
57.4
57.2
55.6
43.3
28.5
O
N
H
Boc
N
OH
F
Ph
MeO
7b[F]
170
160
150
140
130
120
110
100
90
80
70
60
50
40
30
20
10
ppm

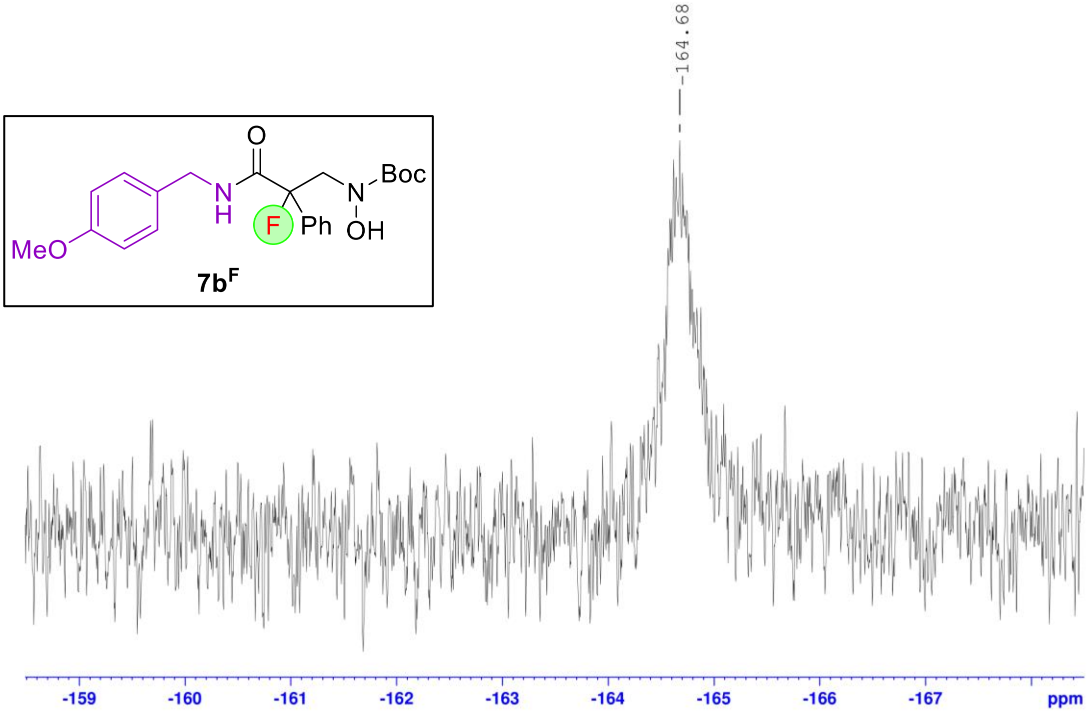

-164.68
MeO
N
H
O
F
Ph
N
Boc
OH
7b^F
-159
-160
-161
-162
-163
-164
-165
-166
-167
ppm

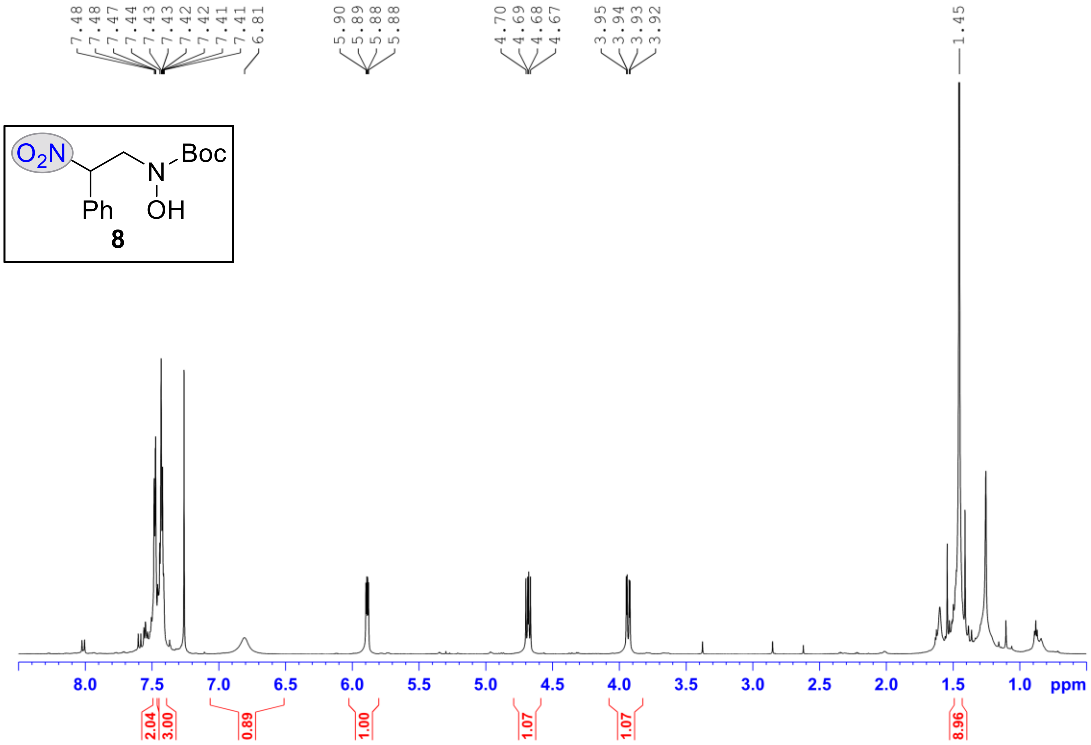

7.48
7.48
7.47
7.44
7.43
7.43
7.43
7.42
7.42
7.41
7.41
6.81
5.90
5.89
5.88
5.88
4.70
4.69
4.68
4.67
3.95
3.94
3.93
3.92
1.45
O2N
N
Boc
Ph
OH
8
8.0
7.5
7.0
6.5
6.0
5.5
5.0
4.5
4.0
3.5
3.0
2.5
2.0
1.5
1.0
ppm
2.04
3.00
0.89
1.00
1.07
1.07
8.96

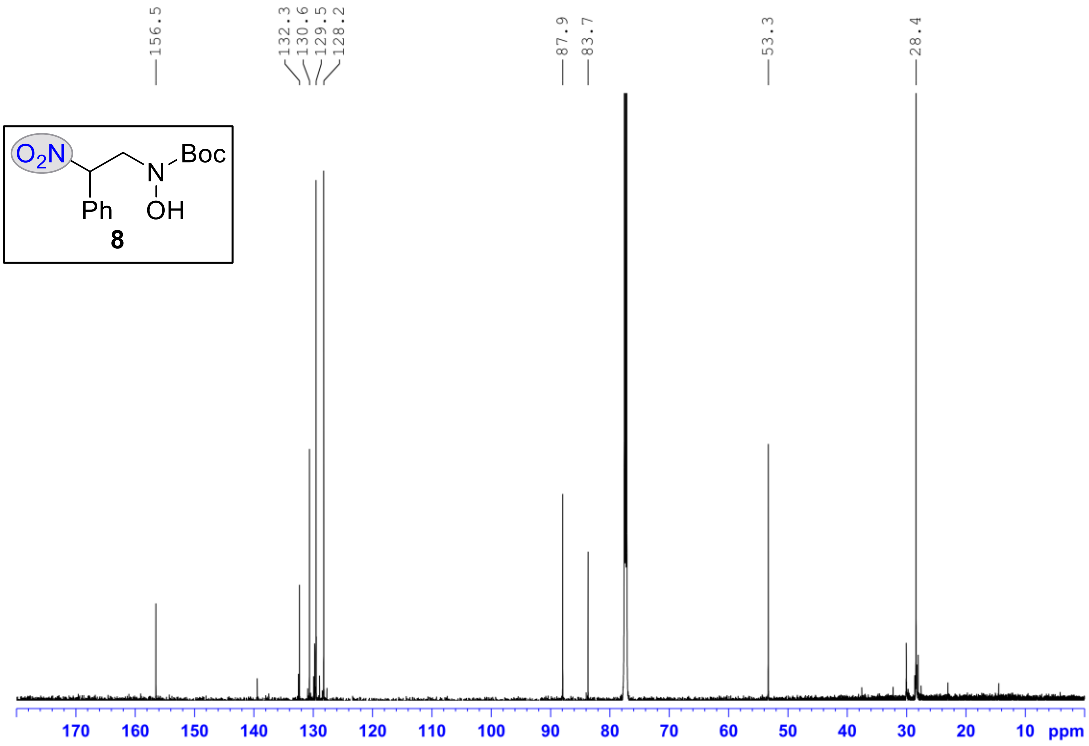
156.5
132.3
130.6
129.5
128.2
87.9
83.7
53.3
28.4
O2N
N
Boc
Ph
OH
8
170
160
150
140
130
120
110
100
90
80
70
60
50
40
30
20
10
ppm

5. Copies of HPLC Chromatograms

Chromatogram

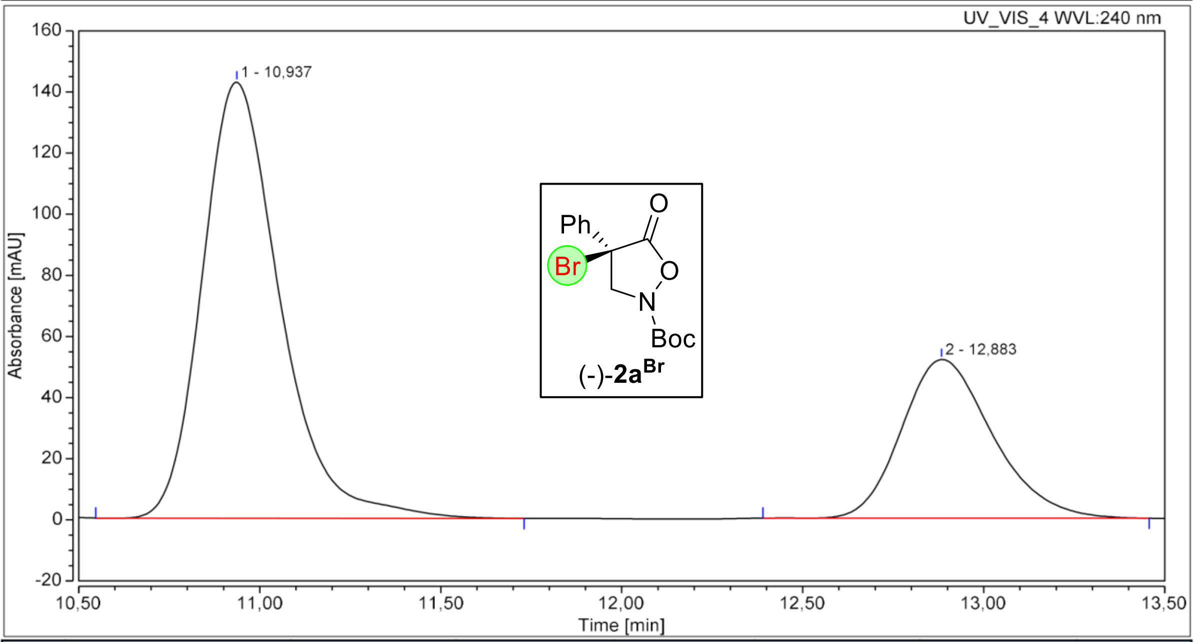

Integration Results

No.	Peak Name	Retention Time min	Area mAU*min	Height mAU	Relative Area %	Relative Height %	Amount n.a.
1		10,937	35,608	142,702	70,15	73,32	n.a.
2		12,883	15,153	51,928	29,85	26,68	n.a.
**Total:**			**50,761**	**194,630**	**100,00**	**100,00**	

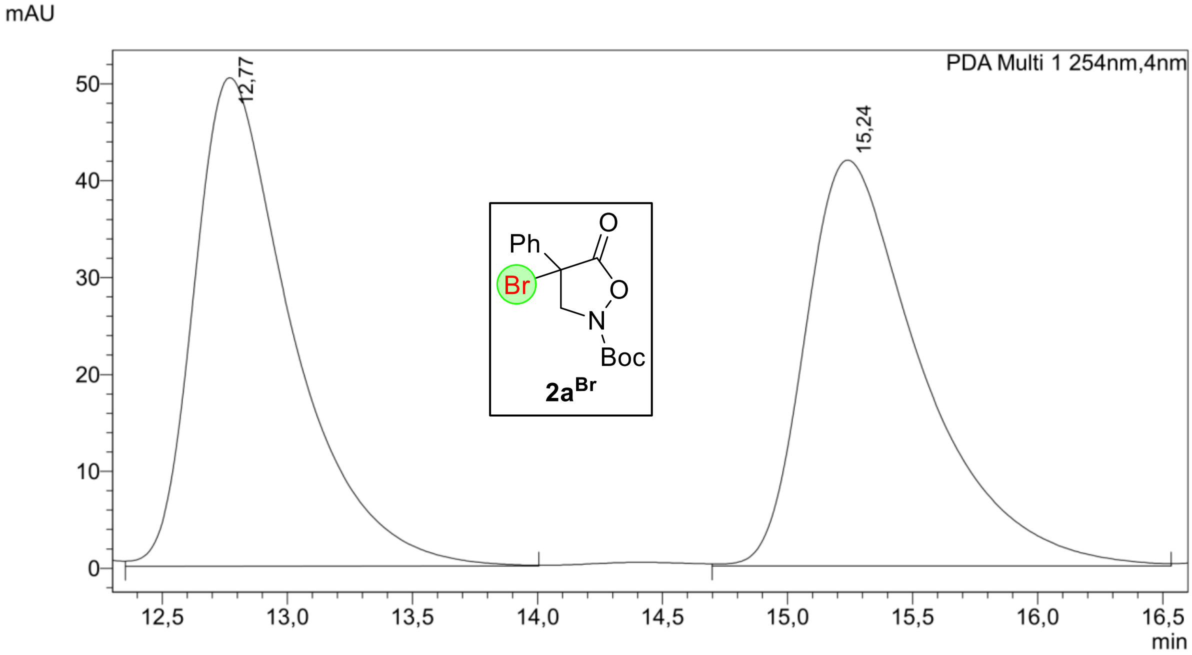

Peak Table

PDA Ch1 254nm

Peak#	Ret. Time	Area	Area%
1	12,77	1373296	50
2	15,24	1367889	50
Total		2741185	100

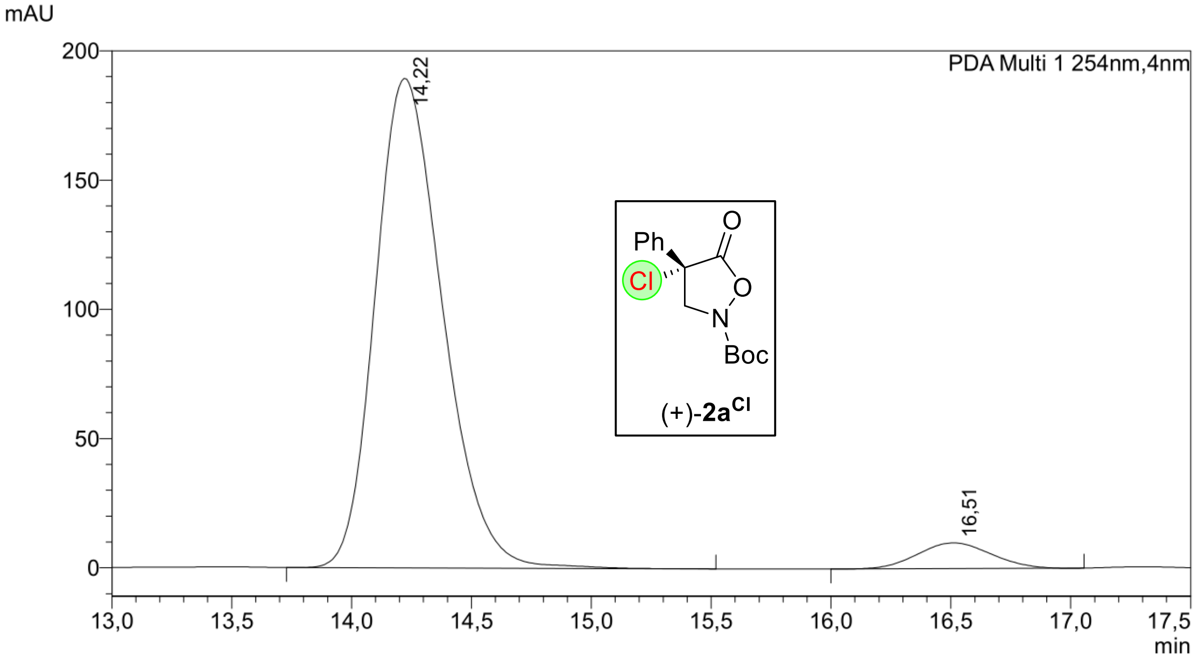

Peak Table

PDA Ch1 254nm

Peak#	Ret. Time	Area	Area%
1	14,22	3702150	95
2	16,51	213186	5
Total		3915335	100

Chromatogram

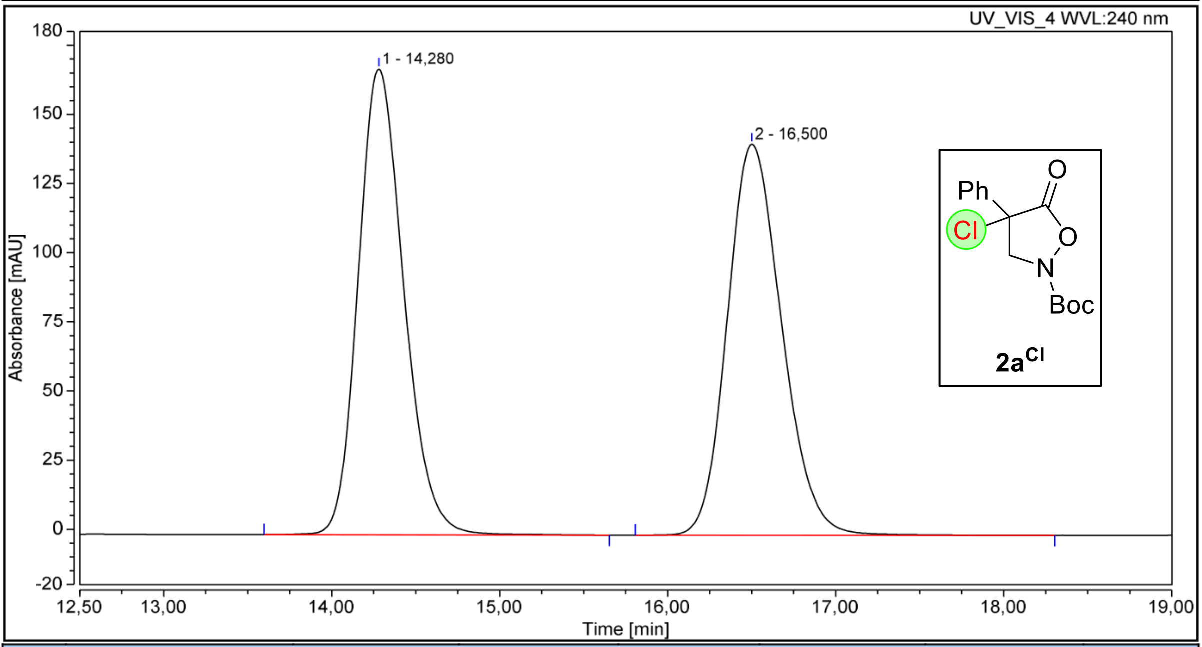

Integration Results

No.	Peak Name	Retention Time min	Area mAU*min	Height mAU	Relative Area %	Relative Height %	Amount n.a.
1		14,280	52,990	168,479	49,97	54,37	n.a.
2		16,500	53,064	141,394	50,03	45,63	n.a.
**Total:**			**106,054**	**309,873**	**100,00**	**100,00**	

Chromatogram

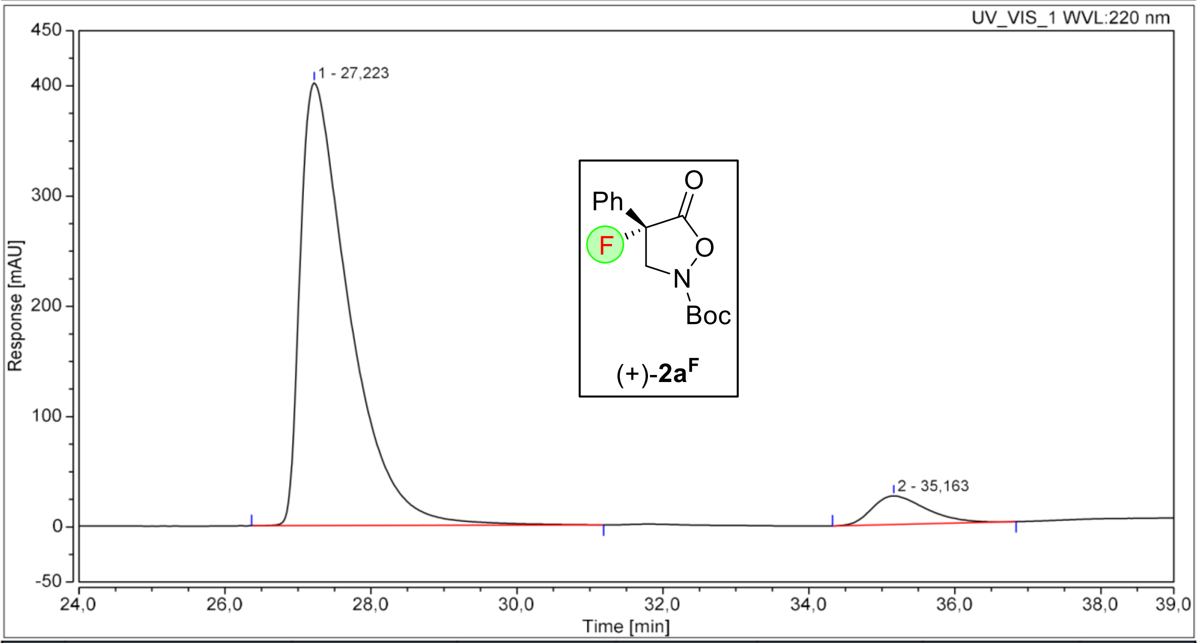

Integration Results

No.	Peak Name	Retention Time min	Area mAU*min	Height mAU	Relative Area %	Relative Height %	Amount
n.a.	Peak 1	n.a.	n.a.	n.a.	n.a.	n.a.	n.a.
1		27,223	311,527	401,414	93,34	93,94	n.a.
2		35,163	22,235	25,890	6,66	6,06	n.a.
**Total:**			**333,762**	**427,304**	**100,00**	**100,00**	

Chromatogram

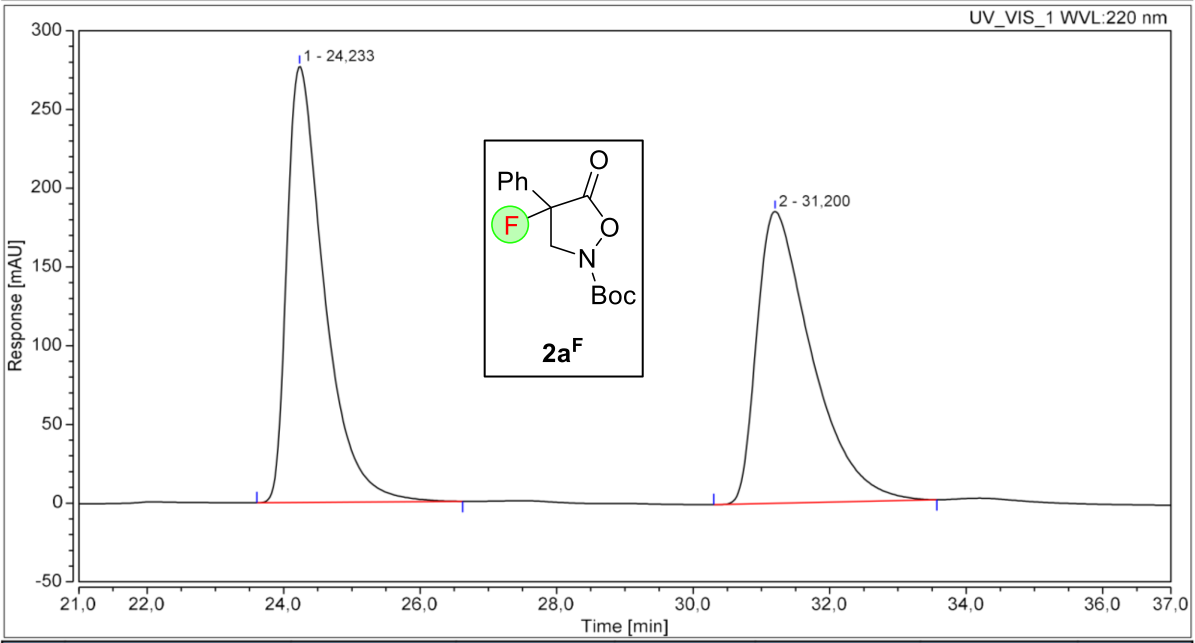

Integration Results

No.	Peak Name	Retention Time min	Area mAU*min	Height mAU	Relative Area %	Relative Height %	Amount
n.a.	Peak 1	n.a.	n.a.	n.a.	n.a.	n.a.	n.a.
1		24,233	175,379	276,957	50,34	59,91	n.a.
2		31,200	173,021	185,332	49,66	40,09	n.a.
**Total:**			**348,400**	**462,289**	**100,00**	**100,00**	

Chromatogram

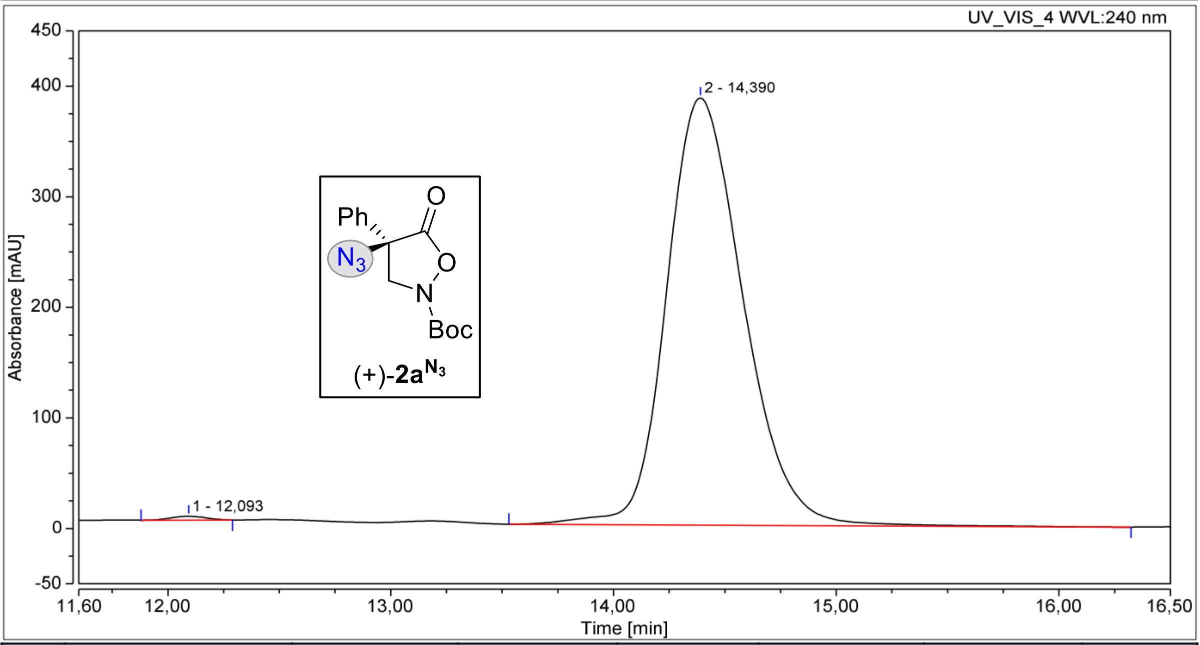

Integration Results

No.	Peak Name	Retention Time min	Area mAU*min	Height mAU	Relative Area %	Relative Height %	Amount n.a.
1		12,093	0,635	3,454	0,41	0,89	n.a.
2		14,390	154,028	386,193	99,59	99,11	n.a.
**Total:**			**154,662**	**389,647**	**100,00**	**100,00**	

Chromatogram

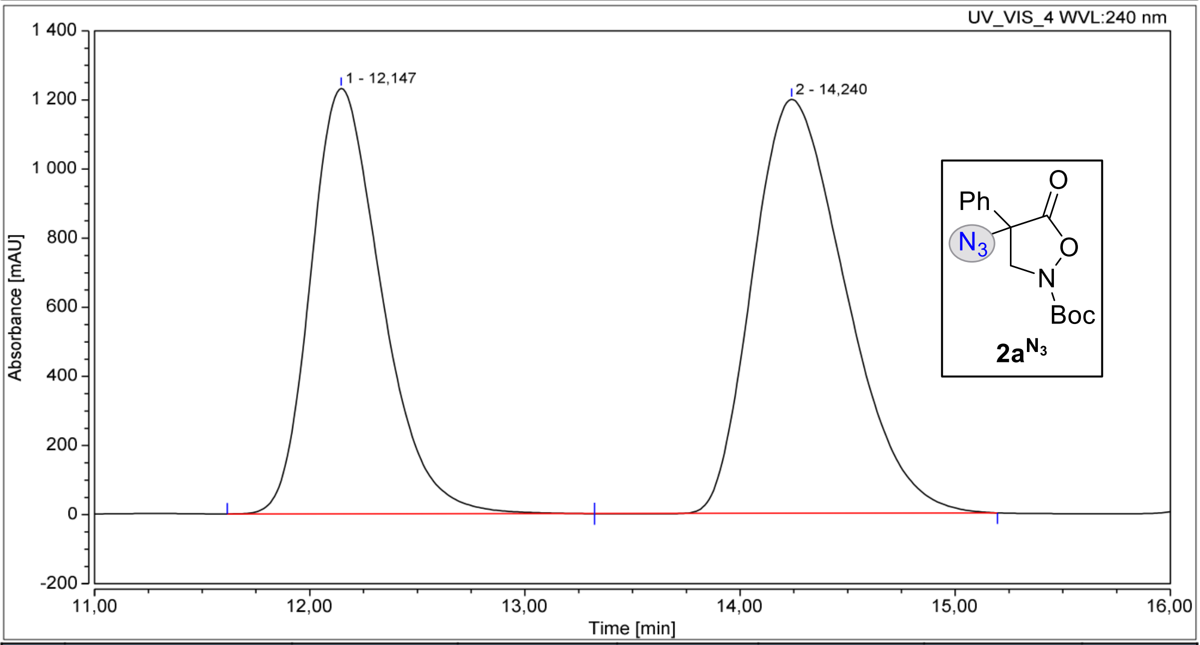

Integration Results

No.	Peak Name	Retention Time min	Area mAU*min	Height mAU	Relative Area %	Relative Height %	Amount n.a.
1		12,147	470,065	1231,140	44,02	50,69	n.a.
2		14,240	597,891	1197,396	55,98	49,31	n.a.
**Total:**			**1067,956**	**2428,536**	**100,00**	**100,00**	

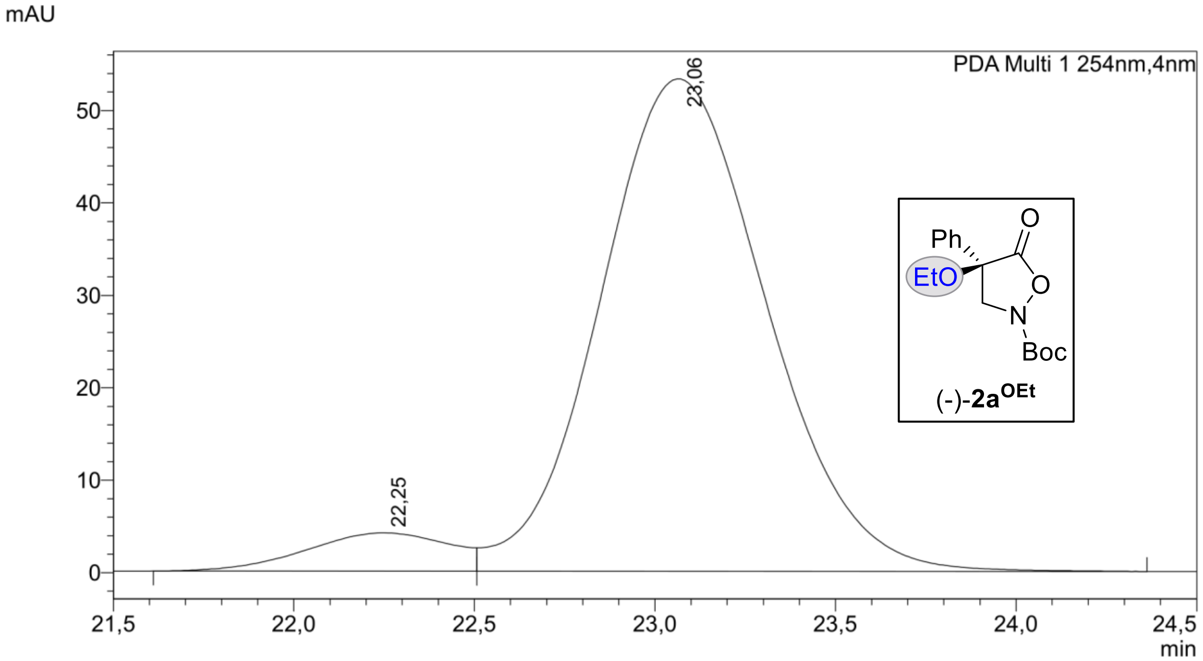

Peak Table

PDA Ch1 254nm

Peak#	Ret. Time	Area	Area%
1	22,25	114650	6
2	23,06	1704633	94
Total		1819283	100

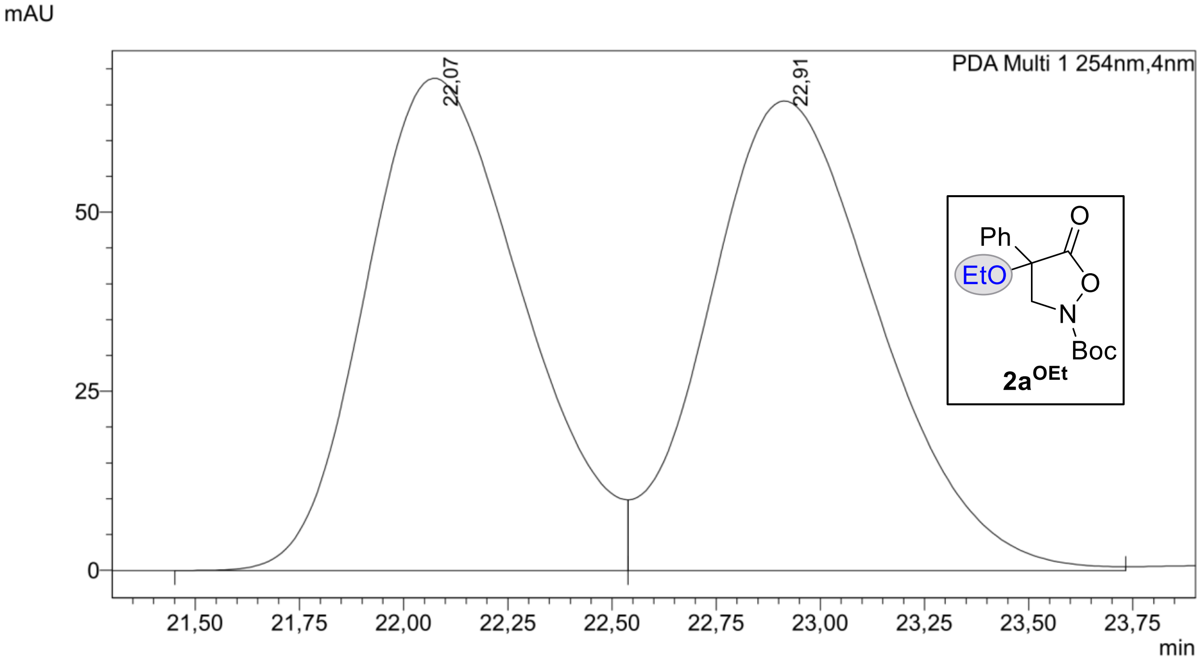

Peak Table

PDA Ch1 254nm

Peak#	Ret. Time	Area	Area%
1	22,07	1812956	49
2	22,91	1872080	51
Total		3685036	100

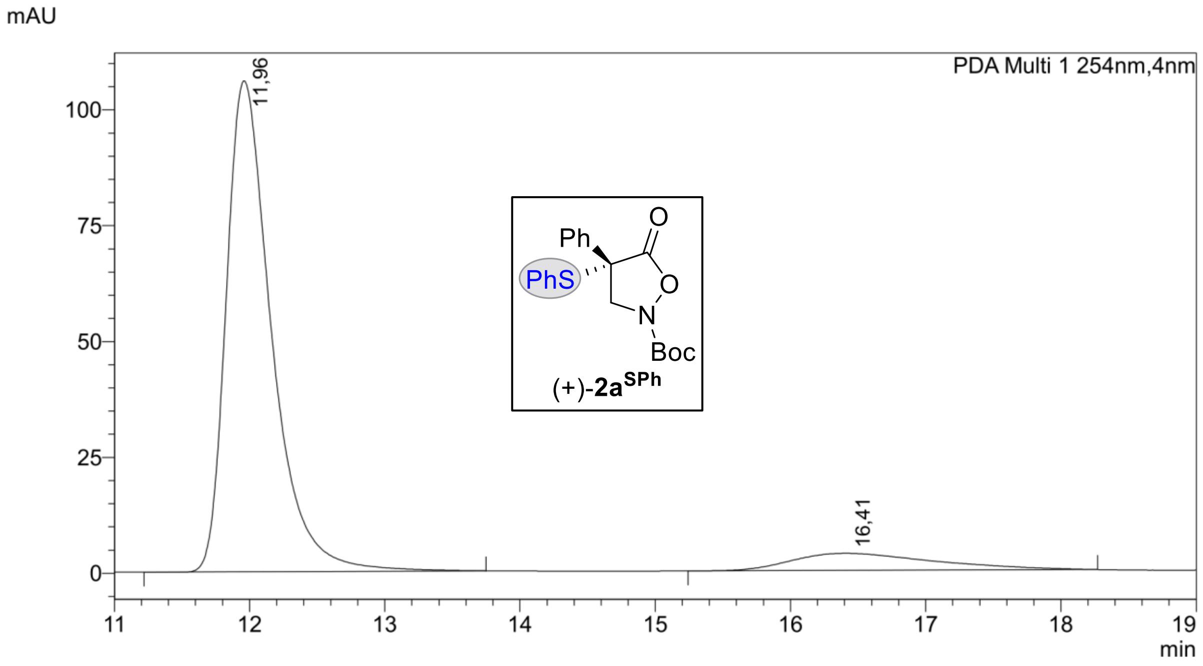

Peak Table

PDA Ch1 254nm

Peak#	Ret. Time	Area	Area%
1	11,96	2459568	90
2	16,41	270438	10
Total		2730007	100

Chromatogram

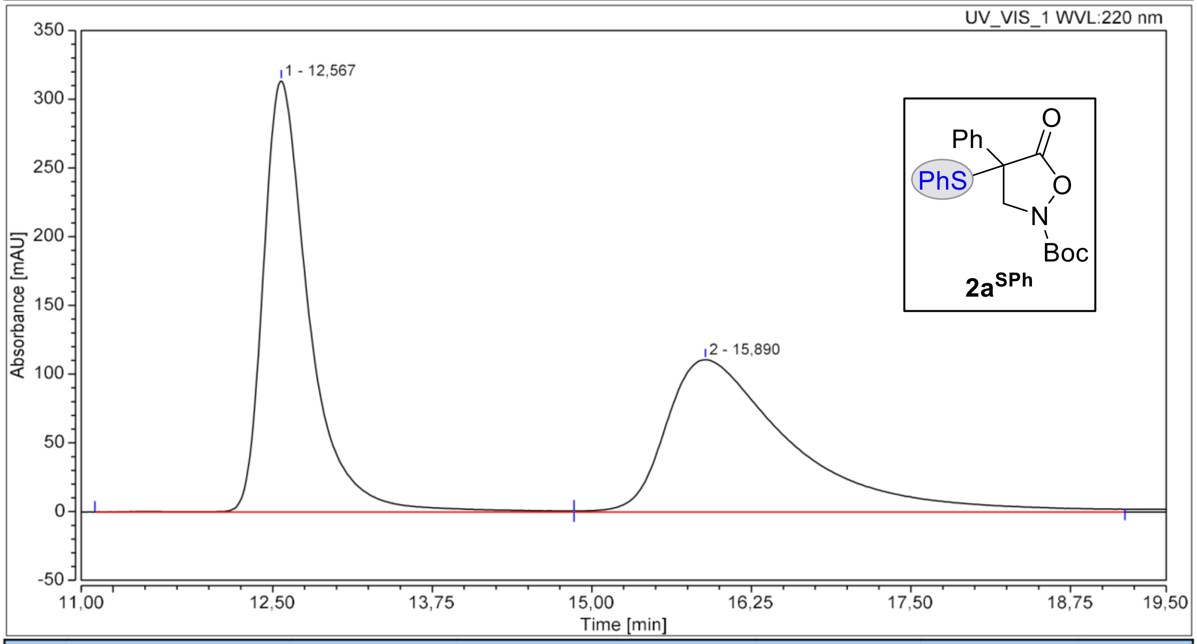

Integration Results

No.	Peak Name	Retention Time min	Area mAU*min	Height mAU	Relative Area %	Relative Height %	Amount n.a.
1		12,567	130,685	313,643	50,80	73,91	n.a.
2		15,890	126,583	110,736	49,20	26,09	n.a.
**Total:**			**257,268**	**424,379**	**100,00**	**100,00**	

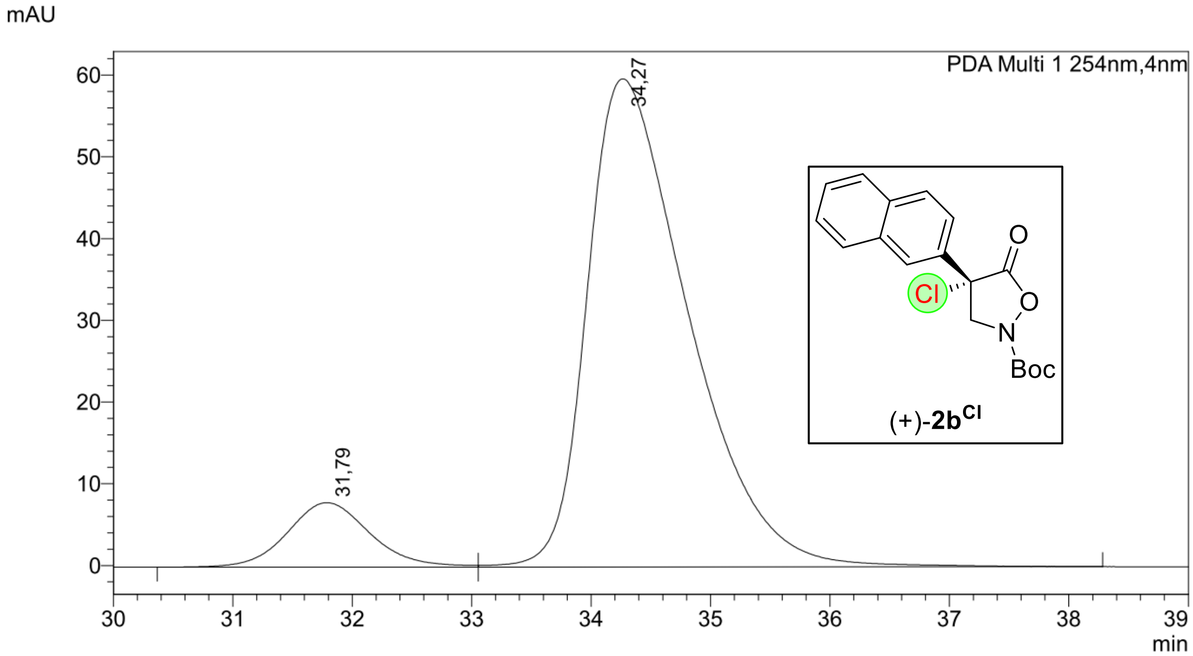

Peak Table

PDA Ch1 254nm

Peak#	Ret. Time	Area	Area%
1	31,79	380874	10
2	34,27	3516792	90
Total		3897666	100

Chromatogram

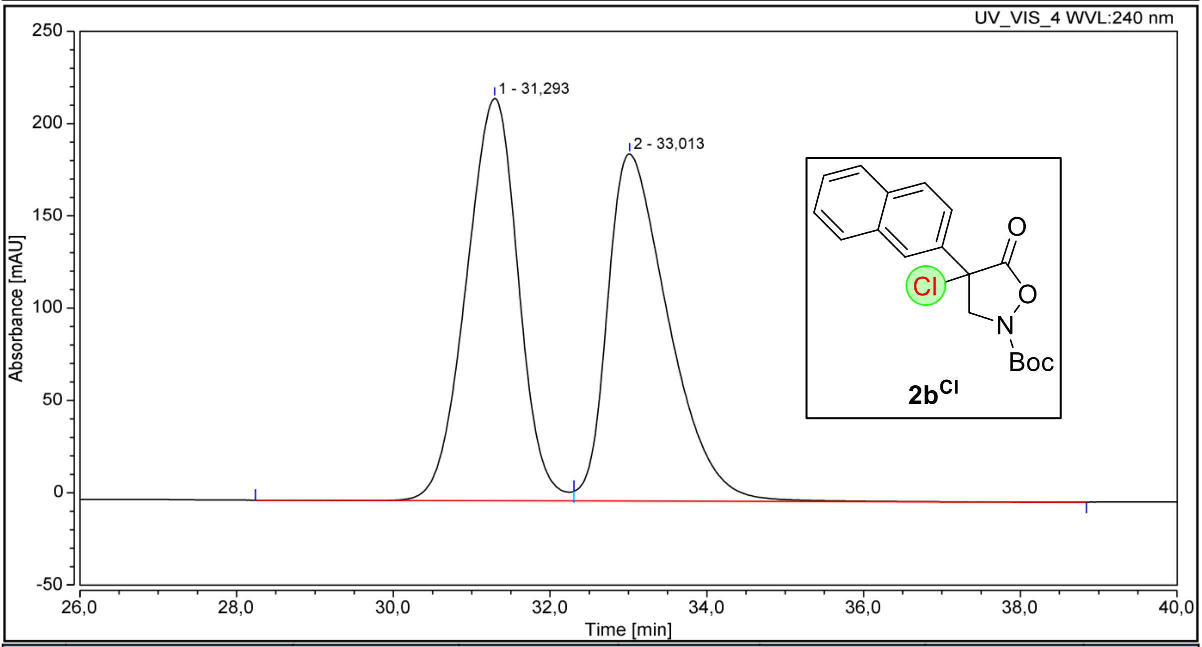

Integration Results

No.	Peak Name	Retention Time min	Area mAU*min	Height mAU	Relative Area %	Relative Height %	Amount n.a.
1		31,293	167,435	217,977	49,73	53,69	n.a.
2		33,013	169,236	188,000	50,27	46,31	n.a.
**Total:**			**336,671**	**405,977**	**100,00**	**100,00**	

Chromatogram

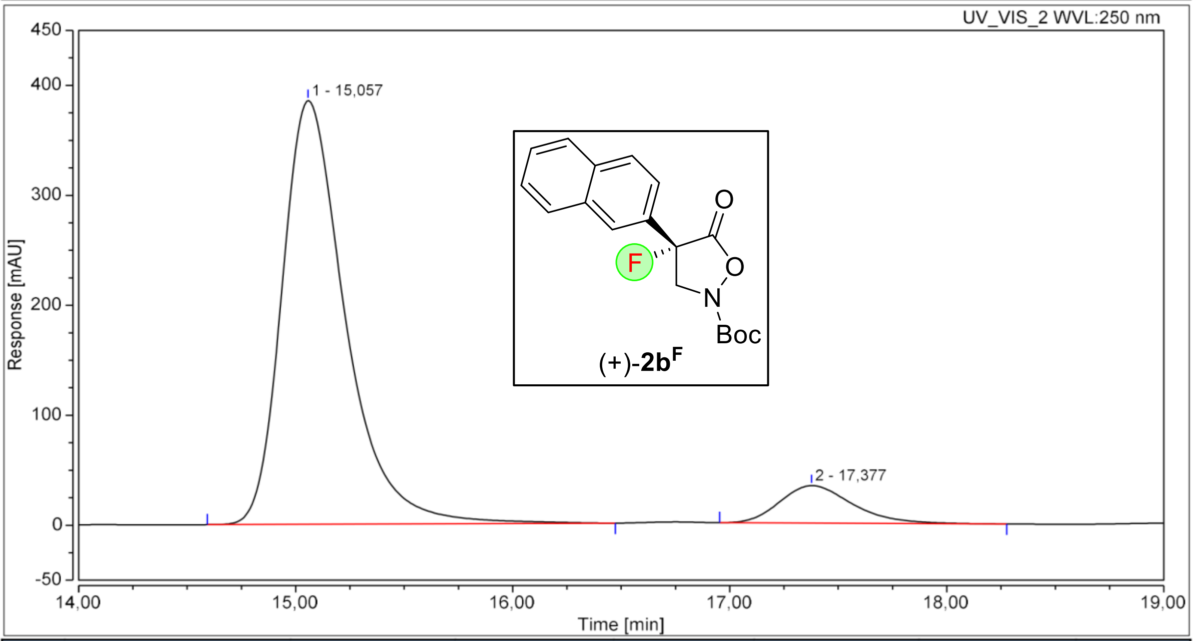

Integration Results

No.	Peak Name	Retention Time min	Area mAU*min	Height mAU	Relative Area %	Relative Height %	Amount
n.a.	Peak 1	n.a.	n.a.	n.a.	n.a.	n.a.	n.a.
1		15,057	130,379	385,453	90,80	91,89	n.a.
2		17,377	13,210	34,034	9,20	8,11	n.a.
**Total:**			**143,589**	**419,487**	**100,00**	**100,00**	

Chromatogram

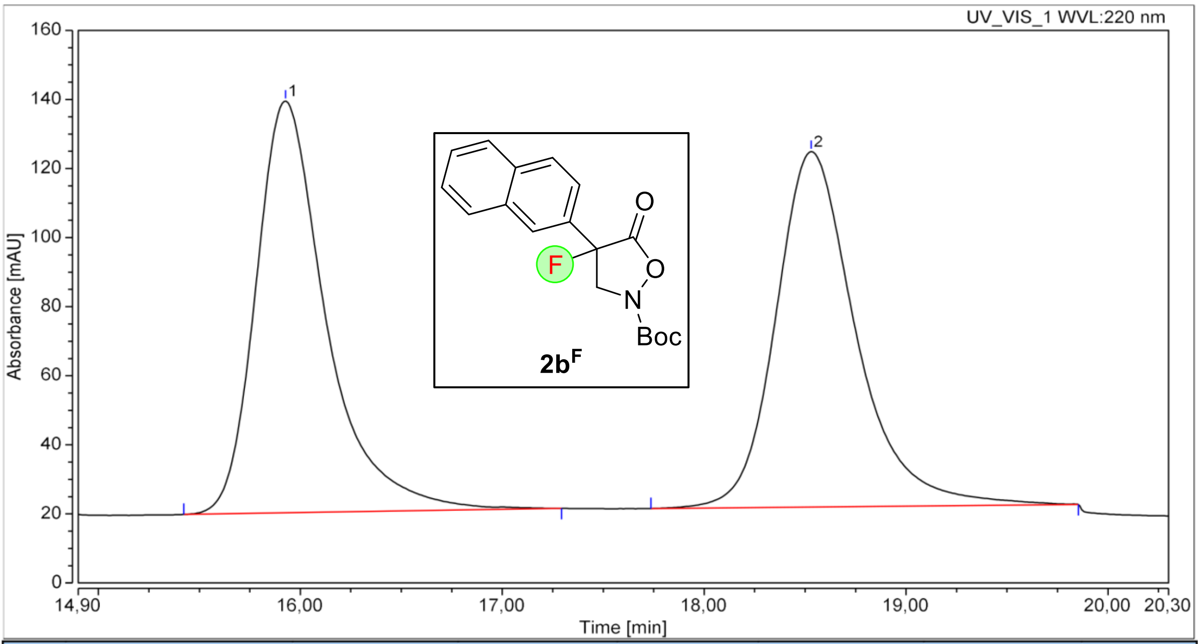

Integration Results

No.	Peak Name	Retention Time min	Area mAU*min	Height mAU	Relative Area %	Relative Height %	Amount n.a.
1		15,927	48,117	119,230	50,32	53,67	n.a.
2		18,530	47,506	102,911	49,68	46,33	n.a.
**Total:**			**95,623**	**222,141**	**100,00**	**100,00**	

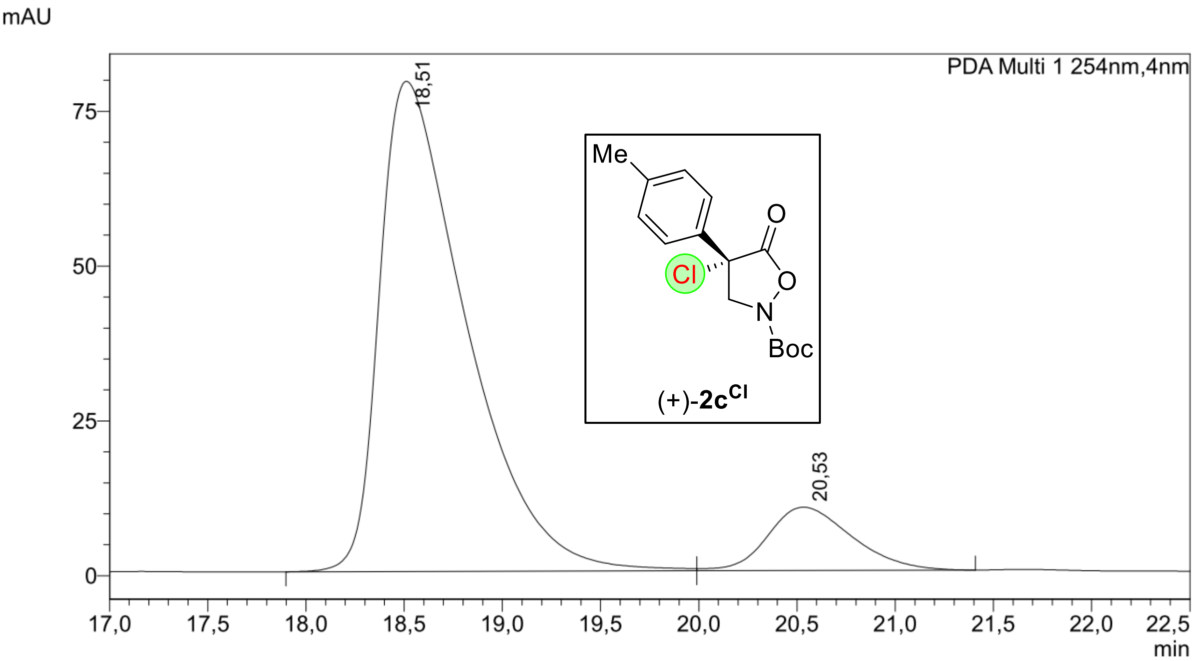

Peak Table

PDA Ch1 254nm

Peak#	Ret. Time	Area	Area%
1	18,51	2430600	89
2	20,53	305647	11
Total		2736247	100

Chromatogram

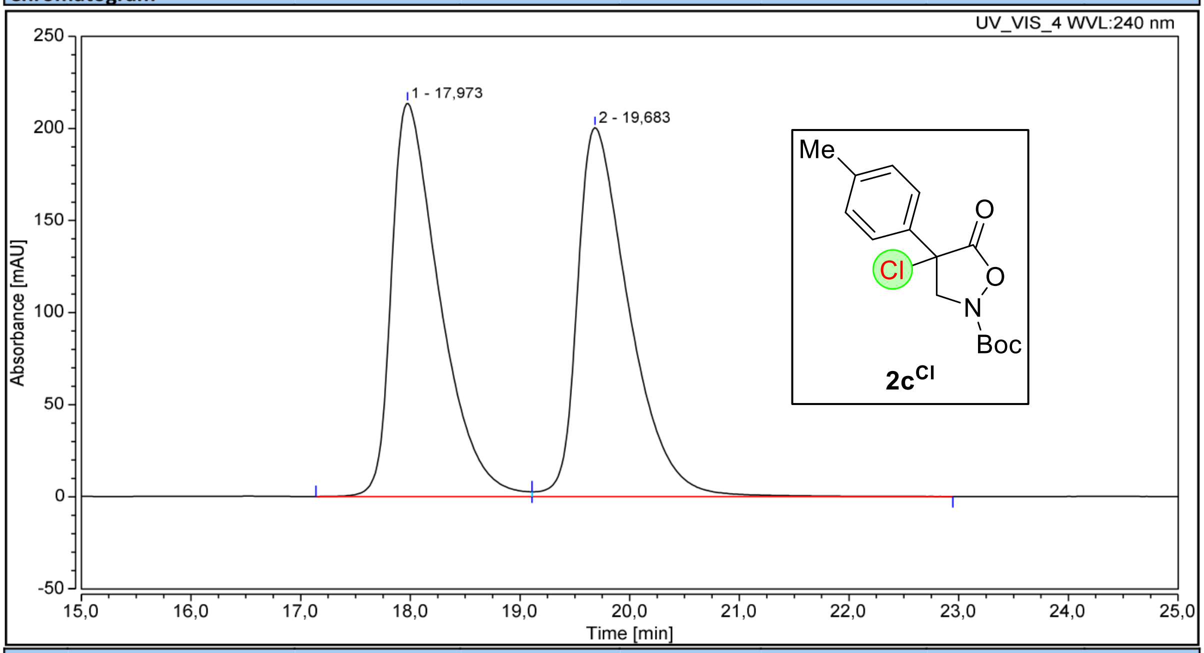

Integration Results

No.	Peak Name	Retention Time min	Area mAU*min	Height mAU	Relative Area %	Relative Height %	Amount n.a.
1		17,973	108,172	213,566	49,71	51,61	n.a.
2		19,683	109,449	200,275	50,29	48,39	n.a.
**Total:**			**217,621**	**413,841**	**100,00**	**100,00**	

Chromatogram

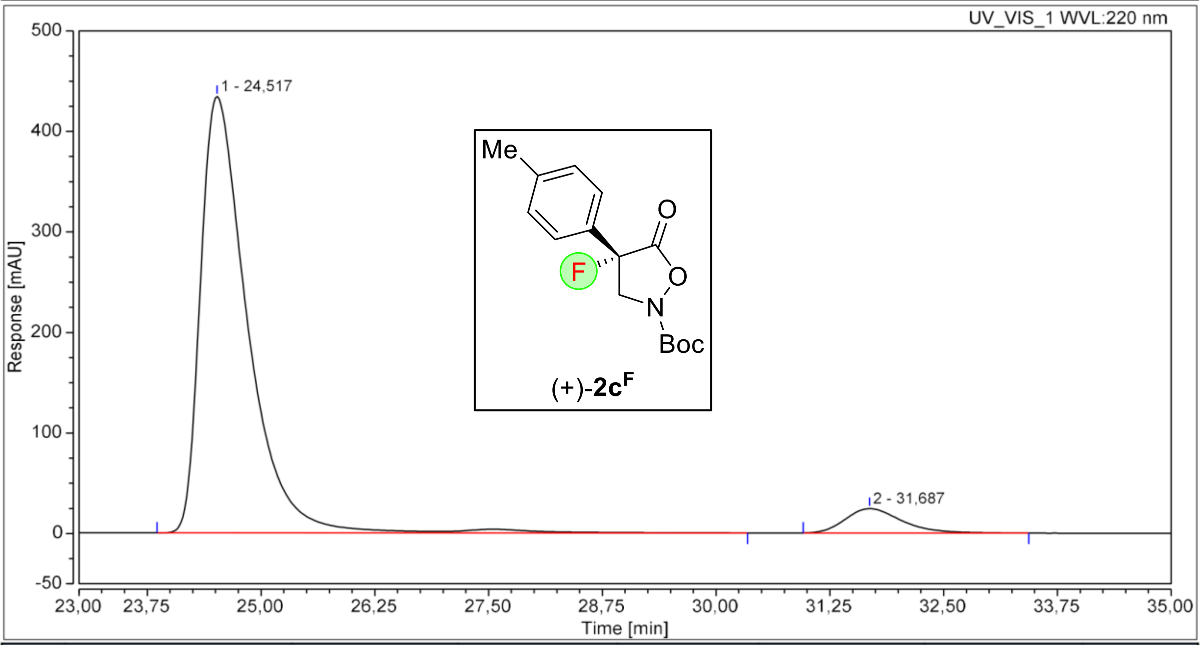

Integration Results

No.	Peak Name	Retention Time min	Area mAU*min	Height mAU	Relative Area %	Relative Height %	Amount
n.a.	Peak 1	n.a.	n.a.	n.a.	n.a.	n.a.	n.a.
1		24,517	262,092	434,152	93,76	94,71	n.a.
2		31,687	17,440	24,267	6,24	5,29	n.a.
**Total:**			**279,532**	**458,419**	**100,00**	**100,00**	

Chromatogram

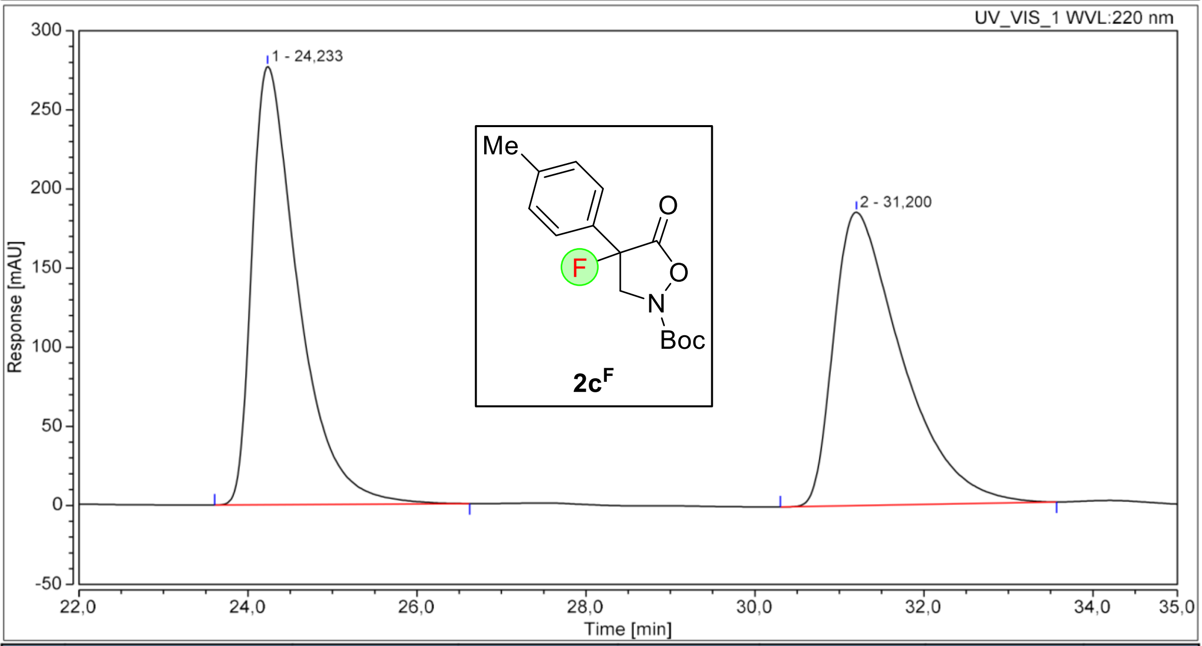

Integration Results

No.	Peak Name	Retention Time min	Area mAU*min	Height mAU	Relative Area %	Relative Height %	Amount
n.a.	Peak 1	n.a.	n.a.	n.a.	n.a.	n.a.	n.a.
1		24,233	175,379	276,957	50,34	59,91	n.a.
2		31,200	173,021	185,332	49,66	40,09	n.a.
**Total:**			**348,400**	**462,289**	**100,00**	**100,00**	

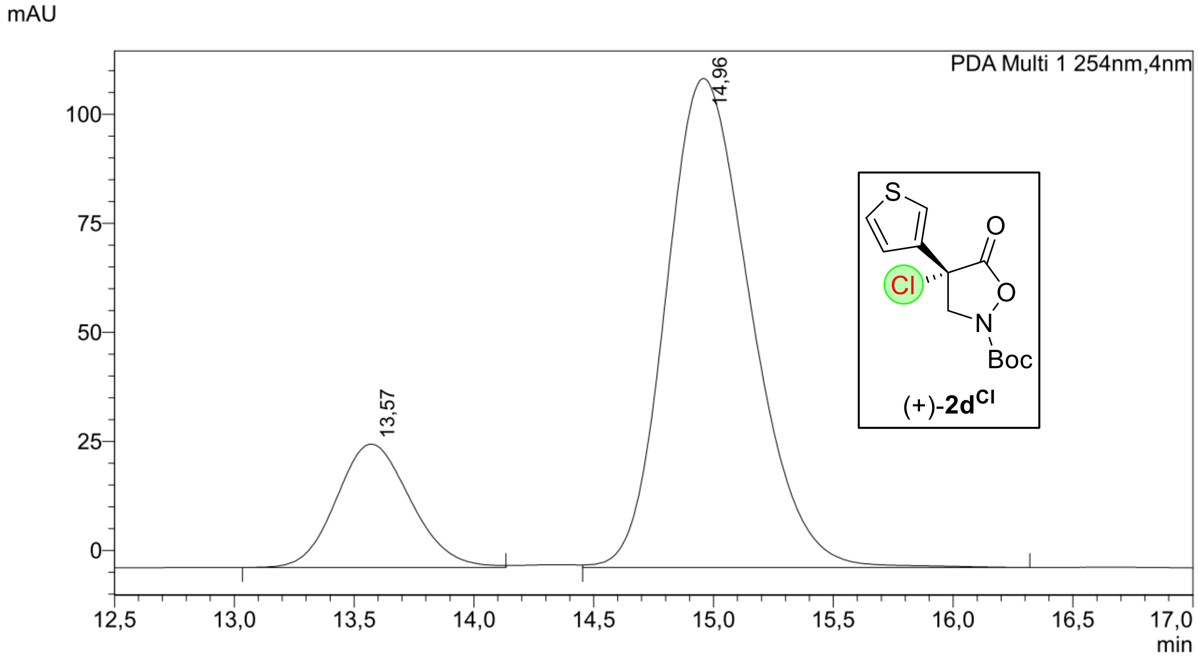

Peak Table

PDA Ch1 254nm

Peak#	Ret. Time	Area	Area%
1	13,57	610865	18
2	14,96	2753317	82
Total		3364182	100

Chromatogram

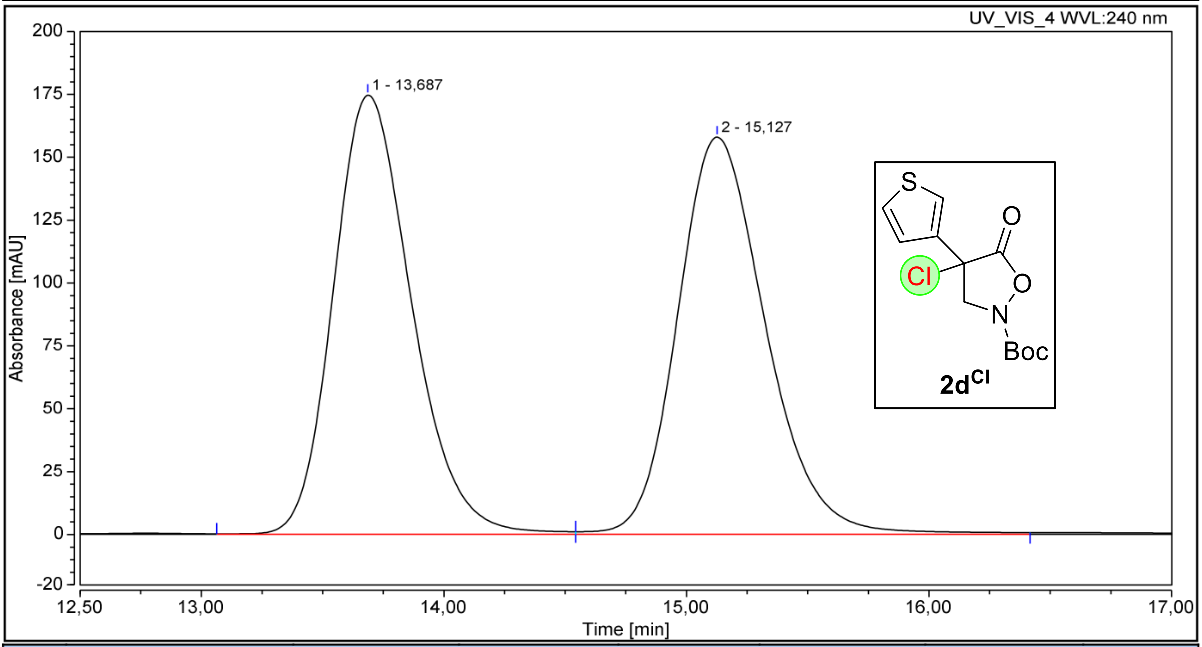

Integration Results

No.	Peak Name	Retention Time min	Area mAU*min	Height mAU	Relative Area %	Relative Height %	Amount n.a.
1		13,687	65,316	174,628	49,51	52,51	n.a.
2		15,127	66,608	157,952	50,49	47,49	n.a.
**Total:**			**131,923**	**332,580**	**100,00**	**100,00**	

Chromatogram

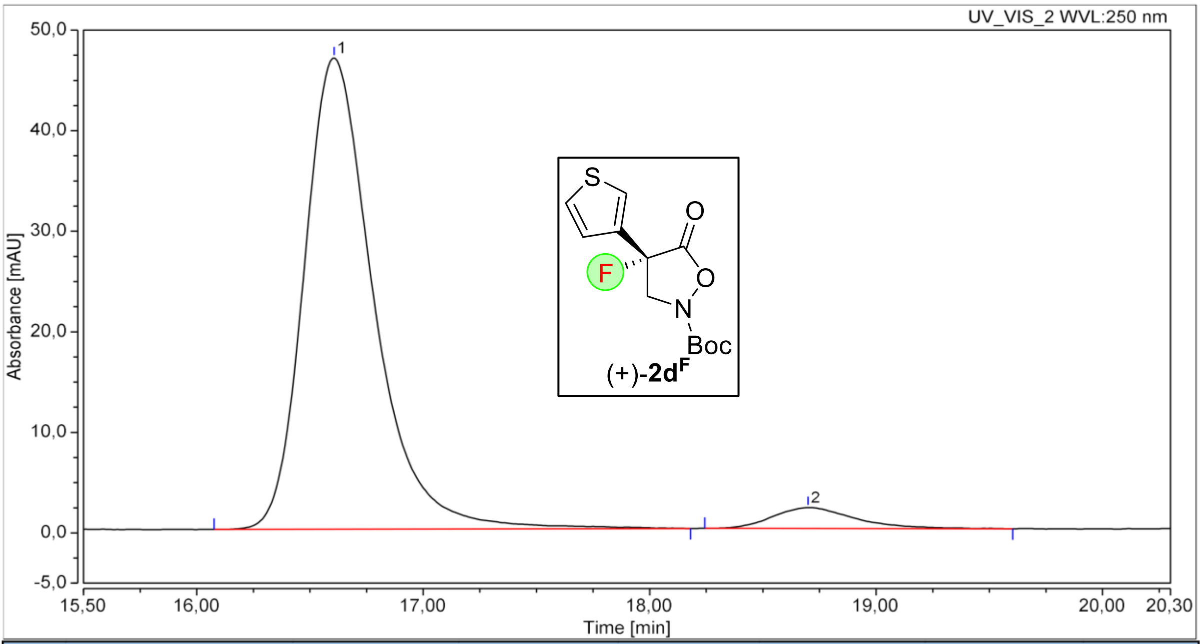

Integration Results

No.	Peak Name	Retention Time min	Area mAU*min	Height mAU	Relative Area %	Relative Height %	Amount n.a.
1		16,607	16,675	46,864	95,04	95,76	n.a.
2		18,700	0,871	2,073	4,96	4,24	n.a.
**Total:**			**17,546**	**48,937**	**100,00**	**100,00**	

Chromatogram

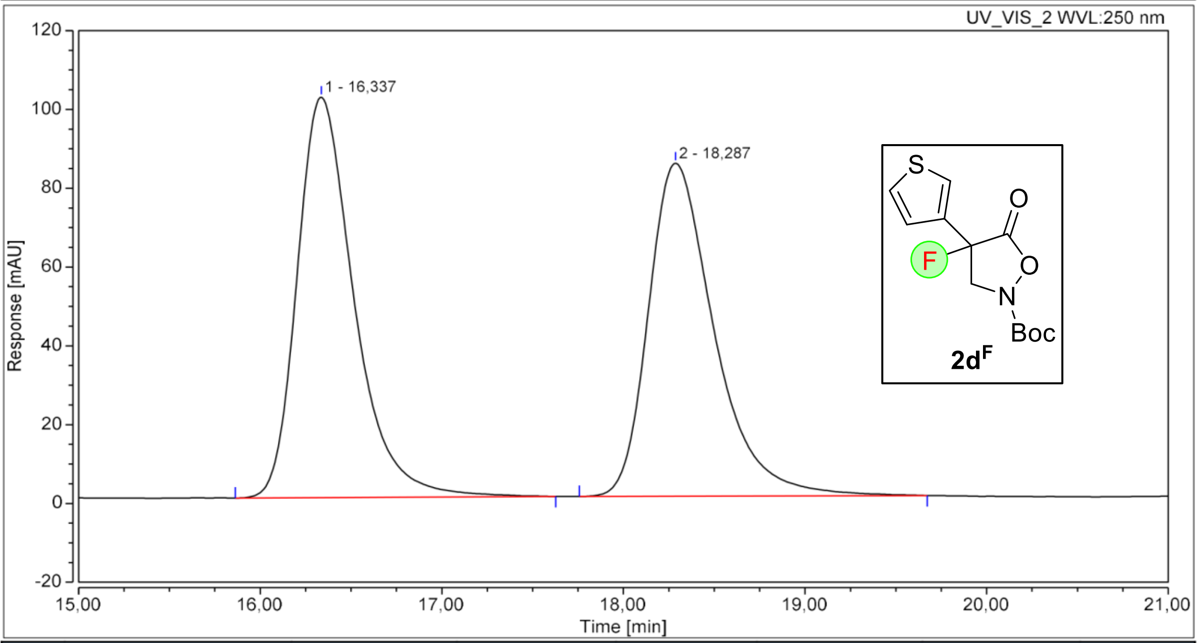

Integration Results

No.	Peak Name	Retention Time min	Area mAU*min	Height mAU	Relative Area %	Relative Height %	Amount
n.a.	Peak 1	n.a.	n.a.	n.a.	n.a.	n.a.	n.a.
1		16,337	36,855	101,648	50,94	54,59	n.a.
2		18,287	35,497	84,543	49,06	45,41	n.a.
**Total:**			**72,352**	**186,191**	**100,00**	**100,00**	

Chromatogram

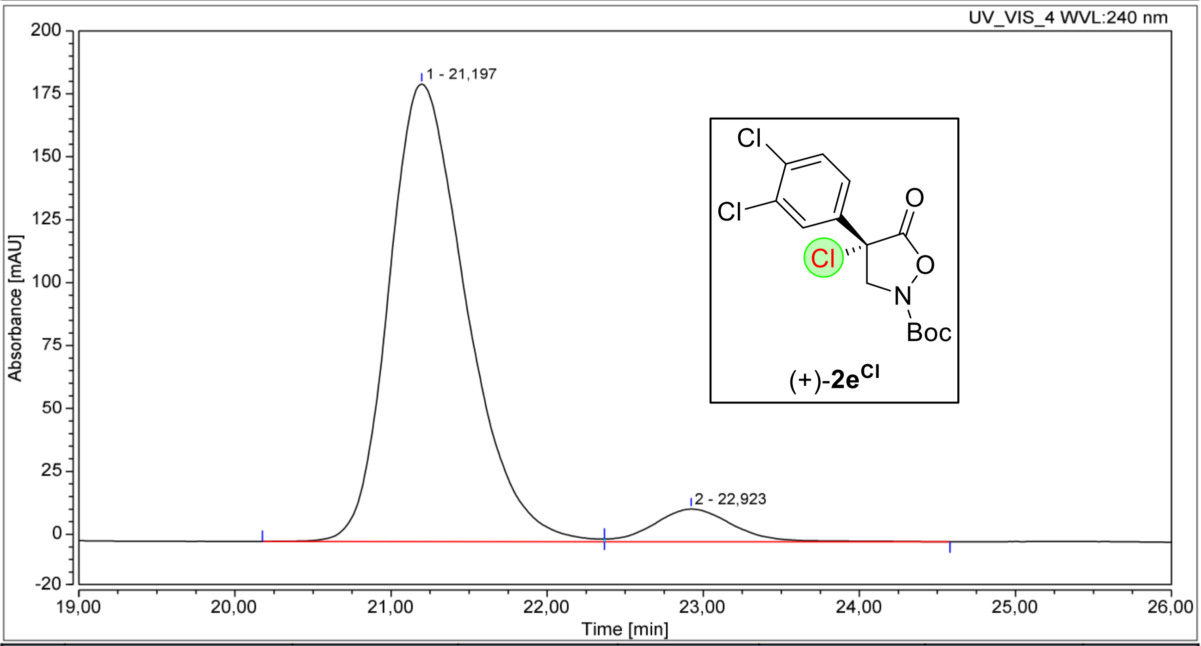

Integration Results

No.	Peak Name	Retention Time min	Area mAU*min	Height mAU	Relative Area %	Relative Height %	Amount n.a.
1		21,197	104,155	181,724	93,20	93,34	n.a.
2		22,923	7,597	12,976	6,80	6,66	n.a.
**Total:**			**111,752**	**194,700**	**100,00**	**100,00**	

Chromatogram

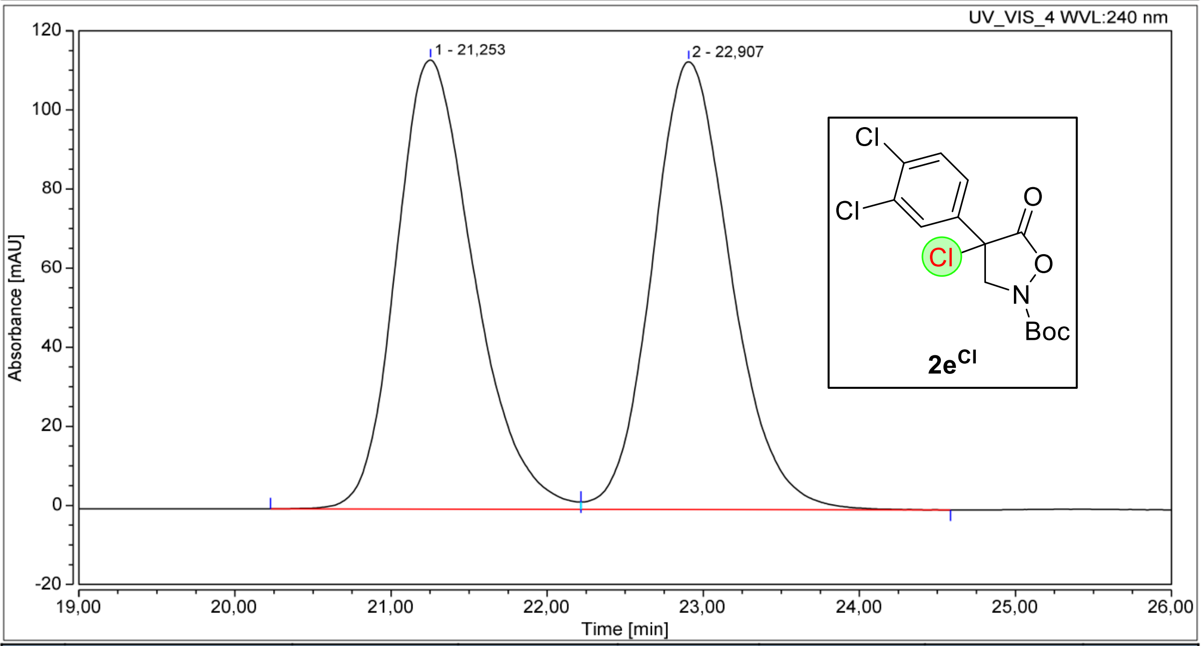

Integration Results

No.	Peak Name	Retention Time min	Area mAU*min	Height mAU	Relative Area %	Relative Height %	Amount n.a.
1		21,253	65,748	113,543	49,76	50,07	n.a.
2		22,907	66,395	113,215	50,24	49,93	n.a.
**Total:**			**132,143**	**226,758**	**100,00**	**100,00**	

Chromatogram

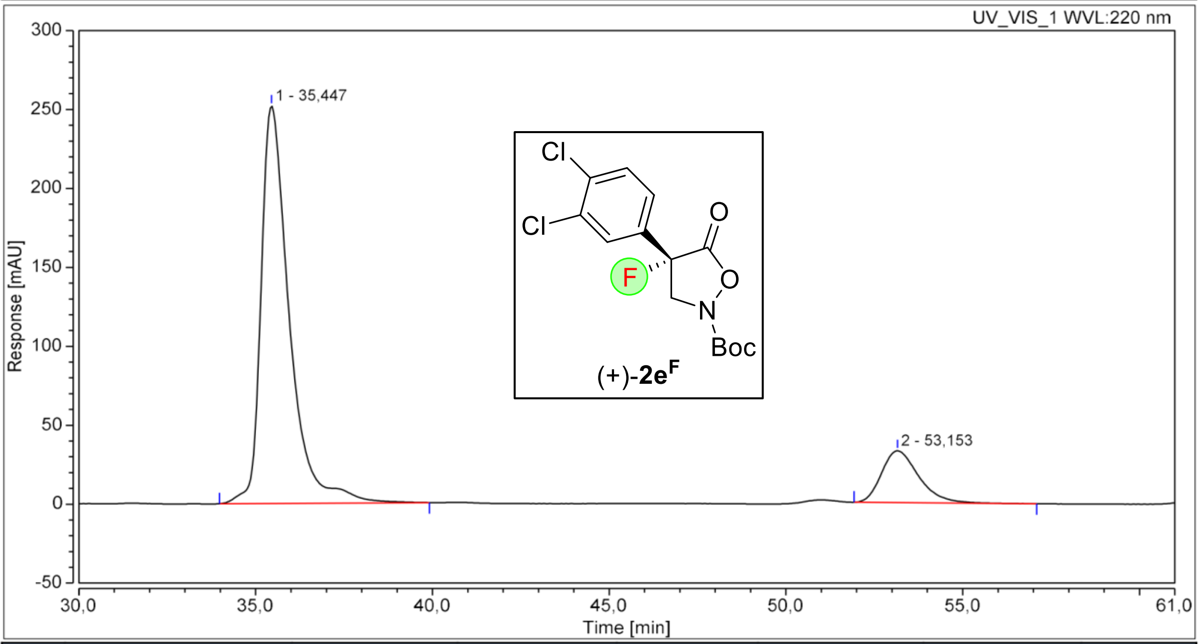

Integration Results

No.	Peak Name	Retention Time min	Area mAU*min	Height mAU	Relative Area %	Relative Height %	Amount
n.a.	Peak 1	n.a.	n.a.	n.a.	n.a.	n.a.	n.a.
1		35,447	233,093	251,788	85,32	88,46	n.a.
2		53,153	40,107	32,836	14,68	11,54	n.a.
**Total:**			**273,201**	**284,624**	**100,00**	**100,00**	

Chromatogram

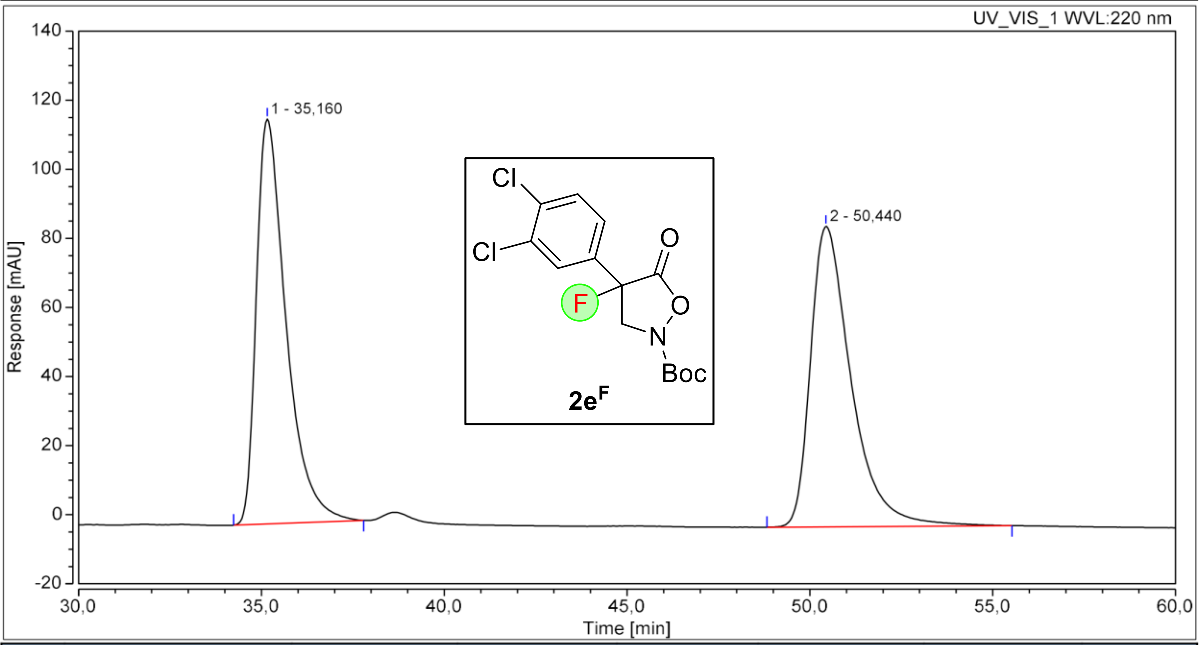

Integration Results

No.	Peak Name	Retention Time min	Area mAU*min	Height mAU	Relative Area %	Relative Height %	Amount
n.a.	Peak 1	n.a.	n.a.	n.a.	n.a.	n.a.	n.a.
1		35,160	110,426	117,269	49,66	57,40	n.a.
2		50,440	111,954	87,016	50,34	42,60	n.a.
**Total:**			**222,380**	**204,284**	**100,00**	**100,00**	

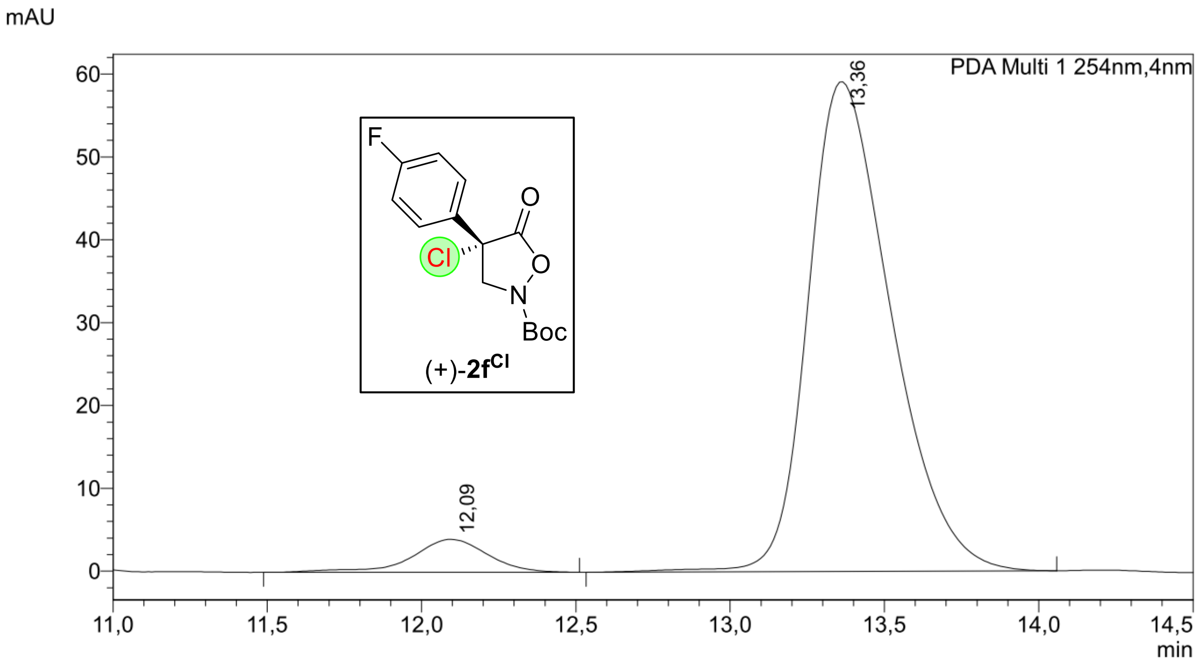

Peak Table

PDA Ch1 254nm

Peak#	Ret. Time	Area	Area%
1	12,09	68737	6
2	13,36	1105993	94
Total		1174730	100

Chromatogram

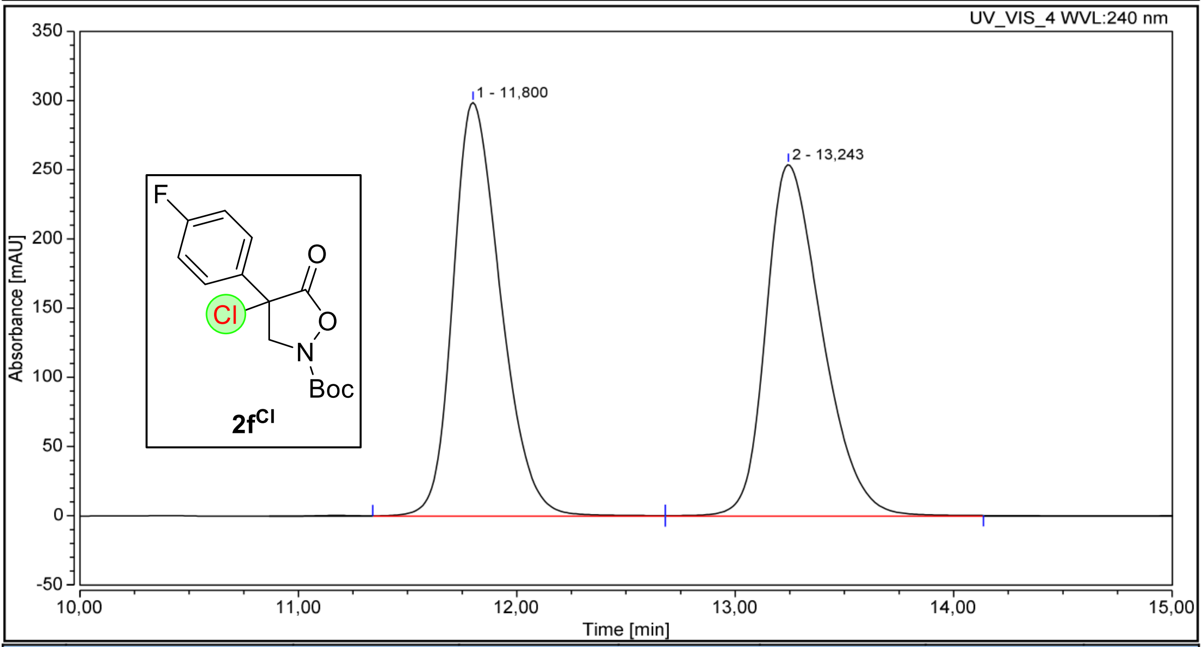

Integration Results

No.	Peak Name	Retention Time min	Area mAU*min	Height mAU	Relative Area %	Relative Height %	Amount n.a.
1		11,800	75,838	298,904	49,99	54,06	n.a.
2		13,243	75,853	254,054	50,01	45,94	n.a.
**Total:**			**151,691**	**552,958**	**100,00**	**100,00**	

Chromatogram

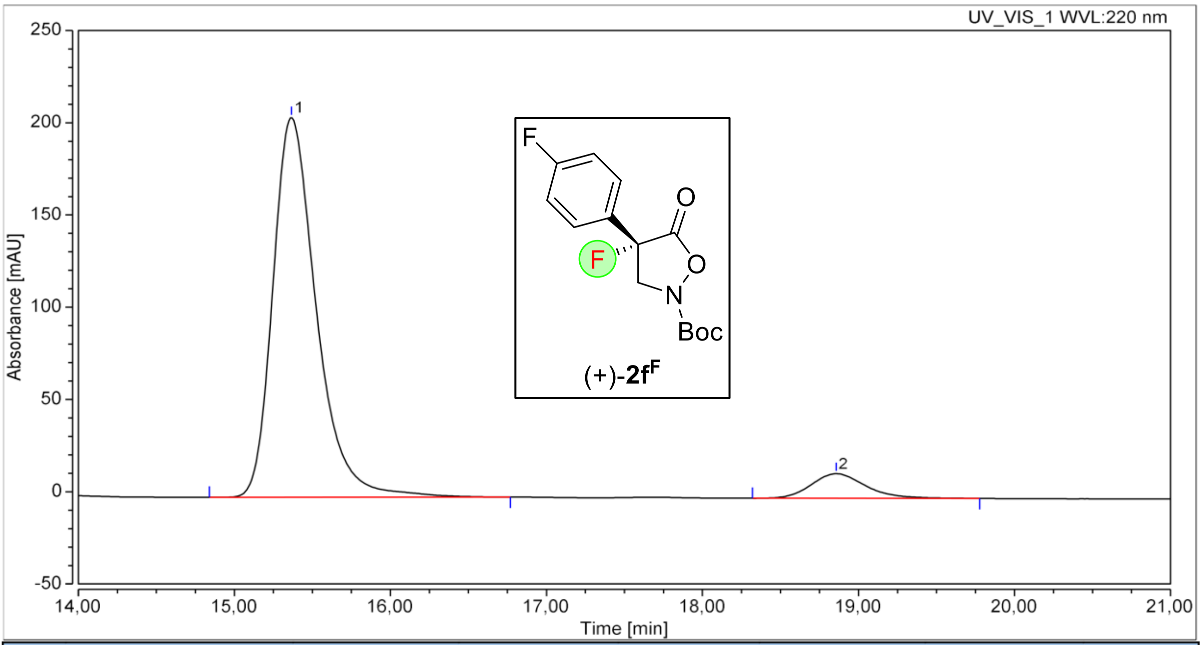

Integration Results

No.	Peak Name	Retention Time min	Area mAU*min	Height mAU	Relative Area %	Relative Height %	Amount n.a.
1		15,367	66,375	205,794	92,46	93,91	n.a.
2		18,857	5,410	13,351	7,54	6,09	n.a.
**Total:**			**71,785**	**219,145**	**100,00**	**100,00**	

Chromatogram

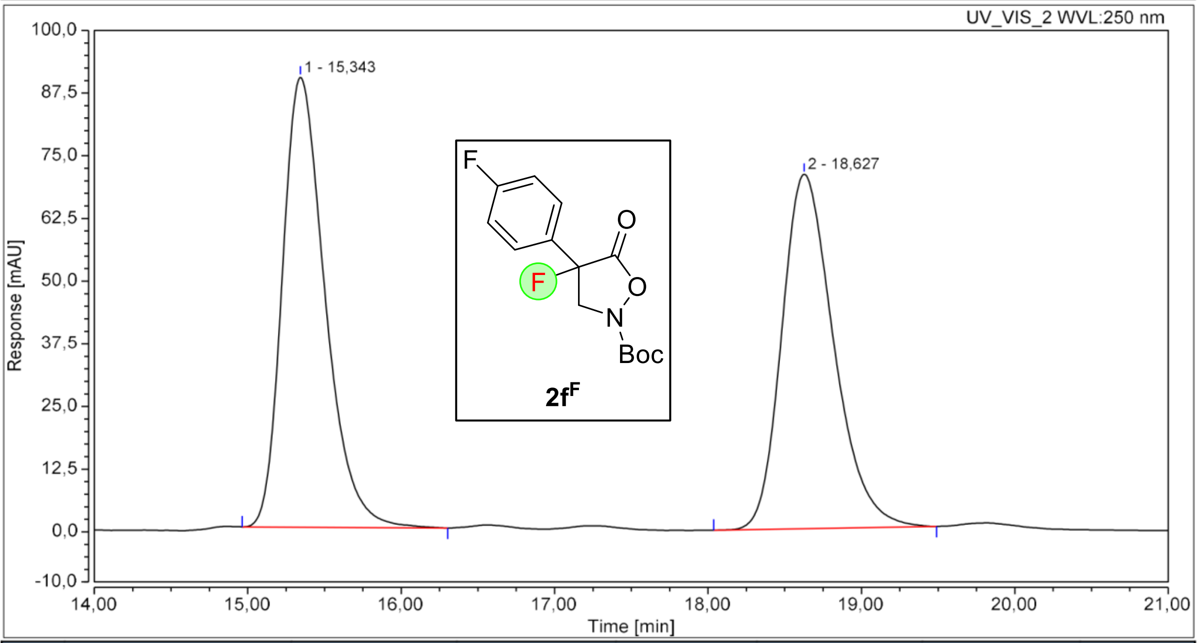

Integration Results

No.	Peak Name	Retention Time min	Area mAU*min	Height mAU	Relative Area %	Relative Height %	Amount
n.a.	Peak 1	n.a.	n.a.	n.a.	n.a.	n.a.	n.a.
1		15,343	28,929	89,752	51,07	55,94	n.a.
2		18,627	27,718	70,681	48,93	44,06	n.a.
**Total:**			**56,647**	**160,433**	**100,00**	**100,00**	

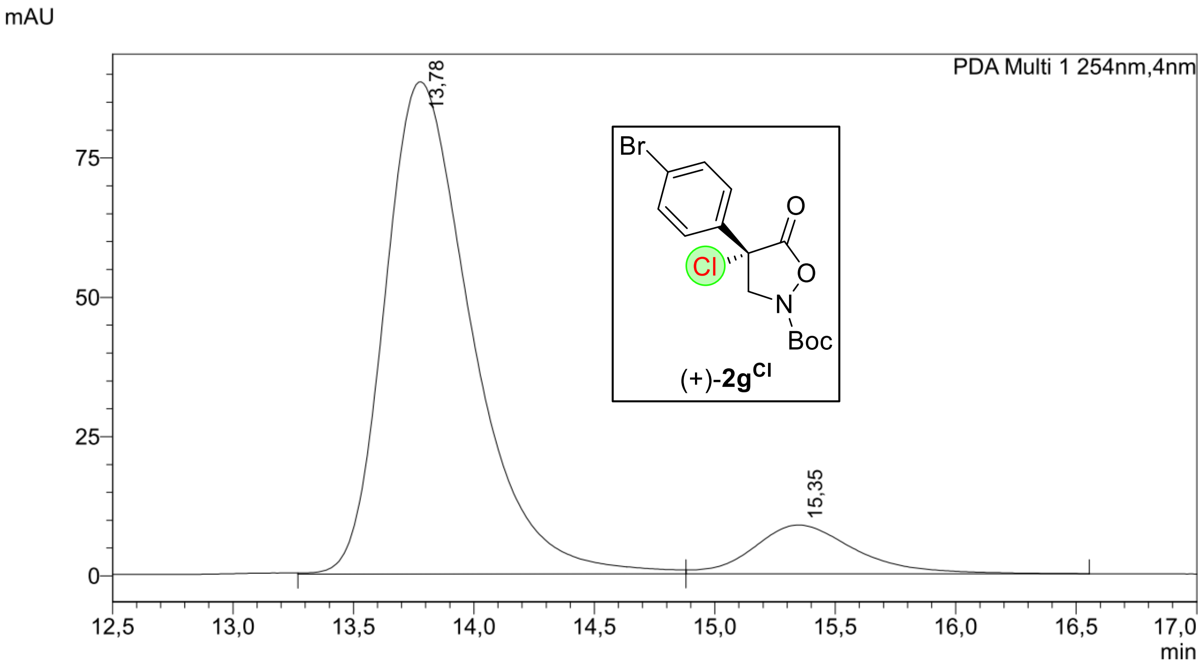

Peak Table

PDA Ch1 254nm

Peak#	Ret. Time	Area	Area%
1	13,78	2213249	90
2	15,35	259268	10
Total		2472516	100

Chromatogram

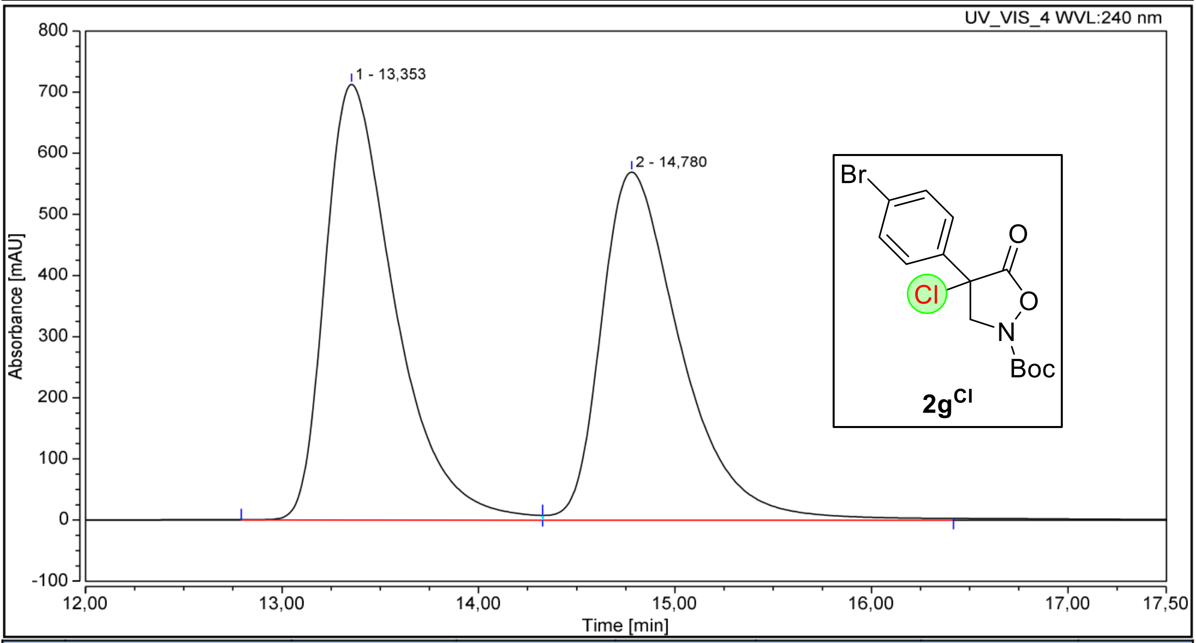

Integration Results

No.	Peak Name	Retention Time min	Area mAU*min	Height mAU	Relative Area %	Relative Height %	Amount n.a.
1		13,353	293,057	712,685	52,21	55,59	n.a.
2		14,780	268,257	569,383	47,79	44,41	n.a.
**Total:**			**561,314**	**1282,068**	**100,00**	**100,00**	

Chromatogram

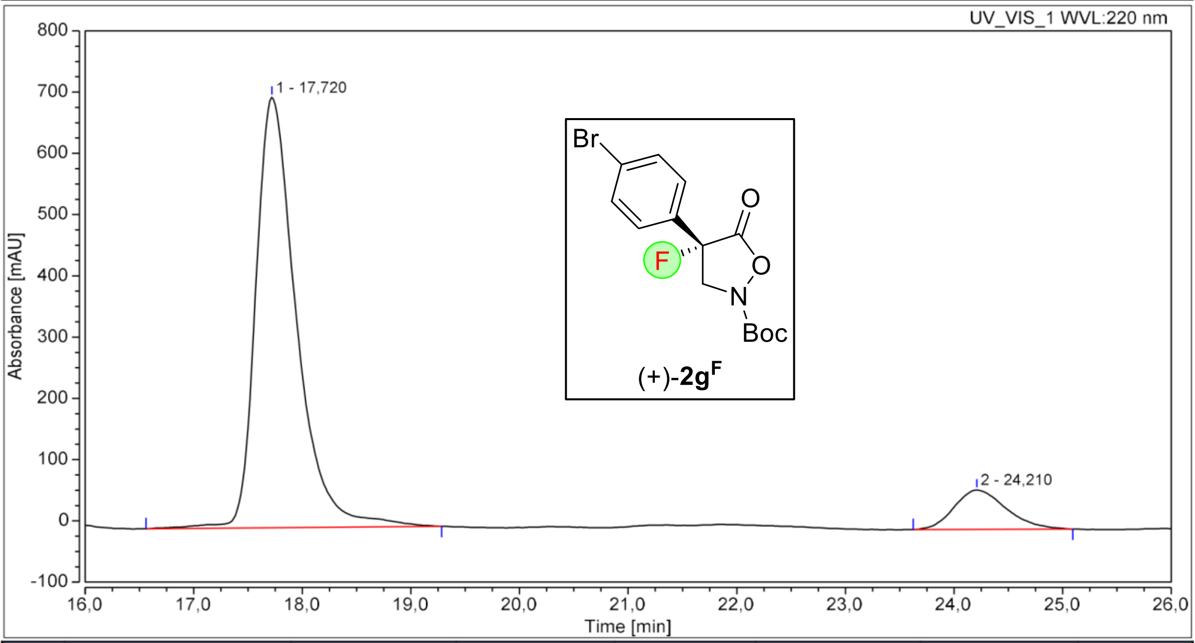

Integration Results

No.	Peak Name	Retention Time min	Area mAU*min	Height mAU	Relative Area %	Relative Height %	Amount
n.a.	Peak 1	n.a.	n.a.	n.a.	n.a.	n.a.	n.a.
1		17,720	304,776	702,639	89,56	91,65	n.a.
2		24,210	35,516	63,977	10,44	8,35	n.a.
**Total:**			**340,292**	**766,616**	**100,00**	**100,00**	

Chromatogram

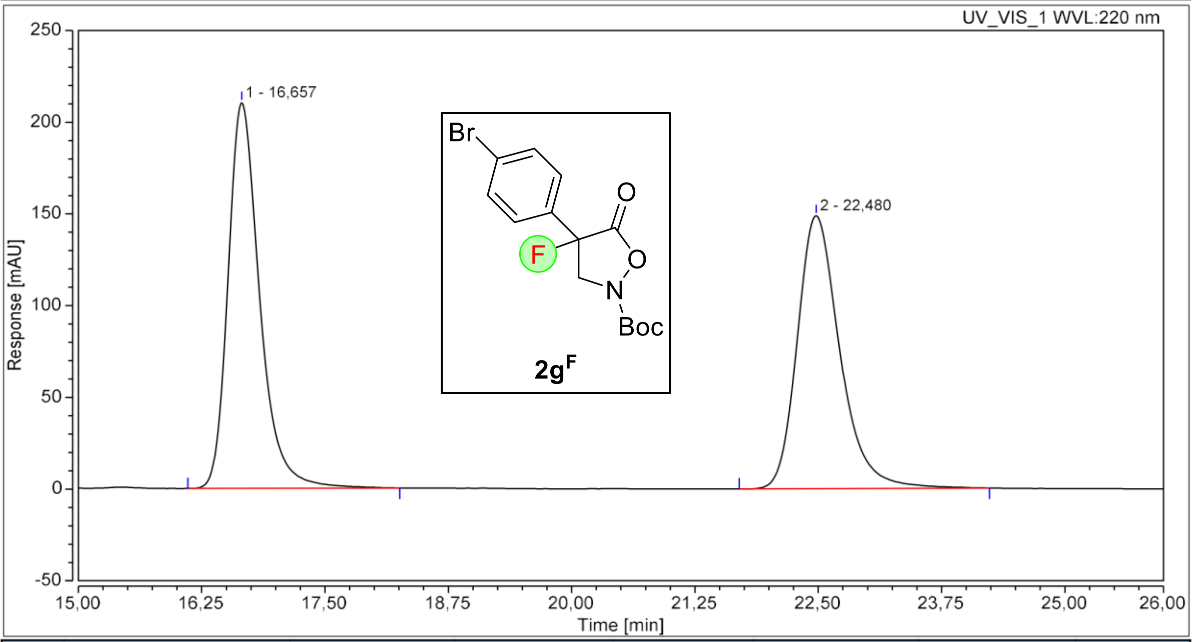

Integration Results

No.	Peak Name	Retention Time min	Area mAU*min	Height mAU	Relative Area %	Relative Height %	Amount
n.a.	Peak 1	n.a.	n.a.	n.a.	n.a.	n.a.	n.a.
1		16,657	78,190	210,292	51,22	58,55	n.a.
2		22,480	74,459	148,856	48,78	41,45	n.a.
**Total:**			**152,649**	**359,148**	**100,00**	**100,00**	

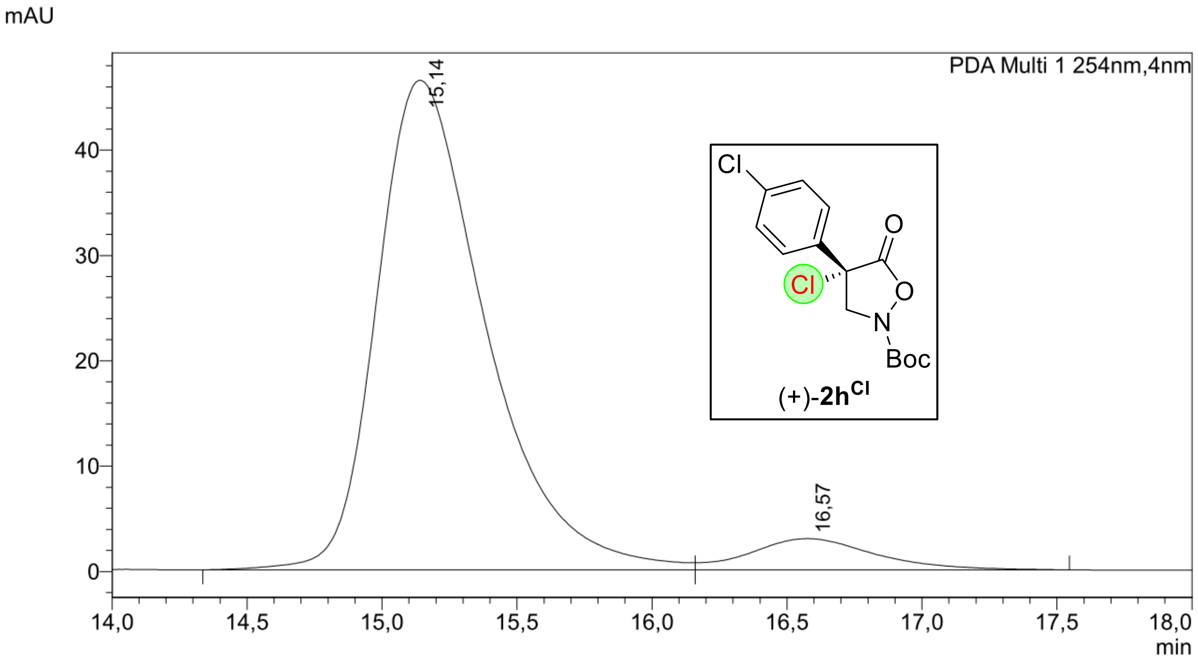

Peak Table

PDA Ch1 254nm

Peak#	Ret. Time	Area	Area%
1	15,14	1305881	93
2	16,57	95058	7
Total		1400939	100

Chromatogram

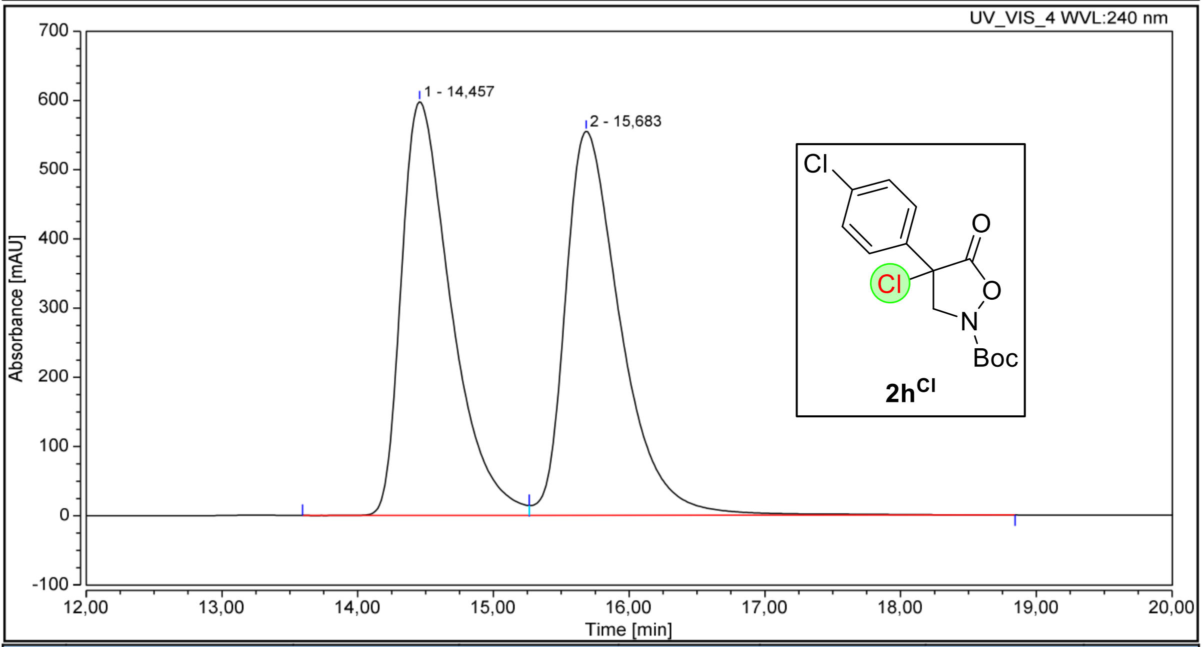

Integration Results

No.	Peak Name	Retention Time min	Area mAU*min	Height mAU	Relative Area %	Relative Height %	Amount n.a.
1		14,457	253,603	597,607	49,15	51,86	n.a.
2		15,683	262,418	554,821	50,85	48,14	n.a.
**Total:**			**516,021**	**1152,427**	**100,00**	**100,00**	

Chromatogram

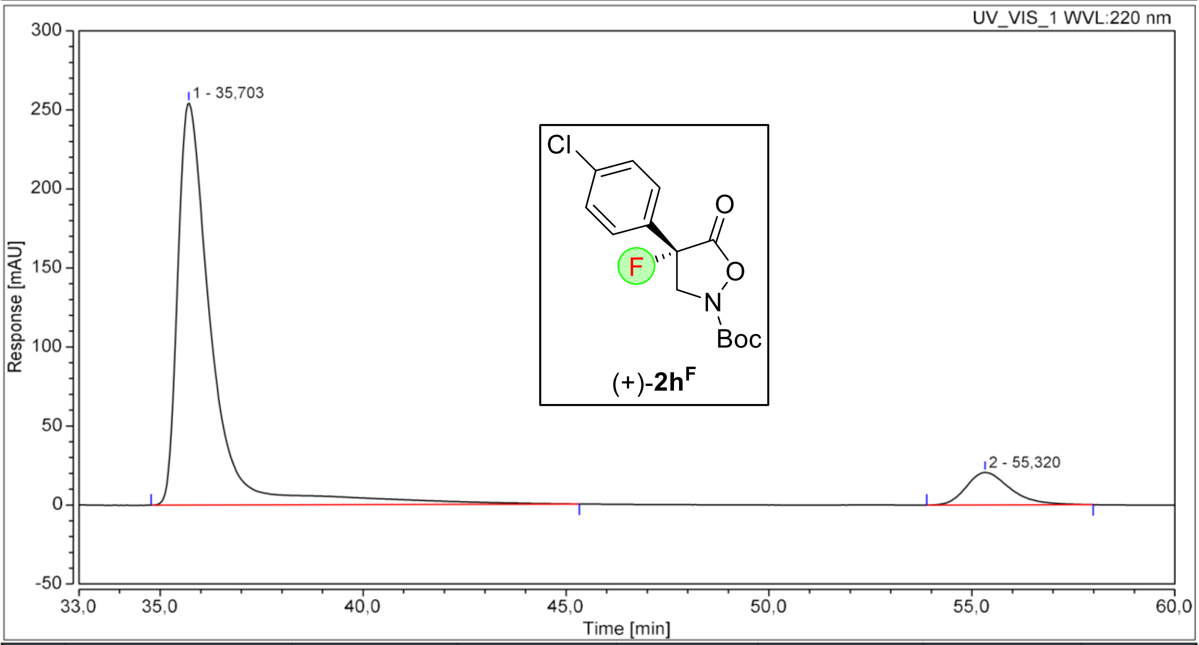

Integration Results

No.	Peak Name	Retention Time min	Area mAU*min	Height mAU	Relative Area %	Relative Height %	Amount
n.a.	Peak 1	n.a.	n.a.	n.a.	n.a.	n.a.	n.a.
1		35,703	246,781	254,407	90,52	92,53	n.a.
2		55,320	25,850	20,546	9,48	7,47	n.a.
**Total:**			**272,631**	**274,953**	**100,00**	**100,00**	

Chromatogram

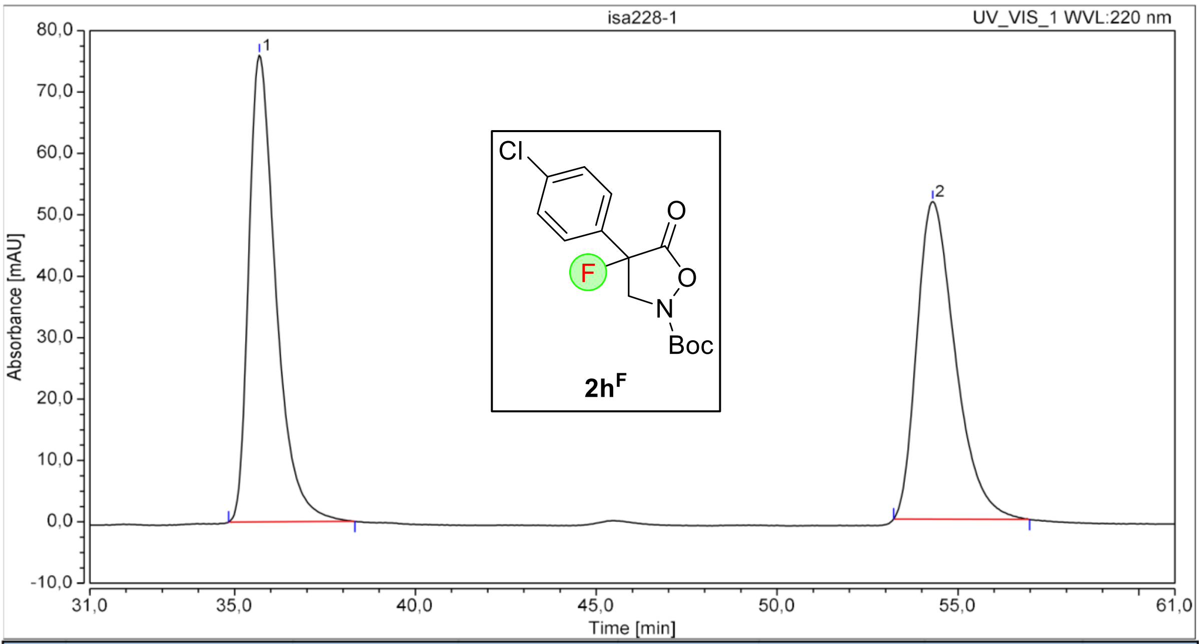

Integration Results

No.	Peak Name	Retention Time min	Area mAU*min	Height mAU	Relative Area %	Relative Height %	Amount n.a.
1		35,687	66,087	76,049	50,94	59,51	n.a.
2		54,307	63,660	51,734	49,06	40,49	n.a.
**Total:**			**129,747**	**127,783**	**100,00**	**100,00**	

Chromatogram

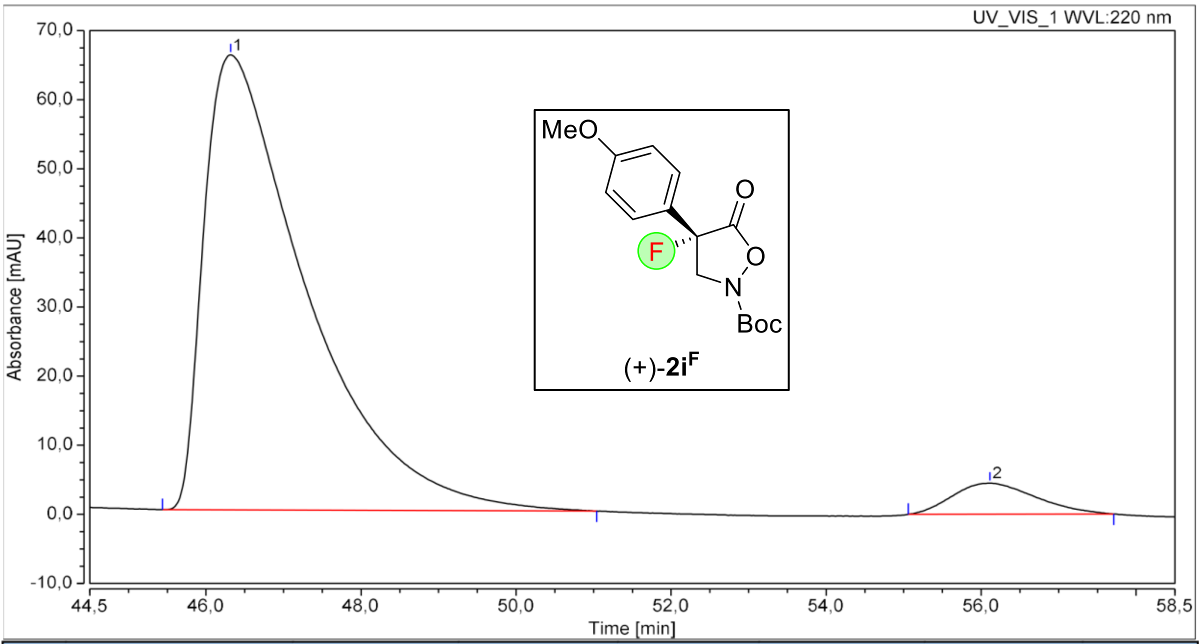

Integration Results

No.	Peak Name	Retention Time min	Area mAU*min	Height mAU	Relative Area %	Relative Height %	Amount n.a.
1		46,317	100,805	65,828	94,74	93,59	n.a.
2		56,113	5,597	4,506	5,26	6,41	n.a.
**Total:**			**106,403**	**70,333**	**100,00**	**100,00**	

Chromatogram

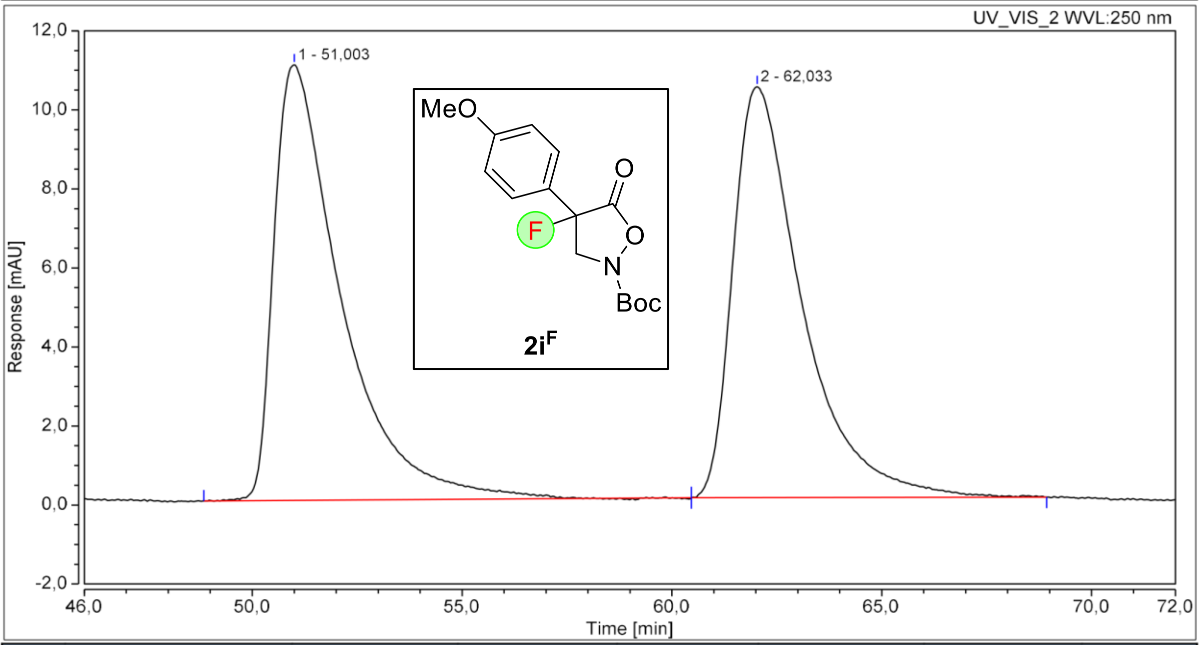

Integration Results

No.	Peak Name	Retention Time min	Area mAU*min	Height mAU	Relative Area %	Relative Height %	Amount
n.a.	Peak 1	n.a.	n.a.	n.a.	n.a.	n.a.	n.a.
1		51,003	20,387	11,020	50,68	51,45	n.a.
2		62,033	19,843	10,400	49,32	48,55	n.a.
**Total:**			**40,230**	**21,420**	**100,00**	**100,00**	

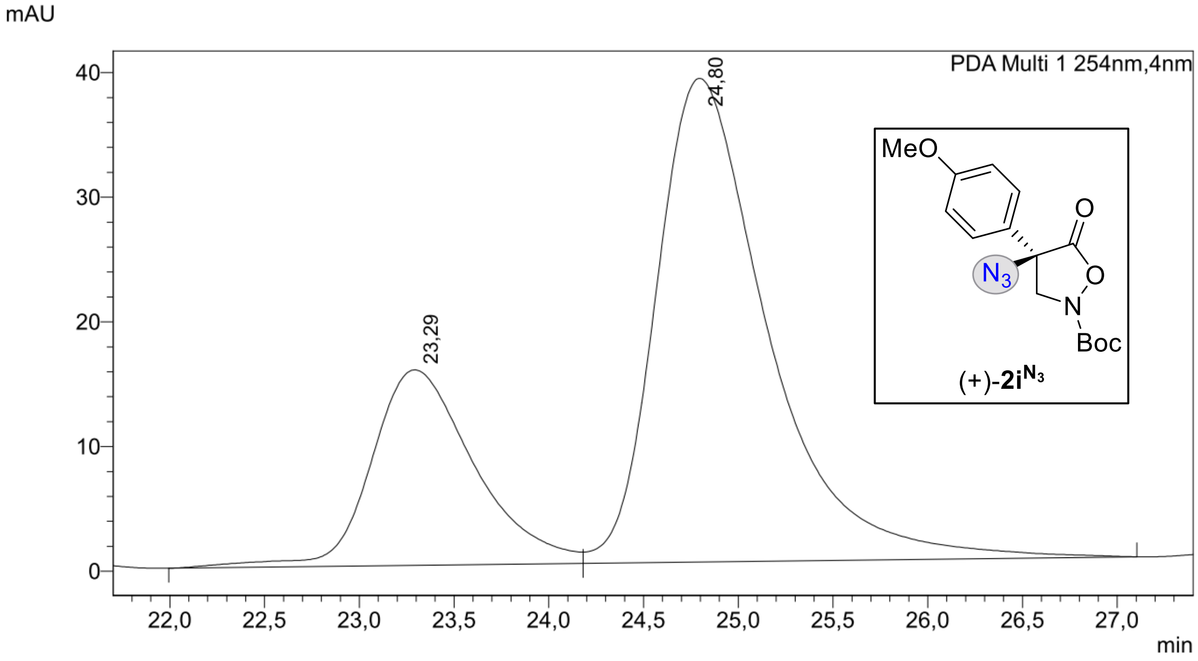

Peak Table

PDA Ch1 254nm

Peak#	Ret. Time	Area	Area%
1	23,29	595528	27
2	24,80	1594214	73
Total		2189742	100

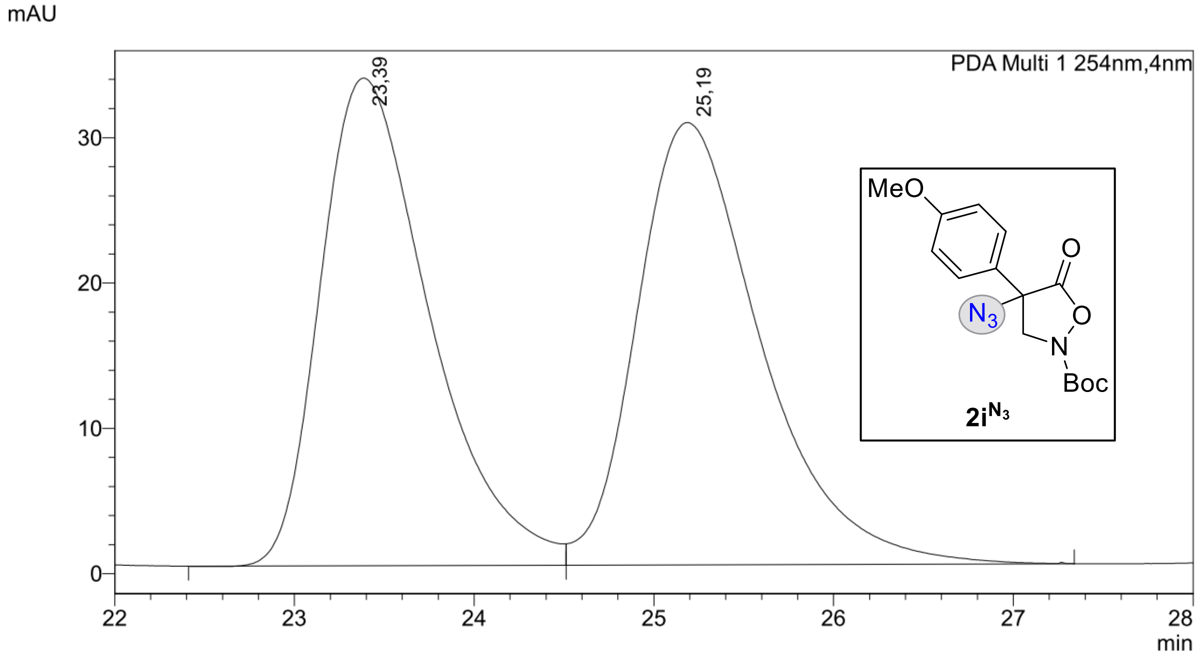

Peak Table

PDA Ch1 254nm

Peak#	Ret. Time	Area	Area%
1	23,39	1420204	49
2	25,19	1464222	51
Total		2884426	100

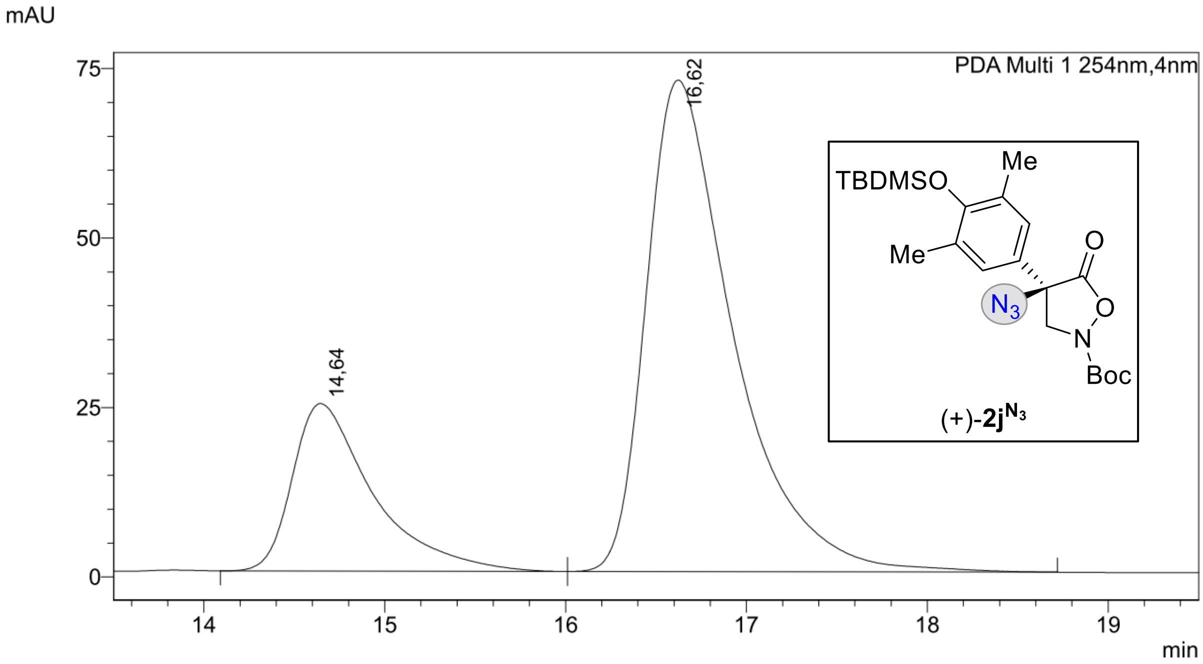

Peak Table

PDA Ch1 254nm

Peak#	Ret. Time	Area	Area%
1	14,64	765347	24
2	16,62	2435352	76
Total		3200699	100

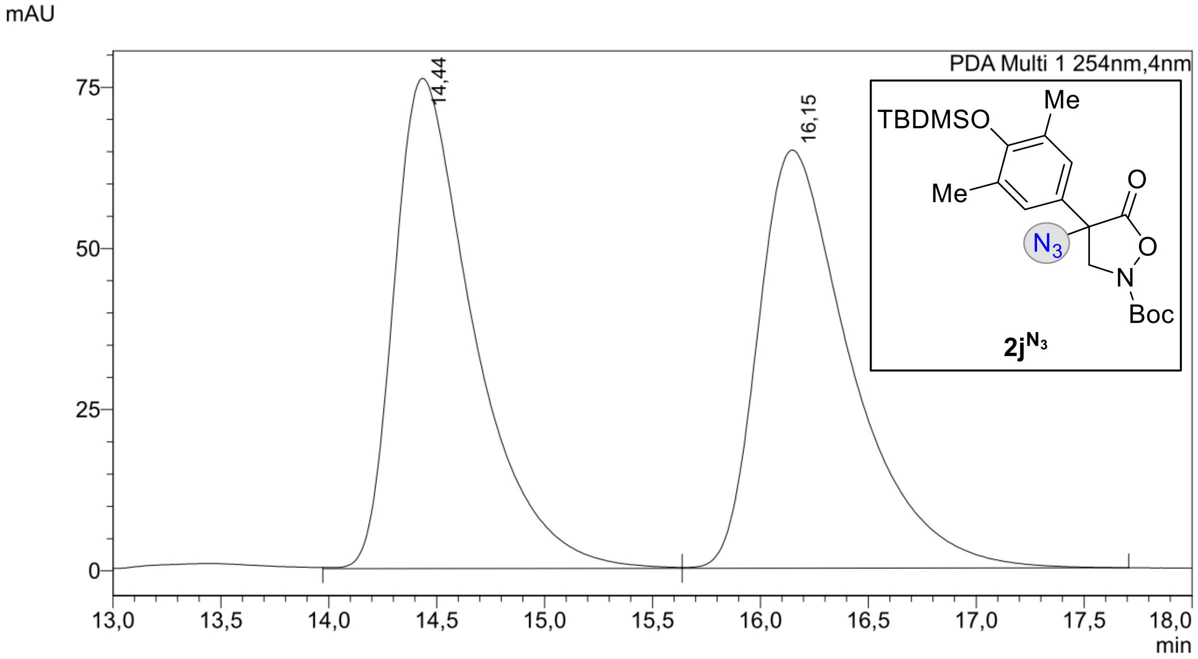

Peak Table

PDA Ch1 254nm

Peak#	Ret. Time	Area	Area%
1	14,44	1953166	50
2	16,15	1954464	50
Total		3907629	100

6. Computational methods.

A thorough potential energy surface scan on all reactants and catalysts was implemented to map the conformational and configurational space for the reactions studied. Grimme's CREST program was used for the conformational search. Two layer ONIOM approach was applied to model all the transition states owing to the size of the systems investigated (~800 electrons) in gas phase. Single point corrections were computed on these systems using polarized continuum model (IEFPCM)[1-3] to account for solvation effects. Thermal corrections were calculated using Grimme's quasi-rigid rotor harmonic oscillator approximation.[4] TS and geometry optimization on the high layer were computed at B3LYP/6-31G*[5,6] and PM7, a semi empirical method, was used to model the low layer in the ONIOM scheme. Single point energies were calculated at M06-2X/6-31+G(d, p)[6-8] for the high layer and UFF for the low layer using Gaussian 16.[9] Steric maps were generated using SambVca program. Distortion-interaction/activation-strain analysis was performed as described by Houk and Bickelhaupt.[10] The interaction energy was decomposd into electrostatic interactions as described by Wheeler and coworkers.[11] Grimme's DFTD3[12] code was used to evaluate the dispersion contributions. NCI plots were generated using JMol. Molecular graphics were generated using CYLview[13].

Besides the calculations for the most active catalyst **A1**, we also proceeded to model the **A2** catalyzed a-chlorination to further elucidate the electronic and steric effects of the CF_3 groups (figure CA and CB). Computed TSS for the major and minor enantiomers gave an e.r. of 91:9 (computed $\Delta\Delta G^{‡}$=1.4 kcal/mol) which is in good agreement with the experimental ee of 85:15 ($\Delta\Delta G^{‡}$=1.0 kcal/mol). Decomposing the TS energy revealed similar trends in electrostatic and steric interactions as that of **A1**. However, for **A2** the electrostatic stabilization of the major TS was lower (1 kcal/mol) than that observed in the **A1** catalyzed reaction. Moreover, the percentage of free volume in the cavity for **A2** was found to be similar for both the enantiomers (43%) This result further supports the assertion that the spirocyclic Maruoka's catalysts **A1** and **A2**, provide a unique chiral space that not only provides stabilizing electrostatic environment due to strong electron withdrawing groups like fluorine and CF3, but also a highly confined space due to the bulky arms.

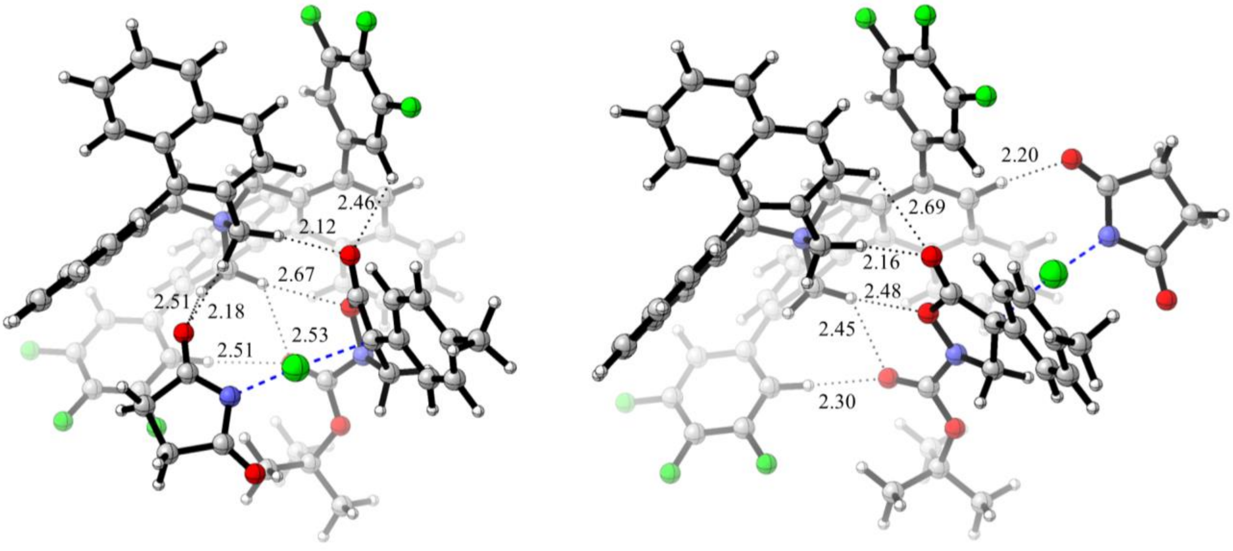

TS (S)$_{major}$
(0.0 kcal/mol)

TS (R)$_{minor}$
(1.4 kcal/mol; $e.r.$ = 91:9)

Figure CA A2 catalyzed α-chlorination computed at PCM(Toluene):UFF:M062X/6-31+G(d,p)//PM7:B3LYP/6-31G(d).

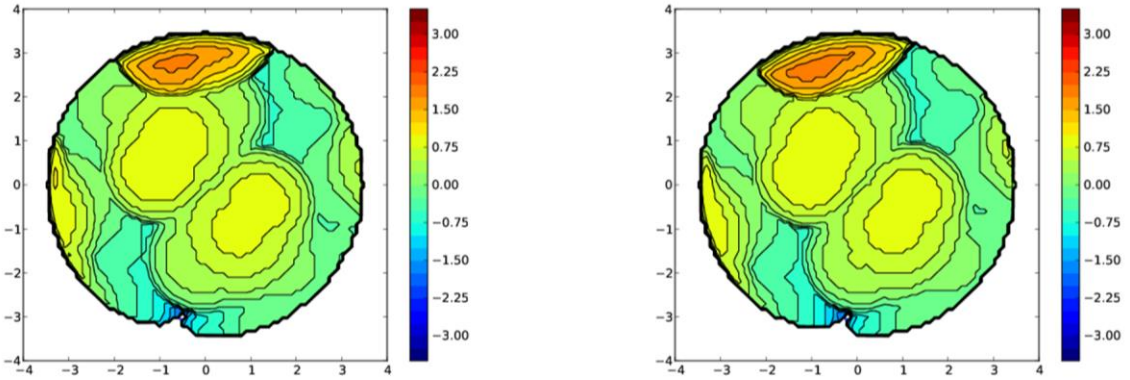

TS A3 (S)$_{major}$

TS A3 (R)$_{minor}$

Figure CB Steric cavity data generated for **A2** caltayzed α-chlorination.

Table: Computed single point energies and free energy corrections for the TS reported in this study.

File Name	Single Point Energy	Free Energy Correction	Free Energy
R_MinorCf03_ConstOpt.log:	-2866.52953325	1.181857	-2865.34767625
R_MinorCf04_ConstOpt.log:	-2866.53637413	1.183726	-2865.35264813
R_MinorCl_14_MaruokaCF3_TS_Diffuse.log:	-1972.01413537	1.101101	-1970.91303437
R_MinorCl_1_MaruokaF3_TS_Diffuse.log:	-1972.01527757	1.040157	-1970.97512057
R_MinorCl_2_MaruokaCF3_TS_Diffuse.log:	-1972.02590405	1.098970	-1970.92693405
R_MinorCl_2_MaruokaF3_TS_Diffuse.log:	-1972.02744680	1.038254	-1970.98919280
R_MinorCl_3_MaruokaCF3_TS_Diffuse.log:	-1972.03697077	1.097604	-1970.93936677
R_MinorCl_4_MaruokaCF3_TS_Diffuse.log:	-1972.01837531	1.099669	-1970.91870631
R_MinorCl_4_MaruokaF3_TS_Diffuse.log:	-1972.02377942	1.041143	-1970.98263642
S_MajorCf00_ConstOpt.log:	-2866.54119015	1.185715	-2865.35547515
S_MajorCf02_B3LYP_TS.log:	-2866.53660142	1.185400	-2865.35120142

File Name	Single Point Energy	Free Energy Correction	Free Energy
S_MajorCf04_B3LYP_TS.log:	-2866.54402484	1.183790	-2865.36023484
S_MajorCl_1_MaruokaCF3_TS_Diffuse.log:	-1972.04572422	1.101353	-1970.94437122
S_MajorCl_1_MaruokaF3_TS_Diffuse.log:	-1972.04585563	1.042476	-1971.00337963
S_MajorCl_2_MaruokaCF3_TS_Diffuse.log:	-1972.04258427	1.101022	-1970.94156227
S_MajorCl_2_MaruokaF3_TS_Diffuse.log:	-1972.04727588	1.041874	-1971.00540188
S_MajorCl_3_MaruokaCF3_TS_Diffuse.log:	-1972.01703622	1.077874	-1970.93916222
S_MajorCl_4_MaruokaCF3_TS_Diffuse.log:	-1972.04329667	1.100117	-1970.94317967
S_MajorCl_4_MaruokaF3_TS_Diffuse.log:	-1972.04776372	1.042493	-1971.00527072
S_MajorCl_5_MaruokaCF3_TS_Diffuse.log:	-1972.03940418	1.108666	-1970.93073818
S_MajorCl_5_MaruokaF3_TS_Diffuse.log:	-1972.03766651	1.046223	-1970.99144351
S_MajorCl_6_MaruokaCF3_TS_Diffuse.log:	-1972.04613032	1.107874	-1970.93825632

References (Computations):

(1) Miertus, S.; Scrocco, E.; Tomasi, J., Electrostatic Interaction of a Solute with a Continuum - a Direct Utilization of Abinitio Molecular Potentials for the Prevision of Solvent Effects. *Chem. Phys.* **1981**, *55*, 117-129.

(2) Tomasi, J.; Mennucci, B.; Cammi, R. Quantum mechanical continuum solvation models. *Chem. Rev*. **2005**, *105*, 2999-3093.

(3) Marenich, A. V.; Cramer, C. J.; Truhlar, D. G. Universal solvation model based on solute electron density and on a continuum model of the solvent defined by the bulk dielectric constant and atomic surface tensions. *J. Phys. Chem. B* **2009**, *113*, 6378-6396.

(4) Grimme, S. Supramolecular binding thermodynamics by dispersion-corrected density functional theory. *Chem. Eur. J.* **2012**, *18*, 9955-9964

(5) Becke, A. D. Density-functional thermochemistry. III. The role of exact exchange. *J. Chem. Phys*. **1993**, *98*, 5648-5652.

(6) Hehre, W. J.; Stewart, R. F.; Pople, J. A. Self-Consistent Molecular-Orbital Methods. I. Use of Gaussian Expansions of Slater-Type Atomic Orbitals. *J. Chem. Phys*. **1969**, *51*, 2657-2664.

(7) Zhao, Y.; Truhlar, D. G. The M06 suite of density functionals for main group thermochemistry, thermochemical kinetics, noncovalent interactions, excited states, and transition elements: two new functionals and systematic testing of four M06-class functionals and 12 other functionals. *Theor. Chem. Acc.* **2008**, *120*, 215-241.

(8) Wheeler, S. E.; Houk, K. N. Integration Grid Errors for Meta-GGA-Predicted Reaction Energies: Origin of Grid Errors for the M06 Suite of Functionals. *J. Chem. Theory Comput*. **2010**, *6*, 395-404.

(9) Frisch, M. J.; Trucks, G. W.; Schlegel, H. B.; Scuseria, G. E.; Robb, M. A.; Cheeseman, J. R.; Scalmani, G.; Barone, V.; Petersson, G. A.; Nakatsuji, H.; Li, X.; Caricato, M.; Marenich, A. V.; Bloino, J.; Janesko, B. G.; Gomperts, R.; Mennucci, B.; Hratchian, H. P.; Ortiz, J. V.; Izmaylov, A. F.; Sonnenberg, J. L.; Williams; Ding, F.; Lipparini, F.; Egidi, F.; Goings, J.; Peng, B.; Petrone, A.; Henderson, T.; Ranasinghe, D.; Zakrzewski, V. G.; Gao, J.; Rega, N.; Zheng, G.; Liang, W.; Hada, M.; Ehara, M.; Toyota, K.; Fukuda, R.; Hasegawa, J.; Ishida, M.; Nakajima, T.; Honda, Y.; Kitao, O.; Nakai, H.; Vreven, T.; Throssell, K.; Montgomery Jr., J. A.; Peralta, J. E.; Ogliaro, F.; Bearpark, M. J.; Heyd, J. J.; Brothers, E. N.; Kudin, K. N.; Staroverov, V. N.; Keith, T. A.; Kobayashi, R.; Normand, J.; Raghavachari, K.; Rendell, A. P.; Burant, J. C.; Iyengar, S. S.; Tomasi, J.; Cossi, M.; Millam, J. M.; Klene, M.; Adamo, C.; Cammi, R.; Ochterski, J. W.; Martin, R. L.; Morokuma, K.; Farkas, O.; Foresman, J. B.; Fox, D. J. Gaussian 16 Rev. C.01, Wallingford, CT, **2016**.

(10) Bickelhaupt, F. M.; Houk, K. N. Analyzing Reaction Rates with the Distortion/Interaction-Activation Strain Model. *Angew. Chem. Int. Ed.* **2017**, *56*, 10070-10086.

(11) Maji, R.; Wheeler, S. E. Importance of Electrostatic Effects in the Stereoselectivity of NHC-Catalyzed Kinetic Resolutions. *J Am. Chem. Soc.* **2017**, *139*, 12441-12449.

(12) Grimme, S.; Antony, J.; Ehrlich, S.; Krieg, H. A consistent and accurate ab initio parametrization of density functional dispersion correction (DFT-D) for the 94 elements H-Pu. *J. Chem. Phys.* **2010**, *132*, 154104.

(13) Legault, C. Y. CYLview, 1.0b; Université de Sherbrooke, **2009** (http://www.cylview.org).

Coordinates for the reported TS
177
R_MinorCf01_ConstOpt.log

C	-0.4362710	3.0656400	-2.5333580
C	-0.1793940	2.8811520	-3.8764880
C	-1.0967170	2.1902950	-4.7197220
C	-0.8113800	1.9886970	-6.1094540
C	-1.6837360	1.2874610	-6.9009530
C	-2.8773580	0.7413330	-6.3431710
C	-3.1779100	0.9306030	-5.0184960
C	-2.3041120	1.6868660	-4.1696240
C	-2.5765670	1.9280200	-2.7796240
C	-1.6517890	2.5683880	-1.9645000
C	-2.0114200	2.7766600	-0.5260940
H	-1.3295200	3.4586110	0.0017590
H	-3.0310310	3.1886590	-0.4463740
N	-2.0150260	1.4710980	0.3077230
C	-0.5609090	1.1688300	0.7936090
C	-0.1891160	2.1023390	1.9126750
C	-0.7489690	1.9606420	3.1742570
C	-0.4805130	2.9315730	4.1999140
C	-1.1335660	2.9070680	5.4745240
C	-0.8621900	3.8634670	6.4200410
C	0.0755920	4.9024940	6.1497040
C	0.7110100	4.9589610	4.9354860
C	0.4424740	3.9765090	3.9294820
C	1.0954110	4.0259410	2.6575980
C	0.7863980	3.1151110	1.6820160
H	1.3128310	3.1251740	0.7259370
H	1.8497940	4.7948300	2.4805700
H	1.4259220	5.7512010	4.7124670
H	0.2716570	5.6469940	6.9206110
H	-1.3600570	3.8465450	7.3890700
H	-1.8569160	2.1164600	5.6814210
C	-1.6828010	0.8373210	3.4095530
C	-2.7939600	0.7314350	2.5860680
C	-2.9904500	1.7169830	1.4732500
H	-4.0002560	1.6731980	1.0403370
H	-2.8071830	2.7452670	1.8263140
C	-3.7708890	-0.2772790	2.8224160
C	-3.5958120	-1.1878200	3.8319350
C	-2.4191680	-1.1558520	4.6455970
C	-2.2041070	-2.1313090	5.6712590
C	-1.0548280	-2.1104160	6.4199270
C	-0.0557040	-1.1227570	6.1760280
C	-0.2411760	-0.1724160	5.2047660
C	-1.4425760	-0.1479940	4.4262020
H	0.5403380	0.5712720	4.9927160
H	0.8567410	-1.1440010	6.7704100
H	-0.8823960	-2.8495200	7.2017510
H	-2.9669500	-2.8919160	5.8364640
H	-4.3425920	-1.9594870	4.0234190
H	-4.6609580	-0.3049770	2.1954950
H	0.0791690	1.2549700	-0.0942690
H	-0.5341480	0.1163980	1.1144280
C	-2.4635740	0.2157210	-0.4929750

C	-3.7719550	0.4551680	-1.1933940
C	-3.8384780	1.4119620	-2.1957960
C	-5.1095480	1.8641710	-2.6926560
C	-5.2374860	2.9877240	-3.5695250
C	-6.4750830	3.4051280	-3.9918690
C	-7.6513930	2.7204440	-3.5692180
C	-7.5590460	1.6347630	-2.7345480
C	-6.2811970	1.1826730	-2.2764710
C	-6.1551280	0.0328080	-1.4384170
C	-4.9362410	-0.3429610	-0.9148810
C	-4.8485000	-1.6310610	-0.1899190
C	-3.8535260	-2.5686870	-0.5290550
C	-3.8239110	-3.8118410	0.1121580
C	-4.7695110	-4.1330490	1.0963740
C	-5.7626060	-3.2018960	1.4231040
C	-5.8118840	-1.9599260	0.7740180
H	-6.6012860	-1.2436890	1.0152090
C	-6.8119500	-3.5304530	2.4473010
F	-7.9882050	-3.9113470	1.9208280
F	-6.5109840	-4.5169680	3.3075600
F	-7.1450340	-2.5090990	3.2597690
H	-4.7292970	-5.1031600	1.6009670
C	-2.7366300	-4.8048940	-0.2251990
F	-2.1956240	-4.7191490	-1.4421200
F	-3.1593600	-6.0910440	-0.1747110
F	-1.7080270	-4.7884180	0.6318460
H	-3.0817320	-2.3272740	-1.2876960
H	-7.0496600	-0.5642370	-1.2501300
H	-8.4513210	1.1014840	-2.4080620
H	-8.6199880	3.0741120	-3.9226220
H	-6.5777150	4.2628310	-4.6564610
H	-4.3340780	3.5063130	-3.8958260
H	-1.6696350	-0.0171220	-1.2183600
H	-2.5112510	-0.5856360	0.2562080
H	-4.0767450	0.4841590	-4.5898990
H	-3.5380100	0.1612750	-6.9866590
H	-1.4768270	1.1260800	-7.9582800
H	0.1164130	2.3917090	-6.5150530
H	0.7507520	3.2519080	-4.3147350
C	0.5779950	3.8228300	-1.7635340
C	0.2744790	5.0964120	-1.2734720
C	1.2715100	5.8553380	-0.6384010
C	2.5749510	5.3631950	-0.5364570
C	2.8743650	4.0841330	-1.0384310
C	1.8807180	3.2995470	-1.6300200
H	2.0774170	2.2610550	-1.9810560
C	4.2997610	3.6027890	-0.9231390
F	4.7643920	3.5576120	0.3330780
F	5.1709620	4.4135460	-1.5598930
F	4.5670240	2.3950700	-1.4291740
H	3.3653730	5.9617960	-0.0726220
C	0.9039550	7.2083250	-0.0973970
F	1.8345850	7.8293630	0.6445200
F	-0.1770110	7.2001750	0.7061230
F	0.5914040	8.1133110	-1.0403480
H	-0.7311840	5.5025380	-1.4029740

O	5.5494940	-0.3135130	-2.0133150
C	5.0324760	-0.5318210	-0.9452900
C	4.7872070	-1.8205110	-0.2330180
C	4.2718190	-1.4669010	1.1256840
H	5.0323060	-1.6461630	1.8977470
H	3.3572610	-2.0083210	1.3799020
N	3.9668890	-0.0322170	1.0111250
C	4.0884070	0.8549710	2.0754890
O	4.2306800	2.0539600	1.9597640
O	3.9528760	0.1438990	3.2180370
C	3.9895200	0.8029400	4.5367540
C	3.8116710	-0.3751140	5.4979950
H	4.6297450	-1.0937840	5.3848110
H	3.8065200	-0.0167920	6.5327080
H	2.8672730	-0.8928840	5.3003300
C	5.3511220	1.4759070	4.7393560
H	5.4184850	1.8677680	5.7604970
H	5.4903490	2.2987640	4.0363330
H	6.1594120	0.7498120	4.6001740
C	2.8308710	1.7918300	4.6634240
H	2.9264320	2.6001610	3.9358860
H	2.8206180	2.2255230	5.6699110
H	1.8660460	1.2837900	4.5028630
O	4.5457270	0.4937790	-0.1811990
C	5.5028380	-3.0547800	-0.5106440
C	6.4186000	-3.1626570	-1.5767140
C	7.0966290	-4.3552390	-1.8009550
C	6.8836310	-5.4820290	-0.9939560
C	5.9640770	-5.3731220	0.0600410
C	5.2860410	-4.1856710	0.3014220
H	4.5615390	-4.1434600	1.1088450
H	5.7728720	-6.2342520	0.6958450
C	7.5981170	-6.7821340	-1.2700880
H	7.0287900	-7.4020470	-1.9761030
H	8.5852960	-6.6119440	-1.7127430
H	7.7305200	-7.3707490	-0.3559830
H	7.8081700	-4.4141790	-2.6214820
H	6.5941790	-2.3031710	-2.2119530
C	1.4549530	-1.0500980	-6.5242310
C	2.8088360	-1.3866540	-6.5703840
C	3.5181940	-1.5995180	-5.3852820
C	2.8855630	-1.4730670	-4.1491390
C	1.5315270	-1.1308680	-4.1206460
C	0.8069410	-0.9223020	-5.2944690
H	-0.2476310	-0.6720540	-5.2331880
S	0.6811400	-0.8640680	-2.5607570
O	1.2312600	0.3819050	-1.9541090
O	-0.7738550	-0.7948060	-2.8683890
N	1.0930430	-2.2232400	-1.7657330
F	3.0613950	-1.9906600	-1.1074530
S	0.3439300	-2.6395020	-0.3790490
O	0.6566740	-1.7630570	0.7789840
O	-1.1150070	-2.8988830	-0.5642040
C	1.1562000	-4.2118170	-0.0754120
C	1.3738440	-4.5801850	1.2537200
C	1.8988620	-5.8427380	1.5376660

C	2.2024850	-6.7240360	0.4980700
C	1.9851010	-6.3413330	-0.8283520
C	1.4557130	-5.0851960	-1.1221120
H	1.2899350	-4.7697280	-2.1455590
H	2.2254180	-7.0246560	-1.6383350
H	2.6053720	-7.7090050	0.7202300
H	2.0626910	-6.1387520	2.5708710
H	1.1293880	-3.8804460	2.0466980
H	3.4115130	-1.6552800	-3.2202550
H	4.5715870	-1.8649310	-5.4197320
H	3.3110920	-1.4876880	-7.5294540
H	0.8982710	-0.8945410	-7.4446130

177
R_MinorCf03_ConstOpt.log

C	-2.5230790	-0.1414020	-2.5532610
C	-2.7783390	1.1744990	-2.8786230
C	-1.7409210	2.0363680	-3.3454620
C	-1.9966030	3.4159460	-3.6296870
C	-1.0019410	4.2132500	-4.1357580
C	0.3002240	3.6812800	-4.3676380
C	0.5782100	2.3688980	-4.0804170
C	-0.4420920	1.5068060	-3.5639020
C	-0.1976820	0.1346060	-3.2214070
C	-1.1764510	-0.6345800	-2.6101310
C	-0.8004210	-1.9795540	-2.0697910
H	-1.6588830	-2.5416000	-1.6745380
H	-0.3072360	-2.5897840	-2.8441770
N	0.1995560	-1.9091130	-0.8886140
C	-0.5359730	-1.4510910	0.4104010
C	-1.4416230	-2.5367990	0.9139610
C	-0.9296530	-3.6943310	1.4806050
C	-1.8148900	-4.7677170	1.8399220
C	-1.3364440	-6.0449990	2.2770640
C	-2.2142850	-7.0479040	2.6020690
C	-3.6209430	-6.8343950	2.5114340
C	-4.1099820	-5.6252570	2.0868270
C	-3.2165510	-4.5643520	1.7316670
C	-3.7142210	-3.3032400	1.2730220
C	-2.8500650	-2.3213760	0.8681410
H	-3.2060560	-1.3280290	0.5476050
H	-4.7919520	-3.1390810	1.2581450
H	-5.1830550	-5.4503970	2.0103120
H	-4.2938140	-7.6474150	2.7823440
H	-1.8524990	-8.0209460	2.9325570
H	-0.2596390	-6.2085600	2.3437000
C	0.5335740	-3.7891300	1.6796940
C	1.3679090	-3.5995120	0.5873030
C	0.7865610	-3.3310510	-0.7685510
H	1.5260570	-3.4227040	-1.5767430
H	-0.0426420	-4.0264390	-0.9744740
C	2.7795030	-3.7190660	0.7336520
C	3.3336470	-3.9747970	1.9603680
C	2.5048400	-4.1020200	3.1195250
C	3.0782290	-4.3209320	4.4128850
C	2.2768540	-4.4036270	5.5230720
C	0.8634920	-4.2655120	5.3993210

C	0.2886670	-4.0656920	4.1698050
C	1.0945780	-3.9964100	2.9872680
H	-0.7929020	-3.9431620	4.0792040
H	0.2510060	-4.3151680	6.2991580
H	2.7040140	-4.5670030	6.5120920
H	4.1608070	-4.4108900	4.4956970
H	4.4134150	-4.0736310	2.0753400
H	3.4093960	-3.6160590	-0.1465520
H	-1.0802530	-0.5297270	0.1614380
H	0.2585760	-1.2176720	1.1356280
C	1.3488300	-0.8791690	-1.0797960
C	1.9192860	-0.9155920	-2.4683060
C	1.1225770	-0.4733590	-3.5145660
C	1.5429910	-0.6156070	-4.8815700
C	0.6913770	-0.3039340	-5.9901670
C	1.1308440	-0.4751930	-7.2786110
C	2.4454910	-0.9634170	-7.5357460
C	3.2848520	-1.2705940	-6.4950480
C	2.8480250	-1.1051130	-5.1417940
C	3.7095520	-1.4032540	-4.0445020
C	3.2730460	-1.3060160	-2.7393910
C	4.2830750	-1.5287780	-1.6785140
C	4.5964610	-0.5019820	-0.7697520
C	5.6250410	-0.6905830	0.1601810
C	6.3373460	-1.8991870	0.2058370
C	6.0288040	-2.9113590	-0.7083290
C	5.0126090	-2.7252020	-1.6582850
H	4.7872410	-3.5096190	-2.3865310
C	6.7808190	-4.2143940	-0.7121970
F	7.6947980	-4.3800690	0.2559670
F	5.9925710	-5.2979310	-0.5889540
F	7.4678720	-4.4443450	-1.8439330
H	7.1293290	-2.0442250	0.9472020
C	5.9683360	0.4078590	1.1337780
F	7.2224130	0.3698060	1.6194800
F	5.2017300	0.4139540	2.2364320
F	5.8646470	1.6576310	0.6562830
H	4.0369390	0.4563310	-0.8181110
H	4.7414990	-1.6868320	-4.2636900
H	4.2923450	-1.6428950	-6.6782810
H	2.7668000	-1.0859300	-8.5702740
H	0.4841800	-0.2405810	-8.1243130
H	-0.3146070	0.0729330	-5.7936730
H	0.9167160	0.1039030	-0.8529180
H	2.0743980	-1.1198050	-0.2942740
H	1.5843550	1.9710130	-4.2243290
H	1.0717210	4.3397440	-4.7650050
H	-1.1873010	5.2663500	-4.3498000
H	-2.9867730	3.8223050	-3.4128080
H	-3.7884380	1.5860270	-2.7833070
C	-3.6752880	-1.0272620	-2.2686900
C	-3.8429590	-2.2028910	-3.0112180
C	-4.9685620	-3.0149050	-2.7990050
C	-5.9440780	-2.6454360	-1.8693150
C	-5.7764810	-1.4617310	-1.1320250
C	-4.6545850	-0.6509190	-1.3295690

H	-4.5025780	0.2832810	-0.7544770
C	-6.8431210	-1.0521680	-0.1506970
F	-6.4395930	-0.2761650	0.8648440
F	-7.4582060	-2.0768490	0.4709870
F	-7.8538370	-0.3674190	-0.7099450
H	-6.8338900	-3.2641640	-1.7201400
C	-5.0998220	-4.2761480	-3.6077010
F	-5.2542010	-4.0683240	-4.9265780
F	-6.1197650	-5.0882350	-3.2912110
F	-4.0237520	-5.0824100	-3.5362050
H	-3.1045310	-2.4740510	-3.7700500
O	1.8697630	-0.2175190	2.2472300
C	1.9901300	0.9571590	1.9533120
C	1.6810820	2.2005900	2.6604710
C	2.2368410	3.3255650	1.8408210
H	3.1825710	3.6998390	2.2680230
H	1.5274740	4.1458520	1.7179050
N	2.4602190	2.6790080	0.5458260
C	3.2376240	3.1280540	-0.4957920
O	3.6755090	2.4047030	-1.3769340
O	3.3586240	4.4637850	-0.3846790
C	3.9634830	5.2658270	-1.4670040
C	5.4348630	4.8783190	-1.6387400
H	5.5327020	3.8525800	-1.9969700
H	5.9111750	5.5536550	-2.3585580
H	5.9663170	4.9712510	-0.6846820
C	3.1546340	5.0858260	-2.7554440
H	3.5322360	5.7716670	-3.5220200
H	2.0986810	5.3158570	-2.5755490
H	3.2353380	4.0624560	-3.1264120
C	3.8322400	6.6927560	-0.9311560
H	2.7821060	6.9508030	-0.7628340
H	4.2560430	7.4029000	-1.6490980
H	4.3681990	6.7995340	0.0177430
O	2.5411790	1.2734770	0.7233320
C	1.3546580	2.3110820	4.0641750
C	1.3963450	1.1992120	4.9316520
C	1.1024250	1.3509780	6.2829160
C	0.7492130	2.5972950	6.8198710
C	0.6869810	3.6968190	5.9479710
C	0.9830090	3.5650010	4.5990020
H	0.8742130	4.4193670	3.9387100
H	0.3871730	4.6684840	6.3332900
C	0.4501210	2.7615130	8.2897830
H	1.2673310	3.2841530	8.8050590
H	-0.4583610	3.3538610	8.4489270
H	0.3156830	1.7937800	8.7835310
H	1.1514050	0.4832000	6.9372790
H	1.6650170	0.2258370	4.5359220
C	-5.1612370	1.9365750	4.3521300
C	-4.2010200	1.5164310	5.2747640
C	-2.8791800	1.3133380	4.8690820
C	-2.5122990	1.5180680	3.5397690
C	-3.4862680	1.9339270	2.6287460
C	-4.8069050	2.1508990	3.0198330
H	-5.5309530	2.4913930	2.2884820

S	-3.0537840	2.1089750	0.8947140
O	-2.7105710	0.7465430	0.4099430
O	-4.2169920	2.7056190	0.1966530
N	-1.6907220	3.0149200	0.7617210
F	-0.1499040	2.2512990	1.6378840
S	-1.7491210	4.6286510	1.1964010
O	-2.8491320	4.9246490	2.1270640
O	-0.3840410	5.0383890	1.5734750
C	-2.1044970	5.5172400	-0.3419980
C	-3.4184470	5.5968230	-0.8079280
C	-3.6822060	6.3373650	-1.9601940
C	-2.6480260	6.9996770	-2.6270590
C	-1.3393420	6.9141410	-2.1482330
C	-1.0611970	6.1689730	-1.0003070
H	-0.0557420	6.1021500	-0.5979270
H	-0.5353190	7.4401400	-2.6571710
H	-2.8647000	7.5895100	-3.5142660
H	-4.7023990	6.4126410	-2.3271610
H	-4.2096890	5.0780500	-0.2796900
H	-1.4876570	1.3943170	3.2075660
H	-2.1259850	1.0018990	5.5880880
H	-4.4815680	1.3539230	6.3127490
H	-6.1873560	2.1038660	4.6696690

177

R_MinorCf04_ConstOpt.log

C	-3.4771340	-0.6656170	2.1812060
C	-3.4676480	-1.8404090	2.9039850
C	-2.4148110	-2.1415500	3.8191180
C	-2.3736370	-3.3900650	4.5171250
C	-1.3609390	-3.6528570	5.4049850
C	-0.3377750	-2.6878250	5.6350240
C	-0.3562340	-1.4827020	4.9785450
C	-1.4073700	-1.1716560	4.0560790
C	-1.4611080	0.0679320	3.3303150
C	-2.3931350	0.2656350	2.3210920
C	-2.2744350	1.4821000	1.4518690
H	-3.1062710	1.5801620	0.7382810
H	-2.2263760	2.3993880	2.0616390
N	-1.0032810	1.4844100	0.5750870
C	-1.1561880	0.4412810	-0.5745710
C	-2.1755900	0.9123420	-1.5732070
C	-1.9109180	1.9763840	-2.4217990
C	-2.9496060	2.4992950	-3.2676950
C	-2.7835810	3.6948810	-4.0382380
C	-3.8037900	4.1694090	-4.8236360
C	-5.0480300	3.4771910	-4.8871470
C	-5.2404920	2.3333970	-4.1542030
C	-4.1959690	1.8221570	-3.3198340
C	-4.3851430	0.6363910	-2.5406850
C	-3.4049770	0.1984040	-1.6908570
H	-3.5150530	-0.7320950	-1.1211140
H	-5.3214860	0.0907740	-2.6448990
H	-6.1873000	1.7964230	-4.1932910
H	-5.8374400	3.8741210	-5.5243510
H	-3.6796440	5.0795630	-5.4092540
H	-1.8300980	4.2242660	-3.9886070

C	-0.5520860	2.5604020	-2.4106230
C	-0.0335630	3.0067920	-1.2030570
C	-0.8607120	2.9303000	0.0450370
H	-0.4390030	3.5149940	0.8746300
H	-1.8840880	3.2904940	-0.1484820
C	1.2644310	3.5894980	-1.1577100
C	2.0308090	3.6771510	-2.2908050
C	1.5442040	3.1773870	-3.5390070
C	2.3485760	3.2432780	-4.7220010
C	1.8820370	2.7364200	-5.9078470
C	0.5988080	2.1179260	-5.9679670
C	-0.1915280	2.0434270	-4.8492450
C	0.2487720	2.5988050	-3.6035100
H	-1.1571790	1.5358900	-4.8870600
H	0.2662240	1.6927870	-6.9138700
H	2.4844960	2.7845590	-6.8135600
H	3.3418620	3.6861380	-4.6517790
H	3.0328380	4.1064760	-2.2554720
H	1.6405160	3.9673980	-0.2116310
H	-1.4501510	-0.5007700	-0.0922490
H	-0.1663930	0.3188810	-1.0386880
C	0.2901450	1.0877130	1.3435690
C	0.3227040	1.6953700	2.7197540
C	-0.5353330	1.1727970	3.6809320
C	-0.5889050	1.7111680	5.0127360
C	-1.5616840	1.3039850	5.9842080
C	-1.5550130	1.8348620	7.2486460
C	-0.5785820	2.8053090	7.6224220
C	0.3525990	3.2311830	6.7111730
C	0.3556540	2.7021690	5.3794390
C	1.2824780	3.1679370	4.4047940
C	1.2573250	2.7121230	3.0997770
C	2.2357080	3.3528740	2.1894380
C	3.2476680	2.6319910	1.5357940
C	4.2437680	3.3283590	0.8345810
C	4.2178200	4.7258110	0.7384280
C	3.1844160	5.4357380	1.3609680
C	2.2029960	4.7549210	2.0928990
H	1.4082160	5.3119730	2.5970480
C	3.1055580	6.9350670	1.2777330
F	4.0235590	7.5423360	0.5108700
F	1.9369530	7.3936300	0.7938210
F	3.2237020	7.5536200	2.4659480
H	4.9958200	5.2573170	0.1818430
C	5.3500070	2.5498490	0.1674160
F	4.9769870	1.9110880	-0.9471380
F	5.9014310	1.6031570	0.9443330
F	6.4126390	3.2827780	-0.2185660
H	3.2506180	1.5227540	1.5551620
H	2.0249240	3.9077450	4.7157250
H	1.1013630	3.9761770	6.9791170
H	-0.5920020	3.1992940	8.6385700
H	-2.2939730	1.5249440	7.9875920
H	-2.3134720	0.5638540	5.7040830
H	0.2936600	-0.0086960	1.4157310
H	1.1257350	1.3798460	0.6968070

H	0.4491630	-0.7590710	5.1224250
H	0.4664910	-2.9309650	6.3287750
H	-1.3158870	-4.6024530	5.9379230
H	-3.1510030	-4.1266500	4.3185490
H	-4.2770260	-2.5659970	2.7969320
C	-4.6965180	-0.3686650	1.3943330
C	-5.3928840	0.8275380	1.6167380
C	-6.6018370	1.0722950	0.9488810
C	-7.1542380	0.1056000	0.1029730
C	-6.4713380	-1.1044730	-0.0921820
C	-5.2324890	-1.3317480	0.5191350
H	-4.6542300	-2.2503130	0.3205760
C	-7.0775890	-2.1838520	-0.9501620
F	-6.4972330	-2.3281600	-2.1528050
F	-8.3799750	-2.0343830	-1.2452030
F	-7.0215720	-3.4134650	-0.4109700
H	-8.1102950	0.2886290	-0.3966740
C	-7.2993410	2.3847470	1.1888970
F	-6.5041470	3.4603350	1.0449020
F	-7.7960650	2.5165570	2.4305650
F	-8.3447210	2.6583700	0.3940460
H	-4.9981580	1.5587480	2.3278470
O	1.1502050	-1.9727940	2.6080360
C	0.7463790	-2.8023300	1.8212910
C	1.3468760	-4.0323860	1.2961340
C	0.3720530	-4.6550570	0.3421910
H	0.0985730	-5.6783030	0.6262770
H	0.7476210	-4.6420250	-0.6850390
N	-0.8102100	-3.7886630	0.4784730
C	-1.7653350	-3.5651900	-0.4884970
O	-2.5507600	-2.6263950	-0.4731510
O	-1.7392700	-4.6006840	-1.3381800
C	-2.6369610	-4.6729380	-2.5122290
C	-4.0677850	-4.8992990	-2.0199820
H	-4.7423640	-5.0061980	-2.8780790
H	-4.1317760	-5.8150020	-1.4218690
H	-4.4034210	-4.0556920	-1.4173010
C	-2.4932660	-3.4177070	-3.3757030
H	-1.4362910	-3.2202560	-3.5771240
H	-2.9243660	-2.5418340	-2.8880550
H	-3.0107930	-3.5782810	-4.3290970
C	-2.1003590	-5.8997740	-3.2515910
H	-2.1360670	-6.7871790	-2.6110360
H	-1.0644690	-5.7328440	-3.5624040
H	-2.7063720	-6.0919310	-4.1436530
O	-0.5135850	-2.6447650	1.2674050
C	2.4456210	-4.7444010	1.9061920
C	3.1249300	-4.2418770	3.0380610
C	4.1610770	-4.9649950	3.6174640
C	4.5743370	-6.2015980	3.0988250
C	3.9012910	-6.6975860	1.9719500
C	2.8605730	-5.9896920	1.3860660
H	2.3711790	-6.3981120	0.5069100
H	4.2012630	-7.6531900	1.5478070
C	5.7233050	-6.9624520	3.7136800
H	5.8647640	-6.6958280	4.7661470

H	5.5665740	-8.0451020	3.6539250
H	6.6655560	-6.7444570	3.1923550
H	4.6615060	-4.5627390	4.4955340
H	2.8221710	-3.2875490	3.4518360
C	3.3614990	-0.5928300	-6.2869470
C	3.6368820	-1.8054690	-6.9228770
C	3.3088990	-3.0132400	-6.3020440
C	2.7060050	-3.0118850	-5.0444470
C	2.4390360	-1.7920060	-4.4195220
C	2.7599980	-0.5777310	-5.0282070
H	2.5482240	0.3546180	-4.5160350
S	1.6573210	-1.7989820	-2.7950780
O	0.8669110	-0.5536470	-2.6968950
O	0.9391710	-3.0843330	-2.6915190
N	2.9077630	-1.8760560	-1.7136050
F	2.1652450	-2.7484340	-0.1355920
S	3.6631600	-0.4908280	-1.1980820
O	4.0415380	0.3947880	-2.3211870
O	2.9851750	0.1655140	-0.0494740
C	5.1649130	-1.2344430	-0.5495820
C	6.3743010	-0.9707900	-1.1912710
C	7.5509590	-1.5097820	-0.6666920
C	7.5069040	-2.3029120	0.4806650
C	6.2845840	-2.5639590	1.1072830
C	5.1035660	-2.0282430	0.5975330
H	4.1438710	-2.2375920	1.0524510
H	6.2464120	-3.1908150	1.9939140
H	8.4251210	-2.7206450	0.8863550
H	8.5006210	-1.3073120	-1.1553080
H	6.3833500	-0.3535410	-2.0832170
H	2.4312390	-3.9370630	-4.5499740
H	3.5187300	-3.9569760	-6.7983950
H	4.1064740	-1.8100270	-7.9031230
H	3.6156790	0.3464530	-6.7701760

159
R_MinorCl_14_MaruokaCF3_TS_Diffuse.log

C	-1.2271100	-0.5574250	2.2847740
C	-2.0878990	-1.6203630	2.1345390
C	-1.6028830	-2.9318080	1.8414530
C	-2.4983300	-3.9948170	1.5115370
C	-2.0147180	-5.2609230	1.2899580
C	-0.6204600	-5.5288620	1.4107490
C	0.2626410	-4.5193350	1.7118470
C	-0.2060980	-3.1820520	1.8990640
C	0.6791980	-2.0617820	2.0725220
C	0.1814680	-0.7681710	2.0874620
C	1.1257150	0.3797950	1.9274080
H	0.6188950	1.3552560	1.9797060
H	1.9271190	0.3551850	2.6829870
N	1.8418990	0.3879520	0.5561460
C	0.8256820	0.6925430	-0.5819590
C	0.3798280	2.1227290	-0.5202710
C	1.2410550	3.1494390	-0.8824640
C	0.8312350	4.5169580	-0.7226030
C	1.7240720	5.6181700	-0.9290560
C	1.2924460	6.9096990	-0.7641500

C	-0.0553380	7.1795180	-0.3841170
C	-0.9312200	6.1467120	-0.1680670
C	-0.5048750	4.7883500	-0.3223370
C	-1.4027840	3.7033020	-0.0767950
C	-0.9666960	2.4064610	-0.1549140
H	-1.6563020	1.5752780	0.0221650
H	-2.4487760	3.9151740	0.1683040
H	-1.9627150	6.3371730	0.1302310
H	-0.3695280	8.2161150	-0.2655390
H	1.9705910	7.7487980	-0.9177500
H	2.7565970	5.4120040	-1.2151870
C	2.5782220	2.7925300	-1.4099160
C	3.3972490	1.9700630	-0.6486660
C	2.9296060	1.4726660	0.6875570
H	3.7357050	1.0205250	1.2812590
H	2.4861430	2.2985200	1.2663630
C	4.7027220	1.6295710	-1.1057920
C	5.1497330	2.0692210	-2.3239100
C	4.3037550	2.8588680	-3.1655960
C	4.7451700	3.2820190	-4.4597120
C	3.9127260	4.0055100	-5.2755150
C	2.5960260	4.3381950	-4.8426300
C	2.1531360	3.9539540	-3.6022420
C	3.0034130	3.2151550	-2.7172240
H	1.1390040	4.1978770	-3.2780800
H	1.9480740	4.8972770	-5.5177600
H	4.2384660	4.3294380	-6.2641810
H	5.7501030	3.0124000	-4.7836990
H	6.1520360	1.8191850	-2.6745410
H	5.3446280	1.0281970	-0.4654110
H	0.0259990	-0.0527130	-0.4991040
H	1.3595950	0.4776270	-1.5191380
C	2.4923030	-0.9745550	0.1470510
C	3.0314620	-1.7290190	1.3265230
C	2.1383860	-2.2605960	2.2474220
C	2.6047440	-2.9499540	3.4212070
C	1.7192500	-3.4273540	4.4410480
C	2.2095880	-4.0701450	5.5497420
C	3.6115490	-4.2742260	5.7071990
C	4.4862800	-3.8260000	4.7500900
C	3.9988540	-3.1511040	3.5856820
C	4.8936760	-2.6856820	2.5763820
C	4.4323020	-2.0027810	1.4718860
C	5.4300020	-1.6415300	0.4379560
C	5.3355740	-2.1893600	-0.8516190
C	6.3084290	-1.8827040	-1.8118190
C	7.3926000	-1.0567650	-1.4893930
C	7.4952610	-0.5341310	-0.1931620
C	6.5175240	-0.8189440	0.7692380
H	6.5892820	-0.4091830	1.7811500
C	8.6527450	0.3638760	0.1597130
F	9.0190290	0.3486380	1.4517170
F	9.7969980	0.1154850	-0.4963020
F	8.4065570	1.6618360	-0.0931550
H	8.1556060	-0.8236660	-2.2394030
C	6.1704890	-2.4802230	-3.1899840

F	6.3578590	-3.8092420	-3.2174340
F	7.0162690	-2.0117650	-4.1215750
F	4.9598850	-2.3028120	-3.7425180
H	4.5046160	-2.8644130	-1.0918120
H	5.9581740	-2.9014310	2.6917950
H	5.5600560	-3.9755660	4.8585810
H	3.9704750	-4.7902970	6.5981710
H	1.5357880	-4.4353920	6.3256770
H	0.6435020	-3.2743170	4.3229140
H	1.6990470	-1.5427640	-0.3919790
H	3.2734080	-0.6829320	-0.5710220
H	1.3306810	-4.7214480	1.7872530
H	-0.2668010	-6.5452840	1.2371350
H	-2.6845540	-6.0717350	1.0000970
H	-3.5633080	-3.7739500	1.3971550
H	-3.1713620	-1.4915240	2.2616030
C	-1.7570090	0.7231940	2.8037290
C	-1.1335410	1.3168870	3.9108240
C	-1.6708810	2.4782440	4.4871330
C	-2.8624090	3.0244700	4.0042470
C	-3.5037460	2.4117690	2.9154900
C	-2.9439360	1.2875010	2.3007490
H	-3.4374050	0.8219090	1.4300420
C	-4.8123120	2.9787540	2.4220990
F	-4.6839470	3.9817220	1.5397960
F	-5.5828490	3.5128470	3.3914010
F	-5.6391520	2.1067610	1.8288880
H	-3.2927990	3.9196620	4.4625130
C	-0.9241350	3.0943810	5.6387740
F	-1.4738060	4.1719360	6.2174450
F	0.3131920	3.5109650	5.3020970
F	-0.7071950	2.2563170	6.6674010
H	-0.2411340	0.8554650	4.3419550
O	-5.0375520	-1.7396170	1.2575580
C	-5.0728200	-1.1609210	0.2001440
C	-5.3293730	-1.6595470	-1.2204530
C	-5.5504550	-0.3432760	-1.9738220
H	-6.6129640	-0.0593340	-1.9473590
H	-5.2010910	-0.3711270	-3.0054490
N	-4.7292340	0.5838640	-1.2077540
C	-4.6531490	1.9623390	-1.3695920
O	-4.2680200	2.7240510	-0.5036670
O	-5.0151730	2.2447110	-2.6331460
C	-4.8501830	3.6049860	-3.1967070
C	-5.3261030	3.4189440	-4.6382750
H	-6.3652750	3.0757880	-4.6625960
H	-5.2641510	4.3706120	-5.1771480
H	-4.7055890	2.6839620	-5.1609490
C	-5.7512110	4.5846970	-2.4409650
H	-5.7141130	5.5664080	-2.9273040
H	-5.4346590	4.6935700	-1.4023550
H	-6.7901870	4.2379640	-2.4585640
C	-3.3748480	4.0048160	-3.1544130
H	-3.0246030	4.1163170	-2.1267770
H	-3.2381320	4.9587080	-3.6766640
H	-2.7592260	3.2515720	-3.6589950

O	-4.7807880	0.1881300	0.1653820
C	-6.4074210	-2.6958650	-1.4179930
C	-6.8777740	-3.5066600	-0.3768640
C	-7.8683280	-4.4573720	-0.6169540
C	-8.4131360	-4.6428820	-1.8929700
C	-7.9326240	-3.8335370	-2.9303480
C	-6.9458240	-2.8796230	-2.6999700
H	-6.5806820	-2.2876380	-3.5349120
H	-8.3317650	-3.9552850	-3.9351540
C	-9.4583710	-5.7018560	-2.1496110
H	-8.9935740	-6.6708960	-2.3761800
H	-10.1047780	-5.8489750	-1.2772450
H	-10.0946460	-5.4409380	-3.0024290
H	-8.2249580	-5.0687450	0.2094390
H	-6.4740290	-3.3876040	0.6203220
C	0.9318300	-4.0765060	-2.3207190
C	0.1752480	-5.1032030	-3.1564800
H	1.3174890	-4.4765460	-1.3749210
H	1.7818350	-3.6099640	-2.8362610
H	0.5479680	-5.1944460	-4.1832390
H	0.1618860	-6.1113930	-2.7306120
N	-1.3354800	-3.3079870	-2.5546350
Cl	-3.2519260	-2.5097250	-1.8815190
C	-1.2653370	-4.5304730	-3.1939640
O	-2.1921810	-5.1108840	-3.7417470
C	-0.1438950	-3.0062240	-2.0227910
O	0.1371390	-1.9864560	-1.3476060

147

R_MinorCl_1_MaruokaF3_TS_Diffuse.log

C	1.4487530	2.8067630	0.9001760
C	2.2690400	3.3246750	-0.0843720
C	1.7686350	3.7999100	-1.3192860
C	2.6432780	4.2283020	-2.3529770
C	2.1354880	4.7189460	-3.5327680
C	0.7360780	4.7995100	-3.7253070
C	-0.1360830	4.3893020	-2.7417750
C	0.3526430	3.8765890	-1.5087850
C	-0.5040350	3.3791570	-0.4707080
C	0.0316830	2.7446210	0.6485640
C	-0.9177280	1.9961720	1.5541780
H	-0.4156300	1.5915470	2.4314680
H	-1.7434590	2.6305170	1.8861960
N	-1.6005330	0.7748610	0.8980240
C	-0.5991320	-0.3837370	0.6911430
C	-0.2315930	-1.1016880	1.9628310
C	-1.1452490	-1.9525630	2.5823490
C	-0.8041770	-2.5635080	3.8395570
C	-1.7355480	-3.3089110	4.6169700
C	-1.3702830	-3.8694840	5.8201160
C	-0.0530080	-3.7210840	6.3156480
C	0.8676940	-2.9899090	5.6030050
C	0.5185170	-2.3834940	4.3660870
C	1.4477830	-1.5888760	3.6485670
C	1.0730720	-0.9491410	2.4932900
H	1.7871240	-0.3328620	1.9609780
H	2.4585770	-1.4795040	4.0329380

H	1.8782150	-2.8539000	5.9799710
H	0.2231830	-4.1762370	7.2625640
H	-2.1017080	-4.4288400	6.3967140
H	-2.7509350	-3.4280820	4.2579170
C	-2.4752850	-2.1607730	1.9464620
C	-3.2539320	-1.0466340	1.6331000
C	-2.7217160	0.3446110	1.8654600
H	-3.5055430	1.0968770	1.7543680
H	-2.2885520	0.4366200	2.8637720
C	-4.5739360	-1.2126610	1.1437450
C	-5.0917580	-2.4652130	0.9221580
C	-4.2924460	-3.6207620	1.1118780
C	-4.7796260	-4.9172760	0.7934920
C	-3.9701210	-6.0229880	0.9155310
C	-2.6322230	-5.8755660	1.3545000
C	-2.1398690	-4.6372170	1.6990520
C	-2.9549790	-3.4735330	1.6101440
H	-1.1075700	-4.5413960	2.0133750
H	-1.9825650	-6.7439640	1.4044060
H	-4.3475240	-7.0084000	0.6572520
H	-5.8002720	-5.0146050	0.4319580
H	-6.1118060	-2.5821160	0.5654840
H	-5.1838760	-0.3349650	0.9618010
H	0.2694550	0.0664330	0.2109800
H	-1.0634050	-1.0511390	-0.0356930
C	-2.1730420	1.0835470	-0.5042550
C	-2.8202880	2.4399170	-0.6384590
C	-1.9849590	3.5544790	-0.5769480
C	-2.5284800	4.8840290	-0.5728670
C	-1.7420300	6.0516700	-0.3617240
C	-2.3194660	7.3016390	-0.3578950
C	-3.7104830	7.4562020	-0.5710860
C	-4.5012600	6.3486060	-0.7657870
C	-3.9366750	5.0440220	-0.7571270
C	-4.7383010	3.8948780	-0.9380800
C	-4.2296750	2.6095480	-0.8737420
C	-5.1985310	1.4970080	-1.1049950
C	-4.9622690	0.4614850	-2.0270550
C	-5.9496570	-0.4912620	-2.2449680
C	-7.1719710	-0.4545420	-1.5780340
C	-7.4017710	0.5782080	-0.6738440
C	-6.4355900	1.5444280	-0.4337410
H	-6.6531460	2.3227850	0.2894110
H	-4.0287610	0.3483940	-2.5715650
H	-5.7933670	4.0356170	-1.1549960
H	-5.5722750	6.4518850	-0.9210260
H	-4.1495390	8.4498100	-0.5732410
H	-1.7013950	8.1788640	-0.1890120
H	-0.6760150	5.9529440	-0.1952250
H	-1.3366000	0.9736270	-1.1945220
H	-2.8446790	0.2582070	-0.7161440
H	-1.2047880	4.4313740	-2.9181750
H	0.3449100	5.1704200	-4.6685260
H	2.8078410	5.0217590	-4.3298040
H	3.7138040	4.1088240	-2.2161920
H	3.3359120	3.3979640	0.0976930

C	2.0569330	2.4830030	2.2226190
C	1.4593540	2.9641020	3.4018690
C	2.0411290	2.7214820	4.6381360
C	3.2305220	2.0081510	4.7449690
C	3.8303330	1.5512330	3.5737250
C	3.2726950	1.7777620	2.3220950
H	3.7950930	1.3804090	1.4552440
H	0.5604940	3.5703590	3.3718820
O	2.7365490	-0.1611490	-4.3993940
C	2.9981710	-0.1816390	-3.2233500
C	3.1635080	-1.2706040	-2.2424450
C	3.3371840	-0.5515450	-0.9116030
H	4.0766090	-0.9912380	-0.2450040
H	2.3809970	-0.4348610	-0.3810990
N	3.7659440	0.8070360	-1.2984530
C	5.0920750	1.2434500	-1.2680850
O	5.5486920	2.0908730	-2.0096700
O	5.7192030	0.6744510	-0.2094390
C	7.1480130	0.9525230	0.0589720
C	7.4454720	0.0390830	1.2505800
H	6.7917200	0.2748790	2.0949320
H	7.2969150	-1.0110790	0.9784910
H	8.4856620	0.1692570	1.5683250
C	7.9941530	0.5527700	-1.1546450
H	7.7963510	1.2006630	-2.0101860
H	7.7829210	-0.4835970	-1.4391650
H	9.0566300	0.6247580	-0.8948110
C	7.3247420	2.4258530	0.4389950
H	8.3670710	2.6075030	0.7256420
H	6.6902390	2.6788290	1.2969570
H	7.0721450	3.0789740	-0.3985480
O	3.1499550	1.0669760	-2.5532080
C	4.0171230	-2.4340220	-2.5826590
C	4.3771840	-2.7304970	-3.9145990
C	5.1770850	-3.8284370	-4.2143310
C	5.6520730	-4.6908420	-3.2144540
C	5.2833750	-4.4071980	-1.8955710
C	4.4819570	-3.3081980	-1.5841000
H	4.1951770	-3.1494860	-0.5472900
H	5.6197900	-5.0624470	-1.0938070
C	6.5122440	-5.8860480	-3.5603300
H	6.9581380	-6.3271630	-2.6593450
H	5.9266790	-6.6757240	-4.0611900
H	7.3334400	-5.6128460	-4.2438360
H	5.4388890	-4.0212650	-5.2541730
H	4.0192870	-2.0905350	-4.7135400
C	-2.3049380	-4.7085130	-2.6566660
C	-2.9181380	-3.3470470	-3.0140640
H	-2.8635420	-5.2433840	-1.8781800
H	-2.1974410	-5.3903920	-3.5097670
H	-3.0438260	-3.1988310	-4.0979650
H	-3.8923380	-3.1456980	-2.5528340
N	-0.7669480	-2.9816550	-2.0604220
Cl	1.0420640	-2.1536430	-2.1198360
C	-1.8839030	-2.3289800	-2.5132580
O	-2.0591680	-1.1069460	-2.5162830

C	-0.9038570	-4.3712240	-2.1118040
O	-0.0713690	-5.1877680	-1.7639820
F	1.4685480	3.1877430	5.7587180
F	3.7861850	1.7705620	5.9383060
F	4.9739700	0.8534570	3.6931980
F	-8.5764050	0.6159860	-0.0249270
F	-8.0968440	-1.3985060	-1.7894190
F	-5.7485450	-1.4902330	-3.1175870

159
R_MinorCl_2_MaruokaCF3_TS_Diffuse.log

C	-1.9560060	0.2135520	2.1069370
C	-2.7417460	-0.9205020	2.1857640
C	-2.1843980	-2.1994670	2.4216720
C	-3.0051070	-3.3599440	2.4394970
C	-2.4611270	-4.5977890	2.6882290
C	-1.0708070	-4.7259440	2.9213830
C	-0.2495470	-3.6206350	2.9090280
C	-0.7810810	-2.3223950	2.6693980
C	0.0246680	-1.1369540	2.6136140
C	-0.5338520	0.0843530	2.2463170
C	0.4034270	1.2433630	2.0252080
H	-0.1246770	2.1797480	1.8514420
H	1.0682840	1.3751530	2.8821910
N	1.3570740	1.1059070	0.8114960
C	0.6386070	1.4331630	-0.5175240
C	0.3560920	2.8987520	-0.7254180
C	1.3875690	3.8014910	-0.9876050
C	1.0872730	5.2055130	-1.0798000
C	2.0937540	6.2102330	-1.1532400
C	1.7631640	7.5445160	-1.2280980
C	0.4079030	7.9524470	-1.2398170
C	-0.5906090	7.0115830	-1.1504430
C	-0.2844160	5.6270560	-1.0513960
C	-1.3078150	4.6551570	-0.9169960
C	-0.9940720	3.3311700	-0.7310660
H	-1.7778450	2.5903480	-0.6371330
H	-2.3482600	4.9645490	-0.9483360
H	-1.6352820	7.3118350	-1.1402790
H	0.1615180	9.0083760	-1.3070320
H	2.5503220	8.2920490	-1.2737500
H	3.1374840	5.9180020	-1.1362300
C	2.7759790	3.2830850	-1.1385230
C	3.3118350	2.4791820	-0.1327490
C	2.4851540	2.1205090	1.0719170
H	3.0968840	1.6812940	1.8625710
H	1.9824850	3.0039810	1.4709780
C	4.6616220	2.0536590	-0.1963230
C	5.4569440	2.3740670	-1.2680380
C	4.9218090	3.0931990	-2.3665970
C	5.7047870	3.3592590	-3.5223260
C	5.1644350	4.0093770	-4.6065730
C	3.8089020	4.4151560	-4.5812780
C	3.0292880	4.1914530	-3.4689950
C	3.5596230	3.5430560	-2.3176740
H	1.9905170	4.4989790	-3.4725880
H	3.3770230	4.9022200	-5.4509180

H	5.7694380	4.1997980	-5.4885380
H	6.7386040	3.0233270	-3.5365730
H	6.4958460	2.0586820	-1.2899320
H	5.0741700	1.4846900	0.6288900
H	-0.2817550	0.8474010	-0.5310230
H	1.3025510	1.0455230	-1.2928900
C	1.9073470	-0.3287010	0.6381780
C	2.4127660	-0.9548820	1.9164440
C	1.4839880	-1.2280930	2.9173040
C	1.9203340	-1.6057490	4.2325800
C	1.0302890	-1.7365620	5.3345250
C	1.4999240	-2.0713250	6.5851700
C	2.8795430	-2.3042890	6.7999100
C	3.7678160	-2.1830470	5.7567890
C	3.3158980	-1.8212930	4.4591490
C	4.2099050	-1.7069470	3.3689230
C	3.7975210	-1.2901980	2.1164870
C	4.7971530	-1.3433890	1.0119630
C	4.5041550	-1.9761670	-0.2073740
C	5.4797570	-2.0788860	-1.2025620
C	6.7596230	-1.5643110	-1.0060670
C	7.0602570	-0.9456780	0.2074090
C	6.0936220	-0.8405100	1.2063220
H	6.3448180	-0.3521510	2.1424340
C	8.4083530	-0.3055890	0.4063650
F	9.3699750	-0.9163630	-0.3127870
F	8.3936860	0.9979210	0.0187670
F	8.7867690	-0.3197200	1.7012460
H	7.5097680	-1.6463010	-1.7831560
C	5.1514260	-2.8082540	-2.4815580
F	3.9163670	-2.4932640	-2.9256970
F	5.1776490	-4.1488150	-2.3028820
F	6.0272290	-2.5189560	-3.4664850
H	3.5215390	-2.4110500	-0.3731770
H	5.2463880	-1.9963270	3.5205820
H	4.8301520	-2.3555640	5.9104940
H	3.2334450	-2.5744080	7.7907970
H	0.8051390	-2.1569470	7.4157410
H	-0.0283100	-1.5593660	5.1840740
H	1.0976720	-0.9167390	0.1997310
H	2.6936030	-0.2230360	-0.1071680
H	0.8158630	-3.7440350	3.0636780
H	-0.6442190	-5.7090910	3.1022490
H	-3.0932640	-5.4811720	2.6885160
H	-4.0627380	-3.2490150	2.2231930
H	-3.8198420	-0.8293010	2.0725740
C	-2.6547110	1.5247060	1.9893320
C	-2.4057530	2.5573110	2.9028810
C	-3.1085040	3.7630310	2.8327930
C	-4.0730650	3.9571950	1.8478910
C	-4.3318760	2.9276120	0.9399730
C	-3.6455880	1.7175850	1.0119310
H	-3.8595080	0.9288000	0.3014990
C	-5.2884280	3.1844030	-0.1941960
F	-4.6701030	3.8902470	-1.1856820
F	-6.3449750	3.9299250	0.1996270

F	-5.7557750	2.0572700	-0.7479590
H	-4.6157780	4.8934290	1.7870740
C	-2.8546280	4.8238210	3.8691840
F	-1.5434930	4.8817220	4.2084940
F	-3.5390760	4.5870540	5.0109830
F	-3.2148200	6.0514970	3.4378810
H	-1.6768760	2.4156690	3.6952420
O	-2.1786700	0.2989270	-1.4994370
C	-2.1170810	-0.9121460	-1.6656150
C	-2.9434790	-1.8600920	-2.3775570
C	-2.1754500	-3.1661950	-2.3862570
H	-1.6588020	-3.3429010	-3.3431100
H	-2.7879210	-4.0409310	-2.1558570
N	-1.1942610	-2.9664250	-1.2989540
C	0.0032670	-3.6393000	-1.2020080
O	1.0176350	-3.1927700	-0.6828750
O	-0.1766760	-4.8721200	-1.7146940
C	0.8692080	-5.9087610	-1.6578640
C	2.0365480	-5.4954190	-2.5563120
H	2.7966370	-6.2844940	-2.5674720
H	1.6892020	-5.3423280	-3.5838780
H	2.4998460	-4.5743170	-2.2042820
C	1.2977650	-6.1576020	-0.2083610
H	0.4213620	-6.3616110	0.4159080
H	1.8264680	-5.2972060	0.2035430
H	1.9572900	-7.0319250	-0.1702690
C	0.1483250	-7.1339660	-2.2255220
H	-0.2151240	-6.9327670	-3.2381560
H	-0.7077750	-7.4046930	-1.5995720
H	0.8344230	-7.9865550	-2.2662170
O	-1.0606110	-1.5749270	-1.0333430
C	-3.7229310	-1.4627480	-3.5659630
C	-4.2068180	-0.1498890	-3.7408920
C	-4.9478520	0.1919280	-4.8659190
C	-5.2524890	-0.7467650	-5.8634840
C	-4.7886310	-2.0521860	-5.6808010
C	-4.0412770	-2.4062550	-4.5575160
H	-3.7207380	-3.4388520	-4.4501340
H	-5.0181790	-2.8115820	-6.4257440
C	-6.0490010	-0.3543800	-7.0859330
H	-5.4678900	0.2921810	-7.7577180
H	-6.3560050	-1.2339080	-7.6617380
H	-6.9555360	0.2006030	-6.8148150
H	-5.3066920	1.2146030	-4.9690860
H	-4.0023690	0.5970970	-2.9832790
C	-8.2330390	-3.3056160	1.1046070
C	-7.9699150	-1.9572550	1.7854930
H	-8.3449670	-4.1367580	1.8090070
H	-9.1165340	-3.3119970	0.4584560
H	-8.6722830	-1.1719650	1.4856320
H	-7.9838340	-1.9976730	2.8794720
N	-6.0881760	-2.5169120	0.4562280
Cl	-4.5390350	-2.1859480	-0.7896750
C	-6.5626540	-1.5550770	1.3149220
O	-5.9729900	-0.5437590	1.6757750
C	-6.9854350	-3.5544540	0.2362970

```
O       -6.8194510       -4.5057200       -0.5003800
147
R_MinorCl_2_MaruokaF3_TS_Diffuse.log
C        1.5935660        0.4046890       -2.4035460
C        2.6438180       -0.4903480       -2.3134410
C        2.4348900       -1.8882730       -2.2570130
C        3.5288310       -2.7825040       -2.1009700
C        3.3218320       -4.1409440       -2.0602970
C        2.0095160       -4.6606660       -2.1684350
C        0.9296470       -3.8201420       -2.3214170
C        1.1090330       -2.4093180       -2.3791450
C        0.0250730       -1.4780090       -2.4958470
C        0.2511710       -0.1063660       -2.4163090
C       -0.9619700        0.7868710       -2.3476200
H       -0.7092070        1.8436910       -2.4129130
H       -1.6660960        0.5481230       -3.1481630
N       -1.7945710        0.6666700       -1.0456290
C       -1.1459420        1.4689440        0.1036190
C       -1.2934600        2.9627240       -0.0389950
C       -2.5285950        3.5826460        0.1538040
C       -2.6575280        4.9935710       -0.0935160
C       -3.9109020        5.6696410       -0.0945930
C       -3.9908410        7.0196780       -0.3517430
C       -2.8232030        7.7736690       -0.6191470
C       -1.5977900        7.1508920       -0.6467180
C       -1.4814390        5.7553010       -0.4031390
C       -0.2269590        5.0981540       -0.4731900
C       -0.1383210        3.7362850       -0.3175260
H        0.8216680        3.2368760       -0.3718460
H        0.6656960        5.6852180       -0.6721920
H       -0.6951300        7.7143740       -0.8688590
H       -2.9005000        8.8396620       -0.8137070
H       -4.9602390        7.5103420       -0.3544530
H       -4.8171640        5.1072520        0.0986110
C       -3.6893040        2.7468720        0.5709340
C       -4.0120260        1.6207980       -0.1856800
C       -3.1782940        1.2486490       -1.3829310
H       -3.6721650        0.4939430       -1.9981500
H       -2.9760300        2.1251810       -2.0017710
C       -5.1562010        0.8473360        0.1298600
C       -5.9422440        1.1566780        1.2118760
C       -5.5951630        2.2311600        2.0695170
C       -6.3515040        2.5140060        3.2389320
C       -5.9766850        3.5227720        4.0945930
C       -4.8190670        4.2884630        3.8177820
C       -4.0754580        4.0524530        2.6836760
C       -4.4451240        3.0327170        1.7612850
H       -3.1854100        4.6405200        2.4945130
H       -4.5099110        5.0655460        4.5110210
H       -6.5569980        3.7248220        4.9904220
H       -7.2260060        1.9041130        3.4513630
H       -6.8263350        0.5651510        1.4352480
H       -5.4151130        0.0062530       -0.5029070
H       -0.0963160        1.1734020        0.1468890
H       -1.6366530        1.1072730        1.0091940
C       -1.9079590       -0.7883790       -0.5346860
```

C	-2.2816260	-1.7822460	-1.6099810
C	-1.3677070	-1.9981500	-2.6374410
C	-1.7487980	-2.7329750	-3.8099360
C	-0.9108320	-2.8519180	-4.9531130
C	-1.3338290	-3.5389420	-6.0693090
C	-2.6093880	-4.1522300	-6.0983820
C	-3.4445380	-4.0558470	-5.0095460
C	-3.0433120	-3.3400630	-3.8495030
C	-3.8729350	-3.2534760	-2.7065270
C	-3.5239220	-2.5088300	-1.5947230
C	-4.3834930	-2.6227970	-0.3829750
C	-3.8078770	-2.8888510	0.8722130
C	-4.6272860	-3.0719310	1.9774420
C	-6.0153950	-3.0067930	1.8780610
C	-6.5774150	-2.7559860	0.6286030
C	-5.7837080	-2.5688120	-0.4944840
H	-6.2682150	-2.3667520	-1.4438890
H	-2.7335610	-2.9887190	1.0003710
H	-4.7922010	-3.8328010	-2.6918340
H	-4.4265710	-4.5222410	-5.0216310
H	-2.9263450	-4.6962120	-6.9837200
H	-0.6831020	-3.6112660	-6.9360930
H	0.0676670	-2.3853880	-4.9423890
H	-0.9463430	-1.0285370	-0.0774450
H	-2.6551380	-0.7383550	0.2549080
H	-0.0690570	-4.2369570	-2.3777280
H	1.8508890	-5.7347980	-2.1197500
H	4.1616210	-4.8170550	-1.9273360
H	4.5238680	-2.3658850	-1.9853590
H	3.6653520	-0.1175390	-2.2904340
C	1.9340210	1.8449180	-2.5775320
C	1.3565440	2.6002270	-3.6135860
C	1.7340610	3.9220540	-3.8044340
C	2.6929510	4.5201840	-2.9933790
C	3.2828000	3.7576300	-1.9858240
C	2.9174440	2.4370670	-1.7663900
H	3.3946430	1.8849150	-0.9654540
H	0.6466080	2.1646530	-4.3088510
O	1.8275290	1.2428760	1.1303790
C	2.0596210	0.1253730	1.5714710
C	3.0869180	-0.4084980	2.4355590
C	2.6678940	-1.8249970	2.7751970
H	2.2314190	-1.9012130	3.7832310
H	3.4743580	-2.5574540	2.6905840
N	1.6466870	-2.1151550	1.7463460
C	0.6349590	-3.0344380	1.8949890
O	-0.4554170	-2.9692830	1.3423610
O	1.0849220	-4.0240050	2.6902500
C	0.3081890	-5.2534250	2.9300510
C	-0.9448610	-4.9176930	3.7437040
H	-1.4653080	-5.8428860	4.0160750
H	-0.6678230	-4.4008070	4.6688790
H	-1.6358420	-4.2854820	3.1846340
C	-0.0145970	-5.9473530	1.6029990
H	0.9019950	-6.1030800	1.0239740
H	-0.7100860	-5.3583000	1.0039350

H	-0.4622750	-6.9269450	1.8049540
C	1.2814440	-6.0971730	3.7576880
H	1.5575640	-5.5750180	4.6792060
H	2.1949860	-6.3014920	3.1906330
H	0.8161250	-7.0515550	4.0256120
O	1.1990820	-0.8995190	1.1590110
C	3.7387770	0.4165220	3.4710770
C	3.7677070	1.8241150	3.4099870
C	4.3952820	2.5681840	4.4030580
C	5.0305860	1.9568480	5.4938540
C	5.0086970	0.5600540	5.5502530
C	4.3803450	-0.1961750	4.5618720
H	4.4077630	-1.2797350	4.6377810
H	5.4953980	0.0496380	6.3790830
C	5.7339480	2.7785020	6.5488750
H	5.1369700	3.6488280	6.8474330
H	5.9366330	2.1864950	7.4478780
H	6.6976710	3.1609800	6.1856810
H	4.3936020	3.6543970	4.3279840
H	3.2856510	2.3268130	2.5800660
C	8.5842970	-1.4233460	-0.8719580
C	8.0231050	-0.3904270	-1.8563170
H	8.8725200	-2.3671000	-1.3472110
H	9.4518290	-1.0721300	-0.3045400
H	8.5264920	0.5810220	-1.8025620
H	8.0508600	-0.7071700	-2.9038370
N	6.3193090	-0.9760520	-0.3165720
Cl	4.7337520	-0.7096750	0.8745480
C	6.5593640	-0.2012330	-1.4248320
O	5.7538210	0.5183320	-2.0025020
C	7.4283700	-1.6953500	0.1090780
O	7.4847190	-2.4269870	1.0770130
F	-4.0911370	-3.3371920	3.1794130
F	-6.7907790	-3.1813020	2.9541700
F	-7.9153530	-2.6826220	0.5330810
F	1.1826730	4.6492450	-4.7927650
F	3.0404290	5.8022660	-3.1761210
F	4.2060610	4.3471450	-1.2131480

159
R_MinorCl_3_MaruokaCF3_TS_Diffuse.log

C	-3.2547100	2.2464610	0.8722590
C	-3.2313150	2.8843140	2.0968350
C	-2.1437070	3.6891570	2.5084750
C	-2.1093420	4.2883160	3.7960680
C	-1.0478330	5.0765420	4.1742030
C	0.0273610	5.2936380	3.2793960
C	0.0166610	4.7386380	2.0192840
C	-1.0735570	3.9318380	1.5905510
C	-1.1271040	3.3108630	0.2988200
C	-2.1315580	2.3981050	-0.0069460
C	-1.9989670	1.6068520	-1.2837340
H	-2.8711230	0.9823080	-1.4737710
H	-1.8550680	2.2712890	-2.1394440
N	-0.8036440	0.6318140	-1.3294000
C	-1.0712260	-0.6532300	-0.4971310
C	-2.0704740	-1.5866570	-1.1307990

C	-1.7414800	-2.3138250	-2.2753950
C	-2.7495610	-3.1165250	-2.9152170
C	-2.5546840	-3.7375200	-4.1817780
C	-3.5491500	-4.4882450	-4.7672480
C	-4.7941640	-4.6672620	-4.1183200
C	-5.0245280	-4.0674750	-2.9026640
C	-4.0266270	-3.2697910	-2.2797240
C	-4.2675440	-2.6184330	-1.0437270
C	-3.3254870	-1.7805810	-0.4990240
H	-3.5013920	-1.3031210	0.4575190
H	-5.2123740	-2.7794250	-0.5329270
H	-5.9815830	-4.1846910	-2.4002110
H	-5.5671270	-5.2695490	-4.5876320
H	-3.3778190	-4.9451170	-5.7380620
H	-1.6094830	-3.6058850	-4.6956860
C	-0.3580990	-2.2163370	-2.8189450
C	0.1703330	-0.9583720	-3.0995050
C	-0.6379960	0.2781040	-2.8217160
H	-0.1911440	1.1624630	-3.2813840
H	-1.6568960	0.1675610	-3.1990250
C	1.4434090	-0.8328270	-3.7057730
C	2.2068930	-1.9385070	-3.9824960
C	1.7575340	-3.2304140	-3.6116940
C	2.5717770	-4.3771500	-3.8126060
C	2.1537830	-5.6206420	-3.4013360
C	0.8994740	-5.7666320	-2.7622030
C	0.0772870	-4.6787760	-2.5724010
C	0.4655060	-3.3798460	-3.0062360
H	-0.8718970	-4.8074680	-2.0662470
H	0.5853860	-6.7452300	-2.4102100
H	2.7894740	-6.4890930	-3.5497320
H	3.5436090	-4.2467660	-4.2819750
H	3.1737330	-1.8267880	-4.4638090
H	1.8093770	0.1528400	-3.9691200
H	-1.3996310	-0.3235430	0.4914740
H	-0.0907260	-1.1249000	-0.3954600
C	0.5012990	1.2313270	-0.7493120
C	0.7751060	2.6544030	-1.1756240
C	-0.0955770	3.6438800	-0.7292810
C	-0.0271390	4.9776070	-1.2575510
C	-0.9804140	5.9882830	-0.9499900
C	-0.8892730	7.2413860	-1.5140870
C	0.1618920	7.5560230	-2.4080740
C	1.0979350	6.6011490	-2.7300990
C	1.0225600	5.2951480	-2.1757370
C	1.9909600	4.3076150	-2.4738020
C	1.9052060	3.0169480	-1.9873540
C	3.0805250	2.1241820	-2.2123640
C	3.7708690	1.5747480	-1.1195040
C	4.9415540	0.8411180	-1.3263980
C	5.4390110	0.6384890	-2.6136700
C	4.7617010	1.1924380	-3.6990840
C	3.5993990	1.9390740	-3.5006080
H	3.0936410	2.3804460	-4.3542000
C	5.2419240	0.9367190	-5.1014650
F	6.5549180	0.6378580	-5.1458260

F	4.5794620	-0.1073400	-5.6680010
F	5.0341910	2.0024460	-5.9057100
H	6.3516590	0.0757010	-2.7668840
C	5.6775400	0.2541770	-0.1448960
F	6.9731090	0.0067220	-0.4418750
F	5.1362590	-0.9148210	0.2599120
F	5.6604540	1.0875690	0.9190900
H	3.4185430	1.7537010	-0.1076440
H	2.8542370	4.5934800	-3.0690520
H	1.9085020	6.8293280	-3.4176660
H	0.2235810	8.5506050	-2.8405890
H	-1.6326070	7.9957980	-1.2722690
H	-1.7949840	5.7623120	-0.2716200
H	0.3990310	1.1564930	0.3331360
H	1.2814660	0.5312230	-1.0415930
H	0.8609350	4.8898000	1.3570890
H	0.8748740	5.8956810	3.5940560
H	-1.0246730	5.5237720	5.1639320
H	-2.9330530	4.1012700	4.4801460
H	-4.0853660	2.7875680	2.7614660
C	-4.5144390	1.5512330	0.4822810
C	-5.1971250	1.9043860	-0.6910830
C	-6.4264020	1.3223440	-1.0042720
C	-7.0022230	0.3824960	-0.1495210
C	-6.3284080	0.0315870	1.0197960
C	-5.0977420	0.6075960	1.3385200
H	-4.5423170	0.2836270	2.2131850
C	-6.8816740	-1.0389360	1.9223050
F	-6.2460100	-2.2261410	1.7250890
F	-8.1954410	-1.2575370	1.7130090
F	-6.7181480	-0.7297880	3.2259370
H	-7.9560530	-0.0694990	-0.3933910
C	-7.1652180	1.7709030	-2.2380420
F	-7.9035500	2.8788090	-2.0003040
F	-8.0091020	0.8213220	-2.6924800
F	-6.3130940	2.0767860	-3.2431780
H	-4.7805630	2.6551160	-1.3556000
O	1.9134640	-1.6883720	-0.0164850
C	2.2745500	-1.3618480	1.1055210
C	2.6071630	-2.0915000	2.3062640
C	3.2814400	-1.0925150	3.2248100
H	4.3800420	-1.1302910	3.1443170
H	3.0001300	-1.1920910	4.2755040
N	2.7608350	0.1852320	2.7122550
C	3.3061140	1.4293080	2.9252890
O	3.1429260	2.3882720	2.1854430
O	3.9736730	1.4077920	4.0970440
C	4.5790200	2.6304650	4.6515980
C	5.6480360	3.1659650	3.6929130
H	5.2000880	3.5359140	2.7697850
H	6.1965920	3.9827830	4.1757340
H	6.3622860	2.3747560	3.4417380
C	3.4855720	3.6619230	4.9471010
H	3.9271110	4.5326940	5.4453660
H	2.7290950	3.2337640	5.6136000
H	2.9985440	3.9899180	4.0271180

C	5.2156740	2.1242070	5.9482050
H	4.4571030	1.6907770	6.6076520
H	5.7036720	2.9512340	6.4747120
H	5.9668620	1.3568650	5.7354790
O	2.3549480	0.0126460	1.3596830
C	3.0608620	-3.4912110	2.2785790
C	2.7721490	-4.3537600	1.2018890
C	3.2131710	-5.6722040	1.2081460
C	3.9488400	-6.1997970	2.2793840
C	4.2316530	-5.3449170	3.3492780
C	3.7974520	-4.0204740	3.3528700
H	4.0286500	-3.3970830	4.2122960
H	4.7993100	-5.7196360	4.1988130
C	4.3922530	-7.6441220	2.2867850
H	5.2052430	-7.8100370	3.0019660
H	3.5699020	-8.3167400	2.5673100
H	4.7440500	-7.9632200	1.2982910
H	2.9793630	-6.3084170	0.3560250
H	2.2100190	-3.9752500	0.3565300
C	-3.6735100	-1.9483020	4.4049870
C	-3.0989600	-2.9306340	5.4305400
H	-4.4541590	-2.3760410	3.7682840
H	-4.0900900	-1.0394820	4.8530400
H	-3.1957360	-2.5946750	6.4677630
H	-3.5394660	-3.9312560	5.3738110
N	-1.3647070	-2.2206810	3.9664000
Cl	0.4708020	-2.1205500	3.1217570
C	-1.6042850	-3.0286470	5.0752520
O	-0.7829410	-3.6960670	5.6670740
C	-2.4719210	-1.5644170	3.5257380
O	-2.5404930	-0.7779810	2.5771710

159

R_MinorCl_4_MaruokaCF3_TS_Diffuse.log

C	-1.3641810	0.1920330	2.4658120
C	-2.2857310	-0.8366120	2.5229750
C	-1.8897840	-2.1956800	2.5936860
C	-2.8552470	-3.2385920	2.5925680
C	-2.4624800	-4.5515080	2.7137760
C	-1.0912500	-4.8749550	2.8411920
C	-0.1322550	-3.8860340	2.8442230
C	-0.5012460	-2.5180270	2.7209820
C	0.4510710	-1.4491930	2.6525210
C	0.0330240	-0.1446460	2.4126090
C	1.0875480	0.8756930	2.0759750
H	0.6777440	1.8801150	1.9820560
H	1.8858340	0.8951030	2.8219620
N	1.8175800	0.6301290	0.7322730
C	0.9207360	0.9833930	-0.4779440
C	0.7119200	2.4614450	-0.6673900
C	1.7545520	3.2787680	-1.1091320
C	1.5548760	4.7017720	-1.1688730
C	2.6100620	5.6223530	-1.4287260
C	2.3785760	6.9789190	-1.4610470
C	1.0788490	7.4948280	-1.2409600
C	0.0393330	6.6374500	-0.9684060
C	0.2464530	5.2321930	-0.9088600

C	-0.8141030	4.3465230	-0.5918070
C	-0.5806030	2.9996670	-0.4513010
H	-1.4075060	2.3366590	-0.2255810
H	-1.8150990	4.7371610	-0.4369040
H	-0.9604920	7.0204110	-0.7808410
H	0.9097900	8.5673800	-1.2763930
H	3.2025790	7.6608090	-1.6522020
H	3.6142230	5.2471660	-1.5898360
C	3.0558110	2.6514690	-1.4777090
C	3.6913470	1.8196380	-0.5563650
C	3.0526470	1.5472650	0.7778870
H	3.7485280	1.0629110	1.4663060
H	2.7035490	2.4759510	1.2338980
C	4.9722580	1.2819890	-0.8354740
C	5.5969640	1.5263680	-2.0329730
C	4.9423740	2.2766270	-3.0424950
C	5.5379180	2.4689640	-4.3183700
C	4.8756910	3.1523400	-5.3106840
C	3.5800990	3.6675310	-5.0679030
C	2.9833750	3.5163410	-3.8366520
C	3.6471090	2.8358180	-2.7767100
H	1.9867250	3.9081990	-3.6722520
H	3.0486180	4.1823990	-5.8631940
H	5.3368280	3.2861900	-6.2851180
H	6.5250040	2.0504520	-4.4981570
H	6.5905280	1.1294230	-2.2197940
H	5.4742850	0.6899320	-0.0784500
H	-0.0110630	0.4367060	-0.3282620
H	1.4264580	0.5361130	-1.3354410
C	2.2183850	-0.8487040	0.5143070
C	2.7706530	-1.5369270	1.7370320
C	1.9087760	-1.7394530	2.8110090
C	2.4133560	-2.2102410	4.0706150
C	1.6093720	-2.2992920	5.2407450
C	2.1456680	-2.7284870	6.4344250
C	3.5088150	-3.0995100	6.5218870
C	4.3153580	-3.0220770	5.4103440
C	3.7952810	-2.5690030	4.1683110
C	4.6040390	-2.5006730	3.0087090
C	4.1261740	-2.0105880	1.8082940
C	5.0076920	-2.1282250	0.6118250
C	4.5669360	-2.7794760	-0.5497790
C	5.4184360	-2.9411080	-1.6457060
C	6.7264810	-2.4659040	-1.6060080
C	7.1778300	-1.8293210	-0.4485800
C	6.3337360	-1.6687380	0.6496390
H	6.7026360	-1.1709110	1.5408420
C	8.5540650	-1.2188820	-0.4227600
F	9.0771920	-1.2047850	0.8200740
F	9.4136470	-1.8722520	-1.2277840
F	8.5171390	0.0726130	-0.8471360
H	7.3840660	-2.5939450	-2.4569690
C	4.8871410	-3.6455400	-2.8696630
F	4.4672650	-4.8933270	-2.5725830
F	5.8121770	-3.7434120	-3.8436740
F	3.8220470	-2.9856470	-3.3838230

H	3.5617050	-3.1892440	-0.6065130
H	5.6190820	-2.8859480	3.0620470
H	5.3644410	-3.3016500	5.4663260
H	3.9152400	-3.4415580	7.4694040
H	1.5167320	-2.7822220	7.3183750
H	0.5643650	-2.0169870	5.1881190
H	1.3091960	-1.3372660	0.1422340
H	2.9418540	-0.8079240	-0.2993510
H	0.9154370	-4.1543230	2.9225500
H	-0.7900690	-5.9158300	2.9244450
H	-3.2038420	-5.3448720	2.6935930
H	-3.8979030	-2.9719000	2.4562300
H	-3.3492630	-0.6177450	2.5326950
C	-1.8408630	1.5957360	2.6369650
C	-1.2140960	2.4299170	3.5754200
C	-1.6746070	3.7262600	3.8101830
C	-2.7854480	4.2109540	3.1253030
C	-3.4285620	3.3827870	2.2027040
C	-2.9694900	2.0876040	1.9564310
H	-3.4726680	1.4722450	1.2184910
C	-4.5976450	3.9451140	1.4302830
F	-4.1802060	4.6613990	0.3510570
F	-5.3152330	4.8055180	2.1944820
F	-5.4341820	2.9972320	0.9907770
H	-3.1501700	5.2141710	3.3110560
C	-1.0032230	4.5660410	4.8632160
F	-1.2171440	5.8847840	4.6728640
F	0.3380140	4.3692100	4.8727020
F	-1.4467600	4.2654580	6.1047360
H	-0.3734620	2.0573910	4.1526000
O	-5.2994900	-1.4957580	1.3630040
C	-5.2141960	-1.1908560	0.1862570
C	-5.5396480	-1.8715100	-1.0576760
C	-5.5321810	-0.7915410	-2.1241020
H	-6.5376010	-0.3773110	-2.3112610
H	-5.1008060	-1.1085080	-3.0759670
N	-4.6519600	0.2014730	-1.5144970
C	-4.2621850	1.4227470	-1.9692070
O	-3.6573720	2.2527620	-1.3037180
O	-4.6150110	1.5276590	-3.2726210
C	-4.2605480	2.7187050	-4.0598930
C	-4.8409600	2.3872870	-5.4374050
H	-5.9213680	2.2240710	-5.3715070
H	-4.6564040	3.2139250	-6.1317480
H	-4.3795990	1.4816020	-5.8441330
C	-4.9440260	3.9555340	-3.4654560
H	-4.7475100	4.8258350	-4.1023070
H	-4.5807900	4.1637490	-2.4579030
H	-6.0283320	3.8044080	-3.4233470
C	-2.7375980	2.8689590	-4.1374940
H	-2.3133700	3.1052020	-3.1607690
H	-2.4847410	3.6743270	-4.8368650
H	-2.2845350	1.9422780	-4.5068620
O	-4.6602990	0.0631810	-0.1066460
C	-6.5397310	-2.9512280	-1.1276770
C	-6.9587240	-3.6735920	0.0064520

```
C        -7.9024530       -4.6898900       -0.1037910
C        -8.4672920       -5.0416540       -1.3374260
C        -8.0517070       -4.3238810       -2.4641220
C        -7.1088970       -3.3032540       -2.3650280
H        -6.8042400       -2.7829540       -3.2690510
H        -8.4697230       -4.5678420       -3.4389090
C        -9.4635820       -6.1719470       -1.4486110
H        -8.9601040       -7.1447040       -1.5370270
H       -10.1122730       -6.2248490       -0.5665300
H       -10.1033720       -6.0577900       -2.3306930
H        -8.2109390       -5.2229890        0.7939970
H        -6.5499480       -3.4168440        0.9762760
C         0.6464220       -3.9671240       -1.6772680
C        -0.0322670       -5.2060480       -2.2685820
H         1.1812570       -4.1594700       -0.7408260
H         1.3524940       -3.4890210       -2.3643760
H         0.3048700       -5.4558910       -3.2792530
H         0.0881540       -6.1061190       -1.6571170
N        -1.6867620       -3.5606110       -1.7790120
Cl        -3.4744410       -2.7834900       -1.3835210
C        -1.5264670       -4.8354540       -2.3113270
O        -2.4078870       -5.5499280       -2.7399780
C        -0.5148230       -3.0005290       -1.3798010
O        -0.3651660       -1.8943080       -0.8554470
147
R_MinorCl_4_MaruokaF3_TS_Diffuse.log
C         0.1550060       -3.4285510        1.0990180
C        -0.0305700       -4.4389660        0.1741290
C         0.9618650       -4.8078300       -0.7612770
C         0.7206920       -5.8243350       -1.7244760
C         1.7056600       -6.2015630       -2.6059070
C         2.9693180       -5.5640130       -2.5680150
C         3.2295970       -4.5685280       -1.6520730
C         2.2388410       -4.1654840       -0.7119240
C         2.4330380       -3.1140910        0.2468540
C         1.3923870       -2.6968890        1.0727720
C         1.6315930       -1.4657780        1.9158700
H         0.8319290       -1.2865130        2.6328700
H         2.5746830       -1.5491100        2.4608230
N         1.7413280       -0.1472170        1.1160480
C         0.3243190        0.3840280        0.7543140
C        -0.3940590        0.9989090        1.9267850
C        -0.0080930        2.2434700        2.4247500
C        -0.6604720        2.7729940        3.5910830
C        -0.2149970        3.9441920        4.2682990
C        -0.8668150        4.4106660        5.3871480
C        -2.0049350        3.7377640        5.8931540
C        -2.4504420        2.5918610        5.2789170
C        -1.7881100        2.0708260        4.1339330
C        -2.2140940        0.8666200        3.5237030
C        -1.5148320        0.3293320        2.4717810
H        -1.8714860       -0.5803950        2.0079560
H        -3.1078760        0.3684250        3.8866420
H        -3.3143250        2.0559830        5.6637920
H        -2.5161490        4.1240750        6.7705960
H        -0.5028750        5.3035700        5.8882210
```

H	0.6581140	4.4708610	3.9013440
C	1.1216890	2.9581010	1.7744520
C	2.3376090	2.2921530	1.6267200
C	2.4643810	0.8516520	2.0408350
H	3.5070700	0.5276300	2.0612570
H	2.0267060	0.6876100	3.0276450
C	3.4789470	2.9843640	1.1523070
C	3.4029310	4.3055000	0.7866010
C	2.1604170	4.9895760	0.8128820
C	2.0441200	6.3327760	0.3652990
C	0.8214590	6.9626050	0.3445840
C	-0.3417230	6.2657290	0.7508190
C	-0.2635670	4.9689080	1.2077240
C	0.9919500	4.3025450	1.2846250
H	-1.1730440	4.4394270	1.4673130
H	-1.3118300	6.7496320	0.6899030
H	0.7419560	7.9882010	-0.0055870
H	2.9391630	6.8472070	0.0240520
H	4.2919810	4.8338040	0.4512280
H	4.4280460	2.4636480	1.1021080
H	-0.2176190	-0.4715810	0.3490310
H	0.4708430	1.0836110	-0.0693700
C	2.4958970	-0.3173420	-0.2167700
C	3.8030310	-1.0674190	-0.0934870
C	3.7486220	-2.4065290	0.2868330
C	4.9397620	-3.1060410	0.6723560
C	4.9229940	-4.4181040	1.2216960
C	6.0915520	-5.0339410	1.6112770
C	7.3372380	-4.3777960	1.4613140
C	7.3883390	-3.1088280	0.9326460
C	6.1986640	-2.4401100	0.5368100
C	6.2282670	-1.1482730	-0.0402020
C	5.0752390	-0.4581470	-0.3680190
C	5.2138420	0.8203400	-1.1182340
C	4.4866130	1.0397850	-2.3023580
C	4.6874200	2.2066870	-3.0294200
C	5.6005990	3.1733040	-2.6133640
C	6.3284300	2.9415860	-1.4493530
C	6.1512820	1.7829390	-0.7071480
H	6.7328380	1.6535900	0.1994890
H	3.7977880	0.3029250	-2.7084130
H	7.1945310	-0.7075820	-0.2702930
H	8.3395510	-2.5959740	0.8144290
H	8.2508920	-4.8790770	1.7678730
H	6.0583940	-6.0319900	2.0387850
H	3.9743590	-4.9289620	1.3440170
H	1.7988930	-0.8332920	-0.8683230
H	2.6171000	0.6925510	-0.6010510
H	4.1971850	-4.0809640	-1.6547500
H	3.7391980	-5.8551780	-3.2774170
H	1.5118870	-6.9744210	-3.3438910
H	-0.2640620	-6.2796610	-1.7632740
H	-0.9620840	-4.9962840	0.1798800
C	-0.8986320	-3.2504670	2.1375900
C	-0.5533470	-3.2377300	3.5004940
C	-1.5480610	-3.1871950	4.4664080

C	-2.8947400	-3.1726290	4.1145860
C	-3.2322360	-3.2079420	2.7623950
C	-2.2556240	-3.2316190	1.7744120
H	-2.5643570	-3.2063410	0.7349580
H	0.4775250	-3.3159860	3.8290030
O	-4.2047140	-0.6384100	1.1648630
C	-4.1886460	0.0521950	0.1617360
C	-4.4888490	-0.2371880	-1.2373890
C	-4.5935320	1.1154440	-1.9105100
H	-5.6361170	1.4702470	-1.9692130
H	-4.1563870	1.1532480	-2.9104660
N	-3.7931280	1.9561180	-1.0131910
C	-3.7740790	3.3309040	-1.0031560
O	-3.3888980	4.0220360	-0.0770680
O	-4.1976920	3.7561250	-2.2198640
C	-4.0687200	5.1631690	-2.6243770
C	-4.6346500	5.1524850	-4.0472250
H	-5.6759470	4.8141700	-4.0459000
H	-4.5977730	6.1603790	-4.4743930
H	-4.0544560	4.4812750	-4.6885890
C	-4.9174100	6.0549480	-1.7110750
H	-4.9092920	7.0833660	-2.0906130
H	-4.5340840	6.0481270	-0.6899360
H	-5.9556910	5.7054040	-1.6990510
C	-2.5912110	5.5668570	-2.6314300
H	-2.1686850	5.5334120	-1.6259170
H	-2.4887950	6.5853390	-3.0236720
H	-2.0157500	4.8932880	-3.2754820
O	-3.7794440	1.3781960	0.2883390
C	-5.4245280	-1.3084930	-1.6296270
C	-5.7761890	-2.3687150	-0.7709910
C	-6.6579750	-3.3607540	-1.1890300
C	-7.2247890	-3.3542850	-2.4711070
C	-6.8762750	-2.3011180	-3.3232400
C	-5.9958920	-1.3013580	-2.9152740
H	-5.7431450	-0.5095460	-3.6153150
H	-7.2990210	-2.2605270	-4.3254240
C	-8.1547160	-4.4566750	-2.9213550
H	-7.5973640	-5.3460370	-3.2469240
H	-8.8228440	-4.7754430	-2.1125990
H	-8.7766430	-4.1372030	-3.7648190
H	-6.9159300	-4.1608770	-0.4970700
H	-5.3684860	-2.3979460	0.2316620
C	1.2764840	-1.2905770	-4.0539640
C	0.6691240	-2.6732460	-4.3025040
H	2.2832510	-1.3172300	-3.6012180
H	1.3361070	-0.6638720	-4.9537590
H	0.3545930	-2.8311120	-5.3396790
H	1.3272630	-3.5042270	-4.0338860
N	-0.7135000	-1.4730040	-2.7715630
Cl	-2.4222310	-0.9216600	-1.9140560
C	-0.5807380	-2.7055390	-3.4030470
O	-1.3135890	-3.6659750	-3.2654870
C	0.3244090	-0.6238900	-3.0434650
O	0.5313620	0.4789490	-2.5420450
F	-4.5292520	-3.2267980	2.4431820

F	-3.8440970	-3.1391950	5.0567230
F	-1.2247670	-3.1801130	5.7703310
F	4.0172310	2.4177420	-4.1679790
F	5.7803190	4.2943720	-3.3187060
F	7.2004140	3.8787160	-1.0439160

177
R_MinorCf01_ConstOpt.log

C	-0.4362710	3.0656400	-2.5333580
C	-0.1793940	2.8811520	-3.8764880
C	-1.0967170	2.1902950	-4.7197220
C	-0.8113800	1.9886970	-6.1094540
C	-1.6837360	1.2874610	-6.9009530
C	-2.8773580	0.7413330	-6.3431710
C	-3.1779100	0.9306030	-5.0184960
C	-2.3041120	1.6868660	-4.1696240
C	-2.5765670	1.9280200	-2.7796240
C	-1.6517890	2.5683880	-1.9645000
C	-2.0114200	2.7766600	-0.5260940
H	-1.3295200	3.4586110	0.0017590
H	-3.0310310	3.1886590	-0.4463740
N	-2.0150260	1.4710980	0.3077230
C	-0.5609090	1.1688300	0.7936090
C	-0.1891160	2.1023390	1.9126750
C	-0.7489690	1.9606420	3.1742570
C	-0.4805130	2.9315730	4.1999140
C	-1.1335660	2.9070680	5.4745240
C	-0.8621900	3.8634670	6.4200410
C	0.0755920	4.9024940	6.1497040
C	0.7110100	4.9589610	4.9354860
C	0.4424740	3.9765090	3.9294820
C	1.0954110	4.0259410	2.6575980
C	0.7863980	3.1151110	1.6820160
H	1.3128310	3.1251740	0.7259370
H	1.8497940	4.7948300	2.4805700
H	1.4259220	5.7512010	4.7124670
H	0.2716570	5.6469940	6.9206110
H	-1.3600570	3.8465450	7.3890700
H	-1.8569160	2.1164600	5.6814210
C	-1.6828010	0.8373210	3.4095530
C	-2.7939600	0.7314350	2.5860680
C	-2.9904500	1.7169830	1.4732500
H	-4.0002560	1.6731980	1.0403370
H	-2.8071830	2.7452670	1.8263140
C	-3.7708890	-0.2772790	2.8224160
C	-3.5958120	-1.1878200	3.8319350
C	-2.4191680	-1.1558520	4.6455970
C	-2.2041070	-2.1313090	5.6712590
C	-1.0548280	-2.1104160	6.4199270
C	-0.0557040	-1.1227570	6.1760280
C	-0.2411760	-0.1724160	5.2047660
C	-1.4425760	-0.1479940	4.4262020
H	0.5403380	0.5712720	4.9927160
H	0.8567410	-1.1440010	6.7704100
H	-0.8823960	-2.8495200	7.2017510
H	-2.9669500	-2.8919160	5.8364640
H	-4.3425920	-1.9594870	4.0234190

H	-4.6609580	-0.3049770	2.1954950
H	0.0791690	1.2549700	-0.0942690
H	-0.5341480	0.1163980	1.1144280
C	-2.4635740	0.2157210	-0.4929750
C	-3.7719550	0.4551680	-1.1933940
C	-3.8384780	1.4119620	-2.1957960
C	-5.1095480	1.8641710	-2.6926560
C	-5.2374860	2.9877240	-3.5695250
C	-6.4750830	3.4051280	-3.9918690
C	-7.6513930	2.7204440	-3.5692180
C	-7.5590460	1.6347630	-2.7345480
C	-6.2811970	1.1826730	-2.2764710
C	-6.1551280	0.0328080	-1.4384170
C	-4.9362410	-0.3429610	-0.9148810
C	-4.8485000	-1.6310610	-0.1899190
C	-3.8535260	-2.5686870	-0.5290550
C	-3.8239110	-3.8118410	0.1121580
C	-4.7695110	-4.1330490	1.0963740
C	-5.7626060	-3.2018960	1.4231040
C	-5.8118840	-1.9599260	0.7740180
H	-6.6012860	-1.2436890	1.0152090
C	-6.8119500	-3.5304530	2.4473010
F	-7.9882050	-3.9113470	1.9208280
F	-6.5109840	-4.5169680	3.3075600
F	-7.1450340	-2.5090990	3.2597690
H	-4.7292970	-5.1031600	1.6009670
C	-2.7366300	-4.8048940	-0.2251990
F	-2.1956240	-4.7191490	-1.4421200
F	-3.1593600	-6.0910440	-0.1747110
F	-1.7080270	-4.7884180	0.6318460
H	-3.0817320	-2.3272740	-1.2876960
H	-7.0496600	-0.5642370	-1.2501300
H	-8.4513210	1.1014840	-2.4080620
H	-8.6199880	3.0741120	-3.9226220
H	-6.5777150	4.2628310	-4.6564610
H	-4.3340780	3.5063130	-3.8958260
H	-1.6696350	-0.0171220	-1.2183600
H	-2.5112510	-0.5856360	0.2562080
H	-4.0767450	0.4841590	-4.5898990
H	-3.5380100	0.1612750	-6.9866590
H	-1.4768270	1.1260800	-7.9582800
H	0.1164130	2.3917090	-6.5150530
H	0.7507520	3.2519080	-4.3147350
C	0.5779950	3.8228300	-1.7635340
C	0.2744790	5.0964120	-1.2734720
C	1.2715100	5.8553380	-0.6384010
C	2.5749510	5.3631950	-0.5364570
C	2.8743650	4.0841330	-1.0384310
C	1.8807180	3.2995470	-1.6300200
H	2.0774170	2.2610550	-1.9810560
C	4.2997610	3.6027890	-0.9231390
F	4.7643920	3.5576120	0.3330780
F	5.1709620	4.4135460	-1.5598930
F	4.5670240	2.3950700	-1.4291740
H	3.3653730	5.9617960	-0.0726220
C	0.9039550	7.2083250	-0.0973970

F	1.8345850	7.8293630	0.6445200
F	-0.1770110	7.2001750	0.7061230
F	0.5914040	8.1133110	-1.0403480
H	-0.7311840	5.5025380	-1.4029740
O	5.5494940	-0.3135130	-2.0133150
C	5.0324760	-0.5318210	-0.9452900
C	4.7872070	-1.8205110	-0.2330180
C	4.2718190	-1.4669010	1.1256840
H	5.0323060	-1.6461630	1.8977470
H	3.3572610	-2.0083210	1.3799020
N	3.9668890	-0.0322170	1.0111250
C	4.0884070	0.8549710	2.0754890
O	4.2306800	2.0539600	1.9597640
O	3.9528760	0.1438990	3.2180370
C	3.9895200	0.8029400	4.5367540
C	3.8116710	-0.3751140	5.4979950
H	4.6297450	-1.0937840	5.3848110
H	3.8065200	-0.0167920	6.5327080
H	2.8672730	-0.8928840	5.3003300
C	5.3511220	1.4759070	4.7393560
H	5.4184850	1.8677680	5.7604970
H	5.4903490	2.2987640	4.0363330
H	6.1594120	0.7498120	4.6001740
C	2.8308710	1.7918300	4.6634240
H	2.9264320	2.6001610	3.9358860
H	2.8206180	2.2255230	5.6699110
H	1.8660460	1.2837900	4.5028630
O	4.5457270	0.4937790	-0.1811990
C	5.5028380	-3.0547800	-0.5106440
C	6.4186000	-3.1626570	-1.5767140
C	7.0966290	-4.3552390	-1.8009550
C	6.8836310	-5.4820290	-0.9939560
C	5.9640770	-5.3731220	0.0600410
C	5.2860410	-4.1856710	0.3014220
H	4.5615390	-4.1434600	1.1088450
H	5.7728720	-6.2342520	0.6958450
C	7.5981170	-6.7821340	-1.2700880
H	7.0287900	-7.4020470	-1.9761030
H	8.5852960	-6.6119440	-1.7127430
H	7.7305200	-7.3707490	-0.3559830
H	7.8081700	-4.4141790	-2.6214820
H	6.5941790	-2.3031710	-2.2119530
C	1.4549530	-1.0500980	-6.5242310
C	2.8088360	-1.3866540	-6.5703840
C	3.5181940	-1.5995180	-5.3852820
C	2.8855630	-1.4730670	-4.1491390
C	1.5315270	-1.1308680	-4.1206460
C	0.8069410	-0.9223020	-5.2944690
H	-0.2476310	-0.6720540	-5.2331880
S	0.6811400	-0.8640680	-2.5607570
O	1.2312600	0.3819050	-1.9541090
O	-0.7738550	-0.7948060	-2.8683890
N	1.0930430	-2.2232400	-1.7657330
F	3.0613950	-1.9906600	-1.1074530
S	0.3439300	-2.6395020	-0.3790490
O	0.6566740	-1.7630570	0.7789840

O	-1.1150070	-2.8988830	-0.5642040
C	1.1562000	-4.2118170	-0.0754120
C	1.3738440	-4.5801850	1.2537200
C	1.8988620	-5.8427380	1.5376660
C	2.2024850	-6.7240360	0.4980700
C	1.9851010	-6.3413330	-0.8283520
C	1.4557130	-5.0851960	-1.1221120
H	1.2899350	-4.7697280	-2.1455590
H	2.2254180	-7.0246560	-1.6383350
H	2.6053720	-7.7090050	0.7202300
H	2.0626910	-6.1387520	2.5708710
H	1.1293880	-3.8804460	2.0466980
H	3.4115130	-1.6552800	-3.2202550
H	4.5715870	-1.8649310	-5.4197320
H	3.3110920	-1.4876880	-7.5294540
H	0.8982710	-0.8945410	-7.4446130

177
R_MinorCf03_ConstOpt.log

C	-2.5230790	-0.1414020	-2.5532610
C	-2.7783390	1.1744990	-2.8786230
C	-1.7409210	2.0363680	-3.3454620
C	-1.9966030	3.4159460	-3.6296870
C	-1.0019410	4.2132500	-4.1357580
C	0.3002240	3.6812800	-4.3676380
C	0.5782100	2.3688980	-4.0804170
C	-0.4420920	1.5068060	-3.5639020
C	-0.1976820	0.1346060	-3.2214070
C	-1.1764510	-0.6345800	-2.6101310
C	-0.8004210	-1.9795540	-2.0697910
H	-1.6588830	-2.5416000	-1.6745380
H	-0.3072360	-2.5897840	-2.8441770
N	0.1995560	-1.9091130	-0.8886140
C	-0.5359730	-1.4510910	0.4104010
C	-1.4416230	-2.5367990	0.9139610
C	-0.9296530	-3.6943310	1.4806050
C	-1.8148900	-4.7677170	1.8399220
C	-1.3364440	-6.0449990	2.2770640
C	-2.2142850	-7.0479040	2.6020690
C	-3.6209430	-6.8343950	2.5114340
C	-4.1099820	-5.6252570	2.0868270
C	-3.2165510	-4.5643520	1.7316670
C	-3.7142210	-3.3032400	1.2730220
C	-2.8500650	-2.3213760	0.8681410
H	-3.2060560	-1.3280290	0.5476050
H	-4.7919520	-3.1390810	1.2581450
H	-5.1830550	-5.4503970	2.0103120
H	-4.2938140	-7.6474150	2.7823440
H	-1.8524990	-8.0209460	2.9325570
H	-0.2596390	-6.2085600	2.3437000
C	0.5335740	-3.7891300	1.6796940
C	1.3679090	-3.5995120	0.5873030
C	0.7865610	-3.3310510	-0.7685510
H	1.5260570	-3.4227040	-1.5767430
H	-0.0426420	-4.0264390	-0.9744740
C	2.7795030	-3.7190660	0.7336520
C	3.3336470	-3.9747970	1.9603680

C	2.5048400	-4.1020200	3.1195250
C	3.0782290	-4.3209320	4.4128850
C	2.2768540	-4.4036270	5.5230720
C	0.8634920	-4.2655120	5.3993210
C	0.2886670	-4.0656920	4.1698050
C	1.0945780	-3.9964100	2.9872680
H	-0.7929020	-3.9431620	4.0792040
H	0.2510060	-4.3151680	6.2991580
H	2.7040140	-4.5670030	6.5120920
H	4.1608070	-4.4108900	4.4956970
H	4.4134150	-4.0736310	2.0753400
H	3.4093960	-3.6160590	-0.1465520
H	-1.0802530	-0.5297270	0.1614380
H	0.2585760	-1.2176720	1.1356280
C	1.3488300	-0.8791690	-1.0797960
C	1.9192860	-0.9155920	-2.4683060
C	1.1225770	-0.4733590	-3.5145660
C	1.5429910	-0.6156070	-4.8815700
C	0.6913770	-0.3039340	-5.9901670
C	1.1308440	-0.4751930	-7.2786110
C	2.4454910	-0.9634170	-7.5357460
C	3.2848520	-1.2705940	-6.4950480
C	2.8480250	-1.1051130	-5.1417940
C	3.7095520	-1.4032540	-4.0445020
C	3.2730460	-1.3060160	-2.7393910
C	4.2830750	-1.5287780	-1.6785140
C	4.5964610	-0.5019820	-0.7697520
C	5.6250410	-0.6905830	0.1601810
C	6.3373460	-1.8991870	0.2058370
C	6.0288040	-2.9113590	-0.7083290
C	5.0126090	-2.7252020	-1.6582850
H	4.7872410	-3.5096190	-2.3865310
C	6.7808190	-4.2143940	-0.7121970
F	7.6947980	-4.3800690	0.2559670
F	5.9925710	-5.2979310	-0.5889540
F	7.4678720	-4.4443450	-1.8439330
H	7.1293290	-2.0442250	0.9472020
C	5.9683360	0.4078590	1.1337780
F	7.2224130	0.3698060	1.6194800
F	5.2017300	0.4139540	2.2364320
F	5.8646470	1.6576310	0.6562830
H	4.0369390	0.4563310	-0.8181110
H	4.7414990	-1.6868320	-4.2636900
H	4.2923450	-1.6428950	-6.6782810
H	2.7668000	-1.0859300	-8.5702740
H	0.4841800	-0.2405810	-8.1243130
H	-0.3146070	0.0729330	-5.7936730
H	0.9167160	0.1039030	-0.8529180
H	2.0743980	-1.1198050	-0.2942740
H	1.5843550	1.9710130	-4.2243290
H	1.0717210	4.3397440	-4.7650050
H	-1.1873010	5.2663500	-4.3498000
H	-2.9867730	3.8223050	-3.4128080
H	-3.7884380	1.5860270	-2.7833070
C	-3.6752880	-1.0272620	-2.2686900
C	-3.8429590	-2.2028910	-3.0112180

C	-4.9685620	-3.0149050	-2.7990050
C	-5.9440780	-2.6454360	-1.8693150
C	-5.7764810	-1.4617310	-1.1320250
C	-4.6545850	-0.6509190	-1.3295690
H	-4.5025780	0.2832810	-0.7544770
C	-6.8431210	-1.0521680	-0.1506970
F	-6.4395930	-0.2761650	0.8648440
F	-7.4582060	-2.0768490	0.4709870
F	-7.8538370	-0.3674190	-0.7099450
H	-6.8338900	-3.2641640	-1.7201400
C	-5.0998220	-4.2761480	-3.6077010
F	-5.2542010	-4.0683240	-4.9265780
F	-6.1197650	-5.0882350	-3.2912110
F	-4.0237520	-5.0824100	-3.5362050
H	-3.1045310	-2.4740510	-3.7700500
O	1.8697630	-0.2175190	2.2472300
C	1.9901300	0.9571590	1.9533120
C	1.6810820	2.2005900	2.6604710
C	2.2368410	3.3255650	1.8408210
H	3.1825710	3.6998390	2.2680230
H	1.5274740	4.1458520	1.7179050
N	2.4602190	2.6790080	0.5458260
C	3.2376240	3.1280540	-0.4957920
O	3.6755090	2.4047030	-1.3769340
O	3.3586240	4.4637850	-0.3846790
C	3.9634830	5.2658270	-1.4670040
C	5.4348630	4.8783190	-1.6387400
H	5.5327020	3.8525800	-1.9969700
H	5.9111750	5.5536550	-2.3585580
H	5.9663170	4.9712510	-0.6846820
C	3.1546340	5.0858260	-2.7554440
H	3.5322360	5.7716670	-3.5220200
H	2.0986810	5.3158570	-2.5755490
H	3.2353380	4.0624560	-3.1264120
C	3.8322400	6.6927560	-0.9311560
H	2.7821060	6.9508030	-0.7628340
H	4.2560430	7.4029000	-1.6490980
H	4.3681990	6.7995340	0.0177430
O	2.5411790	1.2734770	0.7233320
C	1.3546580	2.3110820	4.0641750
C	1.3963450	1.1992120	4.9316520
C	1.1024250	1.3509780	6.2829160
C	0.7492130	2.5972950	6.8198710
C	0.6869810	3.6968190	5.9479710
C	0.9830090	3.5650010	4.5990020
H	0.8742130	4.4193670	3.9387100
H	0.3871730	4.6684840	6.3332900
C	0.4501210	2.7615130	8.2897830
H	1.2673310	3.2841530	8.8050590
H	-0.4583610	3.3538610	8.4489270
H	0.3156830	1.7937800	8.7835310
H	1.1514050	0.4832000	6.9372790
H	1.6650170	0.2258370	4.5359220
C	-5.1612370	1.9365750	4.3521300
C	-4.2010200	1.5164310	5.2747640
C	-2.8791800	1.3133380	4.8690820

C	-2.5122990	1.5180680	3.5397690
C	-3.4862680	1.9339270	2.6287460
C	-4.8069050	2.1508990	3.0198330
H	-5.5309530	2.4913930	2.2884820
S	-3.0537840	2.1089750	0.8947140
O	-2.7105710	0.7465430	0.4099430
O	-4.2169920	2.7056190	0.1966530
N	-1.6907220	3.0149200	0.7617210
F	-0.1499040	2.2512990	1.6378840
S	-1.7491210	4.6286510	1.1964010
O	-2.8491320	4.9246490	2.1270640
O	-0.3840410	5.0383890	1.5734750
C	-2.1044970	5.5172400	-0.3419980
C	-3.4184470	5.5968230	-0.8079280
C	-3.6822060	6.3373650	-1.9601940
C	-2.6480260	6.9996770	-2.6270590
C	-1.3393420	6.9141410	-2.1482330
C	-1.0611970	6.1689730	-1.0003070
H	-0.0557420	6.1021500	-0.5979270
H	-0.5353190	7.4401400	-2.6571710
H	-2.8647000	7.5895100	-3.5142660
H	-4.7023990	6.4126410	-2.3271610
H	-4.2096890	5.0780500	-0.2796900
H	-1.4876570	1.3943170	3.2075660
H	-2.1259850	1.0018990	5.5880880
H	-4.4815680	1.3539230	6.3127490
H	-6.1873560	2.1038660	4.6696690

177
R_MinorCf04_ConstOpt.log

C	-3.4771340	-0.6656170	2.1812060
C	-3.4676480	-1.8404090	2.9039850
C	-2.4148110	-2.1415500	3.8191180
C	-2.3736370	-3.3900650	4.5171250
C	-1.3609390	-3.6528570	5.4049850
C	-0.3377750	-2.6878250	5.6350240
C	-0.3562340	-1.4827020	4.9785450
C	-1.4073700	-1.1716560	4.0560790
C	-1.4611080	0.0679320	3.3303150
C	-2.3931350	0.2656350	2.3210920
C	-2.2744350	1.4821000	1.4518690
H	-3.1062710	1.5801620	0.7382810
H	-2.2263760	2.3993880	2.0616390
N	-1.0032810	1.4844100	0.5750870
C	-1.1561880	0.4412810	-0.5745710
C	-2.1755900	0.9123420	-1.5732070
C	-1.9109180	1.9763840	-2.4217990
C	-2.9496060	2.4992950	-3.2676950
C	-2.7835810	3.6948810	-4.0382380
C	-3.8037900	4.1694090	-4.8236360
C	-5.0480300	3.4771910	-4.8871470
C	-5.2404920	2.3333970	-4.1542030
C	-4.1959690	1.8221570	-3.3198340
C	-4.3851430	0.6363910	-2.5406850
C	-3.4049770	0.1984040	-1.6908570
H	-3.5150530	-0.7320950	-1.1211140
H	-5.3214860	0.0907740	-2.6448990

H	-6.1873000	1.7964230	-4.1932910
H	-5.8374400	3.8741210	-5.5243510
H	-3.6796440	5.0795630	-5.4092540
H	-1.8300980	4.2242660	-3.9886070
C	-0.5520860	2.5604020	-2.4106230
C	-0.0335630	3.0067920	-1.2030570
C	-0.8607120	2.9303000	0.0450370
H	-0.4390030	3.5149940	0.8746300
H	-1.8840880	3.2904940	-0.1484820
C	1.2644310	3.5894980	-1.1577100
C	2.0308090	3.6771510	-2.2908050
C	1.5442040	3.1773870	-3.5390070
C	2.3485760	3.2432780	-4.7220010
C	1.8820370	2.7364200	-5.9078470
C	0.5988080	2.1179260	-5.9679670
C	-0.1915280	2.0434270	-4.8492450
C	0.2487720	2.5988050	-3.6035100
H	-1.1571790	1.5358900	-4.8870600
H	0.2662240	1.6927870	-6.9138700
H	2.4844960	2.7845590	-6.8135600
H	3.3418620	3.6861380	-4.6517790
H	3.0328380	4.1064760	-2.2554720
H	1.6405160	3.9673980	-0.2116310
H	-1.4501510	-0.5007700	-0.0922490
H	-0.1663930	0.3188810	-1.0386880
C	0.2901450	1.0877130	1.3435690
C	0.3227040	1.6953700	2.7197540
C	-0.5353330	1.1727970	3.6809320
C	-0.5889050	1.7111680	5.0127360
C	-1.5616840	1.3039850	5.9842080
C	-1.5550130	1.8348620	7.2486460
C	-0.5785820	2.8053090	7.6224220
C	0.3525990	3.2311830	6.7111730
C	0.3556540	2.7021690	5.3794390
C	1.2824780	3.1679370	4.4047940
C	1.2573250	2.7121230	3.0997770
C	2.2357080	3.3528740	2.1894380
C	3.2476680	2.6319910	1.5357940
C	4.2437680	3.3283590	0.8345810
C	4.2178200	4.7258110	0.7384280
C	3.1844160	5.4357380	1.3609680
C	2.2029960	4.7549210	2.0928990
H	1.4082160	5.3119730	2.5970480
C	3.1055580	6.9350670	1.2777330
F	4.0235590	7.5423360	0.5108700
F	1.9369530	7.3936300	0.7938210
F	3.2237020	7.5536200	2.4659480
H	4.9958200	5.2573170	0.1818430
C	5.3500070	2.5498490	0.1674160
F	4.9769870	1.9110880	-0.9471380
F	5.9014310	1.6031570	0.9443330
F	6.4126390	3.2827780	-0.2185660
H	3.2506180	1.5227540	1.5551620
H	2.0249240	3.9077450	4.7157250
H	1.1013630	3.9761770	6.9791170
H	-0.5920020	3.1992940	8.6385700

H	-2.2939730	1.5249440	7.9875920
H	-2.3134720	0.5638540	5.7040830
H	0.2936600	-0.0086960	1.4157310
H	1.1257350	1.3798460	0.6968070
H	0.4491630	-0.7590710	5.1224250
H	0.4664910	-2.9309650	6.3287750
H	-1.3158870	-4.6024530	5.9379230
H	-3.1510030	-4.1266500	4.3185490
H	-4.2770260	-2.5659970	2.7969320
C	-4.6965180	-0.3686650	1.3943330
C	-5.3928840	0.8275380	1.6167380
C	-6.6018370	1.0722950	0.9488810
C	-7.1542380	0.1056000	0.1029730
C	-6.4713380	-1.1044730	-0.0921820
C	-5.2324890	-1.3317480	0.5191350
H	-4.6542300	-2.2503130	0.3205760
C	-7.0775890	-2.1838520	-0.9501620
F	-6.4972330	-2.3281600	-2.1528050
F	-8.3799750	-2.0343830	-1.2452030
F	-7.0215720	-3.4134650	-0.4109700
H	-8.1102950	0.2886290	-0.3966740
C	-7.2993410	2.3847470	1.1888970
F	-6.5041470	3.4603350	1.0449020
F	-7.7960650	2.5165570	2.4305650
F	-8.3447210	2.6583700	0.3940460
H	-4.9981580	1.5587480	2.3278470
O	1.1502050	-1.9727940	2.6080360
C	0.7463790	-2.8023300	1.8212910
C	1.3468760	-4.0323860	1.2961340
C	0.3720530	-4.6550570	0.3421910
H	0.0985730	-5.6783030	0.6262770
H	0.7476210	-4.6420250	-0.6850390
N	-0.8102100	-3.7886630	0.4784730
C	-1.7653350	-3.5651900	-0.4884970
O	-2.5507600	-2.6263950	-0.4731510
O	-1.7392700	-4.6006840	-1.3381800
C	-2.6369610	-4.6729380	-2.5122290
C	-4.0677850	-4.8992990	-2.0199820
H	-4.7423640	-5.0061980	-2.8780790
H	-4.1317760	-5.8150020	-1.4218690
H	-4.4034210	-4.0556920	-1.4173010
C	-2.4932660	-3.4177070	-3.3757030
H	-1.4362910	-3.2202560	-3.5771240
H	-2.9243660	-2.5418340	-2.8880550
H	-3.0107930	-3.5782810	-4.3290970
C	-2.1003590	-5.8997740	-3.2515910
H	-2.1360670	-6.7871790	-2.6110360
H	-1.0644690	-5.7328440	-3.5624040
H	-2.7063720	-6.0919310	-4.1436530
O	-0.5135850	-2.6447650	1.2674050
C	2.4456210	-4.7444010	1.9061920
C	3.1249300	-4.2418770	3.0380610
C	4.1610770	-4.9649950	3.6174640
C	4.5743370	-6.2015980	3.0988250
C	3.9012910	-6.6975860	1.9719500
C	2.8605730	-5.9896920	1.3860660

H	2.3711790	-6.3981120	0.5069100
H	4.2012630	-7.6531900	1.5478070
C	5.7233050	-6.9624520	3.7136800
H	5.8647640	-6.6958280	4.7661470
H	5.5665740	-8.0451020	3.6539250
H	6.6655560	-6.7444570	3.1923550
H	4.6615060	-4.5627390	4.4955340
H	2.8221710	-3.2875490	3.4518360
C	3.3614990	-0.5928300	-6.2869470
C	3.6368820	-1.8054690	-6.9228770
C	3.3088990	-3.0132400	-6.3020440
C	2.7060050	-3.0118850	-5.0444470
C	2.4390360	-1.7920060	-4.4195220
C	2.7599980	-0.5777310	-5.0282070
H	2.5482240	0.3546180	-4.5160350
S	1.6573210	-1.7989820	-2.7950780
O	0.8669110	-0.5536470	-2.6968950
O	0.9391710	-3.0843330	-2.6915190
N	2.9077630	-1.8760560	-1.7136050
F	2.1652450	-2.7484340	-0.1355920
S	3.6631600	-0.4908280	-1.1980820
O	4.0415380	0.3947880	-2.3211870
O	2.9851750	0.1655140	-0.0494740
C	5.1649130	-1.2344430	-0.5495820
C	6.3743010	-0.9707900	-1.1912710
C	7.5509590	-1.5097820	-0.6666920
C	7.5069040	-2.3029120	0.4806650
C	6.2845840	-2.5639590	1.1072830
C	5.1035660	-2.0282430	0.5975330
H	4.1438710	-2.2375920	1.0524510
H	6.2464120	-3.1908150	1.9939140
H	8.4251210	-2.7206450	0.8863550
H	8.5006210	-1.3073120	-1.1553080
H	6.3833500	-0.3535410	-2.0832170
H	2.4312390	-3.9370630	-4.5499740
H	3.5187300	-3.9569760	-6.7983950
H	4.1064740	-1.8100270	-7.9031230
H	3.6156790	0.3464530	-6.7701760

159
R_MinorCl_14_MaruokaCF3_TS_Diffuse.log

C	-1.2271100	-0.5574250	2.2847740
C	-2.0878990	-1.6203630	2.1345390
C	-1.6028830	-2.9318080	1.8414530
C	-2.4983300	-3.9948170	1.5115370
C	-2.0147180	-5.2609230	1.2899580
C	-0.6204600	-5.5288620	1.4107490
C	0.2626410	-4.5193350	1.7118470
C	-0.2060980	-3.1820520	1.8990640
C	0.6791980	-2.0617820	2.0725220
C	0.1814680	-0.7681710	2.0874620
C	1.1257150	0.3797950	1.9274080
H	0.6188950	1.3552560	1.9797060
H	1.9271190	0.3551850	2.6829870
N	1.8418990	0.3879520	0.5561460
C	0.8256820	0.6925430	-0.5819590
C	0.3798280	2.1227290	-0.5202710

C	1.2410550	3.1494390	-0.8824640
C	0.8312350	4.5169580	-0.7226030
C	1.7240720	5.6181700	-0.9290560
C	1.2924460	6.9096990	-0.7641500
C	-0.0553380	7.1795180	-0.3841170
C	-0.9312200	6.1467120	-0.1680670
C	-0.5048750	4.7883500	-0.3223370
C	-1.4027840	3.7033020	-0.0767950
C	-0.9666960	2.4064610	-0.1549140
H	-1.6563020	1.5752780	0.0221650
H	-2.4487760	3.9151740	0.1683040
H	-1.9627150	6.3371730	0.1302310
H	-0.3695280	8.2161150	-0.2655390
H	1.9705910	7.7487980	-0.9177500
H	2.7565970	5.4120040	-1.2151870
C	2.5782220	2.7925300	-1.4099160
C	3.3972490	1.9700630	-0.6486660
C	2.9296060	1.4726660	0.6875570
H	3.7357050	1.0205250	1.2812590
H	2.4861430	2.2985200	1.2663630
C	4.7027220	1.6295710	-1.1057920
C	5.1497330	2.0692210	-2.3239100
C	4.3037550	2.8588680	-3.1655960
C	4.7451700	3.2820190	-4.4597120
C	3.9127260	4.0055100	-5.2755150
C	2.5960260	4.3381950	-4.8426300
C	2.1531360	3.9539540	-3.6022420
C	3.0034130	3.2151550	-2.7172240
H	1.1390040	4.1978770	-3.2780800
H	1.9480740	4.8972770	-5.5177600
H	4.2384660	4.3294380	-6.2641810
H	5.7501030	3.0124000	-4.7836990
H	6.1520360	1.8191850	-2.6745410
H	5.3446280	1.0281970	-0.4654110
H	0.0259990	-0.0527130	-0.4991040
H	1.3595950	0.4776270	-1.5191380
C	2.4923030	-0.9745550	0.1470510
C	3.0314620	-1.7290190	1.3265230
C	2.1383860	-2.2605960	2.2474220
C	2.6047440	-2.9499540	3.4212070
C	1.7192500	-3.4273540	4.4410480
C	2.2095880	-4.0701450	5.5497420
C	3.6115490	-4.2742260	5.7071990
C	4.4862800	-3.8260000	4.7500900
C	3.9988540	-3.1511040	3.5856820
C	4.8936760	-2.6856820	2.5763820
C	4.4323020	-2.0027810	1.4718860
C	5.4300020	-1.6415300	0.4379560
C	5.3355740	-2.1893600	-0.8516190
C	6.3084290	-1.8827040	-1.8118190
C	7.3926000	-1.0567650	-1.4893930
C	7.4952610	-0.5341310	-0.1931620
C	6.5175240	-0.8189440	0.7692380
H	6.5892820	-0.4091830	1.7811500
C	8.6527450	0.3638760	0.1597130
F	9.0190290	0.3486380	1.4517170

F	9.7969980	0.1154850	-0.4963020
F	8.4065570	1.6618360	-0.0931550
H	8.1556060	-0.8236660	-2.2394030
C	6.1704890	-2.4802230	-3.1899840
F	6.3578590	-3.8092420	-3.2174340
F	7.0162690	-2.0117650	-4.1215750
F	4.9598850	-2.3028120	-3.7425180
H	4.5046160	-2.8644130	-1.0918120
H	5.9581740	-2.9014310	2.6917950
H	5.5600560	-3.9755660	4.8585810
H	3.9704750	-4.7902970	6.5981710
H	1.5357880	-4.4353920	6.3256770
H	0.6435020	-3.2743170	4.3229140
H	1.6990470	-1.5427640	-0.3919790
H	3.2734080	-0.6829320	-0.5710220
H	1.3306810	-4.7214480	1.7872530
H	-0.2668010	-6.5452840	1.2371350
H	-2.6845540	-6.0717350	1.0000970
H	-3.5633080	-3.7739500	1.3971550
H	-3.1713620	-1.4915240	2.2616030
C	-1.7570090	0.7231940	2.8037290
C	-1.1335410	1.3168870	3.9108240
C	-1.6708810	2.4782440	4.4871330
C	-2.8624090	3.0244700	4.0042470
C	-3.5037460	2.4117690	2.9154900
C	-2.9439360	1.2875010	2.3007490
H	-3.4374050	0.8219090	1.4300420
C	-4.8123120	2.9787540	2.4220990
F	-4.6839470	3.9817220	1.5397960
F	-5.5828490	3.5128470	3.3914010
F	-5.6391520	2.1067610	1.8288880
H	-3.2927990	3.9196620	4.4625130
C	-0.9241350	3.0943810	5.6387740
F	-1.4738060	4.1719360	6.2174450
F	0.3131920	3.5109650	5.3020970
F	-0.7071950	2.2563170	6.6674010
H	-0.2411340	0.8554650	4.3419550
O	-5.0375520	-1.7396170	1.2575580
C	-5.0728200	-1.1609210	0.2001440
C	-5.3293730	-1.6595470	-1.2204530
C	-5.5504550	-0.3432760	-1.9738220
H	-6.6129640	-0.0593340	-1.9473590
H	-5.2010910	-0.3711270	-3.0054490
N	-4.7292340	0.5838640	-1.2077540
C	-4.6531490	1.9623390	-1.3695920
O	-4.2680200	2.7240510	-0.5036670
O	-5.0151730	2.2447110	-2.6331460
C	-4.8501830	3.6049860	-3.1967070
C	-5.3261030	3.4189440	-4.6382750
H	-6.3652750	3.0757880	-4.6625960
H	-5.2641510	4.3706120	-5.1771480
H	-4.7055890	2.6839620	-5.1609490
C	-5.7512110	4.5846970	-2.4409650
H	-5.7141130	5.5664080	-2.9273040
H	-5.4346590	4.6935700	-1.4023550
H	-6.7901870	4.2379640	-2.4585640

```
C       -3.3748480        4.0048160       -3.1544130
H       -3.0246030        4.1163170       -2.1267770
H       -3.2381320        4.9587080       -3.6766640
H       -2.7592260        3.2515720       -3.6589950
O       -4.7807880        0.1881300        0.1653820
C       -6.4074210       -2.6958650       -1.4179930
C       -6.8777740       -3.5066600       -0.3768640
C       -7.8683280       -4.4573720       -0.6169540
C       -8.4131360       -4.6428820       -1.8929700
C       -7.9326240       -3.8335370       -2.9303480
C       -6.9458240       -2.8796230       -2.6999700
H       -6.5806820       -2.2876380       -3.5349120
H       -8.3317650       -3.9552850       -3.9351540
C       -9.4583710       -5.7018560       -2.1496110
H       -8.9935740       -6.6708960       -2.3761800
H      -10.1047780       -5.8489750       -1.2772450
H      -10.0946460       -5.4409380       -3.0024290
H       -8.2249580       -5.0687450        0.2094390
H       -6.4740290       -3.3876040        0.6203220
C        0.9318300       -4.0765060       -2.3207190
C        0.1752480       -5.1032030       -3.1564800
H        1.3174890       -4.4765460       -1.3749210
H        1.7818350       -3.6099640       -2.8362610
H        0.5479680       -5.1944460       -4.1832390
H        0.1618860       -6.1113930       -2.7306120
N       -1.3354800       -3.3079870       -2.5546350
Cl       -3.2519260       -2.5097250       -1.8815190
C       -1.2653370       -4.5304730       -3.1939640
O       -2.1921810       -5.1108840       -3.7417470
C       -0.1438950       -3.0062240       -2.0227910
O        0.1371390       -1.9864560       -1.3476060
147
R_MinorCl_1_MaruokaF3_TS_Diffuse.log
C        1.4487530        2.8067630        0.9001760
C        2.2690400        3.3246750       -0.0843720
C        1.7686350        3.7999100       -1.3192860
C        2.6432780        4.2283020       -2.3529770
C        2.1354880        4.7189460       -3.5327680
C        0.7360780        4.7995100       -3.7253070
C       -0.1360830        4.3893020       -2.7417750
C        0.3526430        3.8765890       -1.5087850
C       -0.5040350        3.3791570       -0.4707080
C        0.0316830        2.7446210        0.6485640
C       -0.9177280        1.9961720        1.5541780
H       -0.4156300        1.5915470        2.4314680
H       -1.7434590        2.6305170        1.8861960
N       -1.6005330        0.7748610        0.8980240
C       -0.5991320       -0.3837370        0.6911430
C       -0.2315930       -1.1016880        1.9628310
C       -1.1452490       -1.9525630        2.5823490
C       -0.8041770       -2.5635080        3.8395570
C       -1.7355480       -3.3089110        4.6169700
C       -1.3702830       -3.8694840        5.8201160
C       -0.0530080       -3.7210840        6.3156480
C        0.8676940       -2.9899090        5.6030050
C        0.5185170       -2.3834940        4.3660870
```

C	1.4477830	-1.5888760	3.6485670
C	1.0730720	-0.9491410	2.4932900
H	1.7871240	-0.3328620	1.9609780
H	2.4585770	-1.4795040	4.0329380
H	1.8782150	-2.8539000	5.9799710
H	0.2231830	-4.1762370	7.2625640
H	-2.1017080	-4.4288400	6.3967140
H	-2.7509350	-3.4280820	4.2579170
C	-2.4752850	-2.1607730	1.9464620
C	-3.2539320	-1.0466340	1.6331000
C	-2.7217160	0.3446110	1.8654600
H	-3.5055430	1.0968770	1.7543680
H	-2.2885520	0.4366200	2.8637720
C	-4.5739360	-1.2126610	1.1437450
C	-5.0917580	-2.4652130	0.9221580
C	-4.2924460	-3.6207620	1.1118780
C	-4.7796260	-4.9172760	0.7934920
C	-3.9701210	-6.0229880	0.9155310
C	-2.6322230	-5.8755660	1.3545000
C	-2.1398690	-4.6372170	1.6990520
C	-2.9549790	-3.4735330	1.6101440
H	-1.1075700	-4.5413960	2.0133750
H	-1.9825650	-6.7439640	1.4044060
H	-4.3475240	-7.0084000	0.6572520
H	-5.8002720	-5.0146050	0.4319580
H	-6.1118060	-2.5821160	0.5654840
H	-5.1838760	-0.3349650	0.9618010
H	0.2694550	0.0664330	0.2109800
H	-1.0634050	-1.0511390	-0.0356930
C	-2.1730420	1.0835470	-0.5042550
C	-2.8202880	2.4399170	-0.6384590
C	-1.9849590	3.5544790	-0.5769480
C	-2.5284800	4.8840290	-0.5728670
C	-1.7420300	6.0516700	-0.3617240
C	-2.3194660	7.3016390	-0.3578950
C	-3.7104830	7.4562020	-0.5710860
C	-4.5012600	6.3486060	-0.7657870
C	-3.9366750	5.0440220	-0.7571270
C	-4.7383010	3.8948780	-0.9380800
C	-4.2296750	2.6095480	-0.8737420
C	-5.1985310	1.4970080	-1.1049950
C	-4.9622690	0.4614850	-2.0270550
C	-5.9496570	-0.4912620	-2.2449680
C	-7.1719710	-0.4545420	-1.5780340
C	-7.4017710	0.5782080	-0.6738440
C	-6.4355900	1.5444280	-0.4337410
H	-6.6531460	2.3227850	0.2894110
H	-4.0287610	0.3483940	-2.5715650
H	-5.7933670	4.0356170	-1.1549960
H	-5.5722750	6.4518850	-0.9210260
H	-4.1495390	8.4498100	-0.5732410
H	-1.7013950	8.1788640	-0.1890120
H	-0.6760150	5.9529440	-0.1952250
H	-1.3366000	0.9736270	-1.1945220
H	-2.8446790	0.2582070	-0.7161440
H	-1.2047880	4.4313740	-2.9181750

H	0.3449100	5.1704200	-4.6685260
H	2.8078410	5.0217590	-4.3298040
H	3.7138040	4.1088240	-2.2161920
H	3.3359120	3.3979640	0.0976930
C	2.0569330	2.4830030	2.2226190
C	1.4593540	2.9641020	3.4018690
C	2.0411290	2.7214820	4.6381360
C	3.2305220	2.0081510	4.7449690
C	3.8303330	1.5512330	3.5737250
C	3.2726950	1.7777620	2.3220950
H	3.7950930	1.3804090	1.4552440
H	0.5604940	3.5703590	3.3718820
O	2.7365490	-0.1611490	-4.3993940
C	2.9981710	-0.1816390	-3.2233500
C	3.1635080	-1.2706040	-2.2424450
C	3.3371840	-0.5515450	-0.9116030
H	4.0766090	-0.9912380	-0.2450040
H	2.3809970	-0.4348610	-0.3810990
N	3.7659440	0.8070360	-1.2984530
C	5.0920750	1.2434500	-1.2680850
O	5.5486920	2.0908730	-2.0096700
O	5.7192030	0.6744510	-0.2094390
C	7.1480130	0.9525230	0.0589720
C	7.4454720	0.0390830	1.2505800
H	6.7917200	0.2748790	2.0949320
H	7.2969150	-1.0110790	0.9784910
H	8.4856620	0.1692570	1.5683250
C	7.9941530	0.5527700	-1.1546450
H	7.7963510	1.2006630	-2.0101860
H	7.7829210	-0.4835970	-1.4391650
H	9.0566300	0.6247580	-0.8948110
C	7.3247420	2.4258530	0.4389950
H	8.3670710	2.6075030	0.7256420
H	6.6902390	2.6788290	1.2969570
H	7.0721450	3.0789740	-0.3985480
O	3.1499550	1.0669760	-2.5532080
C	4.0171230	-2.4340220	-2.5826590
C	4.3771840	-2.7304970	-3.9145990
C	5.1770850	-3.8284370	-4.2143310
C	5.6520730	-4.6908420	-3.2144540
C	5.2833750	-4.4071980	-1.8955710
C	4.4819570	-3.3081980	-1.5841000
H	4.1951770	-3.1494860	-0.5472900
H	5.6197900	-5.0624470	-1.0938070
C	6.5122440	-5.8860480	-3.5603300
H	6.9581380	-6.3271630	-2.6593450
H	5.9266790	-6.6757240	-4.0611900
H	7.3334400	-5.6128460	-4.2438360
H	5.4388890	-4.0212650	-5.2541730
H	4.0192870	-2.0905350	-4.7135400
C	-2.3049380	-4.7085130	-2.6566660
C	-2.9181380	-3.3470470	-3.0140640
H	-2.8635420	-5.2433840	-1.8781800
H	-2.1974410	-5.3903920	-3.5097670
H	-3.0438260	-3.1988310	-4.0979650
H	-3.8923380	-3.1456980	-2.5528340

N	-0.7669480	-2.9816550	-2.0604220
Cl	1.0420640	-2.1536430	-2.1198360
C	-1.8839030	-2.3289800	-2.5132580
O	-2.0591680	-1.1069460	-2.5162830
C	-0.9038570	-4.3712240	-2.1118040
O	-0.0713690	-5.1877680	-1.7639820
F	1.4685480	3.1877430	5.7587180
F	3.7861850	1.7705620	5.9383060
F	4.9739700	0.8534570	3.6931980
F	-8.5764050	0.6159860	-0.0249270
F	-8.0968440	-1.3985060	-1.7894190
F	-5.7485450	-1.4902330	-3.1175870

159

R_MinorCl_2_MaruokaCF3_TS_Diffuse.log

C	-1.9560060	0.2135520	2.1069370
C	-2.7417460	-0.9205020	2.1857640
C	-2.1843980	-2.1994670	2.4216720
C	-3.0051070	-3.3599440	2.4394970
C	-2.4611270	-4.5977890	2.6882290
C	-1.0708070	-4.7259440	2.9213830
C	-0.2495470	-3.6206350	2.9090280
C	-0.7810810	-2.3223950	2.6693980
C	0.0246680	-1.1369540	2.6136140
C	-0.5338520	0.0843530	2.2463170
C	0.4034270	1.2433630	2.0252080
H	-0.1246770	2.1797480	1.8514420
H	1.0682840	1.3751530	2.8821910
N	1.3570740	1.1059070	0.8114960
C	0.6386070	1.4331630	-0.5175240
C	0.3560920	2.8987520	-0.7254180
C	1.3875690	3.8014910	-0.9876050
C	1.0872730	5.2055130	-1.0798000
C	2.0937540	6.2102330	-1.1532400
C	1.7631640	7.5445160	-1.2280980
C	0.4079030	7.9524470	-1.2398170
C	-0.5906090	7.0115830	-1.1504430
C	-0.2844160	5.6270560	-1.0513960
C	-1.3078150	4.6551570	-0.9169960
C	-0.9940720	3.3311700	-0.7310660
H	-1.7778450	2.5903480	-0.6371330
H	-2.3482600	4.9645490	-0.9483360
H	-1.6352820	7.3118350	-1.1402790
H	0.1615180	9.0083760	-1.3070320
H	2.5503220	8.2920490	-1.2737500
H	3.1374840	5.9180020	-1.1362300
C	2.7759790	3.2830850	-1.1385230
C	3.3118350	2.4791820	-0.1327490
C	2.4851540	2.1205090	1.0719170
H	3.0968840	1.6812940	1.8625710
H	1.9824850	3.0039810	1.4709780
C	4.6616220	2.0536590	-0.1963230
C	5.4569440	2.3740670	-1.2680380
C	4.9218090	3.0931990	-2.3665970
C	5.7047870	3.3592590	-3.5223260
C	5.1644350	4.0093770	-4.6065730
C	3.8089020	4.4151560	-4.5812780

C	3.0292880	4.1914530	-3.4689950
C	3.5596230	3.5430560	-2.3176740
H	1.9905170	4.4989790	-3.4725880
H	3.3770230	4.9022200	-5.4509180
H	5.7694380	4.1997980	-5.4885380
H	6.7386040	3.0233270	-3.5365730
H	6.4958460	2.0586820	-1.2899320
H	5.0741700	1.4846900	0.6288900
H	-0.2817550	0.8474010	-0.5310230
H	1.3025510	1.0455230	-1.2928900
C	1.9073470	-0.3287010	0.6381780
C	2.4127660	-0.9548820	1.9164440
C	1.4839880	-1.2280930	2.9173040
C	1.9203340	-1.6057490	4.2325800
C	1.0302890	-1.7365620	5.3345250
C	1.4999240	-2.0713250	6.5851700
C	2.8795430	-2.3042890	6.7999100
C	3.7678160	-2.1830470	5.7567890
C	3.3158980	-1.8212930	4.4591490
C	4.2099050	-1.7069470	3.3689230
C	3.7975210	-1.2901980	2.1164870
C	4.7971530	-1.3433890	1.0119630
C	4.5041550	-1.9761670	-0.2073740
C	5.4797570	-2.0788860	-1.2025620
C	6.7596230	-1.5643110	-1.0060670
C	7.0602570	-0.9456780	0.2074090
C	6.0936220	-0.8405100	1.2063220
H	6.3448180	-0.3521510	2.1424340
C	8.4083530	-0.3055890	0.4063650
F	9.3699750	-0.9163630	-0.3127870
F	8.3936860	0.9979210	0.0187670
F	8.7867690	-0.3197200	1.7012460
H	7.5097680	-1.6463010	-1.7831560
C	5.1514260	-2.8082540	-2.4815580
F	3.9163670	-2.4932640	-2.9256970
F	5.1776490	-4.1488150	-2.3028820
F	6.0272290	-2.5189560	-3.4664850
H	3.5215390	-2.4110500	-0.3731770
H	5.2463880	-1.9963270	3.5205820
H	4.8301520	-2.3555640	5.9104940
H	3.2334450	-2.5744080	7.7907970
H	0.8051390	-2.1569470	7.4157410
H	-0.0283100	-1.5593660	5.1840740
H	1.0976720	-0.9167390	0.1997310
H	2.6936030	-0.2230360	-0.1071680
H	0.8158630	-3.7440350	3.0636780
H	-0.6442190	-5.7090910	3.1022490
H	-3.0932640	-5.4811720	2.6885160
H	-4.0627380	-3.2490150	2.2231930
H	-3.8198420	-0.8293010	2.0725740
C	-2.6547110	1.5247060	1.9893320
C	-2.4057530	2.5573110	2.9028810
C	-3.1085040	3.7630310	2.8327930
C	-4.0730650	3.9571950	1.8478910
C	-4.3318760	2.9276120	0.9399730
C	-3.6455880	1.7175850	1.0119310

H	-3.8595080	0.9288000	0.3014990
C	-5.2884280	3.1844030	-0.1941960
F	-4.6701030	3.8902470	-1.1856820
F	-6.3449750	3.9299250	0.1996270
F	-5.7557750	2.0572700	-0.7479590
H	-4.6157780	4.8934290	1.7870740
C	-2.8546280	4.8238210	3.8691840
F	-1.5434930	4.8817220	4.2084940
F	-3.5390760	4.5870540	5.0109830
F	-3.2148200	6.0514970	3.4378810
H	-1.6768760	2.4156690	3.6952420
O	-2.1786700	0.2989270	-1.4994370
C	-2.1170810	-0.9121460	-1.6656150
C	-2.9434790	-1.8600920	-2.3775570
C	-2.1754500	-3.1661950	-2.3862570
H	-1.6588020	-3.3429010	-3.3431100
H	-2.7879210	-4.0409310	-2.1558570
N	-1.1942610	-2.9664250	-1.2989540
C	0.0032670	-3.6393000	-1.2020080
O	1.0176350	-3.1927700	-0.6828750
O	-0.1766760	-4.8721200	-1.7146940
C	0.8692080	-5.9087610	-1.6578640
C	2.0365480	-5.4954190	-2.5563120
H	2.7966370	-6.2844940	-2.5674720
H	1.6892020	-5.3423280	-3.5838780
H	2.4998460	-4.5743170	-2.2042820
C	1.2977650	-6.1576020	-0.2083610
H	0.4213620	-6.3616110	0.4159080
H	1.8264680	-5.2972060	0.2035430
H	1.9572900	-7.0319250	-0.1702690
C	0.1483250	-7.1339660	-2.2255220
H	-0.2151240	-6.9327670	-3.2381560
H	-0.7077750	-7.4046930	-1.5995720
H	0.8344230	-7.9865550	-2.2662170
O	-1.0606110	-1.5749270	-1.0333430
C	-3.7229310	-1.4627480	-3.5659630
C	-4.2068180	-0.1498890	-3.7408920
C	-4.9478520	0.1919280	-4.8659190
C	-5.2524890	-0.7467650	-5.8634840
C	-4.7886310	-2.0521860	-5.6808010
C	-4.0412770	-2.4062550	-4.5575160
H	-3.7207380	-3.4388520	-4.4501340
H	-5.0181790	-2.8115820	-6.4257440
C	-6.0490010	-0.3543800	-7.0859330
H	-5.4678900	0.2921810	-7.7577180
H	-6.3560050	-1.2339080	-7.6617380
H	-6.9555360	0.2006030	-6.8148150
H	-5.3066920	1.2146030	-4.9690860
H	-4.0023690	0.5970970	-2.9832790
C	-8.2330390	-3.3056160	1.1046070
C	-7.9699150	-1.9572550	1.7854930
H	-8.3449670	-4.1367580	1.8090070
H	-9.1165340	-3.3119970	0.4584560
H	-8.6722830	-1.1719650	1.4856320
H	-7.9838340	-1.9976730	2.8794720
N	-6.0881760	-2.5169120	0.4562280

```
Cl       -4.5390350       -2.1859480       -0.7896750
C        -6.5626540       -1.5550770        1.3149220
O        -5.9729900       -0.5437590        1.6757750
C        -6.9854350       -3.5544540        0.2362970
O        -6.8194510       -4.5057200       -0.5003800
147
R_MinorCl_2_MaruokaF3_TS_Diffuse.log
C         1.5935660        0.4046890       -2.4035460
C         2.6438180       -0.4903480       -2.3134410
C         2.4348900       -1.8882730       -2.2570130
C         3.5288310       -2.7825040       -2.1009700
C         3.3218320       -4.1409440       -2.0602970
C         2.0095160       -4.6606660       -2.1684350
C         0.9296470       -3.8201420       -2.3214170
C         1.1090330       -2.4093180       -2.3791450
C         0.0250730       -1.4780090       -2.4958470
C         0.2511710       -0.1063660       -2.4163090
C        -0.9619700        0.7868710       -2.3476200
H        -0.7092070        1.8436910       -2.4129130
H        -1.6660960        0.5481230       -3.1481630
N        -1.7945710        0.6666700       -1.0456290
C        -1.1459420        1.4689440        0.1036190
C        -1.2934600        2.9627240       -0.0389950
C        -2.5285950        3.5826460        0.1538040
C        -2.6575280        4.9935710       -0.0935160
C        -3.9109020        5.6696410       -0.0945930
C        -3.9908410        7.0196780       -0.3517430
C        -2.8232030        7.7736690       -0.6191470
C        -1.5977900        7.1508920       -0.6467180
C        -1.4814390        5.7553010       -0.4031390
C        -0.2269590        5.0981540       -0.4731900
C        -0.1383210        3.7362850       -0.3175260
H         0.8216680        3.2368760       -0.3718460
H         0.6656960        5.6852180       -0.6721920
H        -0.6951300        7.7143740       -0.8688590
H        -2.9005000        8.8396620       -0.8137070
H        -4.9602390        7.5103420       -0.3544530
H        -4.8171640        5.1072520        0.0986110
C        -3.6893040        2.7468720        0.5709340
C        -4.0120260        1.6207980       -0.1856800
C        -3.1782940        1.2486490       -1.3829310
H        -3.6721650        0.4939430       -1.9981500
H        -2.9760300        2.1251810       -2.0017710
C        -5.1562010        0.8473360        0.1298600
C        -5.9422440        1.1566780        1.2118760
C        -5.5951630        2.2311600        2.0695170
C        -6.3515040        2.5140060        3.2389320
C        -5.9766850        3.5227720        4.0945930
C        -4.8190670        4.2884630        3.8177820
C        -4.0754580        4.0524530        2.6836760
C        -4.4451240        3.0327170        1.7612850
H        -3.1854100        4.6405200        2.4945130
H        -4.5099110        5.0655460        4.5110210
H        -6.5569980        3.7248220        4.9904220
H        -7.2260060        1.9041130        3.4513630
H        -6.8263350        0.5651510        1.4352480
```

H	-5.4151130	0.0062530	-0.5029070
H	-0.0963160	1.1734020	0.1468890
H	-1.6366530	1.1072730	1.0091940
C	-1.9079590	-0.7883790	-0.5346860
C	-2.2816260	-1.7822460	-1.6099810
C	-1.3677070	-1.9981500	-2.6374410
C	-1.7487980	-2.7329750	-3.8099360
C	-0.9108320	-2.8519180	-4.9531130
C	-1.3338290	-3.5389420	-6.0693090
C	-2.6093880	-4.1522300	-6.0983820
C	-3.4445380	-4.0558470	-5.0095460
C	-3.0433120	-3.3400630	-3.8495030
C	-3.8729350	-3.2534760	-2.7065270
C	-3.5239220	-2.5088300	-1.5947230
C	-4.3834930	-2.6227970	-0.3829750
C	-3.8078770	-2.8888510	0.8722130
C	-4.6272860	-3.0719310	1.9774420
C	-6.0153950	-3.0067930	1.8780610
C	-6.5774150	-2.7559860	0.6286030
C	-5.7837080	-2.5688120	-0.4944840
H	-6.2682150	-2.3667520	-1.4438890
H	-2.7335610	-2.9887190	1.0003710
H	-4.7922010	-3.8328010	-2.6918340
H	-4.4265710	-4.5222410	-5.0216310
H	-2.9263450	-4.6962120	-6.9837200
H	-0.6831020	-3.6112660	-6.9360930
H	0.0676670	-2.3853880	-4.9423890
H	-0.9463430	-1.0285370	-0.0774450
H	-2.6551380	-0.7383550	0.2549080
H	-0.0690570	-4.2369570	-2.3777280
H	1.8508890	-5.7347980	-2.1197500
H	4.1616210	-4.8170550	-1.9273360
H	4.5238680	-2.3658850	-1.9853590
H	3.6653520	-0.1175390	-2.2904340
C	1.9340210	1.8449180	-2.5775320
C	1.3565440	2.6002270	-3.6135860
C	1.7340610	3.9220540	-3.8044340
C	2.6929510	4.5201840	-2.9933790
C	3.2828000	3.7576300	-1.9858240
C	2.9174440	2.4370670	-1.7663900
H	3.3946430	1.8849150	-0.9654540
H	0.6466080	2.1646530	-4.3088510
O	1.8275290	1.2428760	1.1303790
C	2.0596210	0.1253730	1.5714710
C	3.0869180	-0.4084980	2.4355590
C	2.6678940	-1.8249970	2.7751970
H	2.2314190	-1.9012130	3.7832310
H	3.4743580	-2.5574540	2.6905840
N	1.6466870	-2.1151550	1.7463460
C	0.6349590	-3.0344380	1.8949890
O	-0.4554170	-2.9692830	1.3423610
O	1.0849220	-4.0240050	2.6902500
C	0.3081890	-5.2534250	2.9300510
C	-0.9448610	-4.9176930	3.7437040
H	-1.4653080	-5.8428860	4.0160750
H	-0.6678230	-4.4008070	4.6688790

H	-1.6358420	-4.2854820	3.1846340
C	-0.0145970	-5.9473530	1.6029990
H	0.9019950	-6.1030800	1.0239740
H	-0.7100860	-5.3583000	1.0039350
H	-0.4622750	-6.9269450	1.8049540
C	1.2814440	-6.0971730	3.7576880
H	1.5575640	-5.5750180	4.6792060
H	2.1949860	-6.3014920	3.1906330
H	0.8161250	-7.0515550	4.0256120
O	1.1990820	-0.8995190	1.1590110
C	3.7387770	0.4165220	3.4710770
C	3.7677070	1.8241150	3.4099870
C	4.3952820	2.5681840	4.4030580
C	5.0305860	1.9568480	5.4938540
C	5.0086970	0.5600540	5.5502530
C	4.3803450	-0.1961750	4.5618720
H	4.4077630	-1.2797350	4.6377810
H	5.4953980	0.0496380	6.3790830
C	5.7339480	2.7785020	6.5488750
H	5.1369700	3.6488280	6.8474330
H	5.9366330	2.1864950	7.4478780
H	6.6976710	3.1609800	6.1856810
H	4.3936020	3.6543970	4.3279840
H	3.2856510	2.3268130	2.5800660
C	8.5842970	-1.4233460	-0.8719580
C	8.0231050	-0.3904270	-1.8563170
H	8.8725200	-2.3671000	-1.3472110
H	9.4518290	-1.0721300	-0.3045400
H	8.5264920	0.5810220	-1.8025620
H	8.0508600	-0.7071700	-2.9038370
N	6.3193090	-0.9760520	-0.3165720
Cl	4.7337520	-0.7096750	0.8745480
C	6.5593640	-0.2012330	-1.4248320
O	5.7538210	0.5183320	-2.0025020
C	7.4283700	-1.6953500	0.1090780
O	7.4847190	-2.4269870	1.0770130
F	-4.0911370	-3.3371920	3.1794130
F	-6.7907790	-3.1813020	2.9541700
F	-7.9153530	-2.6826220	0.5330810
F	1.1826730	4.6492450	-4.7927650
F	3.0404290	5.8022660	-3.1761210
F	4.2060610	4.3471450	-1.2131480

159
R_MinorCl_3_MaruokaCF3_TS_Diffuse.log

C	-3.2547100	2.2464610	0.8722590
C	-3.2313150	2.8843140	2.0968350
C	-2.1437070	3.6891570	2.5084750
C	-2.1093420	4.2883160	3.7960680
C	-1.0478330	5.0765420	4.1742030
C	0.0273610	5.2936380	3.2793960
C	0.0166610	4.7386380	2.0192840
C	-1.0735570	3.9318380	1.5905510
C	-1.1271040	3.3108630	0.2988200
C	-2.1315580	2.3981050	-0.0069460
C	-1.9989670	1.6068520	-1.2837340
H	-2.8711230	0.9823080	-1.4737710

H	-1.8550680	2.2712890	-2.1394440
N	-0.8036440	0.6318140	-1.3294000
C	-1.0712260	-0.6532300	-0.4971310
C	-2.0704740	-1.5866570	-1.1307990
C	-1.7414800	-2.3138250	-2.2753950
C	-2.7495610	-3.1165250	-2.9152170
C	-2.5546840	-3.7375200	-4.1817780
C	-3.5491500	-4.4882450	-4.7672480
C	-4.7941640	-4.6672620	-4.1183200
C	-5.0245280	-4.0674750	-2.9026640
C	-4.0266270	-3.2697910	-2.2797240
C	-4.2675440	-2.6184330	-1.0437270
C	-3.3254870	-1.7805810	-0.4990240
H	-3.5013920	-1.3031210	0.4575190
H	-5.2123740	-2.7794250	-0.5329270
H	-5.9815830	-4.1846910	-2.4002110
H	-5.5671270	-5.2695490	-4.5876320
H	-3.3778190	-4.9451170	-5.7380620
H	-1.6094830	-3.6058850	-4.6956860
C	-0.3580990	-2.2163370	-2.8189450
C	0.1703330	-0.9583720	-3.0995050
C	-0.6379960	0.2781040	-2.8217160
H	-0.1911440	1.1624630	-3.2813840
H	-1.6568960	0.1675610	-3.1990250
C	1.4434090	-0.8328270	-3.7057730
C	2.2068930	-1.9385070	-3.9824960
C	1.7575340	-3.2304140	-3.6116940
C	2.5717770	-4.3771500	-3.8126060
C	2.1537830	-5.6206420	-3.4013360
C	0.8994740	-5.7666320	-2.7622030
C	0.0772870	-4.6787760	-2.5724010
C	0.4655060	-3.3798460	-3.0062360
H	-0.8718970	-4.8074680	-2.0662470
H	0.5853860	-6.7452300	-2.4102100
H	2.7894740	-6.4890930	-3.5497320
H	3.5436090	-4.2467660	-4.2819750
H	3.1737330	-1.8267880	-4.4638090
H	1.8093770	0.1528400	-3.9691200
H	-1.3996310	-0.3235430	0.4914740
H	-0.0907260	-1.1249000	-0.3954600
C	0.5012990	1.2313270	-0.7493120
C	0.7751060	2.6544030	-1.1756240
C	-0.0955770	3.6438800	-0.7292810
C	-0.0271390	4.9776070	-1.2575510
C	-0.9804140	5.9882830	-0.9499900
C	-0.8892730	7.2413860	-1.5140870
C	0.1618920	7.5560230	-2.4080740
C	1.0979350	6.6011490	-2.7300990
C	1.0225600	5.2951480	-2.1757370
C	1.9909600	4.3076150	-2.4738020
C	1.9052060	3.0169480	-1.9873540
C	3.0805250	2.1241820	-2.2123640
C	3.7708690	1.5747480	-1.1195040
C	4.9415540	0.8411180	-1.3263980
C	5.4390110	0.6384890	-2.6136700
C	4.7617010	1.1924380	-3.6990840

C	3.5993990	1.9390740	-3.5006080
H	3.0936410	2.3804460	-4.3542000
C	5.2419240	0.9367190	-5.1014650
F	6.5549180	0.6378580	-5.1458260
F	4.5794620	-0.1073400	-5.6680010
F	5.0341910	2.0024460	-5.9057100
H	6.3516590	0.0757010	-2.7668840
C	5.6775400	0.2541770	-0.1448960
F	6.9731090	0.0067220	-0.4418750
F	5.1362590	-0.9148210	0.2599120
F	5.6604540	1.0875690	0.9190900
H	3.4185430	1.7537010	-0.1076440
H	2.8542370	4.5934800	-3.0690520
H	1.9085020	6.8293280	-3.4176660
H	0.2235810	8.5506050	-2.8405890
H	-1.6326070	7.9957980	-1.2722690
H	-1.7949840	5.7623120	-0.2716200
H	0.3990310	1.1564930	0.3331360
H	1.2814660	0.5312230	-1.0415930
H	0.8609350	4.8898000	1.3570890
H	0.8748740	5.8956810	3.5940560
H	-1.0246730	5.5237720	5.1639320
H	-2.9330530	4.1012700	4.4801460
H	-4.0853660	2.7875680	2.7614660
C	-4.5144390	1.5512330	0.4822810
C	-5.1971250	1.9043860	-0.6910830
C	-6.4264020	1.3223440	-1.0042720
C	-7.0022230	0.3824960	-0.1495210
C	-6.3284080	0.0315870	1.0197960
C	-5.0977420	0.6075960	1.3385200
H	-4.5423170	0.2836270	2.2131850
C	-6.8816740	-1.0389360	1.9223050
F	-6.2460100	-2.2261410	1.7250890
F	-8.1954410	-1.2575370	1.7130090
F	-6.7181480	-0.7297880	3.2259370
H	-7.9560530	-0.0694990	-0.3933910
C	-7.1652180	1.7709030	-2.2380420
F	-7.9035500	2.8788090	-2.0003040
F	-8.0091020	0.8213220	-2.6924800
F	-6.3130940	2.0767860	-3.2431780
H	-4.7805630	2.6551160	-1.3556000
O	1.9134640	-1.6883720	-0.0164850
C	2.2745500	-1.3618480	1.1055210
C	2.6071630	-2.0915000	2.3062640
C	3.2814400	-1.0925150	3.2248100
H	4.3800420	-1.1302910	3.1443170
H	3.0001300	-1.1920910	4.2755040
N	2.7608350	0.1852320	2.7122550
C	3.3061140	1.4293080	2.9252890
O	3.1429260	2.3882720	2.1854430
O	3.9736730	1.4077920	4.0970440
C	4.5790200	2.6304650	4.6515980
C	5.6480360	3.1659650	3.6929130
H	5.2000880	3.5359140	2.7697850
H	6.1965920	3.9827830	4.1757340
H	6.3622860	2.3747560	3.4417380

```
C        3.4855720        3.6619230        4.9471010
H        3.9271110        4.5326940        5.4453660
H        2.7290950        3.2337640        5.6136000
H        2.9985440        3.9899180        4.0271180
C        5.2156740        2.1242070        5.9482050
H        4.4571030        1.6907770        6.6076520
H        5.7036720        2.9512340        6.4747120
H        5.9668620        1.3568650        5.7354790
O        2.3549480        0.0126460        1.3596830
C        3.0608620       -3.4912110        2.2785790
C        2.7721490       -4.3537600        1.2018890
C        3.2131710       -5.6722040        1.2081460
C        3.9488400       -6.1997970        2.2793840
C        4.2316530       -5.3449170        3.3492780
C        3.7974520       -4.0204740        3.3528700
H        4.0286500       -3.3970830        4.2122960
H        4.7993100       -5.7196360        4.1988130
C        4.3922530       -7.6441220        2.2867850
H        5.2052430       -7.8100370        3.0019660
H        3.5699020       -8.3167400        2.5673100
H        4.7440500       -7.9632200        1.2982910
H        2.9793630       -6.3084170        0.3560250
H        2.2100190       -3.9752500        0.3565300
C       -3.6735100       -1.9483020        4.4049870
C       -3.0989600       -2.9306340        5.4305400
H       -4.4541590       -2.3760410        3.7682840
H       -4.0900900       -1.0394820        4.8530400
H       -3.1957360       -2.5946750        6.4677630
H       -3.5394660       -3.9312560        5.3738110
N       -1.3647070       -2.2206810        3.9664000
Cl        0.4708020       -2.1205500        3.1217570
C       -1.6042850       -3.0286470        5.0752520
O       -0.7829410       -3.6960670        5.6670740
C       -2.4719210       -1.5644170        3.5257380
O       -2.5404930       -0.7779810        2.5771710
159
R_MinorCl_4_MaruokaCF3_TS_Diffuse.log
C       -1.3641810        0.1920330        2.4658120
C       -2.2857310       -0.8366120        2.5229750
C       -1.8897840       -2.1956800        2.5936860
C       -2.8552470       -3.2385920        2.5925680
C       -2.4624800       -4.5515080        2.7137760
C       -1.0912500       -4.8749550        2.8411920
C       -0.1322550       -3.8860340        2.8442230
C       -0.5012460       -2.5180270        2.7209820
C        0.4510710       -1.4491930        2.6525210
C        0.0330240       -0.1446460        2.4126090
C        1.0875480        0.8756930        2.0759750
H        0.6777440        1.8801150        1.9820560
H        1.8858340        0.8951030        2.8219620
N        1.8175800        0.6301290        0.7322730
C        0.9207360        0.9833930       -0.4779440
C        0.7119200        2.4614450       -0.6673900
C        1.7545520        3.2787680       -1.1091320
C        1.5548760        4.7017720       -1.1688730
C        2.6100620        5.6223530       -1.4287260
```

C	2.3785760	6.9789190	-1.4610470
C	1.0788490	7.4948280	-1.2409600
C	0.0393330	6.6374500	-0.9684060
C	0.2464530	5.2321930	-0.9088600
C	-0.8141030	4.3465230	-0.5918070
C	-0.5806030	2.9996670	-0.4513010
H	-1.4075060	2.3366590	-0.2255810
H	-1.8150990	4.7371610	-0.4369040
H	-0.9604920	7.0204110	-0.7808410
H	0.9097900	8.5673800	-1.2763930
H	3.2025790	7.6608090	-1.6522020
H	3.6142230	5.2471660	-1.5898360
C	3.0558110	2.6514690	-1.4777090
C	3.6913470	1.8196380	-0.5563650
C	3.0526470	1.5472650	0.7778870
H	3.7485280	1.0629110	1.4663060
H	2.7035490	2.4759510	1.2338980
C	4.9722580	1.2819890	-0.8354740
C	5.5969640	1.5263680	-2.0329730
C	4.9423740	2.2766270	-3.0424950
C	5.5379180	2.4689640	-4.3183700
C	4.8756910	3.1523400	-5.3106840
C	3.5800990	3.6675310	-5.0679030
C	2.9833750	3.5163410	-3.8366520
C	3.6471090	2.8358180	-2.7767100
H	1.9867250	3.9081990	-3.6722520
H	3.0486180	4.1823990	-5.8631940
H	5.3368280	3.2861900	-6.2851180
H	6.5250040	2.0504520	-4.4981570
H	6.5905280	1.1294230	-2.2197940
H	5.4742850	0.6899320	-0.0784500
H	-0.0110630	0.4367060	-0.3282620
H	1.4264580	0.5361130	-1.3354410
C	2.2183850	-0.8487040	0.5143070
C	2.7706530	-1.5369270	1.7370320
C	1.9087760	-1.7394530	2.8110090
C	2.4133560	-2.2102410	4.0706150
C	1.6093720	-2.2992920	5.2407450
C	2.1456680	-2.7284870	6.4344250
C	3.5088150	-3.0995100	6.5218870
C	4.3153580	-3.0220770	5.4103440
C	3.7952810	-2.5690030	4.1683110
C	4.6040390	-2.5006730	3.0087090
C	4.1261740	-2.0105880	1.8082940
C	5.0076920	-2.1282250	0.6118250
C	4.5669360	-2.7794760	-0.5497790
C	5.4184360	-2.9411080	-1.6457060
C	6.7264810	-2.4659040	-1.6060080
C	7.1778300	-1.8293210	-0.4485800
C	6.3337360	-1.6687380	0.6496390
H	6.7026360	-1.1709110	1.5408420
C	8.5540650	-1.2188820	-0.4227600
F	9.0771920	-1.2047850	0.8200740
F	9.4136470	-1.8722520	-1.2277840
F	8.5171390	0.0726130	-0.8471360
H	7.3840660	-2.5939450	-2.4569690

C	4.8871410	-3.6455400	-2.8696630
F	4.4672650	-4.8933270	-2.5725830
F	5.8121770	-3.7434120	-3.8436740
F	3.8220470	-2.9856470	-3.3838230
H	3.5617050	-3.1892440	-0.6065130
H	5.6190820	-2.8859480	3.0620470
H	5.3644410	-3.3016500	5.4663260
H	3.9152400	-3.4415580	7.4694040
H	1.5167320	-2.7822220	7.3183750
H	0.5643650	-2.0169870	5.1881190
H	1.3091960	-1.3372660	0.1422340
H	2.9418540	-0.8079240	-0.2993510
H	0.9154370	-4.1543230	2.9225500
H	-0.7900690	-5.9158300	2.9244450
H	-3.2038420	-5.3448720	2.6935930
H	-3.8979030	-2.9719000	2.4562300
H	-3.3492630	-0.6177450	2.5326950
C	-1.8408630	1.5957360	2.6369650
C	-1.2140960	2.4299170	3.5754200
C	-1.6746070	3.7262600	3.8101830
C	-2.7854480	4.2109540	3.1253030
C	-3.4285620	3.3827870	2.2027040
C	-2.9694900	2.0876040	1.9564310
H	-3.4726680	1.4722450	1.2184910
C	-4.5976450	3.9451140	1.4302830
F	-4.1802060	4.6613990	0.3510570
F	-5.3152330	4.8055180	2.1944820
F	-5.4341820	2.9972320	0.9907770
H	-3.1501700	5.2141710	3.3110560
C	-1.0032230	4.5660410	4.8632160
F	-1.2171440	5.8847840	4.6728640
F	0.3380140	4.3692100	4.8727020
F	-1.4467600	4.2654580	6.1047360
H	-0.3734620	2.0573910	4.1526000
O	-5.2994900	-1.4957580	1.3630040
C	-5.2141960	-1.1908560	0.1862570
C	-5.5396480	-1.8715100	-1.0576760
C	-5.5321810	-0.7915410	-2.1241020
H	-6.5376010	-0.3773110	-2.3112610
H	-5.1008060	-1.1085080	-3.0759670
N	-4.6519600	0.2014730	-1.5144970
C	-4.2621850	1.4227470	-1.9692070
O	-3.6573720	2.2527620	-1.3037180
O	-4.6150110	1.5276590	-3.2726210
C	-4.2605480	2.7187050	-4.0598930
C	-4.8409600	2.3872870	-5.4374050
H	-5.9213680	2.2240710	-5.3715070
H	-4.6564040	3.2139250	-6.1317480
H	-4.3795990	1.4816020	-5.8441330
C	-4.9440260	3.9555340	-3.4654560
H	-4.7475100	4.8258350	-4.1023070
H	-4.5807900	4.1637490	-2.4579030
H	-6.0283320	3.8044080	-3.4233470
C	-2.7375980	2.8689590	-4.1374940
H	-2.3133700	3.1052020	-3.1607690
H	-2.4847410	3.6743270	-4.8368650

H	-2.2845350	1.9422780	-4.5068620
O	-4.6602990	0.0631810	-0.1066460
C	-6.5397310	-2.9512280	-1.1276770
C	-6.9587240	-3.6735920	0.0064520
C	-7.9024530	-4.6898900	-0.1037910
C	-8.4672920	-5.0416540	-1.3374260
C	-8.0517070	-4.3238810	-2.4641220
C	-7.1088970	-3.3032540	-2.3650280
H	-6.8042400	-2.7829540	-3.2690510
H	-8.4697230	-4.5678420	-3.4389090
C	-9.4635820	-6.1719470	-1.4486110
H	-8.9601040	-7.1447040	-1.5370270
H	-10.1122730	-6.2248490	-0.5665300
H	-10.1033720	-6.0577900	-2.3306930
H	-8.2109390	-5.2229890	0.7939970
H	-6.5499480	-3.4168440	0.9762760
C	0.6464220	-3.9671240	-1.6772680
C	-0.0322670	-5.2060480	-2.2685820
H	1.1812570	-4.1594700	-0.7408260
H	1.3524940	-3.4890210	-2.3643760
H	0.3048700	-5.4558910	-3.2792530
H	0.0881540	-6.1061190	-1.6571170
N	-1.6867620	-3.5606110	-1.7790120
Cl	-3.4744410	-2.7834900	-1.3835210
C	-1.5264670	-4.8354540	-2.3113270
O	-2.4078870	-5.5499280	-2.7399780
C	-0.5148230	-3.0005290	-1.3798010
O	-0.3651660	-1.8943080	-0.8554470

147

R_MinorCl_4_MaruokaF3_TS_Diffuse.log

C	0.1550060	-3.4285510	1.0990180
C	-0.0305700	-4.4389660	0.1741290
C	0.9618650	-4.8078300	-0.7612770
C	0.7206920	-5.8243350	-1.7244760
C	1.7056600	-6.2015630	-2.6059070
C	2.9693180	-5.5640130	-2.5680150
C	3.2295970	-4.5685280	-1.6520730
C	2.2388410	-4.1654840	-0.7119240
C	2.4330380	-3.1140910	0.2468540
C	1.3923870	-2.6968890	1.0727720
C	1.6315930	-1.4657780	1.9158700
H	0.8319290	-1.2865130	2.6328700
H	2.5746830	-1.5491100	2.4608230
N	1.7413280	-0.1472170	1.1160480
C	0.3243190	0.3840280	0.7543140
C	-0.3940590	0.9989090	1.9267850
C	-0.0080930	2.2434700	2.4247500
C	-0.6604720	2.7729940	3.5910830
C	-0.2149970	3.9441920	4.2682990
C	-0.8668150	4.4106660	5.3871480
C	-2.0049350	3.7377640	5.8931540
C	-2.4504420	2.5918610	5.2789170
C	-1.7881100	2.0708260	4.1339330
C	-2.2140940	0.8666200	3.5237030
C	-1.5148320	0.3293320	2.4717810
H	-1.8714860	-0.5803950	2.0079560

H	-3.1078760	0.3684250	3.8866420
H	-3.3143250	2.0559830	5.6637920
H	-2.5161490	4.1240750	6.7705960
H	-0.5028750	5.3035700	5.8882210
H	0.6581140	4.4708610	3.9013440
C	1.1216890	2.9581010	1.7744520
C	2.3376090	2.2921530	1.6267200
C	2.4643810	0.8516520	2.0408350
H	3.5070700	0.5276300	2.0612570
H	2.0267060	0.6876100	3.0276450
C	3.4789470	2.9843640	1.1523070
C	3.4029310	4.3055000	0.7866010
C	2.1604170	4.9895760	0.8128820
C	2.0441200	6.3327760	0.3652990
C	0.8214590	6.9626050	0.3445840
C	-0.3417230	6.2657290	0.7508190
C	-0.2635670	4.9689080	1.2077240
C	0.9919500	4.3025450	1.2846250
H	-1.1730440	4.4394270	1.4673130
H	-1.3118300	6.7496320	0.6899030
H	0.7419560	7.9882010	-0.0055870
H	2.9391630	6.8472070	0.0240520
H	4.2919810	4.8338040	0.4512280
H	4.4280460	2.4636480	1.1021080
H	-0.2176190	-0.4715810	0.3490310
H	0.4708430	1.0836110	-0.0693700
C	2.4958970	-0.3173420	-0.2167700
C	3.8030310	-1.0674190	-0.0934870
C	3.7486220	-2.4065290	0.2868330
C	4.9397620	-3.1060410	0.6723560
C	4.9229940	-4.4181040	1.2216960
C	6.0915520	-5.0339410	1.6112770
C	7.3372380	-4.3777960	1.4613140
C	7.3883390	-3.1088280	0.9326460
C	6.1986640	-2.4401100	0.5368100
C	6.2282670	-1.1482730	-0.0402020
C	5.0752390	-0.4581470	-0.3680190
C	5.2138420	0.8203400	-1.1182340
C	4.4866130	1.0397850	-2.3023580
C	4.6874200	2.2066870	-3.0294200
C	5.6005990	3.1733040	-2.6133640
C	6.3284300	2.9415860	-1.4493530
C	6.1512820	1.7829390	-0.7071480
H	6.7328380	1.6535900	0.1994890
H	3.7977880	0.3029250	-2.7084130
H	7.1945310	-0.7075820	-0.2702930
H	8.3395510	-2.5959740	0.8144290
H	8.2508920	-4.8790770	1.7678730
H	6.0583940	-6.0319900	2.0387850
H	3.9743590	-4.9289620	1.3440170
H	1.7988930	-0.8332920	-0.8683230
H	2.6171000	0.6925510	-0.6010510
H	4.1971850	-4.0809640	-1.6547500
H	3.7391980	-5.8551780	-3.2774170
H	1.5118870	-6.9744210	-3.3438910
H	-0.2640620	-6.2796610	-1.7632740

H	-0.9620840	-4.9962840	0.1798800
C	-0.8986320	-3.2504670	2.1375900
C	-0.5533470	-3.2377300	3.5004940
C	-1.5480610	-3.1871950	4.4664080
C	-2.8947400	-3.1726290	4.1145860
C	-3.2322360	-3.2079420	2.7623950
C	-2.2556240	-3.2316190	1.7744120
H	-2.5643570	-3.2063410	0.7349580
H	0.4775250	-3.3159860	3.8290030
O	-4.2047140	-0.6384100	1.1648630
C	-4.1886460	0.0521950	0.1617360
C	-4.4888490	-0.2371880	-1.2373890
C	-4.5935320	1.1154440	-1.9105100
H	-5.6361170	1.4702470	-1.9692130
H	-4.1563870	1.1532480	-2.9104660
N	-3.7931280	1.9561180	-1.0131910
C	-3.7740790	3.3309040	-1.0031560
O	-3.3888980	4.0220360	-0.0770680
O	-4.1976920	3.7561250	-2.2198640
C	-4.0687200	5.1631690	-2.6243770
C	-4.6346500	5.1524850	-4.0472250
H	-5.6759470	4.8141700	-4.0459000
H	-4.5977730	6.1603790	-4.4743930
H	-4.0544560	4.4812750	-4.6885890
C	-4.9174100	6.0549480	-1.7110750
H	-4.9092920	7.0833660	-2.0906130
H	-4.5340840	6.0481270	-0.6899360
H	-5.9556910	5.7054040	-1.6990510
C	-2.5912110	5.5668570	-2.6314300
H	-2.1686850	5.5334120	-1.6259170
H	-2.4887950	6.5853390	-3.0236720
H	-2.0157500	4.8932880	-3.2754820
O	-3.7794440	1.3781960	0.2883390
C	-5.4245280	-1.3084930	-1.6296270
C	-5.7761890	-2.3687150	-0.7709910
C	-6.6579750	-3.3607540	-1.1890300
C	-7.2247890	-3.3542850	-2.4711070
C	-6.8762750	-2.3011180	-3.3232400
C	-5.9958920	-1.3013580	-2.9152740
H	-5.7431450	-0.5095460	-3.6153150
H	-7.2990210	-2.2605270	-4.3254240
C	-8.1547160	-4.4566750	-2.9213550
H	-7.5973640	-5.3460370	-3.2469240
H	-8.8228440	-4.7754430	-2.1125990
H	-8.7766430	-4.1372030	-3.7648190
H	-6.9159300	-4.1608770	-0.4970700
H	-5.3684860	-2.3979460	0.2316620
C	1.2764840	-1.2905770	-4.0539640
C	0.6691240	-2.6732460	-4.3025040
H	2.2832510	-1.3172300	-3.6012180
H	1.3361070	-0.6638720	-4.9537590
H	0.3545930	-2.8311120	-5.3396790
H	1.3272630	-3.5042270	-4.0338860
N	-0.7135000	-1.4730040	-2.7715630
Cl	-2.4222310	-0.9216600	-1.9140560
C	-0.5807380	-2.7055390	-3.4030470

O	-1.3135890	-3.6659750	-3.2654870
C	0.3244090	-0.6238900	-3.0434650
O	0.5313620	0.4789490	-2.5420450
F	-4.5292520	-3.2267980	2.4431820
F	-3.8440970	-3.1391950	5.0567230
F	-1.2247670	-3.1801130	5.7703310
F	4.0172310	2.4177420	-4.1679790
F	5.7803190	4.2943720	-3.3187060
F	7.2004140	3.8787160	-1.0439160

177
S_MajorCf00_ConstOpt.log

C	2.3896030	-1.2113990	2.6824430
C	2.9828710	-0.1758590	3.3738720
C	2.2034170	0.7696830	4.1051970
C	2.8168420	1.8722700	4.7812530
C	2.0520200	2.7570110	5.4986190
C	0.6376280	2.5934880	5.5688670
C	0.0255370	1.5434440	4.9320410
C	0.7981770	0.5924240	4.1904770
C	0.2045460	-0.5178530	3.5001800
C	0.9590810	-1.3187250	2.6557330
C	0.2614330	-2.3271660	1.7961600
H	0.9567330	-2.9546740	1.2189170
H	-0.3749950	-2.9852500	2.4104090
N	-0.6817400	-1.7035410	0.7375510
O	-0.9968000	2.7363410	2.2878930
C	-1.2746680	3.0670190	1.1533970
C	-0.7961430	4.1435390	0.2879640
C	-1.6233380	4.1287300	-0.9613830
H	-2.2953770	4.9979370	-0.9992960
H	-1.0226330	4.0874840	-1.8741140
N	-2.4221700	2.8938170	-0.8298410
C	-2.4808770	1.9044040	-1.8079430
O	-2.5968450	0.7121380	-1.5621710
O	-2.5200870	2.5061980	-2.9948290
C	-2.6652180	1.7574230	-4.2658900
C	-4.0518250	1.1119480	-4.3026680
H	-4.1496290	0.3616270	-3.5137080
H	-4.1972520	0.6182250	-5.2702140
H	-4.8346400	1.8676350	-4.1803750
C	-1.5362220	0.7401420	-4.4188450
H	-1.5805520	0.3030440	-5.4235490
H	-0.5716340	1.2327500	-4.2852060
H	-1.6343420	-0.0698670	-3.6908620
C	-2.5322670	2.8721650	-5.3057180
H	-1.5530340	3.3503460	-5.2174230
H	-2.6345520	2.4547820	-6.3130180
H	-3.3104290	3.6290620	-5.1627370
O	-2.2433740	2.3468040	0.4715750
C	-0.0665910	5.3077150	0.7417180
C	0.0195790	5.6405050	2.1085110
C	0.6621000	6.8075860	2.5081360
C	1.2607510	7.6663860	1.5765050
C	1.1895190	7.3202120	0.2174440
C	0.5365050	6.1694540	-0.1994930
H	0.5340930	5.9045850	-1.2519600

H	1.6624920	7.9603580	-0.5236190
C	1.9888120	8.9128790	2.0164250
H	1.7507020	9.7657520	1.3700410
H	3.0766520	8.7702350	1.9701270
H	1.7347230	9.1858610	3.0456950
H	0.7003330	7.0588870	3.5656730
H	-0.4300130	4.9838250	2.8443490
C	0.1449590	-1.1100310	-0.4469510
C	0.7915890	-2.2157890	-1.2310190
C	0.0507110	-3.0905370	-2.0119180
C	0.6912660	-4.2293030	-2.6128970
C	-0.0452110	-5.2693480	-3.2668590
C	0.6022200	-6.3434920	-3.8230500
C	2.0230080	-6.4421330	-3.7614450
C	2.7554240	-5.4690900	-3.1303900
C	2.1040850	-4.3447310	-2.5294250
C	2.8543670	-3.3374630	-1.8435170
C	2.2141420	-2.3140420	-1.1984370
H	2.7739150	-1.5152710	-0.6786720
H	3.9428920	-3.3996890	-1.8476520
H	3.8420070	-5.5339700	-3.0721680
H	2.5101750	-7.3021680	-4.2197100
H	0.0431930	-7.1368350	-4.3179690
H	-1.1329590	-5.1958620	-3.3112620
C	-1.3937570	-2.8283860	-2.1933350
C	-2.1902940	-2.6711110	-1.0680730
C	-1.6052300	-2.8604040	0.2991020
H	-2.3706420	-2.9474550	1.0832110
H	-0.9863100	-3.7720440	0.3186630
C	-3.5851850	-2.4180950	-1.2044720
C	-4.1580380	-2.3212540	-2.4454910
C	-3.3585890	-2.4422770	-3.6251330
C	-3.9455980	-2.3179310	-4.9256410
C	-3.1646240	-2.3885400	-6.0506760
C	-1.7564100	-2.5775460	-5.9344570
C	-1.1720830	-2.7121700	-4.7007530
C	-1.9621320	-2.6723700	-3.5053730
H	-0.0907400	-2.8401750	-4.6146690
H	-1.1548750	-2.6090650	-6.8424210
H	-3.6017260	-2.2970780	-7.0444500
H	-5.0214410	-2.1627240	-4.9989180
H	-5.2273340	-2.1379190	-2.5527670
H	-4.1895260	-2.3035870	-0.3081210
H	0.8831220	-0.4253210	-0.0077490
H	-0.5746600	-0.5357180	-1.0494750
C	-1.5327120	-0.5140920	1.2645090
C	-2.1040100	-0.7920400	2.6254940
C	-1.2300970	-0.8341870	3.7029720
C	-1.6802990	-1.2192160	5.0128280
C	-0.7876950	-1.4073030	6.1174340
C	-1.2646990	-1.7921150	7.3448930
C	-2.6596480	-2.0103500	7.5436720
C	-3.5396690	-1.8421820	6.5051390
C	-3.0655970	-1.4459270	5.2134790
C	-3.9652130	-1.2566550	4.1234750
C	-3.5108910	-0.9301160	2.8616930

C	-4.5508140	-0.6558260	1.8421240
C	-4.6740890	0.6267330	1.2765540
C	-5.7710540	0.9186630	0.4593550
C	-6.7120690	-0.0740330	0.1410240
C	-6.5697730	-1.3538480	0.6851300
C	-5.5050210	-1.6408350	1.5531340
H	-5.4168160	-2.6303410	2.0107930
C	-7.5606940	-2.4445870	0.3811170
F	-8.2755080	-2.8428070	1.4471100
F	-8.4921540	-2.1640100	-0.5428420
F	-7.0003740	-3.5804380	-0.0727750
H	-7.5503540	0.1603980	-0.5231160
C	-5.9831710	2.3085100	-0.0876060
F	-7.2641690	2.7227930	-0.0226170
F	-5.6730910	2.4404790	-1.3857110
F	-5.3124900	3.2938170	0.5229500
H	-3.9093380	1.3899610	1.4899760
H	-5.0358750	-1.3555750	4.3179740
H	-4.6081030	-2.0057860	6.6432950
H	-3.0084140	-2.3118000	8.5316500
H	-0.5866910	-1.9366030	8.1863890
H	0.2812020	-1.2404990	5.9685760
H	-0.8546860	0.3479410	1.3017210
H	-2.2803970	-0.3244790	0.4853270
H	-1.0607410	1.4370610	4.9544700
H	0.0518520	3.3242770	6.1254770
H	2.5089580	3.5999980	6.0162780
H	3.8965160	1.9993410	4.7006560
H	4.0697270	-0.0632620	3.3763800
C	3.2743510	-2.2502510	2.1053480
C	3.1320110	-3.5818400	2.5135490
C	4.0315310	-4.5571370	2.0533540
C	5.0970650	-4.2011090	1.2228980
C	5.2398030	-2.8617010	0.8218540
C	4.3271520	-1.8908060	1.2422830
H	4.3865380	-0.8356700	0.8999790
C	6.4261630	-2.4925810	-0.0302490
F	6.4101710	-1.2791580	-0.5913320
F	6.6420270	-3.3233480	-1.0676040
F	7.5887500	-2.5251080	0.6472420
H	5.8211630	-4.9509790	0.8899370
C	3.8301690	-5.9798730	2.4969840
F	4.0225900	-6.1752810	3.8129600
F	4.6125320	-6.9040670	1.9193610
F	2.5852310	-6.4465080	2.2847230
H	2.3321140	-3.8542730	3.2064370
C	5.2562190	4.6851560	0.9842870
C	4.4912050	4.9260310	2.1263380
C	3.3402050	4.1726980	2.3706460
C	2.9475180	3.1746490	1.4802250
C	3.7295530	2.9369860	0.3478900
C	4.8758300	3.6878650	0.0860500
H	5.4557430	3.4782520	-0.8054220
S	3.3162260	1.5914080	-0.7729580
O	3.0107360	0.4213070	0.0978020
O	4.4626890	1.4411720	-1.6876760

N	1.9117300	1.9160470	-1.5774410
F	0.6153970	2.7725360	-0.4408630
S	1.9354910	3.0640050	-2.7948560
O	2.6224460	4.3122320	-2.4146720
O	0.5388310	3.1607460	-3.2663290
C	2.8772570	2.3320590	-4.1497260
C	3.8709550	3.0925170	-4.7625070
C	4.5543600	2.5624750	-5.8581140
C	4.2449720	1.2850020	-6.3271200
C	3.2494120	0.5304110	-5.7000550
C	2.5590320	1.0522620	-4.6080640
H	1.7951220	0.4699340	-4.1023630
H	3.0140770	-0.4679430	-6.0599160
H	4.7823390	0.8735860	-7.1776500
H	5.3323420	3.1472400	-6.3416260
H	4.1010830	4.0781500	-4.3737170
H	2.0433010	2.6023670	1.6521480
H	2.7345220	4.3695430	3.2506490
H	4.7897360	5.7030780	2.8259520
H	6.1495250	5.2731820	0.7892170

177
S_MajorCf02_B3LYP_TS.log

C	3.5950990	-0.3125860	2.1238200
C	3.7227150	0.6193710	3.1340350
C	2.7328510	0.7564790	4.1507810
C	2.8409360	1.7635880	5.1624630
C	1.8624120	1.8933530	6.1150240
C	0.7259890	1.0321590	6.1011350
C	0.6053380	0.0531910	5.1478340
C	1.6218990	-0.1251530	4.1537400
C	1.5479670	-1.1404530	3.1395110
C	2.4571830	-1.1834130	2.0913340
C	2.2539180	-2.2127260	1.0205510
H	3.0877400	-2.2613350	0.3046850
H	2.1266400	-3.2118390	1.4691230
N	0.9971620	-1.9744810	0.1482230
O	2.3113390	2.0328140	0.2065390
C	1.4384650	2.5331230	0.8974530
C	0.9118610	3.8958720	0.9686810
C	0.0265610	3.9773550	2.1715580
H	0.4627960	4.6109820	2.9567490
H	-0.9776640	4.3342090	1.9226190
N	-0.0329160	2.5827510	2.6452220
C	-1.2083070	1.9269750	3.0092110
O	-1.3834800	0.7272250	2.8994030
O	-1.9999520	2.8273940	3.5989630
C	-3.3060630	2.4592340	4.1902790
C	-3.8756140	3.8235000	4.5809530
H	-3.9918220	4.4466650	3.6891860
H	-3.2141580	4.3371300	5.2872800
H	-4.8563680	3.6976310	5.0524440
C	-3.0489840	1.5820770	5.4200490
H	-2.5948920	0.6319580	5.1300430
H	-2.3886500	2.0950720	6.1280770
H	-3.9982950	1.3745090	5.9273320
C	-4.2026240	1.7736910	3.1587360

H	-5.1983210	1.6327360	3.5977360
H	-4.3014170	2.3969610	2.2669090
H	-3.8034400	0.8049960	2.8646800
O	0.8285580	1.7650890	1.8583300
C	1.4876500	5.0457350	0.3123990
C	2.4852570	4.9191760	-0.6811110
C	3.0207810	6.0464160	-1.2884420
C	2.5950900	7.3399480	-0.9443360
C	1.5937700	7.4637300	0.0294900
C	1.0473860	6.3446250	0.6461320
H	0.2600100	6.4772520	1.3815680
H	1.2326310	8.4522780	0.3029600
C	3.2116360	8.5567630	-1.5892710
H	4.1508060	8.8358550	-1.0919860
H	2.5443060	9.4229970	-1.5346590
H	3.4491520	8.3749390	-2.6434240
H	3.7866490	5.9238060	-2.0517950
H	2.8207400	3.9299330	-0.9657110
C	1.3021910	-0.8579660	-0.8962720
C	2.2344390	-1.3653130	-1.9617240
C	1.8213910	-2.2921270	-2.9061070
C	2.7707170	-2.8561320	-3.8281900
C	2.4400470	-3.9381240	-4.7061790
C	3.3773940	-4.4558520	-5.5642410
C	4.6992280	-3.9238570	-5.5972620
C	5.0499260	-2.8942610	-4.7610310
C	4.0940730	-2.3426520	-3.8498330
C	4.4491700	-1.2791080	-2.9605480
C	3.5472080	-0.8132190	-2.0415840
H	3.7937780	0.0210740	-1.3826340
H	5.4474170	-0.8470270	-3.0332320
H	6.0583200	-2.4816090	-4.7735390
H	5.4202720	-4.3511550	-6.2935890
H	3.1274230	-5.2803060	-6.2315420
H	1.4287590	-4.3482140	-4.6794820
C	0.4003400	-2.7006200	-2.9175960
C	-0.1546490	-3.1974850	-1.7476760
C	0.6870230	-3.3326740	-0.5132120
H	0.2081940	-3.9460970	0.2631990
H	1.6604520	-3.7864950	-0.7621250
C	-1.5056150	-3.6466060	-1.7342330
C	-2.2820370	-3.5610720	-2.8601680
C	-1.7558120	-2.9969280	-4.0644720
C	-2.5663660	-2.8798800	-5.2388470
C	-2.0606210	-2.3064060	-6.3776220
C	-0.7264140	-1.8045830	-6.3960480
C	0.0736910	-1.9135510	-5.2870310
C	-0.4115000	-2.5397920	-4.0932200
H	1.0886130	-1.5109300	-5.2961840
H	-0.3580830	-1.3276610	-7.3039480
H	-2.6682410	-2.2171390	-7.2776260
H	-3.5926950	-3.2451050	-5.2032130
H	-3.3157630	-3.9098990	-2.8523020
H	-1.9094510	-4.0705530	-0.8185110
H	1.7306800	-0.0220140	-0.3257990
H	0.3349880	-0.5395890	-1.3091030

C	-0.2524250	-1.4874120	0.9515020
C	-0.4463860	-2.2982390	2.2036920
C	0.4847420	-2.1698500	3.2270360
C	0.4509710	-3.0207300	4.3851880
C	1.4954220	-3.0447440	5.3663380
C	1.4152750	-3.8778160	6.4530810
C	0.2880570	-4.7340620	6.6282400
C	-0.7238160	-4.7400990	5.7030200
C	-0.6571070	-3.8868880	4.5543280
C	-1.6891640	-3.8831580	3.5733980
C	-1.5957620	-3.1281030	2.4187230
C	-2.7647670	-3.2278110	1.5115790
C	-3.5747360	-2.1191180	1.2027190
C	-4.7925230	-2.3296290	0.5437900
C	-5.1752200	-3.6124150	0.1192890
C	-4.3388240	-4.7009280	0.3809810
C	-3.1498410	-4.5139270	1.0989160
H	-2.5189840	-5.3721880	1.3471790
C	-4.6988430	-6.0906290	-0.0646230
F	-5.7959040	-6.2102920	-0.8275560
F	-3.7422200	-6.6931740	-0.7954830
F	-4.9167660	-6.9508560	0.9453010
H	-6.1266390	-3.7531590	-0.4042560
C	-5.7747770	-1.2074340	0.3073780
F	-6.9356650	-1.4132360	0.9693370
F	-6.1677190	-1.1007970	-0.9748810
F	-5.4222600	0.0247930	0.6610530
H	-3.2565010	-1.0942300	1.4767270
H	-2.5767290	-4.4924020	3.7642370
H	-1.5904260	-5.3898590	5.8222630
H	0.2500400	-5.3786800	7.5063240
H	2.2090670	-3.8987220	7.1997460
H	2.3588950	-2.3900720	5.2365870
H	-0.0887480	-0.4295840	1.1943630
H	-1.0894120	-1.5550750	0.2456750
H	-0.2839060	-0.5811320	5.1098080
H	-0.0499680	1.1765450	6.8522990
H	1.9293150	2.6606890	6.8860050
H	3.7033600	2.4283740	5.1497640
H	4.5926570	1.2789520	3.1718740
C	4.7170890	-0.3904970	1.1600750
C	5.4678100	-1.5645300	1.0324690
C	6.5878110	-1.5901720	0.1857650
C	6.9845380	-0.4397410	-0.5009530
C	6.2339530	0.7400300	-0.3596450
C	5.0974720	0.7669420	0.4523790
H	4.4701550	1.6702590	0.5423570
C	6.6903890	1.9690580	-1.1016730
F	6.6924280	1.8246560	-2.4385980
F	7.9520080	2.3362830	-0.8158250
F	5.9806250	3.0876640	-0.9006410
H	7.8727500	-0.4468720	-1.1406860
C	7.3588610	-2.8767560	0.0569440
F	6.5917510	-3.9493960	-0.2092490
F	8.0248480	-3.2257290	1.1702830
F	8.2950880	-2.9150220	-0.9034510

```
H        5.1888700       -2.4509900        1.6085340
C       -3.7597670        0.4392810       -5.0821080
C       -5.1364220        0.6686620       -5.0538060
C       -5.7564220        1.0494370       -3.8612300
C       -5.0095420        1.2026080       -2.6935550
C       -3.6323180        0.9680740       -2.7347670
C       -3.0002130        0.5864960       -3.9208860
H       -1.9306640        0.4026970       -3.9204610
S       -2.6296160        1.1544040       -1.2468720
O       -1.3316950        0.5225840       -1.5554950
O       -3.3854070        0.5735850       -0.1137110
N       -2.3108860        2.7639480       -1.1300460
F       -0.6196370        3.1352990       -0.2441550
S       -3.3709590        3.8412070       -0.4446160
O       -2.9912540        4.1830470        0.9436380
O       -4.7848070        3.4687630       -0.6526360
C       -3.0353710        5.2838380       -1.4672840
C       -1.7264570        5.6133110       -1.8271120
C       -1.5068710        6.7687170       -2.5762980
C       -2.5796390        7.5809090       -2.9550090
C       -3.8820470        7.2358460       -2.5897730
C       -4.1173660        6.0799150       -1.8430790
H       -5.1230050        5.7837130       -1.5639770
H       -4.7181520        7.8614650       -2.8913460
H       -2.4006750        8.4791880       -3.5408000
H       -0.4929380        7.0323270       -2.8650890
H       -0.9144990        4.9579670       -1.5345030
H       -5.4754240        1.5270360       -1.7717970
H       -6.8277610        1.2301640       -3.8371300
H       -5.7258300        0.5536790       -5.9597430
H       -3.2724420        0.1413180       -6.0072640
177
S_MajorCf04_B3LYP_TS.log
C       -3.5707570        1.0899820        1.4143620
C       -3.8874370        0.4682550        2.6026940
C       -3.0226070        0.5539660        3.7360780
C       -3.3127670       -0.1549920        4.9446730
C       -2.4758040       -0.0494000        6.0270830
C       -1.3103860        0.7679010        5.9581140
C       -1.0143180        1.4643620        4.8130560
C       -1.8712760        1.3802680        3.6690580
C       -1.5910750        2.0726470        2.4400870
C       -2.3396340        1.8209410        1.2997410
C       -1.8762630        2.3705780       -0.0136410
H       -2.5709350        2.1498010       -0.8383150
H       -1.7351060        3.4623190        0.0431000
N       -0.5224690        1.7923200       -0.4782010
O        0.5030640       -2.3333360       -2.8273580
C       -0.0031850       -3.0347300       -1.9817250
C        0.4526480       -4.2850350       -1.3417940
C       -0.6469980       -4.7688050       -0.4482690
H       -1.0150300       -5.7592640       -0.7436910
H       -0.3338440       -4.7784140        0.5994810
N       -1.7105060       -3.7735030       -0.6616870
C       -2.6255260       -3.3619660        0.2898860
O       -3.1658280       -2.2669650        0.2911250
```

O	-2.8822700	-4.4113730	1.0835890
C	-3.9024110	-4.3543710	2.1479330
C	-5.2841710	-4.1737260	1.5121530
H	-6.0590430	-4.2535840	2.2835080
H	-5.4695210	-4.9565480	0.7668550
H	-5.3703450	-3.2012110	1.0279970
C	-3.5701830	-3.2504800	3.1552310
H	-2.5486090	-3.3737850	3.5239080
H	-3.6468220	-2.2631150	2.6984910
H	-4.2667200	-3.3147950	3.9992760
C	-3.7729560	-5.7348740	2.7941380
H	-3.9653430	-6.5268320	2.0620120
H	-2.7658160	-5.8735870	3.1995120
H	-4.4950130	-5.8367360	3.6112900
O	-1.2476670	-2.7149630	-1.4854600
C	1.5237160	-5.1181010	-1.8311700
C	2.3728370	-4.6991990	-2.8802510
C	3.3863880	-5.5317760	-3.3384700
C	3.6052810	-6.7999070	-2.7779910
C	2.7709390	-7.2075870	-1.7251460
C	1.7511420	-6.3896830	-1.2600880
H	1.1341350	-6.7297620	-0.4341030
H	2.9292650	-8.1790820	-1.2630650
C	4.6888000	-7.7084210	-3.3035360
H	5.4931760	-7.1395500	-3.7810730
H	4.2905820	-8.4037820	-4.0551660
H	5.1272360	-8.3145240	-2.5034440
H	4.0260530	-5.1908550	-4.1493780
H	2.2227720	-3.7198630	-3.3195560
C	-0.7006630	0.3120100	-0.9354700
C	-1.4296820	0.2436340	-2.2451860
C	-0.8287180	0.6576360	-3.4240500
C	-1.5893670	0.6936090	-4.6441230
C	-1.0742020	1.2566970	-5.8560890
C	-1.8355740	1.2809730	-6.9972690
C	-3.1556500	0.7434390	-6.9964310
C	-3.6812170	0.2058960	-5.8489790
C	-2.9116650	0.1764710	-4.6421900
C	-3.4527020	-0.3717240	-3.4360030
C	-2.7342570	-0.3333910	-2.2710230
H	-3.1146290	-0.7995130	-1.3507350
H	-4.4408400	-0.8293020	-3.4686190
H	-4.6892610	-0.2070910	-5.8345530
H	-3.7339460	0.7701290	-7.9192090
H	-1.4449650	1.7099140	-7.9192100
H	-0.0640110	1.6698360	-5.8573320
C	0.5882160	1.0843990	-3.3903200
C	0.9585560	2.0939860	-2.5144470
C	-0.0610730	2.7266810	-1.6162700
H	0.3126310	3.6346540	-1.1221980
H	-0.9648750	2.9883820	-2.1901820
C	2.2940050	2.5898230	-2.5179650
C	3.2378630	2.0472850	-3.3498620
C	2.9070500	0.9498970	-4.2058380
C	3.8959120	0.3451420	-5.0452300
C	3.5750320	-0.7351050	-5.8279970

C	2.2569900	-1.2762930	-5.7998130
C	1.2884950	-0.7081100	-5.0113030
C	1.5796250	0.4448170	-4.2120780
H	0.2893320	-1.1501010	-4.9488900
H	2.0363780	-2.1543610	-6.4048660
H	4.3208030	-1.2019060	-6.4700780
H	4.9058400	0.7526000	-5.0389580
H	4.2580460	2.4317920	-3.3617310
H	2.5504160	3.4165880	-1.8598030
H	-1.2364460	-0.1927940	-0.1218630
H	0.3109640	-0.1115170	-1.0093580
C	0.5675050	1.7341130	0.6297250
C	0.5468380	2.9511300	1.5095300
C	-0.5161270	3.0950580	2.3919270
C	-0.6251440	4.2467590	3.2459880
C	-1.7752720	4.5104950	4.0595620
C	-1.8225630	5.6164750	4.8694930
C	-0.7256770	6.5266550	4.9175040
C	0.3824320	6.3116100	4.1393380
C	0.4493610	5.1708370	3.2751950
C	1.5773460	4.9519240	2.4331090
C	1.6283630	3.8916950	1.5485680
C	2.8415480	3.8127060	0.6996200
C	3.6830470	2.6883900	0.6899640
C	4.8859550	2.7315250	-0.0300540
C	5.2423780	3.8696170	-0.7638940
C	4.3871950	4.9804800	-0.7703210
C	3.1970900	4.9581410	-0.0353430
H	2.5333390	5.8267790	-0.0232950
C	4.7838580	6.1900590	-1.5708720
F	5.9167630	6.7787160	-1.1527830
F	5.0125600	5.9349250	-2.8713050
F	3.8963830	7.1981170	-1.6021350
H	6.1788610	3.9004420	-1.3298760
C	5.8023450	1.5330160	-0.0230700
F	5.4485280	0.5667520	-0.8807690
F	5.9244210	0.9198840	1.1616170
F	7.0805490	1.8002160	-0.3609650
H	3.3900550	1.7614690	1.2329550
H	2.4153830	5.6493900	2.5085340
H	1.2267790	7.0000200	4.1623720
H	-0.7886090	7.3904860	5.5790630
H	-2.6967510	5.8172890	5.4887320
H	-2.6182330	3.8179060	4.0259870
H	0.3755340	0.8235210	1.2177470
H	1.5103460	1.5870950	0.0933090
H	-0.1075980	2.0688870	4.7486000
H	-0.6509680	0.8135470	6.8247820
H	-2.6791680	-0.5948170	6.9482670
H	-4.1978680	-0.7879900	4.9807680
H	-4.8162100	-0.0966710	2.7033220
C	-4.5900920	1.0893130	0.3389670
C	-5.0467320	2.3090140	-0.1810900
C	-6.0611050	2.3257660	-1.1495820
C	-6.6532070	1.1338010	-1.5797360
C	-6.2074430	-0.0832070	-1.0434740

C	-5.1787680	-0.1115320	-0.0942630
H	-4.8100330	-1.0687070	0.3184570
C	-6.8632830	-1.3764600	-1.4510690
F	-6.0221440	-2.3920300	-1.6997960
F	-7.6224810	-1.3246730	-2.5590470
F	-7.6976050	-1.8666030	-0.5172720
H	-7.4587960	1.1511600	-2.3196840
C	-6.5053500	3.6563430	-1.6948560
F	-5.4984630	4.4147230	-2.1661710
F	-7.1024530	4.4460090	-0.7859340
F	-7.3777540	3.6247260	-2.7133500
H	-4.6202030	3.2462670	0.1872750
C	1.5182420	-1.4092680	6.6253660
C	1.4236440	-2.6782440	7.2013050
C	1.0733340	-3.7789320	6.4163550
C	0.8176690	-3.6140490	5.0548300
C	0.9160800	-2.3403830	4.4911720
C	1.2671870	-1.2341250	5.2645200
H	1.3511010	-0.2597180	4.7996500
S	0.5714900	-2.1467310	2.7252280
O	0.0521110	-0.7677350	2.5506090
O	-0.3323230	-3.2653650	2.3901640
N	1.9782530	-2.4211400	1.9275590
F	1.3022640	-3.0241250	0.1059020
S	3.1307180	-1.2412930	1.8811930
O	3.4788130	-0.7256690	3.2258150
O	2.8643810	-0.1771520	0.8744700
C	4.5142040	-2.2140210	1.2852830
C	5.7100390	-2.1619440	2.0007930
C	6.8214590	-2.8525940	1.5138390
C	6.7267750	-3.5839520	0.3289040
C	5.5177380	-3.6324530	-0.3715130
C	4.4010300	-2.9435450	0.0991330
H	3.4441040	-2.9902090	-0.4080150
H	5.4391630	-4.2109120	-1.2880630
H	7.5943910	-4.1204400	-0.0476220
H	7.7596770	-2.8180550	2.0615960
H	5.7551810	-1.5927790	2.9228640
H	0.5345060	-4.4541990	4.4302060
H	0.9985140	-4.7669820	6.8632220
H	1.6254470	-2.8099350	8.2613390
H	1.7987200	-0.5546040	7.2347320

159

S_MajorCl_1_MaruokaCF3_TS_Diffuse.log

C	2.8090900	-0.4857900	2.5640400
C	3.0625430	0.5217560	3.4726890
C	2.0473330	1.0829210	4.2801090
C	2.3148630	2.1633750	5.1623450
C	1.3140370	2.7000550	5.9374010
C	0.0009840	2.1775350	5.8579140
C	-0.2846410	1.1189410	5.0254210
C	0.7301990	0.5305130	4.2216490
C	0.4865760	-0.5634660	3.3273030
C	1.4721340	-0.9907640	2.4435850
C	1.0939650	-2.0231160	1.4106830
H	1.9527480	-2.3566770	0.8294150

H	0.6429380	-2.8953630	1.8907190
N	0.0621070	-1.5749720	0.3516100
O	2.5988680	1.7424970	0.1246490
C	1.8602440	2.6742880	0.4217350
C	1.6371950	3.9819750	-0.1462340
C	0.8989740	4.7560020	0.9267490
H	1.5833360	5.3421590	1.5586330
H	0.1177960	5.4176710	0.5531030
N	0.2763610	3.6872710	1.7356270
C	-1.1006430	3.4776050	1.7559570
O	-1.6425530	2.3855340	1.7898620
O	-1.6871020	4.6848950	1.8650920
C	-3.1419060	4.8769210	1.7143690
C	-3.8992760	4.1071680	2.8014010
H	-3.8083100	3.0298230	2.6637340
H	-4.9600130	4.3797680	2.7660790
H	-3.5120230	4.3691100	3.7924030
C	-3.5533870	4.4738010	0.2987930
H	-4.6281330	4.6370560	0.1661800
H	-3.0168450	5.0663740	-0.4486260
H	-3.3430000	3.4201330	0.1152600
C	-3.2962900	6.3859050	1.9206150
H	-2.7138370	6.9380450	1.1766930
H	-4.3485550	6.6703710	1.8154310
H	-2.9546610	6.6794820	2.9187970
O	1.0100760	2.4860740	1.5181640
C	2.6282850	4.6595370	-0.9975470
C	3.6164990	3.9578340	-1.7168190
C	4.5291760	4.6332500	-2.5199930
C	4.5046650	6.0290780	-2.6519440
C	3.5164770	6.7250750	-1.9480580
C	2.5982740	6.0588830	-1.1387810
H	1.8360870	6.6392150	-0.6260910
H	3.4578890	7.8081720	-2.0366330
C	5.5190690	6.7519880	-3.5068990
H	6.4635440	6.9099040	-2.9674870
H	5.1532140	7.7372830	-3.8156410
H	5.7589140	6.1835280	-4.4129300
H	5.2794560	4.0603000	-3.0621660
H	3.6619440	2.8784240	-1.6373100
C	0.7126290	-0.6922620	-0.7437030
C	1.5933250	-1.4547480	-1.7002980
C	1.0542060	-2.3400230	-2.6344740
C	1.9394780	-3.1199810	-3.4592910
C	1.4858680	-4.1816270	-4.2930430
C	2.3659270	-4.9116110	-5.0598910
C	3.7505970	-4.6194840	-5.0436110
C	4.2275670	-3.6145200	-4.2355220
C	3.3489380	-2.8556500	-3.4155740
C	3.8377870	-1.8467120	-2.5479780
C	2.9865040	-1.1877720	-1.6955940
H	3.3616310	-0.4130240	-1.0381670
H	4.8970300	-1.6073630	-2.5515390
H	5.2908400	-3.3902040	-4.2002400
H	4.4330040	-5.1975530	-5.6605650
H	1.9942670	-5.7221690	-5.6807960

H	0.4296920	-4.4240960	-4.3132860
C	-0.4255110	-2.4757460	-2.7330880
C	-1.1586480	-2.7569300	-1.5831410
C	-0.4661000	-2.8888790	-0.2557280
H	-1.1277540	-3.3156180	0.5012810
H	0.4149050	-3.5283250	-0.3453590
C	-2.5507210	-3.0020520	-1.6616640
C	-3.2196990	-2.9174200	-2.8559890
C	-2.5351830	-2.5188540	-4.0311970
C	-3.2262460	-2.3424840	-5.2601630
C	-2.5674960	-1.9027790	-6.3845350
C	-1.1834130	-1.6134030	-6.3210940
C	-0.4810550	-1.7974090	-5.1511840
C	-1.1230670	-2.2749710	-3.9744140
H	0.5744850	-1.5559600	-5.1176740
H	-0.6713130	-1.2365890	-7.2020700
H	-3.1074020	-1.7635300	-7.3171560
H	-4.2936160	-2.5479290	-5.2887230
H	-4.2831240	-3.1336180	-2.9026490
H	-3.0874410	-3.2860690	-0.7639930
H	1.2838490	0.0670380	-0.2079010
H	-0.1192020	-0.2044570	-1.2614100
C	-1.0801770	-0.7130430	0.9417850
C	-1.6507060	-1.2616940	2.2302780
C	-0.8311290	-1.2660220	3.3571390
C	-1.2227710	-1.9661770	4.5500100
C	-0.3613840	-2.1432160	5.6692440
C	-0.7754980	-2.8498210	6.7763740
C	-2.0739390	-3.4104640	6.8322970
C	-2.9292960	-3.2652020	5.7655240
C	-2.5235520	-2.5567060	4.6024400
C	-3.3908990	-2.3908340	3.4988860
C	-2.9949090	-1.7613160	2.3328830
C	-4.0556090	-1.5358980	1.3079710
C	-4.3439890	-0.2508960	0.8242830
C	-5.4465830	-0.0452040	-0.0111870
C	-6.2566920	-1.1080630	-0.4036330
C	-5.9644370	-2.3898910	0.0619120
C	-4.8871120	-2.5987490	0.9202990
H	-4.6863540	-3.5984550	1.2922420
C	-6.7721980	-3.5658360	-0.4175120
F	-6.8572290	-4.5339190	0.5196920
F	-8.0267520	-3.2133890	-0.7612890
F	-6.2067490	-4.1346890	-1.5143900
H	-7.1099360	-0.9388640	-1.0484020
C	-5.7774960	1.3571340	-0.4551280
F	-6.9368510	1.4143360	-1.1422980
F	-4.8134670	1.8693110	-1.2544970
F	-5.8920680	2.1891100	0.6043040
H	-3.7321440	0.5940560	1.1302240
H	-4.4150260	-2.7422280	3.5917940
H	-3.9269950	-3.6962660	5.7917780
H	-2.3882690	-3.9590190	7.7156660
H	-0.0978580	-2.9798730	7.6154330
H	0.6377120	-1.7248600	5.6425750
H	-0.6626760	0.2852920	1.0935230

H	-1.8123860	-0.6430370	0.1383970
H	-1.2996120	0.7452030	4.9589640
H	-0.7910740	2.6230650	6.4531420
H	1.5237820	3.5340270	6.6012250
H	3.3232650	2.5674260	5.2004720
H	4.0780010	0.8922500	3.5838700
C	3.9790350	-1.0776580	1.8542220
C	4.2841020	-2.4394490	1.9896690
C	5.4332620	-2.9769510	1.4070950
C	6.3038010	-2.1636720	0.6838020
C	6.0085430	-0.8066080	0.5505020
C	4.8605320	-0.2629080	1.1283680
H	4.6170980	0.7850860	0.9888000
C	6.8899570	0.0722300	-0.2980090
F	6.3978620	0.1818770	-1.5596470
F	8.1418350	-0.4221080	-0.4075410
F	6.9820240	1.3211680	0.1960160
H	7.2022460	-2.5771290	0.2419130
C	5.7023990	-4.4533210	1.5364140
F	5.3499560	-4.9179930	2.7567910
F	7.0043650	-4.7500080	1.3463780
F	4.9940290	-5.1659690	0.6297210
H	3.6420900	-3.0804920	2.5862140
C	-3.5018300	3.1189130	-4.1124440
C	-3.2267010	1.6237880	-3.9186590
H	-3.4515860	3.4604760	-5.1506030
H	-4.4755690	3.4240790	-3.7143250
H	-4.0887990	1.0458550	-3.5780370
H	-2.8414010	1.1347020	-4.8203810
N	-1.6738970	2.8491850	-2.6234890
Cl	-0.1017880	3.3117890	-1.4720190
C	-2.1269960	1.5782580	-2.8475430
O	-1.7348190	0.5475940	-2.3070190
C	-2.4063110	3.8209120	-3.2895110
O	-2.2395060	5.0230950	-3.2161620

147

S_MajorCl_1_MaruokaF3_TS_Diffuse.log

C	2.4591850	-2.1259560	-2.1833090
C	2.3979180	-3.4916220	-1.9893540
C	1.1937330	-4.2198720	-2.1084520
C	1.1467480	-5.6144450	-1.8441270
C	-0.0325320	-6.3108660	-1.9620840
C	-1.2161520	-5.6348970	-2.3431080
C	-1.1992910	-4.2851950	-2.6148340
C	0.0074810	-3.5375760	-2.5219840
C	0.0847120	-2.1272360	-2.7798120
C	1.2579830	-1.4224630	-2.5262000
C	1.2192900	0.0791320	-2.6641960
H	2.2046710	0.5307870	-2.5606720
H	0.8126530	0.3572960	-3.6400070
N	0.3413100	0.8250650	-1.6382740
O	2.1896520	-1.6256640	1.1312760
C	1.2643750	-2.2721150	1.6316980
C	0.9154800	-2.6153130	2.9900910
C	-0.0744180	-3.7509460	2.8597070
H	0.4190480	-4.7339320	2.8986030

H	-0.8863270	-3.7395550	3.5892520
N	-0.6449390	-3.5413540	1.5161230
C	-1.9539150	-3.1055430	1.3242260
O	-2.3160740	-2.3507560	0.4382400
O	-2.7311250	-3.7815590	2.1920250
C	-4.1832500	-3.5566750	2.3146060
C	-4.8781000	-3.8994680	0.9952370
H	-4.5891220	-3.2062700	0.2047330
H	-5.9640500	-3.8498890	1.1318720
H	-4.6205510	-4.9167540	0.6821740
C	-4.4518370	-2.1235990	2.7690820
H	-5.5241500	-1.9995860	2.9571960
H	-3.9128850	-1.8995210	3.6937270
H	-4.1513730	-1.4016880	2.0099020
C	-4.5766600	-4.5524080	3.4091380
H	-4.0487310	-4.3290810	4.3409360
H	-5.6529440	-4.4921020	3.5998120
H	-4.3337090	-5.5761600	3.1081000
O	0.3014090	-2.7958690	0.7581460
C	1.8872030	-2.5941330	4.0907140
C	3.0343050	-1.7756000	4.0654030
C	3.9228920	-1.7629880	5.1336870
C	3.7184290	-2.5551240	6.2724880
C	2.5719380	-3.3554250	6.3039650
C	1.6756000	-3.3773160	5.2385490
H	0.7874430	-3.9985310	5.3144150
H	2.3717010	-3.9707800	7.1785340
C	4.7108920	-2.5570720	7.4101980
H	5.5088190	-3.2956270	7.2467430
H	4.2303270	-2.8056550	8.3633880
H	5.1962680	-1.5813960	7.5214390
H	4.7986870	-1.1189030	5.0840140
H	3.2179200	-1.1498170	3.2004270
C	1.0645510	0.9875260	-0.2777120
C	2.1783880	1.9980660	-0.2961700
C	1.9103680	3.3654490	-0.3901040
C	3.0059480	4.2898090	-0.5176510
C	2.8225620	5.6715600	-0.8080080
C	3.8981060	6.5215400	-0.9358080
C	5.2197040	6.0416860	-0.7744250
C	5.4365710	4.7095170	-0.5136520
C	4.3495480	3.8014220	-0.3956560
C	4.5663980	2.4181940	-0.1730570
C	3.5109170	1.5409710	-0.1507550
H	3.6775560	0.4837340	0.0161010
H	5.5831800	2.0580160	-0.0393720
H	6.4472820	4.3238510	-0.4050020
H	6.0590830	6.7252550	-0.8697010
H	3.7305510	7.5701030	-1.1656060
H	1.8178730	6.0553140	-0.9419390
C	0.4932790	3.8263390	-0.3986950
C	-0.3907480	3.2717620	-1.3198870
C	0.0727670	2.2030940	-2.2696550
H	-0.6605500	2.0255870	-3.0591590
H	1.0206110	2.4857530	-2.7335300
C	-1.7167560	3.7556350	-1.4253310

C	-2.1781140	4.7411120	-0.5906130
C	-1.3486360	5.2577530	0.4351730
C	-1.8365540	6.2193420	1.3607960
C	-1.0437440	6.6778700	2.3866280
C	0.2740020	6.1831970	2.5328050
C	0.7827890	5.2668830	1.6399740
C	0.0013810	4.7875380	0.5515800
H	1.7881030	4.8874090	1.7777410
H	0.8874560	6.5234520	3.3621830
H	-1.4295880	7.4073440	3.0934880
H	-2.8578880	6.5748660	1.2490300
H	-3.1929830	5.1169660	-0.6921560
H	-2.3683750	3.3447760	-2.1876880
H	1.4141730	-0.0167530	-0.0064570
H	0.2716570	1.2660690	0.4257400
C	-0.9744430	0.0777560	-1.3123070
C	-1.7153660	-0.4176090	-2.5327420
C	-1.1189920	-1.4119950	-3.3030630
C	-1.6549510	-1.7588580	-4.5877100
C	-0.9964290	-2.6378880	-5.4911840
C	-1.5358460	-2.9163400	-6.7268460
C	-2.7676220	-2.3421650	-7.1223320
C	-3.4288530	-1.4845590	-6.2745750
C	-2.8879060	-1.1624470	-5.0007060
C	-3.5589010	-0.2920370	-4.1100720
C	-3.0131300	0.0880100	-2.8990860
C	-3.8424540	0.9185210	-1.9807980
C	-3.9870330	0.5650490	-0.6286760
C	-4.7795960	1.3470750	0.1970800
C	-5.4517840	2.4691300	-0.2776350
C	-5.3330120	2.7911330	-1.6266110
C	-4.5432190	2.0318960	-2.4774700
H	-4.4571980	2.3373580	-3.5148220
H	-3.5032500	-0.3128300	-0.2107710
H	-4.5510210	0.0593380	-4.3799660
H	-4.3730080	-1.0331190	-6.5688680
H	-3.1845950	-2.5772930	-8.0974560
H	-1.0110620	-3.5827340	-7.4055400
H	-0.0509110	-3.0829100	-5.2034900
H	-0.6999410	-0.7579180	-0.6612850
H	-1.5498370	0.7833820	-0.7160160
H	-2.1199440	-3.7798990	-2.8806930
H	-2.1503870	-6.1852100	-2.4102600
H	-0.0629750	-7.3758860	-1.7501830
H	2.0595110	-6.1166100	-1.5333410
H	3.3089850	-4.0322570	-1.7485390
C	3.8083970	-1.4948750	-2.1233080
C	4.3269980	-0.8022680	-3.2295260
C	5.6234100	-0.3060450	-3.1899600
C	6.4326250	-0.4906710	-2.0720780
C	5.9130790	-1.1864250	-0.9821170
C	4.6179470	-1.6854450	-0.9916420
H	4.2234410	-2.1677540	-0.1041420
H	3.7532370	-0.6757580	-4.1415540
C	-3.1928600	2.2323610	4.6639140
C	-2.9289750	2.7231780	3.2426310

H	-2.8200160	2.9108540	5.4365820
H	-4.2466690	2.0484340	4.8824770
H	-3.8312280	2.7657540	2.6309160
H	-2.4496800	3.7035910	3.1887280
N	-1.7070590	0.7267500	3.5910100
Cl	-0.4894300	-0.8249500	3.3120070
C	-1.9744040	1.6750340	2.6459890
O	-1.5395300	1.7130960	1.5010050
C	-2.4220700	0.9046950	4.7617470
O	-2.4399730	0.1485950	5.7094680
F	6.6946420	-1.3422870	0.0973580
F	7.6793130	-0.0026140	-2.0412680
F	6.1296560	0.3519580	-4.2455050
F	-4.9171180	1.0409210	1.4989350
F	-6.1882830	3.2227350	0.5471530
F	-5.9749960	3.8793450	-2.0855150

159

S_MajorCl_2_MaruokaCF3_TS_Diffuse.log

C	2.7969290	0.5693500	2.5469210
C	3.0345070	1.8452580	3.0152850
C	2.0113340	2.6473720	3.5692440
C	2.2675950	3.9768070	3.9997160
C	1.2632120	4.7452500	4.5387200
C	-0.0431420	4.2142460	4.6615410
C	-0.3183770	2.9249990	4.2647250
C	0.7015630	2.0951680	3.7224080
C	0.4712890	0.7465780	3.2891640
C	1.4686360	0.0355980	2.6287440
C	1.1169620	-1.3176770	2.0638150
H	1.9870550	-1.8361640	1.6626980
H	0.6702770	-1.9445920	2.8396410
N	0.0912330	-1.3287980	0.9086550
O	2.5254600	1.7176230	-0.5663780
C	1.7233950	2.6402370	-0.6512630
C	1.4491890	3.6268440	-1.6651980
C	0.5793890	4.6750960	-1.0021830
H	1.1607620	5.5513190	-0.6765440
H	-0.2532790	5.0190750	-1.6177620
N	0.0529050	3.9627170	0.1765710
C	-1.2763180	3.8395270	0.5146210
O	-1.7536260	2.9184380	1.1616310
O	-1.9140150	4.9534160	0.0944660
C	-3.2364110	5.3427980	0.6118310
C	-3.1550610	5.5263620	2.1303850
H	-2.9286220	4.5795910	2.6253910
H	-4.1137380	5.8987130	2.5086450
H	-2.3787340	6.2552770	2.3873770
C	-4.2988380	4.3202940	0.2070700
H	-5.2954200	4.7211320	0.4247770
H	-4.2410550	4.1118090	-0.8643190
H	-4.1729690	3.3850590	0.7528640
C	-3.4795660	6.6814810	-0.0900730
H	-3.4980420	6.5482210	-1.1763760
H	-4.4407820	7.1013960	0.2245260
H	-2.6889400	7.3971140	0.1571450
O	0.8233860	2.7941420	0.4131640

C	2.4416380	4.0287080	-2.6756010
C	3.5297550	3.2089400	-3.0367970
C	4.4448320	3.6213960	-3.9990340
C	4.3214590	4.8554180	-4.6540280
C	3.2389080	5.6666060	-4.3000840
C	2.3171800	5.2645750	-3.3350220
H	1.4825920	5.9211850	-3.1048600
H	3.1088680	6.6299620	-4.7893020
C	5.3052010	5.2749430	-5.7211670
H	6.3355600	5.0259020	-5.4403960
H	5.2588410	6.3534810	-5.9069110
H	5.1020440	4.7708180	-6.6759580
H	5.2775220	2.9663750	-4.2495720
H	3.6541890	2.2499150	-2.5485950
C	0.7402020	-0.9608820	-0.4497610
C	1.6261980	-2.0389760	-1.0178030
C	1.0947130	-3.2308730	-1.5120320
C	1.9882870	-4.2724780	-1.9473820
C	1.5450690	-5.5857480	-2.2742380
C	2.4326690	-6.5581140	-2.6768620
C	3.8148970	-6.2735630	-2.7831860
C	4.2821320	-5.0224020	-2.4565360
C	3.3957260	-4.0021290	-2.0166720
C	3.8752330	-2.7234760	-1.6360820
C	3.0171730	-1.7822860	-1.1232650
H	3.3861440	-0.8049160	-0.8364490
H	4.9323330	-2.4971010	-1.7390600
H	5.3435320	-4.7947150	-2.5168540
H	4.5032090	-7.0476420	-3.1109910
H	2.0688690	-7.5546800	-2.9116580
H	0.4910320	-5.8243440	-2.1919180
C	-0.3843270	-3.4042650	-1.5507390
C	-1.1238240	-3.1953060	-0.3893600
C	-0.4384820	-2.7747520	0.8800900
H	-1.1049630	-2.8583380	1.7411460
H	0.4412350	-3.3964190	1.0615690
C	-2.5138250	-3.4611540	-0.3644030
C	-3.1754630	-3.8821740	-1.4894370
C	-2.4852790	-3.9996750	-2.7215650
C	-3.1688560	-4.3567220	-3.9153240
C	-2.5059840	-4.4159620	-5.1190730
C	-1.1254150	-4.1089950	-5.1778430
C	-0.4299820	-3.7826710	-4.0350660
C	-1.0755150	-3.7383770	-2.7671630
H	0.6230290	-3.5370700	-4.1022170
H	-0.6106590	-4.1245720	-6.1344220
H	-3.0403540	-4.6825200	-6.0267990
H	-4.2340610	-4.5672880	-3.8590270
H	-4.2378180	-4.1036190	-1.4478160
H	-3.0549650	-3.3504130	0.5682650
H	1.3051010	-0.0453280	-0.2679520
H	-0.0983710	-0.7281330	-1.1135620
C	-1.0433470	-0.2957600	1.0985040
C	-1.6502140	-0.3038480	2.4818160
C	-0.8525960	0.1039180	3.5494200
C	-1.2872560	-0.0913790	4.9051290

C	-0.4503780	0.1395960	6.0329240
C	-0.9064640	-0.0901080	7.3118580
C	-2.2254970	-0.5520980	7.5364370
C	-3.0585970	-0.7921590	6.4691300
C	-2.6096810	-0.5830110	5.1369740
C	-3.4530610	-0.8195100	4.0275880
C	-3.0134480	-0.6931920	2.7221070
C	-4.0327040	-0.8631630	1.6462190
C	-4.2020740	0.0945360	0.6320540
C	-5.2349800	-0.0419460	-0.2974310
C	-6.1078620	-1.1282660	-0.2460530
C	-5.9441630	-2.0812040	0.7570990
C	-4.9239180	-1.9456930	1.6978210
H	-4.8128600	-2.6953560	2.4747570
C	-6.8162890	-3.3071820	0.7809030
F	-7.0343550	-3.7450230	2.0395560
F	-8.0159100	-3.0888630	0.2068860
F	-6.2380290	-4.3369730	0.1073880
H	-6.9027420	-1.2288500	-0.9752610
C	-5.4744650	1.0357480	-1.3242920
F	-6.0658440	0.5384200	-2.4380130
F	-4.3362790	1.6436630	-1.7053010
F	-6.2986870	1.9965490	-0.8428460
H	-3.5432910	0.9566230	0.5810500
H	-4.4911090	-1.0789170	4.2172690
H	-4.0716030	-1.1539690	6.6268990
H	-2.5732960	-0.7214600	8.5515500
H	-0.2469460	0.0835970	8.1574610
H	0.5635240	0.4897820	5.8786850
H	-0.6033780	0.6784160	0.8749790
H	-1.7539730	-0.5262700	0.3071700
H	-1.3291830	2.5438060	4.3407000
H	-0.8391810	4.8342380	5.0642470
H	1.4649910	5.7636180	4.8589510
H	3.2713260	4.3774360	3.8829530
H	4.0437220	2.2460220	2.9745300
C	3.9776720	-0.2240310	2.0993130
C	4.3232230	-1.4183880	2.7469940
C	5.4848510	-2.1104130	2.3994630
C	6.3258420	-1.6201120	1.4021990
C	5.9884680	-0.4301930	0.7562960
C	4.8287480	0.2668100	1.0981240
H	4.5531150	1.1725000	0.5681810
C	6.8357930	0.0645240	-0.3866170
F	6.3518550	-0.3785380	-1.5765820
F	8.1106180	-0.3719510	-0.2961020
F	6.8638080	1.4086660	-0.4508910
H	7.2339890	-2.1495860	1.1403940
C	5.8000710	-3.4129810	3.0870940
F	5.4640560	-3.3782740	4.3966700
F	7.1105130	-3.7229740	3.0092970
F	5.1133420	-4.4410040	2.5363680
H	3.7031400	-1.7935400	3.5555550
C	-3.1024960	0.7470840	-5.3944990
C	-3.1647500	-0.2932440	-4.2703350
H	-2.8061420	0.3335480	-6.3638910

H	-4.0443340	1.2840290	-5.5472920
H	-4.1458700	-0.3628640	-3.7931720
H	-2.8852630	-1.3028980	-4.5877960
N	-1.5418830	1.3296210	-3.7050100
Cl	-0.1200850	2.3438190	-2.7251140
C	-2.1398490	0.1961820	-3.2356140
O	-1.9145620	-0.3702700	-2.1671250
C	-2.0306800	1.7519100	-4.9361910
O	-1.6825410	2.7407630	-5.5472780

147
S_MajorCl_2_MaruokaF3_TS_Diffuse.log

C	2.5108910	-2.1238240	-2.1584910
C	2.4474460	-3.4928190	-1.9926050
C	1.2436040	-4.2179230	-2.1377420
C	1.1908330	-5.6177110	-1.9014280
C	0.0088100	-6.3071560	-2.0329430
C	-1.1721170	-5.6198310	-2.4022380
C	-1.1480230	-4.2669470	-2.6555580
C	0.0617670	-3.5264760	-2.5479240
C	0.1446640	-2.1145770	-2.7885200
C	1.3141480	-1.4138550	-2.5079890
C	1.2765830	0.0893000	-2.6311540
H	2.2604830	0.5394140	-2.5068830
H	0.8874800	0.3761750	-3.6113150
N	0.3756730	0.8290600	-1.6170120
O	2.1649850	-1.6563650	1.1302500
C	1.2388310	-2.2851920	1.6272320
C	0.8774750	-2.6080430	2.9877610
C	-0.1286470	-3.7339010	2.8690330
H	0.3478040	-4.7239160	2.9335150
H	-0.9444410	-3.6915740	3.5900390
N	-0.6774330	-3.5423270	1.5104550
C	-1.9819360	-3.1015900	1.2941190
O	-2.3237270	-2.3420800	0.4034650
O	-2.7780050	-3.7665340	2.1513050
C	-4.2261150	-3.5129780	2.2592540
C	-4.9168930	-3.8026690	0.9226460
H	-4.6096620	-3.0924820	0.1542490
H	-6.0030220	-3.7372250	1.0534520
H	-4.6760210	-4.8156890	0.5814420
C	-4.4593960	-2.0853760	2.7553980
H	-5.5305770	-1.9292380	2.9281090
H	-3.9293870	-1.9108530	3.6971540
H	-4.1205380	-1.3479210	2.0278290
C	-4.6561820	-4.5310940	3.3190520
H	-4.1304190	-4.3509000	4.2617680
H	-5.7325520	-4.4471740	3.5023290
H	-4.4381680	-5.5517880	2.9878840
O	0.2803790	-2.8106740	0.7503870
C	1.8508500	-2.5952840	4.0910260
C	3.0226660	-1.8124760	4.0557890
C	3.9174370	-1.8187010	5.1199860
C	3.6915430	-2.5902460	6.2694180
C	2.5267800	-3.3631550	6.3072590
C	1.6239370	-3.3661450	5.2453020
H	0.7254410	-3.9718490	5.3252500

H	2.3173190	-3.9734810	7.1836030
C	4.6570640	-2.5620800	7.4311190
H	5.6990430	-2.5639840	7.0895410
H	4.5175360	-3.4267500	8.0889350
H	4.5224990	-1.6607750	8.0449480
H	4.8159060	-1.2068840	5.0577810
H	3.2257480	-1.2074040	3.1804000
C	1.0705630	0.9954300	-0.2425220
C	2.1833220	2.0119610	-0.2503840
C	1.9126260	3.3774010	-0.3411840
C	3.0052330	4.3081250	-0.4496510
C	2.8185490	5.6917750	-0.7303270
C	3.8910520	6.5480660	-0.8393540
C	5.2134350	6.0731180	-0.6701460
C	5.4339240	4.7394290	-0.4188790
C	4.3501240	3.8253090	-0.3187230
C	4.5711020	2.4410410	-0.1052720
C	3.5184180	1.5592730	-0.0965750
H	3.6892110	0.5012430	0.0626800
H	5.5885980	2.0841470	0.0329320
H	6.4454530	4.3576100	-0.3044390
H	6.0502450	6.7613210	-0.7509510
H	3.7207620	7.5979570	-1.0614860
H	1.8134690	6.0721110	-0.8704450
C	0.4934200	3.8289480	-0.3698110
C	-0.3706740	3.2736150	-1.3103050
C	0.1149400	2.2075650	-2.2534440
H	-0.6020270	2.0277340	-3.0572270
H	1.0701430	2.4946460	-2.6987750
C	-1.6957040	3.7535180	-1.4390730
C	-2.1770980	4.7331030	-0.6084670
C	-1.3701400	5.2465090	0.4372730
C	-1.8793350	6.1994760	1.3602730
C	-1.1083050	6.6532460	2.4050390
C	0.2091770	6.1637670	2.5723220
C	0.7388550	5.2571500	1.6819430
C	-0.0202690	4.7819430	0.5758280
H	1.7435840	4.8816280	1.8350260
H	0.8054750	6.5007890	3.4155930
H	-1.5107930	7.3755750	3.1099020
H	-2.9002250	6.5511640	1.2323930
H	-3.1915290	5.1053810	-0.7278110
H	-2.3301670	3.3439390	-2.2164080
H	1.4384710	0.0035120	0.0261580
H	0.2713670	1.2691120	0.4531890
C	-0.9391110	0.0755560	-1.3146940
C	-1.6617170	-0.4066200	-2.5525050
C	-1.0497000	-1.3925430	-3.3228220
C	-1.5610250	-1.7290820	-4.6225620
C	-0.8865890	-2.6019520	-5.5215170
C	-1.4026970	-2.8687440	-6.7701790
C	-2.6250900	-2.2879560	-7.1849770
C	-3.3007180	-1.4362540	-6.3426590
C	-2.7843030	-1.1273560	-5.0551600
C	-3.4722280	-0.2668320	-4.1685610
C	-2.9523580	0.0989000	-2.9408600

C	-3.8098140	0.9083280	-2.0317140
C	-3.9763940	0.5307940	-0.6890020
C	-4.7950410	1.2887440	0.1336960
C	-5.4702480	2.4117780	-0.3349960
C	-5.3276030	2.7580700	-1.6756830
C	-4.5139540	2.0209660	-2.5241610
H	-4.4120470	2.3437040	-3.5548690
H	-3.4896880	-0.3489540	-0.2782030
H	-4.4596150	0.0861500	-4.4539610
H	-4.2382110	-0.9801590	-6.6511270
H	-3.0235130	-2.5134800	-8.1701740
H	-0.8663770	-3.5307660	-7.4442090
H	0.0520650	-3.0520250	-5.2194870
H	-0.6669150	-0.7620470	-0.6691330
H	-1.5185370	0.7748780	-0.7162360
H	-2.0659710	-3.7531240	-2.9143230
H	-2.1091910	-6.1643580	-2.4771720
H	-0.0261120	-7.3756790	-1.8397850
H	2.1010910	-6.1292620	-1.5990760
H	3.3553420	-4.0381960	-1.7503640
C	3.8565380	-1.4887360	-2.0635450
C	4.3864890	-0.7653850	-3.1449430
C	5.6756750	-0.2551860	-3.0724600
C	6.4691140	-0.4586760	-1.9468860
C	5.9409800	-1.1883060	-0.8834270
C	4.6513660	-1.7007520	-0.9252420
H	4.2483100	-2.2087680	-0.0561740
H	3.8250530	-0.6209400	-4.0620660
C	-3.4176870	2.0594870	4.6194480
C	-3.0046780	2.7145580	3.2962880
H	-3.1780890	2.6573780	5.5047770
H	-4.4840500	1.8175080	4.6718710
H	-3.8398940	2.8777820	2.6103400
H	-2.4919990	3.6743800	3.4199220
N	-1.8160040	0.6824150	3.5370540
Cl	-0.5176470	-0.8104610	3.2419310
C	-2.0196600	1.7161630	2.6664760
O	-1.5132210	1.8546030	1.5548810
C	-2.6112940	0.7498660	4.6715790
O	-2.6798830	-0.0869970	5.5511060
F	6.7088050	-1.3655770	0.2026470
F	7.7091390	0.0435650	-1.8840480
F	6.1907650	0.4350710	-4.1030250
F	-4.9545570	0.9567570	1.4266090
F	-6.2281260	3.1464950	0.4889520
F	-5.9707950	3.8485210	-2.1278180

159

S_MajorCl_3_MaruokaCF3_TS_Diffuse.log

N	-0.7908290	-1.5800150	0.3828290
C	-0.5674050	-2.5388820	1.5665730
C	-1.9605760	-2.1810120	-0.4194110
C	0.2756200	-1.9745720	2.6773800
H	-0.1125050	-3.4331250	1.1347350
H	-1.5694750	-2.7927690	1.9198310
C	-1.1303160	-0.1569980	0.8945760
C	0.5010090	-1.4031070	-0.4573800

C	-2.1282130	-1.6241650	-1.8127610
H	-2.8379850	-2.0108730	0.2033110
H	-1.7631550	-3.2553660	-0.4528850
C	1.5669450	-2.5103150	2.9022750
C	-0.2216780	-0.9666800	3.5020060
C	-2.0666460	-0.1230940	2.0711340
H	-1.5296290	0.3817830	0.0359710
H	-0.1631320	0.2898720	1.1323680
C	0.8335270	-2.5747470	-1.3480210
H	1.2668430	-1.1566000	0.2742200
H	0.3386680	-0.4938740	-1.0367560
C	-3.2738240	-0.8508180	-2.2009290
C	-1.1217540	-1.8847910	-2.7379400
C	2.3856670	-2.0010430	3.8788060
H	1.9056530	-3.3417410	2.2952360
C	0.6591250	-0.3418450	4.4515690
C	-1.6346990	-0.5255730	3.3340440
C	-3.3618760	0.4272540	1.9017440
C	2.0211180	-3.3655410	-1.1786860
C	-0.0330600	-2.8511800	-2.4029600
C	-3.2833450	-0.2522560	-3.4455760
C	-4.5140490	-0.7405510	-1.3831890
C	-1.1028170	-1.2162190	-4.0078360
C	1.9776740	-0.8763780	4.6394810
H	3.3663740	-2.4364280	4.0485810
C	0.3051260	0.8338950	5.1711450
C	-2.5679900	-0.5370160	4.4286670
C	-4.2406620	0.5070640	2.9535260
H	-3.6277800	0.8336620	0.9337070
C	2.1851710	-4.4930500	-1.9638680
C	3.1588990	-3.0062450	-0.2812680
C	0.1071570	-4.0507610	-3.1782500
C	-2.2043520	-0.3715810	-4.3505330
H	-4.1557880	0.3168640	-3.7541550
C	-5.1665380	-1.8808090	-0.8940690
C	-5.1090430	0.5135320	-1.1718870
C	-0.0164880	-1.3113050	-4.9213850
C	2.8600360	-0.2503820	5.5600390
C	1.1949870	1.4333410	6.0333230
H	-0.6699900	1.2791220	5.0126100
C	-2.2587090	-1.0913400	5.7029980
C	-3.8858400	-0.0051880	4.2273930
H	-5.2197240	0.9558840	2.8124020
C	1.2333330	-4.8949350	-2.9283240
H	3.0939340	-5.0799760	-1.8629120
C	3.7053240	-3.9979150	0.5480880
C	3.7897440	-1.7515860	-0.3393430
C	-0.8388740	-4.4632930	-4.1580370
C	-2.2041900	0.3117220	-5.5955450
C	-6.3720260	-1.7709550	-0.1986790
H	-4.7468980	-2.8653180	-1.0745640
C	-6.3152150	0.6157340	-0.4796490
H	-4.5954920	1.4031750	-1.5199850
C	-0.0372520	-0.6225650	-6.1128910
H	0.8490410	-1.9095310	-4.6629770
C	2.4818740	0.8829360	6.2402850

H	3.8529950	-0.6716910	5.6946060
H	0.9108650	2.3450320	6.5509880
C	-3.1841350	-1.0924970	6.7222720
H	-1.2801790	-1.5274770	5.8666730
C	-4.8107700	-0.0064990	5.3063500
C	1.3907040	-6.0942880	-3.6743620
C	4.8504870	-3.7496350	1.3029370
H	3.2298810	-4.9714050	0.6052240
C	4.9487710	-1.5209120	0.4035480
H	3.4141140	-0.9663970	-0.9874060
C	-0.6682620	-5.6391590	-4.8538420
H	-1.7105810	-3.8476490	-4.3479610
C	-1.1428660	0.1895600	-6.4607340
H	-3.0527750	0.9441120	-5.8431750
C	-7.0826680	-3.0188790	0.2573560
C	-6.9537500	-0.5230090	0.0141260
C	-6.8989960	1.9717920	-0.1815600
H	0.8088680	-0.6971100	-6.7897490
H	3.1725840	1.3667970	6.9248780
C	-4.4705670	-0.5351840	6.5291210
H	-2.9254960	-1.5281770	7.6832800
H	-5.8003850	0.4119080	5.1403400
C	0.4598230	-6.4612440	-4.6173950
H	2.2588960	-6.7188800	-3.4798480
C	5.3658840	-4.7826850	2.2661590
C	5.4864440	-2.5128220	1.2224900
C	5.6460270	-0.1821570	0.3710240
H	-1.4072070	-5.9405530	-5.5907570
H	-1.1434870	0.7254370	-7.4053100
F	-7.8778380	-3.5197530	-0.7143570
F	-6.2076900	-3.9944900	0.5911580
F	-7.8654650	-2.7862510	1.3310630
H	-7.8906000	-0.4389240	0.5524800
F	-8.2484370	1.9453250	-0.1710120
F	-6.5061290	2.4163160	1.0406720
F	-6.5139060	2.9003980	-1.0801250
H	-5.1883410	-0.5358370	7.3446860
H	0.5838060	-7.3826680	-5.1791390
F	4.9388960	-6.0265660	1.9604240
F	4.9439360	-4.5230190	3.5322770
F	6.7137820	-4.8094370	2.3050290
H	6.3948470	-2.3239800	1.7837290
F	5.3975860	0.5120230	1.5024550
F	6.9924080	-0.3480840	0.3112780
F	5.2774060	0.5644250	-0.6757740
O	0.1838550	2.0178050	-0.6930560
N	-0.2545950	3.3242280	-0.9989920
C	1.4210410	2.1430820	0.0307940
C	0.7403510	4.3299960	-0.5708330
C	-1.6079300	3.4784060	-1.0021370
C	1.8329680	3.4834540	0.0429390
O	1.8841080	1.0731430	0.4440010
H	1.0545950	4.9247920	-1.4403120
H	0.2779050	5.0207110	0.1493260
O	-1.8857190	4.8037450	-1.0871220
O	-2.4297010	2.5622170	-0.9890350

C	2.8326610	4.0873560	0.9135320
C	-3.2597490	5.3104730	-1.1758030
C	3.0028820	5.4834520	0.9706800
C	3.6669520	3.3086510	1.7506250
C	-3.0484210	6.8247550	-1.2636330
C	-4.0337930	4.9484960	0.0963500
C	-3.9359470	4.7903650	-2.4493320
C	3.9419530	6.0680880	1.8206440
H	2.4056190	6.1282660	0.3309240
C	4.5972740	3.9033860	2.5924600
H	3.5683470	2.2313680	1.7243450
H	-2.5361570	7.1954890	-0.3701620
H	-4.0137800	7.3353920	-1.3481910
H	-2.4417830	7.0787160	-2.1385990
H	-5.0423220	5.3737380	0.0523570
H	-3.5260800	5.3599540	0.9755110
H	-4.1186690	3.8682960	0.2133130
H	-3.3801260	5.1170580	-3.3350290
H	-4.9544840	5.1884740	-2.5161530
H	-3.9859010	3.7012610	-2.4485530
C	4.7578450	5.2968050	2.6528510
H	4.0422640	7.1520400	1.8311430
H	5.2172770	3.2663160	3.2230280
C	5.7728490	5.9293910	3.5764420
H	6.7839840	5.5397750	3.3986850
H	5.5413730	5.7376160	4.6331800
H	5.8105150	7.0156990	3.4416760
C	4.2916370	1.2100510	-3.9584200
C	5.0571190	1.3140790	-5.2753050
N	4.0849760	2.5475230	-3.5349650
O	3.8925920	0.2066730	-3.4018280
C	5.7006430	2.7108520	-5.2368060
H	5.7511250	0.4815170	-5.4015700
H	4.3078240	1.2519700	-6.0727920
C	4.8439540	3.5087030	-4.2515850
Cl	3.0211230	2.9896580	-1.8447570
H	5.7697860	3.2175820	-6.2002340
H	6.7106390	2.6706000	-4.8145370
O	4.8364270	4.7036310	-4.0672350

159

S_MajorCl_4_MaruokaCF3_TS_Diffuse.log

C	2.8255590	0.6295770	2.5205290
C	3.0237820	1.9078380	3.0006200
C	1.9800940	2.6671280	3.5760810
C	2.1961830	3.9986280	4.0218210
C	1.1726540	4.7252950	4.5822010
C	-0.1136000	4.1487440	4.7114060
C	-0.3497770	2.8565950	4.2992380
C	0.6914700	2.0687890	3.7352030
C	0.5027480	0.7184870	3.2870930
C	1.5178240	0.0485820	2.6109430
C	1.2076470	-1.3118640	2.0389810
H	2.0918010	-1.7989940	1.6293430
H	0.7869110	-1.9578270	2.8136380
N	0.1762850	-1.3487220	0.8898710
O	2.4615030	1.7894670	-0.6164420

C	1.6354790	2.6922840	-0.6702500
C	1.2885480	3.6620380	-1.6824950
C	0.4508710	4.7081180	-0.9740380
H	1.0476560	5.5823750	-0.6726220
H	-0.4105550	5.0537060	-1.5469400
N	-0.0192940	3.9911810	0.2264180
C	-1.3413060	3.8188810	0.5827600
O	-1.7782580	2.8731740	1.2216200
O	-2.0184430	4.9197340	0.1938590
C	-3.3500550	5.2534940	0.7264920
C	-3.6437840	6.5956260	0.0510860
H	-2.8762550	7.3332390	0.3064030
H	-4.6164380	6.9764960	0.3799150
H	-3.6656430	6.4815840	-1.0373090
C	-3.2645150	5.4114660	2.2476380
H	-4.2328610	5.7434020	2.6387030
H	-3.0021410	4.4640670	2.7232760
H	-2.5120170	6.1618850	2.5134220
C	-4.3789460	4.2025040	0.3088840
H	-4.2175530	3.2624530	0.8368460
H	-5.3873660	4.5644710	0.5396360
H	-4.3205580	4.0161400	-0.7665240
O	0.7871850	2.8404650	0.4345480
C	2.2298170	4.0743120	-2.7390660
C	3.3125280	3.2691530	-3.1454620
C	4.1756100	3.6893770	-4.1516020
C	4.0025130	4.9169440	-4.8072530
C	2.9247680	5.7135500	-4.4082370
C	2.0551830	5.3035520	-3.3990550
H	1.2215210	5.9482160	-3.1345040
H	2.7570050	6.6715450	-4.8964530
C	4.9295220	5.3450940	-5.9207780
H	4.8625170	6.4226540	-6.1057220
H	4.6867710	4.8368330	-6.8640800
H	5.9746970	5.1078350	-5.6889700
H	5.0063170	3.0458930	-4.4363800
H	3.4751890	2.3159190	-2.6572350
C	0.8083760	-0.9544040	-0.4690990
C	1.7220460	-2.0031570	-1.0482380
C	1.2214890	-3.2058120	-1.5485650
C	2.1411800	-4.2179440	-1.9985430
C	1.7325690	-5.5400710	-2.3345890
C	2.6442900	-6.4836280	-2.7515940
C	4.0173420	-6.1596060	-2.8638340
C	4.4516350	-4.8987390	-2.5290670
C	3.5399740	-3.9075140	-2.0746880
C	3.9855410	-2.6187440	-1.6869700
C	3.1044230	-1.7065050	-1.1611470
H	3.4477740	-0.7213420	-0.8695910
H	5.0349070	-2.3610810	-1.7958620
H	5.5058600	-4.6409640	-2.5941720
H	4.7248550	-6.9112370	-3.2028350
H	2.3069140	-7.4877950	-2.9933660
H	0.6860110	-5.8083350	-2.2482080
C	-0.2522540	-3.4206220	-1.5796180
C	-0.9895800	-3.2430320	-0.4118300

C	-0.3090050	-2.8105110	0.8559030
H	-0.9666850	-2.9198080	1.7208630
H	0.5910010	-3.4052490	1.0279040
C	-2.3716630	-3.5464150	-0.3811220
C	-3.0292430	-3.9716960	-1.5069120
C	-2.3443030	-4.0560200	-2.7447060
C	-3.0266050	-4.4139540	-3.9387880
C	-2.3705580	-4.4399960	-5.1473480
C	-0.9982740	-4.0989460	-5.2103690
C	-0.3035050	-3.7715170	-4.0675330
C	-0.9419040	-3.7591240	-2.7954850
H	0.7426720	-3.4991120	-4.1378680
H	-0.4895610	-4.0889540	-6.1702620
H	-2.9042230	-4.7069670	-6.0553820
H	-4.0858920	-4.6517200	-3.8787690
H	-4.0852750	-4.2210980	-1.4616860
H	-2.9092330	-3.4604740	0.5562860
H	1.3462360	-0.0233290	-0.2843520
H	-0.0390020	-0.7432220	-1.1288060
C	-0.9882090	-0.3510630	1.0918740
C	-1.5884690	-0.3890540	2.4776970
C	-0.7981810	0.0300580	3.5465910
C	-1.2192710	-0.1961070	4.9017260
C	-0.3835770	0.0459320	6.0280600
C	-0.8249090	-0.2152510	7.3061310
C	-2.1275180	-0.7214370	7.5313400
C	-2.9588970	-0.9729660	6.4653150
C	-2.5243880	-0.7318410	5.1338160
C	-3.3667280	-0.9781660	4.0257210
C	-2.9386170	-0.8206810	2.7197610
C	-3.9618850	-1.0008530	1.6487370
C	-4.1730910	-0.0266380	0.6582920
C	-5.2166120	-0.1716330	-0.2586580
C	-6.0565630	-1.2842760	-0.2198160
C	-5.8496220	-2.2547400	0.7581920
C	-4.8203610	-2.1100060	1.6875960
H	-4.6774060	-2.8722280	2.4469100
C	-6.6869600	-3.5051020	0.7689080
F	-6.0866680	-4.5072180	0.0737640
F	-6.8810160	-3.9689390	2.0222070
F	-7.8974400	-3.3108770	0.2093290
H	-6.8602220	-1.3908760	-0.9384450
C	-5.5057030	0.9237080	-1.2541510
F	-6.3388110	1.8540090	-0.7319050
F	-6.1092070	0.4396410	-2.3669420
F	-4.3890730	1.5658840	-1.6466510
H	-3.5402740	0.8553980	0.6188790
H	-4.3956670	-1.2703860	4.2173250
H	-3.9592210	-1.3682890	6.6235490
H	-2.4640100	-0.9153410	8.5458680
H	-0.1663590	-0.0323420	8.1505410
H	0.6179140	0.4299170	5.8731080
H	-0.5775510	0.6373230	0.8746820
H	-1.6952840	-0.5964410	0.3015070
H	-1.3464550	2.4409050	4.3801250
H	-0.9255790	4.7359100	5.1310980

H	1.3435460	5.7454590	4.9144320
H	3.1845060	4.4344620	3.8998730
H	4.0179330	2.3440110	2.9533540
C	4.0286450	-0.1159490	2.0507350
C	4.4269770	-1.3014620	2.6840380
C	5.6088430	-1.9469730	2.3157150
C	6.4175060	-1.4179030	1.3116780
C	6.0273960	-0.2365630	0.6797650
C	4.8471090	0.4139280	1.0422330
H	4.5307750	1.3123610	0.5226400
C	6.8400330	0.2961780	-0.4711720
F	6.3615320	-0.1647530	-1.6564490
F	8.1330200	-0.0865310	-0.3961850
F	6.8108170	1.6403580	-0.5320820
H	7.3411830	-1.9109760	1.0336670
C	5.9810600	-3.2421220	2.9888180
F	5.3186450	-4.2894460	2.4449140
F	5.6693170	-3.2260320	4.3047490
F	7.2994240	-3.5071980	2.8841370
H	3.8320730	-1.7056310	3.4975310
C	-3.2941040	-0.3471630	-4.1239660
C	-3.5747920	0.8701410	-5.0104200
H	-4.1635630	-0.7136870	-3.5733650
H	-2.8792500	-1.1963830	-4.6786080
H	-3.5330310	0.6740410	-6.0856520
H	-4.5487430	1.3240840	-4.7961420
N	-1.7780170	1.3692030	-3.5432490
Cl	-0.3143110	2.3797700	-2.6508250
C	-2.4804820	1.8829150	-4.6271520
O	-2.2873220	2.9529220	-5.1669120
C	-2.2231960	0.1446540	-3.1393950
O	-1.8402160	-0.4963990	-2.1614340

147

S_MajorCl_4_MaruokaF3_TS_Diffuse.log

C	2.5010450	-2.1539400	-2.1409480
C	2.4321480	-3.5210970	-1.9624260
C	1.2252630	-4.2426550	-2.0997910
C	1.1671110	-5.6400380	-1.8508370
C	-0.0177540	-6.3259070	-1.9752850
C	-1.1962180	-5.6372290	-2.3498910
C	-1.1669660	-4.2867580	-2.6152760
C	0.0458410	-3.5501990	-2.5151660
C	0.1342360	-2.1408900	-2.7686650
C	1.3067900	-1.4423220	-2.4957320
C	1.2748940	0.0598100	-2.6325990
H	2.2606360	0.5072420	-2.5132790
H	0.8859690	0.3393470	-3.6149440
N	0.3778150	0.8120700	-1.6242760
O	2.1584860	-1.6588830	1.1460860
C	1.2287330	-2.2762990	1.6490800
C	0.8585560	-2.5705120	3.0161770
C	-0.1433270	-3.7026030	2.9099220
H	0.3370160	-4.6894490	2.9889250
H	-0.9611490	-3.6528810	3.6280220
N	-0.6891230	-3.5321570	1.5473360
C	-1.9914920	-3.0865780	1.3230970

O	-2.3271300	-2.3360240	0.4226930
O	-2.7923100	-3.7370030	2.1865980
C	-4.2390650	-3.4729030	2.2907340
C	-4.6761400	-4.4756540	3.3621970
H	-4.4640790	-5.5015480	3.0434690
H	-5.7521350	-4.3829800	3.5434120
H	-4.1501750	-4.2873290	4.3032000
C	-4.9305170	-3.7740420	0.9570510
H	-6.0162950	-3.6997720	1.0860000
H	-4.6179070	-3.0751000	0.1805360
H	-4.6961250	-4.7926450	0.6282010
C	-4.4633570	-2.0380500	2.7695960
H	-4.1202620	-1.3120430	2.0326200
H	-5.5335480	-1.8733410	2.9404440
H	-3.9320670	-1.8549300	3.7089630
O	0.2732170	-2.8147810	0.7787590
C	1.8355040	-2.5506460	4.1184180
C	3.0093490	-1.7719550	4.0730860
C	3.9055090	-1.7691140	5.1362500
C	3.6784570	-2.5270800	6.2943950
C	2.5112130	-3.2956130	6.3422580
C	1.6071280	-3.3075340	5.2812810
H	0.7065900	-3.9090370	5.3692830
H	2.3006380	-3.8952420	7.2256670
C	4.6457350	-2.4894140	7.4544010
H	4.5006600	-3.3435200	8.1246500
H	4.5186460	-1.5783950	8.0552900
H	5.6872420	-2.5039410	7.1116530
H	4.8057940	-1.1608440	5.0661710
H	3.2132630	-1.1773370	3.1907910
C	1.0748410	0.9873640	-0.2520730
C	2.1918830	1.9990750	-0.2701700
C	1.9265790	3.3647790	-0.3721580
C	3.0227350	4.2901220	-0.4903250
C	2.8411050	5.6720920	-0.7823650
C	3.9168480	6.5230920	-0.9004700
C	5.2376090	6.0442640	-0.7295000
C	5.4531910	4.7118730	-0.4673270
C	4.3658960	3.8030140	-0.3576090
C	4.5816660	2.4196970	-0.1329690
C	3.5254340	1.5423070	-0.1150160
H	3.6924070	0.4849440	0.0525550
H	5.5979680	2.0598470	0.0063200
H	6.4633800	4.3269730	-0.3513790
H	6.0770320	6.7283860	-0.8175480
H	3.7503970	7.5717500	-1.1311660
H	1.8373010	6.0552480	-0.9238970
C	0.5091560	3.8217230	-0.4025670
C	-0.3584140	3.2618950	-1.3371910
C	0.1220090	2.1862250	-2.2720790
H	-0.5964790	2.0025680	-3.0736380
H	1.0779180	2.4657350	-2.7206880
C	-1.6819100	3.7455570	-1.4678290
C	-2.1581460	4.7339600	-0.6447200
C	-1.3475550	5.2532390	0.3952730
C	-1.8515720	6.2161650	1.3107350

C	-1.0771340	6.6759360	2.3503360
C	0.2386620	6.1827080	2.5198640
C	0.7633910	5.2663330	1.6365800
C	0.0006850	4.7846210	0.5357620
H	1.7668570	4.8881660	1.7914430
H	0.8375590	6.5246140	3.3593170
H	-1.4757040	7.4058670	3.0495680
H	-2.8713020	6.5706740	1.1814340
H	-3.1713330	5.1091330	-0.7654760
H	-2.3193420	3.3317320	-2.2405030
H	1.4387950	-0.0037910	0.0241900
H	0.2775930	1.2700890	0.4423790
C	-0.9399220	0.0668290	-1.3145870
C	-1.6649640	-0.4231640	-2.5478850
C	-1.0573590	-1.4188720	-3.3090960
C	-1.5701300	-1.7653360	-4.6056470
C	-0.8995120	-2.6493600	-5.4965410
C	-1.4169820	-2.9255950	-6.7425790
C	-2.6370130	-2.3435700	-7.1625960
C	-3.3088630	-1.4811780	-6.3281810
C	-2.7908570	-1.1624570	-5.0437080
C	-3.4747870	-0.2906210	-4.1651630
C	-2.9532260	0.0845910	-2.9409710
C	-3.8076660	0.9064120	-2.0401100
C	-3.9751950	0.5441340	-0.6933160
C	-4.7935930	1.3117880	0.1207000
C	-5.4674800	2.4302800	-0.3607320
C	-5.3230800	2.7620850	-1.7049290
C	-4.5096490	2.0149780	-2.5447960
H	-4.4068460	2.3264240	-3.5788700
H	-3.4893860	-0.3312890	-0.2724870
H	-4.4606720	0.0638590	-4.4538150
H	-4.2444590	-1.0239860	-6.6407490
H	-3.0365710	-2.5766100	-8.1455810
H	-0.8835660	-3.5961020	-7.4104980
H	0.0373280	-3.1005130	-5.1905500
H	-0.6708170	-0.7664330	-0.6621000
H	-1.5158510	0.7736440	-0.7215210
H	-2.0830410	-3.7716220	-2.8780100
H	-2.1354790	-6.1786960	-2.4193430
H	-0.0567810	-7.3925100	-1.7725420
H	2.0755620	-6.1524940	-1.5446070
H	3.3380890	-4.0679680	-1.7162750
C	3.8494440	-1.5236420	-2.0536460
C	4.3802520	-0.8109540	-3.1416850
C	5.6717470	-0.3057110	-3.0758110
C	6.4667170	-0.5038500	-1.9503530
C	5.9377290	-1.2229890	-0.8801820
C	4.6458610	-1.7302220	-0.9154270
H	4.2429130	-2.2304750	-0.0418590
H	3.8176140	-0.6713320	-4.0588110
C	-2.9819430	2.7496070	3.2780000
C	-3.3877410	2.1126380	4.6119300
H	-3.8217860	2.9050310	2.5957480
H	-2.4667280	3.7100260	3.3847460
H	-3.1289380	2.7162430	5.4880140

H	-4.4562730	1.8850690	4.6809380
N	-1.8053910	0.7101770	3.5305840
Cl	-0.5193370	-0.7822740	3.2499500
C	-2.5958940	0.7941690	4.6670730
O	-2.6705980	-0.0367520	5.5522950
C	-2.0043600	1.7397180	2.6545440
O	-1.4999970	1.8674700	1.5401790
F	-4.9547120	0.9935110	1.4167250
F	-6.2264650	3.1738980	0.4540140
F	-5.9653010	3.8478100	-2.1695250
F	6.7071280	-1.3954850	0.2055280
F	7.7089830	-0.0064400	-1.8940040
F	6.1876090	0.3742830	-4.1127640

159

S_MajorCl_5_MaruokaCF3_TS_Diffuse.log

C	2.9235260	0.6935400	2.2308140
C	3.2102220	2.0094170	2.5365890
C	2.2381600	2.8901760	3.0659960
C	2.5399370	4.2543920	3.3266460
C	1.5837390	5.0984330	3.8410110
C	0.2817490	4.6110170	4.1078810
C	-0.0363080	3.2912180	3.8776930
C	0.9343480	2.3843290	3.3670130
C	0.6574270	1.0011280	3.0984100
C	1.5985790	0.1999860	2.4592760
C	1.1858310	-1.1975980	2.0685090
H	2.0219580	-1.7825790	1.6846710
H	0.7665470	-1.7206650	2.9314660
N	0.0951820	-1.3034180	0.9758570
O	2.3964220	1.5805230	-0.7715420
C	1.5973280	2.4986230	-0.9029950
C	1.3754650	3.4797910	-1.9318660
C	0.4312700	4.5157710	-1.3467770
H	0.9596540	5.4299690	-1.0366430
H	-0.3930230	4.8013960	-2.0047080
N	-0.1080450	3.8187630	-0.1658610
C	-1.4112850	3.7954710	0.2502480
O	-1.9103880	2.9268750	0.9539160
O	-1.9997600	4.9328560	-0.1807050
C	-3.2869690	5.3973280	0.3584850
C	-3.4778010	6.7349320	-0.3616080
H	-2.6432080	7.4107270	-0.1492240
H	-4.4058160	7.2117830	-0.0291750
H	-3.5337120	6.5843660	-1.4443430
C	-3.1639070	5.6032540	1.8718510
H	-4.0917840	6.0367020	2.2615110
H	-2.9815680	4.6540900	2.3800150
H	-2.3422300	6.2914140	2.0992150
C	-4.4074650	4.4242920	-0.0078560
H	-4.3269310	3.4957150	0.5555920
H	-5.3784070	4.8825350	0.2127710
H	-4.3763170	4.1893220	-1.0751540
O	0.6211560	2.6313290	0.0980560
C	2.4460840	3.9297450	-2.8407900
C	3.7621200	3.4396960	-2.7423850
C	4.7671300	3.9106520	-3.5795320

C	4.5085200	4.8858060	-4.5538350
C	3.2005190	5.3770700	-4.6487490
C	2.1873880	4.9113860	-3.8126360
H	1.1794090	5.3022510	-3.9303130
H	2.9668430	6.1344270	-5.3948160
C	5.6012330	5.3680090	-5.4789130
H	5.3096570	6.2875570	-5.9981300
H	5.8369950	4.6189330	-6.2477020
H	6.5313540	5.5700710	-4.9330640
H	5.7752140	3.5124760	-3.4726630
H	3.9722970	2.6900220	-1.9869090
C	0.6779930	-1.1207790	-0.4478710
C	1.4795330	-2.2992900	-0.9337530
C	0.8624710	-3.5044580	-1.2688940
C	1.6730930	-4.6375310	-1.6284290
C	1.1425690	-5.9484810	-1.7938100
C	1.9548940	-7.0084690	-2.1278200
C	3.3441600	-6.8200310	-2.3223030
C	3.8948940	-5.5721990	-2.1507440
C	3.0876680	-4.4608680	-1.7857660
C	3.6546330	-3.1808170	-1.5652050
C	2.8745160	-2.1400820	-1.1259620
H	3.3105460	-1.1623360	-0.9609260
H	4.7167160	-3.0329130	-1.7319960
H	4.9629870	-5.4153820	-2.2800040
H	3.9718820	-7.6639510	-2.5946700
H	1.5257060	-8.0003060	-2.2397600
H	0.0823280	-6.1141590	-1.6417830
C	-0.6217690	-3.5966390	-1.2083120
C	-1.2735990	-3.2322810	-0.0338760
C	-0.4882920	-2.7181040	1.1407090
H	-1.0997330	-2.6793420	2.0447260
H	0.3760080	-3.3575510	1.3343100
C	-2.6711250	-3.4166810	0.0972050
C	-3.4234210	-3.9078320	-0.9400090
C	-2.8176600	-4.1874650	-2.1909530
C	-3.5894950	-4.6231260	-3.3024330
C	-3.0035280	-4.8415420	-4.5276930
C	-1.6151470	-4.6213980	-4.6937910
C	-0.8368330	-4.2209180	-3.6313400
C	-1.4020970	-4.0123640	-2.3419080
H	0.2216210	-4.0439100	-3.7803150
H	-1.1603420	-4.7638550	-5.6699260
H	-3.6044720	-5.1671960	-5.3723340
H	-4.6587980	-4.7655000	-3.1668060
H	-4.4921190	-4.0591880	-0.8182470
H	-3.1441680	-3.1821880	1.0435110
H	1.2779490	-0.2122440	-0.4040210
H	-0.1860010	-0.9225900	-1.0898360
C	-0.9923690	-0.2131590	1.1022560
C	-1.5303550	-0.0529730	2.5041480
C	-0.6660630	0.4267280	3.4892750
C	-1.0410440	0.3854480	4.8750840
C	-0.1429220	0.6944050	5.9350830
C	-0.5446660	0.6104040	7.2496730
C	-1.8666100	0.2247850	7.5775840

C	-2.7583110	-0.0856600	6.5778540
C	-2.3681460	-0.0265150	5.2129260
C	-3.2742020	-0.3330980	4.1710940
C	-2.8947750	-0.3534740	2.8413740
C	-3.9629940	-0.5839880	1.8267720
C	-4.1274980	0.2720920	0.7262790
C	-5.1814250	0.0670800	-0.1687290
C	-6.0863470	-0.9755820	0.0153050
C	-5.9364880	-1.8188300	1.1166820
C	-4.8901560	-1.6229750	2.0149170
H	-4.7812730	-2.2933280	2.8605010
C	-6.8958990	-2.9664980	1.2806820
F	-6.7490700	-3.8764020	0.2817620
F	-6.7201670	-3.6238570	2.4452620
F	-8.1799010	-2.5520570	1.2334740
H	-6.8957940	-1.1303100	-0.6898440
C	-5.4048460	1.0256910	-1.3111410
F	-6.2547380	2.0199340	-0.9563610
F	-5.9642330	0.4032930	-2.3792210
F	-4.2659450	1.6054140	-1.7284000
H	-3.4561370	1.1151190	0.5842970
H	-4.3111970	-0.5272310	4.4318680
H	-3.7746250	-0.3891660	6.8161170
H	-2.1708050	0.1699770	8.6190770
H	0.1600520	0.8410120	8.0436840
H	0.8739590	0.9878050	5.7004740
H	-0.5318480	0.7124020	0.7517190
H	-1.7500260	-0.5006820	0.3763080
H	-1.0450670	2.9428100	4.0608270
H	-0.4775930	5.2881130	4.4894000
H	1.8197360	6.1418090	4.0310070
H	3.5386020	4.6193640	3.0999520
H	4.2193800	2.3815990	2.3826780
C	4.0773490	-0.1728590	1.8395500
C	4.5029700	-1.1884440	2.7097750
C	5.6644230	-1.9183300	2.4505370
C	6.4355180	-1.6389820	1.3224380
C	6.0105970	-0.6405740	0.4474520
C	4.8415890	0.0836660	0.6916590
H	4.5068930	0.7831920	-0.0653200
C	6.7831630	-0.3820850	-0.8211760
F	6.3628470	-1.1883040	-1.8309510
F	8.1025810	-0.6186630	-0.6598190
F	6.6403030	0.8872130	-1.2508610
H	7.3533280	-2.1842110	1.1345190
C	6.0725860	-3.0372820	3.3732160
F	5.5743300	-4.2255680	2.9589850
F	5.6240670	-2.8407060	4.6328820
F	7.4144180	-3.1721340	3.4330560
H	3.9438400	-1.3846450	3.6192340
C	-3.2436190	-0.5196510	-4.2981980
C	-2.9035860	0.1499330	-5.6326630
H	-4.2167650	-0.2003210	-3.9060850
H	-3.2381030	-1.6128720	-4.3122110
H	-2.4015240	-0.5389260	-6.3244690
H	-3.7550630	0.5842540	-6.1647450

N	-1.5008530	1.0342100	-3.9407970
Cl	-0.0983460	2.1193130	-3.0385390
C	-1.8965360	1.2520890	-5.2557660
O	-1.5051380	2.1380430	-5.9870900
C	-2.1704460	0.0060100	-3.3337160
O	-1.9903830	-0.4253270	-2.1975450

147

S_MajorCl_5_MaruokaF3_TS_Diffuse.log

C	2.6480560	-1.3960740	-2.4668470
C	2.9429050	-2.7345010	-2.3466090
C	1.9382550	-3.7293950	-2.3576590
C	2.2565830	-5.1009680	-2.1730570
C	1.2706300	-6.0592580	-2.1885000
C	-0.0790880	-5.6798900	-2.3816190
C	-0.4161770	-4.3591560	-2.5775690
C	0.5814560	-3.3455390	-2.5891610
C	0.2841570	-1.9546520	-2.7784650
C	1.2787240	-0.9952940	-2.6109910
C	0.8788620	0.4554750	-2.6351710
H	1.7433520	1.1178580	-2.5945020
H	0.3150020	0.6786020	-3.5441780
N	-0.0469960	0.9294590	-1.4893460
O	2.5049820	-1.3641410	0.8208880
C	1.7189310	-2.1045160	1.3966180
C	1.6375190	-2.6110800	2.7461940
C	0.6184640	-3.7416720	2.6959840
H	1.1174900	-4.7202570	2.6163670
H	-0.0781930	-3.7762610	3.5322750
N	-0.1241220	-3.4604780	1.4597430
C	-1.4878110	-3.3638140	1.3294140
O	-2.0627290	-2.7628630	0.4330940
O	-2.0527870	-4.1280010	2.2916430
C	-3.4979330	-4.4004480	2.3295830
C	-3.6306410	-5.2872980	3.5707180
H	-3.0091370	-6.1831940	3.4733660
H	-4.6727270	-5.5987400	3.6997590
H	-3.3171840	-4.7449610	4.4684960
C	-3.9114070	-5.1629920	1.0667430
H	-4.9739830	-5.4243830	1.1252880
H	-3.7499380	-4.5540130	0.1749760
H	-3.3362340	-6.0907450	0.9734590
C	-4.2848450	-3.0994580	2.5078590
H	-4.3134490	-2.5259060	1.5823150
H	-5.3136010	-3.3312910	2.8070480
H	-3.8330450	-2.4823980	3.2908910
O	0.6144930	-2.5534830	0.6536480
C	2.8252530	-2.7989280	3.6019630
C	4.1190310	-2.4429300	3.1773590
C	5.2305450	-2.7036880	3.9696590
C	5.1060250	-3.3112540	5.2275340
C	3.8202100	-3.6650020	5.6509490
C	2.7023240	-3.4177850	4.8582100
H	1.7200570	-3.7016040	5.2263140
H	3.6894650	-4.1438320	6.6192820
C	6.3142260	-3.5542700	6.0996920
H	6.0927100	-4.2677080	6.9003560

H	6.6594150	-2.6266520	6.5748150
H	7.1562630	-3.9515270	5.5210180
H	6.2183070	-2.4280530	3.6051130
H	4.2238600	-1.9935470	2.1986210
C	0.7091300	1.1760030	-0.1614430
C	1.5827430	2.4018710	-0.1660420
C	1.0518920	3.6911670	-0.2000230
C	1.9435150	4.8134210	-0.3390650
C	1.4844610	6.1378600	-0.5908840
C	2.3685420	7.1830980	-0.7351580
C	3.7636990	6.9685200	-0.6291370
C	4.2459190	5.7004590	-0.4053430
C	3.3616700	4.5954560	-0.2721590
C	3.8509520	3.2777360	-0.0824670
C	2.9841740	2.2145790	-0.0559780
H	3.3554150	1.2071370	0.0883980
H	4.9209340	3.1118100	0.0184810
H	5.3156890	5.5181300	-0.3391230
H	4.4492990	7.8044260	-0.7366180
H	1.9915850	8.1822130	-0.9364430
H	0.4201260	6.3209090	-0.6837350
C	-0.4238520	3.8657780	-0.1041410
C	-1.2505540	3.1569870	-0.9733120
C	-0.6578890	2.2422610	-2.0116030
H	-1.4065940	1.9436190	-2.7482210
H	0.1601970	2.7437010	-2.5341770
C	-2.6510110	3.3646400	-0.9616630
C	-3.2303290	4.2393950	-0.0775480
C	-2.4411940	4.9101080	0.8913140
C	-3.0346630	5.7605490	1.8646940
C	-2.2716370	6.3583080	2.8408240
C	-0.8778590	6.1176840	2.8899380
C	-0.2701420	5.3156450	1.9492840
C	-1.0208000	4.7048880	0.9039030
H	0.7949410	5.1302170	2.0150260
H	-0.2822930	6.5630800	3.6821380
H	-2.7367330	6.9996870	3.5845460
H	-4.1103020	5.9152950	1.8307070
H	-4.3042500	4.4067360	-0.0942090
H	-3.2676600	2.8337280	-1.6770670
H	1.3019780	0.2811620	0.0177270
H	-0.0725880	1.2242560	0.6008500
C	-1.1381930	-0.1085820	-1.1274280
C	-1.8386970	-0.7056740	-2.3243930
C	-1.0984620	-1.5347350	-3.1634210
C	-1.6417580	-1.9790410	-4.4190500
C	-0.8817190	-2.6856080	-5.3932160
C	-1.4436600	-3.0673330	-6.5912790
C	-2.7975640	-2.7733520	-6.8803280
C	-3.5583880	-2.0840450	-5.9659660
C	-3.0009940	-1.6608870	-4.7289370
C	-3.7701990	-0.9523440	-3.7788250
C	-3.2333440	-0.4715280	-2.5989700
C	-4.1672370	0.1814700	-1.6377030
C	-4.1723890	-0.1716730	-0.2786050
C	-5.0778050	0.4277150	0.5854220

```
C       -6.0098300        1.3577880        0.1321270
C       -6.0173960        1.6835050       -1.2219820
C       -5.1138520        1.1118450       -2.1053920
H       -5.1448020        1.4153880       -3.1464610
H       -3.4894970       -0.9133910        0.1207810
H       -4.8291410       -0.8101790       -3.9770190
H       -4.5975440       -1.8441640       -6.1777340
H       -3.2291820       -3.0875610       -7.8265100
H       -0.8413370       -3.5986720       -7.3227650
H        0.1573810       -2.9166930       -5.1908710
H       -0.6364590       -0.8810610       -0.5399690
H       -1.8032630        0.4240970       -0.4536750
H       -1.4564770       -4.0806360       -2.6953740
H       -0.8575450       -6.4374360       -2.3653760
H        1.5220530       -7.1054890       -2.0383020
H        3.2949090       -5.3759240       -2.0060690
H        3.9806010       -3.0417660       -2.2494580
C        3.7860560       -0.4347310       -2.5785490
C        3.9919060        0.2558790       -3.7844120
C        5.0667360        1.1211180       -3.9125890
C        5.9549450        1.3243330       -2.8597980
C        5.7450110        0.6324860       -1.6703090
C        4.6879750       -0.2498490       -1.5199360
H        4.5195310       -0.7260700       -0.5618930
H        3.3357670        0.1123190       -4.6359750
C       -2.7893740        2.0306030        3.7808170
C       -2.0961660        2.2840210        5.1265530
H       -3.7727280        1.5513910        3.8824800
H       -2.9237070        2.9229380        3.1607240
H       -1.6475990        3.2860040        5.1819100
H       -2.7402750        2.1657550        6.0070360
N       -0.9145080        0.5992620        3.9471870
Cl        0.3636070       -0.8419190        3.4484210
C       -0.9624530        1.2409740        5.1777200
O       -0.2371340        1.0311030        6.1308620
C       -1.8605170        1.0436740        3.0600150
O       -1.9657940        0.7170410        1.8786180
F        6.5731950        0.8785540       -0.6408400
F        6.9772930        2.1777540       -2.9841700
F        5.2725870        1.7826850       -5.0625650
F       -5.0943210        0.1016960        1.8872080
F       -6.8780050        1.9304800        0.9755730
F       -6.9130760        2.5880530       -1.6564750
159
S_MajorCl_6_MaruokaCF3_TS_Diffuse.log
C        2.8143730       -0.5374270        2.5434590
C        3.0674610        0.4493300        3.4749000
C        2.0528380        0.9896530        4.2970970
C        2.3199260        2.0501140        5.2034030
C        1.3194590        2.5669140        5.9923060
C        0.0072550        2.0437720        5.9032290
C       -0.2778060        1.0038090        5.0473820
C        0.7367490        0.4359990        4.2285590
C        0.4938620       -0.6381360        3.3103310
C        1.4782580       -1.0429170        2.4147390
C        1.0995640       -2.0527050        1.3599120
```

H	1.9572640	-2.3710370	0.7687150
H	0.6523080	-2.9367660	1.8215100
N	0.0636560	-1.5834450	0.3139920
O	2.5948230	1.7488870	0.1505980
C	1.8523970	2.6698020	0.4688560
C	1.6118050	3.9847180	-0.0812710
C	0.8881640	4.7376440	1.0173830
H	1.5814920	5.3101770	1.6516300
H	0.1025260	5.4064440	0.6672050
N	0.2759720	3.6534350	1.8132960
C	-1.1007010	3.4357290	1.8341280
O	-1.6365620	2.3404200	1.8459910
O	-1.6921710	4.6371680	1.9722440
C	-3.1487750	4.8258240	1.8329990
C	-3.3089110	6.3287760	2.0755530
H	-2.7323110	6.9009240	1.3422710
H	-2.9641350	6.6003390	3.0788750
H	-4.3628920	6.6107800	1.9816630
C	-3.8971970	4.0269960	2.9051290
H	-3.8020790	2.9536390	2.7414570
H	-3.5062730	4.2671000	3.9002250
H	-4.9592920	4.2954870	2.8813780
C	-3.5649520	4.4542610	0.4102680
H	-4.6409020	4.6163260	0.2862990
H	-3.0340960	5.0661100	-0.3256110
H	-3.3514090	3.4060830	0.2010420
O	1.0143440	2.4597100	1.5692400
C	2.5997950	4.6823230	-0.9229040
C	3.5882020	3.9971120	-1.6577280
C	4.4988130	4.6901830	-2.4476800
C	4.4686730	6.0883420	-2.5545680
C	3.4850650	6.7683210	-1.8297430
C	2.5687590	6.0838270	-1.0329510
H	1.8126590	6.6533630	-0.4995940
H	3.4297830	7.8535240	-1.8894750
C	5.4454750	6.8247010	-3.4412820
H	6.4588640	6.4153280	-3.3520330
H	5.4899750	7.8898310	-3.1898200
H	5.1623000	6.7511850	-4.5003140
H	5.2538840	4.1301020	-2.9967370
H	3.6406160	2.9169630	-1.5942790
C	0.7098730	-0.6753340	-0.7626850
C	1.5902950	-1.4148920	-1.7373700
C	1.0507300	-2.2801760	-2.6898280
C	1.9356090	-3.0408540	-3.5328700
C	1.4818780	-4.0844540	-4.3890650
C	2.3616310	-4.7967390	-5.1727500
C	3.7460780	-4.5037960	-5.1518240
C	4.2232000	-3.5161470	-4.3227140
C	3.3448900	-2.7760930	-3.4853720
C	3.8339670	-1.7857050	-2.5967460
C	2.9832140	-1.1465110	-1.7288910
H	3.3586950	-0.3860970	-1.0551180
H	4.8928420	-1.5447730	-2.5969760
H	5.2862980	-3.2915720	-4.2839890
H	4.4282030	-5.0676210	-5.7821260

H	1.9898840	-5.5938290	-5.8107980
H	0.4258740	-4.3272880	-4.4133710
C	-0.4289910	-2.4160730	-2.7885720
C	-1.1591470	-2.7244970	-1.6437460
C	-0.4635400	-2.8846200	-0.3210330
H	-1.1228530	-3.3290200	0.4277970
H	0.4182860	-3.5205000	-0.4263970
C	-2.5509270	-2.9703990	-1.7249090
C	-3.2225230	-2.8600270	-2.9156690
C	-2.5412080	-2.4336920	-4.0829770
C	-3.2351330	-2.2307450	-5.3062000
C	-2.5795160	-1.7643940	-6.4216280
C	-1.1957930	-1.4741250	-6.3545410
C	-0.4906290	-1.6834730	-5.1905950
C	-1.1294040	-2.1885930	-4.0236140
H	0.5646150	-1.4411260	-5.1537370
H	-0.6862150	-1.0765200	-7.2278030
H	-3.1216600	-1.6048200	-7.3496760
H	-4.3022170	-2.4372680	-5.3372200
H	-4.2856740	-3.0770610	-2.9650480
H	-3.0852390	-3.2755640	-0.8327800
H	1.2805440	0.0729000	-0.2110360
H	-0.1239020	-0.1775190	-1.2678420
C	-1.0790840	-0.7376310	0.9262100
C	-1.6445880	-1.3170000	2.2034030
C	-0.8219250	-1.3446980	3.3276390
C	-1.2082790	-2.0729430	4.5053270
C	-0.3434460	-2.2726210	5.6180730
C	-0.7524790	-3.0055240	6.7098920
C	-2.0489950	-3.5714600	6.7563170
C	-2.9075890	-3.4046670	5.6953100
C	-2.5071030	-2.6685640	4.5476500
C	-3.3779100	-2.4802230	3.4504440
C	-2.9870060	-1.8229000	2.2981560
C	-4.0511150	-1.5768990	1.2816420
C	-4.3438970	-0.2818190	0.8285410
C	-5.4494150	-0.0594380	0.0013320
C	-6.2582590	-1.1148440	-0.4130890
C	-5.9616350	-2.4063920	0.0218180
C	-4.8812710	-2.6325280	0.8719670
H	-4.6771510	-3.6400730	1.2201540
C	-6.7683480	-3.5726730	-0.4823080
F	-8.0252700	-3.2154400	-0.8122200
F	-6.2061220	-4.1133920	-1.5949260
F	-6.8468770	-4.5632440	0.4316910
H	-7.1138190	-0.9327660	-1.0512570
C	-5.7848940	1.3520310	-0.4089550
F	-5.8985830	2.1585720	0.6701880
F	-6.9463970	1.4222780	-1.0912450
F	-4.8243500	1.8848900	-1.1984780
H	-3.7329850	0.5572040	1.1521090
H	-4.4007150	-2.8368090	3.5379730
H	-3.9038710	-3.8393690	5.7142730
H	-2.3592970	-4.1409820	7.6277600
H	-0.0722820	-3.1524150	7.5440890
H	0.6542420	-1.8505320	5.5984510

H	-0.6634350	0.2579400	1.0993280
H	-1.8134420	-0.6509450	0.1263940
H	-1.2921370	0.6295810	4.9742860
H	-0.7845890	2.4740670	6.5098470
H	1.5287930	3.3859010	6.6746520
H	3.3275960	2.4551950	5.2489860
H	4.0823750	0.8192490	3.5927570
C	3.9849630	-1.1076860	1.8173620
C	4.2892660	-2.4735530	1.9084050
C	5.4361050	-2.9928070	1.3058200
C	6.3045170	-2.1576740	0.6045670
C	6.0093220	-0.7973840	0.5140270
C	4.8644030	-0.2712470	1.1143000
H	4.6192990	0.7797820	1.0045930
C	6.8816420	0.1038820	-0.3201460
F	6.3830550	0.2372100	-1.5769710
F	8.1355770	-0.3813980	-0.4459210
F	6.9693850	1.3425230	0.1997120
H	7.1941630	-2.5601350	0.1352250
C	5.7678050	-4.4516940	1.4798020
F	4.6506810	-5.2142640	1.5137060
F	6.4297390	-4.6769050	2.6376230
F	6.5474870	-4.9140470	0.4804050
H	3.6402460	-3.1357860	2.4731040
C	-3.2361810	1.7018890	-3.8702050
C	-3.5129260	3.1998990	-4.0350440
H	-4.0977520	1.1168500	-3.5403310
H	-2.8507120	1.2298430	-4.7808550
H	-3.4557070	3.5624820	-5.0657540
H	-4.4899570	3.4960410	-3.6382350
N	-1.6885580	2.9035890	-2.5442430
Cl	-0.1198490	3.3435540	-1.4005360
C	-2.4232390	3.8862350	-3.1910780
O	-2.2618790	5.0874510	-3.0900340
C	-2.1362410	1.6372910	-2.8000690
O	-1.7399810	0.5949910	-2.2842990

147

S_MajorCl_6_MaruokaF3_TS_Diffuse.log

C	2.6765300	-1.0762800	-2.6392790
C	3.1764850	-2.3600570	-2.5572760
C	2.3413090	-3.4993500	-2.5691000
C	2.8750070	-4.8070730	-2.4158070
C	2.0499410	-5.9065150	-2.4224220
C	0.6533600	-5.7395950	-2.5810850
C	0.1091070	-4.4861150	-2.7465020
C	0.9345480	-3.3279180	-2.7578580
C	0.4223140	-1.9964670	-2.9082140
C	1.2593500	-0.8927750	-2.7552250
C	0.6295390	0.4781950	-2.7732590
H	1.3723150	1.2745250	-2.7619350
H	0.0137600	0.5950280	-3.6685170
N	-0.3184390	0.8116750	-1.5980990
O	2.7052020	-1.0364240	0.6275740
C	2.1350830	-1.9295090	1.2360750
C	2.2630480	-2.4393040	2.5861730
C	1.5182100	-3.7593580	2.5792920

H	2.1826290	-4.6109210	2.3673680
H	0.9605630	-3.9688710	3.4913950
N	0.5699610	-3.5995300	1.4611820
C	-0.8038920	-3.4464910	1.6345080
O	-1.5170840	-2.7881770	0.8987860
O	-1.1927660	-4.2335740	2.6539740
C	-2.5654050	-4.1899470	3.1998190
C	-2.4932180	-5.2060260	4.3429660
H	-1.7402500	-4.9056990	5.0789270
H	-2.2331500	-6.2006000	3.9642180
H	-3.4636790	-5.2690800	4.8474950
C	-3.5743020	-4.6466680	2.1408020
H	-3.6350240	-3.9380180	1.3147800
H	-3.2925890	-5.6283220	1.7430730
H	-4.5657680	-4.7372070	2.5999170
C	-2.8532110	-2.7866240	3.7373940
H	-3.8464600	-2.7701810	4.2011580
H	-2.1114390	-2.5101210	4.4929140
H	-2.8300330	-2.0389450	2.9442500
O	1.1131900	-2.6282200	0.5670200
C	3.5031720	-2.2911520	3.3664780
C	4.3881190	-1.2110790	3.1697860
C	5.5463160	-1.0885930	3.9284510
C	5.8807510	-2.0236720	4.9199620
C	4.9980280	-3.0882360	5.1240240
C	3.8342910	-3.2220830	4.3665980
H	3.1714080	-4.0582510	4.5721990
H	5.2187350	-3.8265450	5.8926020
C	7.1501670	-1.8835440	5.7276360
H	8.0410250	-2.1072090	5.1244620
H	7.1553960	-2.5651900	6.5848510
H	7.2722430	-0.8631230	6.1114180
H	6.2062350	-0.2406450	3.7519600
H	4.1524430	-0.4681720	2.4170660
C	0.4502170	1.2399770	-0.3269450
C	1.0947340	2.5992170	-0.4365250
C	0.3336900	3.7716420	-0.4487580
C	0.9882720	5.0322910	-0.6853640
C	0.2746820	6.2423530	-0.9209150
C	0.9363210	7.4264900	-1.1566440
C	2.3507670	7.4732600	-1.1654990
C	3.0737790	6.3210300	-0.9644260
C	2.4213280	5.0781110	-0.7395430
C	3.1583510	3.8768640	-0.5838740
C	2.5105020	2.6716290	-0.4655240
H	3.0754320	1.7527950	-0.3613190
H	4.2445240	3.9195840	-0.5830450
H	4.1605550	6.3381610	-0.9884760
H	2.8601330	8.4161800	-1.3440450
H	0.3667620	8.3330760	-1.3422430
H	-0.8089650	6.2254350	-0.9268940
C	-1.1420060	3.6823060	-0.2570830
C	-1.8709210	2.8042000	-1.0563640
C	-1.1791350	1.9802850	-2.1092460
H	-1.8971790	1.5349030	-2.8007080
H	-0.4895030	2.6054250	-2.6808790

C	-3.2826370	2.7491930	-0.9649580
C	-3.9632930	3.5256530	-0.0617710
C	-3.2602930	4.3669760	0.8362340
C	-3.9497420	5.1228030	1.8231340
C	-3.2641320	5.8984500	2.7277060
C	-1.8502330	5.9395040	2.6875610
C	-1.1534850	5.2332460	1.7321470
C	-1.8290350	4.4403990	0.7589260
H	-0.0735170	5.2722380	1.7321740
H	-1.3076350	6.5273330	3.4227940
H	-3.8013120	6.4649650	3.4831850
H	-5.0347060	5.0616370	1.8569980
H	-5.0483530	3.4864530	-0.0107390
H	-3.8286030	2.0916220	-1.6309930
H	1.1812410	0.4551800	-0.1447120
H	-0.2882760	1.1968890	0.4786590
C	-1.1712120	-0.4071850	-1.1627710
C	-1.8425630	-1.1164280	-2.3176130
C	-1.0258640	-1.7956230	-3.2203650
C	-1.5581750	-2.3020230	-4.4552690
C	-0.7467010	-2.8488990	-5.4887410
C	-1.3053940	-3.2974510	-6.6648500
C	-2.7047540	-3.2344680	-6.8701130
C	-3.5172530	-2.7044410	-5.8957440
C	-2.9677990	-2.2156420	-4.6794710
C	-3.7873520	-1.6684320	-3.6668500
C	-3.2715740	-1.1207550	-2.5062340
C	-4.2507010	-0.6518190	-1.4837380
C	-4.1357710	-1.0263910	-0.1343010
C	-5.1106290	-0.6248970	0.7700860
C	-6.2103850	0.1335810	0.3755260
C	-6.3246190	0.4868450	-0.9656620
C	-5.3665820	0.1023690	-1.8924380
H	-5.4911040	0.4144010	-2.9237850
H	-3.3128190	-1.6365570	0.2268290
H	-4.8647540	-1.7097600	-3.8002820
H	-4.5926830	-2.6429960	-6.0433420
H	-3.1314890	-3.5995480	-7.8000660
H	-0.6659540	-3.7029590	-7.4438800
H	0.3267800	-2.9014050	-5.3493120
H	-0.4819600	-1.0773180	-0.6408560
H	-1.8663390	-0.0082730	-0.4286080
H	-0.9642650	-4.3763040	-2.8448320
H	0.0033570	-6.6097800	-2.5619290
H	2.4639180	-6.9027560	-2.2942040
H	3.9476610	-4.9184410	-2.2791610
H	4.2507020	-2.5051460	-2.4861510
C	3.6660360	0.0372800	-2.6938980
C	3.6940920	0.9234400	-3.7836020
C	4.6777260	1.9008890	-3.8565570
C	5.6546590	2.0153310	-2.8712880
C	5.6276190	1.1239990	-1.8002220
C	4.6503150	0.1431700	-1.6973010
H	4.6208450	-0.4977610	-0.8225640
H	2.9830370	0.8447390	-4.5994420
C	-2.3146070	2.3166430	4.0325280

C	-1.6931000	2.3410930	5.4308920
H	-3.4022150	2.1902770	4.0292830
H	-2.0778240	3.1899830	3.4186850
H	-1.0654460	3.2218710	5.6083410
H	-2.4164880	2.2943410	6.2508390
N	-0.8205210	0.4887290	4.2215490
Cl	0.6896440	-0.9695510	3.5152860
C	-0.7830510	1.0965970	5.4738160
O	-0.1347380	0.7314670	6.4339300
C	-1.6570840	1.1148540	3.3416150
O	-1.8520080	0.8072550	2.1650600
F	-7.3801150	1.2276410	-1.3466890
F	-7.1367750	0.5143360	1.2640520
F	-5.0299830	-0.9807890	2.0599240
F	4.7138710	2.7506370	-4.8962190
F	6.5939220	2.9674190	-2.9463710
F	6.5631700	1.2562020	-0.8468970

177

S_MajorCf00_ConstOpt.log

C	2.3896030	-1.2113990	2.6824430
C	2.9828710	-0.1758590	3.3738720
C	2.2034170	0.7696830	4.1051970
C	2.8168420	1.8722700	4.7812530
C	2.0520200	2.7570110	5.4986190
C	0.6376280	2.5934880	5.5688670
C	0.0255370	1.5434440	4.9320410
C	0.7981770	0.5924240	4.1904770
C	0.2045460	-0.5178530	3.5001800
C	0.9590810	-1.3187250	2.6557330
C	0.2614330	-2.3271660	1.7961600
H	0.9567330	-2.9546740	1.2189170
H	-0.3749950	-2.9852500	2.4104090
N	-0.6817400	-1.7035410	0.7375510
O	-0.9968000	2.7363410	2.2878930
C	-1.2746680	3.0670190	1.1533970
C	-0.7961430	4.1435390	0.2879640
C	-1.6233380	4.1287300	-0.9613830
H	-2.2953770	4.9979370	-0.9992960
H	-1.0226330	4.0874840	-1.8741140
N	-2.4221700	2.8938170	-0.8298410
C	-2.4808770	1.9044040	-1.8079430
O	-2.5968450	0.7121380	-1.5621710
O	-2.5200870	2.5061980	-2.9948290
C	-2.6652180	1.7574230	-4.2658900
C	-4.0518250	1.1119480	-4.3026680
H	-4.1496290	0.3616270	-3.5137080
H	-4.1972520	0.6182250	-5.2702140
H	-4.8346400	1.8676350	-4.1803750
C	-1.5362220	0.7401420	-4.4188450
H	-1.5805520	0.3030440	-5.4235490
H	-0.5716340	1.2327500	-4.2852060
H	-1.6343420	-0.0698670	-3.6908620
C	-2.5322670	2.8721650	-5.3057180
H	-1.5530340	3.3503460	-5.2174230
H	-2.6345520	2.4547820	-6.3130180
H	-3.3104290	3.6290620	-5.1627370

O	-2.2433740	2.3468040	0.4715750
C	-0.0665910	5.3077150	0.7417180
C	0.0195790	5.6405050	2.1085110
C	0.6621000	6.8075860	2.5081360
C	1.2607510	7.6663860	1.5765050
C	1.1895190	7.3202120	0.2174440
C	0.5365050	6.1694540	-0.1994930
H	0.5340930	5.9045850	-1.2519600
H	1.6624920	7.9603580	-0.5236190
C	1.9888120	8.9128790	2.0164250
H	1.7507020	9.7657520	1.3700410
H	3.0766520	8.7702350	1.9701270
H	1.7347230	9.1858610	3.0456950
H	0.7003330	7.0588870	3.5656730
H	-0.4300130	4.9838250	2.8443490
C	0.1449590	-1.1100310	-0.4469510
C	0.7915890	-2.2157890	-1.2310190
C	0.0507110	-3.0905370	-2.0119180
C	0.6912660	-4.2293030	-2.6128970
C	-0.0452110	-5.2693480	-3.2668590
C	0.6022200	-6.3434920	-3.8230500
C	2.0230080	-6.4421330	-3.7614450
C	2.7554240	-5.4690900	-3.1303900
C	2.1040850	-4.3447310	-2.5294250
C	2.8543670	-3.3374630	-1.8435170
C	2.2141420	-2.3140420	-1.1984370
H	2.7739150	-1.5152710	-0.6786720
H	3.9428920	-3.3996890	-1.8476520
H	3.8420070	-5.5339700	-3.0721680
H	2.5101750	-7.3021680	-4.2197100
H	0.0431930	-7.1368350	-4.3179690
H	-1.1329590	-5.1958620	-3.3112620
C	-1.3937570	-2.8283860	-2.1933350
C	-2.1902940	-2.6711110	-1.0680730
C	-1.6052300	-2.8604040	0.2991020
H	-2.3706420	-2.9474550	1.0832110
H	-0.9863100	-3.7720440	0.3186630
C	-3.5851850	-2.4180950	-1.2044720
C	-4.1580380	-2.3212540	-2.4454910
C	-3.3585890	-2.4422770	-3.6251330
C	-3.9455980	-2.3179310	-4.9256410
C	-3.1646240	-2.3885400	-6.0506760
C	-1.7564100	-2.5775460	-5.9344570
C	-1.1720830	-2.7121700	-4.7007530
C	-1.9621320	-2.6723700	-3.5053730
H	-0.0907400	-2.8401750	-4.6146690
H	-1.1548750	-2.6090650	-6.8424210
H	-3.6017260	-2.2970780	-7.0444500
H	-5.0214410	-2.1627240	-4.9989180
H	-5.2273340	-2.1379190	-2.5527670
H	-4.1895260	-2.3035870	-0.3081210
H	0.8831220	-0.4253210	-0.0077490
H	-0.5746600	-0.5357180	-1.0494750
C	-1.5327120	-0.5140920	1.2645090
C	-2.1040100	-0.7920400	2.6254940
C	-1.2300970	-0.8341870	3.7029720

C	-1.6802990	-1.2192160	5.0128280
C	-0.7876950	-1.4073030	6.1174340
C	-1.2646990	-1.7921150	7.3448930
C	-2.6596480	-2.0103500	7.5436720
C	-3.5396690	-1.8421820	6.5051390
C	-3.0655970	-1.4459270	5.2134790
C	-3.9652130	-1.2566550	4.1234750
C	-3.5108910	-0.9301160	2.8616930
C	-4.5508140	-0.6558260	1.8421240
C	-4.6740890	0.6267330	1.2765540
C	-5.7710540	0.9186630	0.4593550
C	-6.7120690	-0.0740330	0.1410240
C	-6.5697730	-1.3538480	0.6851300
C	-5.5050210	-1.6408350	1.5531340
H	-5.4168160	-2.6303410	2.0107930
C	-7.5606940	-2.4445870	0.3811170
F	-8.2755080	-2.8428070	1.4471100
F	-8.4921540	-2.1640100	-0.5428420
F	-7.0003740	-3.5804380	-0.0727750
H	-7.5503540	0.1603980	-0.5231160
C	-5.9831710	2.3085100	-0.0876060
F	-7.2641690	2.7227930	-0.0226170
F	-5.6730910	2.4404790	-1.3857110
F	-5.3124900	3.2938170	0.5229500
H	-3.9093380	1.3899610	1.4899760
H	-5.0358750	-1.3555750	4.3179740
H	-4.6081030	-2.0057860	6.6432950
H	-3.0084140	-2.3118000	8.5316500
H	-0.5866910	-1.9366030	8.1863890
H	0.2812020	-1.2404990	5.9685760
H	-0.8546860	0.3479410	1.3017210
H	-2.2803970	-0.3244790	0.4853270
H	-1.0607410	1.4370610	4.9544700
H	0.0518520	3.3242770	6.1254770
H	2.5089580	3.5999980	6.0162780
H	3.8965160	1.9993410	4.7006560
H	4.0697270	-0.0632620	3.3763800
C	3.2743510	-2.2502510	2.1053480
C	3.1320110	-3.5818400	2.5135490
C	4.0315310	-4.5571370	2.0533540
C	5.0970650	-4.2011090	1.2228980
C	5.2398030	-2.8617010	0.8218540
C	4.3271520	-1.8908060	1.2422830
H	4.3865380	-0.8356700	0.8999790
C	6.4261630	-2.4925810	-0.0302490
F	6.4101710	-1.2791580	-0.5913320
F	6.6420270	-3.3233480	-1.0676040
F	7.5887500	-2.5251080	0.6472420
H	5.8211630	-4.9509790	0.8899370
C	3.8301690	-5.9798730	2.4969840
F	4.0225900	-6.1752810	3.8129600
F	4.6125320	-6.9040670	1.9193610
F	2.5852310	-6.4465080	2.2847230
H	2.3321140	-3.8542730	3.2064370
C	5.2562190	4.6851560	0.9842870
C	4.4912050	4.9260310	2.1263380

C	3.3402050	4.1726980	2.3706460
C	2.9475180	3.1746490	1.4802250
C	3.7295530	2.9369860	0.3478900
C	4.8758300	3.6878650	0.0860500
H	5.4557430	3.4782520	-0.8054220
S	3.3162260	1.5914080	-0.7729580
O	3.0107360	0.4213070	0.0978020
O	4.4626890	1.4411720	-1.6876760
N	1.9117300	1.9160470	-1.5774410
F	0.6153970	2.7725360	-0.4408630
S	1.9354910	3.0640050	-2.7948560
O	2.6224460	4.3122320	-2.4146720
O	0.5388310	3.1607460	-3.2663290
C	2.8772570	2.3320590	-4.1497260
C	3.8709550	3.0925170	-4.7625070
C	4.5543600	2.5624750	-5.8581140
C	4.2449720	1.2850020	-6.3271200
C	3.2494120	0.5304110	-5.7000550
C	2.5590320	1.0522620	-4.6080640
H	1.7951220	0.4699340	-4.1023630
H	3.0140770	-0.4679430	-6.0599160
H	4.7823390	0.8735860	-7.1776500
H	5.3323420	3.1472400	-6.3416260
H	4.1010830	4.0781500	-4.3737170
H	2.0433010	2.6023670	1.6521480
H	2.7345220	4.3695430	3.2506490
H	4.7897360	5.7030780	2.8259520
H	6.1495250	5.2731820	0.7892170

177

S_MajorCf02_B3LYP_TS.log

C	3.5950990	-0.3125860	2.1238200
C	3.7227150	0.6193710	3.1340350
C	2.7328510	0.7564790	4.1507810
C	2.8409360	1.7635880	5.1624630
C	1.8624120	1.8933530	6.1150240
C	0.7259890	1.0321590	6.1011350
C	0.6053380	0.0531910	5.1478340
C	1.6218990	-0.1251530	4.1537400
C	1.5479670	-1.1404530	3.1395110
C	2.4571830	-1.1834130	2.0913340
C	2.2539180	-2.2127260	1.0205510
H	3.0877400	-2.2613350	0.3046850
H	2.1266400	-3.2118390	1.4691230
N	0.9971620	-1.9744810	0.1482230
O	2.3113390	2.0328140	0.2065390
C	1.4384650	2.5331230	0.8974530
C	0.9118610	3.8958720	0.9686810
C	0.0265610	3.9773550	2.1715580
H	0.4627960	4.6109820	2.9567490
H	-0.9776640	4.3342090	1.9226190
N	-0.0329160	2.5827510	2.6452220
C	-1.2083070	1.9269750	3.0092110
O	-1.3834800	0.7272250	2.8994030
O	-1.9999520	2.8273940	3.5989630
C	-3.3060630	2.4592340	4.1902790
C	-3.8756140	3.8235000	4.5809530

H	-3.9918220	4.4466650	3.6891860
H	-3.2141580	4.3371300	5.2872800
H	-4.8563680	3.6976310	5.0524440
C	-3.0489840	1.5820770	5.4200490
H	-2.5948920	0.6319580	5.1300430
H	-2.3886500	2.0950720	6.1280770
H	-3.9982950	1.3745090	5.9273320
C	-4.2026240	1.7736910	3.1587360
H	-5.1983210	1.6327360	3.5977360
H	-4.3014170	2.3969610	2.2669090
H	-3.8034400	0.8049960	2.8646800
O	0.8285580	1.7650890	1.8583300
C	1.4876500	5.0457350	0.3123990
C	2.4852570	4.9191760	-0.6811110
C	3.0207810	6.0464160	-1.2884420
C	2.5950900	7.3399480	-0.9443360
C	1.5937700	7.4637300	0.0294900
C	1.0473860	6.3446250	0.6461320
H	0.2600100	6.4772520	1.3815680
H	1.2326310	8.4522780	0.3029600
C	3.2116360	8.5567630	-1.5892710
H	4.1508060	8.8358550	-1.0919860
H	2.5443060	9.4229970	-1.5346590
H	3.4491520	8.3749390	-2.6434240
H	3.7866490	5.9238060	-2.0517950
H	2.8207400	3.9299330	-0.9657110
C	1.3021910	-0.8579660	-0.8962720
C	2.2344390	-1.3653130	-1.9617240
C	1.8213910	-2.2921270	-2.9061070
C	2.7707170	-2.8561320	-3.8281900
C	2.4400470	-3.9381240	-4.7061790
C	3.3773940	-4.4558520	-5.5642410
C	4.6992280	-3.9238570	-5.5972620
C	5.0499260	-2.8942610	-4.7610310
C	4.0940730	-2.3426520	-3.8498330
C	4.4491700	-1.2791080	-2.9605480
C	3.5472080	-0.8132190	-2.0415840
H	3.7937780	0.0210740	-1.3826340
H	5.4474170	-0.8470270	-3.0332320
H	6.0583200	-2.4816090	-4.7735390
H	5.4202720	-4.3511550	-6.2935890
H	3.1274230	-5.2803060	-6.2315420
H	1.4287590	-4.3482140	-4.6794820
C	0.4003400	-2.7006200	-2.9175960
C	-0.1546490	-3.1974850	-1.7476760
C	0.6870230	-3.3326740	-0.5132120
H	0.2081940	-3.9460970	0.2631990
H	1.6604520	-3.7864950	-0.7621250
C	-1.5056150	-3.6466060	-1.7342330
C	-2.2820370	-3.5610720	-2.8601680
C	-1.7558120	-2.9969280	-4.0644720
C	-2.5663660	-2.8798800	-5.2388470
C	-2.0606210	-2.3064060	-6.3776220
C	-0.7264140	-1.8045830	-6.3960480
C	0.0736910	-1.9135510	-5.2870310
C	-0.4115000	-2.5397920	-4.0932200

H	1.0886130	-1.5109300	-5.2961840
H	-0.3580830	-1.3276610	-7.3039480
H	-2.6682410	-2.2171390	-7.2776260
H	-3.5926950	-3.2451050	-5.2032130
H	-3.3157630	-3.9098990	-2.8523020
H	-1.9094510	-4.0705530	-0.8185110
H	1.7306800	-0.0220140	-0.3257990
H	0.3349880	-0.5395890	-1.3091030
C	-0.2524250	-1.4874120	0.9515020
C	-0.4463860	-2.2982390	2.2036920
C	0.4847420	-2.1698500	3.2270360
C	0.4509710	-3.0207300	4.3851880
C	1.4954220	-3.0447440	5.3663380
C	1.4152750	-3.8778160	6.4530810
C	0.2880570	-4.7340620	6.6282400
C	-0.7238160	-4.7400990	5.7030200
C	-0.6571070	-3.8868880	4.5543280
C	-1.6891640	-3.8831580	3.5733980
C	-1.5957620	-3.1281030	2.4187230
C	-2.7647670	-3.2278110	1.5115790
C	-3.5747360	-2.1191180	1.2027190
C	-4.7925230	-2.3296290	0.5437900
C	-5.1752200	-3.6124150	0.1192890
C	-4.3388240	-4.7009280	0.3809810
C	-3.1498410	-4.5139270	1.0989160
H	-2.5189840	-5.3721880	1.3471790
C	-4.6988430	-6.0906290	-0.0646230
F	-5.7959040	-6.2102920	-0.8275560
F	-3.7422200	-6.6931740	-0.7954830
F	-4.9167660	-6.9508560	0.9453010
H	-6.1266390	-3.7531590	-0.4042560
C	-5.7747770	-1.2074340	0.3073780
F	-6.9356650	-1.4132360	0.9693370
F	-6.1677190	-1.1007970	-0.9748810
F	-5.4222600	0.0247930	0.6610530
H	-3.2565010	-1.0942300	1.4767270
H	-2.5767290	-4.4924020	3.7642370
H	-1.5904260	-5.3898590	5.8222630
H	0.2500400	-5.3786800	7.5063240
H	2.2090670	-3.8987220	7.1997460
H	2.3588950	-2.3900720	5.2365870
H	-0.0887480	-0.4295840	1.1943630
H	-1.0894120	-1.5550750	0.2456750
H	-0.2839060	-0.5811320	5.1098080
H	-0.0499680	1.1765450	6.8522990
H	1.9293150	2.6606890	6.8860050
H	3.7033600	2.4283740	5.1497640
H	4.5926570	1.2789520	3.1718740
C	4.7170890	-0.3904970	1.1600750
C	5.4678100	-1.5645300	1.0324690
C	6.5878110	-1.5901720	0.1857650
C	6.9845380	-0.4397410	-0.5009530
C	6.2339530	0.7400300	-0.3596450
C	5.0974720	0.7669420	0.4523790
H	4.4701550	1.6702590	0.5423570
C	6.6903890	1.9690580	-1.1016730

F	6.6924280	1.8246560	-2.4385980
F	7.9520080	2.3362830	-0.8158250
F	5.9806250	3.0876640	-0.9006410
H	7.8727500	-0.4468720	-1.1406860
C	7.3588610	-2.8767560	0.0569440
F	6.5917510	-3.9493960	-0.2092490
F	8.0248480	-3.2257290	1.1702830
F	8.2950880	-2.9150220	-0.9034510
H	5.1888700	-2.4509900	1.6085340
C	-3.7597670	0.4392810	-5.0821080
C	-5.1364220	0.6686620	-5.0538060
C	-5.7564220	1.0494370	-3.8612300
C	-5.0095420	1.2026080	-2.6935550
C	-3.6323180	0.9680740	-2.7347670
C	-3.0002130	0.5864960	-3.9208860
H	-1.9306640	0.4026970	-3.9204610
S	-2.6296160	1.1544040	-1.2468720
O	-1.3316950	0.5225840	-1.5554950
O	-3.3854070	0.5735850	-0.1137110
N	-2.3108860	2.7639480	-1.1300460
F	-0.6196370	3.1352990	-0.2441550
S	-3.3709590	3.8412070	-0.4446160
O	-2.9912540	4.1830470	0.9436380
O	-4.7848070	3.4687630	-0.6526360
C	-3.0353710	5.2838380	-1.4672840
C	-1.7264570	5.6133110	-1.8271120
C	-1.5068710	6.7687170	-2.5762980
C	-2.5796390	7.5809090	-2.9550090
C	-3.8820470	7.2358460	-2.5897730
C	-4.1173660	6.0799150	-1.8430790
H	-5.1230050	5.7837130	-1.5639770
H	-4.7181520	7.8614650	-2.8913460
H	-2.4006750	8.4791880	-3.5408000
H	-0.4929380	7.0323270	-2.8650890
H	-0.9144990	4.9579670	-1.5345030
H	-5.4754240	1.5270360	-1.7717970
H	-6.8277610	1.2301640	-3.8371300
H	-5.7258300	0.5536790	-5.9597430
H	-3.2724420	0.1413180	-6.0072640

177

S_MajorCf04_B3LYP_TS.log

C	-3.5707570	1.0899820	1.4143620
C	-3.8874370	0.4682550	2.6026940
C	-3.0226070	0.5539660	3.7360780
C	-3.3127670	-0.1549920	4.9446730
C	-2.4758040	-0.0494000	6.0270830
C	-1.3103860	0.7679010	5.9581140
C	-1.0143180	1.4643620	4.8130560
C	-1.8712760	1.3802680	3.6690580
C	-1.5910750	2.0726470	2.4400870
C	-2.3396340	1.8209410	1.2997410
C	-1.8762630	2.3705780	-0.0136410
H	-2.5709350	2.1498010	-0.8383150
H	-1.7351060	3.4623190	0.0431000
N	-0.5224690	1.7923200	-0.4782010
O	0.5030640	-2.3333360	-2.8273580

C	-0.0031850	-3.0347300	-1.9817250
C	0.4526480	-4.2850350	-1.3417940
C	-0.6469980	-4.7688050	-0.4482690
H	-1.0150300	-5.7592640	-0.7436910
H	-0.3338440	-4.7784140	0.5994810
N	-1.7105060	-3.7735030	-0.6616870
C	-2.6255260	-3.3619660	0.2898860
O	-3.1658280	-2.2669650	0.2911250
O	-2.8822700	-4.4113730	1.0835890
C	-3.9024110	-4.3543710	2.1479330
C	-5.2841710	-4.1737260	1.5121530
H	-6.0590430	-4.2535840	2.2835080
H	-5.4695210	-4.9565480	0.7668550
H	-5.3703450	-3.2012110	1.0279970
C	-3.5701830	-3.2504800	3.1552310
H	-2.5486090	-3.3737850	3.5239080
H	-3.6468220	-2.2631150	2.6984910
H	-4.2667200	-3.3147950	3.9992760
C	-3.7729560	-5.7348740	2.7941380
H	-3.9653430	-6.5268320	2.0620120
H	-2.7658160	-5.8735870	3.1995120
H	-4.4950130	-5.8367360	3.6112900
O	-1.2476670	-2.7149630	-1.4854600
C	1.5237160	-5.1181010	-1.8311700
C	2.3728370	-4.6991990	-2.8802510
C	3.3863880	-5.5317760	-3.3384700
C	3.6052810	-6.7999070	-2.7779910
C	2.7709390	-7.2075870	-1.7251460
C	1.7511420	-6.3896830	-1.2600880
H	1.1341350	-6.7297620	-0.4341030
H	2.9292650	-8.1790820	-1.2630650
C	4.6888000	-7.7084210	-3.3035360
H	5.4931760	-7.1395500	-3.7810730
H	4.2905820	-8.4037820	-4.0551660
H	5.1272360	-8.3145240	-2.5034440
H	4.0260530	-5.1908550	-4.1493780
H	2.2227720	-3.7198630	-3.3195560
C	-0.7006630	0.3120100	-0.9354700
C	-1.4296820	0.2436340	-2.2451860
C	-0.8287180	0.6576360	-3.4240500
C	-1.5893670	0.6936090	-4.6441230
C	-1.0742020	1.2566970	-5.8560890
C	-1.8355740	1.2809730	-6.9972690
C	-3.1556500	0.7434390	-6.9964310
C	-3.6812170	0.2058960	-5.8489790
C	-2.9116650	0.1764710	-4.6421900
C	-3.4527020	-0.3717240	-3.4360030
C	-2.7342570	-0.3333910	-2.2710230
H	-3.1146290	-0.7995130	-1.3507350
H	-4.4408400	-0.8293020	-3.4686190
H	-4.6892610	-0.2070910	-5.8345530
H	-3.7339460	0.7701290	-7.9192090
H	-1.4449650	1.7099140	-7.9192100
H	-0.0640110	1.6698360	-5.8573320
C	0.5882160	1.0843990	-3.3903200
C	0.9585560	2.0939860	-2.5144470

C	-0.0610730	2.7266810	-1.6162700
H	0.3126310	3.6346540	-1.1221980
H	-0.9648750	2.9883820	-2.1901820
C	2.2940050	2.5898230	-2.5179650
C	3.2378630	2.0472850	-3.3498620
C	2.9070500	0.9498970	-4.2058380
C	3.8959120	0.3451420	-5.0452300
C	3.5750320	-0.7351050	-5.8279970
C	2.2569900	-1.2762930	-5.7998130
C	1.2884950	-0.7081100	-5.0113030
C	1.5796250	0.4448170	-4.2120780
H	0.2893320	-1.1501010	-4.9488900
H	2.0363780	-2.1543610	-6.4048660
H	4.3208030	-1.2019060	-6.4700780
H	4.9058400	0.7526000	-5.0389580
H	4.2580460	2.4317920	-3.3617310
H	2.5504160	3.4165880	-1.8598030
H	-1.2364460	-0.1927940	-0.1218630
H	0.3109640	-0.1115170	-1.0093580
C	0.5675050	1.7341130	0.6297250
C	0.5468380	2.9511300	1.5095300
C	-0.5161270	3.0950580	2.3919270
C	-0.6251440	4.2467590	3.2459880
C	-1.7752720	4.5104950	4.0595620
C	-1.8225630	5.6164750	4.8694930
C	-0.7256770	6.5266550	4.9175040
C	0.3824320	6.3116100	4.1393380
C	0.4493610	5.1708370	3.2751950
C	1.5773460	4.9519240	2.4331090
C	1.6283630	3.8916950	1.5485680
C	2.8415480	3.8127060	0.6996200
C	3.6830470	2.6883900	0.6899640
C	4.8859550	2.7315250	-0.0300540
C	5.2423780	3.8696170	-0.7638940
C	4.3871950	4.9804800	-0.7703210
C	3.1970900	4.9581410	-0.0353430
H	2.5333390	5.8267790	-0.0232950
C	4.7838580	6.1900590	-1.5708720
F	5.9167630	6.7787160	-1.1527830
F	5.0125600	5.9349250	-2.8713050
F	3.8963830	7.1981170	-1.6021350
H	6.1788610	3.9004420	-1.3298760
C	5.8023450	1.5330160	-0.0230700
F	5.4485280	0.5667520	-0.8807690
F	5.9244210	0.9198840	1.1616170
F	7.0805490	1.8002160	-0.3609650
H	3.3900550	1.7614690	1.2329550
H	2.4153830	5.6493900	2.5085340
H	1.2267790	7.0000200	4.1623720
H	-0.7886090	7.3904860	5.5790630
H	-2.6967510	5.8172890	5.4887320
H	-2.6182330	3.8179060	4.0259870
H	0.3755340	0.8235210	1.2177470
H	1.5103460	1.5870950	0.0933090
H	-0.1075980	2.0688870	4.7486000
H	-0.6509680	0.8135470	6.8247820

H	-2.6791680	-0.5948170	6.9482670
H	-4.1978680	-0.7879900	4.9807680
H	-4.8162100	-0.0966710	2.7033220
C	-4.5900920	1.0893130	0.3389670
C	-5.0467320	2.3090140	-0.1810900
C	-6.0611050	2.3257660	-1.1495820
C	-6.6532070	1.1338010	-1.5797360
C	-6.2074430	-0.0832070	-1.0434740
C	-5.1787680	-0.1115320	-0.0942630
H	-4.8100330	-1.0687070	0.3184570
C	-6.8632830	-1.3764600	-1.4510690
F	-6.0221440	-2.3920300	-1.6997960
F	-7.6224810	-1.3246730	-2.5590470
F	-7.6976050	-1.8666030	-0.5172720
H	-7.4587960	1.1511600	-2.3196840
C	-6.5053500	3.6563430	-1.6948560
F	-5.4984630	4.4147230	-2.1661710
F	-7.1024530	4.4460090	-0.7859340
F	-7.3777540	3.6247260	-2.7133500
H	-4.6202030	3.2462670	0.1872750
C	1.5182420	-1.4092680	6.6253660
C	1.4236440	-2.6782440	7.2013050
C	1.0733340	-3.7789320	6.4163550
C	0.8176690	-3.6140490	5.0548300
C	0.9160800	-2.3403830	4.4911720
C	1.2671870	-1.2341250	5.2645200
H	1.3511010	-0.2597180	4.7996500
S	0.5714900	-2.1467310	2.7252280
O	0.0521110	-0.7677350	2.5506090
O	-0.3323230	-3.2653650	2.3901640
N	1.9782530	-2.4211400	1.9275590
F	1.3022640	-3.0241250	0.1059020
S	3.1307180	-1.2412930	1.8811930
O	3.4788130	-0.7256690	3.2258150
O	2.8643810	-0.1771520	0.8744700
C	4.5142040	-2.2140210	1.2852830
C	5.7100390	-2.1619440	2.0007930
C	6.8214590	-2.8525940	1.5138390
C	6.7267750	-3.5839520	0.3289040
C	5.5177380	-3.6324530	-0.3715130
C	4.4010300	-2.9435450	0.0991330
H	3.4441040	-2.9902090	-0.4080150
H	5.4391630	-4.2109120	-1.2880630
H	7.5943910	-4.1204400	-0.0476220
H	7.7596770	-2.8180550	2.0615960
H	5.7551810	-1.5927790	2.9228640
H	0.5345060	-4.4541990	4.4302060
H	0.9985140	-4.7669820	6.8632220
H	1.6254470	-2.8099350	8.2613390
H	1.7987200	-0.5546040	7.2347320

159

S_MajorCl_1_MaruokaCF3_TS_Diffuse.log

C	2.8090900	-0.4857900	2.5640400
C	3.0625430	0.5217560	3.4726890
C	2.0473330	1.0829210	4.2801090
C	2.3148630	2.1633750	5.1623450

C	1.3140370	2.7000550	5.9374010
C	0.0009840	2.1775350	5.8579140
C	-0.2846410	1.1189410	5.0254210
C	0.7301990	0.5305130	4.2216490
C	0.4865760	-0.5634660	3.3273030
C	1.4721340	-0.9907640	2.4435850
C	1.0939650	-2.0231160	1.4106830
H	1.9527480	-2.3566770	0.8294150
H	0.6429380	-2.8953630	1.8907190
N	0.0621070	-1.5749720	0.3516100
O	2.5988680	1.7424970	0.1246490
C	1.8602440	2.6742880	0.4217350
C	1.6371950	3.9819750	-0.1462340
C	0.8989740	4.7560020	0.9267490
H	1.5833360	5.3421590	1.5586330
H	0.1177960	5.4176710	0.5531030
N	0.2763610	3.6872710	1.7356270
C	-1.1006430	3.4776050	1.7559570
O	-1.6425530	2.3855340	1.7898620
O	-1.6871020	4.6848950	1.8650920
C	-3.1419060	4.8769210	1.7143690
C	-3.8992760	4.1071680	2.8014010
H	-3.8083100	3.0298230	2.6637340
H	-4.9600130	4.3797680	2.7660790
H	-3.5120230	4.3691100	3.7924030
C	-3.5533870	4.4738010	0.2987930
H	-4.6281330	4.6370560	0.1661800
H	-3.0168450	5.0663740	-0.4486260
H	-3.3430000	3.4201330	0.1152600
C	-3.2962900	6.3859050	1.9206150
H	-2.7138370	6.9380450	1.1766930
H	-4.3485550	6.6703710	1.8154310
H	-2.9546610	6.6794820	2.9187970
O	1.0100760	2.4860740	1.5181640
C	2.6282850	4.6595370	-0.9975470
C	3.6164990	3.9578340	-1.7168190
C	4.5291760	4.6332500	-2.5199930
C	4.5046650	6.0290780	-2.6519440
C	3.5164770	6.7250750	-1.9480580
C	2.5982740	6.0588830	-1.1387810
H	1.8360870	6.6392150	-0.6260910
H	3.4578890	7.8081720	-2.0366330
C	5.5190690	6.7519880	-3.5068990
H	6.4635440	6.9099040	-2.9674870
H	5.1532140	7.7372830	-3.8156410
H	5.7589140	6.1835280	-4.4129300
H	5.2794560	4.0603000	-3.0621660
H	3.6619440	2.8784240	-1.6373100
C	0.7126290	-0.6922620	-0.7437030
C	1.5933250	-1.4547480	-1.7002980
C	1.0542060	-2.3400230	-2.6344740
C	1.9394780	-3.1199810	-3.4592910
C	1.4858680	-4.1816270	-4.2930430
C	2.3659270	-4.9116110	-5.0598910
C	3.7505970	-4.6194840	-5.0436110
C	4.2275670	-3.6145200	-4.2355220

C	3.3489380	-2.8556500	-3.4155740
C	3.8377870	-1.8467120	-2.5479780
C	2.9865040	-1.1877720	-1.6955940
H	3.3616310	-0.4130240	-1.0381670
H	4.8970300	-1.6073630	-2.5515390
H	5.2908400	-3.3902040	-4.2002400
H	4.4330040	-5.1975530	-5.6605650
H	1.9942670	-5.7221690	-5.6807960
H	0.4296920	-4.4240960	-4.3132860
C	-0.4255110	-2.4757460	-2.7330880
C	-1.1586480	-2.7569300	-1.5831410
C	-0.4661000	-2.8888790	-0.2557280
H	-1.1277540	-3.3156180	0.5012810
H	0.4149050	-3.5283250	-0.3453590
C	-2.5507210	-3.0020520	-1.6616640
C	-3.2196990	-2.9174200	-2.8559890
C	-2.5351830	-2.5188540	-4.0311970
C	-3.2262460	-2.3424840	-5.2601630
C	-2.5674960	-1.9027790	-6.3845350
C	-1.1834130	-1.6134030	-6.3210940
C	-0.4810550	-1.7974090	-5.1511840
C	-1.1230670	-2.2749710	-3.9744140
H	0.5744850	-1.5559600	-5.1176740
H	-0.6713130	-1.2365890	-7.2020700
H	-3.1074020	-1.7635300	-7.3171560
H	-4.2936160	-2.5479290	-5.2887230
H	-4.2831240	-3.1336180	-2.9026490
H	-3.0874410	-3.2860690	-0.7639930
H	1.2838490	0.0670380	-0.2079010
H	-0.1192020	-0.2044570	-1.2614100
C	-1.0801770	-0.7130430	0.9417850
C	-1.6507060	-1.2616940	2.2302780
C	-0.8311290	-1.2660220	3.3571390
C	-1.2227710	-1.9661770	4.5500100
C	-0.3613840	-2.1432160	5.6692440
C	-0.7754980	-2.8498210	6.7763740
C	-2.0739390	-3.4104640	6.8322970
C	-2.9292960	-3.2652020	5.7655240
C	-2.5235520	-2.5567060	4.6024400
C	-3.3908990	-2.3908340	3.4988860
C	-2.9949090	-1.7613160	2.3328830
C	-4.0556090	-1.5358980	1.3079710
C	-4.3439890	-0.2508960	0.8242830
C	-5.4465830	-0.0452040	-0.0111870
C	-6.2566920	-1.1080630	-0.4036330
C	-5.9644370	-2.3898910	0.0619120
C	-4.8871120	-2.5987490	0.9202990
H	-4.6863540	-3.5984550	1.2922420
C	-6.7721980	-3.5658360	-0.4175120
F	-6.8572290	-4.5339190	0.5196920
F	-8.0267520	-3.2133890	-0.7612890
F	-6.2067490	-4.1346890	-1.5143900
H	-7.1099360	-0.9388640	-1.0484020
C	-5.7774960	1.3571340	-0.4551280
F	-6.9368510	1.4143360	-1.1422980
F	-4.8134670	1.8693110	-1.2544970

F	-5.8920680	2.1891100	0.6043040
H	-3.7321440	0.5940560	1.1302240
H	-4.4150260	-2.7422280	3.5917940
H	-3.9269950	-3.6962660	5.7917780
H	-2.3882690	-3.9590190	7.7156660
H	-0.0978580	-2.9798730	7.6154330
H	0.6377120	-1.7248600	5.6425750
H	-0.6626760	0.2852920	1.0935230
H	-1.8123860	-0.6430370	0.1383970
H	-1.2996120	0.7452030	4.9589640
H	-0.7910740	2.6230650	6.4531420
H	1.5237820	3.5340270	6.6012250
H	3.3232650	2.5674260	5.2004720
H	4.0780010	0.8922500	3.5838700
C	3.9790350	-1.0776580	1.8542220
C	4.2841020	-2.4394490	1.9896690
C	5.4332620	-2.9769510	1.4070950
C	6.3038010	-2.1636720	0.6838020
C	6.0085430	-0.8066080	0.5505020
C	4.8605320	-0.2629080	1.1283680
H	4.6170980	0.7850860	0.9888000
C	6.8899570	0.0722300	-0.2980090
F	6.3978620	0.1818770	-1.5596470
F	8.1418350	-0.4221080	-0.4075410
F	6.9820240	1.3211680	0.1960160
H	7.2022460	-2.5771290	0.2419130
C	5.7023990	-4.4533210	1.5364140
F	5.3499560	-4.9179930	2.7567910
F	7.0043650	-4.7500080	1.3463780
F	4.9940290	-5.1659690	0.6297210
H	3.6420900	-3.0804920	2.5862140
C	-3.5018300	3.1189130	-4.1124440
C	-3.2267010	1.6237880	-3.9186590
H	-3.4515860	3.4604760	-5.1506030
H	-4.4755690	3.4240790	-3.7143250
H	-4.0887990	1.0458550	-3.5780370
H	-2.8414010	1.1347020	-4.8203810
N	-1.6738970	2.8491850	-2.6234890
Cl	-0.1017880	3.3117890	-1.4720190
C	-2.1269960	1.5782580	-2.8475430
O	-1.7348190	0.5475940	-2.3070190
C	-2.4063110	3.8209120	-3.2895110
O	-2.2395060	5.0230950	-3.2161620

147
S_MajorCl_1_MaruokaF3_TS_Diffuse.log

C	2.4591850	-2.1259560	-2.1833090
C	2.3979180	-3.4916220	-1.9893540
C	1.1937330	-4.2198720	-2.1084520
C	1.1467480	-5.6144450	-1.8441270
C	-0.0325320	-6.3108660	-1.9620840
C	-1.2161520	-5.6348970	-2.3431080
C	-1.1992910	-4.2851950	-2.6148340
C	0.0074810	-3.5375760	-2.5219840
C	0.0847120	-2.1272360	-2.7798120
C	1.2579830	-1.4224630	-2.5262000
C	1.2192900	0.0791320	-2.6641960

H	2.2046710	0.5307870	-2.5606720
H	0.8126530	0.3572960	-3.6400070
N	0.3413100	0.8250650	-1.6382740
O	2.1896520	-1.6256640	1.1312760
C	1.2643750	-2.2721150	1.6316980
C	0.9154800	-2.6153130	2.9900910
C	-0.0744180	-3.7509460	2.8597070
H	0.4190480	-4.7339320	2.8986030
H	-0.8863270	-3.7395550	3.5892520
N	-0.6449390	-3.5413540	1.5161230
C	-1.9539150	-3.1055430	1.3242260
O	-2.3160740	-2.3507560	0.4382400
O	-2.7311250	-3.7815590	2.1920250
C	-4.1832500	-3.5566750	2.3146060
C	-4.8781000	-3.8994680	0.9952370
H	-4.5891220	-3.2062700	0.2047330
H	-5.9640500	-3.8498890	1.1318720
H	-4.6205510	-4.9167540	0.6821740
C	-4.4518370	-2.1235990	2.7690820
H	-5.5241500	-1.9995860	2.9571960
H	-3.9128850	-1.8995210	3.6937270
H	-4.1513730	-1.4016880	2.0099020
C	-4.5766600	-4.5524080	3.4091380
H	-4.0487310	-4.3290810	4.3409360
H	-5.6529440	-4.4921020	3.5998120
H	-4.3337090	-5.5761600	3.1081000
O	0.3014090	-2.7958690	0.7581460
C	1.8872030	-2.5941330	4.0907140
C	3.0343050	-1.7756000	4.0654030
C	3.9228920	-1.7629880	5.1336870
C	3.7184290	-2.5551240	6.2724880
C	2.5719380	-3.3554250	6.3039650
C	1.6756000	-3.3773160	5.2385490
H	0.7874430	-3.9985310	5.3144150
H	2.3717010	-3.9707800	7.1785340
C	4.7108920	-2.5570720	7.4101980
H	5.5088190	-3.2956270	7.2467430
H	4.2303270	-2.8056550	8.3633880
H	5.1962680	-1.5813960	7.5214390
H	4.7986870	-1.1189030	5.0840140
H	3.2179200	-1.1498170	3.2004270
C	1.0645510	0.9875260	-0.2777120
C	2.1783880	1.9980660	-0.2961700
C	1.9103680	3.3654490	-0.3901040
C	3.0059480	4.2898090	-0.5176510
C	2.8225620	5.6715600	-0.8080080
C	3.8981060	6.5215400	-0.9358080
C	5.2197040	6.0416860	-0.7744250
C	5.4365710	4.7095170	-0.5136520
C	4.3495480	3.8014220	-0.3956560
C	4.5663980	2.4181940	-0.1730570
C	3.5109170	1.5409710	-0.1507550
H	3.6775560	0.4837340	0.0161010
H	5.5831800	2.0580160	-0.0393720
H	6.4472820	4.3238510	-0.4050020
H	6.0590830	6.7252550	-0.8697010

H	3.7305510	7.5701030	-1.1656060
H	1.8178730	6.0553140	-0.9419390
C	0.4932790	3.8263390	-0.3986950
C	-0.3907480	3.2717620	-1.3198870
C	0.0727670	2.2030940	-2.2696550
H	-0.6605500	2.0255870	-3.0591590
H	1.0206110	2.4857530	-2.7335300
C	-1.7167560	3.7556350	-1.4253310
C	-2.1781140	4.7411120	-0.5906130
C	-1.3486360	5.2577530	0.4351730
C	-1.8365540	6.2193420	1.3607960
C	-1.0437440	6.6778700	2.3866280
C	0.2740020	6.1831970	2.5328050
C	0.7827890	5.2668830	1.6399740
C	0.0013810	4.7875380	0.5515800
H	1.7881030	4.8874090	1.7777410
H	0.8874560	6.5234520	3.3621830
H	-1.4295880	7.4073440	3.0934880
H	-2.8578880	6.5748660	1.2490300
H	-3.1929830	5.1169660	-0.6921560
H	-2.3683750	3.3447760	-2.1876880
H	1.4141730	-0.0167530	-0.0064570
H	0.2716570	1.2660690	0.4257400
C	-0.9744430	0.0777560	-1.3123070
C	-1.7153660	-0.4176090	-2.5327420
C	-1.1189920	-1.4119950	-3.3030630
C	-1.6549510	-1.7588580	-4.5877100
C	-0.9964290	-2.6378880	-5.4911840
C	-1.5358460	-2.9163400	-6.7268460
C	-2.7676220	-2.3421650	-7.1223320
C	-3.4288530	-1.4845590	-6.2745750
C	-2.8879060	-1.1624470	-5.0007060
C	-3.5589010	-0.2920370	-4.1100720
C	-3.0131300	0.0880100	-2.8990860
C	-3.8424540	0.9185210	-1.9807980
C	-3.9870330	0.5650490	-0.6286760
C	-4.7795960	1.3470750	0.1970800
C	-5.4517840	2.4691300	-0.2776350
C	-5.3330120	2.7911330	-1.6266110
C	-4.5432190	2.0318960	-2.4774700
H	-4.4571980	2.3373580	-3.5148220
H	-3.5032500	-0.3128300	-0.2107710
H	-4.5510210	0.0593380	-4.3799660
H	-4.3730080	-1.0331190	-6.5688680
H	-3.1845950	-2.5772930	-8.0974560
H	-1.0110620	-3.5827340	-7.4055400
H	-0.0509110	-3.0829100	-5.2034900
H	-0.6999410	-0.7579180	-0.6612850
H	-1.5498370	0.7833820	-0.7160160
H	-2.1199440	-3.7798990	-2.8806930
H	-2.1503870	-6.1852100	-2.4102600
H	-0.0629750	-7.3758860	-1.7501830
H	2.0595110	-6.1166100	-1.5333410
H	3.3089850	-4.0322570	-1.7485390
C	3.8083970	-1.4948750	-2.1233080
C	4.3269980	-0.8022680	-3.2295260

C 5.6234100 -0.3060450 -3.1899600
C 6.4326250 -0.4906710 -2.0720780
C 5.9130790 -1.1864250 -0.9821170
C 4.6179470 -1.6854450 -0.9916420
H 4.2234410 -2.1677540 -0.1041420
H 3.7532370 -0.6757580 -4.1415540
C -3.1928600 2.2323610 4.6639140
C -2.9289750 2.7231780 3.2426310
H -2.8200160 2.9108540 5.4365820
H -4.2466690 2.0484340 4.8824770
H -3.8312280 2.7657540 2.6309160
H -2.4496800 3.7035910 3.1887280
N -1.7070590 0.7267500 3.5910100
Cl -0.4894300 -0.8249500 3.3120070
C -1.9744040 1.6750340 2.6459890
O -1.5395300 1.7130960 1.5010050
C -2.4220700 0.9046950 4.7617470
O -2.4399730 0.1485950 5.7094680
F 6.6946420 -1.3422870 0.0973580
F 7.6793130 -0.0026140 -2.0412680
F 6.1296560 0.3519580 -4.2455050
F -4.9171180 1.0409210 1.4989350
F -6.1882830 3.2227350 0.5471530
F -5.9749960 3.8793450 -2.0855150
159
S_MajorCl_2_MaruokaCF3_TS_Diffuse.log
C 2.7969290 0.5693500 2.5469210
C 3.0345070 1.8452580 3.0152850
C 2.0113340 2.6473720 3.5692440
C 2.2675950 3.9768070 3.9997160
C 1.2632120 4.7452500 4.5387200
C -0.0431420 4.2142460 4.6615410
C -0.3183770 2.9249990 4.2647250
C 0.7015630 2.0951680 3.7224080
C 0.4712890 0.7465780 3.2891640
C 1.4686360 0.0355980 2.6287440
C 1.1169620 -1.3176770 2.0638150
H 1.9870550 -1.8361640 1.6626980
H 0.6702770 -1.9445920 2.8396410
N 0.0912330 -1.3287980 0.9086550
O 2.5254600 1.7176230 -0.5663780
C 1.7233950 2.6402370 -0.6512630
C 1.4491890 3.6268440 -1.6651980
C 0.5793890 4.6750960 -1.0021830
H 1.1607620 5.5513190 -0.6765440
H -0.2532790 5.0190750 -1.6177620
N 0.0529050 3.9627170 0.1765710
C -1.2763180 3.8395270 0.5146210
O -1.7536260 2.9184380 1.1616310
O -1.9140150 4.9534160 0.0944660
C -3.2364110 5.3427980 0.6118310
C -3.1550610 5.5263620 2.1303850
H -2.9286220 4.5795910 2.6253910
H -4.1137380 5.8987130 2.5086450
H -2.3787340 6.2552770 2.3873770
C -4.2988380 4.3202940 0.2070700

H	-5.2954200	4.7211320	0.4247770
H	-4.2410550	4.1118090	-0.8643190
H	-4.1729690	3.3850590	0.7528640
C	-3.4795660	6.6814810	-0.0900730
H	-3.4980420	6.5482210	-1.1763760
H	-4.4407820	7.1013960	0.2245260
H	-2.6889400	7.3971140	0.1571450
O	0.8233860	2.7941420	0.4131640
C	2.4416380	4.0287080	-2.6756010
C	3.5297550	3.2089400	-3.0367970
C	4.4448320	3.6213960	-3.9990340
C	4.3214590	4.8554180	-4.6540280
C	3.2389080	5.6666060	-4.3000840
C	2.3171800	5.2645750	-3.3350220
H	1.4825920	5.9211850	-3.1048600
H	3.1088680	6.6299620	-4.7893020
C	5.3052010	5.2749430	-5.7211670
H	6.3355600	5.0259020	-5.4403960
H	5.2588410	6.3534810	-5.9069110
H	5.1020440	4.7708180	-6.6759580
H	5.2775220	2.9663750	-4.2495720
H	3.6541890	2.2499150	-2.5485950
C	0.7402020	-0.9608820	-0.4497610
C	1.6261980	-2.0389760	-1.0178030
C	1.0947130	-3.2308730	-1.5120320
C	1.9882870	-4.2724780	-1.9473820
C	1.5450690	-5.5857480	-2.2742380
C	2.4326690	-6.5581140	-2.6768620
C	3.8148970	-6.2735630	-2.7831860
C	4.2821320	-5.0224020	-2.4565360
C	3.3957260	-4.0021290	-2.0166720
C	3.8752330	-2.7234760	-1.6360820
C	3.0171730	-1.7822860	-1.1232650
H	3.3861440	-0.8049160	-0.8364490
H	4.9323330	-2.4971010	-1.7390600
H	5.3435320	-4.7947150	-2.5168540
H	4.5032090	-7.0476420	-3.1109910
H	2.0688690	-7.5546800	-2.9116580
H	0.4910320	-5.8243440	-2.1919180
C	-0.3843270	-3.4042650	-1.5507390
C	-1.1238240	-3.1953060	-0.3893600
C	-0.4384820	-2.7747520	0.8800900
H	-1.1049630	-2.8583380	1.7411460
H	0.4412350	-3.3964190	1.0615690
C	-2.5138250	-3.4611540	-0.3644030
C	-3.1754630	-3.8821740	-1.4894370
C	-2.4852790	-3.9996750	-2.7215650
C	-3.1688560	-4.3567220	-3.9153240
C	-2.5059840	-4.4159620	-5.1190730
C	-1.1254150	-4.1089950	-5.1778430
C	-0.4299820	-3.7826710	-4.0350660
C	-1.0755150	-3.7383770	-2.7671630
H	0.6230290	-3.5370700	-4.1022170
H	-0.6106590	-4.1245720	-6.1344220
H	-3.0403540	-4.6825200	-6.0267990
H	-4.2340610	-4.5672880	-3.8590270

H	-4.2378180	-4.1036190	-1.4478160
H	-3.0549650	-3.3504130	0.5682650
H	1.3051010	-0.0453280	-0.2679520
H	-0.0983710	-0.7281330	-1.1135620
C	-1.0433470	-0.2957600	1.0985040
C	-1.6502140	-0.3038480	2.4818160
C	-0.8525960	0.1039180	3.5494200
C	-1.2872560	-0.0913790	4.9051290
C	-0.4503780	0.1395960	6.0329240
C	-0.9064640	-0.0901080	7.3118580
C	-2.2254970	-0.5520980	7.5364370
C	-3.0585970	-0.7921590	6.4691300
C	-2.6096810	-0.5830110	5.1369740
C	-3.4530610	-0.8195100	4.0275880
C	-3.0134480	-0.6931920	2.7221070
C	-4.0327040	-0.8631630	1.6462190
C	-4.2020740	0.0945360	0.6320540
C	-5.2349800	-0.0419460	-0.2974310
C	-6.1078620	-1.1282660	-0.2460530
C	-5.9441630	-2.0812040	0.7570990
C	-4.9239180	-1.9456930	1.6978210
H	-4.8128600	-2.6953560	2.4747570
C	-6.8162890	-3.3071820	0.7809030
F	-7.0343550	-3.7450230	2.0395560
F	-8.0159100	-3.0888630	0.2068860
F	-6.2380290	-4.3369730	0.1073880
H	-6.9027420	-1.2288500	-0.9752610
C	-5.4744650	1.0357480	-1.3242920
F	-6.0658440	0.5384200	-2.4380130
F	-4.3362790	1.6436630	-1.7053010
F	-6.2986870	1.9965490	-0.8428460
H	-3.5432910	0.9566230	0.5810500
H	-4.4911090	-1.0789170	4.2172690
H	-4.0716030	-1.1539690	6.6268990
H	-2.5732960	-0.7214600	8.5515500
H	-0.2469460	0.0835970	8.1574610
H	0.5635240	0.4897820	5.8786850
H	-0.6033780	0.6784160	0.8749790
H	-1.7539730	-0.5262700	0.3071700
H	-1.3291830	2.5438060	4.3407000
H	-0.8391810	4.8342380	5.0642470
H	1.4649910	5.7636180	4.8589510
H	3.2713260	4.3774360	3.8829530
H	4.0437220	2.2460220	2.9745300
C	3.9776720	-0.2240310	2.0993130
C	4.3232230	-1.4183880	2.7469940
C	5.4848510	-2.1104130	2.3994630
C	6.3258420	-1.6201120	1.4021990
C	5.9884680	-0.4301930	0.7562960
C	4.8287480	0.2668100	1.0981240
H	4.5531150	1.1725000	0.5681810
C	6.8357930	0.0645240	-0.3866170
F	6.3518550	-0.3785380	-1.5765820
F	8.1106180	-0.3719510	-0.2961020
F	6.8638080	1.4086660	-0.4508910
H	7.2339890	-2.1495860	1.1403940

C	5.8000710	-3.4129810	3.0870940
F	5.4640560	-3.3782740	4.3966700
F	7.1105130	-3.7229740	3.0092970
F	5.1133420	-4.4410040	2.5363680
H	3.7031400	-1.7935400	3.5555550
C	-3.1024960	0.7470840	-5.3944990
C	-3.1647500	-0.2932440	-4.2703350
H	-2.8061420	0.3335480	-6.3638910
H	-4.0443340	1.2840290	-5.5472920
H	-4.1458700	-0.3628640	-3.7931720
H	-2.8852630	-1.3028980	-4.5877960
N	-1.5418830	1.3296210	-3.7050100
Cl	-0.1200850	2.3438190	-2.7251140
C	-2.1398490	0.1961820	-3.2356140
O	-1.9145620	-0.3702700	-2.1671250
C	-2.0306800	1.7519100	-4.9361910
O	-1.6825410	2.7407630	-5.5472780

147

S_MajorCl_2_MaruokaF3_TS_Diffuse.log

C	2.5108910	-2.1238240	-2.1584910
C	2.4474460	-3.4928190	-1.9926050
C	1.2436040	-4.2179230	-2.1377420
C	1.1908330	-5.6177110	-1.9014280
C	0.0088100	-6.3071560	-2.0329430
C	-1.1721170	-5.6198310	-2.4022380
C	-1.1480230	-4.2669470	-2.6555580
C	0.0617670	-3.5264760	-2.5479240
C	0.1446640	-2.1145770	-2.7885200
C	1.3141480	-1.4138550	-2.5079890
C	1.2765830	0.0893000	-2.6311540
H	2.2604830	0.5394140	-2.5068830
H	0.8874800	0.3761750	-3.6113150
N	0.3756730	0.8290600	-1.6170120
O	2.1649850	-1.6563650	1.1302500
C	1.2388310	-2.2851920	1.6272320
C	0.8774750	-2.6080430	2.9877610
C	-0.1286470	-3.7339010	2.8690330
H	0.3478040	-4.7239160	2.9335150
H	-0.9444410	-3.6915740	3.5900390
N	-0.6774330	-3.5423270	1.5104550
C	-1.9819360	-3.1015900	1.2941190
O	-2.3237270	-2.3420800	0.4034650
O	-2.7780050	-3.7665340	2.1513050
C	-4.2261150	-3.5129780	2.2592540
C	-4.9168930	-3.8026690	0.9226460
H	-4.6096620	-3.0924820	0.1542490
H	-6.0030220	-3.7372250	1.0534520
H	-4.6760210	-4.8156890	0.5814420
C	-4.4593960	-2.0853760	2.7553980
H	-5.5305770	-1.9292380	2.9281090
H	-3.9293870	-1.9108530	3.6971540
H	-4.1205380	-1.3479210	2.0278290
C	-4.6561820	-4.5310940	3.3190520
H	-4.1304190	-4.3509000	4.2617680
H	-5.7325520	-4.4471740	3.5023290
H	-4.4381680	-5.5517880	2.9878840

O	0.2803790	-2.8106740	0.7503870
C	1.8508500	-2.5952840	4.0910260
C	3.0226660	-1.8124760	4.0557890
C	3.9174370	-1.8187010	5.1199860
C	3.6915430	-2.5902460	6.2694180
C	2.5267800	-3.3631550	6.3072590
C	1.6239370	-3.3661450	5.2453020
H	0.7254410	-3.9718490	5.3252500
H	2.3173190	-3.9734810	7.1836030
C	4.6570640	-2.5620800	7.4311190
H	5.6990430	-2.5639840	7.0895410
H	4.5175360	-3.4267500	8.0889350
H	4.5224990	-1.6607750	8.0449480
H	4.8159060	-1.2068840	5.0577810
H	3.2257480	-1.2074040	3.1804000
C	1.0705630	0.9954300	-0.2425220
C	2.1833220	2.0119610	-0.2503840
C	1.9126260	3.3774010	-0.3411840
C	3.0052330	4.3081250	-0.4496510
C	2.8185490	5.6917750	-0.7303270
C	3.8910520	6.5480660	-0.8393540
C	5.2134350	6.0731180	-0.6701460
C	5.4339240	4.7394290	-0.4188790
C	4.3501240	3.8253090	-0.3187230
C	4.5711020	2.4410410	-0.1052720
C	3.5184180	1.5592730	-0.0965750
H	3.6892110	0.5012430	0.0626800
H	5.5885980	2.0841470	0.0329320
H	6.4454530	4.3576100	-0.3044390
H	6.0502450	6.7613210	-0.7509510
H	3.7207620	7.5979570	-1.0614860
H	1.8134690	6.0721110	-0.8704450
C	0.4934200	3.8289480	-0.3698110
C	-0.3706740	3.2736150	-1.3103050
C	0.1149400	2.2075650	-2.2534440
H	-0.6020270	2.0277340	-3.0572270
H	1.0701430	2.4946460	-2.6987750
C	-1.6957040	3.7535180	-1.4390730
C	-2.1770980	4.7331030	-0.6084670
C	-1.3701400	5.2465090	0.4372730
C	-1.8793350	6.1994760	1.3602730
C	-1.1083050	6.6532460	2.4050390
C	0.2091770	6.1637670	2.5723220
C	0.7388550	5.2571500	1.6819430
C	-0.0202690	4.7819430	0.5758280
H	1.7435840	4.8816280	1.8350260
H	0.8054750	6.5007890	3.4155930
H	-1.5107930	7.3755750	3.1099020
H	-2.9002250	6.5511640	1.2323930
H	-3.1915290	5.1053810	-0.7278110
H	-2.3301670	3.3439390	-2.2164080
H	1.4384710	0.0035120	0.0261580
H	0.2713670	1.2691120	0.4531890
C	-0.9391110	0.0755560	-1.3146940
C	-1.6617170	-0.4066200	-2.5525050
C	-1.0497000	-1.3925430	-3.3228220

C	-1.5610250	-1.7290820	-4.6225620
C	-0.8865890	-2.6019520	-5.5215170
C	-1.4026970	-2.8687440	-6.7701790
C	-2.6250900	-2.2879560	-7.1849770
C	-3.3007180	-1.4362540	-6.3426590
C	-2.7843030	-1.1273560	-5.0551600
C	-3.4722280	-0.2668320	-4.1685610
C	-2.9523580	0.0989000	-2.9408600
C	-3.8098140	0.9083280	-2.0317140
C	-3.9763940	0.5307940	-0.6890020
C	-4.7950410	1.2887440	0.1336960
C	-5.4702480	2.4117780	-0.3349960
C	-5.3276030	2.7580700	-1.6756830
C	-4.5139540	2.0209660	-2.5241610
H	-4.4120470	2.3437040	-3.5548690
H	-3.4896880	-0.3489540	-0.2782030
H	-4.4596150	0.0861500	-4.4539610
H	-4.2382110	-0.9801590	-6.6511270
H	-3.0235130	-2.5134800	-8.1701740
H	-0.8663770	-3.5307660	-7.4442090
H	0.0520650	-3.0520250	-5.2194870
H	-0.6669150	-0.7620470	-0.6691330
H	-1.5185370	0.7748780	-0.7162360
H	-2.0659710	-3.7531240	-2.9143230
H	-2.1091910	-6.1643580	-2.4771720
H	-0.0261120	-7.3756790	-1.8397850
H	2.1010910	-6.1292620	-1.5990760
H	3.3553420	-4.0381960	-1.7503640
C	3.8565380	-1.4887360	-2.0635450
C	4.3864890	-0.7653850	-3.1449430
C	5.6756750	-0.2551860	-3.0724600
C	6.4691140	-0.4586760	-1.9468860
C	5.9409800	-1.1883060	-0.8834270
C	4.6513660	-1.7007520	-0.9252420
H	4.2483100	-2.2087680	-0.0561740
H	3.8250530	-0.6209400	-4.0620660
C	-3.4176870	2.0594870	4.6194480
C	-3.0046780	2.7145580	3.2962880
H	-3.1780890	2.6573780	5.5047770
H	-4.4840500	1.8175080	4.6718710
H	-3.8398940	2.8777820	2.6103400
H	-2.4919990	3.6743800	3.4199220
N	-1.8160040	0.6824150	3.5370540
Cl	-0.5176470	-0.8104610	3.2419310
C	-2.0196600	1.7161630	2.6664760
O	-1.5132210	1.8546030	1.5548810
C	-2.6112940	0.7498660	4.6715790
O	-2.6798830	-0.0869970	5.5511060
F	6.7088050	-1.3655770	0.2026470
F	7.7091390	0.0435650	-1.8840480
F	6.1907650	0.4350710	-4.1030250
F	-4.9545570	0.9567570	1.4266090
F	-6.2281260	3.1464950	0.4889520
F	-5.9707950	3.8485210	-2.1278180

159
S_MajorCl_3_MaruokaCF3_TS_Diffuse.log

N	-0.7908290	-1.5800150	0.3828290
C	-0.5674050	-2.5388820	1.5665730
C	-1.9605760	-2.1810120	-0.4194110
C	0.2756200	-1.9745720	2.6773800
H	-0.1125050	-3.4331250	1.1347350
H	-1.5694750	-2.7927690	1.9198310
C	-1.1303160	-0.1569980	0.8945760
C	0.5010090	-1.4031070	-0.4573800
C	-2.1282130	-1.6241650	-1.8127610
H	-2.8379850	-2.0108730	0.2033110
H	-1.7631550	-3.2553660	-0.4528850
C	1.5669450	-2.5103150	2.9022750
C	-0.2216780	-0.9666800	3.5020060
C	-2.0666460	-0.1230940	2.0711340
H	-1.5296290	0.3817830	0.0359710
H	-0.1631320	0.2898720	1.1323680
C	0.8335270	-2.5747470	-1.3480210
H	1.2668430	-1.1566000	0.2742200
H	0.3386680	-0.4938740	-1.0367560
C	-3.2738240	-0.8508180	-2.2009290
C	-1.1217540	-1.8847910	-2.7379400
C	2.3856670	-2.0010430	3.8788060
H	1.9056530	-3.3417410	2.2952360
C	0.6591250	-0.3418450	4.4515690
C	-1.6346990	-0.5255730	3.3340440
C	-3.3618760	0.4272540	1.9017440
C	2.0211180	-3.3655410	-1.1786860
C	-0.0330600	-2.8511800	-2.4029600
C	-3.2833450	-0.2522560	-3.4455760
C	-4.5140490	-0.7405510	-1.3831890
C	-1.1028170	-1.2162190	-4.0078360
C	1.9776740	-0.8763780	4.6394810
H	3.3663740	-2.4364280	4.0485810
C	0.3051260	0.8338950	5.1711450
C	-2.5679900	-0.5370160	4.4286670
C	-4.2406620	0.5070640	2.9535260
H	-3.6277800	0.8336620	0.9337070
C	2.1851710	-4.4930500	-1.9638680
C	3.1588990	-3.0062450	-0.2812680
C	0.1071570	-4.0507610	-3.1782500
C	-2.2043520	-0.3715810	-4.3505330
H	-4.1557880	0.3168640	-3.7541550
C	-5.1665380	-1.8808090	-0.8940690
C	-5.1090430	0.5135320	-1.1718870
C	-0.0164880	-1.3113050	-4.9213850
C	2.8600360	-0.2503820	5.5600390
C	1.1949870	1.4333410	6.0333230
H	-0.6699900	1.2791220	5.0126100
C	-2.2587090	-1.0913400	5.7029980
C	-3.8858400	-0.0051880	4.2273930
H	-5.2197240	0.9558840	2.8124020
C	1.2333330	-4.8949350	-2.9283240
H	3.0939340	-5.0799760	-1.8629120
C	3.7053240	-3.9979150	0.5480880
C	3.7897440	-1.7515860	-0.3393430
C	-0.8388740	-4.4632930	-4.1580370

C	-2.2041900	0.3117220	-5.5955450
C	-6.3720260	-1.7709550	-0.1986790
H	-4.7468980	-2.8653180	-1.0745640
C	-6.3152150	0.6157340	-0.4796490
H	-4.5954920	1.4031750	-1.5199850
C	-0.0372520	-0.6225650	-6.1128910
H	0.8490410	-1.9095310	-4.6629770
C	2.4818740	0.8829360	6.2402850
H	3.8529950	-0.6716910	5.6946060
H	0.9108650	2.3450320	6.5509880
C	-3.1841350	-1.0924970	6.7222720
H	-1.2801790	-1.5274770	5.8666730
C	-4.8107700	-0.0064990	5.3063500
C	1.3907040	-6.0942880	-3.6743620
C	4.8504870	-3.7496350	1.3029370
H	3.2298810	-4.9714050	0.6052240
C	4.9487710	-1.5209120	0.4035480
H	3.4141140	-0.9663970	-0.9874060
C	-0.6682620	-5.6391590	-4.8538420
H	-1.7105810	-3.8476490	-4.3479610
C	-1.1428660	0.1895600	-6.4607340
H	-3.0527750	0.9441120	-5.8431750
C	-7.0826680	-3.0188790	0.2573560
C	-6.9537500	-0.5230090	0.0141260
C	-6.8989960	1.9717920	-0.1815600
H	0.8088680	-0.6971100	-6.7897490
H	3.1725840	1.3667970	6.9248780
C	-4.4705670	-0.5351840	6.5291210
H	-2.9254960	-1.5281770	7.6832800
H	-5.8003850	0.4119080	5.1403400
C	0.4598230	-6.4612440	-4.6173950
H	2.2588960	-6.7188800	-3.4798480
C	5.3658840	-4.7826850	2.2661590
C	5.4864440	-2.5128220	1.2224900
C	5.6460270	-0.1821570	0.3710240
H	-1.4072070	-5.9405530	-5.5907570
H	-1.1434870	0.7254370	-7.4053100
F	-7.8778380	-3.5197530	-0.7143570
F	-6.2076900	-3.9944900	0.5911580
F	-7.8654650	-2.7862510	1.3310630
H	-7.8906000	-0.4389240	0.5524800
F	-8.2484370	1.9453250	-0.1710120
F	-6.5061290	2.4163160	1.0406720
F	-6.5139060	2.9003980	-1.0801250
H	-5.1883410	-0.5358370	7.3446860
H	0.5838060	-7.3826680	-5.1791390
F	4.9388960	-6.0265660	1.9604240
F	4.9439360	-4.5230190	3.5322770
F	6.7137820	-4.8094370	2.3050290
H	6.3948470	-2.3239800	1.7837290
F	5.3975860	0.5120230	1.5024550
F	6.9924080	-0.3480840	0.3112780
F	5.2774060	0.5644250	-0.6757740
O	0.1838550	2.0178050	-0.6930560
N	-0.2545950	3.3242280	-0.9989920
C	1.4210410	2.1430820	0.0307940

C	0.7403510	4.3299960	-0.5708330
C	-1.6079300	3.4784060	-1.0021370
C	1.8329680	3.4834540	0.0429390
O	1.8841080	1.0731430	0.4440010
H	1.0545950	4.9247920	-1.4403120
H	0.2779050	5.0207110	0.1493260
O	-1.8857190	4.8037450	-1.0871220
O	-2.4297010	2.5622170	-0.9890350
C	2.8326610	4.0873560	0.9135320
C	-3.2597490	5.3104730	-1.1758030
C	3.0028820	5.4834520	0.9706800
C	3.6669520	3.3086510	1.7506250
C	-3.0484210	6.8247550	-1.2636330
C	-4.0337930	4.9484960	0.0963500
C	-3.9359470	4.7903650	-2.4493320
C	3.9419530	6.0680880	1.8206440
H	2.4056190	6.1282660	0.3309240
C	4.5972740	3.9033860	2.5924600
H	3.5683470	2.2313680	1.7243450
H	-2.5361570	7.1954890	-0.3701620
H	-4.0137800	7.3353920	-1.3481910
H	-2.4417830	7.0787160	-2.1385990
H	-5.0423220	5.3737380	0.0523570
H	-3.5260800	5.3599540	0.9755110
H	-4.1186690	3.8682960	0.2133130
H	-3.3801260	5.1170580	-3.3350290
H	-4.9544840	5.1884740	-2.5161530
H	-3.9859010	3.7012610	-2.4485530
C	4.7578450	5.2968050	2.6528510
H	4.0422640	7.1520400	1.8311430
H	5.2172770	3.2663160	3.2230280
C	5.7728490	5.9293910	3.5764420
H	6.7839840	5.5397750	3.3986850
H	5.5413730	5.7376160	4.6331800
H	5.8105150	7.0156990	3.4416760
C	4.2916370	1.2100510	-3.9584200
C	5.0571190	1.3140790	-5.2753050
N	4.0849760	2.5475230	-3.5349650
O	3.8925920	0.2066730	-3.4018280
C	5.7006430	2.7108520	-5.2368060
H	5.7511250	0.4815170	-5.4015700
H	4.3078240	1.2519700	-6.0727920
C	4.8439540	3.5087030	-4.2515850
Cl	3.0211230	2.9896580	-1.8447570
H	5.7697860	3.2175820	-6.2002340
H	6.7106390	2.6706000	-4.8145370
O	4.8364270	4.7036310	-4.0672350

159

S_MajorCl_4_MaruokaCF3_TS_Diffuse.log

C	2.8255590	0.6295770	2.5205290
C	3.0237820	1.9078380	3.0006200
C	1.9800940	2.6671280	3.5760810
C	2.1961830	3.9986280	4.0218210
C	1.1726540	4.7252950	4.5822010
C	-0.1136000	4.1487440	4.7114060
C	-0.3497770	2.8565950	4.2992380

C	0.6914700	2.0687890	3.7352030
C	0.5027480	0.7184870	3.2870930
C	1.5178240	0.0485820	2.6109430
C	1.2076470	-1.3118640	2.0389810
H	2.0918010	-1.7989940	1.6293430
H	0.7869110	-1.9578270	2.8136380
N	0.1762850	-1.3487220	0.8898710
O	2.4615030	1.7894670	-0.6164420
C	1.6354790	2.6922840	-0.6702500
C	1.2885480	3.6620380	-1.6824950
C	0.4508710	4.7081180	-0.9740380
H	1.0476560	5.5823750	-0.6726220
H	-0.4105550	5.0537060	-1.5469400
N	-0.0192940	3.9911810	0.2264180
C	-1.3413060	3.8188810	0.5827600
O	-1.7782580	2.8731740	1.2216200
O	-2.0184430	4.9197340	0.1938590
C	-3.3500550	5.2534940	0.7264920
C	-3.6437840	6.5956260	0.0510860
H	-2.8762550	7.3332390	0.3064030
H	-4.6164380	6.9764960	0.3799150
H	-3.6656430	6.4815840	-1.0373090
C	-3.2645150	5.4114660	2.2476380
H	-4.2328610	5.7434020	2.6387030
H	-3.0021410	4.4640670	2.7232760
H	-2.5120170	6.1618850	2.5134220
C	-4.3789460	4.2025040	0.3088840
H	-4.2175530	3.2624530	0.8368460
H	-5.3873660	4.5644710	0.5396360
H	-4.3205580	4.0161400	-0.7665240
O	0.7871850	2.8404650	0.4345480
C	2.2298170	4.0743120	-2.7390660
C	3.3125280	3.2691530	-3.1454620
C	4.1756100	3.6893770	-4.1516020
C	4.0025130	4.9169440	-4.8072530
C	2.9247680	5.7135500	-4.4082370
C	2.0551830	5.3035520	-3.3990550
H	1.2215210	5.9482160	-3.1345040
H	2.7570050	6.6715450	-4.8964530
C	4.9295220	5.3450940	-5.9207780
H	4.8625170	6.4226540	-6.1057220
H	4.6867710	4.8368330	-6.8640800
H	5.9746970	5.1078350	-5.6889700
H	5.0063170	3.0458930	-4.4363800
H	3.4751890	2.3159190	-2.6572350
C	0.8083760	-0.9544040	-0.4690990
C	1.7220460	-2.0031570	-1.0482380
C	1.2214890	-3.2058120	-1.5485650
C	2.1411800	-4.2179440	-1.9985430
C	1.7325690	-5.5400710	-2.3345890
C	2.6442900	-6.4836280	-2.7515940
C	4.0173420	-6.1596060	-2.8638340
C	4.4516350	-4.8987390	-2.5290670
C	3.5399740	-3.9075140	-2.0746880
C	3.9855410	-2.6187440	-1.6869700
C	3.1044230	-1.7065050	-1.1611470

H	3.4477740	-0.7213420	-0.8695910
H	5.0349070	-2.3610810	-1.7958620
H	5.5058600	-4.6409640	-2.5941720
H	4.7248550	-6.9112370	-3.2028350
H	2.3069140	-7.4877950	-2.9933660
H	0.6860110	-5.8083350	-2.2482080
C	-0.2522540	-3.4206220	-1.5796180
C	-0.9895800	-3.2430320	-0.4118300
C	-0.3090050	-2.8105110	0.8559030
H	-0.9666850	-2.9198080	1.7208630
H	0.5910010	-3.4052490	1.0279040
C	-2.3716630	-3.5464150	-0.3811220
C	-3.0292430	-3.9716960	-1.5069120
C	-2.3443030	-4.0560200	-2.7447060
C	-3.0266050	-4.4139540	-3.9387880
C	-2.3705580	-4.4399960	-5.1473480
C	-0.9982740	-4.0989460	-5.2103690
C	-0.3035050	-3.7715170	-4.0675330
C	-0.9419040	-3.7591240	-2.7954850
H	0.7426720	-3.4991120	-4.1378680
H	-0.4895610	-4.0889540	-6.1702620
H	-2.9042230	-4.7069670	-6.0553820
H	-4.0858920	-4.6517200	-3.8787690
H	-4.0852750	-4.2210980	-1.4616860
H	-2.9092330	-3.4604740	0.5562860
H	1.3462360	-0.0233290	-0.2843520
H	-0.0390020	-0.7432220	-1.1288060
C	-0.9882090	-0.3510630	1.0918740
C	-1.5884690	-0.3890540	2.4776970
C	-0.7981810	0.0300580	3.5465910
C	-1.2192710	-0.1961070	4.9017260
C	-0.3835770	0.0459320	6.0280600
C	-0.8249090	-0.2152510	7.3061310
C	-2.1275180	-0.7214370	7.5313400
C	-2.9588970	-0.9729660	6.4653150
C	-2.5243880	-0.7318410	5.1338160
C	-3.3667280	-0.9781660	4.0257210
C	-2.9386170	-0.8206810	2.7197610
C	-3.9618850	-1.0008530	1.6487370
C	-4.1730910	-0.0266380	0.6582920
C	-5.2166120	-0.1716330	-0.2586580
C	-6.0565630	-1.2842760	-0.2198160
C	-5.8496220	-2.2547400	0.7581920
C	-4.8203610	-2.1100060	1.6875960
H	-4.6774060	-2.8722280	2.4469100
C	-6.6869600	-3.5051020	0.7689080
F	-6.0866680	-4.5072180	0.0737640
F	-6.8810160	-3.9689390	2.0222070
F	-7.8974400	-3.3108770	0.2093290
H	-6.8602220	-1.3908760	-0.9384450
C	-5.5057030	0.9237080	-1.2541510
F	-6.3388110	1.8540090	-0.7319050
F	-6.1092070	0.4396410	-2.3669420
F	-4.3890730	1.5658840	-1.6466510
H	-3.5402740	0.8553980	0.6188790
H	-4.3956670	-1.2703860	4.2173250

H	-3.9592210	-1.3682890	6.6235490
H	-2.4640100	-0.9153410	8.5458680
H	-0.1663590	-0.0323420	8.1505410
H	0.6179140	0.4299170	5.8731080
H	-0.5775510	0.6373230	0.8746820
H	-1.6952840	-0.5964410	0.3015070
H	-1.3464550	2.4409050	4.3801250
H	-0.9255790	4.7359100	5.1310980
H	1.3435460	5.7454590	4.9144320
H	3.1845060	4.4344620	3.8998730
H	4.0179330	2.3440110	2.9533540
C	4.0286450	-0.1159490	2.0507350
C	4.4269770	-1.3014620	2.6840380
C	5.6088430	-1.9469730	2.3157150
C	6.4175060	-1.4179030	1.3116780
C	6.0273960	-0.2365630	0.6797650
C	4.8471090	0.4139280	1.0422330
H	4.5307750	1.3123610	0.5226400
C	6.8400330	0.2961780	-0.4711720
F	6.3615320	-0.1647530	-1.6564490
F	8.1330200	-0.0865310	-0.3961850
F	6.8108170	1.6403580	-0.5320820
H	7.3411830	-1.9109760	1.0336670
C	5.9810600	-3.2421220	2.9888180
F	5.3186450	-4.2894460	2.4449140
F	5.6693170	-3.2260320	4.3047490
F	7.2994240	-3.5071980	2.8841370
H	3.8320730	-1.7056310	3.4975310
C	-3.2941040	-0.3471630	-4.1239660
C	-3.5747920	0.8701410	-5.0104200
H	-4.1635630	-0.7136870	-3.5733650
H	-2.8792500	-1.1963830	-4.6786080
H	-3.5330310	0.6740410	-6.0856520
H	-4.5487430	1.3240840	-4.7961420
N	-1.7780170	1.3692030	-3.5432490
Cl	-0.3143110	2.3797700	-2.6508250
C	-2.4804820	1.8829150	-4.6271520
O	-2.2873220	2.9529220	-5.1669120
C	-2.2231960	0.1446540	-3.1393950
O	-1.8402160	-0.4963990	-2.1614340

147

S_MajorCl_4_MaruokaF3_TS_Diffuse.log

C	2.5010450	-2.1539400	-2.1409480
C	2.4321480	-3.5210970	-1.9624260
C	1.2252630	-4.2426550	-2.0997910
C	1.1671110	-5.6400380	-1.8508370
C	-0.0177540	-6.3259070	-1.9752850
C	-1.1962180	-5.6372290	-2.3498910
C	-1.1669660	-4.2867580	-2.6152760
C	0.0458410	-3.5501990	-2.5151660
C	0.1342360	-2.1408900	-2.7686650
C	1.3067900	-1.4423220	-2.4957320
C	1.2748940	0.0598100	-2.6325990
H	2.2606360	0.5072420	-2.5132790
H	0.8859690	0.3393470	-3.6149440
N	0.3778150	0.8120700	-1.6242760

O	2.1584860	-1.6588830	1.1460860
C	1.2287330	-2.2762990	1.6490800
C	0.8585560	-2.5705120	3.0161770
C	-0.1433270	-3.7026030	2.9099220
H	0.3370160	-4.6894490	2.9889250
H	-0.9611490	-3.6528810	3.6280220
N	-0.6891230	-3.5321570	1.5473360
C	-1.9914920	-3.0865780	1.3230970
O	-2.3271300	-2.3360240	0.4226930
O	-2.7923100	-3.7370030	2.1865980
C	-4.2390650	-3.4729030	2.2907340
C	-4.6761400	-4.4756540	3.3621970
H	-4.4640790	-5.5015480	3.0434690
H	-5.7521350	-4.3829800	3.5434120
H	-4.1501750	-4.2873290	4.3032000
C	-4.9305170	-3.7740420	0.9570510
H	-6.0162950	-3.6997720	1.0860000
H	-4.6179070	-3.0751000	0.1805360
H	-4.6961250	-4.7926450	0.6282010
C	-4.4633570	-2.0380500	2.7695960
H	-4.1202620	-1.3120430	2.0326200
H	-5.5335480	-1.8733410	2.9404440
H	-3.9320670	-1.8549300	3.7089630
O	0.2732170	-2.8147810	0.7787590
C	1.8355040	-2.5506460	4.1184180
C	3.0093490	-1.7719550	4.0730860
C	3.9055090	-1.7691140	5.1362500
C	3.6784570	-2.5270800	6.2943950
C	2.5112130	-3.2956130	6.3422580
C	1.6071280	-3.3075340	5.2812810
H	0.7065900	-3.9090370	5.3692830
H	2.3006380	-3.8952420	7.2256670
C	4.6457350	-2.4894140	7.4544010
H	4.5006600	-3.3435200	8.1246500
H	4.5186460	-1.5783950	8.0552900
H	5.6872420	-2.5039410	7.1116530
H	4.8057940	-1.1608440	5.0661710
H	3.2132630	-1.1773370	3.1907910
C	1.0748410	0.9873640	-0.2520730
C	2.1918830	1.9990750	-0.2701700
C	1.9265790	3.3647790	-0.3721580
C	3.0227350	4.2901220	-0.4903250
C	2.8411050	5.6720920	-0.7823650
C	3.9168480	6.5230920	-0.9004700
C	5.2376090	6.0442640	-0.7295000
C	5.4531910	4.7118730	-0.4673270
C	4.3658960	3.8030140	-0.3576090
C	4.5816660	2.4196970	-0.1329690
C	3.5254340	1.5423070	-0.1150160
H	3.6924070	0.4849440	0.0525550
H	5.5979680	2.0598470	0.0063200
H	6.4633800	4.3269730	-0.3513790
H	6.0770320	6.7283860	-0.8175480
H	3.7503970	7.5717500	-1.1311660
H	1.8373010	6.0552480	-0.9238970
C	0.5091560	3.8217230	-0.4025670

C	-0.3584140	3.2618950	-1.3371910
C	0.1220090	2.1862250	-2.2720790
H	-0.5964790	2.0025680	-3.0736380
H	1.0779180	2.4657350	-2.7206880
C	-1.6819100	3.7455570	-1.4678290
C	-2.1581460	4.7339600	-0.6447200
C	-1.3475550	5.2532390	0.3952730
C	-1.8515720	6.2161650	1.3107350
C	-1.0771340	6.6759360	2.3503360
C	0.2386620	6.1827080	2.5198640
C	0.7633910	5.2663330	1.6365800
C	0.0006850	4.7846210	0.5357620
H	1.7668570	4.8881660	1.7914430
H	0.8375590	6.5246140	3.3593170
H	-1.4757040	7.4058670	3.0495680
H	-2.8713020	6.5706740	1.1814340
H	-3.1713330	5.1091330	-0.7654760
H	-2.3193420	3.3317320	-2.2405030
H	1.4387950	-0.0037910	0.0241900
H	0.2775930	1.2700890	0.4423790
C	-0.9399220	0.0668290	-1.3145870
C	-1.6649640	-0.4231640	-2.5478850
C	-1.0573590	-1.4188720	-3.3090960
C	-1.5701300	-1.7653360	-4.6056470
C	-0.8995120	-2.6493600	-5.4965410
C	-1.4169820	-2.9255950	-6.7425790
C	-2.6370130	-2.3435700	-7.1625960
C	-3.3088630	-1.4811780	-6.3281810
C	-2.7908570	-1.1624570	-5.0437080
C	-3.4747870	-0.2906210	-4.1651630
C	-2.9532260	0.0845910	-2.9409710
C	-3.8076660	0.9064120	-2.0401100
C	-3.9751950	0.5441340	-0.6933160
C	-4.7935930	1.3117880	0.1207000
C	-5.4674800	2.4302800	-0.3607320
C	-5.3230800	2.7620850	-1.7049290
C	-4.5096490	2.0149780	-2.5447960
H	-4.4068460	2.3264240	-3.5788700
H	-3.4893860	-0.3312890	-0.2724870
H	-4.4606720	0.0638590	-4.4538150
H	-4.2444590	-1.0239860	-6.6407490
H	-3.0365710	-2.5766100	-8.1455810
H	-0.8835660	-3.5961020	-7.4104980
H	0.0373280	-3.1005130	-5.1905500
H	-0.6708170	-0.7664330	-0.6621000
H	-1.5158510	0.7736440	-0.7215210
H	-2.0830410	-3.7716220	-2.8780100
H	-2.1354790	-6.1786960	-2.4193430
H	-0.0567810	-7.3925100	-1.7725420
H	2.0755620	-6.1524940	-1.5446070
H	3.3380890	-4.0679680	-1.7162750
C	3.8494440	-1.5236420	-2.0536460
C	4.3802520	-0.8109540	-3.1416850
C	5.6717470	-0.3057110	-3.0758110
C	6.4667170	-0.5038500	-1.9503530
C	5.9377290	-1.2229890	-0.8801820

```
C        4.6458610       -1.7302220       -0.9154270
H        4.2429130       -2.2304750       -0.0418590
H        3.8176140       -0.6713320       -4.0588110
C       -2.9819430        2.7496070        3.2780000
C       -3.3877410        2.1126380        4.6119300
H       -3.8217860        2.9050310        2.5957480
H       -2.4667280        3.7100260        3.3847460
H       -3.1289380        2.7162430        5.4880140
H       -4.4562730        1.8850690        4.6809380
N       -1.8053910        0.7101770        3.5305840
Cl       -0.5193370       -0.7822740        3.2499500
C       -2.5958940        0.7941690        4.6670730
O       -2.6705980       -0.0367520        5.5522950
C       -2.0043600        1.7397180        2.6545440
O       -1.4999970        1.8674700        1.5401790
F       -4.9547120        0.9935110        1.4167250
F       -6.2264650        3.1738980        0.4540140
F       -5.9653010        3.8478100       -2.1695250
F        6.7071280       -1.3954850        0.2055280
F        7.7089830       -0.0064400       -1.8940040
F        6.1876090        0.3742830       -4.1127640
159
S_MajorCl_5_MaruokaCF3_TS_Diffuse.log
C        2.9235260        0.6935400        2.2308140
C        3.2102220        2.0094170        2.5365890
C        2.2381600        2.8901760        3.0659960
C        2.5399370        4.2543920        3.3266460
C        1.5837390        5.0984330        3.8410110
C        0.2817490        4.6110170        4.1078810
C       -0.0363080        3.2912180        3.8776930
C        0.9343480        2.3843290        3.3670130
C        0.6574270        1.0011280        3.0984100
C        1.5985790        0.1999860        2.4592760
C        1.1858310       -1.1975980        2.0685090
H        2.0219580       -1.7825790        1.6846710
H        0.7665470       -1.7206650        2.9314660
N        0.0951820       -1.3034180        0.9758570
O        2.3964220        1.5805230       -0.7715420
C        1.5973280        2.4986230       -0.9029950
C        1.3754650        3.4797910       -1.9318660
C        0.4312700        4.5157710       -1.3467770
H        0.9596540        5.4299690       -1.0366430
H       -0.3930230        4.8013960       -2.0047080
N       -0.1080450        3.8187630       -0.1658610
C       -1.4112850        3.7954710        0.2502480
O       -1.9103880        2.9268750        0.9539160
O       -1.9997600        4.9328560       -0.1807050
C       -3.2869690        5.3973280        0.3584850
C       -3.4778010        6.7349320       -0.3616080
H       -2.6432080        7.4107270       -0.1492240
H       -4.4058160        7.2117830       -0.0291750
H       -3.5337120        6.5843660       -1.4443430
C       -3.1639070        5.6032540        1.8718510
H       -4.0917840        6.0367020        2.2615110
H       -2.9815680        4.6540900        2.3800150
H       -2.3422300        6.2914140        2.0992150
```

C	-4.4074650	4.4242920	-0.0078560
H	-4.3269310	3.4957150	0.5555920
H	-5.3784070	4.8825350	0.2127710
H	-4.3763170	4.1893220	-1.0751540
O	0.6211560	2.6313290	0.0980560
C	2.4460840	3.9297450	-2.8407900
C	3.7621200	3.4396960	-2.7423850
C	4.7671300	3.9106520	-3.5795320
C	4.5085200	4.8858060	-4.5538350
C	3.2005190	5.3770700	-4.6487490
C	2.1873880	4.9113860	-3.8126360
H	1.1794090	5.3022510	-3.9303130
H	2.9668430	6.1344270	-5.3948160
C	5.6012330	5.3680090	-5.4789130
H	5.3096570	6.2875570	-5.9981300
H	5.8369950	4.6189330	-6.2477020
H	6.5313540	5.5700710	-4.9330640
H	5.7752140	3.5124760	-3.4726630
H	3.9722970	2.6900220	-1.9869090
C	0.6779930	-1.1207790	-0.4478710
C	1.4795330	-2.2992900	-0.9337530
C	0.8624710	-3.5044580	-1.2688940
C	1.6730930	-4.6375310	-1.6284290
C	1.1425690	-5.9484810	-1.7938100
C	1.9548940	-7.0084690	-2.1278200
C	3.3441600	-6.8200310	-2.3223030
C	3.8948940	-5.5721990	-2.1507440
C	3.0876680	-4.4608680	-1.7857660
C	3.6546330	-3.1808170	-1.5652050
C	2.8745160	-2.1400820	-1.1259620
H	3.3105460	-1.1623360	-0.9609260
H	4.7167160	-3.0329130	-1.7319960
H	4.9629870	-5.4153820	-2.2800040
H	3.9718820	-7.6639510	-2.5946700
H	1.5257060	-8.0003060	-2.2397600
H	0.0823280	-6.1141590	-1.6417830
C	-0.6217690	-3.5966390	-1.2083120
C	-1.2735990	-3.2322810	-0.0338760
C	-0.4882920	-2.7181040	1.1407090
H	-1.0997330	-2.6793420	2.0447260
H	0.3760080	-3.3575510	1.3343100
C	-2.6711250	-3.4166810	0.0972050
C	-3.4234210	-3.9078320	-0.9400090
C	-2.8176600	-4.1874650	-2.1909530
C	-3.5894950	-4.6231260	-3.3024330
C	-3.0035280	-4.8415420	-4.5276930
C	-1.6151470	-4.6213980	-4.6937910
C	-0.8368330	-4.2209180	-3.6313400
C	-1.4020970	-4.0123640	-2.3419080
H	0.2216210	-4.0439100	-3.7803150
H	-1.1603420	-4.7638550	-5.6699260
H	-3.6044720	-5.1671960	-5.3723340
H	-4.6587980	-4.7655000	-3.1668060
H	-4.4921190	-4.0591880	-0.8182470
H	-3.1441680	-3.1821880	1.0435110
H	1.2779490	-0.2122440	-0.4040210

H	-0.1860010	-0.9225900	-1.0898360
C	-0.9923690	-0.2131590	1.1022560
C	-1.5303550	-0.0529730	2.5041480
C	-0.6660630	0.4267280	3.4892750
C	-1.0410440	0.3854480	4.8750840
C	-0.1429220	0.6944050	5.9350830
C	-0.5446660	0.6104040	7.2496730
C	-1.8666100	0.2247850	7.5775840
C	-2.7583110	-0.0856600	6.5778540
C	-2.3681460	-0.0265150	5.2129260
C	-3.2742020	-0.3330980	4.1710940
C	-2.8947750	-0.3534740	2.8413740
C	-3.9629940	-0.5839880	1.8267720
C	-4.1274980	0.2720920	0.7262790
C	-5.1814250	0.0670800	-0.1687290
C	-6.0863470	-0.9755820	0.0153050
C	-5.9364880	-1.8188300	1.1166820
C	-4.8901560	-1.6229750	2.0149170
H	-4.7812730	-2.2933280	2.8605010
C	-6.8958990	-2.9664980	1.2806820
F	-6.7490700	-3.8764020	0.2817620
F	-6.7201670	-3.6238570	2.4452620
F	-8.1799010	-2.5520570	1.2334740
H	-6.8957940	-1.1303100	-0.6898440
C	-5.4048460	1.0256910	-1.3111410
F	-6.2547380	2.0199340	-0.9563610
F	-5.9642330	0.4032930	-2.3792210
F	-4.2659450	1.6054140	-1.7284000
H	-3.4561370	1.1151190	0.5842970
H	-4.3111970	-0.5272310	4.4318680
H	-3.7746250	-0.3891660	6.8161170
H	-2.1708050	0.1699770	8.6190770
H	0.1600520	0.8410120	8.0436840
H	0.8739590	0.9878050	5.7004740
H	-0.5318480	0.7124020	0.7517190
H	-1.7500260	-0.5006820	0.3763080
H	-1.0450670	2.9428100	4.0608270
H	-0.4775930	5.2881130	4.4894000
H	1.8197360	6.1418090	4.0310070
H	3.5386020	4.6193640	3.0999520
H	4.2193800	2.3815990	2.3826780
C	4.0773490	-0.1728590	1.8395500
C	4.5029700	-1.1884440	2.7097750
C	5.6644230	-1.9183300	2.4505370
C	6.4355180	-1.6389820	1.3224380
C	6.0105970	-0.6405740	0.4474520
C	4.8415890	0.0836660	0.6916590
H	4.5068930	0.7831920	-0.0653200
C	6.7831630	-0.3820850	-0.8211760
F	6.3628470	-1.1883040	-1.8309510
F	8.1025810	-0.6186630	-0.6598190
F	6.6403030	0.8872130	-1.2508610
H	7.3533280	-2.1842110	1.1345190
C	6.0725860	-3.0372820	3.3732160
F	5.5743300	-4.2255680	2.9589850
F	5.6240670	-2.8407060	4.6328820

F	7.4144180	-3.1721340	3.4330560
H	3.9438400	-1.3846450	3.6192340
C	-3.2436190	-0.5196510	-4.2981980
C	-2.9035860	0.1499330	-5.6326630
H	-4.2167650	-0.2003210	-3.9060850
H	-3.2381030	-1.6128720	-4.3122110
H	-2.4015240	-0.5389260	-6.3244690
H	-3.7550630	0.5842540	-6.1647450
N	-1.5008530	1.0342100	-3.9407970
Cl	-0.0983460	2.1193130	-3.0385390
C	-1.8965360	1.2520890	-5.2557660
O	-1.5051380	2.1380430	-5.9870900
C	-2.1704460	0.0060100	-3.3337160
O	-1.9903830	-0.4253270	-2.1975450

147

S_MajorCl_5_MaruokaF3_TS_Diffuse.log

C	2.6480560	-1.3960740	-2.4668470
C	2.9429050	-2.7345010	-2.3466090
C	1.9382550	-3.7293950	-2.3576590
C	2.2565830	-5.1009680	-2.1730570
C	1.2706300	-6.0592580	-2.1885000
C	-0.0790880	-5.6798900	-2.3816190
C	-0.4161770	-4.3591560	-2.5775690
C	0.5814560	-3.3455390	-2.5891610
C	0.2841570	-1.9546520	-2.7784650
C	1.2787240	-0.9952940	-2.6109910
C	0.8788620	0.4554750	-2.6351710
H	1.7433520	1.1178580	-2.5945020
H	0.3150020	0.6786020	-3.5441780
N	-0.0469960	0.9294590	-1.4893460
O	2.5049820	-1.3641410	0.8208880
C	1.7189310	-2.1045160	1.3966180
C	1.6375190	-2.6110800	2.7461940
C	0.6184640	-3.7416720	2.6959840
H	1.1174900	-4.7202570	2.6163670
H	-0.0781930	-3.7762610	3.5322750
N	-0.1241220	-3.4604780	1.4597430
C	-1.4878110	-3.3638140	1.3294140
O	-2.0627290	-2.7628630	0.4330940
O	-2.0527870	-4.1280010	2.2916430
C	-3.4979330	-4.4004480	2.3295830
C	-3.6306410	-5.2872980	3.5707180
H	-3.0091370	-6.1831940	3.4733660
H	-4.6727270	-5.5987400	3.6997590
H	-3.3171840	-4.7449610	4.4684960
C	-3.9114070	-5.1629920	1.0667430
H	-4.9739830	-5.4243830	1.1252880
H	-3.7499380	-4.5540130	0.1749760
H	-3.3362340	-6.0907450	0.9734590
C	-4.2848450	-3.0994580	2.5078590
H	-4.3134490	-2.5259060	1.5823150
H	-5.3136010	-3.3312910	2.8070480
H	-3.8330450	-2.4823980	3.2908910
O	0.6144930	-2.5534830	0.6536480
C	2.8252530	-2.7989280	3.6019630
C	4.1190310	-2.4429300	3.1773590

C	5.2305450	-2.7036880	3.9696590
C	5.1060250	-3.3112540	5.2275340
C	3.8202100	-3.6650020	5.6509490
C	2.7023240	-3.4177850	4.8582100
H	1.7200570	-3.7016040	5.2263140
H	3.6894650	-4.1438320	6.6192820
C	6.3142260	-3.5542700	6.0996920
H	6.0927100	-4.2677080	6.9003560
H	6.6594150	-2.6266520	6.5748150
H	7.1562630	-3.9515270	5.5210180
H	6.2183070	-2.4280530	3.6051130
H	4.2238600	-1.9935470	2.1986210
C	0.7091300	1.1760030	-0.1614430
C	1.5827430	2.4018710	-0.1660420
C	1.0518920	3.6911670	-0.2000230
C	1.9435150	4.8134210	-0.3390650
C	1.4844610	6.1378600	-0.5908840
C	2.3685420	7.1830980	-0.7351580
C	3.7636990	6.9685200	-0.6291370
C	4.2459190	5.7004590	-0.4053430
C	3.3616700	4.5954560	-0.2721590
C	3.8509520	3.2777360	-0.0824670
C	2.9841740	2.2145790	-0.0559780
H	3.3554150	1.2071370	0.0883980
H	4.9209340	3.1118100	0.0184810
H	5.3156890	5.5181300	-0.3391230
H	4.4492990	7.8044260	-0.7366180
H	1.9915850	8.1822130	-0.9364430
H	0.4201260	6.3209090	-0.6837350
C	-0.4238520	3.8657780	-0.1041410
C	-1.2505540	3.1569870	-0.9733120
C	-0.6578890	2.2422610	-2.0116030
H	-1.4065940	1.9436190	-2.7482210
H	0.1601970	2.7437010	-2.5341770
C	-2.6510110	3.3646400	-0.9616630
C	-3.2303290	4.2393950	-0.0775480
C	-2.4411940	4.9101080	0.8913140
C	-3.0346630	5.7605490	1.8646940
C	-2.2716370	6.3583080	2.8408240
C	-0.8778590	6.1176840	2.8899380
C	-0.2701420	5.3156450	1.9492840
C	-1.0208000	4.7048880	0.9039030
H	0.7949410	5.1302170	2.0150260
H	-0.2822930	6.5630800	3.6821380
H	-2.7367330	6.9996870	3.5845460
H	-4.1103020	5.9152950	1.8307070
H	-4.3042500	4.4067360	-0.0942090
H	-3.2676600	2.8337280	-1.6770670
H	1.3019780	0.2811620	0.0177270
H	-0.0725880	1.2242560	0.6008500
C	-1.1381930	-0.1085820	-1.1274280
C	-1.8386970	-0.7056740	-2.3243930
C	-1.0984620	-1.5347350	-3.1634210
C	-1.6417580	-1.9790410	-4.4190500
C	-0.8817190	-2.6856080	-5.3932160
C	-1.4436600	-3.0673330	-6.5912790

C	-2.7975640	-2.7733520	-6.8803280
C	-3.5583880	-2.0840450	-5.9659660
C	-3.0009940	-1.6608870	-4.7289370
C	-3.7701990	-0.9523440	-3.7788250
C	-3.2333440	-0.4715280	-2.5989700
C	-4.1672370	0.1814700	-1.6377030
C	-4.1723890	-0.1716730	-0.2786050
C	-5.0778050	0.4277150	0.5854220
C	-6.0098300	1.3577880	0.1321270
C	-6.0173960	1.6835050	-1.2219820
C	-5.1138520	1.1118450	-2.1053920
H	-5.1448020	1.4153880	-3.1464610
H	-3.4894970	-0.9133910	0.1207810
H	-4.8291410	-0.8101790	-3.9770190
H	-4.5975440	-1.8441640	-6.1777340
H	-3.2291820	-3.0875610	-7.8265100
H	-0.8413370	-3.5986720	-7.3227650
H	0.1573810	-2.9166930	-5.1908710
H	-0.6364590	-0.8810610	-0.5399690
H	-1.8032630	0.4240970	-0.4536750
H	-1.4564770	-4.0806360	-2.6953740
H	-0.8575450	-6.4374360	-2.3653760
H	1.5220530	-7.1054890	-2.0383020
H	3.2949090	-5.3759240	-2.0060690
H	3.9806010	-3.0417660	-2.2494580
C	3.7860560	-0.4347310	-2.5785490
C	3.9919060	0.2558790	-3.7844120
C	5.0667360	1.1211180	-3.9125890
C	5.9549450	1.3243330	-2.8597980
C	5.7450110	0.6324860	-1.6703090
C	4.6879750	-0.2498490	-1.5199360
H	4.5195310	-0.7260700	-0.5618930
H	3.3357670	0.1123190	-4.6359750
C	-2.7893740	2.0306030	3.7808170
C	-2.0961660	2.2840210	5.1265530
H	-3.7727280	1.5513910	3.8824800
H	-2.9237070	2.9229380	3.1607240
H	-1.6475990	3.2860040	5.1819100
H	-2.7402750	2.1657550	6.0070360
N	-0.9145080	0.5992620	3.9471870
Cl	0.3636070	-0.8419190	3.4484210
C	-0.9624530	1.2409740	5.1777200
O	-0.2371340	1.0311030	6.1308620
C	-1.8605170	1.0436740	3.0600150
O	-1.9657940	0.7170410	1.8786180
F	6.5731950	0.8785540	-0.6408400
F	6.9772930	2.1777540	-2.9841700
F	5.2725870	1.7826850	-5.0625650
F	-5.0943210	0.1016960	1.8872080
F	-6.8780050	1.9304800	0.9755730
F	-6.9130760	2.5880530	-1.6564750

159

S_MajorCl_6_MaruokaCF3_TS_Diffuse.log

C	2.8143730	-0.5374270	2.5434590
C	3.0674610	0.4493300	3.4749000
C	2.0528380	0.9896530	4.2970970

C	2.3199260	2.0501140	5.2034030
C	1.3194590	2.5669140	5.9923060
C	0.0072550	2.0437720	5.9032290
C	-0.2778060	1.0038090	5.0473820
C	0.7367490	0.4359990	4.2285590
C	0.4938620	-0.6381360	3.3103310
C	1.4782580	-1.0429170	2.4147390
C	1.0995640	-2.0527050	1.3599120
H	1.9572640	-2.3710370	0.7687150
H	0.6523080	-2.9367660	1.8215100
N	0.0636560	-1.5834450	0.3139920
O	2.5948230	1.7488870	0.1505980
C	1.8523970	2.6698020	0.4688560
C	1.6118050	3.9847180	-0.0812710
C	0.8881640	4.7376440	1.0173830
H	1.5814920	5.3101770	1.6516300
H	0.1025260	5.4064440	0.6672050
N	0.2759720	3.6534350	1.8132960
C	-1.1007010	3.4357290	1.8341280
O	-1.6365620	2.3404200	1.8459910
O	-1.6921710	4.6371680	1.9722440
C	-3.1487750	4.8258240	1.8329990
C	-3.3089110	6.3287760	2.0755530
H	-2.7323110	6.9009240	1.3422710
H	-2.9641350	6.6003390	3.0788750
H	-4.3628920	6.6107800	1.9816630
C	-3.8971970	4.0269960	2.9051290
H	-3.8020790	2.9536390	2.7414570
H	-3.5062730	4.2671000	3.9002250
H	-4.9592920	4.2954870	2.8813780
C	-3.5649520	4.4542610	0.4102680
H	-4.6409020	4.6163260	0.2862990
H	-3.0340960	5.0661100	-0.3256110
H	-3.3514090	3.4060830	0.2010420
O	1.0143440	2.4597100	1.5692400
C	2.5997950	4.6823230	-0.9229040
C	3.5882020	3.9971120	-1.6577280
C	4.4988130	4.6901830	-2.4476800
C	4.4686730	6.0883420	-2.5545680
C	3.4850650	6.7683210	-1.8297430
C	2.5687590	6.0838270	-1.0329510
H	1.8126590	6.6533630	-0.4995940
H	3.4297830	7.8535240	-1.8894750
C	5.4454750	6.8247010	-3.4412820
H	6.4588640	6.4153280	-3.3520330
H	5.4899750	7.8898310	-3.1898200
H	5.1623000	6.7511850	-4.5003140
H	5.2538840	4.1301020	-2.9967370
H	3.6406160	2.9169630	-1.5942790
C	0.7098730	-0.6753340	-0.7626850
C	1.5902950	-1.4148920	-1.7373700
C	1.0507300	-2.2801760	-2.6898280
C	1.9356090	-3.0408540	-3.5328700
C	1.4818780	-4.0844540	-4.3890650
C	2.3616310	-4.7967390	-5.1727500
C	3.7460780	-4.5037960	-5.1518240

C	4.2232000	-3.5161470	-4.3227140
C	3.3448900	-2.7760930	-3.4853720
C	3.8339670	-1.7857050	-2.5967460
C	2.9832140	-1.1465110	-1.7288910
H	3.3586950	-0.3860970	-1.0551180
H	4.8928420	-1.5447730	-2.5969760
H	5.2862980	-3.2915720	-4.2839890
H	4.4282030	-5.0676210	-5.7821260
H	1.9898840	-5.5938290	-5.8107980
H	0.4258740	-4.3272880	-4.4133710
C	-0.4289910	-2.4160730	-2.7885720
C	-1.1591470	-2.7244970	-1.6437460
C	-0.4635400	-2.8846200	-0.3210330
H	-1.1228530	-3.3290200	0.4277970
H	0.4182860	-3.5205000	-0.4263970
C	-2.5509270	-2.9703990	-1.7249090
C	-3.2225230	-2.8600270	-2.9156690
C	-2.5412080	-2.4336920	-4.0829770
C	-3.2351330	-2.2307450	-5.3062000
C	-2.5795160	-1.7643940	-6.4216280
C	-1.1957930	-1.4741250	-6.3545410
C	-0.4906290	-1.6834730	-5.1905950
C	-1.1294040	-2.1885930	-4.0236140
H	0.5646150	-1.4411260	-5.1537370
H	-0.6862150	-1.0765200	-7.2278030
H	-3.1216600	-1.6048200	-7.3496760
H	-4.3022170	-2.4372680	-5.3372200
H	-4.2856740	-3.0770610	-2.9650480
H	-3.0852390	-3.2755640	-0.8327800
H	1.2805440	0.0729000	-0.2110360
H	-0.1239020	-0.1775190	-1.2678420
C	-1.0790840	-0.7376310	0.9262100
C	-1.6445880	-1.3170000	2.2034030
C	-0.8219250	-1.3446980	3.3276390
C	-1.2082790	-2.0729430	4.5053270
C	-0.3434460	-2.2726210	5.6180730
C	-0.7524790	-3.0055240	6.7098920
C	-2.0489950	-3.5714600	6.7563170
C	-2.9075890	-3.4046670	5.6953100
C	-2.5071030	-2.6685640	4.5476500
C	-3.3779100	-2.4802230	3.4504440
C	-2.9870060	-1.8229000	2.2981560
C	-4.0511150	-1.5768990	1.2816420
C	-4.3438970	-0.2818190	0.8285410
C	-5.4494150	-0.0594380	0.0013320
C	-6.2582590	-1.1148440	-0.4130890
C	-5.9616350	-2.4063920	0.0218180
C	-4.8812710	-2.6325280	0.8719670
H	-4.6771510	-3.6400730	1.2201540
C	-6.7683480	-3.5726730	-0.4823080
F	-8.0252700	-3.2154400	-0.8122200
F	-6.2061220	-4.1133920	-1.5949260
F	-6.8468770	-4.5632440	0.4316910
H	-7.1138190	-0.9327660	-1.0512570
C	-5.7848940	1.3520310	-0.4089550
F	-5.8985830	2.1585720	0.6701880

F	-6.9463970	1.4222780	-1.0912450
F	-4.8243500	1.8848900	-1.1984780
H	-3.7329850	0.5572040	1.1521090
H	-4.4007150	-2.8368090	3.5379730
H	-3.9038710	-3.8393690	5.7142730
H	-2.3592970	-4.1409820	7.6277600
H	-0.0722820	-3.1524150	7.5440890
H	0.6542420	-1.8505320	5.5984510
H	-0.6634350	0.2579400	1.0993280
H	-1.8134420	-0.6509450	0.1263940
H	-1.2921370	0.6295810	4.9742860
H	-0.7845890	2.4740670	6.5098470
H	1.5287930	3.3859010	6.6746520
H	3.3275960	2.4551950	5.2489860
H	4.0823750	0.8192490	3.5927570
C	3.9849630	-1.1076860	1.8173620
C	4.2892660	-2.4735530	1.9084050
C	5.4361050	-2.9928070	1.3058200
C	6.3045170	-2.1576740	0.6045670
C	6.0093220	-0.7973840	0.5140270
C	4.8644030	-0.2712470	1.1143000
H	4.6192990	0.7797820	1.0045930
C	6.8816420	0.1038820	-0.3201460
F	6.3830550	0.2372100	-1.5769710
F	8.1355770	-0.3813980	-0.4459210
F	6.9693850	1.3425230	0.1997120
H	7.1941630	-2.5601350	0.1352250
C	5.7678050	-4.4516940	1.4798020
F	4.6506810	-5.2142640	1.5137060
F	6.4297390	-4.6769050	2.6376230
F	6.5474870	-4.9140470	0.4804050
H	3.6402460	-3.1357860	2.4731040
C	-3.2361810	1.7018890	-3.8702050
C	-3.5129260	3.1998990	-4.0350440
H	-4.0977520	1.1168500	-3.5403310
H	-2.8507120	1.2298430	-4.7808550
H	-3.4557070	3.5624820	-5.0657540
H	-4.4899570	3.4960410	-3.6382350
N	-1.6885580	2.9035890	-2.5442430
Cl	-0.1198490	3.3435540	-1.4005360
C	-2.4232390	3.8862350	-3.1910780
O	-2.2618790	5.0874510	-3.0900340
C	-2.1362410	1.6372910	-2.8000690
O	-1.7399810	0.5949910	-2.2842990

147
S_MajorCl_6_MaruokaF3_TS_Diffuse.log

C	2.6765300	-1.0762800	-2.6392790
C	3.1764850	-2.3600570	-2.5572760
C	2.3413090	-3.4993500	-2.5691000
C	2.8750070	-4.8070730	-2.4158070
C	2.0499410	-5.9065150	-2.4224220
C	0.6533600	-5.7395950	-2.5810850
C	0.1091070	-4.4861150	-2.7465020
C	0.9345480	-3.3279180	-2.7578580
C	0.4223140	-1.9964670	-2.9082140
C	1.2593500	-0.8927750	-2.7552250

C	0.6295390	0.4781950	-2.7732590
H	1.3723150	1.2745250	-2.7619350
H	0.0137600	0.5950280	-3.6685170
N	-0.3184390	0.8116750	-1.5980990
O	2.7052020	-1.0364240	0.6275740
C	2.1350830	-1.9295090	1.2360750
C	2.2630480	-2.4393040	2.5861730
C	1.5182100	-3.7593580	2.5792920
H	2.1826290	-4.6109210	2.3673680
H	0.9605630	-3.9688710	3.4913950
N	0.5699610	-3.5995300	1.4611820
C	-0.8038920	-3.4464910	1.6345080
O	-1.5170840	-2.7881770	0.8987860
O	-1.1927660	-4.2335740	2.6539740
C	-2.5654050	-4.1899470	3.1998190
C	-2.4932180	-5.2060260	4.3429660
H	-1.7402500	-4.9056990	5.0789270
H	-2.2331500	-6.2006000	3.9642180
H	-3.4636790	-5.2690800	4.8474950
C	-3.5743020	-4.6466680	2.1408020
H	-3.6350240	-3.9380180	1.3147800
H	-3.2925890	-5.6283220	1.7430730
H	-4.5657680	-4.7372070	2.5999170
C	-2.8532110	-2.7866240	3.7373940
H	-3.8464600	-2.7701810	4.2011580
H	-2.1114390	-2.5101210	4.4929140
H	-2.8300330	-2.0389450	2.9442500
O	1.1131900	-2.6282200	0.5670200
C	3.5031720	-2.2911520	3.3664780
C	4.3881190	-1.2110790	3.1697860
C	5.5463160	-1.0885930	3.9284510
C	5.8807510	-2.0236720	4.9199620
C	4.9980280	-3.0882360	5.1240240
C	3.8342910	-3.2220830	4.3665980
H	3.1714080	-4.0582510	4.5721990
H	5.2187350	-3.8265450	5.8926020
C	7.1501670	-1.8835440	5.7276360
H	8.0410250	-2.1072090	5.1244620
H	7.1553960	-2.5651900	6.5848510
H	7.2722430	-0.8631230	6.1114180
H	6.2062350	-0.2406450	3.7519600
H	4.1524430	-0.4681720	2.4170660
C	0.4502170	1.2399770	-0.3269450
C	1.0947340	2.5992170	-0.4365250
C	0.3336900	3.7716420	-0.4487580
C	0.9882720	5.0322910	-0.6853640
C	0.2746820	6.2423530	-0.9209150
C	0.9363210	7.4264900	-1.1566440
C	2.3507670	7.4732600	-1.1654990
C	3.0737790	6.3210300	-0.9644260
C	2.4213280	5.0781110	-0.7395430
C	3.1583510	3.8768640	-0.5838740
C	2.5105020	2.6716290	-0.4655240
H	3.0754320	1.7527950	-0.3613190
H	4.2445240	3.9195840	-0.5830450
H	4.1605550	6.3381610	-0.9884760

H	2.8601330	8.4161800	-1.3440450
H	0.3667620	8.3330760	-1.3422430
H	-0.8089650	6.2254350	-0.9268940
C	-1.1420060	3.6823060	-0.2570830
C	-1.8709210	2.8042000	-1.0563640
C	-1.1791350	1.9802850	-2.1092460
H	-1.8971790	1.5349030	-2.8007080
H	-0.4895030	2.6054250	-2.6808790
C	-3.2826370	2.7491930	-0.9649580
C	-3.9632930	3.5256530	-0.0617710
C	-3.2602930	4.3669760	0.8362340
C	-3.9497420	5.1228030	1.8231340
C	-3.2641320	5.8984500	2.7277060
C	-1.8502330	5.9395040	2.6875610
C	-1.1534850	5.2332460	1.7321470
C	-1.8290350	4.4403990	0.7589260
H	-0.0735170	5.2722380	1.7321740
H	-1.3076350	6.5273330	3.4227940
H	-3.8013120	6.4649650	3.4831850
H	-5.0347060	5.0616370	1.8569980
H	-5.0483530	3.4864530	-0.0107390
H	-3.8286030	2.0916220	-1.6309930
H	1.1812410	0.4551800	-0.1447120
H	-0.2882760	1.1968890	0.4786590
C	-1.1712120	-0.4071850	-1.1627710
C	-1.8425630	-1.1164280	-2.3176130
C	-1.0258640	-1.7956230	-3.2203650
C	-1.5581750	-2.3020230	-4.4552690
C	-0.7467010	-2.8488990	-5.4887410
C	-1.3053940	-3.2974510	-6.6648500
C	-2.7047540	-3.2344680	-6.8701130
C	-3.5172530	-2.7044410	-5.8957440
C	-2.9677990	-2.2156420	-4.6794710
C	-3.7873520	-1.6684320	-3.6668500
C	-3.2715740	-1.1207550	-2.5062340
C	-4.2507010	-0.6518190	-1.4837380
C	-4.1357710	-1.0263910	-0.1343010
C	-5.1106290	-0.6248970	0.7700860
C	-6.2103850	0.1335810	0.3755260
C	-6.3246190	0.4868450	-0.9656620
C	-5.3665820	0.1023690	-1.8924380
H	-5.4911040	0.4144010	-2.9237850
H	-3.3128190	-1.6365570	0.2268290
H	-4.8647540	-1.7097600	-3.8002820
H	-4.5926830	-2.6429960	-6.0433420
H	-3.1314890	-3.5995480	-7.8000660
H	-0.6659540	-3.7029590	-7.4438800
H	0.3267800	-2.9014050	-5.3493120
H	-0.4819600	-1.0773180	-0.6408560
H	-1.8663390	-0.0082730	-0.4286080
H	-0.9642650	-4.3763040	-2.8448320
H	0.0033570	-6.6097800	-2.5619290
H	2.4639180	-6.9027560	-2.2942040
H	3.9476610	-4.9184410	-2.2791610
H	4.2507020	-2.5051460	-2.4861510
C	3.6660360	0.0372800	-2.6938980

C	3.6940920	0.9234400	-3.7836020
C	4.6777260	1.9008890	-3.8565570
C	5.6546590	2.0153310	-2.8712880
C	5.6276190	1.1239990	-1.8002220
C	4.6503150	0.1431700	-1.6973010
H	4.6208450	-0.4977610	-0.8225640
H	2.9830370	0.8447390	-4.5994420
C	-2.3146070	2.3166430	4.0325280
C	-1.6931000	2.3410930	5.4308920
H	-3.4022150	2.1902770	4.0292830
H	-2.0778240	3.1899830	3.4186850
H	-1.0654460	3.2218710	5.6083410
H	-2.4164880	2.2943410	6.2508390
N	-0.8205210	0.4887290	4.2215490
Cl	0.6896440	-0.9695510	3.5152860
C	-0.7830510	1.0965970	5.4738160
O	-0.1347380	0.7314670	6.4339300
C	-1.6570840	1.1148540	3.3416150
O	-1.8520080	0.8072550	2.1650600
F	-7.3801150	1.2276410	-1.3466890
F	-7.1367750	0.5143360	1.2640520
F	-5.0299830	-0.9807890	2.0599240
F	4.7138710	2.7506370	-4.8962190
F	6.5939220	2.9674190	-2.9463710
F	6.5631700	1.2562020	-0.8468970
